# Plant Aquaporins: Genome-Wide Identification, Transcriptomics, Proteomics, and Advanced Analytical Tools

**DOI:** 10.3389/fpls.2016.01896

**Published:** 2016-12-20

**Authors:** Rupesh K. Deshmukh, Humira Sonah, Richard R. Bélanger

**Affiliations:** Département de Phytologie–Faculté des Sciences de l'Agriculture et de l'Alimentation, Université LavalQuébec, QC, Canada

**Keywords:** plant aquaporin, omic scale analysis, analytical approaches, yeast assay, *Xenopus oocytes* assay

## Abstract

Aquaporins (AQPs) are channel-forming integral membrane proteins that facilitate the movement of water and many other small molecules. Compared to animals, plants contain a much higher number of AQPs in their genome. Homology-based identification of AQPs in sequenced species is feasible because of the high level of conservation of protein sequences across plant species. Genome-wide characterization of AQPs has highlighted several important aspects such as distribution, genetic organization, evolution and conserved features governing solute specificity. From a functional point of view, the understanding of AQP transport system has expanded rapidly with the help of transcriptomics and proteomics data. The efficient analysis of enormous amounts of data generated through omic scale studies has been facilitated through computational advancements. Prediction of protein tertiary structures, pore architecture, cavities, phosphorylation sites, heterodimerization, and co-expression networks has become more sophisticated and accurate with increasing computational tools and pipelines. However, the effectiveness of computational approaches is based on the understanding of physiological and biochemical properties, transport kinetics, solute specificity, molecular interactions, sequence variations, phylogeny and evolution of aquaporins. For this purpose, tools like *Xenopus* oocyte assays, yeast expression systems, artificial proteoliposomes, and lipid membranes have been efficiently exploited to study the many facets that influence solute transport by AQPs. In the present review, we discuss genome-wide identification of AQPs in plants in relation with recent advancements in analytical tools, and their availability and technological challenges as they apply to AQPs. An exhaustive review of omics resources available for AQP research is also provided in order to optimize their efficient utilization. Finally, a detailed catalog of computational tools and analytical pipelines is offered as a resource for AQP research.

## Introduction

Aquaporins (AQPs) are channel-forming proteins that facilitate selective transport of water and many other small molecules like urea, silicon (Si) in the form of silicic acid, boron (B) in the form of boric acid, and CO_2_ across biological membranes. AQPs are present in almost all living organisms including eukaryotes and prokaryotes (Quigley et al., 2001; Tanghe et al., 2006; Benga and Huber, 2012; Benga, 2013). In animals, minor defects or changes in AQP configuration are known to cause many diseases such as hereditary nephrogenic diabetes insipidus, congenital cataracts and, more commonly, the inability to concentrate solutes in urine (Verkman, 2009; Benga and Huber, 2012). Similarly, plant AQPs have an important role in regulating the overall development of a plant, namely in the maintenance of hydraulic status under extreme conditions. As early as 1986, Benga et al. in a pioneer effort, reported the role of proteins in water transport (Benga et al., 1986a,b). Subsequently, Peter Agre's team proved through cRNA expression studies that those proteins, named aquaporin-1, were specific water channels (Preston et al., 1992), and Agre was awarded the Nobel Prize in chemistry in 2003 for his discovery (Agre, 2004). These findings have sparked a veritable explosion of work that has enhanced our understanding of the importance of AQPs in animals as well as in plants (Papadopoulos and Verkman, 2013; Deshmukh et al., 2015; Kitchen et al., 2015; Maurel et al., 2015; Kirscht et al., 2016; Srivastava et al., 2016).

AQPs from diverse origins have characteristic hourglass-like structures with six transmembranes (TM) alpha helices and two half TM alpha-helices with conserved NPA domains (asparagine–proline–alanine) (Jung et al., 1994; Murata et al., 2000). The two half alpha helices form a constraint in the center of the pore that regulates the selective transport of solutes through the pore. Another constraint known as the aromatic arginine (ar/R) selectivity filter (SF), formed mostly with four amino acid residues, plays also a major role in solute selectivity (Murata et al., 2000; Törnroth-Horsefield et al., 2006). Based on their phylogenetic distribution, plant AQPs are generally categorized into five major sub-families: plasma membrane intrinsic proteins (PIP), nodulin 26-like intrinsic proteins (NIPs), tonoplast intrinsic proteins (TIPs), small intrinsic proteins (SIPs), and uncharacterized intrinsic proteins (XIPs) (Quigley et al., 2001; Deshmukh et al., 2015). The phylogenic classification of each group is very well aligned with the functionality and characteristic features of AQPs (Grégoire et al., 2012; Deshmukh et al., 2015).

Availability of whole genome sequences for animal and plant species has facilitated genome-wide identification and classification of AQPs. For instance, compared to animals, plants have a larger number of AQPs ranging from 23 in *Selaginella moellendorffii* to 72 in soybean (Deshmukh et al., 2015). Apart from identifying novel genes, genome-wide studies have contributed to a better understanding of the molecular evolution of the AQP gene families (Gupta and Sankararamakrishnan, 2009; Deshmukh and Bélanger, 2016). Precise identification of conserved features along with their functional relevance has progressed rapidly with the availability of AQP sequences from many plant species. Similarly, transcriptome profiling of AQPs conducted in several plant species has helped to determine that AQPs have expression specific to tissue, growth stage, or environmental conditions (Gupta and Sankararamakrishnan, 2009).

Regulation of solute transport through AQPs is a very complex phenomenon that involves environmental stimuli, transcriptional changes, and post-translational modifications. For a better understanding of AQP-mediated transport systems, integration of information generated through different approaches such as genomics, transcriptomics, and proteomics is required. In addition, information about analytical tools and available resources is also important to properly characterize AQPs. In the present review, we discuss how different approaches and analytical tools exploiting the many available resources can contribute to the study of AQPs.

## Genome-wide identification of AQPs

The initial genome-wide studies in Arabidopsis have paved the way to understand the distribution, characterization, and evolution of gene families in plants. The Arabidopsis genome has 35 AQPs that can be classified into four subfamilies based on phylogenetic distribution (Quigley et al., 2001). This classification was found to cluster fairly well with the functionality of AQPs. Subsequently, a second genome-wide study was performed in rice, which is considered as a model cereal crop and also represents a distinct monocot clade (Sakurai et al., 2005). Apart from the phylogenetic classification, solute-based classification of AQPs like aquaporins, aquaglyceroporins and S-aquaporins has also been used, particularly in animals (Benga, 2012). The information of AQPs in rice and Arabidopsis facilitated the monocot-dicot comparison that expanded the understanding of AQP gene families in plants. Later on, the genome sequencing of cucumber (*Cucumis sativus*) using next generation sequencing approaches started a new era characterized by a constant flow of reports of plant genome sequences and subsequent genome-wide AQP studies in plants (Huang et al., 2009; Deshmukh et al., 2013; Ariani and Gepts, 2015).

The genome sequences for moss (*Physcomitrella patens*) enabled the identification of 23 AQPs (Danielson and Johanson, 2008). In addition, the study added two new AQP subfamilies: Hybrid Intrinsic Proteins (HIP) and GlpF-like intrinsic proteins (GIPs) (Danielson and Johanson, 2008). Mosses, being primitive plants, are valuable for evolutionary studies, and the features observed in mosses are likely to be present in higher plants. In this regard, the seven AQP subfamilies found in mosses suggest that the diversion of AQPs was an early event and that higher plants lost two sub-families in the course of evolution. Subsequently, tissue-specific expression observed in vascular plants is argued to have evolved after the diversion of subfamilies. A recent study highlighting genome-wide comparison of AQPs in 25 plant species revealed several unique features about the subfamilies (Deshmukh et al., 2015). For instance, it is now clear that the XIP subfamily has been lost throughout the entire monocots, as well as within the *Brassicaceae*. In addition, *Brassicaceae* have also lost NIP2s from their genome (Deshmukh et al., 2015).

Most of the genome-wide studies have used AQP sequences reported in rice and Arabidopsis as a query to perform homology-based searches. However, it would be more accurate if a larger number of AQPs from different species could be included in the query sequences given that some subfamilies and groups are absent from Arabidopsis and rice. For example, NIP2s having characteristic G-S-G-R ar/R SF are missing from Arabidopsis, and, similarly, the entire XIP subfamily is absent in both Arabidopsis and rice (Table 1). In this paper, we have described over 1000 aquaporins from 26 plant species representing a wide range of families and clades (Supplementary Dataset 1). This exhaustive list will be useful as a query in genome-wide identification of AQPs in other plant species. The analytical steps required for genome-wide identification of AQPs are described in Figure S1.

**Table 1:** **Genome-wide identification and classification of aquaporins in 31 plant species**.

**Plant species**	**PIP**	**TIP**	**NIP**	**SIP**	**XIP**	**AQP**	**References**
*Physcomitrella patens*	9	4	5	2	2	23	Danielson and Johanson, 2008
*Selaginella moellendorffii*	3	3	8	1	3	19	Anderberg et al., 2014; Deshmukh et al., 2015
*Picea abies*	18	6	13	2	0	39	Deshmukh et al., 2015
*Musa acuminate*	21	18	9	3	0	51	Deshmukh et al., 2015
*Oryza sativa*	11	10	11	2	0	34	Sakurai et al., 2005
*Brachypodium distachyon*	11	10	9	2	0	32	Deshmukh et al., 2015
*Sorghum bicolor*	14	13	10	3	0	40	Deshmukh et al., 2015
*Zea mays*	10	13	13	7	0	43	Deshmukh et al., 2015
*Setaria italic*	16	16	15	3	0	50	Deshmukh et al., 2015
*Elaeis guineensis*	9	10	9	2	0	30	Deshmukh et al., 2015
*Arabidopsis thaliana*	13	10	9	3	0	35	Quigley et al., 2001
*Arabidopsis lyrata*	14	12	10	3	0	39	Deshmukh et al., 2015
*Brassica rapa*	22	16	15	6	0	59	Deshmukh et al., 2015
*Brassica oleracea*	25	19	17	6	0	67	Deshmukh et al., 2015
*Carica papaya*	10	7	7	2	2	28	Deshmukh et al., 2015
*Citrus sinensis*	11	9	8	3	3	34	Deshmukh et al., 2015
*Citrus clementine*	14	10	9	3	1	37	Deshmukh et al., 2015
*Vitis vinifera*	9	9	9	1	2	30	Deshmukh et al., 2015
*Glycine max*	22	23	17	8	2	72	Deshmukh et al., 2013
*Cajanus cajan*	12	13	10	4	1	40	Deshmukh et al., 2015
*Fragaria vesca*	10	9	14	4	2	39	Deshmukh et al., 2015
*Prunus persica*	7	8	9	3	2	29	Deshmukh et al., 2015
*Ricinus communis*	10	9	8	4	5	36	Deshmukh et al., 2015
*Populus trichocarpa*	15	18	11	7	7	58	Gupta and Sankararamakrishnan, 2009
*Solanum tuberosum*	15	11	11	2	5	44	Deshmukh et al., 2015
*Solanum lycopersicum*	14	10	11	3	6	44	Deshmukh et al., 2015
*Phaseolus vulgaris*	12	13	10	4	2	41	Ariani and Gepts, 2015
*Hevea brasiliensis Muell. Arg*.	15	17	9	4	6	51	Zou et al., 2015
*Hordeum vulgare L*	11	7	4	2	0	22	Hove et al., 2015
*Jatropha curcas*	9	9	8	4	2	32	Zou et al., 2016
*Phyllostachys edulis*	10	6	8	2	0	26	Sun et al., 2016
Total 31 plant species	402	348	316	105	53	1224	

## Transcriptomics studies for AQPs

Transcriptomics progressed initially with the technological improvement in chip-based expression profiling platforms (Schulze and Downward, 2001). Subsequently, the advancements in affordable sequencing technologies have greatly contributed to transcriptome sequencing (Burgess, 2016; Chen et al., 2016). As a result, transcriptomic resources have become widely available with RNA-seq studies performed on many plant species covering major crops, medicinal plants, model species and plant species important for evolutionary studies (www.ncbi.nlm.nih.gov/sra). Available transcriptomic resources are helpful to integrate information with genomics data for a better comprehension of gene functions (Movahedi et al., 2012; Patil et al., 2015; Sonah et al., 2016; Song et al., 2016). For instance, many of the recent studies highlighting genome-wide identification of AQPs have relied on transcriptomic resources to explain tissue-specific expression of those genes (Gupta and Sankararamakrishnan, 2009; Reuscher et al., 2013; Venkatesh et al., 2013; Deshmukh et al., 2015; Hu et al., 2015; Deokar and Tar'an, 2016; Deshmukh and Bélanger, 2016; Zou et al., 2016).

In a study of genome-wide identification of AQPs in soybean, we have used publically available RNA-seq and microarray data to elucidate the expression profile of AQPs across tissues (Deshmukh et al., 2013). Among particular observations, the study revealed a seed-specific expression for all members of the TIP3 subgroup. A similar type of seed-specific expression for TIP3s has been reported with rice and Arabidopsis, transcriptomic data (Deshmukh et al., 2013). These results suggest an important role of TIP3s in seed development, possibly in the desiccation process required for seed maturation. Similarly, Gupta and Sankararamakrishnan (2009) have used microarray data to perform genome-wide expression profiling of AQPs in poplar, and revealed higher expression of TIPs and PIPs in xylem tissues. Using a publicly available RNA-seq data for barley, Hove et al. (2015) observed a high level of HvNIP4;1 expression in inflorescences. Recently, the tissue-specific expression of NIP4s (AtNIP4; 1 and AtNIP4; 2) was found to be required for pollen development and pollination in *Arabidopsis thaliana* (Di Giorgio et al., 2016). Such information about expression profile is instrumental in defining substrate specificity and interdependency among AQPs.

**Figure 1:**
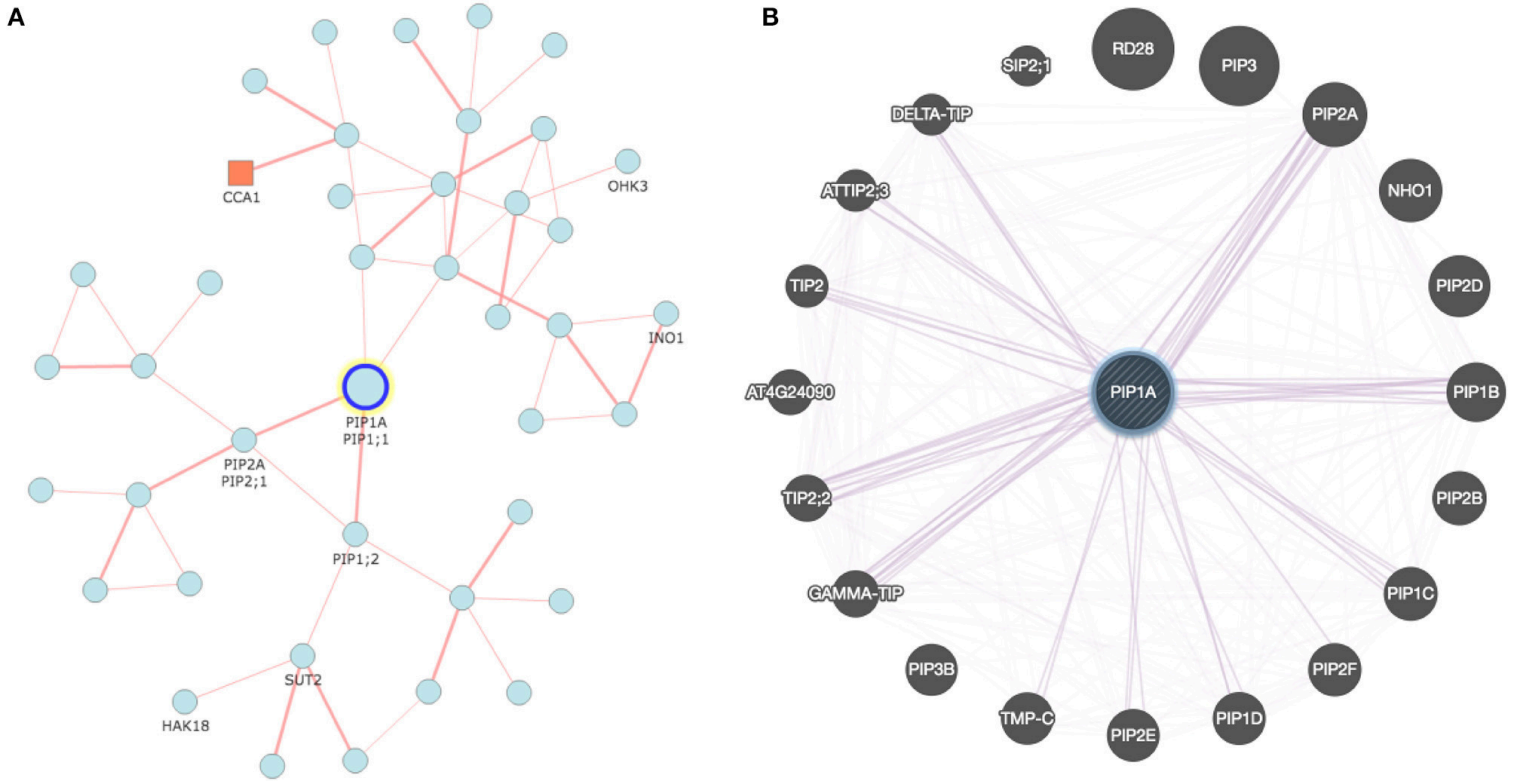
**Co-expression network developed for rice and Arabidopsis aquaporin genes: (A)** Network analyzed with RiceFREND tool (http://ricefrend.dna.affrc.go.jp) showing interdependency of PIP1-1, PIP1-2, and PIP2-1 in rice; and **(B)** Network of Arabidopsis visualized with GENEMANIA tool (http://genemania.org) showing interaction of PIP1A with PIP2 and other genes. Network for all rice AQPs at the third hierarchical level is provided in Supplementary Data 2.

Interdependency of AQPs is a well-known phenomenon, particularly in the case of PIP1s and PIP2s (Yaneff et al., 2014). The AQP-mediated transport system is very complex and will rely on the conjugated action of distinct transporters to carry solutes from one tissue to another (Ma et al., 2007; Sakurai et al., 2015). In this context, recent developments in analytical tools offer great opportunities for construction of co-expression networks and several online tools are available for this purpose (Table 2). As an example, Figure 1 describes a co-expression network in rice using the online tool FREND that revealed the concerted role of PIP2-1, PIP1-1, and PIP1-2. Another tool, PlaNet, allows a comparative analysis of co-expression networks across plant species such as rice, soybean, *Brachypodium*, barley, *Medicago*, poplar and wheat. Furthermore, some tools exploit data from the literature, known protein domains, experimentally proven protein-protein interactions, genetic interactions based on QTL/GWAS studies, and information generated through proteomics interactions to infer specific roles of AQPs (Table 2). As part of an integrated omics approach, these tools will provide precise information about AQP-mediated molecular events in plants.

**Table 2:** **Tools available for the development of co-expression network using transcriptomic data from different plant species**.

**S. no**.	**Database/Online tool**	**Plant species**	**Website**	**Other notes/Features**
1	PlaNet	Arabidopsis, barley, *Medicago truncatula*, poplar, rice, soybean, *Nicotiana tabacum*, and wheat	http://aranet.mpimp-golm.mpg.de	Allows comparative analysis of co-expression networks across plant species
2	PLANEX	*Arabidopsis thaliana*, soybean, barley, rice, tomato, wheat, grape and maize	http://planex.plantbioinformatics.org	Gene Expression Omnibus (GEO)
3	ATTED-II	Arabidopsis, soybean, maize, rice, tomato, wheat, grape poplar, and muster	http://atted.jp	Uses known protein-protein interactions and functional annotations
4	CressExpress	Arabidopsis	http://cressexpress.org	Suitable for downstream data-mining, visualization, and analysis.
5	Genemania	Arabidopsis	http://genemania.org	GeneMANIA's database of 1800+ networks, containing over 500 million interactions across eight organisms
6	CORNET	Maize, Tool	https://bioinformatics.psb.ugent.be/cornet/	Allows co-expression analysis using either predefined or user-defined groups of micro array experiments.
7	VTCdb	Grape	http://vtcdb.adelaide.edu.au/Home.aspx	Retrieves hierarchical optimized Gene Ontology enrichment and tissue/condition specificity genes within the module along with interactive network visualization and analysis via CytoscapeWeb.
8	CORE	Rice	https://core.ac.uk/display/8598313/tab/similar-list	Creates gene co-expression networks using both condition-dependent and condition-independent data
9	Xpressomics	Arabidopsis	https://xpressomics.com/search/	Uses differential expression from expert-curated and analyzed raw data
10	CoP	Arabidopsis, soybean, barley, rice, poplar, wheat, grape, maize	http://webs2.kazusa.or.jp/kagiana/cop0911/	Provides information about gene co-expression, specific gene expression, biological processes, and metabolic pathways that are mutually interconnected
11	RiceFREND	Rice	http://ricefrend.dna.affrc.go.jp/	Based on a large collection of microarray data

## Proteomics contributions in AQP studies

Compared to genomics and transcriptomics, proteomic approaches have contributed limited efforts to the study of AQPs. This is mostly because of the costly and demanding methodological requirements of proteomic studies. Most of the large-scale proteomic analyses focusing on AQPs have been conducted with Arabidopsis, and rice (Table S1). In general, modern proteomic tools yield an enormous amount of valuable data that can be used to resolve complex molecular mechanisms, more specifically regarding post-translation modifications, protein expression, and protein-protein interactions (Deshmukh et al., 2014). However, relatively limited information is available for membrane proteins including AQPs because of constraints such as limited solubility, low expression and restricted use of restriction enzymes (Tan et al., 2008).

Solubility of AQPs is a very critical issue to perform efficient proteomic studies because of their higher hydrophobicity, difficult extraction, and presence in relatively small amounts. This issue prevents or limits reliance on standard approaches utilizing mass spectrometry (MS), matrix-assisted laser desorption ionization (MALDI), and electrospray ionization (ESI). Some of the pioneer studies, including those of Schindler et al. (1993) and Schey et al. (1992), developed procedures for improving solubilisation of membrane proteins by using organic solvents, acetonitrile, 2-propanol, hexafluoro-2-propanol and detergents. Better solubilisation also helps for a more efficient digestion. However, problems with dissolving membrane proteins remain even though the amount of AQPs obtained with fractionated plasma membrane was somewhat alleviated with the advancement in instrumentation that works at the nano-scale level (Table S1).

Santoni et al. (2003) described the first comprehensive efforts for AQP research using proteomic approaches. They developed an inventory of Arabidopsis AQP isoforms expressed in root tissues that were characterized with MALDI and electro-ionization tandem MS. The study also provided key information about phosphorylation and other post-translational modifications of AQPs, particularly within the PIP subfamily. Nearly a decade after this pioneer work, a study by Mirzaei et al. (2012) demonstrated the effects of environmental factors like drought on AQP regulation in rice. The study described the AQP expression profile at precise physiological stages during the progression of drought over time. For this purpose, the authors used label-free quantitative shotgun proteomic approaches involving nano-LC-MS/MS to identify 1548 proteins including AQPs, and predicted the mechanisms involved in drought stress. Similarly, di Pietro et al. (2013) conducted extensive proteomic studies of Arabidopsis AQPs under different physiological conditions. They studied nine physiological treatments modulating root hydraulics over different time periods. They observed 55 AQP peptides undergoing significant changes with respect to different physiological conditions, including several post-translational modifications like methylation, acetylation, and phosphorylation.

## Aquaporin tertiary structure

The precise definition of the molecular structure of a protein is very important to understand its function. It contributes to the elucidation of the specific activity of a protein and its interaction with other molecules including ligands and inhibitors. Since the discovery of the first AQP in human red blood cells and renal proximal tubules, several attempts have been made to resolve AQP structure (Preston et al., 1992). Initial work by Preston et al. (1994), using a-chymotrypsin digestion of intact oocytes and inside-out membrane vesicles, confirmed the cytoplasmic loops and orientations of the six transmembrane alpha helices predicted by the hydropathy analysis. In the same year, another study by Jung et al. (1994) predicted the hourglass model for AQPs based on the topological information and the positioning of the loops, more particularly loop B and loop E, which penetrate in the membrane from opposite sides to form a constrict harboring conserved NPA domains. This study was instrumental in predicting for the first time the role of NPA domains in the tight regulation of solute transport. Later, Murata et al. (2000) solved the atomic structure of AQP1 using electron crystallographic analysis that confirmed the earlier predictions. The AQP1 structure has 3.8 Å resolution describing highly conserved amino-acid residues that stabilize the fold, and form the hourglass structure. The structural model presented by Murata et al. (2000) showed the conserved hydrophobic residues lining the water channel at the center of the protein. The structure also showed constricts with a pore diameter of about 3 Å that provided a clue as to how the AQP was permeable to water but not the proton.

Up to now, about 51 AQP structures have been described using different approaches at varying levels of resolution (Table S2). Most of the solved structures belong to human and *Escherichia coli* AQPs representing eukaryotic and prokaryotic models. Apart from human, high-resolution AQP structures are also available from other animals such as rat, sheep, and cattle (Table S2). Compared to animals and prokaryotes, very few structures are available for plant AQPs. The first plant AQP structure, common bean TIP, was solved by Daniels et al. (1999) at low resolution (7.7 Å) using electron cryo-crystallography. In spite of the low resolution, the AQP structure showed resemblance with animal AQPs. Törnroth-Horsefield et al. (2006) described the first high resolution plant AQP structure (from spinach, SoPIP2;1) in its closed (2.1 Å resolution) and open conformation (3.9 Å resolution). The structure with closed and open conformation explained the gating mechanism in which loop D, through a displacement of up to 16 Å, widened the pore and acted as molecular gating. The mechanism of gating is found to be conserved across all plant species (Törnroth-Horsefield et al., 2006). Recently, the structure of Arabidopsis aquaporin AtTIP2;1 was determined at very high resolution (1.18 Å) (Kirscht et al., 2016). Most of the previously reported structures are from water-transporting AQPs (Table S2) but AtTIP2;1 is a model AQP for ammonia transport. Interestingly, Kirscht et al. (2016) discovered a fifth amino acid involved in the permeability of ammonia, expanding the complexity of solute specificity from the four amino acids ar/R SF. Owing to the increasing availability of high-resolution AQP structures, homology-based computational approaches used to predict 3D-structure have now become more efficient and sophisticated (Tables S2, S3).

Advances in computational methods during the last two decades have made it possible to predict structures more accurately. The success of computational methods can be attributed to the evolutionary conserved features of proteins, the relatively small number of unique protein fold in nature, and the ever increasing number of solved protein structures (www.rcsb.org; Koonin et al., 2002). Compared to proteins that have no similarity with known structures, prediction of structure for candidate AQPs is facilitated by the abundance of resolved AQP structures publicly available (Table S2). In addition, several online servers and tools available for the homology-based prediction of protein structures are helpful to pursue more advanced studies in AQPs (Table S3).

Molecular dynamics simulations are advanced computational approaches used for *in silico* reconstitution of protein structure in its native environment (Lindahl and Sansom, 2008). Molecular dynamics simulations became more advanced with the availability of very high-resolution 3-D structures, increased computing power, and improvement in the analytical algorithms. In addition, detailed information about the interaction between amino acid residues and the surrounding environment makes it possible to reconstitute protein structures in different environments (Lindahl and Sansom, 2008). More particularly, studies focusing on membrane proteins have provided information about the interaction between individual amino acids with the lipid molecules in the membrane environment. There are several methods and tools available for molecular dynamics simulations of membrane proteins like AQPs (Lindahl and Sansom, 2008). Recently, Sakurai et al. (2015) have performed molecular dynamics simulations to study silicic acid uptake through AQP (OsLsi1) coupled with another active transporter (OsLsi2) in rice roots. They developed a mathematical model using diffusion equation along with the effects of active transport by OsLsi2. The study provides s good example for the utilization of *in vivo* experimental data to calibrate the model.

## Analytical and functional approaches

### *Xenopus* oocyte assay

**Figure 2:**
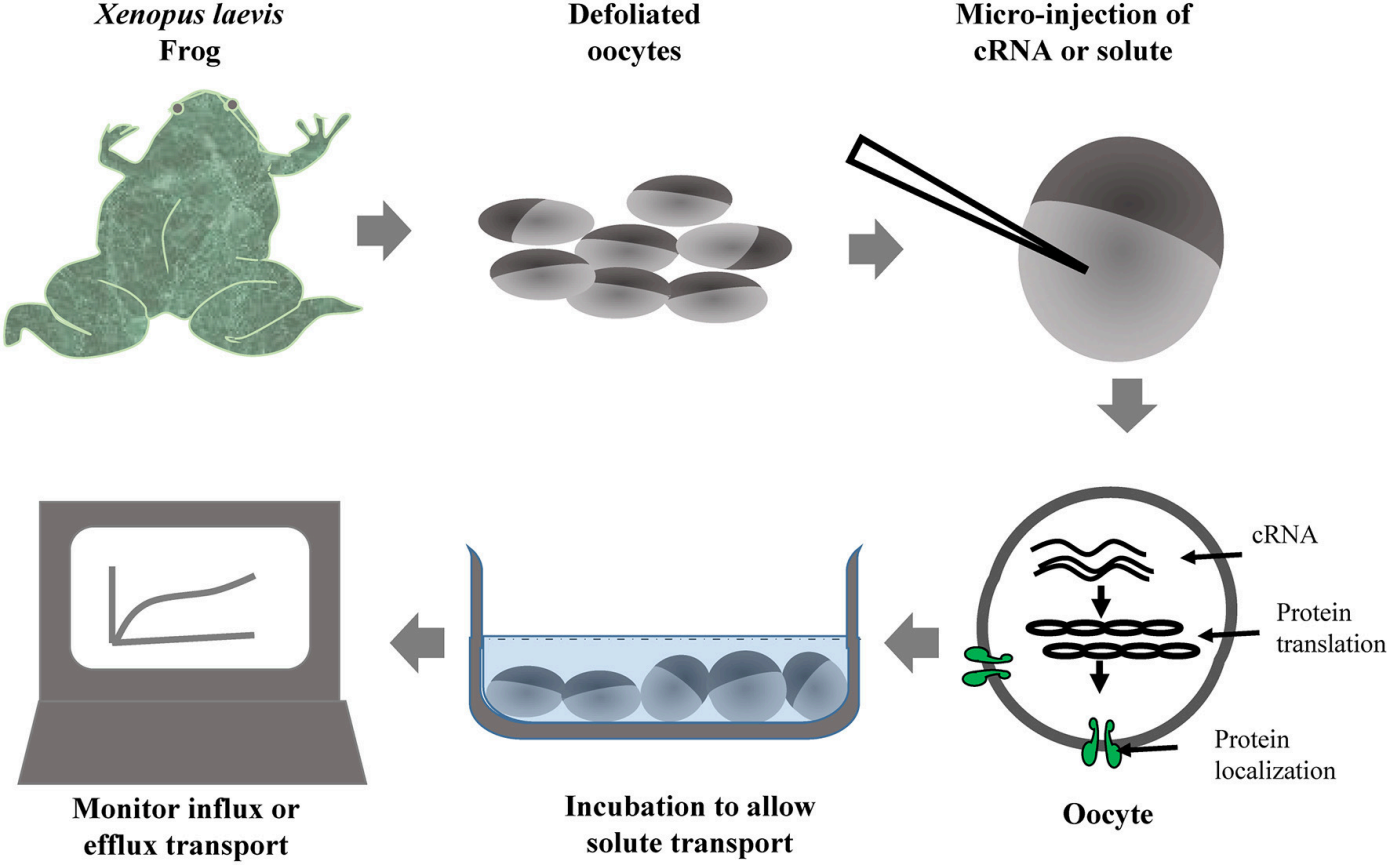
**Simplified workflow of ***Xenopus*** oocyte assay used for the evaluation of solute transport by heterologously expressed foreign transporters including aquaporins**.

Oocytes of *Xenopus laevis* (African clawed frog) are commonly used for the evaluation of solute transport activity by AQPs and many other transporters. cRNA of foreign proteins can be easily injected and expressed in *X. laevis* oocytes. A cRNA volume of up to 50 nL can be injected in the oocyte, which allows production of large amounts of protein. The simplified steps involved in the *X. laevis* oocyte assay are provided in Figure 2. The transport of several different substrates by plant AQPs has been evaluated using *X. laevis* oocyte assay (Table 3). The oocyte assay conveniently allows the use of radiolabeled substrates to facilitate a better estimation of transport kinetics and also to increase the sensitivity of the assay (Ma et al., 2006). However, in the case of certain substrates such as silicic acid, many have used ^68^Ge as silicic acid surrogate, a method often criticized given that ^68^Ge was never shown to represent a perfectly interchangeable surrogate. In recent studies, silicic acid transport (influx and efflux) in oocytes was measured directly by atomic absorption spectrophotometry, a technical improvement that greatly facilitates the study of silicic acid movement in plants (Ma et al., 2006; Grégoire et al., 2012; Deshmukh et al., 2015; Carpentier et al., 2016; Vivancos et al., 2016). The measurement of change in volume of oocyte in response to osmolality of external solution is a simple and effective measure to study water transport by AQPs. The *X. laevis* oocyte system provides several advantages for the study of transporters. For instance, there is very low transport across the oocyte membrane through the endogenous transporters; therefore there is limited background effect and less ambiguity about solute transport. In addition, the relatively large size of *X. laevis* oocytes facilitates their manipulation and allows studying electrophoretic transporters using the two-electrode voltage clamp technique. Nevertheless, one has to keep in mind that the environment of a plant cell is drastically different from that of the *X. laevis* oocyte. Therefore, results obtained with the *X. laevis* oocyte assay need to be corroborated with the actual activity of the protein in plant cells.

**Table 3:** **List of important studies performed to evaluate different solutes transported by plant aquaporins using the ***Xenopus*** oocyte assay**.

**Plant species**	**Gene**	**Solute**	**References**
Arabidopsis	AtTIP2;1, AtTIP2;3	Ammonium	Loqué et al., 2005; Liu et al., 2013
Rice	Lsi1 (OsNIP2;1)	Arsenite	Zhao et al., 2010
Arabidopsis	NIP5;1	Boron	Takano et al., 2006
Arabidopsis	NIP6;1	Boron	Tanaka et al., 2008
*Nicotiana tabacum*	NtAQP1	CO2	Uehlein et al., 2003
*Nicotiana tabacum*	NtXIP1;1	Glycerol, urea, boric acid	Bienert et al., 2011
Soybean	Nodulin 26	Glycerol, Water	Dean et al., 1999
*Nicotiana tabacum*	NtAQP1	Glycerol, Water,	Biela et al., 1999
Poplar	PtNIP2-1	Silicic acid	Deshmukh et al., 2015
Tomato	SlNIP2-1 (Mutant)	Silicic acid	Deshmukh et al., 2015
Barley	HvLsi1	Silicic acid	Chiba et al., 2009
*Equisetum arvense*	EaNIP3;1, EaNIP3;3 and EaNIP3;4	Silicic acid	Grégoire et al., 2012
Soybean	GmNIP2-1, GmNIP2-2	Silicic acid	Deshmukh et al., 2013
Rice	Lsi1 (OsNIP2;1)	Silicic acid (^68^Ge)	Ma et al., 2006
Maize	ZmPIP1-5b	Urea, Water	Bousser et al., 2003
Spinach	PM28A	Water	Johansson et al., 1998
Maize	ZmPIP2a	Water	Chaumont et al., 2000
Rice	OsPIP1;1	Water	Liu et al., 2013
Radish	VM23	Water	Higuchi et al., 1998
Olive	OePIP2.1, OeTIP1.1	Water	Secchi et al., 2007
Tomato	LeAqp2	Water	Werner et al., 2001
Walnut	JrPIP2,1	Water	Sakr et al., 2003
Soybean	GmTIP1-5, GmTIP2-5	Water	Song et al., 2016

Translation of plant AQP transcripts in the oocyte may be altered by the differential codon preference between plants and *Xenopus*. For this reason, western blotting is often required to confirm protein expression/presence in the oocytes. However, codon optimization is rarely considered for plant AQPs when tested with oocytes, which raises the question, whether codon optimization is really a concern or not. Recently, Feng et al. (2013) reported better nitrate transport in oocytes with codon-optimized rice high affinity nitrate transporter. Similarly Bienert et al. (2014) also observed a significantly higher expression of ZmPIP1 and ZmPIP2 in yeast cells only after optimizing the codons. Nowadays, the use of synthesized DNA for gene-cloning related applications is becoming more common because of reduced costs and codon optimization can be routinely applied while synthesizing the gene for oocyte or yeast assays.

### Evaluation of AQPs using yeast assays

Characteristic features of yeasts including ease of growth, short generation time, well established and easy transformation systems, and sequenced genomes, make them amenable as a heterologous system to study eukaryotic proteins. Several yeast species, including *Saccharomyces cerevisiae, Schizosaccharomyces pombe*, and *Pichia pastoris* have been used as a tool to study foreign genes.

**Figure 3:**
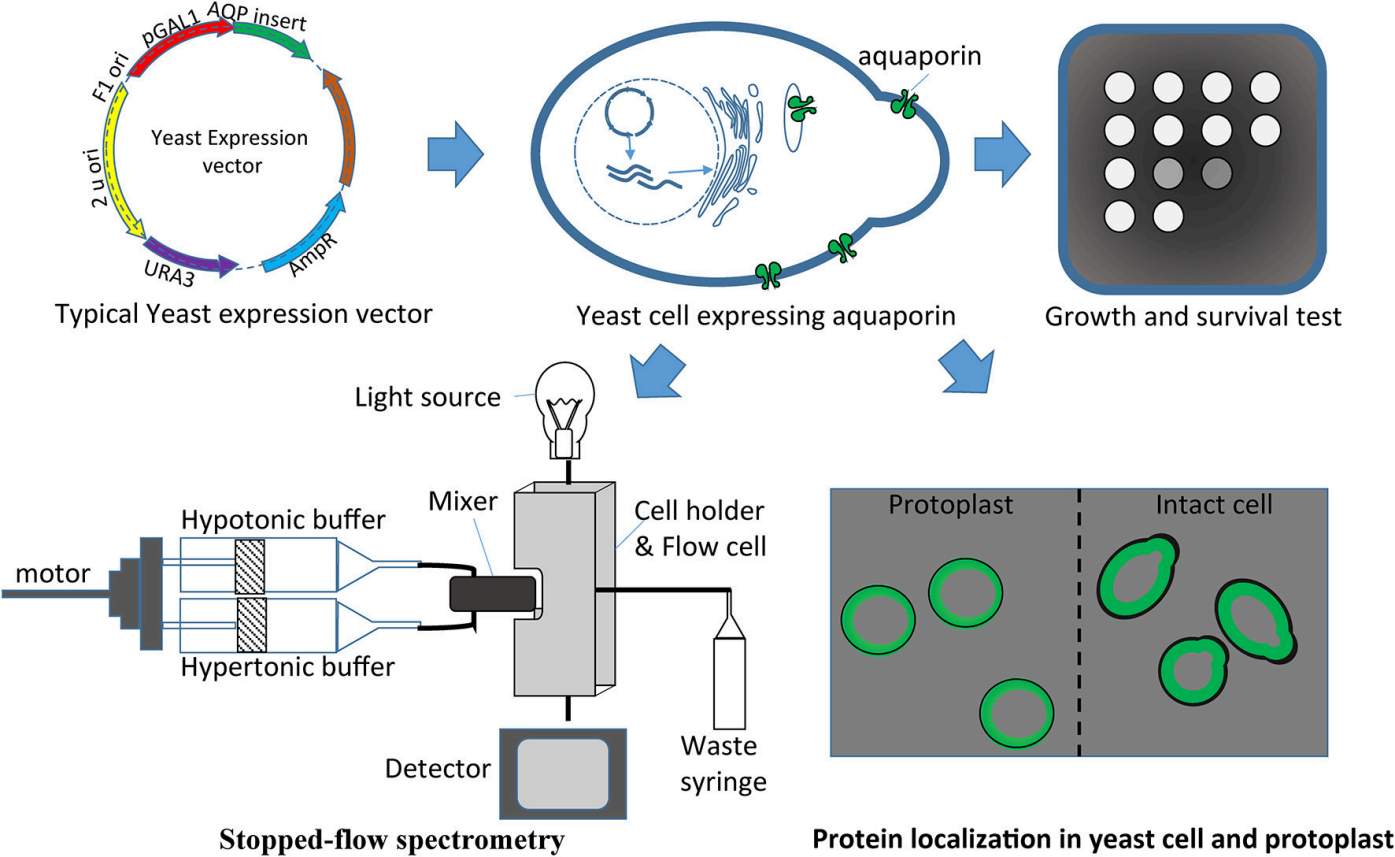
**Yeast heterologous expression system used to evaluate aquaporins**. Aquaporin cloned in the expression vector can be easily transferred in yeasts for different assays like growth and survival tests, protein localization, and/or stopped-flow spectrometry.

Numerous AQPs have been studied using a yeast expression system (Table 4). The study of water fluxes through AQPs in yeast assays is a particularly easy and affordable option. Water transport in yeast results in measurable cell volume changes (swelling or shrinking) in relatively short periods of time. The water transport through AQPs (as a hydrophilic passage) is much faster than the transport through the hydrophobic lipid bilayer membrane. This allows discrimination between water transport through the foreign AQP expressed in the yeast and the transport through the membrane. The volume change in yeast affects several physical parameters that can be used to make quantitative measurements essential to understand transport kinetics. Light absorption, light scattering or reflection with fluorescent dye are effective variables that are being used to monitor cell volume changes (Table 4, Figure 3). Given the small the size of the yeast that takes milliseconds to change volume in response to osmotic pressure, it requires a method known as stopped-flow spectrometry to take precise measurements. In stopped-flow spectrometry, protoplasts, vesicles or even intact cells are subjected simultaneously to hypotonic and hypertonic buffers. The rapidly mixing hypotonic and hypertonic solution stops transport in milliseconds, which allows taking measurements at the scale required to understand transport kinetics. Use of florescent dye in stopped-flow spectrometry increases precision of measurements. Recently, Sabir et al. (2014) evaluated grape AQPs for water conductivity using a stopped-flow fluorescence spectroscopy assay. They pre-loaded yeast cells with the non-fluorescent precursor 5-(and-6)-carboxyfluorescein diacetate (CFDA) that is permeable to membranes, and then intracellularly hydrolyzed CFDA to release the membrane impermeable fluorescent compound. Changes in cell volume in stopped-flow assay in response to osmotic changes resulted in changes in fluorescence intensity that can be measured to deduct transport kinetics.

**Table 4:** **List of plant aquaporins evaluated for transport of different solutes using yeast assays**.

**Aquaporin**	**Plant**	**Yeast strain**	**Solute**	**Yeast assay**	**References**
TIP1;1, TIP1;2	Arabidopsis	Δ*tsa1,2*, Δ*skn7*, Δ*yap1*, Δ*fps1, Δyfl054c*	H_2_O_2_	*Spheroplast Swelling Assay*, Survival test, *Catalase Activity Assay, Fluorescence Assay, Bioimaging*	Bienert et al., 2007
AtNIP1;1, AtNIP2;1, AtNIP5;1,AtNIP6;1, AtNIP7;1	Arabidopsis	W303-1A	As(III), As(V), antimonite	Yeast growth and survival test	Bienert et al., 2008
AtPIP1;2, AtPIP2;3	Arabidopsis	W303	CO_2_	Stopped-flow spectrometry	Heckwolf et al., 2011
SIP1;1, SIP1;2, SIP2;1	Arabidopsis	BJ5458	H_2_O	Stopped-flow spectrometry	Ishikawa et al., 2005
AtNIP7;1	Arabidopsis	acr3Δ, fsp1Δ	As(III), As(V),	Yeast growth and survival test	Isayenkov and Maathuis, 2008
HvNIP2;1	Barley	INVSc2, SY1	Boric acid, Ge, As	Yeast growth and survival test	Schnurbusch et al., 2010
HvNIP1;1, HvNIP1;2, HvNIP2;1, HvNIP2;2	Barley	ΔSKN7, ΔACR3	H_2_O_2_, As(OH)_3_	Yeast growth and survival test	Katsuhara et al., 2014
VvTnPIP2;1, VvTnTIP1;1, VvTnTIP2;2, VvTnPIP1;4, VvTnPIP2;3, VvTnTIP4;1	Grape	10560-6B	H_2_O	Stopped-flow fluorescence spectroscopy	Sabir et al., 2014
HaTIP1;1, HaPIP1;1, HaPIP1;2	*Helianthemum almeriense*	SY1	CO_2_, NH_3_, H_2_O	Stopped-flow spectrometry	Navarro-Ródenas et al., 2013
VALT (TIP), PALT1 (PIP)	Hydrangea (*Hydrangea macrophylla*)	Δhsp150, BY4741	Al	Yeast growth and survival test	Negishi et al., 2012
LjNIP5;1, LjNIP6;1	*Lotus japonicus*	W303-1A	As(III), As(V), antimonite	Yeast growth and survival test	Bienert et al., 2008
LjNIP1	*Lotus japonicus*	31019b, YNVW1	Amonia, Urea, H_2_O	Stopped-flow spectrometry, Yeast growth and survival test	Giovannetti et al., 2012
ZmPIP1;2, ZmPIP2;5	Maize	31019b (*Δmep1–3*), BY4741	H_2_O_2_	Yeast growth and survival test, Codon optimization	Bienert et al., 2014
PvTIP4;1	Pteris vittata	*Δfps1* and *Δacr3*	As(III), As(V)	Yeast growth and survival test	He et al., 2016
RsPIP1-2, RsPIP1-3, RsPIP2-1, RsPIP2-2	Radish	BJ5458	H_2_0	Stopped-flow spectrophotometry	Suga and Maeshima, 2004
OsNIP1;1, OsNIP1;2, OsNIP2;1, OsNIP2;2, OsNIP3;1, OsNIP3;2, OsNIP3;3, OsNIP4;1	Rice	ΔSKN7, ΔACR3	H_2_O_2_, As(OH)_3_	Yeast growth and survival test	Katsuhara et al., 2014
OsNIP2;1, OsNIP2;2, OsNIP3;2	Rice	W303-1A	As(III), As(V), antimonite	Yeast growth and survival test	Bienert et al., 2008
TsTIP1;2	*Thellungiella salsuginea*	*aqy-null* strain	H_2_O_2_	Fluorescence assays, Yeast growth and survival test	Wang et al., 2014
NtXIP1;1, StXIP1;1	Tobacco	YNVW1 (*Δdur3*)	Urea	Yeast growth and survival test	Bienert et al., 2011
TaTIP2;2	Wheat	31019b, BJ5458	Ammonia	Stopped-flow spectrometry	Bertl and Kaldenhoff, 2007

Apart from water, transport of many other solutes through AQPs is also studied with yeast systems. Commonly, yeast growth and survival are used to study solutes (Table 4). For instance, uptake of germanium (Ge), arsenate (As), boric acid and antimonite severely affect yeast growth, and this effect can be measured through heterologous expression of AQPs specific for such solutes. Similarly, mutant strains like YNVW1 carrying the deletion Δ*dur3* cannot grow on media with urea as sole nitrogen source, thus making such deletion strains useful to study urea transport by AQPs (Table 4).

Recently, To et al. (2015) demonstrated rapid screening of an AQP mutant library to evaluate the effects of amino acid changes on solute transport. The authors developed a novel method that looks promising to study water transport ability for hundreds of AQPs simultaneously. The assay can be used to identify inhibitors as well as co-transporting molecules. The method exploits the property of yeast cells that show increased freezing tolerance with expression of functional AQPs. The rapid transport of water through AQP allows removal of water from freezing yeast cells that avoid formation of ice crystals thus preventing cell damage. With this method, a library of yeasts (preferably AQP mutant strains) transformed with different AQPs can grow in 96-well microplates that are exposed to freeze-thaw cycles. Only the yeasts transformed with functional AQPs will survive following freeze-thaw cycles, a quick way to assess AQP properties. Such high-throughput procedures will certainly expand the analytical power required to integrate omics scale research.

### Mesophyll protoplast assay

Mesophyll protoplasts can be easily obtained for several plant species (Shen et al., 2014). More particularly, the procedure for the evaluation of AQPs using Arabidopsis, tobacco and maize mesophyll protoplasts is well established (Yoo et al., 2007; Besserer et al., 2012; Ma et al., 2015). There are several methods for the delivery of macromolecules into protoplasts including, electroporation, microinjection, and PEG–calcium fusion method (Sade et al., 2009; Shen et al., 2014). Transient expression of AQPs in plant mesophyll protoplast allows efficient study of solute transport and also the subcellular localization of the protein. Recently, Wang et al. (2015) exploited Arabidopsis protoplasts for the transient expression of an AQP from maize (MzPIP2;1) tagged with a green fluorescent protein to confirm the plasma membrane specific localization. Similarly, Chevalier et al. (2014) used the mesophyll protoplast assay to study subcellular localization of several maize AQPs belonging to the ZmPIP1s and ZmPIP2s subfamilies. They observed efficient localization in plasma membrane only for ZmPIP2s when expressed alone in the mesophyll protoplasts. They further swapped transmembrane domain-3 along with the ER export diacidic motif to demonstrate its requirement in the localization. Another study conducted using tobacco protoplasts showed the role of phosphoinositides in modulating aquaporin activity (Ma et al., 2015). AQP gene ZmPIP2;4 from maize was transiently expressed in tobacco protoplasts to evaluate phosphoinositides effect on AQP expression and water flux (Ma et al., 2015). In spite of the rather routine application of the mesophyll protoplast assay, a high-throughput method for AQP evaluation using this assay has not been developed yet. Such an assay would allow large-scale exploitation of native rather than surrogate membranes. However, the major limitation with this technique is the requirement of highly skilled expertise to handle protoplasts, and the relatively low rate of success when experiments are replicated across different labs.

### Isolated vesicle assay: right-side out and inside-out

Vesicles isolated from different types of tissues and cell types are being used for the evaluation of AQPs. The vesicles can be easily obtained following ultracentrifugation-based fractioning. After obtaining vesicles separated from the other organelles and cytoplasm, AQPs can be studied with stopped-flow fluorescence spectroscopy that measures shrinking/swelling of the vesicle. Dordas et al. (2000) used plasma membrane vesicles obtained from squash (*Cucurbita pepo*) roots to study the role of AQPs in boric acid transport. They used mercuric chloride and phloretin, a well-known non-specific transporter inhibitor, to conclude that boric acid permeation occurred both through proteinaceous channels and diffusion through the membrane.

Isolated inverted vesicles are useful to measure uptake inside the vesicle by efflux transporter. The reversed membrane vesicle is known as inside-out where the apoplastic side is inside the vesicle. The inside out vesicles are generally used for the evaluation of efflux transporters and energy dependent transporters. Initially, Palmgren et al. (1990) developed the method to prepare inside-out and right-side-out (apoplastic side out) vesicles using sugar beet (*Beta vulgaris* L.) leaves. They used freezing and thawing to turn vesicles inside-out and subsequently separated the inside-out and right-side-out by repeating the phase partition step. ATPase assay is used to verify the proportion of inside-out vesicles, since the ATPase active site is situated on the cytoplasmic side of the membrane, and only sealed, inside-out vesicles efficiently perform ATP-dependent H+ pumping (Palmgren et al., 1990). Similarly, Sutka et al. (2005) used the stopped-flow technique to measure water transport activity of tonoplast vesicles. They were able to conclude that most of the AQPs located in the tonoplast membrane are sensitive to HgCl_2_ and few of them are inhibited by pH. Their study expanded the use of isolated vesicle assays to the tonoplast-specific AQPs. However, the major drawback of the system resides in its labor intensive procedure required to isolate plasma membrane vesicle fractions.

## Transgenic approaches used for aquaporin research

The heterologous expression of AQPs in plants represents a useful approach for functional evaluation of AQPs, even though it can sometimes lead to experimental artifacts. Indeed, transgenic approaches are often criticized over the use of constitutive or non-specific promoters that express AQPs in tissues where natural expression is not observed. In the case where a plant trait is governed by genotypic variations, complementation assays with a transgenic approach is considered more reliable since expression of the transgenes are evaluated under the same conditions. In addition to contributing to the functional annotation of novel AQPs, transgenic approaches are also being exploited to develop crop plants with agronomically important traits (Table S4). Currently, Arabidopsis and tobacco remain the preferred source for the heterologous expression of genes including AQPs. In addition, extensive resource of T-DNA insertion mutant libraries available for Arabidopsis makes it an obvious choice for the functional study of novel AQPs. As a matter of fact, mutants are available for most of the AQPs identified in the Arabidopsis genome, which facilitates evaluation of any native Arabidopsis AQP as well as their homologs in other plant species.

Most of the AQP transgenic studies have evaluated responses against abiotic stresses like high salinity, drought, and cold (Table S4). In a notable effort, Jang et al. (2007) analyzed the effect of expression of *AtPIP1;4* and *AtPIP2;5* in Arabidopsis and tobacco transgenic plants under various abiotic stress conditions. They also noticed a change in endogenous AQP genes with the over expression of *AtPIP1;4* and *AtPIP2;5* transgenes. Another study by Peng et al. (2007) reported that the overexpression of *PgTIP1* from *Panax ginseng* in transgenic Arabidopsis plants led to enhanced tolerance against salt-stress and drought, but lowered cold acclimation ability. The contrasting effect of overexpression of an AQP on drought and cold is expected since altered water movement has a contrasting effect over these stresses. However, many contradictory results with the heterologous expression of AQPs in transgenic plants have been reported. For instance, Peng et al. (2008) observed a significantly lowered freezing tolerance with overexpression of *Rhododendron catawbiense* AQP (RcPIP2) in Arabidopsis. Similarly, with the overexpression of a PIP in transgenic tobacco, Aharon et al. (2003) achieved improvement in plant growth under favorable growth conditions but not under drought or salt stress. AQPs are also extensively studied for physiological parameters like CO_2_ conductance and photosynthesis efficiency (Table S4). For instance, Katsuhara and Hanba (2008) evaluated the effect of *HvPIP2;1*, cloned from barley for the transport of water and CO_2_ conductance in a transgenic rice. In a rare study with biotic stress, Vivancos et al. (2015) showed that expression of a wheat *NIP2* in Arabidopsis conferred higher Si absorption and better protection against powdery mildew.

## Conclusions

Enormous progress has been achieved to understand solute transport in plants over last two decades following the discovery of the first AQP. Currently, over 30 plant species have been analyzed for genome-wide identification of AQPs. These efforts have highlighted the distribution and evolution of AQPs in plants and have also defined the phylogeny of AQPs and subsequent categorization into sub-families and groups. The dataset of over 1000 well-characterized AQPs provided here will be helpful to maximize the use of query sequences in homology-based AQP identification and subsequent characterization. Access to AQPs originating from diverse species is important to insure the identification of the entire set of AQPs in a given genome as well as their proper classification. In addition, the ever-growing resources of transcriptomic data should be exploited to characterize AQPs and refine our understanding of their role in relation with their tissue-specific expression. Integration of omics approaches to complex molecular systems like AQP-mediated transport has been facilitated lately with the development of powerful computational tools. Computational predictions however must be supported and validated by functional studies. For this purpose, biological assays such as *Xenopus* oocytes and yeast systems are now well-established approaches used to study solute specificity and transport kinetics of AQPs. In addition, several novel AQPs identified with omics efforts have been functionally annotated through transgenic approaches that have highlighted their beneficial role. With the current concerns over water resources, it is clear that a better understanding of AQP-mediated transport system in plants can only lead to the development and management of plants better adapted to changing environmental conditions.

## Author contributions

RD, HS, and RRB compiled the data, drew the conclusions and wrote the manuscript.

## Funding

The project was funded by a grant from the Natural Sciences and Engineering Research Council of Canada (NSERC), the Agri-Innovation program Growing Forward 2, SaskCanola and Agriculture and Agri-Food Canada and the Canada Research Chairs Program to RRB.

### Conflict of interest statement

The authors declare that the research was conducted in the absence of any commercial or financial relationships that could be construed as a potential conflict of interest.

## Supplementary material

Figure S1 **Proposed flowchart procedure for genome-wide identification of AQPs in plant species**.

Table S1 **Proteomic approaches applied to the study of plant aquaporins (AQPs)**.

Table S2 **Details of aquaporin tertiary structures revealed with different methods. The data were otained by search perfomed with Protein Data Bank (http://www.rcsb.org/pdb/home/home.do) on August 1st 2016**.

Table S3 **Online tools and servers available for the prediction of tertiary structures of transmembrane proteins including aquaporins**.

Table S4 **List of selected studies describing the effect of heterologous expression of aquaporins in plants**.
