# Supplementary figures and images for: Plant Aquaporins: Genome-Wide Identification, Transcriptomics, Proteomics, and Advanced Analytical Tools
Source: Front Plant Sci. 2016 Dec 20;7:1896. doi: 10.3389/fpls.2016.01896 (PMC5167727; doi:10.3389/fpls.2016.01896)

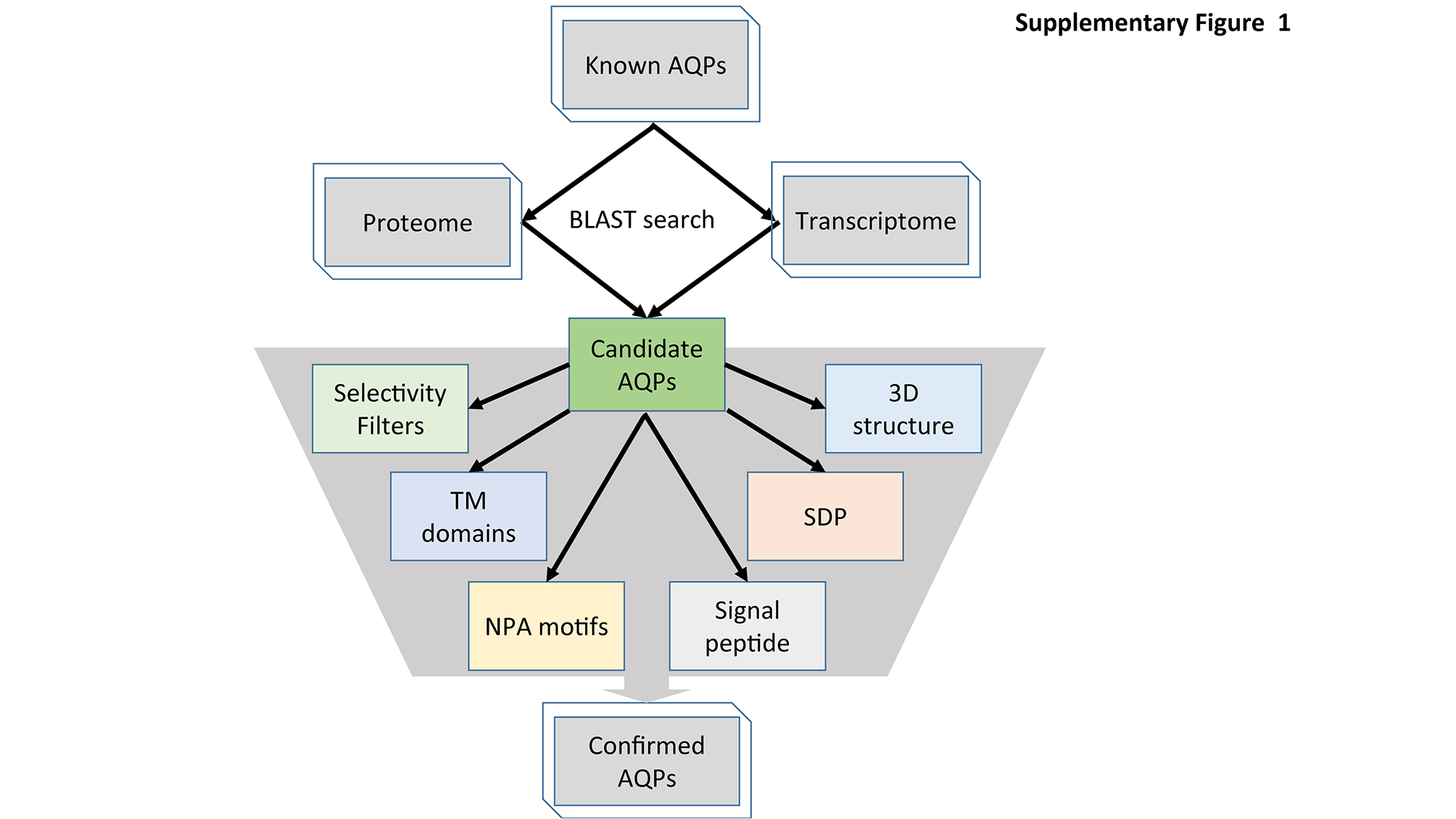

Supplement: Figure S1 — Proposed flowchart procedure for genome-wide identification of AQPs in plant species. [file Image1.TIF]
