# Supplementary material for: Plant Aquaporins: Genome-Wide Identification, Transcriptomics, Proteomics, and Advanced Analytical Tools
Source: Front Plant Sci. 2016 Dec 20;7:1896. doi: 10.3389/fpls.2016.01896 (PMC5167727; doi:10.3389/fpls.2016.01896)
Supplement: Table S1 — Proteomic approaches applied to the study of plant aquaporins (AQPs). [file Table1.DOCX]

**Table S1** Proteomic approaches applied to the study of plant aquaporins (AQPs)

|  | **Proteomic approach** | **AQPs** | **Significant outcome/other information** | **Reference** |
| --- | --- | --- | --- | --- |
| 1 | MALDI-TOF and ESI-MS | Arabidopsis PIP subfamily | Identified phosphorylated AQPs in the PIP1 and PIP2 subgroups | (Santoni et al., 2003) |
| 2 | HPLC−MS/MS | Broccoli PIPs | Identified and characterized PIPs in root tissues | (Casado-Vela et al., 2010) |
| 3 | MALDI-MS, LC-MS/MS | Arabidopsis PMPs | Developed inventory of the plasma membrane proteome including AQPs | (Marmagne et al., 2004) |
| 4 | Nano-scale LC and analyzed by tandem MS | Lotus japonicas PMPs | Identified novel proteins including AQPS in the peribacteroid membrane from *Lotus japonicus* root nodules | (Wienkoop and Saalbach, 2003) |
| 5 | Label-free quantitative proteomic methodology, LC-MS | Arabidopsis AQPs | Described post-translational modifications in AQPs triggered by environmental constraints | (Di Pietro et al., 2013) |
| 6 | LC-MS/MS, Insilicon analysis | Arabidopsis AQPs | Profiled the Arabidopsis thaliana plasma membrane transportome by targeted proteomics | (Monneuse et al., 2011) |
| 7 | LC-MS/MS; PMP extraction with inside-out vesicles | Arabidopsis PMPs and AQPs | Characterized PMPs | (Alexandersson et al., 2004) |
| 8 | Nano-LC-ESI-MS/MS, Nano-LC-MALDI MS/MS | Rice PMPs | Characterized rice PMPs for phosphorylation and revealed about 20% of the detected proteins have clearly identifiable phosphosites | (Whiteman et al., 2008) |
| 9 | LC/MS/MS, Nano-HPLC | Arabidopsis PMPs and AQPs | Characterized rice PMPs for phosphorylation. Studied level of phosphorylation in response to different factors | (Niittylä et al., 2007) |
| 10 | Nano-LC-MS/MS, LTQ-XLion-trap MS | Rice proteome including AQPs | Studied expression of AQPs during different stages of drought and interaction with other molecular mechanisms | (Mirzaei et al., 2012) |
| 11 |  |  |  |  |

MALDI-TOF: Matrix-assisted laser-desorption ionization–time-of-flight; ESI: Electrospray ionization; MS: Mass spectrometry; HPLC: high-performance liquid chromatography, LC: liquid chromatography; PMP: plasma membrane proteins; PIP: plasma membrane intrinsic proteins

Alexandersson, E., Saalbach, G., Larsson, C., and Kjellbom, P. (2004). Arabidopsis plasma membrane proteomics identifies components of transport, signal transduction and membrane trafficking. *Plant and Cell Physiology* 45**,** 1543-1556.

Casado-Vela, J., Muries, B., Carvajal, M., Iloro, I., Elortza, F., and MartíNez-Ballesta, M.C. (2010). Analysis of root plasma membrane aquaporins from Brassica oleracea: post-translational modifications, de novo sequencing and detection of isoforms by high resolution mass spectrometry. *Journal of proteome research* 9**,** 3479-3494.

Di Pietro, M., Vialaret, J., Li, G.-W., Hem, S., Prado, K., Rossignol, M., Maurel, C., and Santoni, V. (2013). Coordinated post-translational responses of aquaporins to abiotic and nutritional stimuli in Arabidopsis roots. *Molecular & Cellular Proteomics* 12**,** 3886-3897.

Marmagne, A., Rouet, M.-A., Ferro, M., Rolland, N., Alcon, C., Joyard, J., Garin, J., Barbier-Brygoo, H., and Ephritikhine, G. (2004). Identification of new intrinsic proteins in Arabidopsis plasma membrane proteome. *Molecular & Cellular Proteomics* 3**,** 675-691.

Mirzaei, M., Pascovici, D., Atwell, B.J., and Haynes, P.A. (2012). Differential regulation of aquaporins, small GTPases and V‐ATPases proteins in rice leaves subjected to drought stress and recovery. *Proteomics* 12**,** 864-877.

Monneuse, J.M., Sugano, M., Becue, T., Santoni, V., Hem, S., and Rossignol, M. (2011). Towards the profiling of the Arabidopsis thaliana plasma membrane transportome by targeted proteomics. *Proteomics* 11**,** 1789-1797.

Niittylä, T., Fuglsang, A.T., Palmgren, M.G., Frommer, W.B., and Schulze, W.X. (2007). Temporal analysis of sucrose-induced phosphorylation changes in plasma membrane proteins of Arabidopsis. *Molecular & Cellular Proteomics* 6**,** 1711-1726.

Santoni, V., Joëlle, V., Pflieger, D., Sommerer, N., and Maurel, C. (2003). A proteomic study reveals novel insights into the diversity of aquaporin forms expressed in the plasma membrane of plant roots. *Biochemical Journal* 373**,** 289-296.

Whiteman, S.A., Nühse, T.S., Ashford, D.A., Sanders, D., and Maathuis, F.J. (2008). A proteomic and phosphoproteomic analysis of Oryza sativa plasma membrane and vacuolar membrane. *The Plant Journal* 56**,** 146-156.

Wienkoop, S., and Saalbach, G. (2003). Proteome analysis. Novel proteins identified at the peribacteroid membrane from Lotus japonicus root nodules. *Plant Physiology* 131**,** 1080-1090.
