# Supplementary material for: Plant Aquaporins: Genome-Wide Identification, Transcriptomics, Proteomics, and Advanced Analytical Tools
Source: Front Plant Sci. 2016 Dec 20;7:1896. doi: 10.3389/fpls.2016.01896 (PMC5167727; doi:10.3389/fpls.2016.01896)
Supplement: Table S3 — Online tools and servers available for the prediction of tertiary structures of transmembrane proteins including aquaporins. [file Table3.DOCX]

**Table S3** Online tools and servers available for the prediction of tertiary structures of transmembrane proteins including aquaporins

| **Tools/Server** | **Specifications** | **Website** |
| --- | --- | --- |
| PHYRE2  (Protein Homology/analogY Recognition Engine) | Uses new *ab initio* folding simulation called Poing Models regions of the proteins with no detectable homology | http://www.sbg.bio.ic.ac.uk/phyre2/html/page.cgi?id=index |
| CPHModels (Center for Biological Sequence Analysis, Technical University of Denmark) | Equipped with tools like Sowhat: A neural network based method to predict contacts between C-alpha atoms from the amino acid sequence. RedHom: Used find a subset with low sequence similarity in a database. Use subsets of the Brookhaven Protein Data Bank (PDB) database with low sequence similarity produced using the RedHom tool. | http://www.cbs.dtu.dk/services/CPHmodels/ |
| SWISS-MODEL - (Glaxo-Wellcome Experimental Research, Switzerland) | Fully automated protein structure homology-modelling server, accessible via the ExPASy web server, or from the program DeepView (Swiss Pdb-Viewer). | https://swissmodel.expasy.org/ |
| I-TASSER ONLINE (Iterative Threading ASSEmbly Refinement) | Multiple-threading alignments by LOMETS and iterative TASSER simulations | http://zhanglab.ccmb.med.umich.edu/I-TASSER/ |
| LOOPP (Learning, Observing and Outputting Protein P atterns) | Fold recognition program based on the collection of numerous signals, merging them into a single score, and generating atomic coordinates based on an alignment into a homologue template structure. | http://cbsuapps.tc.cornell.edu/Sequencing/seqmain.aspx |
| ESyPred3D | Automated homology-modeling program that gets benefit of the increased alignment performances of a new alignment strategy using neural networks. Alignments are obtained by combining, weighting and screening the results of several multiple alignment programs. | http://www.unamur.be/sciences/biologie/urbm/bioinfo/esypred/ |
| (PS)2: Protein Structure Prediction Server Version 3.0 | Protein structure prediction server predicts the three-dimensional structures of protein complexes based on comparative modeling; furthermore, this server examines the coupling between subunits of the predicted complex by combining structural and evolutionary considerations. | http://ps2v3.life.nctu.edu.tw/ |
| AS2TS system | Uses the LGA (local-global alignment) program to search for regions of local similarity and to evaluate the level of structural similarity between compared protein structures | http://voronoi.hanyang.ac.kr/betacavityweb/ |
| RaptorX | Predicts its secondary and tertiary structures as well as contact map, solvent accessibility, disordered regions and binding sites. RaptorX assigns the following confidence scores to indicate the quality of a predicted 3D model: P-value for the relative global quality, GDT (global distance test) and uGDT (un-normalized GDT) for the absolute global quality, and RMSD for the absolute local quality of each residue in the model. RaptorX-Binding predicts the binding sites of a protein sequence, based upon the predicted 3D model by RaptorX | http://raptorx.uchicago.edu/ |
| PSIPRED Protein Sequence Analysis Workbench | PSIPRED is a simple and accurate secondary structure prediction method, incorporating two feed-forward neural networks which perform an analysis on output obtained from PSI-BLAST (Position Specific Iterated - BLAST). | http://bioinf.cs.ucl.ac.uk/psipred/?disopred=1 |
