# Supplementary material for: Plant Aquaporins: Genome-Wide Identification, Transcriptomics, Proteomics, and Advanced Analytical Tools
Source: Front Plant Sci. 2016 Dec 20;7:1896. doi: 10.3389/fpls.2016.01896 (PMC5167727; doi:10.3389/fpls.2016.01896)
Supplement: Table S4 — List of selected studies describing the effect of heterologous expression of aquaporins in plants. [file Table4.DOCX]

**Table S4** List of selected studies describing the effect of heterologous expression of aquaporins in plants

| **S. No.** | **AQP** | **Species** | **Transgenic plants** | **Effect of transgene** | **Reference** |
| --- | --- | --- | --- | --- | --- |
| 1 | HvPIP2;1 | Barley | Rice | Increased internal CO2 conductance and CO2 assimilation in the leaves of transgenic rice plants | (Hanba et al., 2004) |
| 2 | PIP1b | Arabidopsis | Tobacco | Under normal growth condition increased vigour but no effect under salt stress and deleterious effect during drought stress | (Aharon et al., 2003) |
| 3 | HvPIP2;1 | Barley | Rice | Raised salt sensitivity in transgenic rice plants | (Katsuhara et al., 2003) |
| 4 | PgTIP1 | *Panax ginseng* | Arabidopsis | Beneficial effect on salt-stress tolerance, drought tolerance and cold acclimation ability in transgenic Arabidopsis plants | (Peng et al., 2007) |
| 5 | PIP1;4, PIP2;5 | Arabidopsis | Arabidopsis and Tobacco | Enhanced cold tolerance, increased susceptibility to drought | (Jang et al., 2007a) |
| 6 | *SlTIP2;2* | Tomato | Arabidopsis and tomato | Significant increase in fruit yield, harvest index and plant mass relative to the control under both normal and water-stress conditions | (Sade et al., 2009) |
| 7 | *TaNIP* | Wheat | Arabidopsis | Enhanced salt tolerance | (Gao et al., 2010) |
| 8 | NtAQP1 | Tobacco | Tobacco | Enhanced photosynthesis due to different leaf mesophyll conductances to CO_2_, | (Flexas et al., 2006) |
| 9 | NtAQP1 | Tobacco | Tobacco | Lowered the CO_2_ permeability of the inner chloroplast membrane | (Uehlein et al., 2008) |
| 10 | *TaAQP8* | Wheat | Tobacco | Enhanced salt tolerance | (Hu et al., 2012) |
| 11 | *RcPIP2;1* and *RcPIP2;2*, | *Rhododendron catawbiense* | Arabidopsis | Freezing tolerance and cold acclimation | (Bots et al., 2005) |
| 12 | *TaAQP7* | Wheat | Tobacco | Enhanced drought tolerance | (Zhou et al., 2012) |
| 13 | NtAQP1 | Tobacco | Tobacco | Increases membrane permeability for CO2 and water, and increases leaf growth | (Uehlein et al., 2003) |
| 14 | *MusaPIP1;2* | Banana | Banana | lowered malondialdehyde levels, elevated proline and relative water content and higher photosynthetic efficiency | (Sreedharan et al., 2013) |
| 15 | OsNIP2;1 (Lsi1) | Rice | Rice | Enhanced silicon uptake that resulted into increased yield | (Ma et al., 2006) |
| 16 | TIP1;1 | Arabidopsis | Arabidopsis | TIP1;1 RNAi plants also contained high starch and apoplastic carbohydrate increased, role in vesicle-based metabolite routing | (Ma et al., 2004) |
| 17 | *VfPIP1* | *Vicia faba* | Arabidopsis | Enhanced drought tolerance | (Cui et al., 2008) |
| 18 | PgTIP1 | *Panax ginseng* | Arabidopsis | Enhanced seed size and seed mass plus greatly increased growth rate | (Lin et al., 2007) |
| 19 | PIP2;5 and PIP1;4 | Arabidopsis | Arabidopsis | responded to high irradiance with an increase in transpiration rates | (Lee et al., 2009) |
| 20 | HvPIP2;1 | Barley | rice | Increased CO_2_ assimilation | (Katsuhara and Hanba, 2008) |
| 21 | *Td*PIP1;1, *Td*PIP2;1 | Durum wheat | Tobacco | Increased tolerance phenotype towards osmotic and salinity stress | (Ayadi et al., 2011) |
| 22 | PIP2;2 | Arabidopsis | Arabidopsis | Enhanced water uptake | (Javot et al., 2003) |
| 23 | OsPIP2;1 | Rice | Rice | Increased rice seed yield, salt resistance, root hydraulic conductivity, and seed germination rate | (Liu et al., 2013) |
| 24 | OsPIP1-1, OsPIP2-2 | Rice | Arabidopsis | Enhanced tolerance to salt and drought | (Guo et al., 2006) |
| 25 | CfPIP2;1 | Cucumber | Arabidopsis | Enhanced tolerance to salt and drought | (Jang et al., 2007b) |
| 26 | *AtTIP2;1* | Arabidopsis | Arabidopsis | Enhanced ammonium uptake | (Loqué et al., 2005) |
| 27 | *MaPIP1;1* | Banana | Arabidopsis | Enhanced tolerance to drought | (Xu et al., 2014) |
| 28 | OsPIP2;4, OsPIP2;6, and OsPIP2;7 | Rice | Arabidopsis | Enhanced arsenite tolerance and higher biomass accumulation | (Mosa et al., 2012) |
| 29 | EaNIP3;1, EaNIP3;3, EaNIP3;4 | Horsetail | Arabidopsis | Enhanced silicon uptake | (Grégoire et al., 2012) |
| 30 | RsPIP1;1, RsPIP2;1, | Raphanus sativus | Eucalyptus grandis and Eucalyptus urophylla | Enhanced photosynthesis and growth | (Tsuchihira et al., 2010) |
| 31 | HmVALT, HmPALT1 | Hydrangea macrophylla | Arabidopsis | Enhanced Aluminum tolerance | (Negishi et al., 2012) |
| 32 | TIP1;3, TIP5;1 | Arabidopsis | Arabidopsis | Nitrogen recycling in pollen tubes of Arabidopsis thaliana | (Soto et al., 2010) |
| 33 | TsTIP1;2 | Thellungiella salsuginea | Arabidopsis | Enhanced tolerance to salt, drought, and oxidative stress | (Wang et al., 2014) |
| 34 | *PIP1* | Poplar | Poplar (RNAi) | Affect CO2 permeability | (Secchi and Zwieniecki, 2013) |
| 35 | McMIPB | Mesembryanthemum crystallinum | Tobacco | Affect CO2 transporter, as well as control the regulation of stomata to water deficits | (Kawase et al., 2013) |
| 36 | *TaTIP2;2* | Wheat | Arabidopsis | Enhanced tolerance to salt and drought | (Xu et al., 2013) |
| 37 | *MusaPIP2;6* | Banana | Banana | Displayed better photosynthetic efficiency and lower membrane damage under salt stress conditions | (Sreedharan et al., 2015) |
| 38 | *NtAQP1, AtHXK1* | Tobacco, Arabidopsis | Tobacco | NtAQP1 significantly improved growth and increased the transpiration rates of AtHXK1-expressing plants | (Kelly et al., 2014) |
| 39 | NtAQP1 | Tobacco | Tobacco | Diurnal epinastic leaf movement was shown to be reduced in transgenic tobacco lines with an impaired expression of NtAQP1 | (Siefritz et al., 2004) |
| 40 | AtTIP1;3, AtTIP5;1, | Arabidopsis | Transfer DNA insertion (T-DNA) mutant Arabidopsis | Displayed an abnormal rate of barren siliques, this phenotype being more pronounced under limited water or nutrient supply | (Wudick et al., 2014) |
| 41 | AtPIP2;1-GFP and AtPIP1;4-mCherry | Arabidopsis | Arabidopsis | ER-retained AtPIP2;1-GFP interact with other PIPs; reduced the root hydraulic conductivity | (Sorieul et al., 2011) |
| 42 | *NIP5;1* | Arabidopsis | T-DNA mutant Arabidopsis | Enhance boron uptake | (Takano et al., 2006) |
| 43 | *CaAQP* | Pepper | Pepper, over expression and VIGS | Decreased chilling stress in transgenic plants | (Yin et al., 2014) |
| 44 | *AtTIP1;1*, TRANSLUCENT GREEN (TG) | Arabidopsis | Arabidopsis | TG regulates expression of three TIP genes in Arabidopsis leaves | (Zhu et al., 2014) |
| 45 | HvLsi1 | Barley | Rice lsi1 mutant | Enhanced uptake of silicon | (Chiba et al., 2009; Zhu et al., 2014) |
| 46 | AtPIP1;2 | Arabidopsis | T-DNA mutant Arabidopsis | Regulate membrane CO_2_transport | (Uehlein et al., 2012) |
| 47 | TaLsi1 | Wheat | Arabidopsis | Enhanced silicon uptake | (Montpetit et al., 2012) |
| 48 | PtNIP2-1, SlNIP2-1 (mutated) | Poplar, Tomato | Arabidopsis | Enhanced silicon uptake | (Deshmukh et al., 2015) |
| 49 | GmNIP2-2 | Soybean | Arabidopsis | Enhanced silicon uptake | (Deshmukh et al., 2013) |
| 50 | AtPIP1;4 | Arabidopsis | Arabidopsis | Affected apoplastic H2O2 induction leading to  Induction of disease immunity pathways | (Tian et al., 2016) |

Aharon, R., Shahak, Y., Wininger, S., Bendov, R., Kapulnik, Y., and Galili, G. (2003). Overexpression of a plasma membrane aquaporin in transgenic tobacco improves plant vigor under favorable growth conditions but not under drought or salt stress. *The Plant Cell* 15**,** 439-447.

Ayadi, M., Cavez, D., Miled, N., Chaumont, F., and Masmoudi, K. (2011). Identification and characterization of two plasma membrane aquaporins in durum wheat (Triticum turgidum L. subsp. durum) and their role in abiotic stress tolerance. *Plant Physiology and Biochemistry* 49**,** 1029-1039.

Bots, M., Vergeldt, F., Wolters-Arts, M., Weterings, K., Van As, H., and Mariani, C. (2005). Aquaporins of the PIP2 class are required for efficient anther dehiscence in tobacco. *Plant Physiology* 137**,** 1049-1056.

Chiba, Y., Mitani, N., Yamaji, N., and Ma, J.F. (2009). HvLsi1 is a silicon influx transporter in barley. *The Plant Journal* 57**,** 810-818.

Cui, X.-H., Hao, F.-S., Chen, H., Chen, J., and Wang, X.-C. (2008). Expression of the Vicia faba VfPIP1 gene in Arabidopsis thaliana plants improves their drought resistance. *Journal of plant research* 121**,** 207-214.

Deshmukh, R.K., Vivancos, J., Guérin, V., Sonah, H., Labbé, C., Belzile, F., and Bélanger, R.R. (2013). Identification and functional characterization of silicon transporters in soybean using comparative genomics of major intrinsic proteins in Arabidopsis and rice. *Plant molecular biology* 83**,** 303-315.

Deshmukh, R.K., Vivancos, J., Ramakrishnan, G., Guérin, V., Carpentier, G., Sonah, H., Labbé, C., Isenring, P., Belzile, F.J., and Bélanger, R.R. (2015). A precise spacing between the NPA domains of aquaporins is essential for silicon permeability in plants. *The Plant Journal* 83**,** 489-500.

Flexas, J., Ribas‐Carbó, M., Hanson, D.T., Bota, J., Otto, B., Cifre, J., Mcdowell, N., Medrano, H., and Kaldenhoff, R. (2006). Tobacco aquaporin NtAQP1 is involved in mesophyll conductance to CO2in vivo. *The Plant Journal* 48**,** 427-439.

Gao, Z., He, X., Zhao, B., Zhou, C., Liang, Y., Ge, R., Shen, Y., and Huang, Z. (2010). Overexpressing a putative aquaporin gene from wheat, TaNIP, enhances salt tolerance in transgenic Arabidopsis. *Plant and Cell Physiology* 51**,** 767-775.

Grégoire, C., Rémus‐Borel, W., Vivancos, J., Labbé, C., Belzile, F., and Bélanger, R.R. (2012). Discovery of a multigene family of aquaporin silicon transporters in the primitive plant Equisetum arvense. *The Plant Journal* 72**,** 320-330.

Guo, L., Wang, Z.Y., Lin, H., Cui, W.E., Chen, J., Liu, M., Chen, Z.L., Qu, L.J., and Gu, H. (2006). Expression and functional analysis of the rice plasma-membrane intrinsic protein gene family. *Cell research* 16**,** 277-286.

Hanba, Y.T., Shibasaka, M., Hayashi, Y., Hayakawa, T., Kasamo, K., Terashima, I., and Katsuhara, M. (2004). Overexpression of the barley aquaporin HvPIP2; 1 increases internal CO2 conductance and CO2 assimilation in the leaves of transgenic rice plants. *Plant and Cell Physiology* 45**,** 521-529.

Hu, W., Yuan, Q., Wang, Y., Cai, R., Deng, X., Wang, J., Zhou, S., Chen, M., Chen, L., and Huang, C. (2012). Overexpression of a wheat aquaporin gene, TaAQP8, enhances salt stress tolerance in transgenic tobacco. *Plant and Cell Physiology* 53**,** 2127-2141.

Jang, J.Y., Lee, S.H., Rhee, J.Y., Chung, G.C., Ahn, S.J., and Kang, H. (2007a). Transgenic Arabidopsis and tobacco plants overexpressing an aquaporin respond differently to various abiotic stresses. *Plant molecular biology* 64**,** 621-632.

Jang, J.Y., Rhee, J.Y., Kim, D.G., Chung, G.C., Lee, J.H., and Kang, H. (2007b). Ectopic expression of a foreign aquaporin disrupts the natural expression patterns of endogenous aquaporin genes and alters plant responses to different stress conditions. *Plant and cell physiology* 48**,** 1331-1339.

Javot, H., Lauvergeat, V., Santoni, V., Martin-Laurent, F., Güçlü, J., Vinh, J., Heyes, J., Franck, K.I., Schäffner, A.R., and Bouchez, D. (2003). Role of a single aquaporin isoform in root water uptake. *The plant cell* 15**,** 509-522.

Katsuhara, M., and Hanba, Y.T. (2008). Barley plasma membrane intrinsic proteins (PIP aquaporins) as water and CO2 transporters. *Pflügers Archiv-European Journal of Physiology* 456**,** 687-691.

Katsuhara, M., Koshio, K., Shibasaka, M., Hayashi, Y., Hayakawa, T., and Kasamo, K. (2003). Over-expression of a barley aquaporin increased the shoot/root ratio and raised salt sensitivity in transgenic rice plants. *Plant and Cell Physiology* 44**,** 1378-1383.

Kawase, M., Hanba, Y.T., and Katsuhara, M. (2013). The photosynthetic response of tobacco plants overexpressing ice plant aquaporin McMIPB to a soil water deficit and high vapor pressure deficit. *Journal of plant research* 126**,** 517-527.

Kelly, G., Sade, N., Attia, Z., Secchi, F., Zwieniecki, M., Holbrook, N.M., Levi, A., Alchanatis, V., Moshelion, M., and Granot, D. (2014). Relationship between hexokinase and the aquaporin PIP1 in the regulation of photosynthesis and plant growth. *PloS one* 9**,** e87888.

Lee, S.H., Chung, G.C., and Zwiazek, J.J. (2009). Effects of irradiance on cell water relations in leaf bundle sheath cells of wild-type and transgenic tobacco (Nicotiana tabacum) plants overexpressing aquaporins. *Plant Science* 176**,** 248-255.

Lin, W., Peng, Y., Li, G., Arora, R., Tang, Z., Su, W., and Cai, W. (2007). Isolation and functional characterization of PgTIP1, a hormone-autotrophic cells-specific tonoplast aquaporin in ginseng. *Journal of experimental botany* 58**,** 947-956.

Liu, C., Fukumoto, T., Matsumoto, T., Gena, P., Frascaria, D., Kaneko, T., Katsuhara, M., Zhong, S., Sun, X., and Zhu, Y. (2013). Aquaporin OsPIP1; 1 promotes rice salt resistance and seed germination. *Plant Physiology and Biochemistry* 63**,** 151-158.

Loqué, D., Ludewig, U., Yuan, L., and Von Wirén, N. (2005). Tonoplast intrinsic proteins AtTIP2; 1 and AtTIP2; 3 facilitate NH3 transport into the vacuole. *Plant physiology* 137**,** 671-680.

Ma, J.F., Tamai, K., Yamaji, N., Mitani, N., Konishi, S., Katsuhara, M., Ishiguro, M., Murata, Y., and Yano, M. (2006). A silicon transporter in rice. *Nature* 440**,** 688-691.

Ma, S., Quist, T.M., Ulanov, A., Joly, R., and Bohnert, H.J. (2004). Loss of TIP1; 1 aquaporin in Arabidopsis leads to cell and plant death. *The Plant Journal* 40**,** 845-859.

Montpetit, J., Vivancos, J., Mitani-Ueno, N., Yamaji, N., Rémus-Borel, W., Belzile, F., Ma, J.F., and Bélanger, R.R. (2012). Cloning, functional characterization and heterologous expression of TaLsi1, a wheat silicon transporter gene. *Plant molecular biology* 79**,** 35-46.

Mosa, K.A., Kumar, K., Chhikara, S., Mcdermott, J., Liu, Z., Musante, C., White, J.C., and Dhankher, O.P. (2012). Members of rice plasma membrane intrinsic proteins subfamily are involved in arsenite permeability and tolerance in plants. *Transgenic research* 21**,** 1265-1277.

Negishi, T., Oshima, K., Hattori, M., Kanai, M., Mano, S., Nishimura, M., and Yoshida, K. (2012). Tonoplast-and plasma membrane-localized aquaporin-family transporters in blue hydrangea sepals of aluminum hyperaccumulating plant. *PLoS One* 7**,** e43189.

Peng, Y., Lin, W., Cai, W., and Arora, R. (2007). Overexpression of a Panax ginseng tonoplast aquaporin alters salt tolerance, drought tolerance and cold acclimation ability in transgenic Arabidopsis plants. *Planta* 226**,** 729-740.

Sade, N., Vinocur, B.J., Diber, A., Shatil, A., Ronen, G., Nissan, H., Wallach, R., Karchi, H., and Moshelion, M. (2009). Improving plant stress tolerance and yield production: is the tonoplast aquaporin SlTIP2; 2 a key to isohydric to anisohydric conversion? *New Phytologist* 181**,** 651-661.

Secchi, F., and Zwieniecki, M.A. (2013). The physiological response of Populus tremula x alba leaves to the down-regulation of PIP1 aquaporin gene expression under no water stress. *Frontiers in plant science* 4**,** 507.

Siefritz, F., Otto, B., Bienert, G.P., Van Der Krol, A., and Kaldenhoff, R. (2004). The plasma membrane aquaporin NtAQP1 is a key component of the leaf unfolding mechanism in tobacco. *The Plant Journal* 37**,** 147-155.

Sorieul, M., Santoni, V., Maurel, C., and Luu, D.T. (2011). Mechanisms and Effects of Retention of Over‐Expressed Aquaporin AtPIP2; 1 in the Endoplasmic Reticulum. *Traffic* 12**,** 473-482.

Soto, G., Fox, R., Ayub, N., Alleva, K., Guaimas, F., Erijman, E.J., Mazzella, A., Amodeo, G., and Muschietti, J. (2010). TIP5; 1 is an aquaporin specifically targeted to pollen mitochondria and is probably involved in nitrogen remobilization in Arabidopsis thaliana. *The Plant Journal* 64**,** 1038-1047.

Sreedharan, S., Shekhawat, U.K., and Ganapathi, T.R. (2013). Transgenic banana plants overexpressing a native plasma membrane aquaporin MusaPIP1; 2 display high tolerance levels to different abiotic stresses. *Plant biotechnology journal* 11**,** 942-952.

Sreedharan, S., Shekhawat, U.K.S., and Ganapathi, T.R. (2015). Constitutive and stress-inducible overexpression of a native aquaporin gene (MusaPIP2; 6) in transgenic banana plants signals its pivotal role in salt tolerance. *Plant molecular biology* 88**,** 41-52.

Takano, J., Wada, M., Ludewig, U., Schaaf, G., Von Wirén, N., and Fujiwara, T. (2006). The Arabidopsis major intrinsic protein NIP5; 1 is essential for efficient boron uptake and plant development under boron limitation. *The Plant Cell* 18**,** 1498-1509.

Tian, S., Wang, X., Li, P., Wang, H., Ji, H., Xie, J., Qiu, Q., Shen, D., and Dong, H. (2016). Plant Aquaporin AtPIP1; 4 Links Apoplastic H2O2 Induction to Disease Immunity Pathways. *Plant physiology***,** pp. 01237.02015.

Tsuchihira, A., Hanba, Y.T., Kato, N., Doi, T., Kawazu, T., and Maeshima, M. (2010). Effect of overexpression of radish plasma membrane aquaporins on water-use efficiency, photosynthesis and growth of Eucalyptus trees. *Tree physiology* 30**,** 417-430.

Uehlein, N., Lovisolo, C., Siefritz, F., and Kaldenhoff, R. (2003). The tobacco aquaporin NtAQP1 is a membrane CO2 pore with physiological functions. *Nature* 425**,** 734-737.

Uehlein, N., Otto, B., Hanson, D.T., Fischer, M., Mcdowell, N., and Kaldenhoff, R. (2008). Function of Nicotiana tabacum aquaporins as chloroplast gas pores challenges the concept of membrane CO2 permeability. *The Plant Cell* 20**,** 648-657.

Uehlein, N., Sperling, H., Heckwolf, M., and Kaldenhoff, R. (2012). The Arabidopsis aquaporin PIP1; 2 rules cellular CO2 uptake. *Plant, cell & environment* 35**,** 1077-1083.

Wang, L.-L., Chen, A.-P., Zhong, N.-Q., Liu, N., Wu, X.-M., Wang, F., Yang, C.-L., Romero, M.F., and Xia, G.-X. (2014). The Thellungiella salsuginea tonoplast aquaporin TsTIP1; 2 functions in protection against multiple abiotic stresses. *Plant and Cell Physiology* 55**,** 148-161.

Wudick, M.M., Luu, D.-T., Tournaire-Roux, C., Sakamoto, W., and Maurel, C. (2014). Vegetative and sperm cell-specific aquaporins of Arabidopsis highlight the vacuolar equipment of pollen and contribute to plant reproduction. *Plant physiology* 164**,** 1697-1706.

Xu, C., Wang, M., Zhou, L., Quan, T., and Xia, G. (2013). Heterologous expression of the wheat aquaporin gene TaTIP2; 2 compromises the abiotic stress tolerance of Arabidopsis thaliana. *PloS one* 8**,** e79618.

Xu, Y., Hu, W., Liu, J., Zhang, J., Jia, C., Miao, H., Xu, B., and Jin, Z. (2014). A banana aquaporin gene, MaPIP1; 1, is involved in tolerance to drought and salt stresses. *BMC plant biology* 14**,** 1.

Yin, Y.-X., Guo, W.-L., Zhang, Y.-L., Ji, J.-J., Xiao, H.-J., Yan, F., Zhao, Y.-Y., Zhu, W.-C., Chen, R.-G., and Chai, W.-G. (2014). Cloning and characterisation of a pepper aquaporin, CaAQP, which reduces chilling stress in transgenic tobacco plants. *Plant Cell, Tissue and Organ Culture (PCTOC)* 118**,** 431-444.

Zhou, S., Hu, W., Deng, X., Ma, Z., Chen, L., Huang, C., Wang, C., Wang, J., He, Y., and Yang, G. (2012). Overexpression of the wheat aquaporin gene, TaAQP7, enhances drought tolerance in transgenic tobacco. *PLoS One* 7**,** e52439.

Zhu, D., Wu, Z., Cao, G., Li, J., Wei, J., Tsuge, T., Gu, H., Aoyama, T., and Qu, L.-J. (2014). TRANSLUCENT GREEN, an ERF family transcription factor, controls water balance in Arabidopsis by activating the expression of aquaporin genes. *Molecular plant* 7**,** 601-615.
