# Supplementary material for: Plant Aquaporins: Genome-Wide Identification, Transcriptomics, Proteomics, and Advanced Analytical Tools
Source: Front Plant Sci. 2016 Dec 20;7:1896. doi: 10.3389/fpls.2016.01896 (PMC5167727; doi:10.3389/fpls.2016.01896)
Supplement: Supplementary file 6 [file DataSheet1.PDF]

```
LOCUS       AlNIP1-1       295 aa
DEFINITION  AlNIP1-1     295 aa
TITLE       AlNIP1-1
ORIGIN

        1 MADISGNGYG NAREVVMVNL KEEVEHQQEM EAIHNPKPLK KQDSLLSVSV PFLQKLIAES
       61 LGTYFLVFTG CASVVVNMQN DNVVTLPGIA IVWGLTIMVL IYSLGHISGA HINPAVTIAF
      121 ASCGRFPLKQ VPAYVISQVI GSTLAAATLR LLFGLDHDVC SGKHDVFIGS SPVGSDLQAF
      181 VMEFIVTFYL MFIISGVATD NRAIGELAGL AIGSTVLLNV LIAAPVSSAS MNPGRSLGPA
      241 MVYGCYKGIW IYIVAPTLGA IAGAWVYNTV RYTDKPLREI TKSGSFLKSV RIGSS
//
LOCUS       AlNIP1-2       298 aa
DEFINITION  AlNIP1-2     298 aa
TITLE       AlNIP1-2
ORIGIN

        1 MAEISGNGHG ADARDGAVVV NLKEEDEHQQ QKEAIHNPKP MKKQDSLLSI SVPFLQKLMA
       61 EVLGTYFLIF AGCAAVAVNT QHDKAVTLPG IAIVWGLTVM VLVYSLGHIS GAHFNPAVTI
      121 AFASCGRFPL KQVPAYVISQ VIGSTLAAAT LRLLFGLDQD VCSGKHDVFV GTLPSGSDLQ
      181 SFVIEFIITF YLMFVISGVA TDNRAIGELA GLAVGSTVLL NVIIAGPVSG ASMNPGRSLG
      241 PAMVYSCYRG LWIYIVSPIV GAVSGAWVYN MVRYTDKPLR EITKSGSFLK TVRNGSSR
//
LOCUS       AlNIP2-1       258 aa
DEFINITION  AlNIP2-1     258 aa
TITLE       AlNIP2-1
ORIGIN

        1 MDDISVSKSN HGNVVVLNIE ASSVADTKNE SSSSPLISVH FLQKLMAELV GTYYLIFAGC
       61 AAIAVNAQHN HVVTLVGIAV VWGIVVMVLV YCLGHISAHF NPAVTLALAS SQRFPLNQVP
      121 AYITVQVIGS TLASATLRLL FDLNNDFIIT GFLMLVVCAV TTTKKTTEEL EGLIIGAAVT
      181 LNVIFAGEVS GASMNPARSI GPALVWGCYK GIWIYLLAPT LGAVSGALIH KMLPSIQNAE
      241 PEFSKTGSSH KRVTDLPL
//
LOCUS       AlNIP3-1       269 aa
DEFINITION  AlNIP3-1     269 aa
TITLE       AlNIP3-1
ORIGIN

        1 MIFAGCSAIV VNETYGKPVT LPGIALVWGL TVTVMIYSIG HVSGAHFNPA VSIAFASSKK
       61 FPFNQVPGYI AAQVLGSTLA AAALRLVFHL NDDVCSLKGD VYVGTYPSNS TTTSFVMEFI
      121 ATFNLMFVIS AVATDKRATG SLAGIAIGAT VVLDILFSGP ISGASMNPAR SLGPALIWGC
      181 YKDLWLYIIS PVLGALSGAW TYDLLRSTKK SYSEIIRPNC NKVSSRDHQE ASQDEICVLR
      241 VVDPANQNYF ICSSPNDING KCNVTCKLA
//
LOCUS       AlNIP4-1       283 aa
DEFINITION  AlNIP4-1     283 aa
TITLE       AlNIP4-1
ORIGIN

        1 MSLHSDEIEE EQISRIEKGK GKDCHGGIET VICTSPSIVC LTQKLIAEMI GTYFIVFSGC
       61 GVVVVNVLYG GTITFPGICV TWGLIVMVMI YSTGHISGAH FNPAVTVTFA IFRRFPWYQV
      121 PLYIGAQFAG SLLASLTLRL MFKVTPEAFF GTTPADSPAR ALVAEIIISF LLMFVISGVA
      181 TDNRAVGELA GIAVGMTIML NVFVAGPISG ASMNPARSLG PALVMGVYTH IWVYILGPVL
      241 GVISGGFVYN LIRFTDKPLR ELTKSASFLR AVSPSHKASS SKT
```

```
//
LOCUS       AlNIP4-2       283 aa
DEFINITION  AlNIP4-2     283 aa
TITLE       AlNIP4-2
ORIGIN

        1 MTSHCEEIEA EQISRIEKGN GKDSQGGIET AICTSPSIVC LTQKLIAEMI GTYFIIFSGC
       61 GVVVVNVLYG GTITFPGICV TWGLIVMVMI YSTGHISGAH FNPAVTVTFA VFRRFPWFQV
      121 PLYIGAQLTG SLLASLTLRL MFNVTPKAFF GTSPTDSSGQ ALVAEIIISF LLMFVISGVA
      181 TDSRATGELA GIAVGMTIIL NVFVAGPISG ASMNPARSLG PAIVMGRYKG IWVYIVGPFV
      241 GIFAGGFVYN FMRFTDKPLR ELTKSASFLR SVAQKNNASK SEG
//
LOCUS       AlNIP5-1       304 aa
DEFINITION  AlNIP5-1     304 aa
TITLE       AlNIP5-1
ORIGIN

        1 MAPPEAEVGS VVVMAPPTPG TPGTPGGPLI TGMRVDSMSF DHRKPTPRCK CLPVMGSTWG
       61 QHDTCFTDFP SPDVSLTRKL GAEFVGTFIL IFTATAGPIV NQKYDGAETL IGNAACAGLA
      121 VMIIILSTGH ISGAHLNPSL TIAFAALRHF PWAHVPAYIA AQVSASICAS FALKGVFHPF
      181 MSGGVTVPSV GVGQAFALEF IITFILLFVV TAVATDTRAV GELAGIAVGA TVMLNILVAG
      241 PSTGGSMNPV RTLGPAVASG NYRSLWVYLV APTLGAIAGA AVYTGVKLND SVTDPPRQVR
      301 SFRR
//
LOCUS       AlNIP6-1       305 aa
DEFINITION  AlNIP6-1     305 aa
TITLE       AlNIP6-1
ORIGIN

        1 MDHEEIPSMP STPATTPGTP GAPLFGGFEG KRNGHNGKYT PKSLLKSCKC FSVDNEWALE
       61 DGRLPPVSCS LPPPNVSLYR KLGAEFVGTL ILIFAGTATA IVNQKTDGAE TLIGCAASAG
      121 LAVMIVILST GHISGAHLNP AVTIAFAALK HFPWKHVPVY IGAQVMASVC AAFALKAVFE
      181 PTMSGGVTVP TVGLSQAFAL EFIISFNLMF VVTAVATDTR AVGELAGIAV GATVMLNILI
      241 AGPATSASMN PVRTLGPAIA ANNYRAIWVY LTAPILGALI GAGTYTIVKL PEENEAPKER
      301 RSFRR
//
LOCUS       AlNIP6-2       242 aa
DEFINITION  AlNIP6-2     242 aa
TITLE       AlNIP6-2
ORIGIN

        1 MDHEEIPSMP STPATTPGTP GAPLFGGFEG KRNGHNGKYT PKSLLKSCKC FSVDNEWALE
       61 DGRLPPVSCS LPPPNVSLYR KLGAEFVGTL ILIFAGTATA IVNQKTDGAE TLIGCAASAG
      121 LAVMIVILST GHISGAHLNP AVTIAFAALK HFPWKHVPVY IGAQVMASVC AAFALKAVFE
      181 PTMSGGVTVP TVGLSQAFAL EFIISFNLMF VVTAVATDTR AVGELAGIAV GATVMLNILI
      241 AG
//
LOCUS       AlNIP7-1       275 aa
DEFINITION  AlNIP7-1     275 aa
TITLE       AlNIP7-1
ORIGIN

        1 MNGEARSRVV DQEAGSTPST LRDEDHPSRQ RLFGCLPYDI DLNPIRIVMA EFVGTFILMF
       61 SVCGVISSTQ LSGGHVGLLE YAATAGLSVV VVVYSIGHIS GAHLNPSITI AFAVFGGFPW
```

```
      121 SQVPLYITAQ TLGATAATLV GVSVYGVNAD IMATKPALSC VSAFFVELIA TSIVVFLASA
      181 LHCGPHQNLG NLTGFVIGTV ISLGVLITGP ISGGSMNPAR SLGPAVVAWD FEDLWIYMTA
      241 PVIGAIIGVL TYRSISLKTR PCLSPLSPSV SSLLR
//
LOCUS       AlPIP1-1       286 aa
DEFINITION  AlPIP1-1     286 aa
TITLE       AlPIP1-1
ORIGIN

        1 MEGKEEDVRV GANKFPERQP IGTSAQSDKD YKEPPPAPLF EPGEIASWSF WRAGIAEFIA
       61 TFLFLYITVL TVMGVKRSPN MCASVGIQGI AWAFGGMIFA LVYCTAGISG GHINPAVTFG
      121 LFLARKLSLT RAVYYIVMQC LGAICGAGVV KGFQPKQYQA LGGGANTIAH GYTKGSGLGA
      181 EIIGTFVLVY TVFSATDAKR NARDSHVPIL APLPIGFAVF LVHLATIPIT GTGINPARSL
      241 GAAIIFNKDN AWDDHWVFWV GPFIGAALAA LYHVIVIRAI PFKSRS
//
LOCUS       AlPIP1-2       286 aa
DEFINITION  AlPIP1-2     286 aa
TITLE       AlPIP1-2
ORIGIN

        1 MEGKEEDVRV GANKFPERQP IGTSAQSDKD YNEPPPAPFF EPGELSSWSF WRAGIAEFIA
       61 TFLFLYITVL TVMGVKRSPN MCASVGIQGI AWAFGGMIFA LVYCTAGISG GHINPAVTFG
      121 LFLARKLSLT RALYYIVMQC LGAICGAGVV KGFQPKQYQA LGGGANTVAH GYTKGSGLGA
      181 EIIGTFVLVY TVFSATDAKR NARDSHVPIL APLPIGFAVF LVHLATIPIT GTGINPARSL
      241 GAAIIYNKDH SWDDHWVFWV GPFIGAALAA LYHVVVIRAI PFKSRS
//
LOCUS       AlPIP1-3       286 aa
DEFINITION  AlPIP1-3     286 aa
TITLE       AlPIP1-3
ORIGIN

        1 MEGKEEDVRV GANKFPERQP IGTSAQTDKD YKEPPPAPLF EPGELSSWSF YRAGIAEFIA
       61 TFLFLYITVL TVMGVKRAPN MCASVGIQGI AWAFGGMIFA LVYCTAGISG GHINPAVTFG
      121 LFLARKLSLT RAVFYIVMQC LGAICGAGVV KGFQPNPYQT LGGGANTVAH GYTKGSGLGA
      181 EIIGTFVLVY TVFSATDAKR SARDSHVPIL APLPIGFAVF LVHLATIPIT GTGINPARSL
      241 GAAIIYNKDH AWDDHWIFWV GPFIGAALAA LYHQLVIRAI PFKSRT
//
LOCUS       AlPIP1-4       287 aa
DEFINITION  AlPIP1-4     287 aa
TITLE       AlPIP1-4
ORIGIN

        1 MEGKEEDVRV GANKFPERQP IGTSAQSTDK DYKEPPPAPL FEPGELSSWS FYRAGIAEFI
       61 ATFLFLYITV LTVMGVKRAP NMCASVGIQG IAWAFGGMIF ALVYCTAGIS GGHINPAVTF
      121 GLFLARKLSL TRAVFYMIMQ CLGAICGAGV VKGFQPTHYQ TLGGGANTVA HGYTKGSGLG
      181 AEIIGTFVLV YTVFSATDAK RSARDSHVPI LAPLPIGFAV FLVHLATIPI TGTGINPARS
      241 LGAAIIYNKD HSWDDHWIFW VGPFIGAALA ALYHQIVIRA IPFKSKS
//
LOCUS       AlPIP1-5       287 aa
DEFINITION  AlPIP1-5     287 aa
TITLE       AlPIP1-5
ORIGIN

        1 MEGKEEDVNV GANKFPERQP IGTAAQTESK DYKEPPPAPF FEPGELKSWS FYRAGIAEFI
```

```
       61 ATFLFLYVTV LTVMGVKRAP NMCASVGIQG IAWAFGGMIF ALVYCTAGIS GGHINPAVTF
      121 GLFLARKLSL TRALFYIVMQ CLGAICGAGV VKGFQPGLYQ TNGGGANVVA HGYTKGSGLG
      181 AEIVGTFVLV YTVFSATDAK RSARDSHVPI LAPLPIGFAV FLVHLATIPI TGTGINPARS
      241 LGAAIIYNKG HAWDDHWIFW VGPFIGAALA ALYHQIVIRA IPFKSKT
//
LOCUS       AlPIP1-6       155 aa
DEFINITION  AlPIP1-6     155 aa
TITLE       AlPIP1-6
ORIGIN

        1 MGQEYIEQVS DKICSFGGLA PGGERNRCLR SLFWLGDTNR RCGAGVVKGF QPGLYQANGG
       61 GANVVAHGYT KGSGLGAEII GTFVLVYTVF SATDAKRSAR DSHVHILVPL PIGFAVFLVH
      121 LATIPITGTS INPARSLGAA IIYNKDHAWD DHVNA
//
LOCUS       AlPIP2-1       287 aa
DEFINITION  AlPIP2-1     287 aa
TITLE       AlPIP2-1
ORIGIN

        1 MAKDVEAVPG EGFQTRDYHD PPPAPFIDGA ELKKWSFYRA VIAEFVATLL FLYITVLTVI
       61 GYKIQSDTTA GGVDCGGVGI LGIAWAFGGM IFILVYCTAG ISGGHINPAV TFGLFLARKV
      121 SLPRAILYIV AQCLGAICGV GFVKAFQSSY YDRYGGGANS LADGYSTGTG LAAEIIGTFV
      181 LVYTVFSATD PKRSARDSHV PVLAPLPIGF AVFMVHLATI PITGTGINPA RSFGAAVIFN
      241 KSKPWDDHWI FWVGPFIGAA IAAFYHQFVL RASGSKSLGS FRSAANV
//
LOCUS       AlPIP2-2       285 aa
DEFINITION  AlPIP2-2     285 aa
TITLE       AlPIP2-2
ORIGIN

        1 MAKDVEGPEG FQTRDYEDPP PTPFFDAEEL TKWSLYRAVI AEFVATLLFL YVTVLTVIGY
       61 KIQSDKTAGG VDCGGVGILG IAWAFGGMIF ILVYCTAGIS GGHINPAVTF GLFLARKVSL
      121 IRAVLYMVAQ CLGAICGVGF VKAFQSSYYV RYGGGANSLA DGYNTGTGLA AEIIGTFVLV
      181 YTVFSATDPK RNARDSHVPV LAPLPIGFAV FMVHLATIPI TGTGINPARS FGAAVIFNES
      241 KPWDDHWIFW VGPFIGAAIA AFYHQFVLRA SGSKSLGSFR SAANV
//
LOCUS       AlPIP2-3       261 aa
DEFINITION  AlPIP2-3     261 aa
TITLE       AlPIP2-3
ORIGIN

        1 MAKDVGGPDG FQTRDYEDPP PTPFFDAEEL TKWSLYRAVI AEFVATLLFL YVTVLTVIGY
       61 KYKIELTVAA LEFLASRGHI NPAVTFGLFL ARKVSLIRAV LYMVAQCLGA ICGVGFVKAF
      121 QSSYYVRYGG GANSLADGYN TGTGLAAEII GTFVLVYTVF SATDPKRNAR DSHVPVLAPL
      181 PIGFAVFMVH LATIPITGTG INPARSFGAA VIFNKSKPWD DHWIFWVGPF IGAAIAAFYH
      241 QFVLRASGSK SLGSFRSAAN V
//
LOCUS       AlPIP2-4       285 aa
DEFINITION  AlPIP2-4     285 aa
TITLE       AlPIP2-4
ORIGIN

        1 MAKDDDVQEG GAPAARDYRD PPPAPLLDME EIRKWSLYRA VIAEFVATLL FLYVSVLTVI
       61 GYKAQTDATA GGVDCGGVGI LGIAWAFGGM IFVLVYCTAG ISGGHINPAV TVGLFLARKV
```

```
      121 SLVRTVLYIV AQCLGAICGC GLVKAFQSSY YTRYGGGANE LADGYNKGTG LGAEIIGTFV
      181 LVYTVFSATD PKRSARDSHV PVLAPLPIGF AVFMVHLATI PITGTGINPA RSFGAAVIYN
      241 NEKAWDDQWI FWVGPMIGAA AAAFYHQFIL RAAAIKALGS FRSFA
//
LOCUS       AlPIP2-5       289 aa
DEFINITION  AlPIP2-5     289 aa
TITLE       AlPIP2-5
ORIGIN

        1 MTKEVVGDKR SFSGKDYQDP PPEPLFDATE LGKWSFYRAL IAEFIATLLF LYVTVMTVIG
       61 YKSQTDQALN PDQCAGVGVL GIAWAFGGMI FILVYCTAGI SGSHINPAVT FGLLLARKVT
      121 LLRAVMYMVA QCLGAICGVA LVKSFQSAYY NRYGGGTNGL SDGYSIGTGV AAEIIGTFVL
      181 VYTVFSATDP KRSARDSHVP VSPVLAPLPI GFAVFIVHLA TIPITGTGIN PARSLGAAII
      241 YNKDKAWDHH WIFWVGPFAG AAIAAFYHQF VLRAGAIKAL GSFRSQPHV
//
LOCUS       AlPIP2-6       289 aa
DEFINITION  AlPIP2-6     289 aa
TITLE       AlPIP2-6
ORIGIN

        1 MTKDELTEEE SLSGKDYLDP PPVKTFEVRE LKKWSFYRAV IAEFIATFLF LYVTVLTVIG
       61 FKSQTDINAG GGACASVGLL GISWAFGGMI FILVYCTAGI SGGHINPAVT FGLFLASKVS
      121 LVRAVSYMVA QCLGATCGVG LVKVFQSTYY NRYGGGANML SEGYNVGVGV GAEIIGTFVL
      181 VYTVFSATDP KRNARDSHIP VLAPLPIGFS VFMVHLATIP ITGTGINPAR SFGAAVIYNN
      241 QKAWDDQWIF WVGPFVGAAI AAFYHQFVLR AGAMKAYGSV RSQLHELHA
//
LOCUS       AlPIP2-7       280 aa
DEFINITION  AlPIP2-7     280 aa
TITLE       AlPIP2-7
ORIGIN

        1 MSKEVSEEGK THHGKDYVDP PPAPFFDMGE LKSWSFYRAL IAEFIATLLF LYVTVATVIG
       61 HKKQTGACDG VGLLGIAWAF GGMIFVLVYC TAGISGGHIN PAVTFGLFLA RKVSLVRALG
      121 YMIAQCLGAI CGVGFVKAFM KTPYNTLGGG ANTVADGYSK GTALGAEIIG TFVLVYTVFS
      181 ATDPKRSARD SHIPVLAPLP IGFAVFMVHL ATIPITGTGI NPARSFGAAV IYNNEKAWDD
      241 QWIFWVGPFL GALAAAAYHQ YILRASAIKA LGSFRSNATN
//
LOCUS       AlPIP2-8       278 aa
DEFINITION  AlPIP2-8     278 aa
TITLE       AlPIP2-8
ORIGIN

        1 MSKEVSEEGR HGKDYVDPPP APLLDMAEIK LWSFYRAIIA EFIATLLFLY VTVATVIGHK
       61 NQNGPCDGVG LLGIAWAFGG MIFVLVYCTA GISGGHINPA VTFGLFLARK VSLPRAVAYM
      121 VAQCLGAICG VGLVKAFMMT PYKRLGGGAN TVADDYSTGT ALGAEIIGTF VLVYTVFSAT
      181 DPKRSARDSH VPVLAPLPIG FAVFMVHLAT IPITGTGINP ARSFGAAVIY NNEKAWDDHW
      241 IFWVGPFLGA LAAAAYHQYI LRAAAIKALA SFRSNPTN
//
LOCUS       AlSIP1-1       240 aa
DEFINITION  AlSIP1-1     240 aa
TITLE       AlSIP1-1
ORIGIN

        1 MMGVVKSAIG DMLMTFSWVV LSATFGIQTA AIISAGDFQG ITWAPLVILT SLIFVYVSIF
```

```
       61 TVIFGSASFN PTGNAAFYVA GIPGDTLFSL AIRLPAQAAG AAGGALAIME FIPEKYKHMI
      121 GGPSLQVDVH TGAIAETILS FGITFAVLLI ILRGPRRLLA KTFLLALATI SFVVAGSKYT
      181 GPAMNPAIAF GWAYMYSSHN TWDHIYVVYWI SSFVGALSAA LLFRSIFPPA RPQKKKQKKA
//
LOCUS       AlSIP1-2       243 aa
DEFINITION  AlSIP1-2     243 aa
TITLE       AlSIP1-2
ORIGIN

        1 MSAVKSALGD MVITFLWVIL SATFGIQTAA IVSAVGFHGI TWAPLVISTL VVFVSISIFT
       61 VIGNVLGGAS FNPCGNAAFY TAGVSRDSLF SLAIRSPAQA VGAAGGAITI MEMIPEKYKT
      121 KIGGKPSLQV GAHNGAIAEV ILSFSVTFLV LLIIILRGPRK LLAKTFLLAL ATVSVFVVGS
      181 KFTRPFMNPA IAFGWAYIYK SHNTWDHFYV YWISSYTGAI LSAMLFRILF PAPPLVQKKQ
      241 KKA
//
LOCUS       AlSIP2-1       237 aa
DEFINITION  AlSIP2-1     237 aa
TITLE       AlSIP2-1
ORIGIN

        1 MGRIGLVVSD LVLSFMWIWA GVLVNILVHG VLGFSRSDPT GEIVRYLFSI ISMFIFAFLQ
       61 QITKGGLYNP LTALAAGVSG GFSSFIFSVF VRIPVEVIGS ILAVKHVIHV FPEIGKGPKL
      121 QVAIHHGALT EGILTFFIVL LSMGLTRKIP GSFFMKTWIG SLAKLTLHIL GSDLTGGCMN
      181 PAAVMGWAYA RGEHITKEHL LVYWLGPVKA TLLAVWFFKV VFKPLTEEQQ KPKAKTE
//
LOCUS       AlTIP1-1       251 aa
DEFINITION  AlTIP1-1     251 aa
TITLE       AlTIP1-1
ORIGIN

        1 MPIRNIAVGR PDEATRPDAL KAALAEFIST LIFVVAGSGS GMAFNKLTEN GATTPSGLVA
       61 AALAHAFGLF VAVSVGANIS GGHVNPAVTF GAFIGGNITL LRGILYWIAQ LLGSVVACLI
      121 LKFATGGLAV PAFGLSAGVG VLNAFVFEIV MTFGLVYTVY ATAIDPKNGS LGTIAPIAIG
      181 FIVGANILAG GAFSGASMNP AVAFGPAVVS WTWTNHWVYW AGPLVGGGIA GLIYEVFFIN
      241 TTHEQLPTTD Y
//
LOCUS       AlTIP1-2       253 aa
DEFINITION  AlTIP1-2     253 aa
TITLE       AlTIP1-2
ORIGIN

        1 MPTRNIAIGG VQEEVYHPNA LRAALAEFIS TLIFVFAGSG SGIAFNKLTD NGATTPSGLV
       61 AAALAHAFGL FVAVSVGANI SGGHVNPAVT FGVLLGGNIT LLRGILYWIA QLLGSVAACF
      121 LLSFATGGEP IPAFGLSAGV GSLNALVFEI VMTFGLVYTV YATAVDPKNG SLGTIAPIAI
      181 GFIVGANILA GGAFSGASMN PAVAFGPAIV SWTWTNHWVY WAGPLVGGGL AGIIYDFVFI
      241 DENAHEQLPT TDY
//
LOCUS       AlTIP1-3       252 aa
DEFINITION  AlTIP1-3     252 aa
TITLE       AlTIP1-3
ORIGIN

        1 MAINRIAIGT PGEASRPDAI RAAFAEFFSM VIFVFAGQGS GMAYGKLTGD GPATPSGLVA
       61 ASLSHAFALF VAVSVGANVS GGHVNPAVTF GAFIGGNITL LRAVLYWIAQ LLGAVVACLL
```

```
      121 LKVSTGGMET AAFSLSHGVT PWNAVVFEIV MTFGLVYTVY ATAVDPKKGD IGIVAPLAIG
      181 LIVGANILVG GAFDGASMNP AVSFGPAVVS WTWTNHWVYW VGPFIGAAIA ATVYDTIFIG
      241 SNGHEPLPSN DF
//
LOCUS       AlTIP2-1       251 aa
DEFINITION  AlTIP2-1     251 aa
TITLE       AlTIP2-1
ORIGIN

        1 MAGVAFGSFD DSFSLASLRA YLAEFISTLL FVFAGVGSAI AYGSKLTSDA ALDTPGLVAI
       61 AVCHGFALFV AVAIGANISG GHVNPAVTFG LAVGGQITVI TGVFYWIAQL LGSTAACFLL
      121 KYVTGGLAVP THSVGAGLGA LEGVVMEIII TFALVYTVYA TAADPKKGSL GTIAPLAIGL
      181 IVGANILAAG PFSGGSMNPA RSFGPAVAAG DFSGHWVYWV GPLIGGGLAG LIYGNVFMGS
      241 SEHAPLASAD F
//
LOCUS       AlTIP2-2       250 aa
DEFINITION  AlTIP2-2     250 aa
TITLE       AlTIP2-2
ORIGIN

        1 MVKIEIGSLG DSFSVASLKA YLSEFIATLL FVFAGVGSAL AFAKLTSDAA LDPAGLVAVA
       61 VAHAFALFVG VSIAANISGG HLNPAVTLGL AIGGNITVIT GFFYWIAQCL GSIVACLLLV
      121 FVTNGESVPT HGVAAGLGAI EGVVMEIVVT FALVYTVYAT AADPKKGSLG TIAPIAIGFI
      181 VGANILAAGP FSGGSMNPAR SFGPAVVSGD FSQIWIYWVG PLVGGALAGL IYGDVFIGSY
      241 APAPTTESYP
//
LOCUS       AlTIP2-3       355 aa
DEFINITION  AlTIP2-3     355 aa
TITLE       AlTIP2-3
ORIGIN

        1 MVKIEVGSVG DSFSVSSLKA YLSEFIATLL FIFAGVGSAI AFDKLTSDGA LDPAGLVAIA
       61 IAHAFVFSLE FPLRLIYLVV TLTQQHYRSE NIVFFCLGEL IIIRRVLMLT QLCKFLFLHL
      121 PTSTSYCLSF FINKCPVFIS SFTTTNSNYI IFFLVNMLIA NYNVFISSFM TTTRTRSKIL
      181 PVRKPYIISW LTLGLAIGGN ITLITGFFYW IAQCLGSIVA CLLLVFVTNG KSVPTHGVAA
      241 GLGAVEGIVM EIVVTFALVY TVYATAADPK KGSLGTIAPI AIGFIVGANI LAAGPFSGGS
      301 MNPARSFGPA VVSGDLSQIW IYWVEPLVGG ALAGLIYGDV FIGSYEAVET REIRV
//
LOCUS       AlTIP2-4       250 aa
DEFINITION  AlTIP2-4     250 aa
TITLE       AlTIP2-4
ORIGIN

        1 MVKIELGSVG DSFSVSSLKA YLSEFIATLL FVFAGVGSAI AFDKLTSDGA LDPAGLVAIA
       61 IAHAFALFVG VSIAANISGG HLNPAVTLGL AIGGNITLII GFFYWIAQCL GSIVACLLLV
      121 FVTNGKSVPT HGVAAGLGAV EGIVMEIVVT FALVYTVYAT AADPKKGSLG TIAPIAIGFI
      181 VGANILAAGP FSGGSMNPAR SFGPAVVSGD LSQIWIYWVG PLVGGALAGL IYGDVFIGSY
      241 EAVETREIRV
//
LOCUS       AlTIP2-5       248 aa
DEFINITION  AlTIP2-5     248 aa
TITLE       AlTIP2-5
ORIGIN
```

```
        1 MAGLDIIGSF EDSFKLVTLK AYLAEFISSL LFVFAGVGSA IAFGKLTENA ALDASGLVAV
       61 SIAANHSGGH VNPAVTFGLV VGGKVTIITG VFYWIAQLLG STVACFLLKF VTGGLVWRFQ
      121 STDHNVAAGV GTAQGVVMEI IITFALVYTV YATAIDSNNG TLGTIAPLAI GLIVGANILA
      181 AGPFSGGSMN PARSFGPALA VGDFSGHWVY WVGPLVGGGL AGVIYSNAFI ESKPEPTESE
      241 RVPLISSA
//
LOCUS       AlTIP3-1       268 aa
DEFINITION  AlTIP3-1     268 aa
TITLE       AlTIP3-1
ORIGIN

        1 MATSARRAYG FGRADEATHP DSIRATLAEF LSTFVFVFAA EGSILSLDKL YWDHAAHAGT
       61 NTPGGLILVA LAHAFALFAA VSAAINVSGG HVNPAVTFGA LIGGRLSAIR AIYYWVAQLL
      121 GAILACLLLR LATNGMRPVG FRVASGVGAV NGLVLEIILT FGLVYVVYST LIDPKRGSLG
      181 IIAPLAIGLI VGANILVGGP FSGASMNPAR AFGPALVGWR WHDHWIYWVG PFIGSALAAL
      241 IYEYMVIPTE PPTHHTHGVH QPLAPEDY
//
LOCUS       AlTIP3-2       267 aa
DEFINITION  AlTIP3-2     267 aa
TITLE       AlTIP3-2
ORIGIN

        1 MATSTRRAYG FGRADEATHP DSIRATLAEF LSTFVFVFAG EGSILALDKL YWDTAAHTGT
       61 NTPGGLVLVA LAHALALFAA VSAAINVSGG HVNPAVTFAA LIGGRISVIR AIYYWIAQLL
      121 GAIIACLLLR LATNGLRPIG FHVASGVSEL HGLVMEIILT FALVYVVYST AIDPKRGSIG
      181 IIAPLAIGLI VGANTLVGGP FDGASMNPAR AFGPALVGWR WNNHWIYWVG PFIGGALAAL
      241 IYEYMIIPSV NEPPHHSTHQ PLAPEDY
//
LOCUS       AlTIP4-1       249 aa
DEFINITION  AlTIP4-1     249 aa
TITLE       AlTIP4-1
ORIGIN

        1 MKKIELGHHS EAAKPGCIKA LIVEFITTFL FVFAGVGSAM ATDSLVGNTL VGLFAVAVAH
       61 ALVVAVMISA GHISGGHLNP AVTLGLLLGG HISVFRAFLY WIDQLLASSA ACFLLSYLTG
      121 GMATPVHTLA IGVSYTQGII WEIILTFSLL FTVYATMVDP KKGSLDGLGP LLTGFVVGAN
      181 ILAGGAFSGA SMNPARSFGP ALVSGNWTDH WVYWVGPLIG GGLAGFIYEN VLIDRPHVPV
      241 ADDEQPLLD
//
LOCUS       AlTIP5-1       256 aa
DEFINITION  AlTIP5-1     256 aa
TITLE       AlTIP5-1
ORIGIN

        1 MRTMIPTSFS SRFQGVISMN ALRCYVSEFI STFFFVLAAV GSVMSSRKLM AGDVSGPFGV
       61 LIPAIANAFA LSSSVYISWN VSGGHVNPAV TFGMAVAGRI SVPTAMFYWT SQMIASVMAC
      121 LVLKVTVVEQ HVPIYKIAGE MTGFGASVLE GVLAFVLVYT VFTANDPRRG LPLAVGPIFI
      181 GFVAGANVLA AGPFSGGSMN PACAFGSAMV YGSFKNQAVY WVGPLLGGAT AALVYDNVVV
      241 PAEDDRGSST GDAIGV
//
LOCUS       AtNIP1-1       296 aa
DEFINITION  AtNIP1-1     296 aa
TITLE       AtNIP1-1
ORIGIN
```

```
        1 MADISGNGYG NAREEVVMVN LKDEVEHQQE MEDIHNPRPL KKQDSLLSVS VPFLQKLIAE
       61 FLGTYFLVFT GCASVVVNMQ NDNVVTLPGI AIVWGLTIMV LIYSLGHISG AHINPAVTIA
      121 FASCGRFPLK QVPAYVISQV IGSTLAAATL RLLFGLDHDV CSGKHDVFIG SSPVGSDLQA
      181 FTMEFIVTFY LMFIISGVAT DNRAIGELAG LAIGSTVLLN VLIAAPVSSA SMNPGRSLGP
      241 ALVYGCYKGI WIYLVAPTLG AIAGAWVYNT VRYTDKPLRE ITKSGSFLKT VRIGST
//
LOCUS       AtNIP1-2       294 aa
DEFINITION  AtNIP1-2     294 aa
TITLE       AtNIP1-2
ORIGIN

        1 MAEISGNGGD ARDGAVVVNL KEEDEQQQQQ QAIHKPLKKQ DSLLSISVPF LQKLMAEVLG
       61 TYFLIFAGCA AVAVNTQHDK AVTLPGIAIV WGLTVMVLVY SLGHISGAHF NPAVTIAFAS
      121 CGRFPLKQVP AYVISQVIGS TLAAATLRLL FGLDQDVCSG KHDVFVGTLP SGSNLQSFVI
      181 EFIITFYLMF VISGVATDNR AIGELAGLAV GSTVLLNVII AGPVSGASMN PGRSLGPAMV
      241 YSCYRGLWIY IVSPIVGAVS GAWVYNMVRY TDKPLREITK SGSFLKTVRN GSSR
//
LOCUS       AtNIP2-1       288 aa
DEFINITION  AtNIP2-1     288 aa
TITLE       AtNIP2-1
ORIGIN

        1 MDDISVSKSN HGNVVVLNIK ASSLADTSLP SNKHESSSPP LLSVHFLQKL LAELVGTYYL
       61 IFAGCAAIAV NAQHNHVVTL VGIAVVWGIV IMVLVYCLGH LSAHFNPAVT LALASSQRFP
      121 LNQVPAYITV QVIGSTLASA TLRLLFDLNN DVCSKKHDVF LGSSPSGSDL QAFVMEFIIT
      181 GFLMLVVCAV TTTKRTTEEL EGLIIGATVT LNVIFAGEVS GASMNPARSI GPALVWGCYK
      241 GIWIYLLAPT LGAVSGALIH KMLPSIQNAE PEFSKTGSSH KRVTDLPL
//
LOCUS       AtNIP3-1       323 aa
DEFINITION  AtNIP3-1     323 aa
TITLE       AtNIP3-1
ORIGIN

        1 MAEISDITTQ TQTVVLDIEN YQSIDDSRSS DLSAPLVSVS FVQKLIGEFV GTFTMIFAGC
       61 SAIVVNETYG KPVTLPGIAL VWGLVVTVMI YSIGHVSGAH FNPAVSIAFA SSKKFPFNQV
      121 PGYIAAQLLG STLAAAVLRL VFHLDDDVCS LKGDVYVGTY PSNSNTTSFV MEFIATFNLM
      181 FVISAVATDK RATGSFAGIA IGATIVLDIL FSGPISGASM NPARSLGPAL IWGCYKDLWL
      241 YIVSPVIGAL SGAWTYGLLR STKKSYSEII RPNCNKVSSR DRQEASQDEI CVLRVVDPAN
      301 QNYFICSSPT DINGKCNVTC KLA
//
LOCUS       AtNIP4-1       283 aa
DEFINITION  AtNIP4-1     283 aa
TITLE       AtNIP4-1
ORIGIN

        1 MSSHSDEIEE EQISRIEKGK GKDCQGGIET VICTSPSIVC LTQKLIAEMI GTYFIVFSGC
       61 GVVVVNVLYG GTITFPGICV TWGLIVMVMI YSTGHISGAH FNPAVTVTFA IFRRFPWHQV
      121 PLYIGAQFAG SLLASLTLRL MFKVTPEAFF GTTPADSPAR ALVAEIIISF LLMFVISGVA
      181 TDNRAVGELA GIAVGMTIMV NVFVAGPISG ASMNPARSLG PALVMGVYKH IWVYIVGPVL
      241 GVISGGFVYN LIRFTDKPLR ELTKSASFLR AVSPSHKGSS SKT
//
LOCUS       AtNIP4-2       283 aa
DEFINITION  AtNIP4-2     283 aa
```

```
TITLE       AtNIP4-2
ORIGIN

        1 MTSHGEEIED EQISRIEKGN CKDSQGGMET AICSSPSIVC LTQKLIAEMI GTYFIIFSGC
       61 GVVVVNVLYG GTITFPGICV TWGLIVMVMI YSTGHISGAH FNPAVTVTFA VFRRFPWYQV
      121 PLYIGAQLTG SLLASLTLRL MFNVTPKAFF GTTPTDSSGQ ALVAEIIISF LLMFVISGVA
      181 TDSRATGELA GIAVGMTIIL NVFVAGPISG ASMNPARSLG PAIVMGRYKG IWVYIVGPFV
      241 GIFAGGFVYN FMRFTDKPLR ELTKSASFLR SVAQKDNASK SDG
//
LOCUS       AtNIP5-1       304 aa
DEFINITION  AtNIP5-1     304 aa
TITLE       AtNIP5-1
ORIGIN

        1 MAPPEAEVGA VMVMAPPTPG TPGTPGGPLI TGMRVDSMSF DHRKPTPRCK CLPVMGSTWG
       61 QHDTCFTDFP SPDVSLTRKL GAEFVGTFIL IFTATAGPIV NQKYDGAETL IGNAACAGLA
      121 VMIIILSTGH ISGAHLNPSL TIAFAALRHF PWAHVPAYIA AQVSASICAS FALKGVFHPF
      181 MSGGVTIPSV SLGQAFALEF IITFILLFVV TAVATDTRAV GELAGIAVGA TVMLNILVAG
      241 PSTGGSMNPV RTLGPAVASG NYRSLWVYLV APTLGAISGA AVYTGVKLND SVTDPPRPVR
      301 SFRR
//
LOCUS       AtNIP6-1       305 aa
DEFINITION  AtNIP6-1     305 aa
TITLE       AtNIP6-1
ORIGIN

        1 MDHEEIPSTP STPATTPGTP GAPLFGGFEG KRNGHNGRYT PKSLLKSCKC FSVDNEWALE
       61 DGRLPPVTCS LPPPNVSLYR KLGAEFVGTL ILIFAGTATA IVNQKTDGAE TLIGCAASAG
      121 LAVMIVILST GHISGAHLNP AVTIAFAALK HFPWKHVPVY IGAQVMASVS AAFALKAVFE
      181 PTMSGGVTVP TVGLSQAFAL EFIISFNLMF VVTAVATDTR AVGELAGIAV GATVMLNILI
      241 AGPATSASMN PVRTLGPAIA ANNYRAIWVY LTAPILGALI GAGTYTIVKL PEEDEAPKER
      301 RSFRR
//
LOCUS       AtNIP7-1       275 aa
DEFINITION  AtNIP7-1     275 aa
TITLE       AtNIP7-1
ORIGIN

        1 MNGEARSRVV DQEAGSTPST LRDEDHPSRQ RLFGCLPYDI DLNPLRIVMA ELVGTFILMF
       61 SVCGVISSTQ LSGGHVGLLE YAVTAGLSVV VVVYSIGHIS GAHLNPSITI AFAVFGGFPW
      121 SQVPLYITAQ TLGATAATLV GVSVYGVNAD IMATKPALSC VSAFFVELIA TSIVVFLASA
      181 LHCGPHQNLG NLTGFVIGTV ISLGVLITGP ISGGSMNPAR SLGPAVVAWD FEDLWIYMTA
      241 PVIGAIIGVL TYRSISLKTR PCPSPVSPSV SSLLR
//
LOCUS       AtPIP1-1       286 aa
DEFINITION  AtPIP1-1     286 aa
TITLE       AtPIP1-1
ORIGIN

        1 MEGKEEDVRV GANKFPERQP IGTSAQSDKD YKEPPPAPFF EPGELSSWSF WRAGIAEFIA
       61 TFLFLYITVL TVMGVKRSPN MCASVGIQGI AWAFGGMIFA LVYCTAGISG GHINPAVTFG
      121 LFLARKLSLT RALYYIVMQC LGAICGAGVV KGFQPKQYQA LGGGANTVAH GYTKGSGLGA
      181 EIIGTFVLVY TVFSATDAKR NARDSHVPIL APLPIGFAVF LVHLATIPIT GTGINPARSL
      241 GAAIIYNKDH SWDDHWVFWV GPFIGAALAA LYHVVVIRAI PFKSRS
```

```
//
LOCUS       AtPIP1-2       286 aa
DEFINITION  AtPIP1-2     286 aa
TITLE       AtPIP1-2
ORIGIN

        1 MEGKEEDVRV GANKFPERQP IGTSAQSDKD YKEPPPAPLF EPGELASWSF WRAGIAEFIA
       61 TFLFLYITVL TVMGVKRSPN MCASVGIQGI AWAFGGMIFA LVYCTAGISG GHINPAVTFG
      121 LFLARKLSLT RAVYYIVMQC LGAICGAGVV KGFQPKQYQA LGGGANTIAH GYTKGSGLGA
      181 EIIGTFVLVY TVFSATDAKR NARDSHVPIL APLPIGFAVF LVHLATIPIT GTGINPARSL
      241 GAAIIFNKDN AWDDHWVFWV GPFIGAALAA LYHVIVIRAI PFKSRS
//
LOCUS       AtPIP1-3       286 aa
DEFINITION  AtPIP1-3     286 aa
TITLE       AtPIP1-3
ORIGIN

        1 MEGKEEDVRV GANKFPERQP IGTSAQTDKD YKEPPPAPFF EPGELSSWSF YRAGIAEFIA
       61 TFLFLYITVL TVMGVKRAPN MCASVGIQGI AWAFGGMIFA LVYCTAGISG GHINPAVTFG
      121 LFLARKLSLT RAVFYIVMQC LGAICGAGVV KGFQPNPYQT LGGGANTVAH GYTKGSGLGA
      181 EIIGTFVLVY TVFSATDAKR SARDSHVPIL APLPIGFAVF LVHLATIPIT GTGINPARSL
      241 GAAIIYNKDH AWDDHWIFWV GPFIGAALAA LYHQLVIRAI PFKSRS
//
LOCUS       AtPIP1-4       287 aa
DEFINITION  AtPIP1-4     287 aa
TITLE       AtPIP1-4
ORIGIN

        1 MEGKEEDVRV GANKFPERQP IGTSAQSTDK DYKEPPPAPL FEPGELSSWS FYRAGIAEFI
       61 ATFLFLYITV LTVMGVKRAP NMCASVGIQG IAWAFGGMIF ALVYCTAGIS GGHINPAVTF
      121 GLFLARKLSL TRAVFYMIMQ CLGAICGAGV VKGFQPTPYQ TLGGGANTVA HGYTKGSGLG
      181 AEIIGTFVLV YTVFSATDAK RSARDSHVPI LAPLPIGFAV FLVHLATIPI TGTGINPARS
      241 LGAAIIYNKD HSWDDHWIFW VGPFIGAALA ALYHQIVIRA IPFKSKS
//
LOCUS       AtPIP1-5       287 aa
DEFINITION  AtPIP1-5     287 aa
TITLE       AtPIP1-5
ORIGIN

        1 MEGKEEDVNV GANKFPERQP IGTAAQTESK DYKEPPPAPF FEPGELKSWS FYRAGIAEFI
       61 ATFLFLYVTV LTVMGVKRAP NMCASVGIQG IAWAFGGMIF ALVYCTAGIS GGHINPAVTF
      121 GLFLARKLSL TRALFYIVMQ CLGAICGAGV VKGFQPGLYQ TNGGGANVVA HGYTKGSGLG
      181 AEIVGTFVLV YTVFSATDAK RSARDSHVPI LAPLPIGFAV FLVHLATIPI TGTGINPARS
      241 LGAAIIYNKD HAWDDHWIFW VGPFIGAALA ALYHQIVIRA IPFKSKT
//
LOCUS       AtPIP2-1       287 aa
DEFINITION  AtPIP2-1     287 aa
TITLE       AtPIP2-1
ORIGIN

        1 MAKDVEAVPG EGFQTRDYQD PPPAPFIDGA ELKKWSFYRA VIAEFVATLL FLYITVLTVI
       61 GYKIQSDTDA GGVDCGGVGI LGIAWAFGGM IFILVYCTAG ISGGHINPAV TFGLFLARKV
      121 SLPRALLYII AQCLGAICGV GFVKAFQSSY YTRYGGGANS LADGYSTGTG LAAEIIGTFV
      181 LVYTVFSATD PKRSARDSHV PVLAPLPIGF AVFMVHLATI PITGTGINPA RSFGAAVIYN
```

```
      241 KSKPWDDHWI FWVGPFIGAA IAAFYHQFVL RASGSKSLGS FRSAANV
//
LOCUS       AtPIP2-2       285 aa
DEFINITION  AtPIP2-2     285 aa
TITLE       AtPIP2-2
ORIGIN

        1 MAKDVEGPEG FQTRDYEDPP PTPFFDADEL TKWSLYRAVI AEFVATLLFL YITVLTVIGY
       61 KIQSDTKAGG VDCGGVGILG IAWAFGGMIF ILVYCTAGIS GGHINPAVTF GLFLARKVSL
      121 IRAVLYMVAQ CLGAICGVGF VKAFQSSYYD RYGGGANSLA DGYNTGTGLA AEIIGTFVLV
      181 YTVFSATDPK RNARDSHVPV LAPLPIGFAV FMVHLATIPI TGTGINPARS FGAAVIYNKS
      241 KPWDDHWIFW VGPFIGAAIA AFYHQFVLRA SGSKSLGSFR SAANV
//
LOCUS       AtPIP2-3       285 aa
DEFINITION  AtPIP2-3     285 aa
TITLE       AtPIP2-3
ORIGIN

        1 MAKDVEGPDG FQTRDYEDPP PTPFFDAEEL TKWSLYRAVI AEFVATLLFL YVTVLTVIGY
       61 KIQSDTKAGG VDCGGVGILG IAWAFGGMIF ILVYCTAGIS GGHINPAVTF GLFLARKVSL
      121 IRAVLYMVAQ CLGAICGVGF VKAFQSSHYV NYGGGANFLA DGYNTGTGLA AEIIGTFVLV
      181 YTVFSATDPK RNARDSHVPV LAPLPIGFAV FMVHLATIPI TGTGINPARS FGAAVIFNKS
      241 KPWDDHWIFW VGPFIGATIA AFYHQFVLRA SGSKSLGSFR SAANV
//
LOCUS       AtPIP2-4       291 aa
DEFINITION  AtPIP2-4     291 aa
TITLE       AtPIP2-4
ORIGIN

        1 MAKDLDVNES GPPAARDYKD PPPAPFFDME ELRKWPLYRA VIAEFVATLL FLYVSILTVI
       61 GYKAQTDATA GGVDCGGVGI LGIAWAFGGM IFVLVYCTAG ISGGHINPAV TVGLFLARKV
      121 SLVRTVLYIV AQCLGAICGC GFVKAFQSSY YTRYGGGANE LADGYNKGTG LGAEIIGTFV
      181 LVYTVFSATD PKRNARDSHV PVLAPLPIGF AVFMVHLATI PITGTGINPA RSFGAAVIYN
      241 NEKAWDDQWI FWVGPMIGAA AAAFYHQFIL RAAAIKALGS FGSFGSFRSF A
//
LOCUS       AtPIP2-5       286 aa
DEFINITION  AtPIP2-5     286 aa
TITLE       AtPIP2-5
ORIGIN

        1 MTKEVVGDKR SFSGKDYQDP PPEPLFDATE LGKWSFYRAL IAEFIATLLF LYVTIMTVIG
       61 YKSQTDPALN PDQCTGVGVL GIAWAFGGMI FILVYCTAGI SGGHINPAVT FGLLLARKVT
      121 LVRAVMYMVA QCLGAICGVA LVKAFQSAYF TRYGGGANGL SDGYSIGTGV AAEIIGTFVL
      181 VYTVFSATDP KRSARDSHVP VLAPLPIGFA VFIVHLATIP ITGTGINPAR SLGAAIIYNK
      241 DKAWDHHWIF WVGPFAGAAI AAFYHQFVLR AGAIKALGSF RSQPHV
//
LOCUS       AtPIP2-6       289 aa
DEFINITION  AtPIP2-6     289 aa
TITLE       AtPIP2-6
ORIGIN

        1 MTKDELTEEE SLSGKDYLDP PPVKTFEVRE LKKWSFYRAV IAEFIATLLF LYVTVLTVIG
       61 FKSQTDINAG GGACASVGLL GISWAFGGMI FILVYCTAGI SGGHINPAVT FGLFLASKVS
      121 LVRAVSYMVA QCLGATCGVG LVKVFQSTYY NRYGGGANML SDGYNVGVGV GAEIIGTFVL
```

```
      181 VYTVFSATDP KRNARDSHIP VLAPLPIGFS VFMVHLATIP ITGTGINPAR SFGAAVIYNN
      241 QKAWDDQWIF WVGPFVGAAI AAFYHQFVLR AGAMKAYGSV RSQLHELHA
//
LOCUS       AtPIP2-7       280 aa
DEFINITION  AtPIP2-7     280 aa
TITLE       AtPIP2-7
ORIGIN

        1 MSKEVSEEGK THHGKDYVDP PPAPLLDMGE LKSWSFYRAL IAEFIATLLF LYVTVATVIG
       61 HKKQTGPCDG VGLLGIAWAF GGMIFVLVYC TAGISGGHIN PAVTFGLFLA RKVSLVRALG
      121 YMIAQCLGAI CGVGFVKAFM KTPYNTLGGG ANTVADGYSK GTALGAEIIG TFVLVYTVFS
      181 ATDPKRSARD SHIPVLAPLP IGFAVFMVHL ATIPITGTGI NPARSFGAAV IYNNEKAWDD
      241 QWIFWVGPFL GALAAAAYHQ YILRASAIKA LGSFRSNATN
//
LOCUS       AtPIP2-8       278 aa
DEFINITION  AtPIP2-8     278 aa
TITLE       AtPIP2-8
ORIGIN

        1 MSKEVSEEGR HGKDYVDPPP APLLDMAELK LWSFYRAIIA EFIATLLFLY VTVATVIGHK
       61 NQTGPCGGVG LLGIAWAFGG MIFVLVYCTA GISGGHINPA VTFGLFLARK VSLPRAVAYM
      121 VAQCLGAICG VGLVKAFMMT PYKRLGGGAN TVADGYSTGT ALGAEIIGTF VLVYTVFSAT
      181 DPKRSARDSH VPVLAPLPIG FAVFMVHLAT IPITGTGINP ARSFGAAVIY NNEKAWDDHW
      241 IFWVGPFVGA LAAAAYHQYI LRAAAIKALA SFRSNPTN
//
LOCUS       AtSIP1-1       240 aa
DEFINITION  AtSIP1-1     240 aa
TITLE       AtSIP1-1
ORIGIN

        1 MMGVLKSAIG DMLMTFSWVV LSATFGIQTA AIISAGDFQA ITWAPLVILT SLIFVYVSIF
       61 TVIFGSASFN PTGSAAFYVA GVPGDTLFSL AIRLPAQAIG AAGGALAIME FIPEKYKHMI
      121 GGPSLQVDVH TGAIAETILS FGITFAVLLI ILRGPRRLLA KTFLLALATI SFVVAGSKYT
      181 GPAMNPAIAF GWAYMYSSHN TWDHIYVYWI SSFVGALSAA LLFRSIFPPP RPQKKKQKKA
//
LOCUS       AtSIP1-2       243 aa
DEFINITION  AtSIP1-2     243 aa
TITLE       AtSIP1-2
ORIGIN

        1 MSAVKSALGD MVITFLWVIL SATFGIQTAA IVSAVGFHGI TWAPLVISTL VVFVSISIFT
       61 VIGNVLGGAS FNPCGNAAFY TAGVSSDSLF SLAIRSPAQA IGAAGGAITI MEMIPEKYKT
      121 RIGGKPSLQF GAHNGAISEV VLSFSVTFLV LLIIILRGPRK LLAKTFLLAL ATVSVFVVGS
      181 KFTRPFMNPA IAFGWAYIYK SHNTWDHFYV YWISSYTGAI LSAMLFRIIF PAPPLVQKKQ
      241 KKA
//
LOCUS       AtSIP2-1       260 aa
DEFINITION  AtSIP2-1     260 aa
TITLE       AtSIP2-1
ORIGIN

        1 MGRIGLVVTD LVLSFMWIWA GVLVNILVHG VLGFSRTDPS GEIVRYLFSI ISMFIFAYLQ
       61 QATKGGLYNP LTALAAGVSG GFSSFIFSVF VRIPVEVIGS ILAVKHIIHV FPEIGKGPKL
      121 NVAIHHGALT EGILTFFIVL LSMGLTRKIP GSFFMKTWIG SLAKLTLHIL GSDLTGGCMN
```

```
      181 PAAVMGWAYA RGEHITKEHL LVYWLGPVKA TLLAVWFFKV VFKPLTEEQE KPKAKSEFCN
      241 VFFFYVANST VSTSSIKSEI
//
LOCUS       AtTIP1-1       251 aa
DEFINITION  AtTIP1-1     251 aa
TITLE       AtTIP1-1
ORIGIN

        1 MPIRNIAIGR PDEATRPDAL KAALAEFIST LIFVVAGSGS GMAFNKLTEN GATTPSGLVA
       61 AAVAHAFGLF VAVSVGANIS GGHVNPAVTF GAFIGGNITL LRGILYWIAQ LLGSVVACLI
      121 LKFATGGLAV PAFGLSAGVG VLNAFVFEIV MTFGLVYTVY ATAIDPKNGS LGTIAPIAIG
      181 FIVGANILAG GAFSGASMNP AVAFGPAVVS WTWTNHWVYW AGPLVGGGIA GLIYEVFFIN
      241 TTHEQLPTTD Y
//
LOCUS       AtTIP1-2       253 aa
DEFINITION  AtTIP1-2     253 aa
TITLE       AtTIP1-2
ORIGIN

        1 MPTRNIAIGG VQEEVYHPNA LRAALAEFIS TLIFVFAGSG SGIAFNKITD NGATTPSGLV
       61 AAALAHAFGL FVAVSVGANI SGGHVNPAVT FGVLLGGNIT LLRGILYWIA QLLGSVAACF
      121 LLSFATGGEP IPAFGLSAGV GSLNALVFEI VMTFGLVYTV YATAVDPKNG SLGTIAPIAI
      181 GFIVGANILA GGAFSGASMN PAVAFGPAVV SWTWTNHWVY WAGPLIGGGL AGIIYDFVFI
      241 DENAHEQLPT TDY
//
LOCUS       AtTIP1-3       252 aa
DEFINITION  AtTIP1-3     252 aa
TITLE       AtTIP1-3
ORIGIN

        1 MPINRIAIGT PGEASRPDAI RAAFAEFFSM VIFVFAGQGS GMAYGKLTGD GPATPAGLVA
       61 ASLSHAFALF VAVSVGANVS GGHVNPAVTF GAFIGGNITL LRAILYWIAQ LLGAVVACLL
      121 LKVSTGGMET AAFSLSYGVT PWNAVVFEIV MTFGLVYTVY ATAVDPKKGD IGIIAPLAIG
      181 LIVGANILVG GAFDGASMNP AVSFGPAVVS WIWTNHWVYW VGPFIGAAIA AIVYDTIFIG
      241 SNGHEPLPSN DF
//
LOCUS       AtTIP2-1       250 aa
DEFINITION  AtTIP2-1     250 aa
TITLE       AtTIP2-1
ORIGIN

        1 MAGVAFGSFD DSFSLASLRA YLAEFISTLL FVFAGVGSAI AYAKLTSDAA LDTPGLVAIA
       61 VCHGFALFVA VAIGANISGG HVNPAVTFGL AVGGQITVIT GVFYWIAQLL GSTAACFLLK
      121 YVTGGLAVPT HSVAAGLGSI EGVVMEIIIT FALVYTVYAT AADPKKGSLG TIAPLAIGLI
      181 VGANILAAGP FSGGSMNPAR SFGPAVAAGD FSGHWVYWVG PLIGGGLAGL IYGNVFMGSS
      241 EHVPLASADF
//
LOCUS       AtTIP2-2       250 aa
DEFINITION  AtTIP2-2     250 aa
TITLE       AtTIP2-2
ORIGIN

        1 MVKIEIGSVG DSFSVASLKA YLSEFIATLL FVFAGVGSAL AFAKLTSDAA LDPAGLVAVA
       61 VAHAFALFVG VSIAANISGG HLNPAVTLGL AVGGNITVIT GFFYWIAQCL GSIVACLLLV
```

```
      121 FVTNGESVPT HGVAAGLGAI EGVVMEIVVT FALVYTVYAT AADPKKGSLG TIAPIAIGFI
      181 VGANILAAGP FSGGSMNPAR SFGPAVVSGD FSQIWIYWVG PLVGGALAGL IYGDVFIGSY
      241 APAPTTESYP
//
LOCUS       AtTIP2-3       250 aa
DEFINITION  AtTIP2-3     250 aa
TITLE       AtTIP2-3
ORIGIN

        1 MVKIEVGSVG DSFSVSSLKA YLSEFIATLL FVFAGVGSAV AFAKLTSDGA LDPAGLVAIA
       61 IAHAFALFVG VSIAANISGG HLNPAVTLGL AIGGNITLIT GFFYWIAQCL GSIVACLLLV
      121 FVTNGKSVPT HGVSAGLGAV EGVVMEIVVT FALVYTVYAT AADPKKGSLG TIAPIAIGFI
      181 VGANILAAGP FSGGSMNPAR SFGPAVVSGD LSQIWIYWVG PLVGGALAGL IYGDVFIGSY
      241 EAVETREIRV
//
LOCUS       AtTIP3-1       268 aa
DEFINITION  AtTIP3-1     268 aa
TITLE       AtTIP3-1
ORIGIN

        1 MATSARRAYG FGRADEATHP DSIRATLAEF LSTFVFVFAA EGSILSLDKL YWEHAAHAGT
       61 NTPGGLILVA LAHAFALFAA VSAAINVSGG HVNPAVTFGA LVGGRVTAIR AIYYWIAQLL
      121 GAILACLLLR LTTNGMRPVG FRLASGVGAV NGLVLEIILT FGLVYVVYST LIDPKRGSLG
      181 IIAPLAIGLI VGANILVGGP FSGASMNPAR AFGPALVGWR WHDHWIYWVG PFIGSALAAL
      241 IYEYMVIPTE PPTHHAHGVH QPLAPEDY
//
LOCUS       AtTIP3-2       267 aa
DEFINITION  AtTIP3-2     267 aa
TITLE       AtTIP3-2
ORIGIN

        1 MATSARRAYG FGRADEATHP DSIRATLAEF LSTFVFVFAG EGSILALDKL YWDTAAHTGT
       61 NTPGGLVLVA LAHALALFAA VSAAINVSGG HVNPAVTFAA LIGGRISVIR AIYYWVAQLI
      121 GAILACLLLR LATNGLRPVG FHVASGVSEL HGLLMEIILT FALVYVVYST AIDPKRGSIG
      181 IIAPLAIGLI VGANILVGGP FDGASMNPAR AFGPALVGWR WSNHWIYWVG PFIGGALAAL
      241 IYEYMIIPSV NEPPHHSTHQ PLAPEDY
//
LOCUS       AtTIP4-1       249 aa
DEFINITION  AtTIP4-1     249 aa
TITLE       AtTIP4-1
ORIGIN

        1 MKKIELGHHS EAAKPDCIKA LIVEFITTFL FVFAGVGSAM ATDSLVGNTL VGLFAVAVAH
       61 AFVVAVMISA GHISGGHLNP AVTLGLLLGG HISVFRAFLY WIDQLLASSA ACFLLSYLTG
      121 GMGTPVHTLA SGVSYTQGII WEIILTFSLL FTVYATIVDP KKGSLDGFGP LLTGFVVGAN
      181 ILAGGAFSGA SMNPARSFGP ALVSGNWTDH WVYWVGPLIG GGLAGFIYEN VLIDRPHVPV
      241 ADDEQPLLN
//
LOCUS       AtTIP5-1       256 aa
DEFINITION  AtTIP5-1     256 aa
TITLE       AtTIP5-1
ORIGIN

        1 MRRMIPTSFS SKFQGVLSMN ALRCYVSEFI STFFFVLAAV GSVMSSRKLM AGDVSGPFGV
```

```
       61 LIPAIANALA LSSSVYISWN VSGGHVNPAV TFAMAVAGRI SVPTAMFYWT SQMIASVMAC
      121 LVLKVTVMEQ HVPIYKIAGE MTGFGASVLE GVLAFVLVYT VFTASDPRRG LPLAVGPIFI
      181 GFVAGANVLA AGPFSGGSMN PACAFGSAMV YGSFKNQAVY WVGPLLGGAT AALVYDNVVV
      241 PVEDDRGSST GDAIGV
//
LOCUS       BdNIP1-1       280 aa
DEFINITION  BdNIP1-1     280 aa
TITLE       BdNIP1-1
ORIGIN

        1 MAGGGDNAQT NGAAARDQAA MEEGRKDDYD QGCGLAISVP FVQKIIAEIF GTYFLIFAGC
       61 GAVTINASRN GQITFPGVAI VWGLAVMVMI YAVGHISGAH FNPAVTFAFA TVGRFPWRQV
      121 PAYVLAQMLG ATLASGTLRL MFGGRHEHFP GTLPGGSEVQ SLVLEFIITF YLMFVISGVA
      181 TDNRAIGELA GLAVGATILL NVLIAGPISG ASMNPARTVG PALVGSEYRS IWVYVVGPVA
      241 GAVAGAWSYN LIRFTNKPLR EITKSTSFLR SMSRMNSVAA
//
LOCUS       BdNIP1-2       282 aa
DEFINITION  BdNIP1-2     282 aa
TITLE       BdNIP1-2
ORIGIN

        1 MPGGEHGGSN GLQEHAGALE EGRGGGGNEA EDPEKSPNSS GKHPMLSVQF VQKILAEIFG
       61 TYFLIFAGCA AVAVNQRTAG TVTFPGICIT WGLAVMVMVY SVGHISGAHF NPAVTFAFAT
      121 CGRFPWKQVP AYAAAQLIGS TAAGLTLRLL FGREHFVGTV PAGSDVQSLV LEFIITFYLM
      181 FVVSGVATDN RAIGELAGLA VGATVLLNVL FAGPISGASM NPARTLGPAM VAGRYKGIWV
      241 YIVGPVGGAV AGAWAYNLIR FTNKPLREIT RTGSFLRSAR MG
//
LOCUS       BdNIP1-3       282 aa
DEFINITION  BdNIP1-3     282 aa
TITLE       BdNIP1-3
ORIGIN

        1 MARREDDSYT NGSVSMNDFS VEDGRKEKEV YDHDEPEQDG LCGMPVSVPF LQMLLAEFFS
       61 TYFLLFAGMG AIVVNNEKDG ALTFPGITMV WGLAVMVMIY TVGHISGAHM NPAVSLGFAI
      121 AGRMPWKRVP AYMLVQVFAA IIVSVVLRLM FGGRHEFVPV TAPTGSNIQS LVTEFTTTFY
      181 LVFVVMAVAT DDRAVGSMAG VAVGATITLN ALFSGPVTGA SMNPARSIGP ALVGGKYTSL
      241 WVYILGPFAG GAAGAWAYNL MRYTDKPAAV LSDVAKSTDR AA
//
LOCUS       BdNIP2-1       296 aa
DEFINITION  BdNIP2-1     296 aa
TITLE       BdNIP2-1
ORIGIN

        1 MSTNSRSNSR ANFSNEIHDM ATPQNSNMPN MMYYNERSLA DFFPPHLLKK MVSEVVSTFL
       61 LVFVTCGASA INGNDPSRIS QLGQSVAGGL IVTVMIYSVG HISGAHMNPA VTTAFAVFRH
      121 FPWIQVPFYW ASQFTGAICA SFVLKAVLHP IEVLGTTTPV GPHWHSLLIE IIVTFNMMFV
      181 TLAVATDTRA VGELAGLAVG SSVCITSIFA GAVSGGSMNP ARTLGPALAS NRYTGLWLYF
      241 LGPILGTLSG AWTYTFIRFE DSPKDAPQKL SSFKLRRLQS QSVAAEDDDV LDHIPV
//
LOCUS       BdNIP2-2       302 aa
DEFINITION  BdNIP2-2     302 aa
TITLE       BdNIP2-2
ORIGIN
```

```
        1 MAASGTGTPT RANSRVNYSN EIHDLSTVQD GAPSLAPSMY YQEKSFADFF PPHLLKKVIS
       61 EVVATFLLVF VTCGAASIYG ADVTRVSQLG QSLVGGLIVT VMIYATGHIS GAHMNPAVTL
      121 SFACFRHFPW IQVPFYWAAQ FTGAMCAAFV LRAVLHPITV LGTTTPTGPH WHALVIEIVV
      181 TFNMMFVTCA VATDSRAVGE LAGLAVGAAV CITSIFAGPV SGGSMNPART LAPAVASGVY
      241 SGLWIYFLGP VIGTLSGAWV YTYIRFEEAP SVKDGPQKLS SFKLRRLQSQ RSMANVDEFD
      301 HV
//
LOCUS       BdNIP3-1       178 aa
DEFINITION  BdNIP3-1     178 aa
TITLE       BdNIP3-1
ORIGIN

        1 MSKHGSCAGA TDQEGDSRVP RDVHPDVHPS VRHHHGRAAR RRGGPHGLMG IAVSVGLAVT
       61 VLVFSTIHIS GCHLNPAVSI AMAVFSHLPP AHLVPYIAAQ VLGSTAASFV GNAIYHPVNP
      121 GIATVPRVGT AEAFAIKFIT TFVLLFVITA VATDPHAVKE LIAVAVGATV VMNILIAG
//
LOCUS       BdNIP3-2       187 aa
DEFINITION  BdNIP3-2     187 aa
TITLE       BdNIP3-2
ORIGIN

        1 MGSTMIVPME PMNHEEDANI VTHRFFGRRP KIPANMAAVP LLKKVMAEFL GTFILMFTQV
       61 SSIMIMDEVQ GLMGIAVSVG LAVTVLVISL VHISGCHMNP AVSITMAVFG HLPPAHLVPY
      121 MAAQVLGSTA ASFFVCKVIH HRVHPGIATV PGVGVGAAEA FFVEFIVTFI LLFVITAVAT
      181 DPHAVGA
//
LOCUS       BdNIP3-3       301 aa
DEFINITION  BdNIP3-3     301 aa
TITLE       BdNIP3-3
ORIGIN

        1 MEGAATPNMS APATPGTPAP LFPGARVDSM SYERKSSMSV PRCRCLPVEA WMSSQHACVL
       61 EIPAPDVSLT RKLGAEFVGT FILIFFATAA PIVNQKYNNA ISPFGNAACA GLAVTTIILS
      121 TGHISGAHLN PSLTIAFAAL RHFPWLQVPA YVAVQSLASV CAGFALKGVF HPFLSGGVTV
      181 PDAAVSTAQA FFTEFIITFN LLFVVTAVAT DTRAVGELAG IAVGAAVTLN ILIAGPTTGG
      241 SMNPVRTLGP AVAAGNYRQL WIYLVAPTLG AVAGAGVYTA VKLRDVDGGG EAPRPQRSFR
      301 R
//
LOCUS       BdNIP4-1       285 aa
DEFINITION  BdNIP4-1     285 aa
TITLE       BdNIP4-1
ORIGIN

        1 MPTTVDLVRK DILVGVGGDD RAAVAANGHD LEQQARRDAA ADHGSKRLAI GRLIKELVME
       61 GVATFVVIFW SCTAALLQGT HHSLSFPMVC LVVALTVALV LGWIGPAHLN PAVTLTFAAF
      121 RYFPWRKLPL YVMVQLAASV LACLAVNALM RPRHGDFYGT VPMAGQGRRL PFVFEFLGSA
      181 VLMIVIATAA RAQRKVVGGV AIGAAVGTLG LVIGPVSGGS MNPVRSLGPA IVMGRYESVW
      241 IYLVAPVSGM LLGALCNKAV RQADELVEFL CRGGRGGAAA RSGSN
//
LOCUS       BdPIP1-1       280 aa
DEFINITION  BdPIP1-1     280 aa
TITLE       BdPIP1-1
ORIGIN
```

```
        1 MEGKEEDVRL GANKFSERQP IGTAAQGGGD DKDYKEPPPA PLFEPGELKS WSFYRAGIAE
       61 FIATFLFLYI TVLTVMGVSK SPSKCATVGI QGIAWSFGGM IFALVYCTAG ISGGHINPAV
      121 TFGLFLARKL SLTRAIFYIV MQCLGAICGA GVVKGFQQGL YMGNGGGANA VASGYTKGDG
      181 LGAEIIGTFV LVYTVFSATD AKRNARDSHV PILAPLPIGF AVFLVHLATI PITTLMANVL
      241 SSQAEYIRHW IFWVGPFVGA ALAAVYHQVI IRAIPFKSRS
//
LOCUS       BdPIP1-2       288 aa
DEFINITION  BdPIP1-2     288 aa
TITLE       BdPIP1-2
ORIGIN

        1 MEGKEEDVRL GANKFSERQP IGTAAQGSED KDYKEPPPAP LFEPGELKSW SFYRAGIAEF
       61 MATFLFLYVT ILTVMGVNNS PSKCATVGIQ GIAWSFGGMI FALVYCTAGI SGGHINPAVT
      121 FGLFLARKLS LTRAVFYIVM QCLGAICGAG VVKGFQSGLY MSSGGGANAV AAGYTKGDGL
      181 GAEIVGTFVL VYTVFSATDA KRNARDSHVP ILAPLPIGFA VFLVHLATIP ITGTGINPAR
      241 SLGAAIIYNK SHNWADHWIF WVGPFIGAAL AAVYHQVVIR AIPFKTKS
//
LOCUS       BdPIP1-3       289 aa
DEFINITION  BdPIP1-3     289 aa
TITLE       BdPIP1-3
ORIGIN

        1 MEGKEEDVRL GANRYSERQP IGTAAQGGDS EKDYKEPPPA PIFEAEELTS WSFYRAGIAE
       61 FIATFLFLYI SVLTVMGVSS SSSKCGTVGI QGIAWSFGGM IFVLVYCTAG ISGGHINPAV
      121 TFGLFLARKL SLTRAVFYMV MQCLGAICGA GVVKGFQTGL YMGKGGGANS VAVGYTKGDG
      181 LGAEIVGTFV LVYTVFSATD AKRSARDSHV PILAPLPIGF AVFLVHLATI PITGTGINPA
      241 RSLGAAIIYN RKQAWDDHWI FWVGPFIGAA LAAIYHVVVI RAIPFKSRD
//
LOCUS       BdPIP2-1       290 aa
DEFINITION  BdPIP2-1     290 aa
TITLE       BdPIP2-1
ORIGIN

        1 MAKDEVMESG GGHQDFAAKD YTDPPPAPLI DAAELASWSL YRAVIAEFIA TLLFLYITVA
       61 TVIGYKHQTD VSASGPDAAC GGVGILGIAW AFGGMIFVLV YCTAGISGGH INPAVTFGLF
      121 LARKVSLVRA LLYMIAQCLG AMCGVGLVKA FQSAYFVRYG GGANTLAAGY SKGTGLAAEI
      181 IGTFVLVYTV FSATDPKRSA RDSHVPVLAP LPIGFAVFMV HLATIPITGT GINPARSLGA
      241 AVIFNKDKAW DDQWIFWVGP MVGAAIAAFY HQYILRAGAI KALGSFRSNA
//
LOCUS       BdPIP2-2       290 aa
DEFINITION  BdPIP2-2     290 aa
TITLE       BdPIP2-2
ORIGIN

        1 MAPSVCTACS NGNGNDNNGK DYLDPPPALL LDTAELTKWS LYRAAIAEFT ATLLFVYIAI
       61 ATVIGHNHQS STACSSGAGI LGIAWSFGGT IFVLVYSTAG ISGGHINPAV TFALLLARKL
      121 TLLRAVFYIV SQCVGAIFGA AIARSVHGRA RYLLHGGGAN ELAPGFSAAA GLGAEVVGTF
      181 VLVYIVFSAT DPKRKARDCH VPVLAPLPIG FAVFMVHLAT IPITGTGINP ARSLGAAVAY
      241 NGDKAWSEHW IFWVGPLLGA ALAMVYHEFV LRGKAMASFR RSNYTAAAAY
//
LOCUS       BdPIP2-3       295 aa
DEFINITION  BdPIP2-3     295 aa
TITLE       BdPIP2-3
ORIGIN
```

```
        1 MTPDTDSNNK VAKPSAGDDT EIPSKDYLNP PPTPLFDGSE LGKWSLYRAV IAEFTATLLF
       61 VYVTVATVIG HKRQQQQPDT AGAGVGLLGI AWAFGGSIAV LVYCTAGISG GHINPAVTFG
      121 LLLARKVSLP RAGLYMLAQC LGAICGAGLV RTVNGGDAYL KHGGGANEVA DGYSNAAGFV
      181 AEVVGTFVLV YTVFSATDPK RMARDSHISV LAPLLIGFSV FVVHLATIPV TGTSINPARS
      241 FGPAVVYNGE KAWADLWIFW VGPFTGAAVA MVYHQFVLRN TVSVFFHRSD YAAAV
//
LOCUS       BdPIP2-4       288 aa
DEFINITION  BdPIP2-4     288 aa
TITLE       BdPIP2-4
ORIGIN

        1 MAKDIEASAP EGGEFSAKDY SDPPPAPIVD FEELTKWSLY RAAIAEFVAT LLFLYITVAT
       61 VIGYKHQTDV SASGPDAACG GVGILGIAWA FGGMIFVLVY CTAGVSGGHI NPAVTFGLFL
      121 ARKVSLVRAV LYIVAQCLGA ICGVGLVKGF QSAYYVRYGG GANELSAGYS KGTGLAAEII
      181 GTFVLVYTVF SATDPKRNAR DSHIPVLAPL PIGFAVFMVH LATIPITGTG INPARSLGAA
      241 VIYNTDKAWD DQWIFWVGPL IGAAIAAAYH QYVLRASAAK LGSYRSSS
//
LOCUS       BdPIP2-5       287 aa
DEFINITION  BdPIP2-5     287 aa
TITLE       BdPIP2-5
ORIGIN

        1 MAKDIEAAPG EYAAKDYSDP PPAPLFDAEE LTKWSLYRAA IAEFVATLLF LYITVATVIG
       61 YKHQADASAS GADAACGGVG ILGIAWAFGG MIFVLVYCTA GVSGGHINPA VTFGLFLARK
      121 VSLVRAVLYM VAQCLGAICG VGLVKGFQSA YFVRYGGGAN GLSAGYSKGT GLAAEIIGTF
      181 VLVYTVFSAT DPKRSARDSH VPVLAPLPIG FAVFMVHLAT IPITGTGINP ARSLGAAVIY
      241 NNDKAWDDQW IFWVGPFIGA AIAAAYHQYV LRASATKLGS SASFGRN
//
LOCUS       BdPIP2-6       289 aa
DEFINITION  BdPIP2-6     289 aa
TITLE       BdPIP2-6
ORIGIN

        1 MGKEVDVASL EAGGGARDYS DPPPAPLIDI DELGKWSLYR AVIAEFVATL LFLYITVATV
       61 IGYKHQTDAS APGAGADAAC GGVGILGIAW AFGGMIFVLV YCTAGVSGGH INPAVTFGLF
      121 LARKVSLVRA LLYMVAQCLG AICGVGLVKG FQRDFYARYG GGANGVSAGY SMGTGLAAEI
      181 IGTFVLVYTV FSATDSKRNA RDSHVPVLAP LPIGFAVFMV HLATIPITGT GINPARSLGA
      241 AVVYNNDKAW SDQWIFWVGP FIGAAIAALY HQTVLRASAR GYGSFRSNA
//
LOCUS       BdPIP2-7       290 aa
DEFINITION  BdPIP2-7     290 aa
TITLE       BdPIP2-7
ORIGIN

        1 MSKEEVAAAD TSEQPITKAP YWDPPPAPLL DTSELSRWSL YRAAIAEFTA TFIFLYVSIA
       61 TVIGYKSQAA AETCTGVGYL GVAWSFGATI FVLVYCTGGV SGGHINPAVT FGLLVGRKLS
      121 LVRAVLYIVA QCLGAICGAG LVKGITGRSY ETLGGGANSV SDGFSVGAAF GAEIAGTFVL
      181 VYTVLSATDP KRTARDSFIP VLVPLPIGFA VFVVHLATIP ISGTGINPAR SLGAAVMYNQ
      241 HKAWKDHWIF WVGPLLGATV AAFYHRFVLR GEAAKALGSF RSTGAATART
//
LOCUS       BdPIP2-8       297 aa
DEFINITION  BdPIP2-8     297 aa
TITLE       BdPIP2-8
```

```
ORIGIN

        1 MPNTKEEVAA AKLAKQATLT APYWDPPAEL LDRSELSRWS LYRAVIAEFM ATLIFLYISI
       61 ATVIGYKSQA AAQACTGVGY LGVAWSFGAT IFVLVHCTGG LSGGHMNPAV TFGMLVGRKL
      121 SLVRASMYIA AQCLGAICGA AIVKAITRHA HSYEAFGGGA NTVADGFTVG GAIGAEIVGT
      181 FVLVYTVFSS TDPKRTARDS FVPVLQPLPI GFAVFVVHLA TIPVTGTGIN PARSLGAAVM
      241 YNQHIAWKHH WIFWVGPFVG ATVAALYHQC VLRGKAAKAL DSFRGTGAAT APQDAGT
//
LOCUS       BdSIP1-1       246 aa
DEFINITION  BdSIP1-1     246 aa
TITLE       BdSIP1-1
ORIGIN

        1 MAMAASAVKA AAADGVVTFL WVLCVSTLGA STAAVTRYLS LHEEGAAYAL LVTVSLLSLL
       61 LFAFNLLCDA LGGASFNPTA LAAFYAAGLT SPSLFSVALR LPAQAAGAVG GALAISELMP
      121 AQYKHMLGGP SLKVDPHTGA VAEGLLTFVI TLAVLWIIVR GPRNAVLKTA MLSVSTVSLV
      181 LAGAAYTGPS MNPANAFGWA YVNDRHNTWE QLYVYWICPF VGATLAAWTF RAVFPPPAPK
      241 PKAKKA
//
LOCUS       BdSIP2-1       194 aa
DEFINITION  BdSIP2-1     194 aa
TITLE       BdSIP2-1
ORIGIN

        1 MAWLTYAMRQ LTQPETGVEG GQTHQPRIWT TVKSLRGKIK PIRSWRLKSE LGQEFKVLGA
       61 VLGVTVIQEA FPKVGKGAVL SVGVHHGALV EGLATLMVVM MSVTLKKKEM NGFFMKTWIS
      121 SIWKNTIHIL SSHITGGIMN PASAFAWAYA RGDHTTFDHL LVYWLAPLQA TLLGVWVVTF
      181 LTKPKKIKDN KKDQ
//
LOCUS       BdTIP1-1       250 aa
DEFINITION  BdTIP1-1     250 aa
TITLE       BdTIP1-1
ORIGIN

        1 MPISRVAVGS HHEVYQAGAL KAAVAEFIST LIFVFAGQGS GMAFNKLNAG GAATPAGLIS
       61 AAIAHAFALF VAVSVGANIS GGHVNPAVTF GAFLGGNITL FRGLLYWVAQ LLGSTAACFL
      121 LRFSTGGLPT GTFGLTGVSV WEALVLEIVM TFGLVYTVYA TAVDPKKGSI GTIAPLAIGF
      181 IVGANILVGG AFDGASMNPA VSFGPALVSW EWGYQWVYWV GPLIGGGLAG VIYEVLFISH
      241 THEQLPTTDY
//
LOCUS       BdTIP1-2       252 aa
DEFINITION  BdTIP1-2     252 aa
TITLE       BdTIP1-2
ORIGIN

        1 MPVSRIAIGS PGELSHPDTF KAAVAEFISM LIFVFAGSGS GMAFGKLTDG GATTPSGLIA
       61 AALAHAFALF VAVSVGANIS GGHVNPAVTF GAFIGGNISL LKAIVYWVAQ LLGSTAACLL
      121 LQISTGGASV GAFSLSAGVG VWNAVVFEIV MTFGLVYTVY ATAVDPKRGD LGVIAPIAIG
      181 FIVGANILAG GAFDGASMNP AVSFGPAVVS GVWENHWVYW LGPFAGAAIA ALVYDIVFIG
      241 QRPHDQLPSA EY
//
LOCUS       BdTIP2-1       249 aa
DEFINITION  BdTIP2-1     249 aa
TITLE       BdTIP2-1
```

```
ORIGIN

        1 MVKLAFGSCG DSFSATSIKS YVAEFIATLL FVFAGVGSAI AYGKLTDDGA LDPAGLVAVA
       61 IAHAFALFVG VAIAANISGG HLNPAVTFGL AVGGNITILT GIFYWVAQLL GATVACFLLK
      121 FVTHGKAIPT HGVAAGMNEL EGVVMEIVIT FALVYTVYAT AADPKKGSLG TIAPIAIGFI
      181 VGANILAAGP FSGGSMNPAR SFGPAVAAGN FAGNWVYWVG PLIGGGLAGL VYGDVFIASY
      241 QPVADQDYA
//
LOCUS       BdTIP2-2       248 aa
DEFINITION  BdTIP2-2     248 aa
TITLE       BdTIP2-2
ORIGIN

        1 MVKLAFGSLG DSFSVTSIRS YVAEFIATLL FVFAGVGSAI AYGQLTKGGA LDPSGLVAIA
       61 IAHAFALFVG VAMAANISGG HLNPAVTFGL AVGGHITILT GIFYWVAQLL GASVACLLLQ
      121 FVTHTQAMPR HAVAGISEME GVVMEIVITF ALVYTVYATA ADPKKGSLGT IAPIAIGFIV
      181 GANILAAGPF SGGSMNPARS FGPAVAAGDF SGHWVYWVGP LIGGGLAGLV YGDVFIASYQ
      241 PVAQQEYP
//
LOCUS       BdTIP3-1       265 aa
DEFINITION  BdTIP3-1     265 aa
TITLE       BdTIP3-1
ORIGIN

        1 MSTAARSTAG RRGFTVGRSE DATHPDTIRA AISEFLATAI FVFAAEGSIL SLGKLYHDMS
       61 TAGGLVAVAL AHALALAVAV SVAVNISGGH VNPAITFGAL LGGRITLVRA LFYWVAQLLG
      121 AIVASLLLRL TTGGMRPPGF SLAAGVRDWH AVLLEAVMTF GLMYAYYATV MDPKRGSVGT
      181 IGPLAVGFLL GANMLAGGPF DGAGMNPARV FGPALVGWRW GHHWVYWVGP FLGAGIAGLL
      241 YEYLVIPASE TAAAHTHQPL APEDY
//
LOCUS       BdTIP3-2       262 aa
DEFINITION  BdTIP3-2     262 aa
TITLE       BdTIP3-2
ORIGIN

        1 MLPTRFPSRR ADDAAGPEPL MPSTSRAVLS ELVATAVFVF AAEGSLYGLW KMYSETGTVG
       61 GLLVVAVAHA LALAAAVALS INTSGGHVNP AVTFGVLVGR RISFARAVLY WVAQLLGSVL
      121 AALLLSLVSG GARPMGSALG HGIHERHALL LEVVMTFGLM YTVYATAVDR NDGVGAIAPV
      181 AIGFVLGANI LTGGPFDGAA MNPARAFGPA LVGWNWSHHW VYWVGPMIGA GLAGALYEFV
      241 VGEQPDQAPP AAARLPEPVE DY
//
LOCUS       BdTIP4-1       252 aa
DEFINITION  BdTIP4-1     252 aa
TITLE       BdTIP4-1
ORIGIN

        1 MATKHVDSFD ERDVVDAGCV RAVLGELVLT FLFVFTGVAA VMAAGVPEVP GAAMPMATLA
       61 GVALAQALAA GVLVTAGFHV SGGHLNPAVT LALLARGHIS AFRAVLYVAA QLLASSLACI
      121 LLRYLTGGQA TPVHALGAGI GPMQGLVMEI IFTFSLLFVV YATILDPRTT VPGYGPMLTG
      181 LIVGANTLAG GNFSGASMNP ARSFGPALAT GVWTNHWVYW VGPLVGGPLA GFVYESLFLV
      241 KRTHEPLLAG DF
//
LOCUS       BdTIP4-2       252 aa
DEFINITION  BdTIP4-2     252 aa
```

```
TITLE       BdTIP4-2
ORIGIN

        1 MPLPLTKLVL GHGREASDPG CLRAVLGELV LTFLFVFVGV GSTITAGSAA AAGADPSAAL
       61 IAVALAHALV VAVFATAGFH ISGAHMNPAV TLSLAVGGHI TLLRSSFFVV AQMLGSSCAC
      121 LLLRALTGGL VTPVHALAAG VGPIQGVVAE VVFTFTLLFT IYAAILDPRS SAPGFGPLLT
      181 GLLVGANTIA GGALTGASMN PARSFGPALA SGDWANHWVY WVGPLAGGPL AVAVYEFVFT
      241 VPPTHQQLPT VE
//
LOCUS       BdTIP4-3       254 aa
DEFINITION  BdTIP4-3     254 aa
TITLE       BdTIP4-3
ORIGIN

        1 MAKLALGHLR EAAEPGCARA VLAEAVLTFL FVFSGVASAM AAGKLGGGAD TIMGLTAVAS
       61 AHAMAVAVMV SAGLHVSGGH INPAVTLGLA AGGHITLFRS ALYVPAQLLG SSLACLLLSF
      121 LSGSGAGAPP IPVHALAAGV GAAQGLLWEA TLTFSLLFAV YATVVDPART VGNLGPLLVG
      181 LVVGANALAG GPFSGASMNP ARSFGPALVT GEWAGHWVYW VGPMIGGLLA GLVYDGVFMV
      241 RPGHQQLPTD DAEF
//
LOCUS       BdTIP5-1       263 aa
DEFINITION  BdTIP5-1     263 aa
TITLE       BdTIP5-1
ORIGIN

        1 MASNLRAHMK HCFSAASLRS YLAEFVSTFL FVFVAVGSAM SARMLTPDVT SDASSLVATA
       61 VAQSFGLFAA VFIAADVSGG HVNPAVTFAL AIGGHIAVPS AIFYWSCQLL GSTFACLVLH
      121 YFSAGQAVPT TRIAVEMTGF GAAIVEGVMT FMLVYAVHVA ADPRACGRSR GLATTAMGSL
      181 VVGLVAGACV LAAGSLTGAS MNPARSFGPA VVSGDFKNQA VYWVGPMIGA AVAALVHQNL
      241 VFPSAPEPLP HEVRHGSVET VVA
//
LOCUS       BrNIP1-1       298 aa
DEFINITION  BrNIP1-1     298 aa
TITLE       BrNIP1-1
ORIGIN

        1 MAEISGNGHG DSKEGAVMVN INQEVELQQQ QKEAIHTTKS MKKQDSVLSF SVPFLQKLMA
       61 EILGTYFLIF AGCASVAVNA QHDKAVTLPG IAIVWGLTVM VLVYSLGHIS GAHFNPAVTI
      121 AFASCGRFPL KQVPAYVISQ VIGSTLAAAT LRLLFGLDQD VCSGKHDVFV GTLPSGSDLQ
      181 SFVIEFIITF YLMFIISGVA TDNRAIGELA GLAVGSTVLL NVIIAGPVSG ASMNPGRSLG
      241 PAMVYNCYKG IWIYIASPIL GAVAGAWVYN TVRYTDKPLR EITKSGSFLK SVRNGSSR
//
LOCUS       BrNIP1-2       297 aa
DEFINITION  BrNIP1-2     297 aa
TITLE       BrNIP1-2
ORIGIN

        1 MAEISGNGHD DAREGAVVVN INEEHERQQH KEAIHISKSM KKQDSLLSIS VPFLQKLMAE
       61 ILGTYFLIFA GCASVAVNAQ HDKAVTLPGI AIVWGLTVMV LVYSLGHISG AHFNPAVTIA
      121 FASSGRFPLK QVPAYVISQV IGSTLAAATL RLLFGLDQDV CSGKHDVFVG TLPAGSDLQS
      181 FVIEFIITFY LMFIISGVAT DNRAIGELAG LAVGSTVLLN VIIAGPVSGA SMNPGRSLGP
      241 AMVYNCYKGI WIYIASPILG AVAGAWVYNT VRYTDKPLRE ITKSGSFLKA LQNSSSR
//
LOCUS       BrNIP2-1       286 aa
```

```
DEFINITION  BrNIP2-1     286 aa
TITLE       BrNIP2-1
ORIGIN

        1 MDDISVSKSN HGNVVVLNIQ APPVSKTSFP SSPSTSPPLL SVHFLQKLIA ELVGTYYLIF
       61 AGCAAIAVNA QHNNVVTLVG IAVVWGLVVM VLVYSLGHIS AHFNPAVTIA LASCKRFPLY
      121 QLPAYLIVQV IGSTLASATL RLLFDLNNDV CSKKHDVFLG SSPSGTDLQA FGMEFIITGF
      181 LMIVVCAVTT SKRTSEELEG LIIGATVTLN VIFAGEVSGA SMNPARSLGP ALVWGCYKGI
      241 WIYLLAPTLG AVLAALIHKL LPATQKANSE FSKTGSSHKR ITDLPL
//
LOCUS       BrNIP2-2       287 aa
DEFINITION  BrNIP2-2     287 aa
TITLE       BrNIP2-2
ORIGIN

        1 MDDISVSKSN NGNVVVLNIQ APPAVSKTQL PSSPPTSPPL LSVHFMQKLI AELVGTYYLI
       61 FAGCAAIAVN AQHNNVVTLV GIAVVWGLVV MVLVYSLGHI SAHFNPAVTI ALASCKRFPL
      121 YQLPAYLIVQ VIGSTLASAT LRLLFDLNND VCSKKHDVFL GSSPSGTDLQ AFGMEFIITG
      181 FLMIVVCAVT TSKRTTKELE GLIIGATVTL NVIFAGEVSG ASMNPARSIG PALVWGCYQG
      241 IWIYLLAPTL GAVSAALIHK LLPATQKANP EFSKTGSSHK RVTDLPL
//
LOCUS       BrNIP3-1       337 aa
DEFINITION  BrNIP3-1     337 aa
TITLE       BrNIP3-1
ORIGIN

        1 MSKPLTFSEK SILSMAEISG ITVRTQTTIL NIEDGRSSGD SRLPDSPCSL ICISFVQKLI
       61 GEFVGTFSLV FAGCSATVVN DTYGELVTLP GIALAWGLTV MVMTYSIGHV SGAHFNPAVS
      121 IALASSRKFP FKQVPGYIAA QLLGSTLAAE ALRLMFHLNN NVCSLKGDVY VGTRPSGSNT
      181 ATFIVELIAT FNLLFVISAV ATDKRANRSF AGVAIGATVV LNILFSGPIS GASMNPARSL
      241 APAYIWGCYK NLWLYIVSPV IGALIGAWTY NMLRSTNKSY GEIIRPNCNK VSSNDHQEAS
      301 QDDSCVLRVV DPNNRKIFIL SSPTDINETC NVTCKLA
//
LOCUS       BrNIP3-2       323 aa
DEFINITION  BrNIP3-2     323 aa
TITLE       BrNIP3-2
ORIGIN

        1 MAEISDTTPQ TQTVTFDIED GGSGGDSRSP GISLPLVSVS FVQKLIGEFV GTFSLIFAGC
       61 AAIVVNDTYG KAVTLPGIAL VWGLTVMVMI YSIGHVSGAH FNPAVSIAFA SSRKFPFKQV
      121 PGYIAAQLLG STLAAEALRL VFHLDDNVCS LKGDIYVGTY PSSSNTATFV MEFITTFNLM
      181 FVISAVATDK RANGSFAGIA IGATVVLDIL FCGPISGASM NPARSLGPAL IWGCYKDLWL
      241 YIVSPVIGAL TGAWTYDMLR STKKSYGEII RPNCNKISSR DRQEASQDEI CVLQVVNQAN
      301 RKEFICSSPT DINDKRNVTC KLP
//
LOCUS       BrNIP4-1       164 aa
DEFINITION  BrNIP4-1     164 aa
TITLE       BrNIP4-1
ORIGIN

        1 MVMIYSVGHI SGAHFNPAVT ICFAIFRRFP WYQVPSYIGA QLAGSLLASL TLRLMFKVTP
       61 EAFFGTTPAD SAARALASEI IISFLLIPIS GASMNPARSL GPAIVMGVYK GIWVYIVGPI
      121 IGIVAGGFVY NFIRFTDKPI GELTKSSSFL RKASSNNNAS SSNS
//
```

```
LOCUS       BrNIP4-2       197 aa
DEFINITION  BrNIP4-2     197 aa
TITLE       BrNIP4-2
ORIGIN

        1 MVMIYSVGHI SGAHFNPAVT ICFAIFRRFP WYQVPSYIGA QLAGSLLASL TLRLMFKVTP
       61 EAFFGTTPAD SAARALASEI IISFLLMFVI SGVATDSRAI GELAGIAVGM TIILNVFVAG
      121 PISGASMNPA RSLGPAIVMG VYKGIWVYIV GPIIGIVAGG FVYNFIRFTD KPIGELTKSS
      181 SFLRKASSNN NASSSNS
//
LOCUS       BrNIP4-3       276 aa
DEFINITION  BrNIP4-3     276 aa
TITLE       BrNIP4-3
ORIGIN

        1 MTSNGEEIEE EEISRIEKGQ AKNCHGGIET VICTSPSIVC LTQKLIAEMI GTYFVIFSGC
       61 GVVVVNVLYG GTVTFPGVCV TWGLIVMVMI YSTGHISGAH FNPAVTLTFA IFRRFPWYQV
      121 PLYVGAQLAG SLLASLTIRL MFKVTPEAYF GTTPTDSAAR ALVAEIIISF LLMFVISGVS
      181 TDSRAIGELA GIAVGMTIML NVFVAGPISG ASMNPARSLG PAIVMGVYKD IWVYIVGPIS
      241 GVMAGGFVYN FIRFTDKPLR ELTKSASFLR SISPKQ
//
LOCUS       BrNIP4-4       283 aa
DEFINITION  BrNIP4-4     283 aa
TITLE       BrNIP4-4
ORIGIN

        1 MTSHVEEIEE EEISKIEKGK GKDCHRGIET VICTSPTTVC LTQKLIAEMI GTYFLIFAGC
       61 GVVVVNVLYG GTVTFPGICV TWGLIVMVMI YSTGHISGAH FNPAVTLTFA VFRRFPWYQV
      121 PLYIGAQLTG SLLGSLTLKL MFHVTPAAYF GTIPSDSAAQ ALAAEIIISF LLMFVISGVA
      181 TDNRAVGELA GIAVGMTIML NVFVAGPVSG ASMNPARSLG PAIVMGVYDG LWIYIVGPLV
      241 GIMAGGFVYN LIRFTDKPLK ELTRNGSFLR SASPKHKTST SKS
//
LOCUS       BrNIP5-1       301 aa
DEFINITION  BrNIP5-1     301 aa
TITLE       BrNIP5-1
ORIGIN

        1 MSPPEAEMGA VAVTAPPTPG TPGGPLITGM RVDSMSFDHR KPIPPCKCLP VMGNRWGQHD
       61 TCFADFPSPG VSLTRKLGAE FVGTFILIFT ATAGPIVNQK YDGAETLIGN AACAGLAVMI
      121 IILSTGHISG AHLNPSLTIA FAALRHFPWA HVPAYIAAQV SASVCASFAL KAVFHPFMSG
      181 GVTVPSVSVG QAFALEFIIS FILLFVVTAV ATDTRAVGEL AGIAVGATVM LNILVAGPST
      241 GASMNPVRTL GPALASGNYR LLWVYLVAPT LGAISGAAVY TGVKLNDNVT DPPRQVRSFR
      301 R
//
LOCUS       BrNIP5-2       301 aa
DEFINITION  BrNIP5-2     301 aa
TITLE       BrNIP5-2
ORIGIN

        1 MAPTEAEMGA VAVTAPPTPG TPGGPLITGM RVDSMSFDHR KPMPPCKCLP VMGHTWGQPD
       61 TCFTDFPSPV VSLTRKLGAE FVGTFILIFT ATAGPIVNQK YDGAETLIGN AACAGLAVMI
      121 IILSTGHISG AHLNPSLTIA FAALRHFPWA HVPAYIAAQV SASICASFAL KAVFHPFMSG
      181 GVTVPSVSVG QAFALEFIIT FILLFVVTAV ATDTRAVGEL AGIAVGATVM LNILVAGPSS
      241 GGSMNPVRTL GPALASGNYR SLWVYLVAPT LGAISGAAVY TGVKLNDSAT DPPRQVRSFR
```

```
      301 R
//
LOCUS       BrNIP6-1       305 aa
DEFINITION  BrNIP6-1     305 aa
TITLE       BrNIP6-1
ORIGIN

        1 MDHEEIPSMP STPATTPGTP GAPLFGGFDG KRSGHNGRYT PKSLLKSCKC FSVDNEWALE
       61 DGRLPPVSCA LPPPNVSLYR KLGAEFVGTL ILIFAGTATA IVNQKTDGAV TLIGCAASAG
      121 LAVMIVILST GHISGAHLNP AVTISFAALK HFPWKHVPVY IGAQVLASVC AAFALKAVFE
      181 PTMSGGVTVP TVALSQAFAL EFIISFNLMF VVTAVATDTR AVGELAGIAV GATVMLNILI
      241 AGPATSASMN PVRTLGPAIA ANNYRAIWVY LTAPILGALI GAGTYTVVKL PEEDEEHKET
      301 RSFRR
//
LOCUS       BrNIP6-2       305 aa
DEFINITION  BrNIP6-2     305 aa
TITLE       BrNIP6-2
ORIGIN

        1 MDHEEIPSMP STPATTPGTP GAPLFGGFEG KRNGHNGKYT PKSILKSCKC FNVDNEWALE
       61 DGRLPPVSCA LPPPNVSLYR KLGAEFVGTL ILIFAGAATA IVNQKTDGAV TLIGCAASAG
      121 LAVMIVILST GHISGAHLNP AITIAFAALK HFPWKHVPVY IGAQVMASLC AAFALKAVFE
      181 PTMSGGVTVP TVGLSQAFAL EFIISFNLMF VVTAVATDTR AVGELAGIAV GATVMLNILI
      241 AGPATSASMN PVRTLGPAIA ANNYRAIWVY LTSPILGALI GAGTYTIVKL PEEDEAPKEK
      301 RSFRR
//
LOCUS       BrNIP7-1       272 aa
DEFINITION  BrNIP7-1     272 aa
TITLE       BrNIP7-1
ORIGIN

        1 MNVEVRSRVF DQEAGSTLSS LRDGDLSTQR LFRCIPYELD LNPLRIVIAE LVGTFILMFS
       61 VCGVISSTQL SGGHVGLLEY AATAGLSVVV VVYSIGHISG AHLNPSITIA FALFGGFPWS
      121 QVPLYIMAQT LGATAATLAG VSVYGVNPDL MITKPALSCV SAFFVELVAT SIVVFLASAL
      181 HCGPHQNSSN LTGLVIGAVI SLGVLITGPI SGGSMNPARS LGPAVVAWDF EYIWVYMTAP
      241 VIGAIMGVLT YRTISLKSRP SPHSPPVSSL LR
//
LOCUS       BrPIP1-1       286 aa
DEFINITION  BrPIP1-1     286 aa
TITLE       BrPIP1-1
ORIGIN

        1 MEGKEEDVRV GANKFPERQP IGTSAQSDKD YNEPPPAPLF EPGELSSWSF WRAGIAEFIA
       61 TFLFLYITVL TVMGVKRSPN MCSSVGIQGI AWAFGGMIFA LVYCTAGISG GHINPAVTFG
      121 LFLARKLSLT RALYYIVMQC WGAICGAGVV KGFQPNQYQA IGGGANTVAP GYTKGSGLGA
      181 EIIGTFVLVY TVFSATDAKR NARDSHVPIL APLPIGFAVF LVHLATIPIT GTGINPARSL
      241 GAAIIYNKDH SWDDHWVFWV GPFIGAALAA LYHVIVIRAI PFKSRN
//
LOCUS       BrPIP1-2       286 aa
DEFINITION  BrPIP1-2     286 aa
TITLE       BrPIP1-2
ORIGIN

        1 MEGKEEDVRV GANKFPERQP IGTSAQTDKD YKEPPPAPLF EPGELSSWSF WRAGIAEFIA
```

```
       61 TFLFLYITVL TVMGVKRAPN MCASVGIQGI AWAFGGMIFA LVYCTAGISG GHINPAVTFG
      121 LFLARKLSLT RAVPYIVMQC LGAVCGAGVV KGFQPTPYQT LGGGANTVAP GYSKGSGLGA
      181 EIIGTFVLVY TVFSATDAKR SARDSHVPIL APLPIGFAVF LVHLATIPIT GTGINPARSL
      241 GAAIIYNKDH SWDDHWIFWV GPFIGAALAA LYHQLVIRAI PFKTRS
//
LOCUS       BrPIP1-3       286 aa
DEFINITION  BrPIP1-3     286 aa
TITLE       BrPIP1-3
ORIGIN

        1 MEGKEEDVRV GANKFPERQP IGTSAQSDKD YKEPPPAPLF EPGELSSWSF WRAGIAEFIA
       61 TFLFLYITVL TVMGVKRAPN MCASVGIQGI AWAFGGMIFA LVYCTAGISG GHINPAVTFG
      121 LFLARKLSLT RAVFYIVMQC LGAVCGAGVV KGFQPSPYQT LGGGANTVAP GYTKGSGLGA
      181 EIIGTFVLVY TVFSATDAKR SARDSHVPIL APLPIGFAVF LVHLATIPIT GTGINPARSL
      241 GAAIIYNKDH AWDDHWIFWV GPFIGAALAA LYHQLVIRAI PFKTRS
//
LOCUS       BrPIP1-4       288 aa
DEFINITION  BrPIP1-4     288 aa
TITLE       BrPIP1-4
ORIGIN

        1 MEGKEEDVRV GANKFPERQP IGTSAQSDKD YKEPPPAPLF EPGELSSWSF WRAGIAEFIA
       61 TFLFLYITVL TVMGVKRAPN MCASVGIQGI AWAFGGMIFA LVYCTAGISG GHINPAVTFG
      121 LFLARKLSLT RAVFYMIMQC LGAICGAGVV KGFQPTPYQT LGGGANTVAP GYSKGSGLGA
      181 EIIGTFVLVY TVFSATDAKR SARDSHVPIL APLPIGFAVF LVHLATIPIT GTGINPARSL
      241 GAAIIYNKDH SWDDHWIFWV GPFIGAALAA LYHTIVIRAI PFKSKSKS
//
LOCUS       BrPIP1-5       287 aa
DEFINITION  BrPIP1-5     287 aa
TITLE       BrPIP1-5
ORIGIN

        1 MEGKEEDVNV GANKFPERQP IGTAAQTEGK DYKEPPPAPF FEPGELKSWS FYRAGIAEFI
       61 ATFLFLYVTV LTVMGVKRAP NMCASVGIQG IAWAFGGMIF ALVYCTAGIS GGHINPAVTF
      121 GLFLARKLSL TRTVFYIVMQ CLGAMCGAGV VKGFQPWPYQ SNGGGANLVA HGYTKGSGLG
      181 AEIVGTFVLV YTVFSATDAK RSARDSHVPI LAPLPIGFAV FLVHLATIPI TGTGINPARS
      241 LGAAIIYNKD HAWDDHWIFW VGPFIGAALA ALYHQIVIRA IPFKSKT
//
LOCUS       BrPIP1-6       286 aa
DEFINITION  BrPIP1-6     286 aa
TITLE       BrPIP1-6
ORIGIN

        1 MEGKEEDIRV GANKFPERQP IGTSAQSDKD YNEPPPAPLF EPGELSSWSF WRAGIAEFIA
       61 TFLFLYITVL TVMGVKRSPN MCASVGIQGI AWAFGGMIFA LVYCTAGISG GHINPAVTFG
      121 LFLARKLSLT RALYYIVMQC LGAICGAGVV KGFQPKQYQA LGGGANTVAP GYTKGSGLGA
      181 EIIGTFVLVY TVFSATDAKR NARDSHVPIL APLPIGFAVF LVHLATIRMT GTGINPARSL
      241 GAAIIYNKDH SWDDHWVFWV GPFIGAALAA LYHVIVIRAI PFKSRS
//
LOCUS       BrPIP1-7       286 aa
DEFINITION  BrPIP1-7     286 aa
TITLE       BrPIP1-7
ORIGIN
```

```
        1 MEGKEEDVRV GANKFPERQP IGTSAQSDKD CKEPPPAPLF EPGELASWSF GRAGIAEFIA
       61 AFLFLYITVL TVMGVKRSPN MCASVGIQGI AWAFGGMIFA LVYCTAGISG GHINPAVTFG
      121 LFLARKLSLT RAVYYIVMQC LGAICGAGVV KGFQPKQYQA LGGGANTVAP GYTKGSGLGA
      181 EIIGTFVLVY TVFSATDAKR NARDSHVPIL APLPIGFAVF LVHLATIPIT GTGINPARSL
      241 GAAIISNKDN AWDDHWVFWV GPFIGAALAA LYHVIVIRAI PFKSRS
//
LOCUS       BrPIP1-8       280 aa
DEFINITION  BrPIP1-8     280 aa
TITLE       BrPIP1-8
ORIGIN

        1 MEGKEEDVRV GANKFPERQP IGTSAQSDKD YKEPPPAPLF EPGELGSWSF WRAGIAEFIA
       61 TFLFLYITVL TVMGVKRAPN MCASVGIQGI AWAFGGMIFA LVYCTAGISG GHINPAVTFG
      121 LFLARKLSLT RAVYYIVMQC LGAICGAGVV KGFQPKQYQA LGGGANTVAP VTPKEVVLEL
      181 RLLEPLSLFT PSFPPLTPRE TLILAPLPIG FAVFLVHLAT IPITGTGINP ARSLGAAIIF
      241 NKDNAWDDHW VFWVGPFIGA ALAALYHVIV IRAIPFKSRS
//
LOCUS       BrPIP2-1       287 aa
DEFINITION  BrPIP2-1     287 aa
TITLE       BrPIP2-1
ORIGIN

        1 MAKDVEAVSG EGFQTRDYQD PPPAPLFDPA ELTKWSFYRA VIAEFVATLL FLYITVLTVI
       61 GYKIQTDSTA GGVDCGGVGI LGIAWAFGGM IFILVYCTAG ISGGHINPAV TFGLLLARKV
      121 SLVRAILYMV AQCLGAICGV GFVKAFQSSY YVRYGGGANS LADGYSTGTG LAAEIIGTFV
      181 LVYTVFSATD PKRSARDSHV PVLAPLPIGF AVFMVHLATI PITGTGINPA RSFGAAVIFN
      241 ESKPWDDHWI FWVGPFVGAA IAAFYHQFVL RASGSKSLGS FRSAANV
//
LOCUS       BrPIP2-2       238 aa
DEFINITION  BrPIP2-2     238 aa
TITLE       BrPIP2-2
ORIGIN

        1 MAKDVEGAEG FAARDYEDPP PTPFFDAEEL TKWSLYRAVI AEFVATLLFL YVTVLTVIGY
       61 KISSDTKAGG DECGGVGILG ISWAFGGMIF ILVYCTAGIS GGHINPAVTF GLFLARKVSL
      121 VRAVLYMVAQ CLGAICGVGF VKAFQSAYYV RYGGGANSLA DGYSTGTGLA AEIIGTFVLV
      181 YTVFSATDPK RNARDSHVPW IFWVGPFIGA AIAAFYHQFV LRASGSKSLG SFRSAANV
//
LOCUS       BrPIP2-3       231 aa
DEFINITION  BrPIP2-3     231 aa
TITLE       BrPIP2-3
ORIGIN

        1 MRRADQVISS DTKAGGDECG GVGILGISWA FGGMIFILVY CTAGISGGHI NPAVTFGLFL
       61 ARKVSLVRAV LYMVAQCLGA ICGVGFVKAF QSAYYVRYGG GANSLADGYS TGTGLAAEII
      121 GTFVLVYTVF SATDPKRNAR DSHVPVLAPL PIGFAVFMVH LATIPITGTG INPARSFGAA
      181 VIYNESKPWD DHWIFWVGPF IGAAIAAFYH QFVLRASGSK SLGSFRSAAN V
//
LOCUS       BrPIP2-4       283 aa
DEFINITION  BrPIP2-4     283 aa
TITLE       BrPIP2-4
ORIGIN

        1 MAKDVEGAEG VTARDYEDPP PTPFFDAEEL TKWSLYRAVI AEFVATLLFL YVTVLTVIGY
```

```
      61 KISSDTKAGG DDCGGVGILG ISWAFGGMIF ILVYCTAGIS GGHINPAVTF GLFLARKVSL
     121 VRAVLYMVAQ CLGAICGVGF VKAFQSAYYV RYGGGANSLA DGYSTGTGLA AEIIGTFVLV
     181 YTVFSATDPK RNARDSHVPV LAPLPIGFAV FMVHLATIPI TGTGINPARS FGAAVIYNES
     241 KPWDDHWIFW VGPFIGAAIA AFYHQFVLRA SGSKSLGSFR SAA
//
LOCUS       BrPIP2-5       285 aa
DEFINITION  BrPIP2-5     285 aa
TITLE       BrPIP2-5
ORIGIN

       1 MAKDVEGAEG FAARDYQDPP PTPFFDAEEL TRWSLYRAVI AEFVATLLFL YVTVLTVIGY
      61 KISSDTTAGG VECGGVGILG IAWAFGGMIF ILVYCTAGIS GGHINPAVTF GLFLARKVTL
     121 VRALLYMVAQ CLGAICGVGF VKAFQSAYYV RYGGGANSLA DGYSTGTGLA AEIIGTFVLV
     181 YTVFSATDPK RNARDSHVPV LAPLPIGFAV FMVHLATIPI TGTGINPARS FGAAVIYNES
     241 KPWDDHWIFW VGPFIGAAIA AFYHQFVLRA SGSKSLGSFR SAANV
//
LOCUS       BrPIP2-6       260 aa
DEFINITION  BrPIP2-6     260 aa
TITLE       BrPIP2-6
ORIGIN

       1 MAKDLEVQEG GATAARDYVD PPPAPLLDME EFGKWSLYRA TDANAGGVDC GGVGILGIAW
      61 AFGGMIFVLV YCTAGVSGGH INPAVTFGLF LARKVSLVRT VLYIVAQCLG AICGCGLVKA
     121 FQSSYYTRYG GGANELADGY NKGTGLGAEI IGTFVLVYTV FSATDPKRSA RDSHIPVLAP
     181 LPIGFAVFMV HLATIPITGT GINPARSLGA AVIYNQEKAW DDQWIFWVGP MIGAAAAALY
     241 HQFVLRAAGI KSLGSFRSSA
//
LOCUS       BrPIP2-7       285 aa
DEFINITION  BrPIP2-7     285 aa
TITLE       BrPIP2-7
ORIGIN

       1 MAKDLEVQEG GAMAARDYQD PPPAPLFDME ELGKWSLYRA VIAEFVATLL FLYVSILTVI
      61 GYKAQTDANA GGVDCGGVGI LGIAWAFGGM IFVLVYCTAG ISGGHINPAV TVGLFLARKV
     121 SLVRTVLYIV AQCLGAICGC GLVKAFQSSY YNRYGGGANQ LADGYNKGTG LGAEIIGTFV
     181 LVYTVFSATD PKRSARDSHI PVLAPLPIGF AVFMVHLATI PITGTGINPA RSFGAAVIYN
     241 QEKAWDDQWI FWVGPMIGAA AAALYHQFVL RAAGIKSLGS FRSSA
//
LOCUS       BrPIP2-8       283 aa
DEFINITION  BrPIP2-8     283 aa
TITLE       BrPIP2-8
ORIGIN

       1 MAKDLEVQEG RAARDYQDPP PAPLFDMEEL GKWSLYRAVI AEFVATLLFL YVSILTVIGY
      61 KAQTDASAGG ADCGGVGILG IAWAFGGMIF VLVYCTAGIS GGHINPAVTF GLFLARKVSL
     121 VRTVLYIVAQ CLGAICGCGL VKAFQSSYYN RYGGGANQLA EGYNKGTGLG AEIIGTFVLV
     181 YPVFAATDPK RSARDSHIPV LAPLPIGFAA FMVHLATIPI TGTGINPARS FGAAVIYNQE
     241 KAWDDQWIFW VGPMIGAAAA ALYHQFVLRA AGIKSLGSFR SSA
//
LOCUS       BrPIP2-9       286 aa
DEFINITION  BrPIP2-9     286 aa
TITLE       BrPIP2-9
ORIGIN
```

```
        1 MTKDVAGEKG SFSGKDYQDP PPEPLFDATE LGRWSFYRAL IAEFIATLLF LYVTVMTVIG
       61 YKSQTDPALN PDQCAGVGVL GIAWAFGGLI FILVYCTVGI SGGHINPAVT FGLFLARKVT
      121 LVRAVMYMVA QCLGAICGVA LVKSFQSSYY TRYGGGANGL THGYSIGTGV AAEIIGTFVL
      181 VYTVFSATDP KRSARDSHVP VLAPLPIGFA VFIVHLATIP ITGTGINPAR SLGAAIICNK
      241 DQAWDHHWIF WVGPFAGAAL AAFYHQFVLR AGAVKALGSF RSQSHV
//
LOCUS       BrPIP2-10       286 aa
DEFINITION  BrPIP2-10     286 aa
TITLE       BrPIP2-10
ORIGIN

        1 MTKEVVGEKG SFSGKDYQDP PPEPLFDATE LGKWSFYRAL IAEFIATLLF LYVTVMTVIG
       61 YKSQTDPALN PDQCAGVGVL GIAWAFGGMI FILVYCTAGI SGGHINPAVT FGLLLARKVT
      121 LLRAVMYMVA QCLGAICGVA LVKSFQSSYY TRYGGGANGL SNGYSVGTGV AAEIIGTFVL
      181 VYTVFSATDP KRSARDSHVP VLAPLPIGFA VFIVHLATIP ITGTGINPAR SLGAAIIYNK
      241 DQAWDHHWIF WAGPFAGAAI AAFYHQFVLR AGAVKALGSF RSQSRV
//
LOCUS       BrPIP2-11       288 aa
DEFINITION  BrPIP2-11     288 aa
TITLE       BrPIP2-11
ORIGIN

        1 MSTDLTEEES LSGKDYQDPP RVKIFEAREL GKWSFYRAVI AEFIATLLFL YVTVLTVIGF
       61 KSQTDLQTGG GACASVGLLG ISWAFGGMIF ILVYCTAGIS GGHINPAVTF GLFLASKVSL
      121 VRAISYIVAQ CLGATCGVGL VKVFQKTYYN RYGGGANVLA DGYNVGVGVG AEIIGTFVLV
      181 YTVFSATDPK RNARDSHIPV LAPLPIGFSV FMVHLATIPI TGTGINPARS FGAAVIYNNQ
      241 KAWDDQWIFW VGPFVGAAIA ALYHQFVLRA GAMKAYGSVR SQLHELHA
//
LOCUS       BrPIP2-12       281 aa
DEFINITION  BrPIP2-12     281 aa
TITLE       BrPIP2-12
ORIGIN

        1 MSKEVSEEGQ THSHGKDYVD PPPAPLLDMG ELKSWSFYRA LIAEFIATLL FLYVTVATVI
       61 GHKKQTGPCD GVGLLGIAWA FGGMIFVLVY CTAGISGGHI NPAVTFGLFL ARKVSLVRAV
      121 GYMIAQCLGA ICGVGFVKAF MKTPYNTLGG GANTVADGYS TGTALGAEII GTFVLVYTVF
      181 SATDPKRSAR DSHIPVLAPL PIGFAVFMVH LATIPITGTG INPARSFGAA VIYNNEKAWD
      241 DHWIFWVGPF VGALAAAAYH QYILRAAAVK ALASFRSSAT N
//
LOCUS       BrPIP2-13       281 aa
DEFINITION  BrPIP2-13     281 aa
TITLE       BrPIP2-13
ORIGIN

        1 MSKEVSEEGQ THHHGKDYVD PPPAPLLDMA ELGKWSFYRA LIAEFVATLL FLYVTVATVI
       61 GHKKQTGPCD GVGLLGIAWA FGGMIFVLVY CTAGISGGHI NPAVTFGLFL ARKVSLVRAV
      121 GYMIAQCLGA VCGVGFVKAF MKTPYNTLGG GANTVADGYS NGTALGAEII GTFVLVYTVF
      181 SATDPKRSAR DSHIPVLAPL PIGFAVFMVH LATIPITGTG INPARSFGAA VIYNNEKAWD
      241 DHWIFWVGPF VGALAAAAYH QYILRAAAIK ALASFRSNAT N
//
LOCUS       BrPIP2-14       281 aa
DEFINITION  BrPIP2-14     281 aa
TITLE       BrPIP2-14
ORIGIN
```

```
        1 MSKEVSEEGH TQSHGKDYVD PPPAPFLDMG ELKSWSFYRA LIAEFIATLL FLYVTVATVI
       61 GHKKQTGPCD GVGLLGIAWA FGGMIFVLVY CTAGISGGHI NPAGTFGLFL ARKVSLVRAV
      121 GYMIAQCLGA ICGVGFVKAF MKTPYNTLGG GANTVAPGYS KGTALGAEII GTFVLVYTVF
      181 SATDPKRSAR DSHIPVLAPL PIGFAVFMVH LATIPITGTG INPARSFGAA VIYNNEKARD
      241 DHWIFWVGPF VGALAAAAYH QYILRAAAVK ALASFRSSAT N
//
LOCUS       BrSIP1-1       239 aa
DEFINITION  BrSIP1-1     239 aa
TITLE       BrSIP1-1
ORIGIN

        1 MGVVKSAIGD MLMTFSWVVL SATFGLQTTE IISAAGFQGI AWAPLAITTF LIFFYVSIFT
       61 VVFGSASFNP TGNAAFYAAG VPGDTLFTLA IRLPAQAAGA AGGALAIMEF IPEKYKHMIS
      121 GPSLQVDVHT GAIAETILSF GITFAALLII IRGPRRLLAK TFLLALATIC FVVAGSKYTG
      181 PAMNPAIAFG WAYMYSSHNT WDHFYVVYWIN SFVGALSAAL VFRTIFPPPT PQQKKQKKA
//
LOCUS       BrSIP1-2       254 aa
DEFINITION  BrSIP1-2     254 aa
TITLE       BrSIP1-2
ORIGIN

        1 MGVVKSATGD MLMTFSWVVL SATFGLQTTE IISAAGLHGI TWAPLAITTF LIFVYVSIFT
       61 VVFGSASFNP TGNAAFYAAG IPGDTLFTLA IRLPAQAAGA AGGALAIMEF IPEKYKHMIS
      121 GPSLLVDVHT GAIAETILSF GITFAVLLII LKGPRRLLAK TLLLSLATIC FVVAGSKYTG
      181 PAMNPAIAFG WAYMTSSHNT WDHFYVVYWIS SFVGALSAAL IFRTIFPPSP SPPRPQKKQK
      241 KQKKQKKAEK QKKA
//
LOCUS       BrSIP1-3       243 aa
DEFINITION  BrSIP1-3     243 aa
TITLE       BrSIP1-3
ORIGIN

        1 MNAVRSALGD MVITFFWVIL SATFGLQTAA IVSAAGFHGI TWAPPLITTV VVFFSISVFT
       61 VIGNFLGGAS FNPCGNAAFY TAGVSADSLF SLAIRSPAQA LGAAAGAITI MEMIPEKYKT
      121 MIGGRPSFRV DAHSGAISEV ILSFCVTFLV LLIIILRGPRK LLAKTFLLAI ATVSVFIAGS
      181 TFTRPFMNPA IAFGWAYIHK SHNTWNHFYV YWFSSFTGAI LSAILFRSLF PPPLPVQKKQ
      241 KKA
//
LOCUS       BrSIP2-1       238 aa
DEFINITION  BrSIP2-1     238 aa
TITLE       BrSIP2-1
ORIGIN

        1 MGRISLVVSD LVLSFMWIWA GVLVNVLVHG VLGFSRKDTT GDIVRYLFSV ISMFVFAFLQ
       61 KLTKGGLYNP LTALASGVSG GFSSFIFSVV VRIPVEVLGS ILAVKHIIHV FPEIGKGPKL
      121 NVAIHHGALT EGILTFFIVM LSLGLTRKIP GSFFMKTWIG SIAKLTLHVL GADLTGGCMN
      181 PAAVMGWAYA RGEHITQEHL LVYWLGPVKA TLLAVWFFNV VFKPLTEEQQ EKPKAKSE
//
LOCUS       BrSIP2-2       234 aa
DEFINITION  BrSIP2-2     234 aa
TITLE       BrSIP2-2
ORIGIN
```

```
        1 MSRISIVVSD LVLSFMWIWS GVLVSILVHG VLGFSRNVTT GEIVGYTFSV ISMFIFAFLQ
       61 KLTKGGHYNP VAALASGGFG SFIFTIMVRV PAEVIGSILA VKHIIHVFPE IGKGPKLNVS
      121 IHQGALTEGV LTFFTVLISM ELSRKIPGSF FMKTWISSIA KLSLHVLGAD LTGGCMNPAA
      181 VMGWAYALGE HIAKEHLLVY WLGPVMATLL AVWFFNAVFK PLTKEQEKPK AKSD
//
LOCUS       BrSIP2-3       237 aa
DEFINITION  BrSIP2-3     237 aa
TITLE       BrSIP2-3
ORIGIN

        1 MGRIGIVVSD LVLSFMWTWA GVLVNILVHG VLGFSRKDTT GEIVRYLFSV ISMFVFAFLQ
       61 KLSKGGLYNP LTALAAGVTG GFSNFIFTVL VRIPVEVIGS ILGVKHIIHV FPEIGKGPKL
      121 NVAIHHGALT EGILTFFIVM LSLGLARKIP GSFFMKTWIG SIAKLTLHVL GADLTGGCMN
      181 PAAVMGWAYA RGEHITQEHL LVYWLGPIKA TLLAVWFFNV VFKPLTEEEE KPKAKTD
//
LOCUS       BrTIP1-1       251 aa
DEFINITION  BrTIP1-1     251 aa
TITLE       BrTIP1-1
ORIGIN

        1 MPIRNIAVGS PNEATRPDAL KAALAEFIST MIFVFAGSGS GMAFNKLTEN GATTPAGLVA
       61 ASLAHAFGLF VAVSVGANIS GGHVNPAVTF GAFVGGNITL LRGILYWIAQ LLGSVVACLL
      121 LKFATGGLVV PAFGLSAGVG VSNALVFEIV MTFGLVYTVY ATAVDPKNGS LGTIAPIAIG
      181 FIVGANILAG GAFSGASMNP AVAFGPAVVS WSWNNHWVYW AGPLVGGGLA GLIYEVFFIN
      241 TTHEQLPTTD Y
//
LOCUS       BrTIP1-2       253 aa
DEFINITION  BrTIP1-2     253 aa
TITLE       BrTIP1-2
ORIGIN

        1 MPIRNIAIGG VQEEVTHPSA LRAALAEFIS TLIFVFAGSG SGIAFNKLTD NGATTPSGLV
       61 AAALAHAFGL FVAVSVGANI SGGHVNPAVT FGAFVGGNIT LLRGILYWIA QLLGSVVACL
      121 LLKFATGGLA VPAFGLSAGV ESLNGFVFEI VMTFGLVYTV YATAVDPKNG SLGTIAPIAI
      181 GFIVGANILA GGASSGASMN PAVAFGPAVV SWTWTNHWIY WAGPLVGGGL AGLIYEFVFI
      241 NQNGHEQLPT TDY
//
LOCUS       BrTIP1-3       253 aa
DEFINITION  BrTIP1-3     253 aa
TITLE       BrTIP1-3
ORIGIN

        1 MPTRNIAIGG VQEEVTHPSA LRAALAEFIS TLIFVFAGSG SGIAFNKLTD NGATTPSGLV
       61 AAALAHAFGL FVAVSVGANI SGGHVNPAVT FGAFLGGNIT LLRGLLYWIA QLLGSVVACF
      121 LLQFATGGLA VPAFGLSAGV GTLNGLVFEI VMTFGLVYTV YATAIDPKNG SLGTIAPIAI
      181 GFIVGANILA GGAFSGASMN PAVAFGPAVV SWSWSNHWIY WVGPLVGGGL AGIIYDFVYI
      241 IENGHEQLPT TDY
//
LOCUS       BrTIP1-4       252 aa
DEFINITION  BrTIP1-4     252 aa
TITLE       BrTIP1-4
ORIGIN

        1 MAINRIAIGT PGEASGRDAI RAAFAEFFSM VIFVFAGQGS GMAYGKLTGD GPATPSGLVA
```

```
       61 ASLSHAFALF VAVSVGANVS GGHVNPAVTF GAFIGGNITL LRAILYWIAQ LLGAVVACLL
      121 LKVSTGGMET AAFSLSHGVT PWNAVVFEIV MTFGLVYTVY ATAVDPKKGD IGIIAPLAIG
      181 LIVGANILVG GAFDGASMNP AVSFGPAVVS WTWTNHWVYW VGPFIGAAIA AIVYDTIFID
      241 SNGHEPLPSS DF
//
LOCUS       BrTIP2-1       249 aa
DEFINITION  BrTIP2-1     249 aa
TITLE       BrTIP2-1
ORIGIN

        1 MAGIAFGSFD DSFSLASLKA YLAEFISTLL FVFAGVGSAI AYGKLTSDAA LDTSGLVAIA
       61 VCHGFALFVA VAIGANISGG HVNPAVTFGL ALGGQITLIT GVFYWIAQLL GSTAACFLLK
      121 FVTGGLAVPT HSVAAGLGAI EGVVMEIIIT FALVYTVYAT AADPKKGSLG TIAPLAIGLI
      181 VGANILAAGP FSGGSMNPAR SFGPAVASGG FSGHWVFWVG PLIGGGLAGL IYGNVFMSSS
      241 EHVPLASDF
//
LOCUS       BrTIP2-2       210 aa
DEFINITION  BrTIP2-2     210 aa
TITLE       BrTIP2-2
ORIGIN

        1 MYIAKLTSDA ALDTPGLVAI AVCHGFALFV AVAIGANISG GHVNPAVTFG LALGGQITLI
       61 TGVFYWIAQL LGSTAACFLL KFVTGGLAVP THSVAAGVGA IEGVVMEIII TFALVYTVYA
      121 TAADPKKGSL GTIAPLAIGL IVGANILAAG PFSGGSMNPA RSFGPAVAAG DFSGPWVYWV
      181 GPLIGGGLAG LVYGNVFMPS SEHVPLASEF
//
LOCUS       BrTIP2-3       248 aa
DEFINITION  BrTIP2-3     248 aa
TITLE       BrTIP2-3
ORIGIN

        1 MAGVAFGSFD DSFSLASLRA YLAEFISTLL FVFAGVGSAI AYAKLTSDAA LDTPGLVAIA
       61 VCHGFALFVA VAVGANISGG HVNPAVTFGL AVGGQITLIT GVFYWVAQLL GSTAACFLLK
      121 YVTGGLAVPT HSVAAGVGAI EGVVMEIIIT FALVYTVYAT AADPKKGSLG TIAPLAIGLI
      181 VGANILAAGP FSGGSMNPAR SFGPAVAAGD FSGHWVYWVG PLIGGGLAGI TYGNVFMTSE
      241 HVPLASDF
//
LOCUS       BrTIP2-4       250 aa
DEFINITION  BrTIP2-4     250 aa
TITLE       BrTIP2-4
ORIGIN

        1 MVKIAIGSLG DSFSVASLKA YLSEFIATLL FVFAGVGSAI AFGKLTSNAA LDPAGLVAVA
       61 VAHAFALFVG VSIAANISGG HLNPAVTLGL AVGGNITVIT GFFYWIAQCL GSIVACLLLA
      121 FVTNGESVPT HGVAAGLGAV EGIVMEIVVT FALVYTVYAT AADPKKGSLG TIAPIAIGFI
      181 VGANILAAGP FSGGSMNPAR SFGPAVVSGD FSQIWIYWVG PLVGGALAGL IYGDVFIGSY
      241 APAPTTESYP
//
LOCUS       BrTIP2-5       204 aa
DEFINITION  BrTIP2-5     204 aa
TITLE       BrTIP2-5
ORIGIN

        1 MPRALDPAGL VAIAVAHAFA LFVGVSIAAN ISGGHLNPAV TLGLAVGGNI TLITGFLYWI
```

```
      61 AQCLGSIVAC LLLVYVTNGE SVPTHGVGAG LGALEGIVME IVVTFALVYT VYATAADPKK
     121 GSLGTIAPIA IDFIVGANIL AAGPFSGGSM NPARSFGPAV VSGDLSQIWI YWVGPLVGGG
     181 LAGLIYGDVF IGSYQEVETR EIRV
//
LOCUS       BrTIP2-6       251 aa
DEFINITION  BrTIP2-6     251 aa
TITLE       BrTIP2-6
ORIGIN

       1 MVKIAVASLG DSFSVASLKA YLPEFIATLI FVFAGVGSAI AFGKITSDAA LDPAGLVAIA
      61 VAHAFALFVG VSVAANISGG HLNPAVTLGL AVGGNITLIT GFLYWVAQCL GSTVACLLLV
     121 FVTNGESVPT HGVGAGLGAV EAIVMEIIVT FALVYTVYAT AADPKKGSLG TIAPIAIGFI
     181 VGANILAAGP FSGGSMNPAR SFGPAIVSGD LSQIWIYWVG PLVGGALAGL IYGDVFIGSP
     241 YEAVETREIR V
//
LOCUS       BrTIP3-1       266 aa
DEFINITION  BrTIP3-1     266 aa
TITLE       BrTIP3-1
ORIGIN

       1 MATSARRAYG FGRADEATHP DSIRATLAEF LSTFVFVFAA EGSILSLDKL YWDHAAHAGT
      61 NTPGGLVLAA LAHAFALFAA VSAAANVSGG HANPAVTFGA LIGGRLSAIR AIYYWIAQLL
     121 GAILACLLLR LATNGMRPVG FRLASGVGAV NGLVLEIILT FGLVYVVYST MIDPKRGSLG
     181 VIGPLAIGLI VGANILVGGQ FSGASMNPAR AFGPALVGWR WDDHWIYWVG PFIGGALAAL
     241 IYEYMVIPTE PPTQHTHHQP LAPEDY
//
LOCUS       BrTIP3-2       265 aa
DEFINITION  BrTIP3-2     265 aa
TITLE       BrTIP3-2
ORIGIN

       1 MAASTVRTYG FGRADEATHP DSLRATLAEF LSTFVFVFAA EGSILSLDKL YWDHAAHVGT
      61 NTPGGLVLVA LAHAFALFAA VSAAINVSGG HVNPAVTFGA LIGGRISAIR AIYYWIAQLL
     121 GALLACLLLR LSTNGMRPVG FSLASGVKAH NGLVLEIILT FGLVYVVYST LIDPKRGSLG
     181 IIGPLAVGLI VGANILMGGP FSGASMNPAR AFGPALVGWR WDDHWIYWVG PFIGGALAAF
     241 IYEFMVIPTE PPAHHTHQPL APEDY
//
LOCUS       BrTIP3-3       267 aa
DEFINITION  BrTIP3-3     267 aa
TITLE       BrTIP3-3
ORIGIN

       1 MATYARRTYG FGRTDEATHP DSIRATLAEF LSTFVFVFAG EGSILSLDKL YWDTAAHTGI
      61 DTPGGLLLVA LAHALALFAA VSAAINVSGG HVNPAVTFAA LVGGRLSVIR AIYYWVAQLL
     121 GAILACLLLR LSTNGKRPIG FHVASGVSEL HGLLMEIILT FALVYVFYST VIDPKRGSIG
     181 IIAPLAIGLI VGANMLVGGP FDGASMNPAR AFGPSLVGWR WDNHWIYWVG PFIGGALAAL
     241 IYEYMIIPNV NEPPRHSVHQ PLAPEDY
//
LOCUS       BrTIP3-4       267 aa
DEFINITION  BrTIP3-4     267 aa
TITLE       BrTIP3-4
ORIGIN

       1 MATYARRTYG FGRADEASHP DSIRATLAEF VSTFVFVFAG EGSILALDKL YWDTAAHTGT
```

```
       61 DTPGGLVLVA LAHALALFAA ISAAINVSGG HVNPAVTFAA LVGGRLSVIR AIYYWIAQLL
      121 GAILACLLLR LATNGSRPIG FHVASGVSEL HGLLMEIILT FALVYVFYST VIDPKRGSIG
      181 IIAPLAIGLI VGANMLVGGP FEGASMNPAR AFGPSLVGWR WHNHWIYWVG PFIGGALAAL
      241 IYEYMIIPSV NEPPRHSTHQ PLAPEDY
//
LOCUS       BrTIP4-1       249 aa
DEFINITION  BrTIP4-1     249 aa
TITLE       BrTIP4-1
ORIGIN

        1 MKKIDLGNHR EAAQPDCIKA LIVEFITTFF FVFAGVGSAM ATDSLVGNTL VGLLAVAVAH
       61 ALVVAVMISA GHISGGHLNP AVTIGLLFGG HISVFRAFLY WIDQLLASSA ACFLLSYLTG
      121 GMGTPVHTLA SGISYTQGII WEIILTFSLL FTVYATMVDP KKGSLDGLGP LLTGFVVGAN
      181 ILAGGAFSGA SMNPARSFGP ALVSGNWTDH WVYWVGPLIG GGLAGFIYEN VLIDRSDAPL
      241 ADDEQPFLN
//
LOCUS       BrTIP5-1       255 aa
DEFINITION  BrTIP5-1     255 aa
TITLE       BrTIP5-1
ORIGIN

        1 MIPTTFSSKF QGAVSMNALR CYVSEFISTF FFVLAAVGSV MASRKLTAGD VTGPFSVLLP
       61 AIANAFALSS SVYISWNVSG GHVNPAVTFG MAVAGRISVP TAMFYWTSQM IASVMACLVL
      121 KVTVVEQHVP IYKIAGEMTG FGASVLEGVL AFVLVYTVFT ANDPRLGLPL AVGPIFIGFV
      181 AGANVLAAGP FSGGAMNPAC AFGSAMIYGS FKNQAVYWVG PLLGGATAAL VYDNMVVVPA
      241 AEDDRGSSTG DATGV
//
LOCUS       CcNIP1-1       235 aa
DEFINITION  CcNIP1-1     235 aa
TITLE       CcNIP1-1
ORIGIN

        1 QVIAEVIGTY FVVFAGCCSV VLNNAEETKG TITFPGVCVV WGLIVMIMVY SVGHVSGAHF
       61 NPAVTLSFAL YRHFPLKLVP LYFIAQVLGS FLASGTLYLL FHIDDKSYFG TRPAGPHSQS
      121 LVFEILTSFL LMFVVSAVAT DNRAIGKFAG IAVGMTIIVD VFIAGPVSGA SMNPARSLGP
      181 ALVMHIYTGF WIYMVGPFVG AIVGASVYNL IRFTEKPLRE LGSESIRFAT RSNSP
//
LOCUS       CcNIP1-2       260 aa
DEFINITION  CcNIP1-2     260 aa
TITLE       CcNIP1-2
ORIGIN

        1 MATKAEGIQE EEFSRMEDGV SSSPSRCDTL HNCCSNNVVT LVQKVIAELI GTYFVVFAGC
       61 GSVAVNKIYG SVTFPGICVT WGLIVMVMIY SVNHISGAHF NPAVTITLAI FRRFSYKEVP
      121 LYVIAQLLGS ILASGTLALM LDVTPKDYFG TVPVGSIAQS LVAEIIITFL LMFVVSAVAT
      181 DHRAVNEFAG VAVGMTIMLN VFIAGPVSGA SMNPARSIGP ALIKHVYKGL WIYVIGPIIG
      241 AIAGALAYNL LRSTEMPSSE
//
LOCUS       CcNIP1-3       271 aa
DEFINITION  CcNIP1-3     271 aa
TITLE       CcNIP1-3
ORIGIN

        1 MADYSTGTES HEVVVNVTKD PSKICERSDS FVSVHCLQKL VAETVGTYFL IFAGCGSVVV
```

```
      61 NKNNDNIVTL PGIAITWGLV VMVLVYSVGH ISGAHFNPAV TIAFTSIGRF PLKQVPAYVA
     121 AQLLGSTLAS GTLRLIFMGK HDQFSGTLPT GSYLQAFVFE FIITFLLMFV ICGVATDNRA
     181 IGELAGIAIG STILLNVMIG GPITGASMNP VRSLGPAFVH TQYRGIWIYL LAPIVGAVAG
     241 AWVYNVIRYT EKPLREITKT GSFLKGRADI K
//
LOCUS       CcNIP1-4       231 aa
DEFINITION  CcNIP1-4     231 aa
TITLE       CcNIP1-4
ORIGIN

       1 MQLVAETVGT YFLIFAGCGS VVVNKNNDNI VTLPGIAITW GLVVMVLVYS VGHISGAHFN
      61 PAVTIAFTSI GRFPLKQVPV YVIAQVAGST LASGSLRLIF NGKHDHFSGT LPTGSHLQSF
     121 VIEFIITFFL MFVVSGVATD NRAIGELAGI AVGSTVLLNV MFAGPVSGAS MNPARSIGPA
     181 FVHNEYHAIW IYLVAPTLGA VAGAWVYNTI RYTDKSLREI TKSASFLKSR E
//
LOCUS       CcNIP1-5       495 aa
DEFINITION  CcNIP1-5     495 aa
TITLE       CcNIP1-5
ORIGIN

       1 MSVMADNSTN NGSHQVVLNV NSDASKKCDS DNQDCVPLLQ KLVAEVVGTY FLIFAGCGSV
      61 VVNLDKEKVI TQPGISIVWG LTVMVLVYSV GHISGAHFNP AVTIAHASTK RFPLKQVPAY
     121 VIAQVVGSTL ASGTLRLIFN GRNDHFVGTL PAGSNLQSFV VEFIITFYLM FVISGVATDN
     181 RAIGELAGLA VGSTVLLNVM FTGPISGASM NPARSLGPAI VHNEYKGIWI YLVSPTLGAV
     241 AGTWVYNFIR YTNKPVREIT KSASFLKEVV GTYFLIFAGC CSVVVNLDNE KVVTLPGISI
     301 VWGLTVMVLA YSLGHISGAH FNPAVTLAHA STKRFPLKEV PAYIVAQVLG STLASGTLRL
     361 IFSGKNDHFA GTQPAGSDLQ AFVVEFIITF YLMFVISGVA TDNRAIGELA GLAVGSTVLL
     421 NVMFAGPITG ASMNPARSLG PAIVHHEYRG IWIYLVSPNL GAIAGTWAYN FIRYTDKPVR
     481 EITKSASFLK GVEVK
//
LOCUS       CcNIP2-1       297 aa
DEFINITION  CcNIP2-1     297 aa
TITLE       CcNIP2-1
ORIGIN

       1 MEGSNQNTFS FMANTLEPPS PSTLDPEASS SSSPAFSFAR LAECYPPGFS RKVFAEVIGT
      61 FLLVFVGSGS AGLSAIDASK VSKLGASLAG GLIVTVMIYS IGHISGAHMN PAVSLAFAAV
     121 RHFPWPQVPF YIAAQLTGAI SASYTLRELF QPSKEIGATS PAGSHIQALI MEMVSTFTMV
     181 FISMAVATDT NATGQLSGVA VGSSVCIASI VAGPISGGSM NPARTLGPAI ATSSYKGLWV
     241 YFVGPITGAV LAAWSYNVIR DTEHPGFPFS LSSLSFKIRQ GIGGNDQDAK NSHRCLV
//
LOCUS       CcNIP3-1       301 aa
DEFINITION  CcNIP3-1     301 aa
TITLE       CcNIP3-1
ORIGIN

       1 MENNEEIPST PATPGTPGAP LFGGFNNGSG RNKSNKSLLK SCRCFSVEEW SLEDGGLPAV
      61 SCSLPAPPPV PLARKVGAEF IGTFILMFAG TAAAIVNQKT NGSETLIGCA ATTGLAVMIV
     121 ILATGHISGA HLNPAVTISF AALKHFPWKH VPMYIGAQVL ASICAAFALK AVYHPFMSGG
     181 VTVPSGGYAQ AFALEFIIAF NLMFVVTAVA TDTRAVGELA GIAVGATVML NILIAGPVSG
     241 GSMNPVRTLG PAVAANNYKA IWVYLVAPIL GALGGAGTYT AVKLPEEDDD AKAKASISFR
     301 R
//
LOCUS       CcNIP3-2       303 aa
```

```
DEFINITION  CcNIP3-2     303 aa
TITLE       CcNIP3-2
ORIGIN

        1 MDSEEVPSVP STPATPGTPG APLFGGLKFD KTNGVVRKSS FLKSCKCFSV EEWTLEDGAM
       61 PRVSCSLPAP HIPLAKKVGA EFIGTFILMF AAIGTAIVNQ KTHGSETLIG CAAANGLAVM
      121 IIIFSTGHIS GAHLNPAVTI SFAALKHFPW KNVPVYIGTQ VLASISAAFA LKVVFHPFMS
      181 GGVTVPSVGY GQAFATEFIV SFILMFVVTA VATDTRAVGE LAGIAVGATV MLNILIAGPA
      241 TGSSMNPVRT LGPAIAANNY KGIWLYLLAP ILGTLCGAGA YTVVKLPQED TKPPSAPAGS
      301 FRR
//
LOCUS       CcNIP4-1       235 aa
DEFINITION  CcNIP4-1     235 aa
TITLE       CcNIP4-1
ORIGIN

        1 MVMAEVVGTF ILMFCVCGIT ASTQFQNGAV GLLEYAATAG LTVVVIIFCI GPISCAHVNP
       61 AVTIAFATIG QFPWFKVPVY IIAQTVGSMS ATYIGSLVYG IKSDAMMTMP LQGCNSAFWV
      121 EVIATFIIMF LIAALTSESQ SVGHLSGFVA GIAIGLGVLI TGPFSGGSMN PARSLGPAIL
      181 SWKFKDIWIY MLAPSGGAVA GAATFRFLRL RDQHSSTLSS PNINDVGRPI PFCSS
//
LOCUS       CcNIP5-1       288 aa
DEFINITION  CcNIP5-1     288 aa
TITLE       CcNIP5-1
ORIGIN

        1 MSAYKNTNLQ FGIQVKAMMA DSLSVNVDSS FKSEFSTEQK RISTQEAQYS PSNIQKAIAE
       61 VVGTYILIFA GCGAALVNEK LPITILGIAI VSGLALTVAI YSVGHVSGGH FNPAVTVALA
      121 AVRKVHWKLV PVYVLCQMMG ATLAPLTLKV LYHDKVDIGV TVTKYLSSTS DLEAIVWEFI
      181 ITFILMLTIC GVATDHRGSK DLTGVAIGIA IIINIIIAGP ITGASMNPAR SFGPAIVSGD
      241 YKHIWVYITS PTLGAVTASA LYKLLEVTKP TKPEPCHCNM CNHNHLPL
//
LOCUS       CcPIP1-1       287 aa
DEFINITION  CcPIP1-1     287 aa
TITLE       CcPIP1-1
ORIGIN

        1 MEGKDEDVRV GANRYGERQA IGTAAQTQDS RDYREAPPAP LFEAKELTSW SFFRAGIAEF
       61 VATFLFLYVT VLTVMGVAKS PSKCSTVGVQ GIAWSFGGMI FALVYCTAGI SGGHINPAVT
      121 FGLFLARKLS LTRTVFYIIM QCLGAICGAA VVKGFQSNQY ERLGGAANIL NKGYSKGDGL
      181 GAEIVGTFIL VYTVFSATDA KRNARDSHVP ILAPLPIGFA VFLVHLATIP ITGTGINPAR
      241 SLGAALVYNK DQAWDNHWIF WVGPFIGAAL AALYHQIVIR AIPFKSK
//
LOCUS       CcPIP1-2       287 aa
DEFINITION  CcPIP1-2     287 aa
TITLE       CcPIP1-2
ORIGIN

        1 MEGREEDVRV GANRYGERQP IGTAAQGQHA KDYREPPSAP FFEPREFSSW SFYRAGIAEF
       61 VATFLFLYIT VLTVMGVAKS KTKCSTVGVQ GIAWAFGGMI FALVYCTAGI SGGHINPAVT
      121 FGLFLARKLS LTRAIFYIIM QCLGAICGAA VVKGFQPHQY ERLGGGANTL SKGYTKGDGL
      181 GAEIVGTFVL VYTVFSATDA KRNARDSHVP ILAPLPIGFA VFLVHLATIP ITGTGINPAR
      241 SLGAAIVYNK DQAWDDHWIF WVGPFIGAAL AALYHQVVIR AIPFSSK
//
```

```
LOCUS       CcPIP1-3       286 aa
DEFINITION  CcPIP1-3     286 aa
TITLE       CcPIP1-3
ORIGIN

        1 MEREEDVKVG AQKFSERHAL GTGAGGDKDY REAPAAPLFE GGELKSWSFY RAGIAEFVAT
       61 FLFLYITILT VMGVNRSPNK CSSVGIQGIA WAFGGMIFAL VYCTAGISGG HINPAVTFGL
      121 FLARKLSLTR AVFYIVMQCL GAICGAAVVK GFEGNARYQL FKGGANFVSH GYTKGDGLGA
      181 EIVGTFLLVY TVFSATDAKR NARDSHVPIL APLPIGFAVF LVHLATIPIT GTGINPARSL
      241 GAAIVYNRDH AWDHHWIFWV GPFTGAALAA LYHQIVIRAI PFKTRG
//
LOCUS       CcPIP1-4       306 aa
DEFINITION  CcPIP1-4     306 aa
TITLE       CcPIP1-4
ORIGIN

        1 MEGKEEDVSL GANKFPERQP IGTAAQSQDD GKDYTEPPPA PLFEPSELTS WSFYRAGIAE
       61 FVATFLFLYI TILTVMGVNR ADSKCATVGI QGIAWAFGGM IFALVYCTAG ISGGHINPAV
      121 TFGLFLARKL SLTRAVFYIV MQVLGAIVGA GVVKGFEGKT FYGKHNGGAN FVAPGYTKGD
      181 GLGAEIVGTF ILVYTVFSAT DAKRNARDSH VPHMPSTLIN IMHANESITL LAPLPIGFAV
      241 FLVHLATIPI TGTGINPARS LGAAIIFNKD LGWDDHWIFW VGPFIGAALA ALYHQVVIRA
      301 IPFKSK
//
LOCUS       CcPIP1-5       287 aa
DEFINITION  CcPIP1-5     287 aa
TITLE       CcPIP1-5
ORIGIN

        1 MENKEEDVKV GANKFSERQP IGTAAQSDKD YKEPPPAPLF EPGELKSWSF YRAGIAEFVA
       61 TFLFLYITIL TVMGVNNSSS KCSSVGIQGI AWAFGGMIFA LVYCTAGISG GHINPAVTFG
      121 LFLARKLSLT RALFYIVMQC LGAICGAGVV KGFEGNGRYE MFKGGANFVA SGYTKGDGLG
      181 AEIVGTFILV YTVFSATDAK RNARDSHVPI LAPLPIGFAV FLVHLATIPI TGTGINPARS
      241 LGAAIIYNRD HAWDDQWIFW VGPFIGAALA AVYHQIIIRA IPFKTRA
//
LOCUS       CcPIP1-6       289 aa
DEFINITION  CcPIP1-6     289 aa
TITLE       CcPIP1-6
ORIGIN

        1 MEGKEQDVSL GANKFPERQP IGTAAQSQDD GKDYQEPAPA PLIDPSEFTS WSFYRAGIAE
       61 FVATFLFLYI TILTVMGVNR SPSKCQSVGI QGIAWAFGGM IFALVYCTAG ISGGHINPAV
      121 TFGLFLARKL SLPRAVFYIV MQVLGAICGA GVVKGFEGKT RYGFYKGGAN FVAPGYTKGD
      181 GLGAEIVGTF VLVYTVFSAT DAKRNARDSH VPILAPLPIG FAVFLVHLAT IPITGTGINP
      241 ARSLGASIIF NKDLGWDDHW IFWVGPFIGA ALAALYHQVV IRAIPFKSK
//
LOCUS       CcPIP2-1       289 aa
DEFINITION  CcPIP2-1     289 aa
TITLE       CcPIP2-1
ORIGIN

        1 MAKHDVEVAE RGSFSAKDYH DPPPAPLIDA EELTKWSFYR ALIAEFIATL LFLYITVLTV
       61 IGYKSQSDVK AGGDVCGGVG ILGIAWAFGG MIFILVYCTA GISGGHINPA VTFGLFLARK
      121 VSLIRAILYM VAQCLGAICG VGLVKAFQKA YYNRYGGGAN ELSAGYSTGV GLGAEIVGTF
      181 VLVYTVFSAT DPKRSARDSH VPVLAPLPIG FAVFMVHLAT IPVTGTGINP ARSLGAAVMY
```

```
      241 NQQKAWDDHW IFWVGPFIGA AIAAFYHQFI LRAGAAKALG SFRSNPSTL
//
LOCUS       CcPIP2-2       287 aa
DEFINITION  CcPIP2-2     287 aa
TITLE       CcPIP2-2
ORIGIN

        1 MAKDVEVTER GSFSGKDYHD PPPAPLIDAE ELTKWSFYRA LIAEFIATLL FLYITVLTVI
       61 GYKHQSDLKA GGDLCGGVGI LGIAWAFGGM IFILVYCTAG ISGGHINPAV TFGLFLARKV
      121 SLIRAIMYMV AQCLGAICGV GLVKAFQKSY FNKYGGGANS LADGYNKGTG LGAEIIGTFV
      181 LVYTVFSATD PKRNARDSHV PVLAPLPIGF AVFMVHLATI PVTGTGINPA RSLGAAVIYN
      241 QDKPWDDHWI FWVGPFIGAA IAAFYHQFIL RAGAVKALGS FRSNPNV
//
LOCUS       CcPIP2-3       285 aa
DEFINITION  CcPIP2-3     285 aa
TITLE       CcPIP2-3
ORIGIN

        1 MAKDVEVQEQ GEYSSKDYQD PPPAPLIDPE ELTKWSFWRA LIAEFIATLL FLYITVLTII
       61 GYKSQTDPSK KGTECDGVGI LGIAWSFGGM IFILVYCTAG ISGGHINPAV TFGLFLGRKV
      121 SLIRALLYMV AQCAGAICGA GLAKGFQTAY YDRYGGGANS VADGYNKGTA LGAEIIGTFV
      181 LVYTVFSATD PKRNARDSHV PVLAPLPIGF AVFMVHLATI PITGTGINPA RSFGAAVIYN
      241 EDKIWDDQWI FWVGPIVGAA VAAFYHQYIL RAAAIKALGS FRSNA
//
LOCUS       CcPIP2-4       288 aa
DEFINITION  CcPIP2-4     288 aa
TITLE       CcPIP2-4
ORIGIN

        1 MAKDVEQVTE QGGEYSAKDY TDPPPAPLID PEELTKWSLY RAAIAEFIAT LLFLYITVLT
       61 IIGYKRQTDT NIPGNTECDG VGILGIAWAF GGMIFILVYC TAGISGGHIN PAVTFGLFLG
      121 RKVSLVRALL YIIAQCAGAI SGAGLAKGFQ KSYYNRYGGG VNLVSDGYNK GTALGAEIIG
      181 TFVLVYTVFS ATDPKRNARD SHVPVLAPLP IGFAVFMVHL ATIPVTGTGI NPARSFGPAV
      241 IFNEDKAWDD QWIYWVGPFI GAAVAAFYHQ YILRAAAIKA LGSFRSNA
//
LOCUS       CcPIP2-5       285 aa
DEFINITION  CcPIP2-5     285 aa
TITLE       CcPIP2-5
ORIGIN

        1 MAKDMEGEGH VGLAHKDYQD PPPAPFFDPA ELRKWSFYRA LIAEFVATLL FLYITILTVI
       61 GYNHQTDSTA DLCNGVGILG IAWAFGGMIF VLVYCTAGIS GGHINPAVTF GLFLARKVSL
      121 VRAVAYMVAQ CLGAISGVGL VKGFQSSYYN RYKGGANTLS HGYSKGTGLG AEIIGTFLLV
      181 YTVFSATDPK RKARDSHVPV LAPLPIGFAV FMVHLATIPI TGTGINPARS LGPAVIYNNQ
      241 KAWDDQWIFW VGPFIGAAIA AIYHQYVLRA HAVKALGSFR SSTNL
//
LOCUS       CcPIP2-6       278 aa
DEFINITION  CcPIP2-6     278 aa
TITLE       CcPIP2-6
ORIGIN

        1 MSKEVSQEGE QRKDYVDPPP APLIDLAEIK LWSFYRALIA EFIATLLFLY VTVATVIGHK
       61 KQSGACEGVG LLGIAWAFGG MIFVLVYCTA GISGGHINPA VTFGLFLARK VSLLRALLYM
      121 VAQCLGAISG VGLVKAFMKH SYNSLGGGAN SVNSGYSKGS ALGAEIIGTF VLVYTVFSAT
```

```
      181 DPKRSARDSH VPVLAPLPIG FAVFMVHLAT IPITGTGINP ARSFGAAVIY NNAKVWDDHW
      241 IFWVGPFVGA LAAAAYHQYI LRAAAIKALG SFRSNPTN
//
LOCUS       CcSIP1-1       245 aa
DEFINITION  CcSIP1-1     245 aa
TITLE       CcSIP1-1
ORIGIN

        1 MVSAIKAAIG DLVVTFLWVF FSSMLGLATD AITTALDLKG VSYNGFDYAS AVIITSLIFI
       61 LVTIFTFVAN ALGGASFNPT GNASFYAAGV GTDTLLSMAL RFPAQALGSV GGVLAIMEVM
      121 PPKYKHLIGG PSLKVSLHTG AIAEGVLTFV ITFVVLLIIL RGPRSEAKKT WLMAMSTVAL
      181 ITVGSSYTGP AMNPAFAFGW AYFQNWHNTW DQFYVYWICP FIGAILASWL FRIVFPPPVV
      241 KQKKA
//
LOCUS       CcSIP1-2       247 aa
DEFINITION  CcSIP1-2     247 aa
TITLE       CcSIP1-2
ORIGIN

        1 MVGAIKAAIG DAVLTFMWVF CSSLLGIASS AITKALDLQH LSYNGFPYPS FLVTTTLVFA
       61 LVFLFTVIGN AFGGASFNPT GTASFYAVGL GSDTLFSMAL RFPAQAVGAA GGAMAIMEVI
      121 PAEYRHKIGG PSLKVDVHTG AVAEGVLTFL ITFAVLLIIL KGPRSNLLKT WLLATATVAL
      181 VMVGSAYTGP AMNPANAFGW AYMNNLHNTW DQFYVYWICP FTGAILAAWL FRAIFPPPPP
      241 EVKQKKA
//
LOCUS       CcSIP1-3       239 aa
DEFINITION  CcSIP1-3     239 aa
TITLE       CcSIP1-3
ORIGIN

        1 MGVIKGAIGD ATLTSIWVFI ISTLRIVATE VALLLGLQPF SLLGLFITTI LNTLYVLTIS
       61 FIGRVLGGAS FNPSTSVSFY TAGLRPDSSL ASMAVRFPAQ AAGGAVGAKA LLMVVPPQYK
      121 DMLKGPFLKV DLHTGAVAEG VLTFTHNLAI LFVMLRGPKN PFLKVYLLSV ATVALAIPGA
      181 GFTGPAMNPA NAFGWAFVNN RHNTWEQFYV YWICPFVGAS LAAFIYRVKF MSPIKQKKA
//
LOCUS       CcSIP2-1       236 aa
DEFINITION  CcSIP2-1     236 aa
TITLE       CcSIP2-1
ORIGIN

        1 MGRTKLLLSD FVLSFMWVWS GVLIRIFLFK YLSFADALLA ELLKTTLSVT NMFLFAFLVK
       61 VTRGGAYNPL TVLADAISGD FNNFLYCVGA RIPTQVIGSI VGVKLLIDTI PEVGQGPRLN
      121 VDIHQGALTE GLLTFGIVII SLGLAMKVRE NFFMKTWISS LSKLTLHILG SDFTGGCMNP
      181 ASVMGWAYAR GDHITKEHIL VYWLAPIEAT ILAVWTFKFL VQPVIKDKTA SKSKSD
//
LOCUS       CcTIP1-1       178 aa
DEFINITION  CcTIP1-1     178 aa
TITLE       CcTIP1-1
ORIGIN

        1 MAFRNTIVRR AQDAAHRDTW RATLSEFIST LIFVFAASGS SLAVNRLTVD KPASVAVIGL
       61 SGGVRAGKAV VLEIVMTFGL VFAVYATSVD PRSRRKDGLV NIAPIVIGFM VGANVLVAGP
      121 FDGASMNPAA AFGPALVTWT WKNHWVYWVG PLIGGGLAGF SYELIFVSHT RQRWRTTY
//
```

```
LOCUS       CcTIP1-2       250 aa
DEFINITION  CcTIP1-2     250 aa
TITLE       CcTIP1-2
ORIGIN

        1 MPIRNIAVGR PEEVTHPDTL KAALAEFIST LIFVFAGSGS GIAYNKLTDN GAATPAGLIS
       61 AAIAHAFALF VAVSVGANIS GGHVNPAVTF GAFVGGNITF LRGIAYVIAQ LLGSIVASLL
      121 LAFVTASSVP AFGLSAGVGV GNALVLEIVM TFGLVYTVYA TAVDPKKGSL GTIAPIAIGF
      181 IVGANILLGG AFDGASMNPA VSFGPAVVSW TWDNHWIYWV GPLIGGGLAG LIYEVVFISH
      241 THEQLPTTDY
//
LOCUS       CcTIP1-3       252 aa
DEFINITION  CcTIP1-3     252 aa
TITLE       CcTIP1-3
ORIGIN

        1 MPISRISIGN PSELGQADAL KAALAEFISM LIFVFAGEGS GMAYNKLTNN GSATPAGVVA
       61 ASLSHSFALF VAVSVGANIS GGHVNPAVTF GAFIGGHITL LRGILYWIAQ LLGSVVACLL
      121 LKFATGGLET SAFALSSGVG AANALVFEIV MTFGLVYTVY ATAVDPKKGD LGIIAPIAIG
      181 FIVGANILAG GAFDGASMNP AVSFGPAVVS GTWANHWVYW AGPLIGSAIA AVVYEIFFIT
      241 PSSYEQLPVT DY
//
LOCUS       CcTIP1-4       252 aa
DEFINITION  CcTIP1-4     252 aa
TITLE       CcTIP1-4
ORIGIN

        1 MPISKIAIGN STELSQADAL KAALAEFISM LIFVFAGEGS GMAYNKLTNN GSATAAGLVA
       61 ASLSHAFALF VAVSVGANIS GGHVNPAVTF GAFVGGHITL FRSILYWIAQ LLGSVVACLL
      121 LKFATGGLET CAFALSSGVG AANALVFEIV MTFGLVYTVY ATAVDPKKGE VGIIAPIAIG
      181 FIVGANILAG GAFDGASMNP AVSFGPAVVS WTWDNHWVYW AGPFIGSAIA ALVYEIFFIT
      241 PNTYEQLPVT DY
//
LOCUS       CcTIP1-5       252 aa
DEFINITION  CcTIP1-5     252 aa
TITLE       CcTIP1-5
ORIGIN

        1 MAVYRIAIGS PREASNPAAI RAAFAEFFSM LIFVFAGQGS GMAYSKLTGN GPATPSGLIV
       61 ASLSHAFGLF VAVSVGANIS GGHVNPAVTF GAFIGGNITL LRSILYWIAQ LLGSVVACIL
      121 LNFATGGMEI TAFSVSSGVS VWNALVFEIV MTFGLVYTVY ATAVDPKKGN VGVIAPIAIG
      181 SIVGANILVG GAFDGASMNP AVSFGPAMVS WSWTHHWIYW LGPFIGSATA AILYDNIFIG
      241 DDGHEPLSSS DF
//
LOCUS       CcTIP1-6       252 aa
DEFINITION  CcTIP1-6     252 aa
TITLE       CcTIP1-6
ORIGIN

        1 MAIYRIAIGS PGEAGQPDAI RAAFAEFFSM IIFVFAGEGS GMAYSKLTNN GPATPGGLIA
       61 ASLSHAFGLF VAVSVGANIS GGHVNPAVTF GAFLGGNITL LRSILYWIAQ LLGSVVACIL
      121 LKTATGGMET SGFSISPGVS VWNALVFEIV MTFGLVYTVY ATAVDPKRGN VGVIAPIAIG
      181 FIVGANILVG GAFDGASMNP AVSFGPAVVT WSWTHHWVYW VGPFIGAAIA AIIYDNIFIG
      241 DDGHEPLSSS DF
```

```
//
LOCUS       CcTIP2-1       223 aa
DEFINITION  CcTIP2-1     223 aa
TITLE       CcTIP2-1
ORIGIN

        1 MAGIAFGRFD DSFSLSSIKA YIAEFISTLL FVFAGVGSAI AYAKLTSDAA LDPAGLVAVA
       61 ICHGFALFVA VSVGANISGG HVNPAVTFGL ALGGQITILT GIFYVIAQLL GSIVACLLLK
      121 FITGFTTPIH SVAAGVGAGE GVVTEIIITF GLVYTVYATA ADPKKGSLGT IAPIAIGFIV
      181 VVSGDFHDNW IYWVGPLVGG GLAGLIYTYA FIPTQHAPLA TEF
//
LOCUS       CcTIP2-2       248 aa
DEFINITION  CcTIP2-2     248 aa
TITLE       CcTIP2-2
ORIGIN

        1 MAGIAFGRFD DSFSLSSIKA YIAEFISTLL FVFAGVGSAI AYGKLTSDAA LDPAGLVAVA
       61 ISHGFALFVA VSVGANISGG HVNPAVTFGL ALGGQITILT GIFYWIAQLL GSIVACFLLH
      121 YVTGGLATPI HSVASGVGAV QGVVTEIIIT FGLVYTVYAT AADPKKGSLG TIAPIAIGFI
      181 VGANILAAGP FSGGSMNPAR SFGPAVVSGD FHDNWIYWVG PLIGGGLAGL IYGNVFIRSD
      241 HAPLSSEF
//
LOCUS       CcTIP2-3       226 aa
DEFINITION  CcTIP2-3     226 aa
TITLE       CcTIP2-3
ORIGIN

        1 MVKIDLGSFS DSFSVASLKA YFAEFHATLI FVFAGVGSAI AYNELTSDAA LDATGLVAVA
       61 VAHALALFVG VAVAANISGG HLNPAVTFGL AIGGNITLLT GFLYWIAQLL GSIVASLLLS
      121 LITAKSIPTH GLAAGVNAFQ GVVFEIIVTF GLVYTVYATA ADPKKGSLGV IAPIAIGFIV
      181 AVVSGNFADN WIYWVGPLIG GGLAGLIYGD VFIGSHTYAP PSETYP
//
LOCUS       CcTIP3-1       256 aa
DEFINITION  CcTIP3-1     256 aa
TITLE       CcTIP3-1
ORIGIN

        1 MATRRYEFGR SDEANHPDTL RATLAEFIST CIFVFVGEGS ALALRQIYKE PGSSAGEVVV
       61 LALAHAFALF AAISASAHVS GGHVNPAVTF GALLGGRISL LKAIYYWVAQ LLASIVAALL
      121 LRLVTNNMRP EGFSVSVGVG AFHGFVLEIA LTFGLMYTVY ATAIDPKRGS VASIAPLAIG
      181 LVVGANILAG GPFDGACMNP ARAFGPALVG WRWDNHWIFW VGPLIGAALA ALLYEFVMVP
      241 IEPPHAHHQP LASEDY
//
LOCUS       CcTIP3-2       255 aa
DEFINITION  CcTIP3-2     255 aa
TITLE       CcTIP3-2
ORIGIN

        1 MATRRYAFGR ADEATHPDSM RATLAEFAST FIFVFAGEGS GLALVKIYHD SAFSAGELLA
       61 LSLAHAFALF AAVSASMHVS GGHVNPAVTF GALLGGRISV VRAVYYWIAQ LLGAIVAALV
      121 LRLVTNNMRP SGFHVSEGVG VGHMLILEII MTFGLMYTVY ATAIDPKRGA VSNIAPLAIG
      181 LIVGANILVG GPFDGACMNP ALAFGPSLVG WRWHNHWIFW VGPLIGAALA ALVYEYVVIP
      241 TEPPHQHQPL ASEDY
//
```

```
LOCUS       CcTIP4-1       207 aa
DEFINITION  CcTIP4-1     207 aa
TITLE       CcTIP4-1
ORIGIN

        1 MLVDKLGGDP LVALFAVALA HALVVAVMIS AAHISGGHLN PSVTLGLLVG GHITVFRSIL
       61 YWIDQLLASA AASYLLYYLS GGQAIPVHTL ASGVGYGQGV VWEIVLTFSL LFTVYATMVD
      121 PKKGALAGLG PTLVGFVVGA NILAGGAYSA ASMNPARSFG PALVAGNWTD HWVYWVGPLI
      181 GGALAGFIYE TFFIDRSHVP LPRDEES
//
LOCUS       CcTIP5-1       254 aa
DEFINITION  CcTIP5-1     254 aa
TITLE       CcTIP5-1
ORIGIN

        1 MAPSSVTVTS RFHESVTRNA LRSYFAEFIS TFFYVFLVVG SGMSSRKLMP DASLNPTSLV
       61 VVAIASAFAL SCVLYIAWDI SGGHVNPAVT FAMAVGGHVS VPTALFYWAA QLIASVMACL
      121 VLRVIVVGMH VPTYTIAEEM TGFGASVLEG LLTFVLVYTV YAARDPRRGP LTSIGTLAIG
      181 LMAGACVLAA GPFSGGSMNP ACAFGSAAIA GSFRNQAVYW VGPLIGATVA GLLYDNVLFR
      241 SYPADSTRGE GIGV
//
LOCUS       CcXIP2-1       280 aa
DEFINITION  CcXIP2-1     280 aa
TITLE       CcXIP2-1
ORIGIN

        1 MWKAALTELT ATASLMFTLT TSIIACLDSH EVDPKLLVPF AVFIIAFLFL VVMVPLTGGH
       61 MSPVFTFIAA LKGVVTLTRA LVYVLAQCIG SIIGFFILKC VIEPRLAYTY SLGGCAISGT
      121 GVNYGIKSQD ALLVEFTCTF LVLFVGVTLA FDKKRSTHLG LPMVCLVVAG AMALAVFVSI
      181 TVTGRAGYAG VGLNPARCLG PALLHGGSLW EGHWVFWVGP FLACLVYYAV SINLPKEGLV
      241 WVDGEYDVLK LALGSSASLY NPSVPNSLPE ERNAEFQVQV
//
LOCUS       CclNIP1-1       239 aa
DEFINITION  CclNIP1-1     239 aa
TITLE       CclNIP1-1
ORIGIN

        1 MAATKIDGIE EETNQLAKME EGSLTETNGH APSCLSHSLF IVYLLAQPMI AELIGTYFII
       61 FAGCGAVAVD KIYGKVTFPG VCVTWGLIVM VMIYSLAHIS GAHFNPGVTI ALAIFRQFKW
      121 RQVPLYIVAQ VVGSLLASGT LSLMLDVTPQ AYFGTVPVGS NAQSFVAEII ISFLLMFVIS
      181 GAVTDDRAIA MFDCRLDNSE ELLPISGASM NPARSIGPAI VKHKFRGIWL YIIGPVIGT
//
LOCUS       CclNIP1-2       250 aa
DEFINITION  CclNIP1-2     250 aa
TITLE       CclNIP1-2
ORIGIN

        1 MGDSQKAEEN VGFLASDLQA IAEVIGTYFL IFTGCGSVAV NKIYGSVTFP GICVVWGLIV
       61 MVMIYSVGHI SGAHFNPAVT ITFAIFRHFP RKQVPIYIVA QLLGSVLASG SLYLIFDVKD
      121 EAFFGTIPVG TNVQSFVLEI IISFLLMFVI SGVATDNRSI GELAGIAIGM TILLNVLVAG
      181 PVSGASMNPA RSLGPAIVMH QYKGLWVYIA GPIIGTILGG LAYNMIRFTD KPLREITKSG
      241 SFLKSFSRPP
//
LOCUS       CclNIP1-3       283 aa
```

```
DEFINITION  CclNIP1-3     283 aa
TITLE       CclNIP1-3
ORIGIN

        1 MADNSGTGSN GVCVTFKDCE DAIQNCPSSA PPPPPSCHTK DSVYFSVPFM QKLMAEILGT
       61 YFMIFAGCAA VVVNLNNEKI VSLPGISIVW GLVVMVLVYS LGHISGAHFN PSVTIAHATC
      121 KRFPWKQVPP YILCQVLGST LAAGTLRLLF QEKQDQFAGT LPAGSNIQAF VMEFIITFYL
      181 MFVISGVATD NRAIGELAGL AVGSTVLLNV MFAGPITGAS MNPARSLGPA IVSSQYKGLW
      241 IYIVAPPLGA TAGAWVYNMV RYTDKPLREI TKSASFLKGA GRS
//
LOCUS       CclNIP1-4       275 aa
DEFINITION  CclNIP1-4     275 aa
TITLE       CclNIP1-4
ORIGIN

        1 MASSPSVTVE FSPKHSLPTK QPVAEEAKAH CLKWLPSDSA SPSHFQKIVA ELIGTFILIF
       61 AGCGSILVNK IQSLTIVGIG LVWGLVLIAM IYTVGHISGA HFNPAVTLAF AATRKLPWKQ
      121 VPMYMLAQVL GATLASLTLR ILFHEQDNIQ PTVTQYKDTT SDLEAVAWEF IVTFILMFTI
      181 SGVATDHRAS KDVAGVVIGV TVLFNVVISG PITGASMNPA RSIGPAVVSG VYKNLWVYIV
      241 APIIGALAAA MVYSILRVPK PVAEKPEETK STINQ
//
LOCUS       CclNIP2-1       287 aa
DEFINITION  CclNIP2-1     287 aa
TITLE       CclNIP2-1
ORIGIN

        1 MASMDPNLNT NIDELVSVQS PPSEKPKLCL VWNEHYPPGF LRKVIAEIIA TYLLVFVTCG
       61 SAALSAYDEH RVSKLGASVA GGLIVTVMIY AVGHISGAHM NPAVTLAFAA VRHFPWKQVP
      121 IYAAAQLTGA VSASLTLRVL LHPIKHIGTT SPSGSDLQAL IMEIVVTFSM MFVTSAVATD
      181 TKAIGELAGI AVGSAVCITS VLAGPVSGGS MNPARTVGPA IASSFYKGIW VYLVGPVTGT
      241 FMGAWSYNMI RETDKPAHAI SPGSLSFKLR RLKNNEQAHK NDPLDAL
//
LOCUS       CclNIP2-2       204 aa
DEFINITION  CclNIP2-2     204 aa
TITLE       CclNIP2-2
ORIGIN

        1 MASMDPNLNT NIDELVSVQS PPSEKPKLCL VWNEHYPPGF LRKVIAEIIA TYLLVFVTCG
       61 SAALSAYDEH RVSKLGASVA GGLIVTVMIY AVGHISGAHM NPAVTLAFAA VRHFPWKQVP
      121 IYAAAQLTGA VSASLTLRVL LHPIKHIGTT SPSGSDLQAL IMEIVVTFSM MFVTSAVATD
      181 TKAIGELAGI AVGSAVCITS VLAG
//
LOCUS       CclNIP3-1       304 aa
DEFINITION  CclNIP3-1     304 aa
TITLE       CclNIP3-1
ORIGIN

        1 MDHEDVPSAP STPATPGTPG APLFGGFRGD HRGTTGRKSL LKSCKCFSVE EWAIEDGRLP
       61 SVSCSLPPPP VSLARKVGAE FVGTLILIFA GTATAIVNQK TQGSETLIGL AGSTGLAVMV
      121 VILSTGHISG AHLNPAVTIA FAALKHFPWK HVPVYIGAQI MASLCAAFAL KGIFHPIMGG
      181 GVTVPSAGYG EAFALEFIIS FNLMFVVTAV ATDTRAVGEL AGIAVGATVM LNILIAGPST
      241 GGSMNPVRTL GPAVAVNNYK AIWIYLTAPI LGALCGAGTY SAVKLPEEDT DALAKPATAS
      301 SFRR
//
```

```
LOCUS       CclNIP3-2       300 aa
DEFINITION  CclNIP3-2     300 aa
TITLE       CclNIP3-2
ORIGIN

        1 MAESEPGTPA VSTPATPGTP GGALMSAARV DSLSYERQAK SGFKCLPVTA PSSWGHTNSC
       61 SFPDIPVPNV SLTRKVGAEF VGTFILIFAA TAGPIVNQKY SGAETLIGNA ACAGLAVMII
      121 ILSTGHISGA HLNPSLTIAF AALRHFPWVQ VPAYIMAQVS ASICASFALK AVFHPFMSGG
      181 VTVPSVNTGQ AFALEFLITF NLLFVVTAVA TDTRAVGELA GIAVGATVML NILIAGPSSG
      241 GSMNPVRTLG PAVAAGNYEK LWIFLLAPTL GALAGATTYT VVKLRDNETD PPREARSFRR
//
LOCUS       CclNIP3-3       290 aa
DEFINITION  CclNIP3-3     290 aa
TITLE       CclNIP3-3
ORIGIN

        1 MKMNPCFDKQ SCSEISTCAS TSGQSGDDPE TGSNAMSIRN KGLLCIPHDI DLNPARMVIA
       61 ELVGTFILML CVCGIMASTV LTRGEVGLLE YAATAGLTII VLVYSIGPIS GAHVNPAVTI
      121 AFAVVGHFPL SKVPFYIMAQ TAGSVLGTYI GILVYGIKSN LMITRPAQHC VSAFWVELLA
      181 TSIIVFLAAS LACEAQCFGN LSGFVVGVAI GLAVLITGPV SGGSMNPARS LGPAIVSWNF
      241 SDIWIYIIGP TIGAVAGGFV YRFLRLRPRA CSPSTSPNTS LLSHSFMFVR
//
LOCUS       CclPIP1-1       286 aa
DEFINITION  CclPIP1-1     286 aa
TITLE       CclPIP1-1
ORIGIN

        1 MEGKEEDVRL GANKFAERQP IGTAAQTQDG KDYVEPPPAP LFEPGELKSW SFYRAGIAEF
       61 IATFLFLYIS VLTVMGVVKS NTKCSTVGIQ GIAWAFGGMI FVLVYCTAGI SGGHINPAVT
      121 LGLFLARKLS LTRALFYMVM QCLGAICGAG VVKGFMGAGQ YGRLAGGANV VAHGYTKGDG
      181 LGAEIIGTFV LVYTVFSATD AKRSARDSHV PILAPLPIGF AVFLVHLATI PITGTGINPA
      241 RSLGAAIIYN KDRGWDDHWI FWVGPFVGAG LAALYHQVVI RAIPFK
//
LOCUS       CclPIP1-2       286 aa
DEFINITION  CclPIP1-2     286 aa
TITLE       CclPIP1-2
ORIGIN

        1 MEGKEEDVRL GANKFAERQP IGTAAQTQDG KDYVEPPPAP LFEPGELKSW SFYRAGIAEF
       61 IATFLFLYIS VLTVMGVVKS NTKCSTVGIQ GIAWAFGGMI FVLVYCTAGI SGGHINPAVT
      121 LGLFLARKLS LTRALFYMVM QCLGAICGAG VVKGFMGAGQ YGRLAGGANV VAHGYTKGDG
      181 LGAEIIGTFV LVYTVFSATD AKRSARDSHV PILAPLPIGF AVFLVHLATI PITGTGINPA
      241 RSLGAAIIYN KDRGWDDHWI FWVGPFVGAG LAALYHQVVI RAIPFK
//
LOCUS       CclPIP1-3       288 aa
DEFINITION  CclPIP1-3     288 aa
TITLE       CclPIP1-3
ORIGIN

        1 MEGKEEDVRL GANKFTERQP IGTAAQSQDG KDYVEPPPAP LFEEEELTSW SFYRAGIAEF
       61 IATFLFLYIS VLTVMGVVKS NTKCSTVGIQ GIAWAFGGMI FALVYCTAGI SGGHINPAVT
      121 FGLFLARKLS LTRALFYMVM QCLGAICGAG VVKGFMGAGQ YGRLGGGANA VAHGYTKGDG
      181 LGAEIIGTFV LVYTVFSATD AKRSARDSHV PILAPLPIGF AVFLVHLATI PITGTGINPA
      241 RSLGAAIIYN KDHGWDDHWI FWVGPFIGAA LAALYHQVVI RAIPFKSS
```

```
//
LOCUS       CclPIP1-4       287 aa
DEFINITION  CclPIP1-4     287 aa
TITLE       CclPIP1-4
ORIGIN

        1 MEGKEEDVKL GANKFSERQP IGTSAQSDKD YKEPPPAPLF EPGELKSWSF YRAGIAEFMA
       61 TFLFLYITVL TVMGVSKSNT KCSTVGIQGI AWAFGGMIFA LVYCTAGISG GHINPAVTFG
      121 LLLARKLSLT RAVFYMVMQC LGAICGAGVV KGFEGSKNYE RLGGGANVVA HGYTKGDGLG
      181 AEIIGTFVLV YTVFSATDAK RNARDSHVPI LAPLPIGFAV FLVHLATIPI TGTGINPARS
      241 LGAAIIFNKD HAWDDHWIFW VGPFIGAALA AVYHQIVIRA IPFKSRA
//
LOCUS       CclPIP1-5       287 aa
DEFINITION  CclPIP1-5     287 aa
TITLE       CclPIP1-5
ORIGIN

        1 MEGKEEDVRV GANRYRESQP IGTAAQTQDV KDYTEPPPAP LFEPGELSSW SFYRAGIAEF
       61 VATFLFLYIT VLTVMGVSKS KSKCSTVGIQ GIAWAFGGMI FALVYCTAGI SGGHINPAVT
      121 FGLFLARKLS LTRAVFYMIM QCLGAICGAA VVKGFQKSQY EMLGGGANML SKGYSKGDGL
      181 GAEIVGTFVL VYTVFSATDA KRNARDSHVP ILAPLPIGFA VFLVHLATIP ITGTGINPAR
      241 SLGAALIYNK DQAWDDHWIF WAGPFIGAAL AALYHQIVIR AIPFKSK
//
LOCUS       CclPIP2-1       283 aa
DEFINITION  CclPIP2-1     283 aa
TITLE       CclPIP2-1
ORIGIN

        1 MSKEVNEEGQ THRHHHGKDY VDPPPAPLID MAELKLWSFY RALIAEFVAT LLFLYVSVAT
       61 VIGHKKQSDA CGGVGLLGIA WAFGGMIFVL VYCTAGISGG HINPAVTFGL FLARKVSLIR
      121 AVAYMVAQCL GAICGVGLVK AFMKHEYNSL GGGANTVASG YNKGSALGAE IIGTFVLVYT
      181 VFSATDPKRS ARDSHVPVLA PLPIGFAVFM VHLATIPITG TGINPARSFG AAVIYNNDKA
      241 WDDHWIFWVG PFVGALAAAA YHQYILRAAA IKALGSFRSN PSN
//
LOCUS       CclPIP2-2       204 aa
DEFINITION  CclPIP2-2     204 aa
TITLE       CclPIP2-2
ORIGIN

        1 MSKEVNEEGQ THRHHHGKDY VDPPPAPLID MAELKLWSFY RALIAEFVAT LLFLYVSVAT
       61 VIGHKKQSDA CGGVGLLGIA WAFGGMIFVL VYCTAGISGG HINPAVTFGL FLARKVSLIR
      121 AVAYMVAQCL GAICGVGLVK AFMKHEYNSL GGGANTVASG YNKGSALGAE IIGTFVLVYT
      181 VFSATDPKRS ARDSHVPVCI SSPP
//
LOCUS       CclPIP2-3       287 aa
DEFINITION  CclPIP2-3     287 aa
TITLE       CclPIP2-3
ORIGIN

        1 MAKDTGVAEH GSYSAKDYTD PPPEPLFDAV ELTKWSFYRA LIAEFIATLL FLYVTVLTVI
       61 GYKSQTDPAK NVDGCAGVGI LGIAWAFGGM IFVLVYCTAG ISGGHINPAV TFGLFLARKV
      121 SLVRAVMYMV AQCLGAISGV GLVKAFQKSY YTRYGGGANE LADGYSTGVG LAAEIIGTFV
      181 LVYTVFSATD PKRSARDSHV PVLAPLPIGF AVFMVHLATI PITGTGINPA RSLGAAVIYN
      241 KDKAWDDQWL FWVGPFIGAA IAAFYHQFIL RAGAVKALGS FRSQSRV
```

```
//
LOCUS       CclPIP2-4       206 aa
DEFINITION  CclPIP2-4     206 aa
TITLE       CclPIP2-4
ORIGIN

        1 MAKDTGVAEH GSYSAKDYTD PPPEPLFDAV ELTKWSFYRA LIAEFIATLL FLYVTVLTVI
       61 GYKSQTDPAK NVDGCAGVGI LGIAWAFGGM IFVLVYCTAG ISGGHINPAV TFGLFLARKV
      121 SLVRAVMYMV AQCLGAISGV GLVKAFQKSY YTRYGGGANE LADGYSTGVG LAAEIIGTFV
      181 LVYTVFSATD PKRSARDSHV PVSLTN
//
LOCUS       CclPIP2-5       287 aa
DEFINITION  CclPIP2-5     287 aa
TITLE       CclPIP2-5
ORIGIN

        1 MAKDIEVGGQ GEFHAKDYHD PPPAPLIDAE ELTKWSFYRA IIAEFIATLL FLYITVLTVI
       61 GYKSQTDANH GGDGCGGVGI LGIAWAFGGM IFVLVYCTAG ISGGHINPAV TFGLFLARKV
      121 SLVRAVMYMV AQCLGAICGC GLVKAFQKSY YTRYGGGANE LADGYSTGTG LGAEIIGTFV
      181 LVYTVFSATD PKRNARDSHV PVLAPLPIGF AVFMVHLATI PVTGTGINPA RSLGAAVIYK
      241 KDKAWDDQWI FWVGPFIGAA IAAFYHQFIL RASASKALGS FKSSSNI
//
LOCUS       CclPIP2-6       235 aa
DEFINITION  CclPIP2-6     235 aa
TITLE       CclPIP2-6
ORIGIN

        1 MAKDIEIGGQ GEFHAKDYHD PPPAPLIDAE ELTQWSFYRA TIAEFIATLL FLYITVLTVI
       61 GYKSQTDANH GGDGCGGVGI LGIAWAFGGM IFVLVYCTAG ISGGHINPAV TFGLFLARKL
      121 SLVRAVMYMV AQCLGAICGC GLVKAFQKSY YTRYGGGANE LADGYSTGTG LGAEIIGTFV
      181 LVYTVFSATD PKRNARDSHV PVLAPLPIGF AVFMVHLATI PVTGTGINPV RRELQ
//
LOCUS       CclPIP2-7       287 aa
DEFINITION  CclPIP2-7     287 aa
TITLE       CclPIP2-7
ORIGIN

        1 MAKDIEVGRE GEFHDKDYHD NPPAPLIGAE ELTRWSFYRA IIAEFIATLL FLYITVLTVI
       61 GHKSQTDAKH GGDGCGGVGI LGIAWAFGGM IFVLVYCTAG ISGGHINPAV TFGLFLARKV
      121 SLVRAVMYMV AQCLGAICGC GLVKAFQKSY YTRYGGGANE LADGYSTGAG LGAEIIGTFV
      181 LVYTVFSATD PKRKARDPHV PVLAPLSIGF AVFMVHLATI PVTGTGINPA RSFGPAVIYN
      241 KDKAWDDQWI FWVGPFIGAA IAAFYHQFIL RASAAKALGS RRSSPNI
//
LOCUS       CclPIP2-8       289 aa
DEFINITION  CclPIP2-8     289 aa
TITLE       CclPIP2-8
ORIGIN

        1 MGKDVEVAEQ QGGGGEFSAK DYHDPPPAPL IDFEELGKWS FYRAVIAEFI ATLLFLYVTV
       61 LTVIGYKSQT DPNLNTDQCG GVGILGIAWA FGGMIFILVY CTAGISGGHI NPAVTFGLFL
      121 GRKVSLIRAL LYMVAQCLGA ICGCGFVKAF QKSYYNRYGG GANELADGYN KGTGLGAEII
      181 GTFVLVYTVF SATDPKRSAR DSHVPVLAPL PIGFAVFMVH LATIPITGTG INPARSFGAA
      241 VIYNKEKAWD DQWIFWVGPF IGAFVAAFYH QYILRAAAIK ALGSFRSNA
//
```

```
LOCUS       CclPIP2-9       264 aa
DEFINITION  CclPIP2-9     264 aa
TITLE       CclPIP2-9
ORIGIN

        1 MGKDVEVAEQ QGGGGEFSAK DYHDPPPAPL IDFEELGKWS FYRAVIAEFI ATLLFLYVTV
       61 LTVIGYKSQT DPNLNTDQCG GVGILGIAWA FGGMIFILVY CTAGISGGHI NPAVTFGLFL
      121 GRKVSLIRAL LYMVAQCLGA ICGCGFVKAF QKSYYNRYGG GANELADGYN KGTGLGAEII
      181 GTFVLVYTVF SATDPKRSAR DSHVPVLAPL PIGFAVFMVH LATIPITGTG INPARSFGAA
      241 VIYNKEKAWD DQVLKFFHYT ITFS
//
LOCUS       CclSIP1-1       244 aa
DEFINITION  CclSIP1-1     244 aa
TITLE       CclSIP1-1
ORIGIN

        1 MGVIKAAIGD AVLTSLWVFN LPFLGVLTGI VSKFLGVEAL LPVTLLITTF LATINVLVFS
       61 LLGHVLGGAS FNPSTTIAFY AAGLKPDSSL LSMAVRFPAQ AAGGVAGAKA ILQVMPSQHR
      121 HRLKGPSLKV DLHAGAVAEG AITFVFCFAL LFIMLKGPKT LILQLWLLSV TTVGLVLTGS
      181 AYTGPSMNPA NAFGWAYVNN WHNTWDLFYV YWICPFFGAF LAAWVFKFLF PAPSTPTTKK
      241 QKKS
//
LOCUS       CclSIP1-2       244 aa
DEFINITION  CclSIP1-2     244 aa
TITLE       CclSIP1-2
ORIGIN

        1 MGPVKAAAGD LVLTFMWVFV SSTFGVLTTE IASALGVHGL VWPPMLITTA IVFVFVFIFN
       61 IIGDALGGAS FNPTGTAAFY AVGVGSDSLL SMALRFPAQA AGAVGGALAI MEYMPPKYKH
      121 MLGGPSLQVD LHTGAIAEGV LTFLITFAVL VIMLKGPQSN ILKTWLVAVA TVVLIVTGSA
      181 YTGPSMNPAN AFGWAYVNKW HDTWDQFYVY WICPFIGAVA ASMIFRVVFP PPAPARPAKK
      241 KKKN
//
LOCUS       CclSIP2-1       236 aa
DEFINITION  CclSIP2-1     236 aa
TITLE       CclSIP2-1
ORIGIN

        1 MAGIRLVVSD FILSFMWVWQ SVLIKIFVYK VLGLGHAPSG EVFKCGLSII SMFLFAFLGK
       61 VTKGGAYNPL TVLASGISGD FSNFLFTVGA RIPAQVIGSI TGVRFILDTF PQIGRGPSLN
      121 VGIHHGALTE GLLTFAIVTI SLGLARKIPG SFYMKTWISS VSKLALHILG SDMTGGCMNP
      181 ASVMGWAYAR GDHITKEHIL VYWLAPIQAT VIALWLFKLV VRPLAEEKKD SKSKSE
//
LOCUS       CclTIP1-1       252 aa
DEFINITION  CclTIP1-1     252 aa
TITLE       CclTIP1-1
ORIGIN

        1 MPIYRIAIGT PGEASHPDAL KAALAEFFSM IIFVFAGQGS GMAFSKLTDG GASTPAGLIS
       61 ASLAHAFALF VAVSVGANIS GGHVNPAVTF GAFVGGHITL LRSILYWIAQ LLGSVVACLL
      121 LKFSTGGLET SAFALSSGVS SWNAVVFEIV MTFGLVYTVY ATAVDPKKGN LGTIAPIAIG
      181 FIVGANILAG GAFDGASMNP AVSFGPAVVS WTWTNHWVYW LGPFIGAAIA AIVYDHIFID
      241 DNAHQPLPAN DF
//
```

```
LOCUS       CclTIP1-2       250 aa
DEFINITION  CclTIP1-2     250 aa
TITLE       CclTIP1-2
ORIGIN

        1 MPGIAIGNPA EIGQPDALRA AVAEFFSMVI FVFAGQGSGM AYSKLTDNGA ATPAGLVSAS
       61 IAHAFALFVA VSVGANISGG HVNPAVTFGA FVGGHITFVR SILYWIAQLL GSVVACLLLK
      121 FSTGGLETSA FALSSGVGAW NAVVFEIVMT FGLVYTVYAT AVDPKRGNIG IIAPIAIGFI
      181 VGANILAGGA FDGASMNPAV SFGPAVVSWT WDNHWVYWLG PFVGAAIAAI AYTVAFICPN
      241 THEQLPSTDF
//
LOCUS       CclTIP1-3       251 aa
DEFINITION  CclTIP1-3     251 aa
TITLE       CclTIP1-3
ORIGIN

        1 MPIRNIAVGH PREATHPDAL RAALAEFIST LIFVFAGEGS GMAFNKLTHN GANTPSGLVA
       61 ASVAHAFALF VAVAVGANIS GGHVNPAVTF GAFVGGNISL LRGILYWIAQ LLGSTVACLL
      121 LKFVTNGQTT SAFALSSGVG AWNAVVFEIV MTFGLVYTVY ATALDPKKGS LGTIAPIAIG
      181 FIVGANILAG GAFDGASMNP AVSFGPALVS WSWDNHWVYW VGPLIGGGLA GIVYEFFFIN
      241 QSHEQLPTTE Y
//
LOCUS       CclTIP2-1       247 aa
DEFINITION  CclTIP2-1     247 aa
TITLE       CclTIP2-1
ORIGIN

        1 MAIAFGRFDD SFSLGSFKAY LAEFISTLLF VFAGVGSAIA FNKMTADAAL DPSGLVAIAI
       61 CHGFALFVAV AIGANISGGH VNPAVTFGLA LGGQITILTG IFYWIAQLLG SIVASFLLKV
      121 VTGGLAVPTH NVAAGVGAIE GVVMEIIITF GLVYTVYATA ADPKKGSLGT IAPIAIGFIV
      181 GANILAAGPF SGGSMNPARS FGPAVASGNF QDNWIYWVGP LIGGGLAGLI YGNVFMHSEH
      241 APLSNDY
//
LOCUS       CclTIP2-2       204 aa
DEFINITION  CclTIP2-2     204 aa
TITLE       CclTIP2-2
ORIGIN

        1 MTADAALDPS GLVAIAICHG FALFVAVAIG ANISGGHVNP AVTFGLALGG QITILTGIFY
       61 WIAQLLGSIV ASFLLKVVTG GLAVPTHNVA AGVGAIEGVV MEIIITFGLV YTVYATAADP
      121 KKGSLGTIAP IAIGFIVGAN ILAAGPFSGG SMNPARSFGP AVASGNFQDN WIYWVGPLIG
      181 GGLAGLIYGN VFMHSEHAPL SNDY
//
LOCUS       CclTIP2-3       249 aa
DEFINITION  CclTIP2-3     249 aa
TITLE       CclTIP2-3
ORIGIN

        1 MVKITLGSLG DSFSVGSLKS YLAEFIATLL FVFAGVGSAI AYSKLTADAA LDPAGLVAVA
       61 VAHALALFVG VAIAANISGG HLNPAVTLGL AVGGNITILT GIFYWIAQCL GSIVACLLLQ
      121 FVTSGLSIPT HAVGAGLNAA EGLVMEIVIT FALVYTVYAT AADPKKGPLG TIAPIAIGFI
      181 VGANILAAGP FSGGSMNPAR SFGPAVVSGD FSQIWIYWVG PLIGGGLAGL VYGDIFIGSY
      241 TPASTEDYA
//
```

```
LOCUS       CclTIP3-1       258 aa
DEFINITION  CclTIP3-1     258 aa
TITLE       CclTIP3-1
ORIGIN

        1 MARRRYELGK AEEATQPETL KAIVAEFLAT FTFVFATEGS ILALGKYEDT HEVISTSRLV
       61 AIAGTHACSL FLAVSMSLNI SGGHVNPAVT FGALVAGRIS LLRASLYCLA QLFRSVVASL
      121 LLRLVIFAGP RPVGFPLAFD RDWLGLIIEA AMTFGLVYTF YATTMDPRKS HLTTIAPLAM
      181 GFFAGANILI RGPFHGASMN PARAFGPALV GWRWTKNWIY WVGPLVGGGL GAITYEYMVM
      241 PSETPVLHHC IQQSCTSS
//
LOCUS       CclTIP3-2       256 aa
DEFINITION  CclTIP3-2     256 aa
TITLE       CclTIP3-2
ORIGIN

        1 MPPRRYAIGR ADEAAHPDTM RAALAEFVST LIFVFAGEGS ILALDKISKA TTTSPSDLVV
       61 IALAHALALF AAVASSINTS GGHVNPAVTF GALLGGRISV VRAIYYWIAQ LLGAIVAALL
      121 LRFATNGMRP VGFYVASGVG EGHGLVLEIV MTFGLVYTVY ATVIDPKRGS LGIVGPLAIG
      181 FIVGANILVG GPFDGASMNP ARAFGPALVG WRWRNHWIYW VGPFIGAGLA ALIYEYIVIP
      241 TEPPIHTHQP LAPEDY
//
LOCUS       CclTIP4-1       247 aa
DEFINITION  CclTIP4-1     247 aa
TITLE       CclTIP4-1
ORIGIN

        1 MAKIALGSHR EATQPDCIKA LVVEFITTFL FVFVGVGSAM AADKLVGNAL VGLFFVAVAH
       61 AFVVAVMISA GHISGGHLNP AVTLGLLFGG HITFFRSILY WIDQLLASSA ACFLLSYLTG
      121 GLSTPVHTLA SGVGYFQGII WEIILTFSLL FTVYATIVDP KKGSIDGLGP LLTGFVVGAN
      181 ILAGGPFSGA SMNPARSFGP ALVSGDWTDH WVYWVGPLIG GGLAGFIYEQ FFIVRSHVPI
      241 PYEEARF
//
LOCUS       CclTIP5-1       254 aa
DEFINITION  CclTIP5-1     254 aa
TITLE       CclTIP5-1
ORIGIN

        1 MASTTLTARF NQSVTKEALR SYLAEFISTF FYVFAVVGSA MASKKLSPDA ASNTSSLVVA
       61 AIANVFALSS TVYIAANISG GHVNPAVTFA KAVSGHITVP TALFYWVSQM LASVMASLLL
      121 RVTAIGQNIP AYTIAQEMTG FGASLLEGVL TFALVYTVYA AGDPRGSPLG AIGPLAIGMM
      181 AGANVLATGP FSGGSMNPAC AFGSAVVAGS FKNQAVYWVG PLFGAAVAGL VYDIVVFPAQ
      241 VQDSIAGVSD GTAV
//
LOCUS       CclXIP1-1       235 aa
DEFINITION  CclXIP1-1     235 aa
TITLE       CclXIP1-1
ORIGIN

        1 VWRASLTEVL GTALLVFALD TIVISSIQTD TIIAILLLAT FPISGGHINP LVTFSAALIG
       61 HMTITRAAIY ILAQCVGGVF GALAPKAVVS TKIEHAFSLG GRTLIVVEQQ PNGPVELGLD
      121 TGVALWLEIF CSFVFLFASM WMAFDERQAK ALARVSVCII LGVVLGLLIF VSTTVTAQKG
      181 YGGAGRNPAR CLGPAFVRGG HLWDRHWVFW AGPATACVAF ALYIKLIPSQ HLHTH
//
```

```
LOCUS       CpNIP1-1       285 aa
DEFINITION  CpNIP1-1     285 aa
TITLE       CpNIP1-1
ORIGIN

        1 MAEISMNSAN NNRVVLNVND GDVGHNSLPA STSESLEKRD SALSFSVPFI QKLMAEFFGT
       61 FFLIFAGCAS VAVNDEYEKV VTLPGISIVW GLAVMVLVYS VGHISGAHFN PAVTIAFASC
      121 RRFPLKQVPA YILVQLLGST LAAGTLRLLF NGQHQVFTGT APSGSDMQSF GIEFIITFYL
      181 MFIISGVATD NRAIGELAGL AVGATVLLNV MFAGPISGAS MNPARSLGPA IVFSRYKGIW
      241 IYIFSPVLGA ISGAWVYNMV RYTDKPLREI TKSSSFLKSA RSCST
//
LOCUS       CpNIP1-2       190 aa
DEFINITION  CpNIP1-2     190 aa
TITLE       CpNIP1-2
ORIGIN

        1 MVMVYSVGHI SGAHFNPAVT ITFAIFRRFP FKQVPLYILA QLLGAILASY TLWIIFHVNE
       61 ESFFGTVPVG SDLQSLWIEI IISFILMFVI SGVATDNRAI GELAGIAVGM TIILNVFVAG
      121 PVSGASMNPA RSIAPAIVMH VYKGLWVYIV GPPIGTILGG AAYNLIRFTD KPLREITRTG
      181 SFLKSISRRN
//
LOCUS       CpNIP1-3       270 aa
DEFINITION  CpNIP1-3     270 aa
TITLE       CpNIP1-3
ORIGIN

        1 MASKNDSIEE VPPDVEEEGT ATSTNRLEPT SKNSSNLWAF TLAQKVIAEL VGTYFIIFSG
       61 CGAVAVNKIY GSVTFPGICV TWGLIVTVMI YTVGHVSGAH FNPAVTIAFT IFQKFPPSEV
      121 LFYIVAQFLG SILASGTLAL MFDITPNAYF GTTPVGSNGQ SLAIEIIITF LLTFVIFGAS
      181 IDERAIGQLG GIAVGMTVML NVFVAGPISG ASMNPARSLG PAFVKHEFKG LWIYVIGPVA
      241 GAIAGASAYS LVRAGDRPSE TLSFLTGSSK
//
LOCUS       CpNIP2-1       292 aa
DEFINITION  CpNIP2-1     292 aa
TITLE       CpNIP2-1
ORIGIN

        1 MAGTLTHPNL NNQTDINDLV SVESPISDRS SIWKSFEHHY PPCFLRKVAA EVIATYLLVF
       61 VTCGSAAISA IDESRVSKLA ASVAGGLIVT VMIYAVGHIS GAHMNPAVTL AFAALRHFPW
      121 KQVPFYAAAQ VTGAISAAFT LRVLLHPIKL IGTTSPAGSD IQALIMEIVV TFSMMFITSA
      181 VATDTKAVGE LAGIAVGSAV CITSILAGPV SGGSMNPARS IGPALASQYY KGIWVYLVGP
      241 VTGTLLGAYS YNLIRVKDEP VQAISPRSFS FKLRRMKSHE EQINMKDPLN SL
//
LOCUS       CpNIP3-1       310 aa
DEFINITION  CpNIP3-1     310 aa
TITLE       CpNIP3-1
ORIGIN

        1 MDQKEDVPSA PSTPATPGTP GAPLFGGFRR ERSSSGFFRS KSLINTCNCF TLRDWSLEDP
       61 SVLPPVTCTL PHPPVSLARK VGAEFIGTLI LIFAGTATAI VNQKSQGSET LIGLAASTGL
      121 AVMIVILSTG HISGAHLNPA VTISFAALHH FPWKHVPAYI AAQTVGSLCA AFALKIVFHP
      181 MMGGGVTVPS PSVGYAQAFA LEFIISFNLM FVVTAVATDT RAVGELAGIA VGATVMLNIL
      241 IAGPSTGASM NPVRTLGPAI AANNYKAIWV YLIAPILGAL SGAGIYTAVK LPEEDADTHE
      301 KPSTARSFRR
```

```
//
LOCUS       CpNIP3-2       298 aa
DEFINITION  CpNIP3-2     298 aa
TITLE       CpNIP3-2
ORIGIN

        1 MPESETGTPT ASAPATPGTP GGPLISGLRV DSLSYDRKSM ARCKCLPVTA PTWGQPHTCF
       61 IDFPAPDVSL TRKLGAEFVG TFILIFAATA APIVNQKYNG AETLIGNAAC SGLAVMIIIL
      121 STGHISGAHL NPSVTICFAA LRHFPWAQVP AYIAAQISAS ICASFTLKGV FHPFLSGGVT
      181 VPSVSLGQAF ALEFLISFIL LFVITAVATD TRAVGELAGI AVGATVMLNI LVAGPSSGGS
      241 MNPVRTLGPA VAAGNYKAIW VYLLAPILGG LVGAGTYTAV KLRDDEAEPP RQVRSFRR
//
LOCUS       CpNIP3-3       225 aa
DEFINITION  CpNIP3-3     225 aa
TITLE       CpNIP3-3
ORIGIN

        1 MVGTFILVLC VCGIIASQQL MRGEVGLMEY AATAGLTVVV VIFCIGPISG AHVNPAVTIA
       61 FAIFGHFSWS RVPFYILAQM LGSTLATWAG RSVYGVRADL MATRPVQGCF AAFWVEFFGT
      121 FIIMFLSAAL ICEAHTIGHL SGFVVGIAIG LAVLITGPVS GGSMNPARSL GPAIVSWDFD
      181 NIWIYMIAPV VGAIAGVLLY QFLRLKHRPC TATSSPSTVS YLVTP
//
LOCUS       CpPIP1-1       289 aa
DEFINITION  CpPIP1-1     289 aa
TITLE       CpPIP1-1
ORIGIN

        1 MEGKEEDVRL GANKYSERQP IGTAAQSQDG GKDYKEPPPA PLFEPGELTS WSFYRAGIAE
       61 FMATFLFLYI TVLTVMGVVK ESSKCSTVGI QGIAWAFGGM IFALVYCTAG ISGGHINPAV
      121 TFGLLLARKL SLTRAIFYMV MQCLGAICGA GVVKGFEGSA TFELKGGGAN VVNHGYTKGD
      181 GLGAEIVGTF VLVYTVLSAT DAKRNARDSH VPILAPLPIG FAVFLVHLAT IPITGTGINP
      241 ARSLGAAIIF NRDKAWDDHW IFWVGPFIGA ALAALYQQVV IRAIPFKSK
//
LOCUS       CpPIP1-2       286 aa
DEFINITION  CpPIP1-2     286 aa
TITLE       CpPIP1-2
ORIGIN

        1 MEGKEEDVKL GANKFSERQP IGTSAQTDKD YKEPPPAPLF EPGELHSWSF WRAGIAEFMA
       61 TFLFLYITVL TVMGVNRAPN KCASVGIQGI AWAFGGMIFA LVYCTAGISG GHINPAVTFG
      121 LFLARKLSLT RSIFYMVMQC LGAICGAGVV KGFQPRPYQM LGGGANMVNH GYTKGDGLGA
      181 EIVGTFVLVY TVFSATDAKR NARDSHVPIL APLPIGFAVF LVHLATIPIT GTGINPARSL
      241 GAAIIYNDDT AWDHHWVFWV GPFIGAALAA LYHQIVIRAI PFKTRG
//
LOCUS       CpPIP1-3       286 aa
DEFINITION  CpPIP1-3     286 aa
TITLE       CpPIP1-3
ORIGIN

        1 MEGKEEDVKL GANKFSERQP IGTAAQTDKD YKEPPPAPLF EPGELKSWSF YRAGIAEFIA
       61 TFLFLYITIL TVMGVKRSDS MCASVGIQGI AWAFGGMIFA LVYCTAGISG GHINPAVTFG
      121 LLLARKLSLT RAVFYMIMQC LGAICGAGVV KGFQPGPYQR LGGGANVVNH GYTKGDGLGA
      181 EIVGTFVLVY TVFSATDAKR NARDSHVPIL APLPIGFAVF LVHLATIPIT GTGINPARSL
      241 GAAIIFNTDH AWDDHWIFWV GPFIGAALAA VYHQIVIRAI PFKTRA
```

```
//
LOCUS       CpPIP1-4       287 aa
DEFINITION  CpPIP1-4     287 aa
TITLE       CpPIP1-4
ORIGIN

        1 MEGKEEDVRL GANRYRERQP IGTAAQTQDA KDYTEPPPAP LIEPGELFSW SFYRAGIAEF
       61 VATFLFLYIT VLTVMGVVKS PTKCSTVGIQ GIAWAFGGMI FALVYCTAGI SGGHINPAVT
      121 FGLLLARKLS LVRAVFYMIM QCLGAICGAG VVKAFEKTQY EMLGGGANTV GPAYSKTAGL
      181 GAEIVGTFVL VYTVFSATDA KRNARDSHVP ILAPLPIGFA VFLVHLATIP VTGTGINPAR
      241 SLGAALIYNK SQAWDDHWIF WVGPFIGAAL AALYHQIVIR AIPFRSK
//
LOCUS       CpPIP1-5       235 aa
DEFINITION  CpPIP1-5     235 aa
TITLE       CpPIP1-5
ORIGIN

        1 MEGKEEDVRL GANRYRERQP IGTAAQTQDA KDYTEPPPAP LIEPGELFSW SFYRAGIAEF
       61 VATFLFLYIT VLTVMGVVKS PTKCSTVGIQ GIAWAFGGMI FALVYCTAGI SGGHINPAVT
      121 FGLLLARKLS LVRAVFYMIM QCLGAICGAG VVKAFEKTQY EMLGGGANTV GPAYSKTAGL
      181 GAEIVGTFVL VYTVFSATDA KRNARDSHVP ILAPLPIGFA VFLVHLATIP VTGTX
//
LOCUS       CpPIP2-1       278 aa
DEFINITION  CpPIP2-1     278 aa
TITLE       CpPIP2-1
ORIGIN

        1 MTKDAGEHGS FSAKDYHDPP PAPLFDAVEL TKWSFYRALI AEFIATLLFL YVTVLTVIGH
       61 KSQHAADQCG GVGILGIAWA FGGMIFILVY CTAGGHINPA VTFGLFLARK VSLVRAVMYM
      121 VAQCLGAICG VGLVKAFQKS FYNRFGGGAN TLADGYNVGT GLGAEIIGTF VLVYTVFSAT
      181 DPKRSARDSH VPVLAPLPIG FAVFMVHLAT IPVTGTGINP ARSFGAAVIF NDKKAWDDHW
      241 IFWVGPFIGA AIAAFYHQFI LRAAAVKALG SFRSQSHV
//
LOCUS       CpPIP2-2       279 aa
DEFINITION  CpPIP2-2     279 aa
TITLE       CpPIP2-2
ORIGIN

        1 MSKEVSEEGQ SHGKDYVDPP PAPLIDLAEI KLWSFYRALI AEFIATLLFL YVTIATVIGH
       61 KKQAGPCDGV GILGIAWAFG GMIFILVYCT AGISGGHINP AVTFGLFLAR KVSLIRAVAY
      121 MVAQCLGAIC GVGLVKAFMK NYYNRLGGGA NTVATGYNTG TALGAEIIGT FVLVYTVFSA
      181 TDPKRSARDS HVPVLAPLPI GFAVFMVHLA TIPITGTGIN PARSFGAAVI WNNKKGWDDH
      241 WIFWVGPFVG ALAAAAYHQY ILRAAAIKAL GSFRSNPTN
//
LOCUS       CpPIP2-3       242 aa
DEFINITION  CpPIP2-3     242 aa
TITLE       CpPIP2-3
ORIGIN

        1 MTKDVEVAEQ GEFSAKDYHD PPPAPLIDVE ELTKWSFYRA LIAEFVATLL FLYVTVLTVI
       61 GHKSQTDLTK AGTDVCGGVG LLGIAWAFGG MIFILVYCTA GISGGHINPA VTLGLFLARK
      121 VSLIRAIMYM VAQCLGAICG VGLVKAFQSS FYNRYGGGAN SLNGGYNKGT GLGAEIIGTF
      181 VLVYTVFAAT DPKRNARDSH VPVWAPLPIG FAVFMVHLAT IPITGTGINP ARSFGACCDF
      241 QR
```

```
//
LOCUS       CpPIP2-4       285 aa
DEFINITION  CpPIP2-4     285 aa
TITLE       CpPIP2-4
ORIGIN

        1 MAKDIEVGGE FQAKDYHDPP PAPLIDPEEL TKWSFYRALT AEFVATLLFL YITVLTVIGY
       61 KSQTDKNQGG DDRGGVGILG IAWAFGGMIF ILVYCTAGIS GGHINPAVTF GLFLARKVSL
      121 VRAVLYMVAQ CLGAICGCGL VKAFQKACYN RYGGGANQLA LGYSTGTGLG AEIIGTFVLV
      181 YTVFSATDPK RNARDSHVPV LAPLPIGFAV FMVHLATIPV TGTGINPARS FGAAVIFNQD
      241 KPWDDHWIFW VGPFIGAAIA AFYHQFVLRA SGSKSLGSLR SSSNI
//
LOCUS       CpPIP2-5       280 aa
DEFINITION  CpPIP2-5     280 aa
TITLE       CpPIP2-5
ORIGIN

        1 MSGGEGNEQT VGAEIRGRDY EDPPPASLID MEELKKWSFY RAVIAEFVAT FLFLYVGIAT
       61 VIGNKKQIHP CDGVGLLGIS WSFGGMIFIL VYCTAGISGG HINPAVTLGL FAARKMSLIR
      121 AAAYMVSQCG GAICGVGLVK LFMTRSYNMH GGGANSVAPG FSTTTGLGAE IIGSFVLVYT
      181 VFSATDPKRS ARESFIPVLA PLPIGLAVFM VHLATIPITG TGINPARSLG AAVVYNNQQV
      241 WDEQWIFWVG PFLGALAAAA YHEYVLRAAA VKALLSFRPH
//
LOCUS       CpSIP1-1       246 aa
DEFINITION  CpSIP1-1     246 aa
TITLE       CpSIP1-1
ORIGIN

        1 MGVIRSAIGD SVLTSMWVFN LPVIGLLAGR ASDFLRTHYI SLPFTGLFIT ILLATINVLL
       61 FTLLGTLLGG ASFNPSTTVS FHAAGLTKPG SSLISMAVRL PAQAAGGAIG AMGIWQVMPV
      121 GWLKGGPSLK VDWHTGALAE GLLAFAHCLS VLVVVVRGPR SVFVKVLLLA MVTTGLVRVG
      181 SGYTGPSLNP ANAFGWAYVK NWHNSLELYY VYWVGPLVGA TMAAWVFRVL FAPSLVKKKK
      241 KKKKRE
//
LOCUS       CpSIP2-1       236 aa
DEFINITION  CpSIP2-1     236 aa
TITLE       CpSIP2-1
ORIGIN

        1 MAKIRLLISD FIVSFMWVWS GVLIKIFVHR VLGLGHEPRA EIIRGAMAIV NMFFFAFLGK
       61 VGKGASYNPL TVLAPAVSGD FSSFLFSLGC RIPVQVIGSI VGVRFILETI PEAGLGPRLK
      121 VDIHQGALTE GFLTFAIVMI SLGLAAKIPG SFFMKTWISS VSKLALHILG SDLTGGCMNP
      181 ASVMGWAFAR GDHITKEHIF VYWLAPVEAT LLAVWTFKLV TKSLTEDKAK LKAKSE
//
LOCUS       CpTIP1-1       252 aa
DEFINITION  CpTIP1-1     252 aa
TITLE       CpTIP1-1
ORIGIN

        1 MPISQIAIGS PAEIARPDAL KAALAEFISM LIFVFAGEGS GMAFNKLTNY GSATPAGLIS
       61 ASLAHAFALF VAVSVGANIS GGHVNPAVTF GAFVGGHISL FRSVLYWIAQ CLGSVLACLL
      121 LKFSTGGLET SAFALSSGVG ELNALVFEIV MTFGLVYTVY ATAIDPKRGN IGIIAPIAIG
      181 FIVGANILAG GAFDGASMNP AVSFGPAVVS WTWNSHWVYW VGPFAGAGIA ALVYEIIFIG
      241 SSTHEQLASA DF
```

```
//
LOCUS       CpTIP1-2       324 aa
DEFINITION  CpTIP1-2     324 aa
TITLE       CpTIP1-2
ORIGIN

        1 MGSPSAHLIV IFFDPQPVTG YPGSSTSSLS GYKSKSQELR SGDQYLQNHL DPCPYQDTSL
       61 PLTHPSFHLN QTNMPIRNIA IGRPEEATHP DALKAALAEF ISMLIFVFAG EGSGMAFNKL
      121 TNNGAATPAG LIAAAIAHAF ALFVAVAVGA NISGGHVNPA VTFGAFVGGN ISLLRGILYW
      181 IAQLLGSVAA CALLKFATGG LTTSAFALSS GVGVWNAFVF EIVMTFGLVY TVYATAVDPK
      241 KGSLGTIAPI AIGFIVGANI LAGGAFDGAS MNPAVSFGPA VVSWSWDNHW VYWAGPLIGG
      301 GLAGLIYDFF FISHSHEQLP TADY
//
LOCUS       CpTIP1-3       240 aa
DEFINITION  CpTIP1-3     240 aa
TITLE       CpTIP1-3
ORIGIN

        1 MPIYRVAIGA PRELSHPSAI RAALAEFFSM VIFVFAGEGS GMAFDKLTNN GSSTPAGLVA
       61 ASLAHAFALF VAVSVGANIS GGHVNPAVTF GAFLGGNITF FRSILYWIAQ LLGSVVACLL
      121 LKFATGNMET AAFGLSSGVS PMNALVFEIV MTFGLVYTVY ATAVDPKKGN LGTIAPIAIG
      181 FIVASMNPAV SFGPAVVSWT WTHHWVYWVG PFIGAAIAAI VYDNIFIGND SHEPLPTNDF
//
LOCUS       CpTIP2-1       249 aa
DEFINITION  CpTIP2-1     249 aa
TITLE       CpTIP2-1
ORIGIN

        1 MVKIALGSFG DSFSVGSLKA YLSEFIATLL FVFAGVGSAI AFGKVTSDGA LDPAGLVAIA
       61 VAHAFALFVG VAIAANISGG HLNPAVTLGL AIGGNITLLT GLFYWIAQCL GSIVACGLLK
      121 FVTDLSVPTH SVGSGMSVLE GVVMEIVITF ALVYTVYATA ADPKRGSLGI IAPIAIGFIV
      181 GANILAAGPF SGGSMNPARS FGPAVVSGDF TDNWVYWLGP LVGGALAGLV YGDIFIGSYS
      241 PVSASQDYP
//
LOCUS       CpTIP3-1       263 aa
DEFINITION  CpTIP3-1     263 aa
TITLE       CpTIP3-1
ORIGIN

        1 MPVRRYAFGR VEEVTHPDSI RATLAEFLST LVFVFAGEGS VLALDKLYRE TGGDVSRDPS
       61 GLVLIALAHA LSLFSAVSAS INISGGHVNP AVTFGALLGG RISVLRAFYY WLAQLLGAIV
      121 ACLLLRLVTA GMRPVGFRVA SGVGELNGLV LEMVLTFGLM YTVYATTIDP KRGSIGIIGP
      181 LAIGLIVGGN ILVGGPFDGG SMNPARAFGP ALVGWRWRNH WIYWVGPFVG GGLAALVYEF
      241 MVIPSTTEPP LITGHQPLAP EDY
//
LOCUS       CpTIP4-1       247 aa
DEFINITION  CpTIP4-1     247 aa
TITLE       CpTIP4-1
ORIGIN

        1 MAKIALGTRH EVTKPDCIRA LVVEFITTFL FVFTGVGSAM AADRLVGNGL LGLFAVAVAH
       61 ALVVAVMISA GHISGGHLNP AVTLGLLFGG HITLVRSILY WIDQLLASSI ACILLKYLTG
      121 GLDTPIHTLA SGVGYGQGVV WEIVLTFSLL FTVYATIVDP KKGSLDGLGP MLTGFVVGAN
      181 ILAGGAFSGA SMNPARSFGP ALVSWDWTDH WVYWVGPLMG GGLAGFMYEN FFIVRSHVLV
```

```
      241 SHQDDSC
//
LOCUS       CpTIP5-1       254 aa
DEFINITION  CpTIP5-1     254 aa
TITLE       CpTIP5-1
ORIGIN

        1 MAPASLTSRF GQSITKNAFR SYLAEFISTF FYVLTVVGSS MSARKLMGSG AEDPSGLVIV
       61 AIANSLGLSY TVYVASNISG GHVNPAVTFA RAVGGHVSVP TALFYWVSQM LASVMASLIL
      121 RVMTVAQHVP TYAIAEQMTG FGASVLEGVL TFALVYTIYA AGDPRRGQMG AIGPLVIGMA
      181 AGANFLAAGP FSGGSMNPAC AFGSAVVAGS FKNQAVYWVG PLIGATIGGL LYDNVVFPPE
      241 AIDSLTGISE RPVV
//
LOCUS       CpXIP1-1       322 aa
DEFINITION  CpXIP1-1     322 aa
TITLE       CpXIP1-1
ORIGIN

        1 MESVIVSSDR STHSALSTSI ENYDKTSPTR ISKNNSFLAS IGAHEFFSQE MWGAAITELV
       61 GTTCLLFTLT ISIVACLNFH EVESKLLVPI VVFVILFFFL MATIPISGGH MNPVFTFIAT
      121 LKGIITITRA AFYFLAQCLG SIISFIIIKS VMNHDSATKF SLGGCSIKGH GSTGLHLGVA
      181 LMLEFSCTFL VLFVAVNVAF DKRRSKELGV SKVCAQIAGA MALAVFVSIT VTGQTAYAGA
      241 GLNPAKCFGA AILQGGLLWK GHWVFWVGSF FACIVYYGFS LTLPKQGLDW VEGEYDAMRL
      301 AKACWGTKDF PNSSLQEKGD GP
//
LOCUS       CpXIP1-2       298 aa
DEFINITION  CpXIP1-2     298 aa
TITLE       CpXIP1-2
ORIGIN

        1 MARNGGAIVV EDEENPYSAT RIQPVASTPM AQQRNTEKGK KKTPTTLTGV WRASLAELLG
       61 TAVLVFAMDT IVISSYETQT KTPHLIMSFL VAITVTILLL ATSPISGGHI NPIVTVAAVL
      121 TGLISVSRAI VYILAQCIGG ILGALALKAV VNSTIQQTFS LGGCTLTVVV PGRHGPVVIG
      181 LETGQALWLE IICTFVFLFA SIWVAFDYRQ AKALGRFMVC LVIGVVVGLI VFVSTTVTAT
      241 KGYAGVGMNP ARCLGPALIR GGHLWSGHWV FWAGPVIACV AFALYIKMIP REHFHGGD
//
LOCUS       CsNIP1-1       282 aa
DEFINITION  CsNIP1-1     282 aa
TITLE       CsNIP1-1
ORIGIN

        1 MASSPSVTVE FSPKHTLPTK QPVAEEAKAH CLKWLPSDSA SPSHFQKIVA ELIGTFILIF
       61 AGCGSILVNK IQSLTIVGIG LVWGLVLIAM IYTVGHISGA HFNPAVTLAF AATRKLPWKQ
      121 VPMYMLAQVL GATLASLTLR ILFHEQDNIQ PTVTQYKDTT SDLEAVAWEF IVTFILMFTI
      181 SGVATDHRAS KDVAGVVIGV TVLFNVVISG PITGASMNPA RSIGPAVVSG VYKNLWVYIV
      241 APIIGALAAA MVYSILRVPK PVAEKPEETK STINQLYPHA DP
//
LOCUS       CsNIP1-2       284 aa
DEFINITION  CsNIP1-2     284 aa
TITLE       CsNIP1-2
ORIGIN

        1 MADNSGTGSN GVCVTFKDCE DAIQNCPSSA PPPPPPSCDT KDSVYFSVPF MQKLMAEILG
       61 TYFMIFAGCA SVVVNLNNEK IVSLPGISIV WGLVVMVLVY SLGHISGAHF NPSVTIAHAT
```

```
      121 CKRFPWKQVP PYILCQVLGS TLAAGTLRLL FQEKQDQFAG TLPAGSNIQA FVMEFIITFY
      181 LMFVISGVAT DNRAIGELAG LAVGSTVLLN VMFAGPITGA SMNPARSLGP AIVSSQYKGL
      241 WIYIVAPPLG ATAGAWVYNM VRYTDKPLRE ITKSASFLKG AGRS
//
LOCUS       CsNIP1-3       280 aa
DEFINITION  CsNIP1-3     280 aa
TITLE       CsNIP1-3
ORIGIN

        1 MSTPDIIQVE EGNEPCTTPA STKCSKISDA RNSSPSPVLQ LLQKAIAEVI GTYFLIFTGC
       61 GSVAVNKIYG SVTFPGICVV WGLIVMVMIY SVGHISGAHF NPAVTITFAI FRHFPRKQAS
      121 FSYVPIYIVA QLLGSVLASG SLYLIFDVKD EAFFGTIPVG TNVQSFVLEI IISFLLMFVI
      181 SGVATDNRSI GELAGIAIGM TILLNVLVAG PVSGASMNPA RSLGPAIVMH QYKGLWVYIA
      241 GPIIGTILGG LAYNMIRFTD KPLREITKSG SFLKSFSRPP
//
LOCUS       CsNIP1-4       262 aa
DEFINITION  CsNIP1-4     262 aa
TITLE       CsNIP1-4
ORIGIN

        1 MAATKIDGIE EETNQLAKME EGRLTETNGH APSCLSHSFM IAELIGTYFI IFAGCGAVAV
       61 DKIYGKVTFP GVCVTWGLIV MVMIYSLAHI SGAHFNPAVT IALAIFRQFK RRQVPLYIVA
      121 QVVGSLLASG TLSLMLDVTP QAYFGTVPVG SNAQSFVAEI IISFLLMFVI SGAVTDDRAI
      181 GQFGGVAVGM TIMLNVFVAG PISGASMNPA RSIGPAIVKH KFRGIWLYII GPVIGTVTGG
      241 FAYNLIKIHR QANQLHINSS VV
//
LOCUS       CsNIP2-1       222 aa
DEFINITION  CsNIP2-1     222 aa
TITLE       CsNIP2-1
ORIGIN

        1 MASIDPNLNT NIDELVSVQS PPSEKPKLCL VWNEHYPPGF LRKVIAEIIA TYLLVFVTCG
       61 SAALSAYDEH RVSKLGASVA GGLIVTVMIY AVGHISGAHM NPAVTLAFAA VRHFPWKQIG
      121 ELAGIAVGSA VCITSVLAGP VSGGSMNPAR TVGPAIASSF YKGIWVYLVG PVTGTFMGAW
      181 SYNMIRETDK PAHAISPGSL SFKLRRLKSN EQAHKNDPLD AL
//
LOCUS       CsNIP3-1       304 aa
DEFINITION  CsNIP3-1     304 aa
TITLE       CsNIP3-1
ORIGIN

        1 MDHEDVPSAP STPATPGTPG APLFGGFRGD HRGTNGRKSL LKSCKCFSVE EWAIEDGRLP
       61 SVSCSLPPPP VSLARKVGAE FVGTLILIFA GTATAIVNQK TQGSETLIGL AGSTGLAVMV
      121 VILSTGHISG AHLNPAVTIA FAALKHFPWK HVPVYIGAQI MASLCAAFAL KGIFHPIMGG
      181 GVTVPSAGYG EAFALEFIIS FNLMFVVTAV ATDTRAVGEL AGIAVGATVM LNILIAGPST
      241 GGSMNPVRTL GPAVAVNNYK AIWIYLTAPI LGALCGAGTY SAVKLPEEDT DALAKPATAS
      301 SFRR
//
LOCUS       CsNIP3-2       300 aa
DEFINITION  CsNIP3-2     300 aa
TITLE       CsNIP3-2
ORIGIN

        1 MAESEPGTPA VSTPATPGTP GGALMSAARV DSLSYERQAK SGFKCLPVTA PSSWGHPNSC
```

```
       61 SFPDIPVPNV SLTRKVGAEF VGTFILIFAA TAGPIVNQKY SGAETLIGNA ACAGLAVMII
      121 ILSTGHISGA HLNPSLTIAF AALRHFPWVQ VPAYIMAQVS ASICASFALK AVFHPFMSGG
      181 VTVPSVNTGQ AFALEFLITF NLLFVVTAVA TDTRAVGELA GIAVGATVML NILIAGPSSG
      241 GSMNPVRTLG PAVAAGNYEK LWIFLLAPTL GALAGATTYT VVKLRDNETD PPREARSFRR
//
LOCUS       CsNIP3-3       290 aa
DEFINITION  CsNIP3-3     290 aa
TITLE       CsNIP3-3
ORIGIN

        1 MKMNPCFDKQ SCSEISTCAS TSGQSGDDPE TGSNAMSIRN KGLLCIPHDI DLNPARMVIA
       61 ELVGTFILML CVCGIMASTV LTRGEVGLLE YAATAGLTII VLVYSIGPIS GAHVNPAVTI
      121 AFAVVGHFPL SKVPFYIMAQ TAGSVLGTYI GILVYGIKSN LMITRPAQHC VSAFWVELLA
      181 TSIIVFLAAS LACEAQCFGN LSGFVVGVAI GLAVLITGPV SGGSMNPARS LGPAIVSWNF
      241 SDIWIYIIGP TIGAVAGGFV YRFLRLRPRA CSPSTSPNTS LLSHSFMFVR
//
LOCUS       CsPIP1-1       288 aa
DEFINITION  CsPIP1-1     288 aa
TITLE       CsPIP1-1
ORIGIN

        1 MEGKEEDVRL GANKFTERQP IGTAAQSQDG KDYVEPPPAP LFEEEELTSW SFYRAGIAEF
       61 IATFLFLYIS VLTVMGVVKS NTKCSTVGIQ GIAWAFGGMI FALVYCTAGI SGGHINPAVT
      121 FGLFLARKLS LTRALFYMVM QCLGAICGAG VVKGFMGAGQ YGRLGGGANA VAHGYTKGDG
      181 LGAEIIGTFV LVYTVFSATD AKRSARDSHV PILAPLPIGF AVFLVHLATI PITGTGINPA
      241 RSLGAAIIYN KDHGWDDHWI FWVGPFIGAA LAALYHQVVI RAIPFKSS
//
LOCUS       CsPIP1-2       286 aa
DEFINITION  CsPIP1-2     286 aa
TITLE       CsPIP1-2
ORIGIN

        1 MEGKEEDVRL GANKFAERQP IGTAAQTQDG KDYVEPPPAP LFEPGELKSW SFYRAGIAEF
       61 IATFLFLYIS VLTVMGVVKS NTKCSTVGIQ GIAWAFGGMI FVLVYCTAGI SGGHINPAVT
      121 LGLFLARKLS LTRALFYMVM QCLGAICGAG VVKGFMGAGQ YGRLAGGANV VAHGYTKGDG
      181 LGAEIIGTFV LVYTVFSATD AKRSARDSHV PILAPLPIGF AVFLVHLATI PITGTGINPA
      241 RSLGAAIIYN KDRGWDDHWI FWVGPFVGAG LAALYHQVVI RAIPFK
//
LOCUS       CsPIP1-3       287 aa
DEFINITION  CsPIP1-3     287 aa
TITLE       CsPIP1-3
ORIGIN

        1 MEGKEEDVKL GANKFSERQP IGTSAQSDKD YKEPPPAPLF EPGELKSWSF YRAGIAEFMA
       61 TFLFLYITVL TVMGVSKSNT KCSTVGIQGI AWAFGGMIFA LVYCTAGISG GHINPAVTFG
      121 LLLARKLSLT RAVFYMVMQC LGAVCGAGVV KGFEGSKNYE RLGGGANVVA HGYTKGDGLG
      181 AEIIGTFVLV YTVFSATDAK RNARDSHVPI LAPLPIGFAV FLVHLATIPI TGTGINPARS
      241 LGAAIIFNKD HAWDDHWIFW VGPFIGAALA AVYHQIVIRA IPFKSRA
//
LOCUS       CsPIP1-4       287 aa
DEFINITION  CsPIP1-4     287 aa
TITLE       CsPIP1-4
ORIGIN
```

```
        1 MEGKEEDVRV GANRYRESQP IGTAAQTQDV KDYTEPPPAP LFEPGELSSW SFYRAGIAEF
       61 VATFLFLYIT VLTVMGVSKS KSKCSTVGIQ GIAWAFGGMI FALVYCTAGI SGGHINPAVT
      121 FGLFLARKLS LTRAVFYMIM QCLGAICGAA VVKGFQKSQY EMLGGGANML SKGYSKGDGL
      181 GAEIVGTFVL VYTVFSATDA KRNARDSHVP ILAPLPIGFA VFLVHLATIP ITGTGINPAR
      241 SLGAALIYNK DQAWDDHWIF WAGPFIGAAL AALYHQIVIR AIPFKSK
//
LOCUS       CsPIP2-1       287 aa
DEFINITION  CsPIP2-1     287 aa
TITLE       CsPIP2-1
ORIGIN

        1 MAKDIEVGGQ GEFHAKDYHD PPPAPLIDAE ELTQWSFYRA TIAEFIATLL FLYITVLTVI
       61 GYKSQTDANH GGDGCGGVGI LGIAWAFGGM IFVLVYCTAG ISGGHINPAV TFGLFLARKV
      121 SLVRAVMYMV AQCLGAICGC GLVKAFQKSY YTRYGGGANE LADGYSTGAG LGAEIIGTFV
      181 LVYTVFSATD PKRKARDPHV PVLAPLSIGF AVFMVHLATI PVTGTGINPA RSFGPAVIYN
      241 KDKAWDDQWI FWVGPFIGAA IAAFYHQFIL RASAAKALGS RRSSPNI
//
LOCUS       CsPIP2-2       287 aa
DEFINITION  CsPIP2-2     287 aa
TITLE       CsPIP2-2
ORIGIN

        1 MAKDIEVGGQ GEFHAKDYHD PPPAPLIDAE ELTQWSFYRA TIAEFIATLL FLYITVLTVI
       61 GYKSQTDANH GGDGCGGVGI LGIAWAFGGM IFVLVYCTAG ISGGHINPAV TFGLFLARKV
      121 SLVRAVMYMV AQCLGAICGC GLVKAFQKSY YTRYGGGANE LADGYSTGAG LGAEIIGTFV
      181 LVYTVFSATD PKRNARDSHV PVLAPLPIGF AVFMVHLATI PVTGTGINPA RSLGAAVIYK
      241 KDKAWDDQWI FWVGPFIGAA IAAFYHQFIL RASASKALGS FKSSSNI
//
LOCUS       CsPIP2-3       287 aa
DEFINITION  CsPIP2-3     287 aa
TITLE       CsPIP2-3
ORIGIN

        1 MAKDIEIGGQ GEFHAKDYHD PPPAPLIDAE ELTQWSFYRA TIAEFIATLL FLYITVLTVI
       61 GYKSQTDANH GGDGCGGVGI LGIAWAFGGM IFVLVYCTAG ISGGHINPAV TFGLFLARKV
      121 SLVRAVMYMV AQCLGAICGC GLVKAFQKSY YTRYGGGANE LADGYSTGTG LGAEIIGTFV
      181 LVYTVFSATD PKRNARDSHV PVLAPLPIGF AVFMVHLATI PVTGTGINPA RSLGAAVIYS
      241 KEKAWDDQWI FWVGPFIGAA IAAFYHQFIL RASASKALGS FKSSSNI
//
LOCUS       CsPIP2-4       198 aa
DEFINITION  CsPIP2-4     198 aa
TITLE       CsPIP2-4
ORIGIN

        1 MAKDIEVGRE GEFHDKDYHD PPPAPLIGAE ELTRWSFYRA IIAEFIATLL FLYITVLTVI
       61 GHKSQTDAKH GGDGCGGVGI LGIAWAFGGM IFVLVYCTAG ISGGHINPAV TFGLFLARKV
      121 SLVRAVMYMV AQCLGAICGC GLVKAFQKSY YTRYGGGANE LADGYSTGAG LGAEIIGTFV
      181 LVYTVFSSLR SRKATPII
//
LOCUS       CsPIP2-5       287 aa
DEFINITION  CsPIP2-5     287 aa
TITLE       CsPIP2-5
ORIGIN
```

1 MAKDTGVAEH GSYSAKDYTD PPPEPLFDAV ELTKWSFYRA LIAEFIATLL FLYVTVLTVI
61 GYKSQTDPAK NVDGCAGVGI LGIAWAFGGM IFVLVYCTAG ISGGHINPAV TFGLFLARKV
121 SLVRAVMYMV AQCLGAISGV GLVKAFQKSY YTRYGGGANE LADGYSTGVG LAAEIIGTFV
181 LVYTVFSATD PKRSARDSHV PVLAPLPIGF AVFMVHLATI PITGTGINPA RSLGAAVIYN
241 KDKAWDDQWL FWVGPFIGAA IAAFYHQFIL RAGAVKALGS FRSQSRV
//
LOCUS CsPIP2-6 289 aa
DEFINITION CsPIP2-6 289 aa
TITLE CsPIP2-6
ORIGIN

1 MGKDVEVAEQ QGGGGEFSAK DYHDPPPAPL IDFEELGKWS FYRAVIAEFI ATLLFLYVTV
61 LTVIGYKSQT DPNLNTDQCG GVGILGIAWA FGGMIFILVY CTAGISGGHI NPAVTFGLFL
121 GRKVSLIRAL LYMVAQCLGA ICGCGFVKAF QKSYYNRYGG GANELADGYN KGTGLGAEII
181 GTFVLVYTVF SATDPKRSAR DSHVPVLAPL PIGFAVFMVH LATIPITGTG INPARSFGAA
241 VIYNKEKAWD DQWIFWVGPF IGAFVAAFYH QYILRAAAIK ALGSFRSNA
//
LOCUS CsPIP2-7 283 aa
DEFINITION CsPIP2-7 283 aa
TITLE CsPIP2-7
ORIGIN

1 MSKEVNEEGQ THRHHHGKDY VDPPPAPLID MAELKLWSFY RALIAEFVAT LLFLYVSVAT
61 VIGHKKQSDA CGGVGLLGIA WAFGGMIFVL VYCTAGISGG HINPAVTFGL FLARKVSLIR
121 AVAYMVAQCL GAICGVGLVK AFMKHEYNSL GGGANTVASG YNKGSALGAE IIGTFVLVYT
181 VFSATDPKRS ARDSHVPVLA PLPIGFAVFM VHLATIPITG TGINPARSFG AAVIYNNDKA
241 WDDHWIFWVG PFVGALAAAA YHQYILRAAA IKALGSFRSN PSN
//
LOCUS CsSIP1-1 292 aa
DEFINITION CsSIP1-1 292 aa
TITLE CsSIP1-1
ORIGIN

1 MHTNPHPVLT FTITWDQTQI TFTWDQNKNG EKRKSNRKKG KIKDQIRKMG VIKAAIGDAV
61 LTSLWVFNLP FLGVLTGIVS KFLGVEALLP VTLLITTFLA TINVLVFSLL GHVLGGASFN
121 PSTTIAFYAA GLKPDSSLLS MAVRFPAQAA GGVAGAKAIL QVMPSQHRHR LKGPSLKVDL
181 HAGAVAEGAI TFVFCFALLF IMLKGPKTLI LQLWLLSVTT VGLVLTGSAY TGPSMNPANA
241 FGWAYVNNWH NTWDLFYVYW ICPFFGAFLA AWVFKFLFPA PSTPTTKKQK KS
//
LOCUS CsSIP1-2 244 aa
DEFINITION CsSIP1-2 244 aa
TITLE CsSIP1-2
ORIGIN

1 MGPVKAAAGD LVLTFMWVFV SSTFGVLTTE IASALGVHGL VWPPMLITTA IVFVFVFIFN
61 IIGDALGGAS FNPTGTAAFY AVGVGSDSLL SMALRFPAQA AGAVGGALAI MEYMPPKYKH
121 MLGGPSLQVD LHTGAIAEGV LTFLITFAVL VIMLKGPQSN ILKTWLVAVA TVVLIVTGSA
181 YTGPSMNPAN AFGWAYVNKW HDTWDQFYVY WICPFIGAVA ASMIFRVVFP PPAPARPAKK
241 KKKN
//
LOCUS CsSIP2-1 236 aa
DEFINITION CsSIP2-1 236 aa
TITLE CsSIP2-1
ORIGIN

```
        1 MAGIRLIVSD FILSFMWVWQ SVLIKIFVYK VLGLGHAPSG EVFKCGLSII SMFLFAFLGK
       61 VTKGGAYNPL TVLASGISGD FSNFLFTVGA RIPAQVIGSI TGVRFILDTF PQIGRGPSLN
      121 VGIHHGALTE GLLTFAIVTI SLGLARKIPG SFYMKTWISS VSKLALHILG SDMTGGCMNP
      181 ASVMGWAYAR GDHITKEHIF VYWLAPIQAT VLALWLFKLV VRPLAEEKKD SKSKSE
//
LOCUS       CsTIP1-1       250 aa
DEFINITION  CsTIP1-1     250 aa
TITLE       CsTIP1-1
ORIGIN

        1 MPGIAIGNPA EIGQPDALRA AVAEFFSMVI FVFAGQGSGM AYSKLTDNGA ATPAGLVSAS
       61 IAHAFALFVA VSVGANISGG HVNPAVTFGA FVGGHITFVR SILYWIAQLL GSVVACLLLK
      121 FSTGGLETSA FALSSGVGAW NAVVFEIVMT FGLVYTVYAT AVDPKRGNIG IIAPIAIGFI
      181 VGANILAGGA FDGASMNPAV SFGPAVVSWT WDNHWVYWLG PFIGAAIAAI AYTVAFICPN
      241 THEQLPSTDF
//
LOCUS       CsTIP1-2       252 aa
DEFINITION  CsTIP1-2     252 aa
TITLE       CsTIP1-2
ORIGIN

        1 MPIYRIAIGT PGEASHPDAL KAALAEFFSM IIFVFAGQGS GMAFSKLTDG GASTPAGLIS
       61 ASLAHAFALF VAVSVGANIS GGHVNPAVTF GAFVGGHITL LRSILYWIAQ LLGSVVACLL
      121 LKFSTGGLET SAFALSSGVS SWNAVVFEIV MTFGLVYTVY ATAVDPKKGN LGTIAPIAIG
      181 FIVGANILAG GAFDGASMNP AVSFGPAVVS WTWTNHWVYW LGPFIGAAIA AIVYDHIFID
      241 DNAHQPLPAN DF
//
LOCUS       CsTIP1-3       251 aa
DEFINITION  CsTIP1-3     251 aa
TITLE       CsTIP1-3
ORIGIN

        1 MPIRNIAVGH PREATHPDAL RAALAEFIST LIFVFAGEGS GMAFNKLTHN GANTPSGLVA
       61 ASVAHAFALF VAVAVGANIS GGHVNPAVTF GAFVGGNISL LRGILYWIAQ LLGSTVACLL
      121 LKFVTNGQTT SAFALSSGVG AWNAVVFEIV MTFGLVYTVY ATALDPKKGS LGTIAPIAIG
      181 FIVGANILAG GAFDGASMNP AVSFGPALVS WSWDNHWVYW VGPLIGGGLA GIVYEFFFIN
      241 QSHEQLPTTE Y
//
LOCUS       CsTIP2-1       247 aa
DEFINITION  CsTIP2-1     247 aa
TITLE       CsTIP2-1
ORIGIN

        1 MAIAFGRFDD SFSLGSFKAY LAEFISTLLF VFAGVGSAIA FNKVTADAAL DPSGLVAIAI
       61 CHGFALFVAV AVGANISGGH VNPAVTFGLA LGGQITILTG IFYWIAQLLG SIVASFLLKV
      121 VTGGLAVPTH NVAAGVGAIE GVVMEIIITF GLVYTVYATA ADPKKGSLGT IAPIAIGFIV
      181 GANILAAGPF SGGSMNPARS FGPAVASGNF QDNWIYWVGP LIGGGLAGLI YGNVFMHSEH
      241 APLSNDY
//
LOCUS       CsTIP2-2       249 aa
DEFINITION  CsTIP2-2     249 aa
TITLE       CsTIP2-2
ORIGIN
```

```
        1 MVKITLGSLG DSFSVGSFKS YLAEFIATLL FVFAGVGSAI AYSKLTADAA LDPAGLVAVA
       61 VAHALALFVG VAIAANISGG HLNPAVTLGL AVGGNITILT GIFYWIAQCL GSIVACLLLQ
      121 FVTSGLSIPT HAVGAGLNAA EGLVMEIVIT FALVYTVYAT AADPKKGPLG TIAPIAIGFI
      181 VGANILAAGP FSGGSMNPAR SFGPAVVSGD FSQIWIYWVG PLIGGGLAGL VYGDIFIGSY
      241 TPASTEDYA
//
LOCUS       CsTIP3-1       258 aa
DEFINITION  CsTIP3-1     258 aa
TITLE       CsTIP3-1
ORIGIN

        1 MARRRYELGK AEEATQPETL KAIVAEFLAT FTFVFATEGS ILALGKYEDT HEVISTSRLV
       61 AIAGTHACSL FLAVSMSLNI SGGHVNPAVT FGALVAGRIS LLRASLYCLA QLFRSVVASL
      121 LLRLVIFAGP RPVGFPLAFD RDWLGLIIEA AMTFGLVYTF YATTMDPRKS HLTTIAPLAM
      181 GFFAGANILI RGPFHGASMN PARAFGPALV GWRWTKNWIY WVGPLVGGGL GAITYEYMVM
      241 PSETPVLHHC IQQSCTSS
//
LOCUS       CsTIP3-2       329 aa
DEFINITION  CsTIP3-2     329 aa
TITLE       CsTIP3-2
ORIGIN

        1 MHTPNNRSRK IKILKKAAAS SINTLLFTFS PSKSNSHKQK QKQLFGGDNL IVVFEEIKKI
       61 DIKITSTRVY IANMPPRRYA IGRADEAAHP DTMRAALAEF VSTLIFVFAG EGSILALDKI
      121 SKATTTSPSD LVVIALAHAL ALFAAVASSI NTSGGHVNPA VTFGALLGGR ISVVRAIYYW
      181 IAQLLGAIVA ALLLRFATNG MRPVGFYVAS GVGEGHGLVL EIVMTFGLVY TVYATVIDPK
      241 RGSLGIVGPL AIGFIVGANI LVGGPFDGAS MNPARAFGPA LVGWRWRNHW IYWVGPFLGA
      301 GLAALIYEYI VIPTEPPIHT HQPLAPEDY
//
LOCUS       CsTIP4-1       247 aa
DEFINITION  CsTIP4-1     247 aa
TITLE       CsTIP4-1
ORIGIN

        1 MAKIALGSHR EATQPDCIKA LVVEFITTFL FVFVGVGSAM AADKLVGNAL VGLFFVAVAH
       61 AFVVAVMISA GHISGGHLNP AVTLGLLFGG HITFFRSILY WIDQLLASSA ACFLLSYLTG
      121 GLSTPVHTLA SGVGYFQGII WEIILTFSLL FTVYATIVDP KKGSIDGLGP LLTGFVVGAN
      181 ILAGGPFSGA SMNPARSFGP ALVSGDWTDH WVYWVGPLIG GGLAGFIYEH FFIVRSHVPI
      241 PYEEARF
//
LOCUS       CsTIP5-1       254 aa
DEFINITION  CsTIP5-1     254 aa
TITLE       CsTIP5-1
ORIGIN

        1 MASTTLTARF NQSVTKEALR SYLAEFISTF FYVFAVVGSA MASKKLSPDA ASNTSSLVVA
       61 AIANVFALSS TVYIAANISG GHVNPAVTFA KAVSGHITVP TALFYWVSQM LASVMASLLL
      121 RVTAIGQNIP AYTIAQEMTG FGASLLEGVL TFALVYTVYA AGDPRGSPLG AIGPLAIGMM
      181 AGANVLATGP FSGGSMNPAC AFGSAVVAGS FKNQAVYWVG PLFGAAVAGL VYDIVVFPAQ
      241 VQDSIAGVSD GIGV
//
LOCUS       CsXIP1-1       354 aa
DEFINITION  CsXIP1-1     354 aa
```

```
TITLE       CsXIP1-1
ORIGIN

        1 MDLIVSHEAA DPLPSQGFSI EKNEIMNGTI TRREMFLACI GFYEFFSPKN QSQCLTFYSP
       61 CVMRKLYFNY FFNFLAHFWA FRPFMWRAAF TELVATAFLV FTLTTSIISC LDSHVSEQKL
      121 LVPIAVFIIA FLFLMVTVPL SGGHMSPVFT FIAALQGIVT LARAATYVLA QCLGSIVGFL
      181 IINSVMSHNA ARRYSLGGCL IAGNGTSAGI SAETALILEF TCTFVVLFVG VTIAFDKRRC
      241 KELGLVVVCA IVAGAMAIAV FVSITVTQKP GYAGVGLNPA RCLGPALLHG GPLWKGHWVF
      301 WVGPFLACVV YYGFAKTLPE EGLVWEDKFE HDIINLARVW RNGTGTSCHL QRKK
//
LOCUS       CsXIP1-2       306 aa
DEFINITION  CsXIP1-2     306 aa
TITLE       CsXIP1-2
ORIGIN

        1 MAANSELAGD IEGEYGGNRV QPFATPKQEQ RLMDEEKRQN PSTWKKILGF EDIFSVKVWR
       61 ASMAELLGTA VLVFALDTIV ISSIQTDTKT PNLVMSTLVA IIITILLIAT FPISGGHINP
      121 LVSFAAALTG ITSFTRAAIY ILAQCVGGIF GALALEAVVT KKIAHNFSLG GCTLNVVVPG
      181 PDGPVEIGLG TRQALWLEII CSFVFLFASV WMAFDSRQAK ALGRVTVCIV IGTVLGLLVF
      241 VSTTVTAQKG YAGAGLNPAR CLGPALVRGG HLWDRHWVFW AGPAIACVAF ALYTKLIPSQ
      301 NLHTIK
//
LOCUS       CsXIP1-3       254 aa
DEFINITION  CsXIP1-3     254 aa
TITLE       CsXIP1-3
ORIGIN

        1 MEDRLIVGRG KKQQPSSLKK IMGFEELFSL NVWRASLTEV LGTALLVFAL DTIVISSIQT
       61 DTIIAILLLA TFPISGGHIN PLVTFSAALI GHMTVTRAAI YILAQCVGGV FGALAPKAVV
      121 STKIEHAFSL GGCTLSVVEQ QPNGPVELGL DTGAALWLEI FCSFVFLFAS VWMAFDERQA
      181 KALARVSVCI ILGVVLGPLI FVSTLLLPKR DTAVLGVTRQ GVWAPRSSEE AISGIDTGFS
      241 EPAPLLLAWH LPCT
//
LOCUS       EgNIP1-1       206 aa
DEFINITION  EgNIP1-1     206 aa
TITLE       EgNIP1-1
ORIGIN

        1 MGGNGEHSVG NGTQERSMEE GAAGFPLEEE NAIPDHGYEN STTVSFLQKI LAEVFGTYFL
       61 IFAGCGSVTV NLSKGTVTFP GICIVWGLVV MVLVYSVGHI SGAHFNPAVT IAFATCRRFP
      121 WKQVPAYILA ELMGSTLASG TLRLLFGGKR ELFPGTIPAG SDVQSLVLEF IITFYLMFVI
      181 SGVGTDKRAV RHSSQNDVNM DETCGF
//
LOCUS       EgNIP1-2       316 aa
DEFINITION  EgNIP1-2     316 aa
TITLE       EgNIP1-2
ORIGIN

        1 MPGGRENSTG ENGTRERSME EGTAGFPGEE ERSVPGHGLG LSFPFFQKII AEVFATYFLI
       61 FAGCASVTVN LSKGTITLPG VGIVWGLVVM VMIYSVGHIS GGHFNPAVTI AFATCRRFPW
      121 KQVRLYPTVL MLANSMHAFC MTVIYLKFLF PPIMSVQVPV YVAAQLLGAI LASGTLRLLF
      181 GGKHELFPGT IPTGSDMQSL VLEFITTFYL MFVISGVATD NRAIGELAGI AVGATILLNV
      241 IIAGPISGAS MNPVRSLGPA IVANRYKSIW VYIVGPTCGA VAGAWAYNVI RFTNKPLREI
      301 TKSGSFLKSL GRNDSN
```

```
//
LOCUS       EgNIP2-1       227 aa
DEFINITION  EgNIP2-1     227 aa
TITLE       EgNIP2-1
ORIGIN

        1 MASFPRPGRS NEIHDMATPQ NPITSRPPQT KSLEDLLPPF LLKKVVAEVI ATFLLVFVTC
       61 GAAALSKYDQ RIISQLGQSV AGGLIVTVMI YAVGHISGAH MNPAVTLSFA VLRHFPWIQV
      121 PFYWSAQISG AVIASFILRQ LLHPITNLGT TIPSGTPLQA LVMEIVVTFC MMFVTLAVAT
      181 DTKARPSLRN TPKICRRMRP HQRRPDCSRW TDSTQIDLSR SQAYDFE
//
LOCUS       EgNIP2-2       249 aa
DEFINITION  EgNIP2-2     249 aa
TITLE       EgNIP2-2
ORIGIN

        1 MDGKLEPDRV STDGAVAAGV VSEIIATFLL VFVTCGSAAL YTSNHDRISQ LGQSVAGGLI
       61 VTVMIYAVGH ISGAHMNPAV TLSFAVYRHF PWIQMKSELC RPVSGGSMNP ARTLGPAVAS
      121 KKYNGLWVYF IGPPMGTLSA GLAYTFIRLT NKDGSQKLSS FKLRRLQSQG SPIPTEEYLE
      181 NELGMGSARR SSDRRWSSDR PATASSVVSP APSTPSTPSS GSETADSRRC VQPTPELAID
      241 TRQPAIDPR
//
LOCUS       EgNIP3-1       329 aa
DEFINITION  EgNIP3-1     329 aa
TITLE       EgNIP3-1
ORIGIN

        1 MLCDQLPIDR WLVKFLVGPD PQAHSVDMGE DGASALENPG VPLLGGHKSS RFADRWRSLL
       61 GRGNCFAVKP WTIDDDGPSI PASGSLVRKN EKEATTNQQF LVIYGQVGAE FIGTFMLVFA
      121 STAAAIIDQQ SNGAVTLLGL AACSGIAAMI IILSTGHISG AHLNPSVTIA FATFKHFSWE
      181 KVLVYIAAQL SASLCAAFAL KAIFYPILGE GVTVPSGSAG AAFVMEFIIG FNLMFVTTAV
      241 ATDVRAVGEL AGITVGATVA MNNIIAGRIS GASMNPARTL GPAVAANNYK AIWVYFTAPI
      301 LGTLTGAGAY TAVKLTEDDG TPTMRSFRR
//
LOCUS       EgNIP3-2       342 aa
DEFINITION  EgNIP3-2     342 aa
TITLE       EgNIP3-2
ORIGIN

        1 MGGVFVMHKG PMDQKSEINI LNTVQDMGED GASALENPGV PLLGGHKSSR FADRWRSLLG
       61 RGNCFAVKPW TIDDDGPSIP ASGSLVRKVG AEFIGTFMLV FASTAAAIID QQSNGAVTLL
      121 GLAACSGIAA MIIILSTGHI SGAHLNPSVT IAFATFKHFS WEKVLVYIAA QLSASLCAAF
      181 ALKAIFYPIL GEGVTVPSGS AGAAFVMEFI IGFNLMFVTT AVATDVRAVG ELAGITVGAT
      241 VAMNNIIAGR ISGASMNPAR TLGPAVAANN YKAIWVYFTA PILGTLTGAG AYTAVKLTED
      301 DGTPTMRSFR RRLSWSPTPA IGLLSPYIFQ SHLAATDVCP LD
//
LOCUS       EgNIP3-3       288 aa
DEFINITION  EgNIP3-3     288 aa
TITLE       EgNIP3-3
ORIGIN

        1 MADIGDDGAS TPASPGSPLF GGHKAGRAVD GWRSLLNHRN CFRVKPWTID DDAPSPLTSL
       61 SLARKVGAEF IGTFMLVFAS TAAAIIEHQS GGAVTLFGVA AASGLAAMLV ILSTGHISGA
      121 HLNPSVTIAF ATFKHFSWEQ VLVYIAAQMS ASLCAAFTLK AIFYPVLGEG ATVPSGSVSA
```

```
      181 AFVMEFIIGF NLMFVTTAVA TDSRAVGELA GIAVGATVAM NNLIAGRISG ASMNPARTLG
      241 PAVAANNYKA IWVYFTAPIL GALIGAGAYS AVKLPKDDGT PTARSFRR
//
LOCUS       EgNIP3-4       299 aa
DEFINITION  EgNIP3-4     299 aa
TITLE       EgNIP3-4
ORIGIN

        1 MPETENGTPN VSAPPTPGTP GAPLFSSLRV DSLSYDRNSM PRCNRCLPVS SYSWASPPTC
       61 FTEIPKPDVS LTRKLGAEFV GTFILIFGAT AAPIVNQKYN GAETLIGNAA CAGLAVMIVI
      121 LSTGHISGAH LNPSLTIAFA CLRHFPWVQV PAYIFAQVSA SICASFALKA IFHPFLSGGV
      181 TIPSVTTAQA FFLEFCITFN LLFVVTAVAT DTRAVGELAG IAVGATVMLN ILVAGPSSGG
      241 SMNPVRTLGP ALAAGNYKQI WIYLVAPTAG AIAGAATYTL VKLTDEDGVT PRTPRSFRR
//
LOCUS       EgNIP3-5       297 aa
DEFINITION  EgNIP3-5     297 aa
TITLE       EgNIP3-5
ORIGIN

        1 MPETETGTPN VSAPPTPGTP GAPLFSSVRV DSLSYDRNSM PRCGKCLPVD SWASPGTCFT
       61 ELPKPNVSLA RKLGAEFVGT FILIFGATAG PIVNQKYNGA ETLIGNAACA GLAVMVVILS
      121 TGHISGAHLN PSLTIAFACL RHFPWVQVPA YIAAQVSASI CASFALKGIF HPFLSGGVTV
      181 PSVTTAQAFF LEFFITFNLL FVVTAVATDT RAVGELAGIA VGATVMLNIL VAGPSSGGSM
      241 NPVRTLGPAV AAGNYKQVWI YLVAPTAGAI AGAATYTLVK LTDENGVTPR SARSFRR
//
LOCUS       EgPIP1-1       287 aa
DEFINITION  EgPIP1-1     287 aa
TITLE       EgPIP1-1
ORIGIN

        1 MEGKEEDVRL GVDKFPERQP IGTAAQTQDK DYKEPPPAPL FEPGELQSWS FYRAGIAEFM
       61 ATFLFLYITI LTVMGVVKSP SKCSTVGIQG IAWAFGGMIF ALVYCTAGIS GGHINPAVTF
      121 GLLLARKLSL IRALFYMVMQ CLGAICGAGV VKGFQKGLYE SNGGGANVVA PGYTKGDGLG
      181 AEIIGTFVLV YTVFSATDAK RNARDSHVPI LAPLPIGFAV FLVHLATIPI TGTGINPARS
      241 LGAAIIYNRD HAWDDQWIFW VGPFIGAALA ALYHQVVIRA IPFKTRP
//
LOCUS       EgPIP1-2       287 aa
DEFINITION  EgPIP1-2     287 aa
TITLE       EgPIP1-2
ORIGIN

        1 MEGKEEDVKL GANKFAERQP IGTAAQSQDK DYKEPPPAPL FEPGELRSWS FYRAGIAEFM
       61 ATFLFLYISI LTVVGVAKAP SKCSTVGIQG IAWAFGGMIF ALVYCTAGIS GGHINPAVTF
      121 GLLLARKLSL IRAVFYMVMQ CLGAICGVGV VKGFQTGLYE SNGGGANVVA RGYTKGAGLG
      181 AEIIGTFVLV YTVFSATDAK RNARDSHVPI LAPLPIGFAV FLVHLATIPI TGTGINPARS
      241 LGAAVIYNQR HAWDDMWIFW VGPFCGAALA AIYHQIVIRA IPFKSRP
//
LOCUS       EgPIP1-3       287 aa
DEFINITION  EgPIP1-3     287 aa
TITLE       EgPIP1-3
ORIGIN

        1 MEGKEEDVRL GANKFSERQP IGTAAPSQDK DYKEPPPAPL FEPGELRSWS FYRAGIAEFM
       61 ATFLFLYITI LTVMGVVKSK SKCSTVGIQG IAWAFGGMIF ALVYCTAGIS GGHINPAVTF
```

```
      121 GLLLARKLSL TRALFYMVMQ CLGAICGAGV VKGFQKGIYE SNGGGANVVA PGYTKGDGLG
      181 AEIIGTFVLV YTVFSATDAK RNARDSHVPI LAPLPIGFAV FLVHLATIPI TGTGINPARS
      241 LGAAIIYDKD HAWDDQWIFW VGPFIGAALA AFYHQIVIRA IPFKSRP
//
LOCUS       EgPIP2-1       287 aa
DEFINITION  EgPIP2-1     287 aa
TITLE       EgPIP2-1
ORIGIN

        1 MGKDVEAAAE GGEFTAKDYT DPPPAALIDA EEMIKWSLYR AVIAEFIATL LFLYITVATV
       61 IGYKHQSDPN VGGADAACSG VGILGIAWAF GGMIFILVYC TAGISGGHIN PAVTFGLFLA
      121 RKVSLVRALL YMIAQCLGAI CGVGLVKGFQ RAYYDRYGGG ANELSSGYSK GTGLAAEIIG
      181 TFVLVYTVFS ATDPKRNARD SHVPVLAPLP IGFAVFMVHL ATIPITGTGI NPARSLGAAV
      241 IYNNEKAWDD QWIFWVGPFI GAAIAAAYHQ YVLRASAGKA LGSFRSG
//
LOCUS       EgPIP2-2       285 aa
DEFINITION  EgPIP2-2     285 aa
TITLE       EgPIP2-2
ORIGIN

        1 MRKDMEAGAE FTAKDYTDPP PAPLVDAEEL TKWSLYRAVI AEFIATLLFL YITVATVIGY
       61 KHQSDPNVSG PDAACDGVGI LGIAWAFGGM IFVLVYCTAG ISGGHINPAV TFGLFLARKV
      121 SLIRALLYMI AQCLGAICGV GLVKAFQKAY YNRYGGGVNE LSSGYSKGTG LAAEIIGTFV
      181 LVYTVFSATD PKRSARDSHV PVLAPLPIGF AVFMVHLATI PITGTGINPA RSLGPAVIYN
      241 NDKAWDDQWI FWVGPFIGAA IAAAYHQYIL RAGAIKALGS FRSNA
//
LOCUS       EgPIP2-3       189 aa
DEFINITION  EgPIP2-3     189 aa
TITLE       EgPIP2-3
ORIGIN

        1 MEAEPAREAL GRAVEDFQGS KEYNEEIFEN GFASYYVGYE DSRDTVKKLY PSLDLSSIIL
       61 PRSEDGVVEE EATPTQEEAP TGSEIVQVDV ATPEQRNKND DEAWSGVLAP LPIGFAVFMV
      121 HLATIPITGT GINPARSFGA AVIYNEDKAW DHQWIFWVGP FIGAAIAAAY YQYVLRASAG
      181 KPLGSYRSG
//
LOCUS       EgPIP2-4       282 aa
DEFINITION  EgPIP2-4     282 aa
TITLE       EgPIP2-4
ORIGIN

        1 MSKEVSEEAE QAPPKDYADP PPAPLLDMGE LRLWNLYRAL IAEYVATLLF LYVTIATVIG
       61 YKVQSQNDPC GGVGYLGIAW SFGGMIFILV YCTAGISGGH INPAVTFGLF LARKVSLLRA
      121 LLYMVAQCLG AICGVGIVKG IMKHPYNSLG GGVNEVASGY SKGTALGAEI IGTFILVYTV
      181 FSATDPKRKA RDSHVPVLAP LPIGFAVFMV HLATIPITGT GINPARSLGP AVIYNNKKAW
      241 DDQWIFWVGP FVGALAAAAY HQYILRAAAI KALGSFRSSH GN
//
LOCUS       EgPIP2-5       282 aa
DEFINITION  EgPIP2-5     282 aa
TITLE       EgPIP2-5
ORIGIN

        1 MSKEVSEEAA HPSAKDYTDP PPAPLLDMGE LRLWSFYRAL IAEFVATLLF LYVTIATVIG
       61 YKVQSQNDQC GGVGVLGIAW AFGGMIFILV YCTAGISGGH INPAVTFGLF LARKVSLVRA
```

```
      121 LLYMVAQCAG AICGVGIVKG IMKHPYNSLG GGANEVAPGY SKGTALGAEI IGTFVLVYTV
      181 FSATDPKRNA RDSHIPVLAP LPIGFAVFMV HLATIPITGT GINPARSFGA AVIYNQGKAW
      241 DDQWIFWVGP FVGALAAAAY HQYILRAAAI KALGSFRSNP HN
//
LOCUS       EgPIP2-6       282 aa
DEFINITION  EgPIP2-6     282 aa
TITLE       EgPIP2-6
ORIGIN

        1 MVKEMSMEGE HQMVKDYKDP PPAPLLGVEE LRRWALYRAL IAEFIATLLF LYVTIATVIG
       61 YKRQSQADRC NGVGLLGVAW SFGGMIFVLV YCTAGISGGH INPAVTFGLF LARKVSLIRA
      121 VLYMVAQCLG AICGAGLVKA FMKHSYNSLG GGASEVAPGY SKGSALGAEI IGTFILVYTV
      181 FSATDPKRKA RDSHVPVLGP LPIGFSVFMV HLATIPITGT SINPARSFGP AVIYNQHKAW
      241 RDQWIFWVGP MLGAAAAAAY HQYVLRAHVV KALTSFRSNS HN
//
LOCUS       EgSIP1-1       193 aa
DEFINITION  EgSIP1-1     193 aa
TITLE       EgSIP1-1
ORIGIN

        1 MGAIKAAVGD AVITFLWVFC VSSVGALTTV IASALQVQGA AYSLVITTTL IFLLLFVFSP
       61 ICDAIGGASF NPTGTAAFYA AGFGGDNLVS MALRFPAQAA GAVGGALAIA ELMPRPYKHM
      121 LGGPSLKVDL HTGAAAEGVL TFCITLAVLW IVIKGPSSAF VKTWLLAVTT VTFVLAGAGY
      181 TGPSMNPANL PPR
//
LOCUS       EgSIP2-1       246 aa
DEFINITION  EgSIP2-1     246 aa
TITLE       EgSIP2-1
ORIGIN

        1 MVDGGGEVVA EDLRTKPDSG GVVAEVTRRR HSGRSTARAS VLLLPSSSYA FKSGLSKRLT
       61 LVFLVSELLE FSGRHHEPSP QLPFRSFELD ELNRRFVPLR GPRLSSNTTS KCSTPFPQTP
      121 LSPTLTRPRR GRRERTDEEV ELHKVMARFR LVVSDFLVSF LWVWSGSILR YLAYGILGLG
      181 MHPIALMIKG SFAVVYVYCF SWLGKATRGG SYNPLIVLCY GISGNFSGFL FTVCGRIPAQ
      241 VSFLYP
//
LOCUS       EgTIP1-1       250 aa
DEFINITION  EgTIP1-1     250 aa
TITLE       EgTIP1-1
ORIGIN

        1 MPISRIAIGT REEATHPSAL RAALAELIST LIFVFAGQGS GMAFSKLTGG SSTTPAGLVA
       61 ASLAHAFGLF VAVSVAANIS GGHVNPAVTF GAFVGGNITL LRGILYWFAQ LLGSTIACFL
      121 LRFSTGGLST GTFGLTGVSV WEALVLEIVM TFGLVYTVYA TAVDPKRGNL GTIAPIAIGF
      181 IVGANILAGG AFDGASMNPA VSFGPALVSW SWSDHWVYWV GPLIGGGLAG LVYEFFFISN
      241 THEQLPSADY
//
LOCUS       EgTIP1-2       250 aa
DEFINITION  EgTIP1-2     250 aa
TITLE       EgTIP1-2
ORIGIN

        1 MPISRIAIGT RDEATHPSAL KAAFAEFIST LIFVFAGQGS GMAFSKLTGG GSTTPAGLVA
       61 ASLAHAFGLF VAVSVGANIS GGHVNPAVTF GAFIGGNITL LRGILYWIAQ LLGSTAACLL
```

```
      121 LRFCTGGLAT GTFGLTGVTV WEAFVLEIVM TFGLVYTVYA TAVDPKRGSL GTIAPIAIGF
      181 IVGANILVGG AFDGASMNPA VSFGPALVSW SWDDHWVYWG GPLIGGGLAG LVYEFFFISH
      241 THEQLPTTDY
//
LOCUS       EgTIP1-3       250 aa
DEFINITION  EgTIP1-3     250 aa
TITLE       EgTIP1-3
ORIGIN

        1 MPISRIAIGT RDEATHPSAL KAAFAEFIST LIFVFAGQGS GMAFSKLTGG GSTTPAGLVA
       61 ASLAHAFGLF VAVSVGANIS GGHVNPAVTF GAFIGGNITL LRGILYWIAQ LLGSTAACLL
      121 LRFCTGGLAT GTFGLTGVTV WEAFVLEIVM TFGLVYTVYA TAVDPKRGSL GTIAPIAIGF
      181 IVGANILVGG AFDGASMNPA VSFGPALVSW SWDDHWVYWG GPLIGGGLAG LVYEFFFISH
      241 THEQLPTTDY
//
LOCUS       EgTIP1-4       252 aa
DEFINITION  EgTIP1-4     252 aa
TITLE       EgTIP1-4
ORIGIN

        1 MPLNRIAIGE PGEASQPDAL KAAVAEFFSM LIFVFAGEGS GMAFSKLTGD GPATPSGLVA
       61 AALAHAFALI VAVSVGANIS GGHVNPAVTF GAFVGGNVTL LRAILYWIAQ LLGSVVACIL
      121 LKFATGGMKT AAFAVSADVT PMNAFVLEIV MTFGLVYTVY ATAVDPKKGN LGVIAPIAIG
      181 FIVGANILAG GAFDGASMNP AVSFGPAVVS WTWDHHWVFW LGPMIGAALA AIVYDMCFIC
      241 RGTHEQLPTT DY
//
LOCUS       EgTIP1-5       253 aa
DEFINITION  EgTIP1-5     253 aa
TITLE       EgTIP1-5
ORIGIN

        1 MPISRIAIGS PGEASQPDTL KAALAEFISM LIFVFAGEGS GMAFNKLTND GSTTPAGLVA
       61 AALAHAFALF VAVSVGANIS GGHVNPAVTF GAFIGGNITL LRGILYWIAQ LLGSVVACLL
      121 LKFATGGLKT AAFSLSTDVS AWNALVFEIV MTFGLVYTVY ATAVDPKKGD LGVIAPIAIG
      181 FIVGANILAG GAFDGASMNP AVSFGPAVVS WTWDNHWVYW LGPLIGAAVA AIVYDLFFIG
      241 HGTTHEQLPT TDY
//
LOCUS       EgTIP2-1       248 aa
DEFINITION  EgTIP2-1     248 aa
TITLE       EgTIP2-1
ORIGIN

        1 MAGIAFGRCD DSFSVHSLKA YLAEFISTLL FVFAGVGSAI AYNKLTSGSA LDPTGLVAVA
       61 ICHGFALFVA VSIAANISGG HVNPAVTFGL ALGGQITILT GILYWIAQLL GAIVGAFLIK
      121 LVTGMATPTH GLAAGVGAIE GVVMEIITTF ALVYTVYATA ADPKKGSLGT IAPIAIGFIV
      181 GANILAAGPF SGGSMNPARS FGPAVASGDF TDNWIYWVGP LIGGGLAGLV YTYAFMCSVH
      241 EPIASSEF
//
LOCUS       EgTIP2-2       248 aa
DEFINITION  EgTIP2-2     248 aa
TITLE       EgTIP2-2
ORIGIN

        1 MAGIAFGRCD DSFSVHSLKA YLAEFISTLL FVFAGVGSAI AYNKLTSGSA LDPTGLVAVA
```

```
      61 ICHGFALFVA VSIAANISGG HVNPAVTFGL ALGGQITILT GILYWIAQLL GAIVGAFLIK
     121 LVTGMATPTH GLAAGVGAIE GVVMEIITTF ALVYTVYATA ADPKKGSLGT IAPIAIGFIV
     181 GANILAAGPF SGGSMNPARS FGPAVASGDF TDNWIYWVGP LIGGGLAGLV YTYAFMCSVH
     241 EPIASSEF
//
LOCUS       EgTIP3-1       256 aa
DEFINITION  EgTIP3-1     256 aa
TITLE       EgTIP3-1
ORIGIN

       1 MPLRPRFAFG RTEDAVHPDT MRAALSEFIG TAIFVFAGEG SVLSLEKLYR DTSTAGGLVM
      61 VAIAHALAFF VAVSIAFNTS GGHINPAVTF GALVGGRISL IHAVCYWVAQ LLGAIIASLL
     121 LRVTTGGMRP LGFSLGSGIG EWHAVLLEIV LTFGLVYTVY ATAIDPKRGY LGTIAPLAIG
     181 FIVGANILAG GPFDGASMNP ARAFGPALVG WRWRRHWVYW LGPFVGAGLA GLTYEFLVIP
     241 TETTPHAHQP LAPEDY
//
LOCUS       EgTIP4-1       311 aa
DEFINITION  EgTIP4-1     311 aa
TITLE       EgTIP4-1
ORIGIN

       1 MAKIAVGSRE EVREPDCIRA VLAETILTFL FVFICVGAAM TAEKMTGGKD AIVGLMAVAV
      61 TNALVVAVMI AAGLHISGGH LNPAVTAGMA LGGRITLFRS ILYVAAQLLG SSVACFLLKY
     121 ITGGMDTPVH SLGAGMVAVQ GVIMETVLTF ALLFSVYATI LDPRKGIVAG LGPLFVGLVV
     181 GANILACGPF TGASMNPARS FGPALASQNW TNHWVYWVGP MIGSLLATFV YEHLFMAGTH
     241 EPLYSNEENF SGNDHITLIR EWFKKSEYKR FHKIDEQFSF CHQIQSPKYS TTYDAAHCSP
     301 PYLPVHYRKG P
//
LOCUS       EgTIP4-2       332 aa
DEFINITION  EgTIP4-2     332 aa
TITLE       EgTIP4-2
ORIGIN

       1 MAKIALGSRQ EVAEPDCIRA VLAEIVLTFL FVFAGVGAAM TAGKMAGGTD TIMGLTAVAV
      61 AHALVVAVMI SAGLHISGGH LNPAVTLGLA VGGHITLFRS ILYIIAQLLG SSLACLLLKY
     121 LTGGMDTPIH ALAAGMGAVQ GVIMEIVLTF SLLFSVYATI VDPKKGIIAG LGPLLVGLVV
     181 GANILAGGPF SGASMNPARS FGPALASWDW TDHWVYWVGP LIGGPLAGFI YEHVFLIRTH
     241 EPLPRDDDKV TLGLALDPAG LVAVAICHGF ALYVAVSVGA NISGGHVNPA VTFGLALGGQ
     301 ITILTGIFYW IAQLLGAIAA SFLLKAVTGL IS
//
LOCUS       FvNIP1-1       271 aa
DEFINITION  FvNIP1-1     271 aa
TITLE       FvNIP1-1
ORIGIN

       1 MAENHESSRN HVVSVEDDKP SSKTNQESSR AVLSVPFMQK VIAEVLGTYF LIFAGCGAVI
      61 VNFNTDKTVS SPAVAIVWGL VVMVLNYSVG HISGAHFNPA VTIAFAATKR FPWKQVLPYI
     121 AAQVFGSILA SGTLRLIFNH HEDHFVGTSP SGTQLQSFVI EFIITFYLMF VVSGVATDNR
     181 ATGELAGLAV GSTILLNVMF AGPVTGASMN PARSLGPAIV WSQYKGLWIY LVSPILGALC
     241 GALVYNVIRF TDKPLHELTK TSSFLKGVDR T
//
LOCUS       FvNIP1-2       268 aa
DEFINITION  FvNIP1-2     268 aa
TITLE       FvNIP1-2
```

```
ORIGIN

        1 MKVVKTILSM LKKANLPLET VIAEVLGTYF LIFAGCGSVI VNLSTDKLVS FPGISITWGL
       61 TVMVMVYSVG HISGAHFNPA VTLAFAIIHR FPWKEMRKFL ELYVLARKHF DASKWPLAPC
      121 YREMHFLHAY TTTSLVPAYI IAQVLGSTLA SGTAFVLEFI ITFYLMFVVS GVATDSRAVG
      181 ELAGLAVGST VLLNVMFAGP ITGASMNPAR SLGPAIVWSH YKGLWIYLVS PILGALCGAL
      241 VYNVIRFTDK PLNELTKTSS FLKGVDRT
//
LOCUS       FvNIP1-3       265 aa
DEFINITION  FvNIP1-3     265 aa
TITLE       FvNIP1-3
ORIGIN

        1 MANSPSIPSP TLGVSSKLST VEEADANRIQ AEVPPTIFQK MVAELVGTYV LVFMGCGAAL
       61 VNKTHPLTTV GIAIVWGLVL MALTYTVGHV SGCHLNPAVT IALAAARKFP SKHVPMYALS
      121 QMMGAILASL TLRLLFQDNI DLMVTQYSDS TTHLQALVWE FIATFILMFT ICGVATDHRA
      181 SKKLAGVAIG VTVLFNAMVA GPITGASMNP ARSFGPAAVT GIYKNLWVYL IAPILGALAA
      241 TALYSVLRVP EPVKADPKDK SLPPV
//
LOCUS       FvNIP1-4       271 aa
DEFINITION  FvNIP1-4     271 aa
TITLE       FvNIP1-4
ORIGIN

        1 MAENHATNGN HVLNVEDDRP PSITNQESSI SFLCVPFMQK VIAEVLGTYF LIFAGCGAVV
       61 VNLNTDKTVS SPGIAIVWGL VVMVMIYSVG HISGAHFNPA VTIAFATTKR FPLKQVPPYV
      121 VAQVLGSTLA SGTLRLIFNN HQDHFAGTSP NGTPLQSFVI EFIITFYLMF VVSGVATDNR
      181 AIGELAGLAV GSTVLLNVMF AGPISGASMN PARSLGPAIV SSHYKNLWIY LVAPTLGAVC
      241 GALVYNVIRF TDKPLRELTK TGSFLKGPGR T
//
LOCUS       FvNIP1-5       270 aa
DEFINITION  FvNIP1-5     270 aa
TITLE       FvNIP1-5
ORIGIN

        1 MAENHATNGN HVLNVEHDSP SKTNQESSSS FLCVPFMQKV IAEVLGTYFL IFAGCGAVVV
       61 NLNTDKTVSF PGIAIVWGLV VMVMIYSVGH ISGAHFNPAV TIAFATTKRF PWKQVPPYVV
      121 AQVLGSTLAS GTLRLIFNNH QDHFAGTSPK GTPLQSFVIE FIITFYLMFV VSGVATDNRA
      181 IGELAGLAVG STVLLNVMFA GPISGASMNP ARSLGPAIVS SHYKNLWIYL VAPTLGAVCG
      241 ALVYNVIRFT DKPLRELTKS GSFLKGAGRT
//
LOCUS       FvNIP2-1       206 aa
DEFINITION  FvNIP2-1     206 aa
TITLE       FvNIP2-1
ORIGIN

        1 MAKTELVSVE NPIVEHPFYP PGFLKKVVAE IIATFLLVFV TCGSSALSAS DERKISKLGA
       61 SMTGGLIVTV MIYAVGHVPA YALAQLIGSI SASFTLSLLL HPIKHVGTTS PSGSELQALV
      121 TEIIIGELAG IAVGSAVCIT SIFAGPISGG SMNPARTIGP ALASAYYNGI WICMVGPVIG
      181 TLLGAWSYSF IRMNDKPVQA SPPRSL
//
LOCUS       FvNIP2-2       468 aa
DEFINITION  FvNIP2-2     468 aa
TITLE       FvNIP2-2
```

```
ORIGIN

        1 MATKEPTELR NETNEFDFQK SSVFEQHYPF LKKVVAEIIA TFLLVFVTCG SAALSASDEH
       61 KVSKLGASIV GGLIVTAMIY AVGHISGAHM NPAVTLAFSA VRHFPWKQVP IYAVAQLTGS
      121 ISASFTLSIL LHPIKHVGTT SPSGSDIQAL IVEIVMTFCM MFITSAVATD TKAQIGELAG
      181 IAVGSAVCIT SIFAGPISGG SMNPARTLGP ALASSYYKGI WVYVVGPVTG TLLGAWSYNF
      241 IRVTDKPIQA ISPRSFSLKL RRMKSDNERQ GDDDEVRRTG ILDFDELSSS TSSVPRYWRR
      301 WLKPNDHKIC VKVDKETGHR FGKEHPLPDK ENTKVPVYAL AQLIGSISAS FTVSVLLHPI
      361 KHVGTTSPSG SELQALVTEI IVTFSMMFIT SAVATDTKAI GELAGIAVGS AVCITSIFAG
      421 PISGGSMNPA RTIGPALASA YYNGIWIYMV GPVIGALLGA YGLTASFG
//
LOCUS       FvNIP2-3       196 aa
DEFINITION  FvNIP2-3     196 aa
TITLE       FvNIP2-3
ORIGIN

        1 MATKEPTELR NETNEFDFQK SSVFEQHYPP GFLKKVVADI IATFLLVFVT CGSAALSASD
       61 EHKVSKLGAS IVGGLIVTAM IYAVGHISGA HMNPAVTLAF AAVRHFPWKQ VPIYAVAQLT
      121 GSISASFTLS ILLHPIKHVG TTSPSGSDIQ ALIVEIVMTF SMMFITSAVA TDTKAIGELA
      181 GIAVGSAVCI TSIFAG
//
LOCUS       FvNIP2-4       272 aa
DEFINITION  FvNIP2-4     272 aa
TITLE       FvNIP2-4
ORIGIN

        1 MARTELVSVE NPIVEHPFYP PGFLKKVVAE IIATFLLVFV TCGSAALSAS DERKVSKLGA
       61 SMTGGLIVTV MIYAVGHISG AHMNPAVTLA FATFRHFPWK QVPVYALAQL IGSISASFAV
      121 SALLHPIKHV GTTSPSGSEL QALVTEIIVT FSMMFITSAV ATDTKAIGEL AGIAVGSAVC
      181 ITSIFAGPIS GGSMNPARTI GPALASAYYN GIWIYMVGPV IGALLGAWSY SFIRVNDKPV
      241 QASPPRSLSL QLRRIKSDVQ AASICKDPLD FA
//
LOCUS       FvNIP2-5       282 aa
DEFINITION  FvNIP2-5     282 aa
TITLE       FvNIP2-5
ORIGIN

        1 MATKEPTELR NETNEFDFQK SSVFEQHYPP GFLKKVVAEI IATFLLVFVT CGSAALSASD
       61 EHKVSKLGAS IVGGLIVTAM IYAVGHISGA HMNPAVTLAF AAVRHFPWKQ VPIYAVAQLT
      121 GSISASFTLS MLLHPIKHVG TTSPSGSDIQ ALIIEIVMTF SMMFITSAVA TDTKAIGELA
      181 GIAVGSAVCI TSIFAGPISG GSMNPARTLG PALASSYYKG IWVYVVGPVI GTLLGAWSYN
      241 FIRVTDKPIQ AISPRSFSFK LRRMKSDNDR QVVTCTDPLD SA
//
LOCUS       FvNIP3-1       291 aa
DEFINITION  FvNIP3-1     291 aa
TITLE       FvNIP3-1
ORIGIN

        1 MKGLYEEAKP SPQNSNSAST SGHSKEDDPE IGSNALSNSA DHHVLKNSTF CFPLEINLNL
       61 LRAVVAEMVG TFILMFCVCG IIASTQLLRG EVGLMEYAST AGLTVVVVIF SIGTISGAHV
      121 NPAVTVAFAT LGHFPWSRVP LYTMAQTLGS VLAALVGKLV YGIKSDLMLT KPVQSCVSAF
      181 WVELIATFIV LFLAASMIHH AQAVGPLSGF VLGIAIGLAV LITGPVSGGS LNPARSLGPA
      241 IVSWNFKDIW IYVCAPTIGA VSGALLFQVL KLRVPPSNPS LTSSPDTCRL G
//
```

```
LOCUS       FvNIP3-2       306 aa
DEFINITION  FvNIP3-2     306 aa
TITLE       FvNIP3-2
ORIGIN

        1 MDNSNGNEEV PSAPSTPVTP GTPGAPLFGG FKPDHRSSGI GRKSLLKSCS KCFTVDDWAL
       61 EEGALPKAFC ALPPPPVSLA RKVGAEFLGT FILIFAGTAT AIVNQKTEGT ESLVGLAAST
      121 GLAVMVVILS TGHISGAHLN PAVTIAFAAL KHFSWKLVPV YIGAQVLASM CSAFALKVIF
      181 HPIMGGGVTV PSGSYGQAFA LEFIISFNLM FVVTAVATDT RAVGELAGIA VGATVMLNIL
      241 IAGETTGASM NPVRTLGPAI AANNFKAIWV YLTAPVLGAL CGAGTYTAVK LPEEEGEKPL
      301 TRSFRR
//
LOCUS       FvNIP3-3       350 aa
DEFINITION  FvNIP3-3     350 aa
TITLE       FvNIP3-3
ORIGIN

        1 MPEYEPGTPP VSAPPTPGTP APLFSGLVGR VDSLSYDRKS MPRCNSCLPV NAPTWGQPHT
       61 CFTDFPSPDV SLTRKIGAEF VGTFILMFAA TAGPIVNQKY NGAESLIGNA ACAGLGVMIV
      121 ILSTGHISGA HLNPSLTIAF ACLRHFPWTQ VPAYIAAQVS ASICACFALK GVYHPYMSGG
      181 VTVPSVSDGQ AFALEFIITF NLLFVVTAVA TDTRAVGELA GLAVGATVML NILIAGPSSG
      241 GSMNPVRTLG PAVAAGNYRA LWIYLVAPTL GALAGAGTYT AVKLQGDEIG APARECWCAC
      301 SLSMSIISEK VLCRRWNVFF ENLDGEGSDA VGCRDTATSP VKLVQQAQCR
//
LOCUS       FvNIP5-1       280 aa
DEFINITION  FvNIP5-1     280 aa
TITLE       FvNIP5-1
ORIGIN

        1 MELHVYIGLK RLRVVGKAGD VLYMITRIMI ITNSRHSNGM YLQVGAEFLG TFILIFAAAA
       61 GPIVNQKYDG AETLLGNAAC SGLAVMVVVF STGHISGAHL NPSVTVAFAA FRHFPWSQVP
      121 LYITAQVSAS ISASFALKSV FHPFMNGGVT VPSVGNGQAF ALEFIVTFIL MFVIIAVATD
      181 TRAVGELAGV AIGTAVLLDI LIAGPTTGAS MNPARTLGPA IAAGHYKSLW IYLVAPTLGA
      241 LAGAAAYTAV KLPEIPQKTQ IQIHDDSNGE ATPQPRVHFP
//
LOCUS       FvPIP1-1       287 aa
DEFINITION  FvPIP1-1     287 aa
TITLE       FvPIP1-1
ORIGIN

        1 MEGKEEDVRL GANKFSERQP IGTSAQTDKD YKEPPPAPLF EPGELQSWSF WRAGIAEFVA
       61 TFLFLYITIL TVMGVNRAPN KCASVGIQGI AWAFGGTIFA LVYSTAGISG GHINPAVTFG
      121 LFLARKLSLT RAVFYIVMQT LGAVAGAAVV KGFEGNTRYE VFGGGANSVN HGYTKGDGLG
      181 AEIVGTFVLV YTVFSATDAK RNARDSHVPI LAPLPIGFAV FLVHLATIPI TGTGINPARS
      241 LGAAIIYNKE HAWDHHWIFW VGPFIGAALA ALYHQIVIRA IPFKTRD
//
LOCUS       FvPIP1-2       290 aa
DEFINITION  FvPIP1-2     290 aa
TITLE       FvPIP1-2
ORIGIN

        1 MEAKEEDVSL GANKYPERQP IGTAAQSQDD GKDYKEPPPA PLFEPGELTS WSFYRAGIAE
       61 FIATFLFLYI TVLTVMGVLK GPTKCQTVGI QGIAWAFGGM IFALVYCTAG ISGGHINPAV
      121 TFGLFLARKL SLTRAVFYII MQTLGAIAGA AVVKAFENKN HFFELNGGGA NSVAHGYTKG
```

```
      181 SGLGAEIIGT FVLVYTVFSA TDAKRSARDS HVPILAPLPI GFAVFLVHLA TIPITGTGIN
      241 PARSLGAAII YNKKHAWDDH WIFWVGPFIG AALAALYHVV VIRAIPFKNK
//
LOCUS       FvPIP1-3       286 aa
DEFINITION  FvPIP1-3     286 aa
TITLE       FvPIP1-3
ORIGIN

        1 MEGREEDVKV GANRFPERQP LGTSAQTHKD YKEPPPAPLF EPHELVSWSF YRAGIAEFVA
       61 TFLFLYITVN TVMGVNRAPS KCASVGIQGI AWAFGGMIFA LVYCTAGISG GHINPAVTFG
      121 LFLARKLSLT RAVFYIIMQT LGAIAGAGVV KGFQKTQYEL LGGGANVVSH GYTKGDGLGA
      181 EIVGTFVLVY TVFSATDAKR KARDSHVPVL APLPIGFAVF LVHLATIPIT GTGINPARSL
      241 GAAIIYDKAH AWDDQWIFWV GPFIGAALAA FYHMVVIRAI PFHSRA
//
LOCUS       FvPIP2-1       281 aa
DEFINITION  FvPIP2-1     281 aa
TITLE       FvPIP2-1
ORIGIN

        1 MAKDVEGAEH GEFAAKDYHD PPPTPLFDAD ELTKWSFYRA VIAEFIATLL FLYITVLTVI
       61 GYKSQSATDQ CGGVGILGIA WAFGGMIFVL VYCTAGISGG HINPAVTFGL FLARKVSLLR
      121 AVLYIVAQSL GAICGVGLVK AFQKTLYDEY GGGANTLAPG YSKGTGLAAE IIGTFVLVYT
      181 VFSATDPKRN ARDSHVPVLA PLPIGFAVFM VHLATIPITG TGINPARSFG AAVIYNQDKA
      241 WDDHWMFWLG PFIGAAIAAF YHQFVLRAGA IKALGSFRSN A
//
LOCUS       FvPIP2-2       285 aa
DEFINITION  FvPIP2-2     285 aa
TITLE       FvPIP2-2
ORIGIN

        1 MAKDVEVAER GSFSAKDYQD PPPAPLFDSV ELTKWSFYRA VIAEFIATLL FLYITVLTVI
       61 GYKSQIDLGG DACGGVGILG IAWAFGGMIF ILVYCTAGIS GGHINPAVTF GLFLARKVSL
      121 VRAVMYIVAQ SLGAIAGVGL VKAFQKSYYT KYGGGANELA EGYSTGVGLA AEIIGTFVLV
      181 YTVFSATDPK RSARDSHVPV LAPLPIGFAV FMVHLATIPI TGTGINPARS LGAAVIYDKK
      241 KAWDDQWIFW VGPFIGAAIA AFYHQFILRA GAVKALGSFR SNPHN
//
LOCUS       FvPIP2-3       348 aa
DEFINITION  FvPIP2-3     348 aa
TITLE       FvPIP2-3
ORIGIN

        1 MGRDIEVGGF AAKDYQDPPP TPLIDPEEFG KWSFYRAIIA EFVATLLFLY ITVLTVIGYK
       61 SQSDTLKGGD QCGGVGILGI AWAFGGMIFV LVYCTAGISG GHINPAVTFG LFLARKVSLP
      121 RAVLYIVAQC LGAICGCGLV KSFQSALYSN YGGGANGLAD GYSEGTGLAA EIIGTFVLVY
      181 TVFSATDPKR NARDSHVPVL APLPIGFAVF MVHLATIPIT GTGINPARSF GAAVIYNNEK
      241 AWDDHWIFWV GPFIGAAIAA LYHQNILRAG AAKASGSFRS SSNPLSLSTR TQPPENNTRR
      301 PPKLISTIDF EDDLDAKEFD GEGWGVEGGV IERCWWWLSS SHVVGSSD
//
LOCUS       FvPIP2-4       287 aa
DEFINITION  FvPIP2-4     287 aa
TITLE       FvPIP2-4
ORIGIN

        1 MGKDIEVGPQ GGFSAKDYND PPPTPLIDAE EFGKWSFYRA IIAEFIATLL FLYITVLTVI
```

```
      61 GYKSQSDTLK GGDQCGGVGI LGIAWAFGGM IFVLVYCTAG ISGGHINPAV TFGLFLARKV
     121 SLPRAVLYIV AQSLGAICGC GLVKSFQSAL YTNYGGGANG LADGYSKGTG LAAEIIGTFV
     181 LVYTVFSATD PKRNARDSHV PVLAPLPIGF AVFMVHLATI PITGTGINPA RSFGAAVIYN
     241 NEKAWDDHWI FWVGPFIGAA IAALYHQYIL RAGAVKALGS FRSSSNI
//
LOCUS       FvPIP2-5       284 aa
DEFINITION  FvPIP2-5     284 aa
TITLE       FvPIP2-5
ORIGIN

       1 MAKDIEVGGF AAKDYHDPPP TPLIDPEEFG KWSFYRAIIA EFIATLLFLY ITVLTVIGYK
      61 SQSDTLKGGD QCGGVGILGI AWAFGGMIFV LVYCTAGISG GHINPAVTFG LFLARKVSLP
     121 RAVMYIVAQS LGAICGCALV KSFQSALYTN YGGGANGLAD GYNKGTGLAA EIIGTFVLVY
     181 TVFSATDPKR NARDSHVPVL APLPIGFAVF MVHLATIPIT GTGINPARSL GAAVIYNNEK
     241 AWDDHWIFWV GPFIGAAIAA MYHQYILRAG AVKALGSFRS SSNI
//
LOCUS       FvPIP2-6       284 aa
DEFINITION  FvPIP2-6     284 aa
TITLE       FvPIP2-6
ORIGIN

       1 MLKEVSEEGL KVHEEQHGKD YVEPPPVPLL ELGELQRWSF YRAVIAEFVA TLLFLYVTVA
      61 TVIGHKKEAV QCDGVGVLGI AWAFGGMIFV LVYSTAGISG GHINPAVTFG LLVARRVSLL
     121 RAVAYTVAQC LGGICGVGLV KALMKHYYLT YGGGANSVAP DYTKGAALGA ETVGTFLLVY
     181 TVFSATDAKR SARDSHVPVL APLPIGFAVF MVHLATIPIT GTGINPARSF AAAVVYNNDK
     241 VWDDQWIFWV GPFLGALIAA AYNQHVLRAS SSKAFGSFYI NTTN
//
LOCUS       FvPIP2-7       281 aa
DEFINITION  FvPIP2-7     281 aa
TITLE       FvPIP2-7
ORIGIN

       1 MSKEVSEEAQ AHHHGKDYVD PPPAPLFDTD ELKRWSFYRA LIAEFVATLL FLYITVATVI
      61 GHKHQTGPCD GVGLLGIAWA FGGMIFVLVY CTAGISGGHI NPAVTFGLFL ARKVSLIRAV
     121 SYMIAQCLGA IAGVALVKAF QSHYFTTLGG GTNSVADGYS KGTALGAEII GTFVLVYTVF
     181 SATDPKRSAR DSHVPVLAPL PIGFAVFIVH LATIPITGTG INPARSFGAA VIYNNEKVWD
     241 EHWIFWVGPF IGALAAAAYH QYILRAAAIK ALGSFRSNPS N
//
LOCUS       FvSIP1-1       351 aa
DEFINITION  FvSIP1-1     351 aa
TITLE       FvSIP1-1
ORIGIN

       1 MGAMKAAVGD AVVTFMWVFC ASTLGLMTSL IANVIGVQGF WASFGITTAL VFFLVLVFNM
      61 IGDALGGASF NPTGTASFYA AGVGADTLFS MALRFPAQTL GAVGGALAIK EVVPAQYNHM
     121 IGGPSLKVDL HTGAIAEGLL TFLITFLVLV IILKGPRSSL LKSLLLAVVT VALVVAGSVY
     181 TGPSMNPANY AETERVSAYD DADWAGCPDS CRPTSGYLVY LGTNLVSWCF RKQPTIARSS
     241 AEFEYRSLSH ASAETTWLAF LLNELGAHIE FLILLYCDNL NATYMASNYV FPTRTKHIEL
     301 DYHFVCEKVA FGSHRTLSHQ LISRQIFSLN PSISTVMCFL LANLFVQARQ V
//
LOCUS       FvSIP1-2       241 aa
DEFINITION  FvSIP1-2     241 aa
TITLE       FvSIP1-2
ORIGIN
```

```
        1 MGNLIKAAVG DAILTSLFVF VAPIMRVFTA ILASFLGFQA RSLIGRFITT IHATVLVLIF
       61 SMIGKLLGGA SFNPSTTVSF YAAGLNPGTS LISMAVQFPA QAVGGAGGAK AILQLMPEKY
      121 EQFVKGPSLK VDLKSGAIAE GLFTFSLCFS LLWVMLKGPK NLILQIWLVK VATVGLAIVG
      181 SGYTGPSMNP ANAFGWAYVN NWHNTWELFF VYWIGPLVGA VLAARVFKIL FPPPETKEKK
      241 A
//
LOCUS       FvSIP1-3       307 aa
DEFINITION  FvSIP1-3     307 aa
TITLE       FvSIP1-3
ORIGIN

        1 MASFFSHNLA WFHVLVQVSP ILDTNEGIEE RERDQDKKPG AEIFSPQNNF KAEDFHSQSR
       61 RENSEKMGSM IKAALGDAIL TSMWVFSAPS MGVFTTIIAS FLGVQARSLT GLFITTILAT
      121 TFVLTFSLIG KLLGGASFNP STTASFYAAG LSPGTSLISM AVRFPAQAAG GVGGAKAILQ
      181 VMPSKFKHFV KGPSLKVDVK SGAVAEGLFT FSLCFALLWI ILRGPKNPIV QLWLLSVATV
      241 GLVVVGGGYT GPSMNPANAF GWAYVNNWHN TWELFFVYWI GPLVGAILAA RVFKVLFPPP
      301 EPKEKKA
//
LOCUS       FvSIP2-1       232 aa
DEFINITION  FvSIP2-1     232 aa
TITLE       FvSIP2-1
ORIGIN

        1 MGRIGLLVSD FIISFMWVSS GVFVKKFVHN VLGFSHEPTA EILKSALSIV NMFFFAFLAK
       61 LTKGGTYNPL TVFSDAITGD FSRFLFTVAA RVPAQVLGSV AGVRFIMGTF PGIGLGPRLT
      121 VDIHKGALTE GLLAYAIVTI SLGLSRNIPG SFFRKTWISS VSKLTLHILG SDLTGGCMNP
      181 ASVMGWAYAR GDHITKEHIL VYWLAPIEAT LLAVWTSRLL FPPKEDKKAK VE
//
LOCUS       FvTIP1-1       254 aa
DEFINITION  FvTIP1-1     254 aa
TITLE       FvTIP1-1
ORIGIN

        1 MPISSIAVGS PSEFGSADAL RAALAEFIST LIFVFAGSGS GVAFAELTDN GATTPAGLVA
       61 AAVAHAFALF VAVAIAANIS GGHVNPAVTF GLFLGGNITL IRGILYWIAQ LLGSVVACLL
      121 LKLATGGLTI SAFSLSTGVG AWNAVVFEIV MTFGLVYTVY ATAVDKRSAG NISIIAPIAI
      181 GFIVGANILA GGAFDGASMN PAVSFGPAVV SWSWASHWVY WLGPLLGAAI AAIIYDWILI
      241 APTTHEPLPT TDYS
//
LOCUS       FvTIP1-2       254 aa
DEFINITION  FvTIP1-2     254 aa
TITLE       FvTIP1-2
ORIGIN

        1 MPISSIAVGS PSEFGSVDAL RAALAEFIST LIFVFAGSGS GVAFAELTDN GATTPAGLVA
       61 AAIAHAFALF VAVAIAANIS GGHVNPAVTF GAFLGGNITL VRSILYWIAQ LLGSVVACLL
      121 LKFATGGMTI SAFSLSTGVG VWNAVVFEIV MTFGLVYTVY ATALDKRSAG NISIIAPIAI
      181 GFIVGANILA GGAFDGASMN PAVSFGPAVV SWSWESHWVY WLGPFLGAGI AALIYDWIFI
      241 GPATHEPLPT TDYS
//
LOCUS       FvTIP1-3       253 aa
DEFINITION  FvTIP1-3     253 aa
TITLE       FvTIP1-3
```

```
ORIGIN

        1 MPIQNIAVGR PEDAYHPDAL KAGLAEFIST LIFVFAGEGS GMAFNKLTDD GATTPAGLVA
       61 AALAHAFGLF VAVSVAANIS GGHVNPAVTF GAFVGGNISL LRGILYWIAQ LLGSTVACLL
      121 LKFVTNGQTT SAFALSSGVG VWNAFVFEIV MTFGLVYTVY ATAVDPKRGS VGTIAPIAIG
      181 FIVGANILAG GAFDGASMNP AVSFGPALVS WSWENHWVYW AGPLVGGGLA GLVYEFFFIN
      241 NSGHEPLPGG EYA
//
LOCUS       FvTIP1-4       252 aa
DEFINITION  FvTIP1-4     252 aa
TITLE       FvTIP1-4
ORIGIN

        1 MAISRIAIGA PGEASHPDAI RAAFAEFFSM LIFVFAGEGS GMAYSKLTDN GSATPSGLIA
       61 ASLSHAFALF VAVSVGANIS GGHVNPAVTF GAFLGGNITL LRASLYWIAQ LLGSVVACLL
      121 LKFATGGLET AAFSLSSGVS IWNALVFEIV MTFGLVYTVY ATAVDPKKGN VGIVAPIAIG
      181 LIVGANILAG GAFDGASMNP AVSFGPAVVS WSWTHHWVYW AGPMIGAAIA ALVYDNLFIG
      241 DGAHEPLPHN DF
//
LOCUS       FvTIP2-1       248 aa
DEFINITION  FvTIP2-1     248 aa
TITLE       FvTIP2-1
ORIGIN

        1 MAGIAFGRFD DSFSLGSFKA YLAEFISTLL FVFAGVGSAI AYNKLTSNAA LDPAGLVAIA
       61 ICHGFALFVA VSVGANISGG HVNPAVTFGL ALGGQITILT GIFYWIAQLA GAIVACFILK
      121 FVTGGLTIPI HTVSAGVGAI EGVVFEIIIT FALVYTVYAT AADPKKGAIG TIAPIAIGFI
      181 VGANILAAGP FSGGSMNPAR SFGPAVASGD FTDNWIYWVG PLIGGGLAGL IYGNVYIHSE
      241 HQPLVSEY
//
LOCUS       FvTIP2-2       317 aa
DEFINITION  FvTIP2-2     317 aa
TITLE       FvTIP2-2
ORIGIN

        1 MGVPPLFTIG FVLRFVLQLA SILLQIESLW WRRETVYRSS SLHFGTTHDL LVSSGGGSGS
       61 GGGVSDQTMV KIGFGSFGDS FSVCSLKSYL AEFIATLLFV FAGVGSAIAY DKLTTDAALD
      121 PAGLVAVAVA HAFALFVGVS VAANISGGHL NPAVTFGLAI GGNITILTGI FYWIAQLLGS
      181 IVACFLLKFV TSESVPTHGL ASGVGAIEGV VFEIIITFGL VYTVYATAAD PKKGSLGTIA
      241 PIAIGFIVGA NILAAGPFSG GSMNPARSFG PAVVSGNFAD NWIYWVGPLV GGGLAGLVYG
      301 DIFIGSYSQV PISEDYA
//
LOCUS       FvTIP3-1       260 aa
DEFINITION  FvTIP3-1     260 aa
TITLE       FvTIP3-1
ORIGIN

        1 MPQPYRRYEF GRADEATHPD SIRATLAEFI ATFIFVFAGE GSILALGKIY KDSDTSVAEL
       61 IAIALAHAFA LFSAISASIN ISGGHVNPAV TFGALIGGRI SVVRAFYYWV AQLLGAIVAS
      121 LVLRLVTNGM RTVGFNVAPG VGEWHGLVLE IVMTFGLVYT VYATAIDPKR GSLGTIAPLA
      181 IGFIVGANIL VGGPFDGASM NPARAFGPAL VGWRWKNHWI YWLGPFIGGG LAGLIYEYMV
      241 IPTEPPHTHH THQPLAPEDY
//
LOCUS       FvTIP4-1       248 aa
```

```
DEFINITION  FvTIP4-1     248 aa
TITLE       FvTIP4-1
ORIGIN

        1 MAKIAFGSTS EAAKPEVWRA LIVEFVTTFL FIFAGVGSAM ATDKLGADTT VALFFIAITH
       61 ALVVAVMISA GHISGGHLNP AVTLGLLAGG HITLFRSVLY WIDQLLAAAA SCYLLKYLTG
      121 GLTTPIHSLA SGVGFSQGVI WEIILTFSLL FTVYATMVDP KKGSLDGLGP TLTGFVVGAN
      181 ILAGGAFSGA SMNPARSFGP ALVSWDWTDH WVYWVGPLIG GGLAGFIYES FFIERPRSYQ
      241 PIPVADDA
//
LOCUS       FvTIP5-1       253 aa
DEFINITION  FvTIP5-1     253 aa
TITLE       FvTIP5-1
ORIGIN

        1 MARIALTARF QQVITPNALR SYLAEFISTF FFVFAVIGSL MSSRKLMPDA ASDPASLVLV
       61 AMTNAFALAS AVYISASTSG GHVNPAVTFA MAVGGHISVP IALIYWICQM VAAVMACLLL
      121 RVTVVGQHVP TYAITEEMTG FGASVLEGVL TFALVYTVYA AGDPRHGALR AMGPLAIGLM
      181 AGANVLATGP FSGGSMNPAC AFGSAVITGT FKNQAVYWVG PLIGAAVAGL LYDNVVYPVQ
      241 DSLTRNNADG IAV
//
LOCUS       FvXIP1-1       283 aa
DEFINITION  FvXIP1-1     283 aa
TITLE       FvXIP1-1
ORIGIN

        1 MELIIHPHED NKLPRVYGTG TDNSSVETNS LGQRFLSSIG AHEYFSPEMW RAALTEFVAT
       61 ACLMFTLTSS IIACLDSHEV DPKLLVPLAV FVIAFLFLMV TVPLSGGHMS PVFTFIAALK
      121 GVITLARASI YMLMQCVGSI VGFLIIKTVM DHNAAQKYSL GGCTTHGVGT HTALVLEFVC
      181 TFVVLFVGVT VAFDKNRCKE LGLAMVCLVV AGAMALAVFV SITVTGKSGY AGVGLNPARC
      241 LGPALLQGGI LWDGHWVFWV GSILACSVYY CFSLSLPKEG LTL
//
LOCUS       FvXIP1-2       614 aa
DEFINITION  FvXIP1-2     614 aa
TITLE       FvXIP1-2
ORIGIN

        1 MADTTSSFAS NPVSELNSWN RSSLSGCAAQ GARVGATRQE TAKLGDGSPG AGDRLGAASQ
       61 TIFTVIALYL VVMLCADLIK SIMLIKRNPA CWFLLDLDNR GSFGSVPLIW VRSYDCLKIY
      121 GYLSDFCRSG MSEIWMKTDS EIRASQHFVV HVEAAHDVEI SPSHDSYPSL FPSSMFTPDL
      181 SFAFQSSLCF PHATTSLPIG RSLLCPVTVL SVCSLESLGI SLPESCLAVV DEPLNDFGGG
      241 FLGIYCVICG RDLFCLFLQI VEKLTASAHG GLACVRDDMM AVVFTLVLGG SAPCLINFIG
      301 MPGYSLALAT ATGWLLCFTR SEHWKTEEGK KSSRPSSLSE VLGLRELFCP QVWRASLAEL
      361 VGTAVLVFAI DTIVISSIET NTSSPNLLLS ILVAIIVAIL LIATNPVSGG HINPVITFSA
      421 ATVGLISFSR AAVYIFMQCV GGVLGALALK AVVNSSLQDT FSLGGCTLTV VAPGPQGPVT
      481 IGIETGQALW LEVICTFIFL FASIWIAFDH RQAHPMGKVM VLSIVGIVVG ILVFISTSVT
      541 TVKGYAGAGM NPARCLGPAL VRGGHLWTGH WVFWVGPTIA CLAFYLYVKL IPRQHFHVEG
      601 YKHDIFDYMK NLPK
//
LOCUS       GmNIP1-1       268 aa
DEFINITION  GmNIP1-1     268 aa
TITLE       GmNIP1-1
ORIGIN
```

```
        1 MEENGGNIHA DSTFCGSPAV VQVIQKVIAE LIGTYFLIFA GCCSVIINNA EETKGRITFP
       61 GICLVWGFSV TILVYSLAHV SGAHFNPAVT LSFAIYRHFP LRLAYIKSTV PLYFIAQVLG
      121 SFLASGTLYL LFEVNEKTYF GTIPSGSYIQ SLVFEILTSF LLMFVVCAVS TDNRAIGKLG
      181 GIAVGMTIIV NVFIAGPISG ASMNPARSLG PALVMWVYNG IWIYVVGPFV GAILGATCYN
      241 LIRYTDKPLR EIGASSKIFK TSACTSAT
//
LOCUS       GmNIP1-2       215 aa
DEFINITION  GmNIP1-2     215 aa
TITLE       GmNIP1-2
ORIGIN

        1 MAEVIGTYFV VFAGCGSVAV NKIYGSVTFP GVCVTWGLIV MVMIYSLRRI SGAHFNPAVT
       61 ITLAIFRRFS YKEVPLYIFA QLLGSILASG TLALMLDVTP KAYFGTVPVG SNGQSLVAEI
      121 IITFLLMFVI SAVSTDDRAV GDFAGVAVGM TIMLNVFIAG PVSGASMNPA RSIGPALIKH
      181 VYKGLWVYVV GPVVGSIAGA LAYYFLRSID KSSSE
//
LOCUS       GmNIP1-3       261 aa
DEFINITION  GmNIP1-3     261 aa
TITLE       GmNIP1-3
ORIGIN

        1 MAAKSEGIQE EMPSMEEGVS SPSPSRTCNV SHNCCSNHVV ALAQKVFAEV IGTYFVVFAG
       61 CGSVAVNKIY GSVTFPGVCV TWGLIVMVMI YSLRHISGAH FNPAVTITLA IFRRFSYKQV
      121 PLYIFAQLLG SILASGTLAL MLDVTPKAYF GTVPVGSNGQ SLVAEVIITF LLMFVISAVS
      181 TDDKAVGDFA GVAVGMTIML NVFIAGPVSG ASMNPARSIG PALIKHVYQG LWIYVVGPIV
      241 GSIAGALAYN FLRSPYKPPS E
//
LOCUS       GmNIP1-4       243 aa
DEFINITION  GmNIP1-4     243 aa
TITLE       GmNIP1-4
ORIGIN

        1 MEPSDSFVSV PFLQKLVAEV VGTYFLIFAG CASVVVNKNN DNVVTLPGIA IAWGLVVTVL
       61 VYTVGHISGA HFNPAVTIAF ASTRRFPLMQ VPAYVAAQLL GSTLASGTLK LLFMGKHDQF
      121 SGTLPNGTNL QAFVFEFIIT FLLMFVISGV ATDNRAVTSL TLLPLLKFVH TSWPVTGASM
      181 NPVRSLGPAI VHGEYRGIWI YLLAPVVGAI AGALVYNTIR YTDKPLREIT KSASFLKGRG
      241 GST
//
LOCUS       GmNIP1-5       271 aa
DEFINITION  GmNIP1-5     271 aa
TITLE       GmNIP1-5
ORIGIN

        1 MADYSAGTES QEVVVNVTKN TSETIQRSDS LVSVPFLQKL VAEAVGTYFL IFAGCASLVV
       61 NENYYNMITF PGIAIVWGLV LTVLVYTVGH ISGGHFNPAV TIAFASTRRF PLIQVPAYVV
      121 AQLLGSILAS GTLRLLFMGN HDQFSGTVPN GTNLQAFVFE FIMTFFLMFV ICGVATDNRA
      181 VGELAGIAIG STLLLNVIIG GPVTGASMNP ARSLGPAFVH GEYEGIWIYL LAPVVGAIAG
      241 AWVYNIVRYT DKPLSEITKS ASFLKGRAAS K
//
LOCUS       GmNIP1-6       270 aa
DEFINITION  GmNIP1-6     270 aa
TITLE       GmNIP1-6
ORIGIN
```

```
        1 MDENSATNGT HEVVLDVNRD VSRTTQASRS CVNVSFLQKL VAEVVGTYFL IFAGSASVVV
       61 NKNNNNVVTL PGISIVWGLV VMVLVYSVGH ISGAHFNPAV TIAFASTKRF PLKQVPVYVV
      121 AQVVGSTLAS GTLRLLFSGK EAQFSGTLPS GSNLQAFVIE FLITFFLMFV VSGVATDNRA
      181 IGELAGIAVG STVLLNVMFA GPITGASMNP ARSIGPAIVH KEYRGIWIYL VSPTLGAVAG
      241 AWVYNSIRYT DKPLREITKS ASFLKGVASR
//
LOCUS       GmNIP1-7       274 aa
DEFINITION  GmNIP1-7     274 aa
TITLE       GmNIP1-7
ORIGIN

        1 MDENSATNGT HEVILDVNKD VSRTTQPSRS CVNVSFLQKL VAEVVGTYFL IFAGCASVVV
       61 NKNNNNVVTH PGISIVWGLV VMVLVYSVGH ISGAHFNPAV TIAFASTRRF PLKQVPVYVV
      121 AQVVGSTLAS ATLRLLFSGK ETQFSGTLPS GSNLQAFVIE FLITFFLMFV ISGVATDDRA
      181 IGELAGIAVG STVLLNVMFA GPITGASMNP ARSIGPAILH NEYRGIWIYI VSPTLGAVAG
      241 TWVYNTIRYT DKPLREITKS TSFLKGVGRS GSSR
//
LOCUS       GmNIP1-8       273 aa
DEFINITION  GmNIP1-8     273 aa
TITLE       GmNIP1-8
ORIGIN

        1 MSVVADNSAN NGSHQVVLNV NGDAPKKCDD SANQDCVPLL QKLVAEVVGT YFLIFAGCAS
       61 VVVNLDKDKV VTQPGISIVW GLTVMVLVYS VGHISGAHFN PAVTIAHATT KRFPLKQVPA
      121 YVIAQVVGAT LASGTLRLIF NGKNDHFAGT LPSGSDLQSF VVEFIITFYL MFVISGVATD
      181 NRAIGELAGL AVGSTVLLNV MFAGPITGAS MNPARSLGPA IVHHEYRGIW IYLVSPTLGA
      241 VAGTWAYNFI RYTNKPVREI TKSASFLKGS EAE
//
LOCUS       GmNIP1-9       267 aa
DEFINITION  GmNIP1-9     267 aa
TITLE       GmNIP1-9
ORIGIN

        1 MYTNNGSHQV VLNVNGDASK KCDDSSNQDC VPLLQKLVAE VVGTYFLIFA GCASVVVNLD
       61 KDKVVTQPGI SIVWGLTVMV LVYSVGHISG AHFNPAVTIA HATTKRFPLK QVPAYVIAQV
      121 VGATLASGTL RLIFNGKSDH FTGTLPGGSD LQSFVVEFII TFYLMFVISG VATDNRAIGE
      181 LAGLAVGSTV LLNVMFAGPI TGASMNPARS LGPAIVHNEY KGIWIYLVSP TLGAVAGTWA
      241 YNFIRYTNKP VREITKSASF LKGGEAE
//
LOCUS       GmNIP2-1       293 aa
DEFINITION  GmNIP2-1     293 aa
TITLE       GmNIP2-1
ORIGIN

        1 MEGTSSQSTF AFIPSTIETP SPSIPEISSS SSPGSLARIA QSYPPGFPRK VLAEIIGTFL
       61 LVFVGSGSAG LSKIDERMVS KLGASLAGGL IVTVMIYSIG HISGAHMNPA VSLAFTAVRH
      121 LPWPQLPFYI AAQLTGAISA SYTLRELLRP SNEIGGTSPA GSHIQALIME MVTTYTMVFI
      181 SMAVATDSNA TGQLSGVAVG SSVCIASIVA GPISGGSMNP ARTLGPAIAT SYYKGLWVYF
      241 VGPITGAVLA AWSYNVIRDT EHPGFPISLS SISSKVRQSI GGTEQKSDQR CLV
//
LOCUS       GmNIP2-2       295 aa
DEFINITION  GmNIP2-2     295 aa
TITLE       GmNIP2-2
ORIGIN
```

```
        1 MEGTTSQSTF TFIPSTIETP SPSIPEISSS SPSPGGSLAR VAQSYPPGFP RKVFAEVIGT
       61 FLLVFVGSGS AGLSKIDESM VSKLGASLAG GLIVTVMIYS IGHISGAHMN PAVSLAFTAV
      121 RHLPWPQLPF YVAAQLTGAI SASYTLRELL RPSDEIGGTS PAGSHIQALI MEMVSTYTMV
      181 FISMAVATDS NATGQLSGVA VGSSVCIASI VAGPISGGSM NPARTLGPAI ATSYYKGLWV
      241 YFVGPITGAV LAAWSYNVIR DTEHPGFPIS LSSISSKVRQ SIGGTEQKSD QRCLV
//
LOCUS       GmNIP3-1       304 aa
DEFINITION  GmNIP3-1     304 aa
TITLE       GmNIP3-1
ORIGIN

        1 MDNNEEIPST PATPGTPGAP LFGGFSNGRN NNSKKSLLKS CRCFSVEEWS LEDGGLPAVS
       61 CSLPLPSPPP VVPLARKIGA EFIGTFILMF AGTAAAIVNQ KTNGSETLIG CAATTGLAVM
      121 IVILATGHIS GAHLNPAVTI SFAALKHFPW KHVPMYIGAQ VLASICAGFA LKGVYHPFMS
      181 GGVTVPSGGY GQSFALEFII GFNLMFVVTA VATDTRAVGE LAGIAVGATV MLNILIAGPV
      241 SGGSMNPVRT LGPAVAANNY KAIWVYLVAP ILGALAGAGT YTAVKLPEED DDAKAKTSIS
      301 SFRR
//
LOCUS       GmNIP3-2       306 aa
DEFINITION  GmNIP3-2     306 aa
TITLE       GmNIP3-2
ORIGIN

        1 MNNEEVPSLP STSATPGTPG APLFGGLRFE KPNGSVVRKS SFLKSCKCFS VAEWTLEDGA
       61 MPRVSCSLPS PHIPLAKKIG AEFIGTFILM FAAIGTAIVN QKTHGSETLI GCAAANGLAV
      121 MIIIFSTGHI SGAHLNPAVT ISFAALKHFP WKNVPVYIGT QVLASVSAAF ALKVVFHPFM
      181 SGGVTVPSVG YGQAFATEFI VSFILMFVVT AVATDTRAVG ELAGIAVGAT VMLNILIAGP
      241 TTGSSMNPVR TLGPAIAANN YKGIWVYLIA PILGTLCGAG AYTVVKLPEE EATKTPSSAP
      301 NGSFRR
//
LOCUS       GmNIP3-3       263 aa
DEFINITION  GmNIP3-3     263 aa
TITLE       GmNIP3-3
ORIGIN

        1 MPESETGTPT AASVPATPDT PGGPLFTSLR VDSLSHERDS FAMARCKCLP TKGHICFTDF
       61 SVGVPLPNVS LTQKVGAEFV GTFILIFAAT AGPIVNNKYN GVETLMGNAA CAGLTVMFII
      121 LSIGHISGAH LNPSLTIAFA AFRHFPWAHV PAYIAAQVSA SICACYALKG NWWCYAVQVG
      181 ELAGIAVGAT VLLNILISGP TSGGSMNPVR TLGPAVAAGN YKHIWIYLVA PTLGALAGAG
      241 VYTLVKLRDN EAEPPRQVRS FRR
//
LOCUS       GmNIP4-1       340 aa
DEFINITION  GmNIP4-1     340 aa
TITLE       GmNIP4-1
ORIGIN

        1 MTDIFEKHQS SDSSNYASSS GLCEEDKEIG YRAATSKHRY VLANNSALKF IPIKIDLNCA
       61 RMVMAEVVGT FILMFCVCGI TASTRFQNGA VGLLEYAATA GLTVVVIIFS IGPISCAHVN
      121 PAVTIAFATI GQFPWLKVPV YIIAQTVGSM SATYVGSLVY GIKSDAMMTM PLQGCNSAFW
      181 VEVIATFIIM FLVAALTSES QSVGHLSGFV AGMAIGLAVL ITGPVSGGSM NPARSLGPAI
      241 LSWKFKNIWI YMVAPSGGAI AGAAMFRFLR LRDQHSSTLS SPNIIDVGRS IPFCSRRSGP
      301 MILLVENNWS LSYGRVEGFR QRYVLRTKGI SDGVYHKFPL
//
```

```
LOCUS       GmNIP4-2       282 aa
DEFINITION  GmNIP4-2     282 aa
TITLE       GmNIP4-2
ORIGIN

        1 MEYEVTAELV GTFILMFCVC GITASTRFQN GAVGLLEYAA IAGLTVVVII FSIGPISCAH
       61 VNPAVTIAFA TIGQFPWFKV PVYIIAQTVG SMSATYIGSL VYGIKSEAMM TMPLQGCNSA
      121 FWVEVIATFI IMFLIAALTS ESQSVGHLSG FVAGMAIGLA VLITGPVSGG SMNPARSLGP
      181 AILSWKFKNI WIYMVAPSGG AVAGAAMFRF LRLRDQHSSI LSSPNISDVG RSLPFCSRRS
      241 GPMILLVKKN WSSFSERVEG FRQRCVLRTK GISEGVYHKL PL
//
LOCUS       GmNIP5-1       277 aa
DEFINITION  GmNIP5-1     277 aa
TITLE       GmNIP5-1
ORIGIN

        1 MADSLSVNFD SSIKSEFSTE QAHKTTHEAK HSPSNIQKAI AEVVGTYILI FAGCGAALVN
       61 EKLPLTIVGI AMVSGLGLTV ATYSVGHVSG GHFNPAVTIA LAAVRKVQFK LMMGATLAPL
      121 TLKVLYHDKA DIGVTVTKYL SSTSDLEAIV WEFITTSILM LTIRGVATDH RGSKDLTGVA
      181 IGISVLINVI IAGPITGASM NPARSLGPAI VSGDYKNIWV YIISPILGAV SASTLYKFLE
      241 GRPSGGGIWS SMHGVLVQTS LRPLYPEKPS NYTLPCT
//
LOCUS       GmPIP1-1       284 aa
DEFINITION  GmPIP1-1     284 aa
TITLE       GmPIP1-1
ORIGIN

        1 MEGKEEDVRV GANRYGERQP IGTAAQAKDY REPPSAPLFE PGELSSWSFY RAGIAEFVAT
       61 FLFLYITVLT VMGVFKSKSK CSTVGIQGIA WAFGGMIFAL VYSTAGISGG HINPAVTFGL
      121 FLARKLSLTR AIFYIIMQCL GAICGAGVVK GFEPHLYERL GGGANTIAKG YTNSAGLGAE
      181 IVGTFVLVYT VFSATDAKRN ARDSHVPILA PLPIGFAVFL VHLATIPVTG TGINPARSLG
      241 AAIIFNKDQA WDDHWIFWVG PFIGAALAAL YHQIVIRAIP FSSK
//
LOCUS       GmPIP1-2       287 aa
DEFINITION  GmPIP1-2     287 aa
TITLE       GmPIP1-2
ORIGIN

        1 MEGRDEDVRV GANRYGERQP IGTAAQTQDA KDYREAPPAP LFEPRELTSW SFYRAGIAEF
       61 VATFLFLYVT VLTVMGVAKS PSKCSTVGVQ GIAWSFGGMI FALVYCTAGI SGGHINPAVT
      121 FGLFLARKLS LTRTVFYMIM QCLGAICGAA VVKGFQSNQY ERLGGGANTL SKGYSKGDGL
      181 GAEIVGTFIL VYTVFSATDA KRNARDSHVP ILAPLPIGFA VFLVHLATIP ITGTGINPAR
      241 SLGAALVYNK DQAWDNHWIF WVGPFIGAAL AALYHQIVLR AIPFKSK
//
LOCUS       GmPIP1-3       286 aa
DEFINITION  GmPIP1-3     286 aa
TITLE       GmPIP1-3
ORIGIN

        1 MEREEDVKVG AQKFSERQAL GTGAKSDKDY KEAPPAPLFE PGELKSWSFY RAGIAEFVAT
       61 FLFLYITVLT VMGVNRAPNK CSSVGIQGIA WAFGGMIFAL VYCTAGISGG HINPAVTFGL
      121 FLARKLSLTR AVFYIVMQCL GAICGAGVVK GFEGNARYEL FKGGANFVSH GYTKGDGLGA
      181 EIVGTFILVY TVFSATDAKR NARDSHVPIL APLPIGFAVF LVHLATIPIT GTGINPARSL
      241 GAAIIYNRDH AWDDHWIFWV GPFIGAALAA VYHQIVIRAI PFKTRG
```

```
//
LOCUS       GmPIP1-4       286 aa
DEFINITION  GmPIP1-4     286 aa
TITLE       GmPIP1-4
ORIGIN

        1 MEREEDVKVG AQKFSERQAL GTGAQGDKDY KEAPPAPLFE PGELKSWSFY RAGIAEFVAT
       61 FLFLYITVLT VMGVNRAPNK CSSVGIQGIA WAFGGMIFAL VDCTAGISGG HINPAVTFGL
      121 FLARKLSLTR ALFYIVMQCL GAICGAGVVK GFEGNARYEL FKGGANFVSH GYTKGDGLGA
      181 EIVGTFILVY TVFSATDAKR NARDSHVPIL APLPIGFAVF LVHLATIPIT GTGINPARSL
      241 GAAIIYNRDH AWDDHWIFWV GPFIGAALAA LYHQIVIRAI PFKTRG
//
LOCUS       GmPIP1-5       287 aa
DEFINITION  GmPIP1-5     287 aa
TITLE       GmPIP1-5
ORIGIN

        1 MESKEEDVRV GATKFSERQP IGTAAQGDKD YKEPPPAPLF EPGELKSWSF YRAGIAEFVA
       61 TFLFLYITIL TVMGVNRSPS KCASVGIQGI AWAFGGMIFA LVYCTAGISG GHINPAVTFG
      121 LFLARKLSLT RALFYIIMQC LGAICGAGVV KGFEGNARYE MFKGGANFVN SGYTKGDGLG
      181 AEIVGTFVLV YTVFSATDAK RNARDSHVPI LAPLPIGFAV FLVHLATIPI TGTGINPARS
      241 LGAAIIYNRD HAWDDQWIFW VGPFIGAALA AVYHQIVIRA IPFKTRA
//
LOCUS       GmPIP1-6       289 aa
DEFINITION  GmPIP1-6     289 aa
TITLE       GmPIP1-6
ORIGIN

        1 MESKEEDVNV GANKFSERQP IGTAAQGGGD KDYKEAPPAP LFEPGELKSW SFYRAGIAEF
       61 VATFLFLYIT ILTVMGVNRS PSKCASVGIQ GIAWAFGGMI FALVYCTAGI SGGHINPAVT
      121 FGLFLARKLS LTRALFYIIM QCLGAICGAG VVKGFEGNAN YELFKGGANF VNSGYTKGDG
      181 LGAEIVGTFV LVYTVFSATD AKRNARDSHV PILAPLPIGF AVFLVHLATI PITGTGINPA
      241 RSLGAAIIYN RDHAWDDQWI FWVGPFIGAA LAAVYHQIVI RAIPFKTRA
//
LOCUS       GmPIP1-7       289 aa
DEFINITION  GmPIP1-7     289 aa
TITLE       GmPIP1-7
ORIGIN

        1 MEGKEEDVSL GANKFSERQP IGTAAQSQDD GKDYTEPPPA PLFEPSELTS WSFYRAGIAE
       61 FVATFLFLYI TILTVMGVNR SSSKCATVGI QGIAWAFGGM IFALVYCTAG ISGGHINPAV
      121 TFGLFLARKL SLTRALFYMV MQVLGAIVGA GVVKGFEGKT FYGQHNGGAN FVAPGYTKGD
      181 GLGAEIVGTF ILVYTVFSAT DAKRSARDSH VPILAPLPIG FAVFLVHLAT IPITGTGINP
      241 ARSLGAAIIF NKDLGWDDHW IFWVGPFVGA ALAALYHQVV IRAIPFKSS
//
LOCUS       GmPIP1-8       289 aa
DEFINITION  GmPIP1-8     289 aa
TITLE       GmPIP1-8
ORIGIN

        1 MEGKEQDVSL GANKFPERQP IGTAAQSQDD GKDYQEPAPA PLVDPTEFTS WSFYRAGIAE
       61 FVATFLFLYI TVLTVMGVAG AKSKCSTVGI QGIAWAFGGM IFALVYCTAG ISGGHINPAV
      121 TFGLFLARKL SLPRAIFYIV MQCLGAICGA GVVKGFEGKT KYGALNGGAN FVAPGYTKGD
      181 GLGAEIVGTF ILVYTVFSAT DAKRSARDSH VPILAPLPIG FAVFLVHLAT IPITGTGINP
```

```
      241 ARSLGAAIIF NKDLGWDEHW IFWVGPFIGA ALAALYHQVV IRAIPFKSK
//
LOCUS       GmPIP2-1       285 aa
DEFINITION  GmPIP2-1     285 aa
TITLE       GmPIP2-1
ORIGIN

        1 MAKHDVEGGS FSAKDYHDPP PAPLIDAEEL TQWSFYRALI AEFIATMLFL YITVLTVIGY
       61 KSQSDVKAGG DVCGGVGILG IAWAFGGMIF ILVYCTAGIS GGHINPAVTF GLFLARKVSL
      121 IRAIMYMVAQ CLGAICGVGL VKAFQKAYYN RYGGGANELS EGYSTGVGLG AEIIGTFVLV
      181 YTVFSATDPK RNARDSHVPV LAPLPIGFAV FMVHLATIPV TGTGINPARS LGAAVMYNQQ
      241 KAWDDHWIFW VGPFIGAAIA AFYHQFILRA GAAKALGSFR SNPAI
//
LOCUS       GmPIP2-2       285 aa
DEFINITION  GmPIP2-2     285 aa
TITLE       GmPIP2-2
ORIGIN

        1 MAKHDVEGGS FAAKDYHDPP PAPLIDAEEL TQWSFYRALI AEFIATMLFL YITVLTVIGY
       61 KSQSDVKAGG DVCGGVGILG IAWAFGGMIF ILVYCTAGIS GGHINPAVTF GLFLARKVSL
      121 IRAIMYMVAQ CLGAICGVGL VKAFQKAYYN RYGGGANELS EGYSTGVGLG AEIIGTFVLV
      181 YTVFSATDPK RNARDSHVPV LAPLPIGFAV FMVHLATIPV TGTGINPARS LGAAVMYNQQ
      241 KAWDDHWIFW VGPFIGAAIA AFYHQFILRA SAAKALGSFR SNPTI
//
LOCUS       GmPIP2-3       285 aa
DEFINITION  GmPIP2-3     285 aa
TITLE       GmPIP2-3
ORIGIN

        1 MAKHDVEGGS FSAKDYHDPP PAPLIDAEEL TQWSFYRALI AEFIATLLFL YITVLTVIGY
       61 KSQSDVKAGG DVCGGVGILG IAWAFGGMIF ILVYCTAGIS GGHINPAVTF GLFLARKVSL
      121 IRAIMYMVAQ CLGAICGVGL VKAFQKAYYN RYGGGANELS EGYSTGVGLG AEIIGTFVLV
      181 YTVFSATDPK RNARDSHVPV LAPLPIGFAV FMVHLATIPV TGTGINPARS LGAAVMYNQQ
      241 KAWDDHWIFW VGPFIGAAIA AFYHQFILRA GAAKALGSFR SNPTI
//
LOCUS       GmPIP2-4       285 aa
DEFINITION  GmPIP2-4     285 aa
TITLE       GmPIP2-4
ORIGIN

        1 MAKHDVEGGS FSAKDYHDPP PAPLIDAEEL TQWSFYRALI AEFIATLLFL YITVLTVIGY
       61 KSQSDVKAGG DVCGGVGILG IAWAFGGMIF ILVYCTAGIS GGHINPAVTF GLFLARKVSL
      121 IRAIMYMVAQ CLGAMCGVGL VKAFQKAYYN RYGGGANELS EGYSTGVGLG AEIIGTFVLV
      181 YTVFSATDPK RNARDSHVPV LAPLPIGFAV FMVHLATIPV TGTGINPARS FGAAVMYNQK
      241 KAWDDQWIFW VGPFIGAAIA AFYHQFILRA SAAKAVGSFR SNPTI
//
LOCUS       GmPIP2-5       296 aa
DEFINITION  GmPIP2-5     296 aa
TITLE       GmPIP2-5
ORIGIN

        1 MSVFWQEGGM AKDVEVAERG SFSGKDYQDP PPAPLIDAEE LTKWSFYRAL IAEFIATLLF
       61 LYITVLTVIG YNHQTDLKEN GEICGGVGIL GIAWAFGGMI FILVYCTAGI SGGHINPAVT
      121 FGLFLARKVS LIRAIMYMVA QCLGAICGVG LVKAFQKSYF NKYGGGANSL AAGYSTGTGL
```

```
      181 GAEIIGTFVL VYTVFSATDP KRNARDSHVP VLAPLPIGFA VFMVHLATIP VTGTGINPAR
      241 SLGAAVIYNQ DKPWDDHWIF WVGPFIGAAI AAFYHQFILR AGAAKALGSF RSNPHN
//
LOCUS       GmPIP2-6       284 aa
DEFINITION  GmPIP2-6     284 aa
TITLE       GmPIP2-6
ORIGIN

        1 MAKDVEVAER GSFSGKDYQD PPPAPLIDAE ELTKWSFYRA LIAEFIATLL FLYITVLTVI
       61 GYKHQTDHAD ACGGVGILGI AWAFGGMIFI LVYCTAGISG GHINPAVTFG LFLARKVSLI
      121 RAIMYMVAQC LGAICGVGLV KAFQKSYFNK YGGGANSLAD GYSTGTGLGA EIIGTFVLVY
      181 TVFSATDPKR NARDSHVPVL APLPIGFAVF MVHLATIPVT GTGINPARSL GAAVIYNQDK
      241 PWDDHWIFWV GPFIGAAIAA FYHQFILRAG AAKALGSFRS NPHN
//
LOCUS       GmPIP2-7       286 aa
DEFINITION  GmPIP2-7     286 aa
TITLE       GmPIP2-7
ORIGIN

        1 MAKDVEVQEQ GGEYSAKDYH DPPPAPLFDP EELTQWSFYR ALIAEFIATL LFLYVTVLTI
       61 IGYKRQTDAT LGGTECDGVG ILGIAWAFGG MIFILVYCTA GISGGHINPA VTFGLFLGRK
      121 VSLIRALLYM VAQCAGAICG TGLAKGFQKS YYNRYGGGAN SVADGYNNGT ALGAEIIGTF
      181 VLVYTVFSAT DPKRNARDSH VPVLAPLPIG FAVFMVHLAT IPITGTGINP ARSFGAAVIY
      241 NKDKIWDDQW IFWVGPIVGA AVAAFYHQYI LRAAAIKALG SFRSNA
//
LOCUS       GmPIP2-8       286 aa
DEFINITION  GmPIP2-8     286 aa
TITLE       GmPIP2-8
ORIGIN

        1 MAKDVEVQEQ GGEYSAKDYH DPPPAPLFDP EELTQWSFYR ALIAEFIATL LFLYVTVLTI
       61 IGYKRQTDTT VGGTDCDGVG ILGIAWAFGG MIFILVYCTA GISGGHINPA VTFGLFLGRK
      121 VSLIRALLYM VAQCAGAICG TGLAKGFQKA YYNRYGGGAN SVADGYNNGT ALGAEIIGTF
      181 VLVYTVFSAT DPKRNARDSH VPVLAPLPIG FAVFMVHLAT IPITGTGINP ARSFGAAVIY
      241 NEDKIWDDQW IFWVGPIVGA AVAAFYHQYI LRAAAIKALG SFRSNA
//
LOCUS       GmPIP2-9       287 aa
DEFINITION  GmPIP2-9     287 aa
TITLE       GmPIP2-9
ORIGIN

        1 MAKDVEQVTE QGEYSAKDYH DPPPAPLIDP DELTKWSLYR AAIAEFIATL LFLYITVLTI
       61 IGYKRQSDTK IPGNTECDGV GILGIAWAFG GMIFILVYCT AGISGGHINP AVTFGLFLGR
      121 KVSLVRALLY MIAQCAGAIC GAGLAKGFQK SYYNRYGGGV NTVSDGYNKG TALGAEIIGT
      181 FVLVYTVFSA TDPKRSARDS HVPVLAPLPI GFAVFMVHLA TIPVTGTGIN PARSFGPAVI
      241 FNNDKAWDDQ WIYWVGPFVG AAVAAFYHQY ILRAAAIKAL GSFRSNT
//
LOCUS       GmPIP2-10       287 aa
DEFINITION  GmPIP2-10     287 aa
TITLE       GmPIP2-10
ORIGIN

        1 MAKDVEQVTE QGEYSAKDYH DPPPAPLIDP DELTKWSLYR AAIAEFIATL LFLYITVLTI
       61 IGYKRQSDTK IPGNTECDGV GILGIAWAFG GMIFILVYCT AGISGGHINP AVTFGLFLGR
```

```
      121 KVSLVRALLY MIAQCAGAIC GAGLAKGFQK SFYNRYGGGV NTVSDGYNKG TALGAEIIGT
      181 FVLVYTVFSA TDPKRNARDS HVPVLAPLPI GFAVFMVHLA TIPVTGTGIN PARSFGPAVI
      241 FNNDKAWDDQ WIYWVGPFVG AAVAAIYHQY ILRGSAIKAL GSFRSNA
//
LOCUS       GmPIP2-11       286 aa
DEFINITION  GmPIP2-11     286 aa
TITLE       GmPIP2-11
ORIGIN

        1 MAKDIETEVQ SGLPHKDYHD PPAAAFYDPA ELRKWSFYRA LIAEFVATLL FLYVTILTVI
       61 GYNHQTATGS PDLCNGVGVL GIAWAFGGMI FVLVYCTAGI SGGHINPAVT FGLFLARKVS
      121 LIRAVGYMVA QVLGAISGVG LVKALQKSYY NRYNGGVNML ADGYSKGTGL GAEIIGTFIL
      181 VYTVFSATDP KRVARDSHVP VLAPLPIGFA VFIVHLATIP ITGTGINPAR SLGPAVIFNN
      241 EKAWDDQWIF WVGPFIGAAI AAFYHQSVLR AQAAKALGSF RSSSNL
//
LOCUS       GmPIP2-12       285 aa
DEFINITION  GmPIP2-12     285 aa
TITLE       GmPIP2-12
ORIGIN

        1 MAKDLETEIQ SGLPHKDYHD PPPAPFYDPA ELRKWSFFRA LIAEFVATLL FLYVTILTVI
       61 GYNHQTATAA EPCSGVGVLG IAWAFGGMIF VLVYCTAGIS GGHINPAVTF GLFLARKVSL
      121 TRAVGYMVAQ VLGAISGVGL VKALQKSYYN RYKGGVNMLA DGYSKGTGLG AEIIGTFILV
      181 YTVFSATDPK RVARDSHVPV LAPLPIGFAV FMVHLATIPI TGTGINPARS LGPAVIFNNE
      241 KAWDDQWIFW VGPFIGAALA AFYHQSVLRA QAAKALGSFR SSSNL
//
LOCUS       GmPIP2-13       275 aa
DEFINITION  GmPIP2-13     275 aa
TITLE       GmPIP2-13
ORIGIN

        1 MSKEVSQQRK DYVDPPPAPL IDLAEIKLWS FYRALIAEFI ATLLFLYVTV ATVIGHKKQT
       61 GPCDGVGLLG IAWAFGGMIF VLVYCTAGIS GGHINPAVTF GLFLARKVSL IRALFYMVAQ
      121 CLGAICGVGL VKAFMKHSYN SLGGGANSVS AGYNKGSALG AEIIGTFVLV YTVFSATDPK
      181 RSARDSHIPV LAPLPIGFAV FMVHLATIPI TGTGINPARS FGAAVIYNNG KVWDDHWIFW
      241 VGPFVGALAA AAYHQYILRA AAIKALGSFR SNPTN
//
LOCUS       GmPIP2-14       278 aa
DEFINITION  GmPIP2-14     278 aa
TITLE       GmPIP2-14
ORIGIN

        1 MSKEVSQEGL QRKDYVDPPP APLFDLAEIK LWSFYRALIA EFIASLLFLY VTVATIIGHK
       61 KQTGPCDGVG LLGIAWSFGG MIFVLVYCTA GISGGHINPA VTFGLFLARK VSLIRAVFYM
      121 VAQCLGAICG VGLVKAFMKH SYNSLGGGAN SVSAGYNKGS ALGAEIIGTF VLVYTVFSAT
      181 DPKRSARDSH VPVLAPLPIG FAVFMVHLAT IPITGTGINP ARSLGAAVIY NNGKVWDEHW
      241 IFWVGPLVGA LAAAAYHQYI LRAGAIKALG SFRSNPTN
//
LOCUS       GmSIP1-1       247 aa
DEFINITION  GmSIP1-1     247 aa
TITLE       GmSIP1-1
ORIGIN

        1 MVSAIKAAIG DLVLTFLWVF FSSMLGLATN TITTALDLHH VSYNGFDYPS AVIITSLIFI
```

```
       61 LVTIFTFVGN ALGGASFNPT ANASSYAAGL GSDSLFSMAL RFPAQALGSV GGVLAVMEVM
      121 PPKYRHLIGG PSLKVSLHTG AIAEGVLTFV ITFVVLLIMI RGPRSEAVKT WLMAISTVVL
      181 ITAGSAYTGP AMNPAFAFGW AYFENWHNTW DQFYVYWICP FFGAILAAWL FRIVIPPAPR
      241 VVKQKKA
//
LOCUS       GmSIP1-2       245 aa
DEFINITION  GmSIP1-2     245 aa
TITLE       GmSIP1-2
ORIGIN

        1 MASAIKAAIG DLVLTFLWVF FSSMLGLVTN AITTALDLHH VSYNGFDYPS AVIITSLIFI
       61 LVTIFTFVGN ALGGASFNPT GNASSYAVGL GSDTLFSMAL RFPAQALGSV GGVLAVMEVM
      121 PPKYRHLIGG PSLKVSLHTG AIAEGVLTFV ITFVVLLIMI RGPRSEAVKT LLMAISTVVL
      181 ITAGSAYTGP AMNPAFAFGW AYFENWHNTW DQFYVYWICP FFGAILAAWL FRIVFPPRVV
      241 KQKKA
//
LOCUS       GmSIP1-3       248 aa
DEFINITION  GmSIP1-3     248 aa
TITLE       GmSIP1-3
ORIGIN

        1 MVGAIKAAIG DAVLTFMWVF CSSVLGIASG YITNALNLQH ITYNGFPYPS FLVTTTLVFV
       61 LVFLFTIIGN VLGGASFNPT GTASFYAVGL GSDTLFSMAL RFPAQAAGAA GGALAIMEVI
      121 PAKYRHMIGG PSLKVDLHTG AVAEGVLTFV ITFVVLLIFL KGPRSDLLKT WLLATATVVL
      181 VMVGSAYTGP AMNPANAFGW AYINNWHNTW DQFYVYWICP FAGAILAAWL FRAVFPPPSP
      241 PEVKQKKA
//
LOCUS       GmSIP1-4       248 aa
DEFINITION  GmSIP1-4     248 aa
TITLE       GmSIP1-4
ORIGIN

        1 MVGAIKAAIG DAVLTFMWVF CSSVLGIASG YITNALNLQH ITYNGFPYAS FLVTTTLVFV
       61 LVFLFTIIGN VLGGASFNPT GTASFYAVGL GSDTLFSMAL RFPAQAAGAA GGAMAIMEVI
      121 PAKYRHMIGG PSLKVDLHTG AVAEGVLTFV ITFAVLLIFL RGPRSDLLKT WLLATATVVL
      181 VMVGSAYTGP AMNPANAFGW AYLNNWHNTW DQFYVYWICP FTGAILAAWL FRAVFPPPPP
      241 PEVKQKKA
//
LOCUS       GmSIP1-5       239 aa
DEFINITION  GmSIP1-5     239 aa
TITLE       GmSIP1-5
ORIGIN

        1 MGLIKAAIGD GVLTSMWVFI ISTLRIVTTE VALFLGLQPL SLAGLIISTI LNSFYVLTIS
       61 FIGRILGGAN FNPSTSLSFY TAGLRPDSSL SSMAVRFPVQ AYGGAVGVKT LLLVMPSKYN
      121 DMLKGPFLKV DLHSGAVAEG VLTFTHNMAI FFVMFKGPRN PFVKVYLLSV TTAVLAILGG
      181 GFTGPSMNPA NAFGWAFVNN KHNTWEQFYV YWICPFIGAS SAALIFRSMF MPPIKQKKA
//
LOCUS       GmSIP1-6       239 aa
DEFINITION  GmSIP1-6     239 aa
TITLE       GmSIP1-6
ORIGIN

        1 MGWIKAAIGD AILTSMWVFI ISTLRIVTTE ITVFLGLQPF FLAGLIISTI LNSIYVLTIS
```

```
       61 FIGRILGGVS FNPSTSLSFY TAGLRPDSSL SSMAVRFPAQ AYGGAVGIKT LLLVMPSHYK
      121 DMLKGPFLKV DLHSGAVAEG LLTFIHNMAI FFVMFKGPRN PFVKVYLLSV TTAALAILGG
      181 GFTGPSMNPA NAFGWAFVNN KHNTWEQFYV YWIGPFIGAS SAALIFRSMF MPPIKQKKA
//
LOCUS       GmSIP2-1       230 aa
DEFINITION  GmSIP2-1     230 aa
TITLE       GmSIP2-1
ORIGIN

        1 MGRARLLVSD FVLSFMWVWS GVLLRILVFK HLGFAHGPLG EVIKTTFSVA NMFFFAFLVK
       61 VTRGAAYNPL TVLADAITGD FNTFLYCVGA RIPAQVVGSI VGVKLLIDTI PEVGVGPRLN
      121 VDIHQGSLTE GLLTFAIVTI SLGLATKIRE NFFMKTWISS LSKLTLHILG SDLTGGCMNP
      181 ASVMGWAYAR GDHITKEHFL VYWLAPIEAT IFAVWTSKFL VQPGKEHKKA
//
LOCUS       GmSIP2-2       236 aa
DEFINITION  GmSIP2-2     236 aa
TITLE       GmSIP2-2
ORIGIN

        1 MGRARLLVSD FVLSFMWVWS GVLLRIIVFN HLGFAHGPLG EVIKTTFSIA NMFFFAFLVK
       61 VTRGGAYNPL TVLADAISRD FNNFLYCAGA RIPTQVVGSI VGVKLLIDTI PEVGLGPRLN
      121 VDIHRGALTE GLLTFAIVTI SLGLASKIRE NFFMKTWISS LSKLTLHILG SDLTGGCMNP
      181 ASVMGWAYAR GDHITKEHFL VYWLAPIEAT ILAVWTFKFL VQPGKEDKST SKSKSD
//
LOCUS       GmTIP1-1       250 aa
DEFINITION  GmTIP1-1     250 aa
TITLE       GmTIP1-1
ORIGIN

        1 MAYRSAIVRR AQEASHRDTW RAALSEFIST LIFVFAGSGS SVAVNKLTVD KPSALVVAAV
       61 AHAFALFVAV SVSTNISGGH VNPAVTFGAF VGGNLTLLRC VLFWIAQILG SVIACLLLKF
      121 ITGGQDVPVF KLSSGVGVGN AVVLEMVMTF GLVYTVYATT VDPRSRRGSL GVMAPIVIGF
      181 IVGANVLVGG PFDGASMNPA ASFGPAVVGW SWKNHWVYWV GPLVGGGLAG FMYELIFVSH
      241 SRQRFRRSYY
//
LOCUS       GmTIP1-2       250 aa
DEFINITION  GmTIP1-2     250 aa
TITLE       GmTIP1-2
ORIGIN

        1 MPIRNIAVGR PEEATHPDTL KAALAEFIST FIFVFAGSGS GIAYNKLTDN GAATPAGLIS
       61 ASIAHAFALF VAVSVGANIS GGHVNPAVTF GAFVGGNITF LRGIVYVIAQ LLGSIVASLL
      121 LAFVTASTVP AFGLSAGVGV GNALVLEIVM TFGLVYTVYA TAIDPKKGNL GIIAPIAIGF
      181 IVGANILLGG AFSGAAMNPA VTFGPAVVSW TWTNHWIYWA GPLIGGGIAG LVYEVVFISH
      241 THEQLPTTDY
//
LOCUS       GmTIP1-3       250 aa
DEFINITION  GmTIP1-3     250 aa
TITLE       GmTIP1-3
ORIGIN

        1 MPIRNIAIGR PEEATHPDTL KAGLAEFIST LIFVFAGSGS GIAYNKLTDN GAATPAGLIS
       61 ASIAHAFALF VAVSVGANIS GGHVNPAVTF GAFVGGNITL LRGIVYVIAQ LLGSIVASLL
      121 LAFVTASPVP AFGLSAGVGV GNALVLEIVM TFGLVYTVYA TAVDPKKGNL GIIAPIAIGF
```

```
      181 IVGANILLGG AFSGAAMNPA VTFGPAVVSW TWTNHWIYWA GPLIGGGIAG LIYEVVFISH
      241 THEQLPSTDY
//
LOCUS       GmTIP1-4       245 aa
DEFINITION  GmTIP1-4     245 aa
TITLE       GmTIP1-4
ORIGIN

        1 MPISRIAIGN PSEFGQADAL KAALAEFISM LIFVFAGEGS GMAYNKLTDN GSATPAGVVA
       61 ASLSHAFALF VAVSVGANIS GGHVNPAVTF GAFIGGHISL LRGILFWIAQ LLGSVVACLL
      121 LKFATVGLSP GVGAANALVF EIVMTFGLVY TVYATAVDPK KGKLGIIAPI AIGFIVGANI
      181 LAGGTFSGAS MNPAVSFGPA VVSGTWANHW VYWAGPLIGS AIAAVVYETF FITPNSYEQL
      241 PVTDY
//
LOCUS       GmTIP1-5       252 aa
DEFINITION  GmTIP1-5     252 aa
TITLE       GmTIP1-5
ORIGIN

        1 MPISRIAIGN PSEFGQADAL KAALAEFISM LIFVFAGEGS GMAYNKLTDN GSATPAGVVA
       61 ASLSHAFALF VAVSVGANIS GGHVNPAVTF GAFIGGHISL LRGILYWIAQ LLGSVVACLL
      121 LKFATGGLET SAFSLSPGVG AANALVFEIV MTFGLVYTVY ATAVDPKKGN LGIIAPIAIG
      181 FIVGANILAG GAFDGASMNP AVSFGPAVVS GTWANHWVYW VGPLIGSAIA AIIYETFFIT
      241 PNSYEHLPVT DY
//
LOCUS       GmTIP1-6       252 aa
DEFINITION  GmTIP1-6     252 aa
TITLE       GmTIP1-6
ORIGIN

        1 MPISRIAIGN SSELNQSDAL KAALAEFISM LIFVFAGEGS GMAYNKLTNN GSATPAGLVA
       61 ASLSHAFALF VAVSVGANIS GGHVNPAVTF GAFVGGHITL FRSILYWIAQ LLGSVVACLL
      121 LKFATGGLET SAFALSPGVE AGNALVFEIV MTFGLVYTVY ATAVDPKKGD LGIIAPIAIG
      181 FIVGANILAG GAFDGASMNP AVSFGPAVVS WTWSNHWVYW VGPFAGAAIA AVVYEIFFIS
      241 PNTHEQLPVT DY
//
LOCUS       GmTIP1-7       252 aa
DEFINITION  GmTIP1-7     252 aa
TITLE       GmTIP1-7
ORIGIN

        1 MAVYRIAIGS PREASNPAAI RAAFAEFFSM LIFVFAGQGS GMAYSKLTGN GPATPGGLVV
       61 ASLSHTFGLF VAVAVGANIS GGHVNPAVTF GAFIGGNITL LRSILYWIAQ LLGSVVACIL
      121 LKVATGGMET SAFSLSSGVS VWNALVFEIV MTFGLVHTVY ATTVDPKKGN VGVIGPIAIG
      181 SIVGANILVG GAFDGASMNP AVCFGPALIN WSWTHHWVYW LGPFIGSATA AILYDNIFIG
      241 DDGHEPLSNS DF
//
LOCUS       GmTIP1-8       252 aa
DEFINITION  GmTIP1-8     252 aa
TITLE       GmTIP1-8
ORIGIN

        1 MAMHRIAIGT PGEAAQPDAI RAAFAEFFCM IIFVFAGEGS GMAYSKLTNN GPATPAGLIA
       61 ASLSHAFGLF VAVSVGANIS GGHVNPAVTF GAFIGGNITL LRSILYWIAQ LFGSVVACIL
```

```
      121 LKHATGGMET SGFSLSPGVS VWNALVFEIV MTFGLVYTVY ATAVDPKKGN AGVVAPIAIG
      181 FIVGANILVG GAFDGASMNP AVSFGPAVVT WSWTHHWVYW VGPFIGAAIA AIIYDNIFIG
      241 DDGHEPLSSS DF
//
LOCUS       GmTIP1-9       252 aa
DEFINITION  GmTIP1-9     252 aa
TITLE       GmTIP1-9
ORIGIN

        1 MAIYRIAIGT PGEAGQPDAI RAAFAEFFSM IIFVFAGEGS GMAYSKLTNN GPATPAGLIA
       61 ASLSHAFGLF VAVSVGANIS GGHVNPAVTF GAFIGGNITL LRSILYWIAQ LLGSVVACIL
      121 LKSATGGMET TGFSLSPGVS VWNALVFEIV MTFGLVYTVY ATAVDPKKGN VGVVAPIAIG
      181 FIVGANILVG GAFDGASMNP AVSFGPAVVT WSWTHHWVYW VGPFIGAAIA AVIYDNIFIG
      241 DDGHEPLSSS DF
//
LOCUS       GmTIP2-1       247 aa
DEFINITION  GmTIP2-1     247 aa
TITLE       GmTIP2-1
ORIGIN

        1 MAGIAFGSFN DSVSFASIKA YIAEFISTLL FVFAGVGSAI AYAKLTSDAA LDPTGLVAVA
       61 ICHGFALFVA VSVGANISGG HVNPAVTFGL ALGGHITILT GLFYWIAQLL GSIVASLLLK
      121 FVTGYDTPIH SVAAGIGAGE GVVTEIIITF GLVYTVYATA ADPKKGSLGT IAPIAIGFIV
      181 GANILAAGPF SGGSMNPARS FGPAVVSGDF HDNWIYWVGP LIGGGLAGLI YTYAFIPTNH
      241 APLATEF
//
LOCUS       GmTIP2-2       247 aa
DEFINITION  GmTIP2-2     247 aa
TITLE       GmTIP2-2
ORIGIN

        1 MAGIAFGSFN DSFSLASIKA YIAEFISTLL FVFAGVGSAI AYAKLTSDAA LDPTGLVAVA
       61 ICHGFALFVA VSVGANISGG HVNPAVTFGL ALGGHITILT GLFYWIAQLL GSIVASLLLK
      121 FVTGYDTPIH SVAAGVGAGE GVVTEIIITF GLVYTVYATA ADPKKGSLGT IAPIAIGFIV
      181 GANILAAGPF SGGSMNPARS FGPAVVSGDF HDNWIYWVGP LIGGGLAGLI YTYAFIPTQH
      241 APLATDF
//
LOCUS       GmTIP2-3       237 aa
DEFINITION  GmTIP2-3     237 aa
TITLE       GmTIP2-3
ORIGIN

        1 MAGIAFGNFN DSVSFASIKA YIAEFISTLL FVFAGVGSAI AYAKLTSDAA LDPTGLVAVA
       61 ICHGFALFVA VSVGANISGG HVNPAVTFGL ALGGHITILT GLFYWIAQLL GSIVASLLLK
      121 FVTGYDTPIH SVAAGIGAGE GVVTEIIITF GLVYTVYATT ADPKKGSLGT IAPIAIGFIV
      181 GANILAAGPF SGGSMNPARS FGPAVVSGDF HDNWIYWVGT LIGGGLAGLI YTYAFIM
//
LOCUS       GmTIP2-4       248 aa
DEFINITION  GmTIP2-4     248 aa
TITLE       GmTIP2-4
ORIGIN

        1 MGGIAFGRFD DSFSLTSIKA YIAEFHSTLL FVFAGVGSAI AYGKLTSDAA LDPAGLLAVA
       61 ICHGFALFVA VSVGANISGG HVNPAVTFGL ALGGHITILT GFFYWIAQLL GSIVACFLLN
```

```
      121 YVTGGLPTPI HSVASGVGAV EGVVTEIIIT FGLVYTVYAT AADPKKGSLG IIAPIAIGFI
      181 VGANILAAGP FSGGSMNPAR SFGPAVVSGD FHDNWIYWVG PLIGGGLAGL IYGNVFIRSD
      241 HAPLSSEF
//
LOCUS       GmTIP2-5       248 aa
DEFINITION  GmTIP2-5     248 aa
TITLE       GmTIP2-5
ORIGIN

        1 MGGIAFGRLD DSFSLTSIKA YIAEFHSTLL FVFAGVGSAI AYGKLTSDAA LDPAGLLAVA
       61 ICHGFALFVA VSVGANISGG HVNPAVTFGL ALGGHITILT GFFYWIAQLL GSIVACFLLN
      121 YVTGGLPTPI HSVASGVGAV EGVVTEIIIT FGLVYTVYAT AADPKKGSLG TIAPIAIGFI
      181 VGANILAAGP FSGGSMNPAR SFGPAVVSGD FHDNWIYWVG PLIGGGLAGL IYGNVFIRSD
      241 HAPLSSEF
//
LOCUS       GmTIP2-6       249 aa
DEFINITION  GmTIP2-6     249 aa
TITLE       GmTIP2-6
ORIGIN

        1 MVKITLGTFD DSFGVASLKA YLAEFHATLI FVFAGVGSAI AYNELTKDAA LDPTGLVAVA
       61 VAHAFALFVG VSVAANISGG HLNPAVTFGL AIGGNITLIT GFLYWIAQLL GSIVACLLLN
      121 FITAKSIPSH APATGVNDFQ AVVFEIVITF GLVYTVYATA ADPKKGSLGI IAPIAIGFVV
      181 GANILAAGPF SGGSMNPARS FGPAVVSGDF AANWIYWVGP LIGGGLAGLI YGDVFIGSYA
      241 AVPASETYP
//
LOCUS       GmTIP2-7       249 aa
DEFINITION  GmTIP2-7     249 aa
TITLE       GmTIP2-7
ORIGIN

        1 MVKIALGTLD DSFSAASLKA YFAEFHATLI FVFAGVGSAI AYNELTKDAA LDPTGLVAVA
       61 VAHAFALFVG VSVAANISGG HLNPAVTFGL AIGGNITLIT GFLYWIAQLL GSIVACLLLN
      121 LITAKSIPSH SPANGVNDLQ AVVFEIVITF GLVYTVYATA VDPKKGSLGI IAPIAIGFVV
      181 GANILAAGPF SGGSMNPARS FGPAVVSGDL AANWIYWVGP LIGGGLAGLI YGDVFIGSYA
      241 PVPASETYP
//
LOCUS       GmTIP3-1       254 aa
DEFINITION  GmTIP3-1     254 aa
TITLE       GmTIP3-1
ORIGIN

        1 MATRRYEFGR MNEASHPDSI RAALVEFLST CIFVFAGEGS ALALRQIYKE PGSSAGELVV
       61 IALAHAFALF AAISASMHVS GGHVNPAVTF GALLGGRISV LRALYYWVAQ LLGSIVAALL
      121 LRLVTNNMRP QGFSVSIGLG AFHGLILEIA LTFGLMYTVY ATAIDPKRGS IGSIAPLAIA
      181 FVVGANILAG GPFDGACMNP ARAFGPAMVG WRWHYHWIFW VGPLIGAALA ALLYEYVMVP
      241 IEPPHHQPLA GVDY
//
LOCUS       GmTIP3-2       254 aa
DEFINITION  GmTIP3-2     254 aa
TITLE       GmTIP3-2
ORIGIN

        1 MATRRYEFGR MNEASHPDSI RAALAEFLST CIFVFAGEGS ALALRQIYKE PGSSAGELVV
```

```
       61 IALAHAFALF AAISASMHVS GGHVNPAVTF GALLGGRISV LRAVYYWVAQ LLGSIVAALL
      121 LRLVTNNMRP QGFSVSIGLG AFHGLVLEIA LTFGLMYTVY ATAIDPKRGS IGSIAPLAIG
      181 FVVGANILAG GPFDGACMNP ARAFGPAMVG WRWHYHWIFW VGPFIGAALA ALLYEYVMVP
      241 NEPPHHQPLA AEDY
//
LOCUS       GmTIP3-3       255 aa
DEFINITION  GmTIP3-3     255 aa
TITLE       GmTIP3-3
ORIGIN

        1 MATRRYSFGR ADEATHPDSM RATLAEFAST FIFVFAGEGS SLALVKIYQD SAFSAGELLA
       61 VALAHAFALF AAVSSSMHVS GGHVNPAVTF GALIGGRISV LRAVYYWIAQ ILGAIVAALV
      121 LRLVTNNMRP SGFHVGQGVG VGHMLILEII MTFGLMYTVY GTAIDPKRGS VSNIAPLAIG
      181 LIVGANILVG GPFDGACMNP ALAFGPSLVG WRWHQHWIFW VGPLIGAALA ALVYEYVVIP
      241 TEPPHQHQPL APEDY
//
LOCUS       GmTIP3-4       255 aa
DEFINITION  GmTIP3-4     255 aa
TITLE       GmTIP3-4
ORIGIN

        1 MATRRYAFGR ADEATHPDSM RATLAEFVST FIFVFAGEGS GLALVKIYQD SAFSAGELLA
       61 VALAHGFALF AAVSASMHVS GGHVNPAVTF GALIGGRISV LRAVYYWIAQ ILGAIVAALV
      121 LRLVTNNMRP SGFHVGQGVG VGHMLILEIV MTFGLMYTVY GTAIDPKRGA VSNIAPLAIG
      181 LIVGANILVG GPFDGACMNP ALAFGPSLVG WRWHQHWIFW VGPLIGAALA ALVYEYVVIP
      241 TEPPHQHQPL APEDY
//
LOCUS       GmTIP4-1       246 aa
DEFINITION  GmTIP4-1     246 aa
TITLE       GmTIP4-1
ORIGIN

        1 MARIALGSTR EATQPDCIQA LIVEFIATFL FVFVGVASSM VVDKLGGDAL VGLFAVAVAH
       61 ALVVAVMISA AHISGGHLNP AVTLGLLAGG HITIFRSLLY WIDQLVAAAA ASYLLYYLSG
      121 GQATPVHTLA SGVGYGQGVV WEIVLTFSLL FTVYATMVDP KKGALAGLGP TLVGFVVGAN
      181 ILAGGAYSAA SMNPARSFGP ALVTGNWTDH WVYWVGPLIG GGLAGFIYET FFIDRSHVPL
      241 PRDEES
//
LOCUS       GmTIP4-2       246 aa
DEFINITION  GmTIP4-2     246 aa
TITLE       GmTIP4-2
ORIGIN

        1 MAKIALGSTR EATQPDCIQA LIVEFIATFL FVFVGVGSSM VVDKLGGDAL VGLFAVAVAH
       61 ALVVAVMISA AHISGGHLNP AVTLGLLAGG HITIFRSMLY WIDQLVAAAT ASYLLYYLSG
      121 GQATPVHTLA SGVGYGQGVV WEIVLTFSLL FTVYATMVDP KKGALAGLGP TLVGFVVGAN
      181 ILAGGAYSAA SMNPARSFGP ALVAGNWTDH WVYWVGPLIG GGLAGYIYET FFIDRSHVPL
      241 PRDEEN
//
LOCUS       GmTIP5-1       247 aa
DEFINITION  GmTIP5-1     247 aa
TITLE       GmTIP5-1
ORIGIN
```

```
        1 MAPSSVTVTS RFHESVTRNA LRSYLSEFIS TFFYVFLVIG AGMSSRKLMP DASLNPTSLV
       61 VVGIGSAFAL SSVLYIAWDI SGGHVNPAVT FAMAVGGHIS VPTALFYWVA QLIASVMACL
      121 VLRVIVVGMH VPTYTIAEEM TGFGASVLEG TLTFVLVYTV YAARDPRRGP MSSTGILVVG
      181 LIAGASVLAS GPFSGGSMNP ACAFGSAAIA GSFRNQAVYW VGPLIGATIA GLLYDNELGS
      241 KLCSVMQ
//
LOCUS       GmXIP1-1       312 aa
DEFINITION  GmXIP1-1     312 aa
TITLE       GmXIP1-1
ORIGIN

        1 MDDQFSTVHS HSREPMPRGL VHEKSSEPKF LAYIGAHEFF TIETWKAALV ELIATAALMF
       61 TLTSCNIACL ESQDVNPKLI LPFAVFIIVF LFLIVIVPLS GGHMNPIFTF IAALKGVVTL
      121 SRALLYVSAQ CIGSIIGFFV LKSVMEPKLA DTYSLGGCAL GDKGQSSGLR PQDALLLEFS
      181 CTFLVLFVGL TLAFDKKRCK ELGLPMVCLV VAASLALAVF VSITVTGRPG YAGAGLSPAR
      241 CLGPALLHGG PLWNGHWVFW LGPFLACIIY YSVSINLPKK GLNWVDGEYD VLRLALGSCR
      301 TISNNTDLND LS
//
LOCUS       GmXIP2-1       270 aa
DEFINITION  GmXIP2-1     270 aa
TITLE       GmXIP2-1
ORIGIN

        1 MWKAALTELT ATASLMFTLT TSIIACLDSH EIDPKLLVPF AVFTIAFLFL IVTVPLTGGH
       61 MSPVFTFIAA LKGVVTLTRA LIYVLAQCIG SIIGFFILKC VMDPKLAYTY SLGGCAISGQ
      121 GVINSSSGGI KPLDALLVEF TCTFVVLFVG VTLAFDKKRS RDLGLPMVCL VVAGAMALAV
      181 FVSITVTGRA GYAGVGLNPA RCLGPALLHG GLLWEGHWVF WLGPFLACGL VWVDGEYDVL
      241 KLALGSCGNV YNTSVSKDHQ LEEPSAGFQV
//
LOCUS       MaNIP1-1       271 aa
DEFINITION  MaNIP1-1     271 aa
TITLE       MaNIP1-1
ORIGIN

        1 MEEGAAGDGR EEGVNPDHGY ASSADKGCGL SLSIPFLQKI LAEIFGTYFL IFAGCASVTV
       61 NLSKGMITFP GICVVWGLAV MVMVYSVGHI SGAHFNPAVT IAFATCGRFP WKQVPAYVFA
      121 QLLGATLASG TLRLMFGGKH EHFPGTIPAG SDVQSLVLEF IISFYLMFVI SGVATDNRAI
      181 GELAGLAVGA TILLNVLIAG PISGASMNPA RTLGPAIVAN RWEGFWVYIV GPICGTVVGA
      241 WAYNLIRFTN KPLREITKSG SFLKSFRNNS T
//
LOCUS       MaNIP1-2       252 aa
DEFINITION  MaNIP1-2     252 aa
TITLE       MaNIP1-2
ORIGIN

        1 MTVRSKVFIV SWFNSNPDHC RDLGHLLYDI CWVWFGRSES KHRHRHIPRH MLGLGARGHG
       61 HGVLPRPRLR RPLQSRRHHR LRHMRKVPVE AGASLRFSSG FGIDHLDRDA EVAVRREAWP
      121 VPGNGSRRLR PPVPGSRVHH LQNLMFVISG VATDSRAIGE LAGLVVGATV VVNVLFAGPI
      181 SGASMNPARS LGPAIIANRW EGLWVYIVGP ICGTVLGAWA YNLIRFTDRP LLEITNTATA
      241 SFLKRLTRKD SA
//
LOCUS       MaNIP1-3       188 aa
DEFINITION  MaNIP1-3     188 aa
TITLE       MaNIP1-3
```

```
ORIGIN

        1 MVYSVGHISG AHFNPAVTTT FTILKQFPLK QLPLYMVAQL VGAILASGAV YLLFDPKAEH
       61 FYGTTPVGSA VQSFVLEIII SFLLMFVISG VATDTRAIGE LAGIAVGSTI LLNVLVAGPI
      121 SGASMNPARS IGPAIVMRNY KAIWAYVLGP MIGTLAGGFT YNLVRYTDKP LREITKSSSF
      181 LKSVSRNR
//
LOCUS       MaNIP2-1       293 aa
DEFINITION  MaNIP2-1     293 aa
TITLE       MaNIP2-1
ORIGIN

        1 MASSTRPNSS NEIHDIDVVT AQNSYISPTL LHQKSLKEVF PPFLARKVVA ETIATFLLVF
       61 ATCGSAALSK SNPGLVSQLG ASVAGGLIVT VMIYAVGHIS GAHMNPAVTL AFAVARHFPW
      121 IQVPFYMAAQ ISGAMIASFV LRELLHPITD LGTTAPSDTA VKALVMEIVV TFCMMFVTSA
      181 VATDTKAVGE LAGLAVGSSV CITSILAGPI SGGSMNPART LGPAVASSNY DSLWVYFLGP
      241 VLGTLSGACS YSFIRMTETQ PQATAAQKLS SFKLRRLQSL EMASPTNNAF DNI
//
LOCUS       MaNIP2-2       293 aa
DEFINITION  MaNIP2-2     293 aa
TITLE       MaNIP2-2
ORIGIN

        1 MASHGTRPTT TACNEIHDAV TTHTSISPSL LHRKSLEELF PPFLAEKVVA ETIATFLLVF
       61 VTCGSAALSK SEAGAVSQLG ASVAGGLIVT VMIYAVGHIS GAHMNPAVTL AFAVSRHFPW
      121 IQVPFYISAQ ISGAMVSSFV LRELLHPITD LGTTTPSDTA LKALVMEIVV TFCMMFVTSA
      181 VATDSKAVGE LAGLAVGSAV CITSILAGPI SGGSMNPART LGPALASRNY DALWVYLLGP
      241 VVGTLLGAFS YSFIRMTEKQ PLSTTTQKLS SFKLRRLQSQ DMPSPLADAS GRV
//
LOCUS       MaNIP2-3       301 aa
DEFINITION  MaNIP2-3     301 aa
TITLE       MaNIP2-3
ORIGIN

        1 MASSHVRPNN SNEIHDIDVV TAQTLTTPSF FDPPRVHRRR NLKELFPPFL PRKVVSEMIA
       61 TFLLVFVTCG AGALNKNNPG VVSQLGQSVA GGLIVTVMIY AVGHISGAHM NPAVTLAFAV
      121 ARHFPWIQVP FYMLAQIAGS TTASYILREL LDPIHDLGTT TPSHTAAKAL VAEIVVTFNM
      181 MFVTAAVATD TKAVGELAGL AVGSAVCITS ILAGPISGGS MNPARTLGPA LASNKFDSLW
      241 VYFVGPPVGT VAGALAYSFI RLDEHSLSSQ KDSQKSPSLK MRRVQSQDMA SPTNDAFESG
      301 V
//
LOCUS       MaNIP2-4       323 aa
DEFINITION  MaNIP2-4     323 aa
TITLE       MaNIP2-4
ORIGIN

        1 MKCLKRSEIF SQPGRTQEDH TLRRSSSWRL EMASQTRPNI SNEIHDIDVV TAQSSVSPRL
       61 LHHESLRELF PPFLARKVVA EMISTFLLVF VTCGAGALNK SNSGVVSQLG ASVAGGLIVT
      121 VMIYAVGHIS GAHMNPAVTF AFAVSRHFPW IQVPFYMCAQ ISGAMVASFV LRELLHPITN
      181 LGTTTPSDTA AKALVMETVV TFCMMFVTSA VATDTKAVGE LAGLAVGSSV CITSILAGPV
      241 SGGSMNPART LGPAVASRNY QSLWVYFVGP VLGTVSGSFS YSFIRMTEKQ QHTTAAQKLS
      301 SFKLRRLQSQ EMASPTSNAF ENV
//
LOCUS       MaNIP3-1       359 aa
```

```
DEFINITION  MaNIP3-1     359 aa
TITLE       MaNIP3-1
ORIGIN

        1 MHQYLIQQIL TKNTLKPKPL AMPEPETPNV SAPATPGTPG APLFNSLRVD SLSYDRKSMP
       61 RCNRCLPLES WASSPHTCFI ELPKPDVSLT RKLGAEFVGT FILIFGATAA PIVNQKYNGA
      121 ETLIGNAACA GLAVMIVILS TGHISGAHLN PSLTIAFAML RHFPWAHVPA YILAQVSASI
      181 CASFALKAVF HPFLSGGVTV PSVSSPQAFF IEFLITFNLL FVVTAVATDT RAVGELAGIA
      241 VGATVMLNIL VAGPSSGGSM NPVRTLGPAV AAGNYERIWI YLVAPTAGAV TGAAVYTAVK
      301 LKEEDGEMPR QQLLEDYPRH TRYALQNDSW IVASYFYLCS VKHIFFLLQM LNCEYCKLR
//
LOCUS       MaNIP3-2       223 aa
DEFINITION  MaNIP3-2     223 aa
TITLE       MaNIP3-2
ORIGIN

        1 IGAEFVGTFI LIFGATAAPI VNQKYNGAEA LIGNAACAGL AVMIVILSTG HISGAHLNPS
       61 LTIAFAALRH FPWSHVPAYV VAQVSASICA SFALKGVFHP FLSGGVTVPS VSTAQAFFIE
      121 FVITFNLLFV VTAVATDTRA VGELAGIAVG ATVMLNILIA GPSSGGSMNP VRTLGPAVAA
      181 GNYKQIWIYL VAPTAGAVAG AAVYTAVKLK GDDGELPRRS FRR
//
LOCUS       MaPIP1-1       214 aa
DEFINITION  MaPIP1-1     214 aa
TITLE       MaPIP1-1
ORIGIN

        1 MGVVKSDTKC STVGIQGIAW AFGGMIFALV YCTAGISGGH INPAVTFGLF LARKLSLTRA
       61 LFYMVMQCLG AICGAGVVKG FRKGLYESNG GGANVVAAGY TKGDGLGAEI VGTFILVYTV
      121 FSATDAKRNA RDSHVPILAP LPIGFAVFLV HLATIPITGT GINPARSLGA AVIYNKDHAW
      181 DDHWIFWVGP FIGAALAAMY HQVVIRAIPF KSRP
//
LOCUS       MaPIP1-2       190 aa
DEFINITION  MaPIP1-2     190 aa
TITLE       MaPIP1-2
ORIGIN

        1 MIFALVYCTA GISGGHINPA VTFGLFLARK LSLTRAIFYM VMQCLGAICG AGVVKGFQKG
       61 VYQSNGGGAN VVASGYSKGD GLGAEIVGTF ILVYTVFSAT DAKRNARDSH VPILAPLPIG
      121 FAVFLVHLAT IPITGTGINP ARSLGAAVIY NKDHAWDDHW IFWVGPFIGA ALAALYHQVV
      181 IRAIPFKSRS
//
LOCUS       MaPIP1-3       190 aa
DEFINITION  MaPIP1-3     190 aa
TITLE       MaPIP1-3
ORIGIN

        1 MIFALVYCTA GISGGHINPA VTFGLFLARK LSLTRAVFYM VMQCLGAVCG AGVVKGFQKG
       61 VYESNGGGAN VVASGYSKGD GLGAEIVGTF ILVYTVFSAT DAKRNARDSH VPLLAPLPIG
      121 FAVFLVHLAT IPITGTGINP ARSLGAAIIY NKDHAWDDHW IFWVGPFIGA ALAAIYHQVV
      181 IRAIPFKNRS
//
LOCUS       MaPIP1-4       190 aa
DEFINITION  MaPIP1-4     190 aa
TITLE       MaPIP1-4
```

```
ORIGIN

        1 MIFALVYCTA GISGGHINPA VTFGLLLARK LSLTRAIFYV VMQCLGAICG AGVVKGFQKG
       61 VYESNGGGAN VVAAGYTKGD GLGAEIVGTF ILVYTVFSAT DAKRSARDSH VPVLAPLPIG
      121 FAVFLVHLAT IPITGTGINP ARSLGAAIIY NKEHAWNDHW IFWVGPLIGA ALAAIYHQVV
      181 IRAIPFRSKR
//
LOCUS       MaPIP1-5       214 aa
DEFINITION  MaPIP1-5     214 aa
TITLE       MaPIP1-5
ORIGIN

        1 MGVVKSSTKC STVGIQGIAW AFGGMIFALV YCTAGISGGH INPAVTFGLF LARKLSLTRA
       61 LFYMVMQCLG AICGAGVVKG FQKGLYENNG GGANVVAPGY TKGDGLGAEI VGTFILVYTV
      121 FSATDAKRSA RDSHVPILAP LPIGFAVFLV HLATIPITGT GINPARSLGA AIIYNKGHAW
      181 DDHWIFWVGP FIGAALAALY HQVVIRAIPF KSRS
//
LOCUS       MaPIP1-6       190 aa
DEFINITION  MaPIP1-6     190 aa
TITLE       MaPIP1-6
ORIGIN

        1 MIFALVYCTA GISGGHINPA VTFGLLLARK LSLNRALFYM VMQCLGAICG AGVVKGFQKG
       61 LYQSNGGGAN VVAAGYTKGD GLGAEIVGTF ILVYTVFSAT DAKRNARDSH VPILAPLPIG
      121 FAVFLVHLAT IPITGTGINP ARSFGAAVIY NKDHAWDDHW IFWVGPFIGA ALAALYHQVV
      181 IRAIPFKNRT
//
LOCUS       MaPIP1-7       190 aa
DEFINITION  MaPIP1-7     190 aa
TITLE       MaPIP1-7
ORIGIN

        1 MIFALVYCTA GISGGHINPA VTFGLFLARK LSLTRAIFYM VMQCLGAICG AGVVKGFQKG
       61 VYENNGGGAN VVAAGYSKGD GLGAEIVGTF ILVYTVFSAT DAKRNARDSH VPILAPLPIG
      121 FAVFLVHLAT IPITGTGINP ARSLGAAIIY NKDHAWDDHW IFWVGPFIGA ALAAFYHQIV
      181 IRAIPFKSRS
//
LOCUS       MaPIP1-8       190 aa
DEFINITION  MaPIP1-8     190 aa
TITLE       MaPIP1-8
ORIGIN

        1 MIFALVYCTA GISGGHINPA VTFGLFLARK LSLTRALFYM VMQCLGAICG AGVVKGYQKG
       61 LYESNGGGAN VVAPGYTKGD GLGAEIVGTF ILVYTVFSAT DAKRNARDSH VPILAPLPIG
      121 FAVFLVHLAT IPITGTGINP ARSLGAAIIY NKKHAWDDHW IFWVGPFIGA ALAAIYHQIV
      181 IRAIPFKSRP
//
LOCUS       MaPIP1-9       190 aa
DEFINITION  MaPIP1-9     190 aa
TITLE       MaPIP1-9
ORIGIN

        1 MIFILVYCTA GISGGHINPA VTFGLLLARK LSLTRALFYM VMQCLGAICG AGVVKGFKKG
       61 LYENNGGGAN VVAPGYTKGG GLGAEIVGTF ILVYTVFSAT DAKRSARDSH VPVLAPLPIG
```

```
      121 FAVFLVHLAT IPITGTGINP ARSLGAAIVY DKSHAWNDHW IFWVGPFIGA ALAAMYQQIV
      181 IRAIPFKSRP
//
LOCUS       MaPIP2-1       196 aa
DEFINITION  MaPIP2-1     196 aa
TITLE       MaPIP2-1
ORIGIN

        1 MIFILVYCTA GISGGHINPA VTFGLFLARK VSLVRAFLYI VAQCLGAICG VGLVKGFQKA
       61 YFVRYGGGAN ELSDGYSKGT GLGAEIIGTF VLVYTVFAAT DPKRSARDSH VPVLAPLPIG
      121 FAVFMVHLAT IPITGTGINP ARSFGAAVIY NKDKAWDDQW IFWVGPAIGA AIAAAYHQYV
      181 LRASGVKALG SFRSSA
//
LOCUS       MaPIP2-2       198 aa
DEFINITION  MaPIP2-2     198 aa
TITLE       MaPIP2-2
ORIGIN

        1 MIFVLVYCTA DISGGHINPA VTLGLFVARK VSLLRAVLYM VAQCLGAICG VRIVKAIMKQ
       61 QFNAFGGGVN VVAPGHSKGT ALGTEIVGTF VLVYTVFSAT DPKRSARDSH VPVLAPLSIG
      121 FAVFMVHLAT IPITGTGINP ARSLGAAVIF NQHKPWHDHW IFWVGPFGGA LAAAVYYQYV
      181 LRASTIKDLV SFRSSRSN
//
LOCUS       MaPIP2-3       196 aa
DEFINITION  MaPIP2-3     196 aa
TITLE       MaPIP2-3
ORIGIN

        1 MIFILVYCTA GISGGHINPA VTFGLFLARK VSLIRALLYM IGQCLGAICG VGLVKGFQKA
       61 FFVRYGGGAN ELSDGYSKGT GLAAEIIGTF VLVYTVFSAT DPKRSARDSH VPVLAPLPIG
      121 FAVFMVHLAT IPITGTGINP ARSFGAAVIY NKDKAWDDQW IFWVGPLIGA AIAAAYHQYI
      181 LRAGAVKALG SFRSNA
//
LOCUS       MaPIP2-4       196 aa
DEFINITION  MaPIP2-4     196 aa
TITLE       MaPIP2-4
ORIGIN

        1 MIFILVYCTA GISGGHINPA VTLGLFLARK VSLVRALLYM VAQCLGAICG VGLVKGFQEA
       61 YFVRYGGGAN ELSAGYSKGT GLAAEIIGTF VLVYTVFAAT DPKRNARDSH VPVLAPLPIG
      121 FAVFMVHLAT IPITGTGINP ARSLGAAVIY NQDKAWDDQW IFWVGPFVGA AIAAAYHQYV
      181 LRASGAKAMG SFGSNA
//
LOCUS       MaPIP2-5       195 aa
DEFINITION  MaPIP2-5     195 aa
TITLE       MaPIP2-5
ORIGIN

        1 MIFILVYCTA GISGGHINPA VTFGLFLARK ISLVRALLYM IAQCLGAICG VGLVKGFQSA
       61 YYVRYGGGAN ELSDGYSKGT GLAAEIIGTF VLVYTVFSAT DPKRNARDSH VPVLAPLPIG
      121 FAVFMVHLAT IPITGTGINP ARSFGAAVIY NKDKAWDDQW MFWVGPFIGA AVAAAYHQYI
      181 LRASGAKALG SSSSI
//
LOCUS       MaPIP2-6       282 aa
```

```
DEFINITION  MaPIP2-6     282 aa
TITLE       MaPIP2-6
ORIGIN

        1 MSKEVSVEVE QPPAKDYSDP PPAPLLDFGE VRLWSFYRAL IAEFVATLLF LYVSIATVIG
       61 HKEQNAADQC SGVGLLGIAW AFGGMIFILV YCTAGISGGH INPAVTFGLF LARKVSLIRA
      121 VLYIVAQCLG GIVGVGIVKG IMKHQYNSLG GGANVVATGY SKGTALGAEI IGTFVLVYTV
      181 FSATDPKRSA RDSHVPVLAP LPIGFAVFMV HLATIPITGT GINPARSLGA AVIYNQDKAW
      241 DDHWIFWVGP FIGALAAAAY HQYILRAAAI KALGSFRSNP SN
//
LOCUS       MaPIP2-7       175 aa
DEFINITION  MaPIP2-7     175 aa
TITLE       MaPIP2-7
ORIGIN

        1 MRLLPGEHIN PAVSFGLLLG RKISLLRAVL DMVLQCMGAI CGVGIVKGIM NHPYNSLGGR
       61 ANQVAAGCSQ GTALGAEIIG TFVLVYTVFS ATDPKRSARD SHVPVMAPLP IGFAVFTVHL
      121 GTGINPARSL GAAVLYDQRK AWLAVVQWIF WVGPFAGALA AAVKASSSFR SSNGK
//
LOCUS       MaPIP2-8       281 aa
DEFINITION  MaPIP2-8     281 aa
TITLE       MaPIP2-8
ORIGIN

        1 MTDEVRVVTE HPPAPLFDVG ELKLWSFYRA LIAEFVATLL FLYVLVATVI GHKAASLDNQ
       61 CGGVGLLGIA WAVGGMIFLL VYCTAGISGG HINPAVTFGL LLGRKVSVLR AVSYMVAQCA
      121 GAICGVGIAR AIMKHQFDAF GGGTNVVALC YSNGAALGAE IIGTFVLVYT VFTATDPTRN
      181 ARDSHVPVLA PLSIGFAVFL VHLATIPITG TGINPARSLG AAVIYNRRKA WNDQWIFWVG
      241 PFIGAAAAAL YHEYVLKSAA IKAFRRSTAT PEASAPRPPP P
//
LOCUS       MaPIP2-9       281 aa
DEFINITION  MaPIP2-9     281 aa
TITLE       MaPIP2-9
ORIGIN

        1 MSKEVSEAEQ APAKDYRDPP PAPLLDFGEL RLWSFYRALI AEFVATLLFL YVTIATVIGH
       61 KEQNAADQCS GVGILGIAWA FGGMIFILVY CTAGISGGHI NPAVTFGLFL ARKVSLIRAL
      121 LYIVAQCLGA IVGVGIVKGI MKHQYNSLGG GANEVASGYS KGTALGAEII GTFVLVYTVF
      181 SATDPKRSAR DSHVPVLAPL PIGFAVFMVH LATIPITGTG INPARSLGAA VIYNQDKPWD
      241 DHWIFWVGPF VGALAAAAYH QYILRAAAIK ALGSFRSNPT N
//
LOCUS       MaPIP2-10       198 aa
DEFINITION  MaPIP2-10     198 aa
TITLE       MaPIP2-10
ORIGIN

        1 MIFILVYCTA GISGGHINPA VTLGLFLARK VSLIRALLYM VAQCLGAIVG VGIVKGIMKH
       61 QYNSLGGGAN MVAAGYSKGT ALGAEIIGTF VLVYTVFSAT DPKRSARDSH VPVLAPLPIG
      121 FAVFMVHLAT IPITGTGINP ARSLGAAVIY NQDKPWDDHW IFWVGPFVGA LAAAAYHQYI
      181 LRAAAIKALG SFRSNPTN
//
LOCUS       MaPIP2-11       199 aa
DEFINITION  MaPIP2-11     199 aa
TITLE       MaPIP2-11
```

```
ORIGIN

        1 MIFVLVYCTA GISGGHINPA VTFGLFLARK VSLLRAVFYM VAQCMGAICG VGIVKGIMKH
       61 QFNRFGGGAN VVAPGYSKGT ALGAEIIGTF LLVYTVFAAT DPKRRARDSH VPVLAPLPIG
      121 FAVFMVHLAT IPITGTGINP ARSFGPAVIY NRHKPWHDHW IFWVGPFVGA LAAEVYHQHV
      181 LRAANVKTLG SFRSSRSNC
//
LOCUS       MaPIP2-12       198 aa
DEFINITION  MaPIP2-12     198 aa
TITLE       MaPIP2-12
ORIGIN

        1 MIFILVYCTA GISGGHINPA VTLGLFLARK VSLLRAVMYM VAQCLGAICG VGIVKGIMKH
       61 QFNAFGGGAN SVAAGYSKGT AFGAESIGTF VLVYTVLSAT DPKRSARDSH VPVLAPLPIG
      121 FAVFMVHLAT IPITGTGINP ARSFGAAVIY NQHKPWHDHW IFWVGPFVGA LAAAVYHQYV
      181 LRAAALKALG SFRSSRSN
//
LOCUS       MaSIP1-1       238 aa
DEFINITION  MaSIP1-1     238 aa
TITLE       MaSIP1-1
ORIGIN

        1 MGAIRAAAAD GLITFLWVFC VSTVRAATSL VTAALQIQGV AFSLFVTTTL IFALVFVFGL
       61 ITAAIGGASF NPTATAAFYA AGLGSDNLLS MALRFPAQAA GAVAGALAIM EVMPPQHKRM
      121 LGGPSLKVDL HTGALAEGVL TFIITLAVLW IIIRGPRSPV VKTWMVAVST VAMVVAGAGY
      181 TGPAMNPANA FGWAYINNRH NTWEQFYVYW ICPFIGAIVA GWFFRIIFPQ RAEKAKKA
//
LOCUS       MaSIP2-1       261 aa
DEFINITION  MaSIP2-1     261 aa
TITLE       MaSIP2-1
ORIGIN

        1 MLDQKEFVNK KETDVEEEKE HSNQASRVKL IVSDSFLSFM WVLSGSVIRY LIYMILGTGM
       61 DPISVLLKGY LALVYLYYFS QLRKVTNGGT YNPLFVLCHA ISDNFVEFLY AVFGRIPAQV
      121 LGSVIGVWLI NATFPAAANG PRLNVDVSYG ALIEGLITFA IIIVSLGLNK FPRSSHKTWI
      181 SSFAKLALHV LASDITGGVM NPASAFGWAY AQGKHLTKEH LCVYWLAPLE ATLLVVWICS
      241 LFIKLPKRKR QHEMQYKDKL V
//
LOCUS       MaSIP2-2       238 aa
DEFINITION  MaSIP2-2     238 aa
TITLE       MaSIP2-2
ORIGIN

        1 MGRLGLVMCD AAMSFMWVWA GALVKLLVYD ALGLGHRPGG EALKMALVVG YMFLFAWLGH
       61 VTRGGAYNPL TVLSYAFSGG PEGFLFTALG RIPAQVIGSA TGVRFIKQTF PSIGHGPRLS
      121 IDIHRGAWTE GFLTFMIVMA SLMLKKKDPG SFFMKTWISS IFKLALNVLG SDLTGGIMNP
      181 ASAFAWAYAR GDHITLDQLI VYWFAPIQAT LLAVWTFGLL TEPKSKVQKA EENKVKSE
//
LOCUS       MaTIP1-1       250 aa
DEFINITION  MaTIP1-1     250 aa
TITLE       MaTIP1-1
ORIGIN

        1 MPITQIAIGT TAEATHPTAL KAALAEFICT FIFVFAGQGS GMAYNKLTSD GAATPEGLIA
```

```
       61 AALAHGFALF VAVSVGANIS GGHVNPAVTF GAFVGGNITL LRGILYWIAQ LLGSTVACLL
      121 LRFSTGGLET GTFGLTGVSV WEALVLEIVM TFGLVYTVYA TAVDPKKGSL GTIAPIAIGF
      181 IVGANILVGG PFDGASMNPA VSFGPALVSW SWTHQWVYWL GPLIGGALAG IVYEIFFISH
      241 SHEQLPTADY
//
LOCUS       MaTIP1-2       250 aa
DEFINITION  MaTIP1-2     250 aa
TITLE       MaTIP1-2
ORIGIN

        1 MPIPRIAVGT QEEATHPGTL KAALAEFIST LIFVFAGQGS GMAFSKLTGG AATTPAGLIA
       61 AALAHAFALF VAVSVGANIS GGHVNPAVTF GVFIGGNITL LRSIIYWIAQ LLGSTVACLL
      121 LRYSTGGLST GSFALSGVSV WEALVLEIVM TFGLVYTVYA TAVDPKKGSL GTIAPIAIGF
      181 IVGANILAGG AFDGASMNPA VSFGPALVSW SWDDHWVYWA GPLIGGGLAG LVYEFFFISH
      241 THEQLSSADY
//
LOCUS       MaTIP1-3       251 aa
DEFINITION  MaTIP1-3     251 aa
TITLE       MaTIP1-3
ORIGIN

        1 MPISRIAIGT TEEATHPSAL KAALAEFICT FIFVFAGQGS GMAYSKMTSG GAATPTGLIM
       61 AALAHAFALF VAVSVGANIS GGHVNPAVTF GAFVGGNITL LRGVLYWVAQ LLGSTAACLL
      121 LHFATGGLET GTFGLSSGVG VWEALVLEAV MTFGLVYTVY ATAVDPRRGS LGAIAPIAIG
      181 FIVGANILVG GAFDGASMNP AVSFGPALVS WSWTHQWVYW LGPLSGGALA GLVYEIFFIC
      241 STHEQLASAD Y
//
LOCUS       MaTIP1-4       250 aa
DEFINITION  MaTIP1-4     250 aa
TITLE       MaTIP1-4
ORIGIN

        1 MPFSQIAIGR PEEATHPSAL KAALAEFICT LIFVFAGQGS GMAYNKLTSD GAATPAGLIA
       61 AALAHGFALF VAVSVGANIS GGHVNPAVTF GAFVGGNITL LRGILYWIAQ LLGSTVACLL
      121 LRFSTGGLET GTFGLSGVSA WEALVLEIVM TFGLVYTVYA TAVDPKKGSL GTIAPIAIGF
      181 IVGANILVGG PFSGASMNPA VSFGPALVSW SWTHQWIYWL GPLIGGGLAG IVYEFFFISH
      241 SHEQLPTTDY
//
LOCUS       MaTIP1-5       252 aa
DEFINITION  MaTIP1-5     252 aa
TITLE       MaTIP1-5
ORIGIN

        1 MPIGSIAIGA PGEASHPDTI KASLAEFIST LIFVFAGEGS GMAFNKLTND GSTTPAGLVA
       61 ASLAHGFALF VAVSVGANIS GGHVNPAVTF GAFLGGNISL IRGILYWIAQ LLGSVVACLL
      121 LKLATGGLET SAFSLSSDVS VWNAVVFEIV MTFGLVYTVY ATAVDPRKGD LGVIAPIAIG
      181 FIVGANILAG GAFDGASMNP AVSFGPAVVS WTWDNHWVYW VGPLIGAAIA ALVYDGVFIG
      241 QATHEQLPPS DY
//
LOCUS       MaTIP1-6       252 aa
DEFINITION  MaTIP1-6     252 aa
TITLE       MaTIP1-6
ORIGIN
```

```
        1 MPILRITIGT PEEARHPTAL KAALAEFISV LIFVFAGQGS GMAFNKLTDD GSTTPAGLVS
       61 ASLAHGFGLY VAVAVGANIS GGHVNPAVTF GAFLGGNITL LRGILYWIAQ LLGSVVACLL
      121 LKFATGGLET TPFSLSSSVT VWNALVFEIV MTFGLVYTVY ATAIDPKKGN LGIIAPLAIG
      181 LVVGANILAG GAFDGASMNP AVSFGPAVVS WTWDNHWVYW VGPLLGGGIA ALVYDGVFIG
      241 FGTHEQLPTT DY
//
LOCUS       MaTIP2-1       249 aa
DEFINITION  MaTIP2-1     249 aa
TITLE       MaTIP2-1
ORIGIN

        1 MVKLTLGSLG DSFSAGSLKA YLAEFIATLL FVFAGVGSAI AYGKLTSGAA LDPAGLVAVA
       61 LAHGLALFVG VSMAANISGG HLNPAVTFGL AVGGHITLLT GVFYWIAQLL GSTVACLLLK
      121 FVTGGMAVPT HGVAAGMSEL EGVVMEVVIT FALVYTVYAT AADPKKGPLG TVAPIAIGFI
      181 VGANILAAGP FSGGSMNPAR SFGPAVASGD FSGNWVYWVG PLIGGGLAGL IYGDIFIGSY
      241 EAVAAQDYP
//
LOCUS       MaTIP2-2       249 aa
DEFINITION  MaTIP2-2     249 aa
TITLE       MaTIP2-2
ORIGIN

        1 MVKLALGSLG DSFSVVSLKS YLAEFIATLL FVFAGVGSAI AYGKLTGGAA LDPAGLVAVA
       61 LAHGLALFVG VSMAANISGG HLNPAVTFGL AVGGHITILT GIFYWVAQLL GSTVACLLLK
      121 FVTGGLAVPT HGVAAGMSEL EGVVMEVVIT FALVYTVYAT AADPKKGSLG TVAPIAIGFI
      181 VGANILAAGP FSGGSMNPAR SFGPAVASGD FAGNWVYWVG PLIGGGLAGL IYGDIFIGSY
      241 QPVAAQDYP
//
LOCUS       MaTIP2-3       152 aa
DEFINITION  MaTIP2-3     152 aa
TITLE       MaTIP2-3
ORIGIN

        1 MLNLTSMVAM ALSHGLSLFI GVSMMVNISG GHLNLLLDST IACLLLKFIT EGMAFPMHGV
       61 VAGMSEQEGM VMEVVITLAL VYTVYATAAD PKKGPLGTAD MVNSDHLPLH ASSAGLTPNN
      121 TYKTAIHTIG GKGTPEEHPE LPFLNFACIR SI
//
LOCUS       MaTIP2-4       243 aa
DEFINITION  MaTIP2-4     243 aa
TITLE       MaTIP2-4
ORIGIN

        1 MACIAFGRCD DSFSATSLKA YLAEFISTLL FVFAGVGSAI AYGKLTSGAA LDAAGLVAVA
       61 LCHGLALFVA VAIAANISGG HVNPAVTFGL ALGGQITILT GLLYWVAQLL GAVVGAFLLK
      121 FATGLDTPTH SLGVGAVEGV VMEIIITFAL VYTVYATAVD PKRGSLGTVA PIAIGLIVGA
      181 NILAAGPFSG GSMNPARSFG PAVASGDFAD LWVYWVGPLI GGGLAGLVYT YAYMCTDHTP
      241 LPQ
//
LOCUS       MaTIP2-5       245 aa
DEFINITION  MaTIP2-5     245 aa
TITLE       MaTIP2-5
ORIGIN

        1 MAGIAFGRFD DSFSVGSLKA YLAEFISTLL FVFAGVGSAI AYNKLTSSAA LDPAGLVAIA
```

```
       61 VCHGFALFVA VSVGFNISGG HVNPAVTFGL ALGGQITILT GILYWISQLL GAVVGAFLLK
      121 FSTGLDTPTH GLGAGVGAGE GVVMEIIITF ALVYTVYATA ADPKKGSLGT IAPIAIGFIV
      181 GANILAAGPF SGGSMNPARS FGPAVASGDF SDLWIYFVGP LIGGGLAGLV YTYAYLLHDH
      241 QPLPQ
//
LOCUS       MaTIP2-6       245 aa
DEFINITION  MaTIP2-6     245 aa
TITLE       MaTIP2-6
ORIGIN

        1 MAGIAFGQFD DSFSVGTLKA YLAEFISTLL FVFAGVGSAI AYNKLTSSAA LDPAGLVAIA
       61 VCHGLALFVA VSVGANISGG HVNPAVTFGL ALGGQITILT GIFYWVAQLL GAVVGAFLVK
      121 FATGLDTPTH GLGDGVGAGE AVVMEIIITF ALVYTVYATA ADPKKGSLGT IAPIAIGFIV
      181 GANILAAGPF SGGSMNPARS FGPAVASGNF SDLWIYWVGP LIGGGIAGLV YTYAYMCSDH
      241 QPLPQ
//
LOCUS       MaTIP3-1       254 aa
DEFINITION  MaTIP3-1     254 aa
TITLE       MaTIP3-1
ORIGIN

        1 MPPRRFAFGR AEDAVHPDTM RAALSEFIAT ALFVFAAEGS VLSLGKLYKD TSTAGGLVVV
       61 AIAHALALSV AVSVSLNISG GHVNPAVTLG ALVGGRISLI LAVFYWVAQL LGAVVAALLL
      121 RLATGGMRPL GFGVASGVSE GHAVLLEIVM TFGLVYTVYA TAIDPRRGHL GIIAPLAIGF
      181 ILGANILAGG PFDGAAMNPA RAFGPAVVGW RWKSHWVYWV GPLVGAALAG LIYEFLVIPD
      241 ETPRTHQPLA PEDY
//
LOCUS       MaTIP3-2       254 aa
DEFINITION  MaTIP3-2     254 aa
TITLE       MaTIP3-2
ORIGIN

        1 MPPRRFAFGR TDDAVHPDTM RAALSEFIAT ALFVFAAEGS ILSLGKLYKD TSTAGGLVVV
       61 AIAHALALAV AVAIAFNISG GHVNPAVTLG ALVGGRISLV RAVFYWVAQL LGAVVAALLL
      121 RLATGGMRPV GFSVASGVSD WHAVLLEIVM TFGLVYTVYA TAIDPKRGHL GTIAPLAIGF
      181 ILGANILAGG PFDGAAMNPA RAFGPALIGW RWKHHWVYWV GPFIGAALAG VMYEFLMIPA
      241 EAPRTHQPLA PEDY
//
LOCUS       MaTIP4-1       249 aa
DEFINITION  MaTIP4-1     249 aa
TITLE       MaTIP4-1
ORIGIN

        1 MAKIALGNHH EAAEPGCIRA VLAEVVLTFL FVFAGVGAAM AAEKMVGGDS IMGLTAVAVA
       61 HALVVAVMIS AGLHISGGHL NPAVTLGLAV GGHVTVVRSL LYVVAQLLGS TLACLLLKYL
      121 TGGLDTPVHT LAAGMGAVQG VIMEIVLTFS LLFSVYATMV DPKKGIIAGL GPLLVGLVVG
      181 ANILAGGPFS GASMNPARSF GPALAAWNWT DHWIYWVGPL AGGGLAGLVY EHLFMVSTHV
      241 PLPREDEGF
//
LOCUS       MaTIP4-2       254 aa
DEFINITION  MaTIP4-2     254 aa
TITLE       MaTIP4-2
ORIGIN
```

```
        1 MRKITLGSRN EAVEPDFVRS VFTELLLTFL FVFAGVGAVM TAEEVAGGED RIMWVVAAPA
       61 AAQAMLVAMI TAVGLDVSAG HLNPAVTIGF AAGGYVTVVR CVLYVIAQLL GSSMACLLLK
      121 YVAAGLDVLP VHALAAGMDP LQGVIMEAVF TFSMVFAIYA LIMDPKKGAI AGSAPLLIGL
      181 TVGANSLAGG AFSGASMNPA RSFGPALANW DWTNHWVYWL GPLVGSGLAG FAHHHLYVAG
      241 THGVLLPKDD EVGF
//
LOCUS       MaTIP4-3       255 aa
DEFINITION  MaTIP4-3     255 aa
TITLE       MaTIP4-3
ORIGIN

        1 MARIKLGSRK EMTDPEFARS VLTELLLTFL FVFVGVAASM TAGKMAGGQD SIMGLTAVAV
       61 AQAMLVAVMV AVGLDVSAGH LNPAVTIGFA AGGYVTVFRC VLYVIVQLLG SSMACLLLQY
      121 IAGGQAVPVH ALGVGIGPLQ GAIMEVVLTF SMVFSIYAII VDPKKGIVSV LAPLLIGLIV
      181 GANTLAGGPF SGASMNPARS FGPALATWDW TNHWVYWAGP FVGSGLAGFV YDHLYLMRPR
      241 DDLPGDEESI TKPLC
//
LOCUS       MaTIP5-1       263 aa
DEFINITION  MaTIP5-1     263 aa
TITLE       MaTIP5-1
ORIGIN

        1 MSSKLLCFFT SGVNFWLSAA SLRSYLAEFI STFFFVFAAV GSAISARMLT PDVTSDASSL
       61 VATALAQGFA LFAAVYIAAD ISGGHVNPAV TFGLAVAGHI GVPTAIFYWI SQLGGSILAC
      121 LLLRVASAGQ AIPTTGIGTE MTGFGGAVVE SAITFILVYT VYVAADPGGG GGDGKRKRKM
      181 RREVAGPLAV GLTAGACVLA AASLTGGSMN PARSFGPAVV SGNFKNHAVY WVGPLIGAAL
      241 AALVHQYLVF PSASSDAYPN STV
//
LOCUS       OsNIP1-1       284 aa
DEFINITION  OsNIP1-1     284 aa
TITLE       OsNIP1-1
ORIGIN

        1 MAGGDNNSQT TNGGSGHEQR AMEEGRKQEE FAADGQGCGL AFSVPFIQKI IAEIFGTYFL
       61 IFAGCGAVTI NQSKNGQITF PGVAIVWGLA VMVMVYAVGH ISGAHFNPAV TLAFATCRRF
      121 PWRQVPAYAA AQMLGATLAA GTLRLMFGGR HEHFPGTLPA GSDVQSLVLE FIITFYLMFV
      181 ISGVATDNRA IGELAGLAVG ATILLNVLIA GPISGASMNP ARSLGPAMIG GEYRSIWVYI
      241 VGPVAGAVAG AWAYNIIRFT NKPLREITKS GSFLKSMNRM NSST
//
LOCUS       OsNIP1-2       369 aa
DEFINITION  OsNIP1-2     369 aa
TITLE       OsNIP1-2
ORIGIN

        1 MAVVVDGVSP PWSKEAVVHL LSEFSSPDHV SISVTAHQPC LLKNGLEETE TTRASGLPNL
       61 RNDSINRVLI ESLVERLIAL PLSQGERWLG ERMEQQQEPW KKGKTARRSN FQRTLIADTD
      121 PWCMCMNKNL LILAEILGTY FMIFAGCGAV VVNQSTGGAV TFPGICAVWG LVVMVLVYTV
      181 SHISGAHFNP AVTVAFATCG RFRWKQVPSY VVAQVLGSTM ASLTLRVVFG GGGGGARGEH
      241 LFFGTTPAGS MAQAAALEFV ISFFLMFVVS GVATDNRAIG ELAGLAVGAT VAVNVLFAGP
      301 VTGASMNPAR SLGPAMVAGR YGGVWVYVAA PVSGTVCGAW AYNLLRFTDK PLRDIANTAS
      361 FLRRSSRRS
//
LOCUS       OsNIP1-3       286 aa
DEFINITION  OsNIP1-3     286 aa
```

```
TITLE       OsNIP1-3
ORIGIN

        1 MAGGEHGVNG QHEETRAMEE GSRDHQARCE NSEQDGGSKS SSNNHPMFSV QFAQKVIAEI
       61 LGTFFLIFAG CAAVAVNKRT GGTVTFPGIC ITWGLAVMVM VYSVGHISGA HLNPAVTLAF
      121 ATCGRFPWRR VPAYAAAQVA GSAAASAALR ALFGGAPEHF FGTAPAGSDV QSLAMEFIIT
      181 FYLMFVVSGV ATDNRAIGEL AGLAVGATVL VNVLFAGPIS GASMNPARTI GPAIILGRYT
      241 GIWVYIAGPV FGAVAGAWAY NLIRFTDKPL REITMTASFI RSTRRN
//
LOCUS       OsNIP1-4       273 aa
DEFINITION  OsNIP1-4     273 aa
TITLE       OsNIP1-4
ORIGIN

        1 MARREVDDSY TNGSVVEVVS IEEGSKMDKE DDHQNPQAPD GGDVVVCGMP MSFTFLQMLL
       61 AEFLATFFLM FAGLGAITVE EKKGAVTFPG VAVAWGAAVM AMVYAVGHVS GAHLNPAVTL
      121 GFAVAGRFPW RRAPAYALAQ TAAATAASVV LRLMFGGRHA PVPATLPGGA HAQSLVIEFV
      181 ITFYLMFVIM AVATDDQAVG HMAGVAVGGT IMLNVLFAGP VSGASMNPAR SIGPALVGSK
      241 YTALWVYILG PFAGAAAGAW AYSLIRLTGD RTD
//
LOCUS       OsNIP2-1       298 aa
DEFINITION  OsNIP2-1     298 aa
TITLE       OsNIP2-1
ORIGIN

        1 MASNNSRTNS RANYSNEIHD LSTVQNGTMP TMYYGEKAIA DFFPPHLLKK VVSEVVATFL
       61 LVFMTCGAAG ISGSDLSRIS QLGQSIAGGL IVTVMIYAVG HISGAHMNPA VTLAFAVFRH
      121 FPWIQVPFYW AAQFTGAICA SFVLKAVIHP VDVIGTTTPV GPHWHSLVVE VIVTFNMMFV
      181 TLAVATDTRA VGELAGLAVG SAVCITSIFA GAISGGSMNP ARTLGPALAS NKFDGLWIYF
      241 LGPVMGTLSG AWTYTFIRFE DTPKEGSSQK LSSFKLRRLR SQQSIAADDV DEMENIQV
//
LOCUS       OsNIP2-2       298 aa
DEFINITION  OsNIP2-2     298 aa
TITLE       OsNIP2-2
ORIGIN

        1 MASTTAPSRT NSRVNYSNEI HDLSTVQSVS AVPSVYYPEK SFADIFPPNL LKKVISEVVA
       61 TFLLVFVTCG AASIYGEDMK RISQLGQSVV GGLIVTVMIY ATGHISGAHM NPAVTLSFAF
      121 FRHFPWIQVP FYWAAQFTGA MCAAFVLRAV LYPIEVLGTT TPTGPHWHAL VIEIVVTFNM
      181 MFVTCAVATD SRAVGELAGL AVGSAVCITS IFAGPVSGGS MNPARTLAPA VASNVYTGLW
      241 IYFLGPVVGT LSGAWVYTYI RFEEAPAAAG GAAPQKLSSF KLRRLQSQSM AADEFDNV
//
LOCUS       OsNIP3-1       311 aa
DEFINITION  OsNIP3-1     311 aa
TITLE       OsNIP3-1
ORIGIN

        1 MEMAAPNGGG AAGMSSPVNG ASAPATPGTP APLFAGPRVD SLSYERKSMP RCKCLPAAVA
       61 EAWAPSAHGC VVEIPAPDVS LTRKLGAEFV GTFILIFFAT AAPIVNQKYG GAISPFGNAA
      121 CAGLAVTTII LSTGHISGAH LNPSLTIAFA ALRHFPWLQV PAYVAVQVLG SICAGFALKG
      181 VFHPFLSGGV TVPDPTISTA QAFFTEFIIT FNLLFVVTAV ATDTRAVGEL AGIAVGAAVT
      241 LNILIAGPTT GGSMNPVRTL GPAVAAGNYR QLWIYLIAPT LGAVAGAGVY TAVKLRDENG
      301 ETPRPQRSFR R
//
```

```
LOCUS       OsNIP3-3       276 aa
DEFINITION  OsNIP3-3     276 aa
TITLE       OsNIP3-3
ORIGIN

        1 MAENMVMVSS SDENHNQVAI DLCSASPVDR SLSAAAGGST TPRSPGFSMV VVPVESPEKT
       61 TGKPQTDDHD QQQGRAKEVP LVKKAAAEFI GTFILVFTVL STVVMDARHG GAETLVGVAA
      121 SAGLAVVAVV LSVVHISGSH LNPAVSLAMA ALGHLPPAHL LPYAAVQTAA SLAAAFLAKG
      181 VYRPARPAVM ATVPAAGVGA GEAFVVESKE LVAIAIAAAI MMNALVGGPS TGPSMNPART
      241 IGAAVATGEY RQMWIYLVAP PLGAIAGAAT YTLIKP
//
LOCUS       OsNIP3-2a       305 aa
DEFINITION  OsNIP3-2a     305 aa
TITLE       OsNIP3-2a
ORIGIN

        1 MEGGKMSSMG MDAASASVTV PPMQMQAGDQ SNRIAIIISP RAGSSKILPF ELVNGAANAG
       61 SQRHADPAES TPEAHHHLWH PVDLPKIKPP VPLVKKVGAE FFGTFTLIFT VLSTIIMDEQ
      121 HKGVESLLGI ATSAGLAVTV LVLSLIHISG CHLNPAVSIA MTVFGHLPPA HLLPYIAAQI
      181 LGSITASFAV KGMYHPVNPG IVTVPKVGTV EAFFLEFVTT FVLLFIITAL ATDPNAVKEL
      241 IAVAVGATIM MNALVAGPST GASMNPARTL GPAIATGRYT QIWVYLVATP LGAVAGEGFY
      301 FAIKL
//
LOCUS       OsNIP3-2b       329 aa
DEFINITION  OsNIP3-2b     329 aa
TITLE       OsNIP3-2b
ORIGIN

        1 MKNKKPTNSE KKKIQLAITV HIGIGYYTCV CMSGSKTHSA SKKMDEGSSP ASTSATAAAA
       61 AANLESTSFD DGRSHSSKIT PIELVVVNPE EPPPASRSRG HGPRRRSWRR RPCPPLAKKA
      121 AAEFVGTFIL IFAMLSTIVT DAQRGGVEGL VGVAASIGLA VAVLVMSLAH VSGAHINPAV
      181 SVAMAAFGRL QPAHLLPYAA AQVLGAVAAA AAVDGIFHPA SRGWMVSVPK VGTVEAFFLE
      241 FVTTFVLLFV ITAVSADPNA VKELIAVAVG GTAMMNVLVA GPSTGASMNP ARTLGTAIVA
      301 GNYTQIWVYM VSTPLGAIAG TGAYFAIKL
//
LOCUS       OsNIP4-1       286 aa
DEFINITION  OsNIP4-1     286 aa
TITLE       OsNIP4-1
ORIGIN

        1 MTTDHAGKKV DVVVVGNVDG EHVGVEQARH DLHEEAAAAA AADHHATRGL AIGFLIREVM
       61 VEGLASFLVV FWSCVAALMQ EMYGTLTFPM VCLVVAMTVA FVLSWLGPAH FNPAVTITFA
      121 AYRRFPVWPK LPLYVAAQLA GSLLACLSVN AVMRPRHDHF YGTAPVVVHG TRLPFLMEFL
      181 ASAVLMIVIA TVATDGTAGK TVGGIAIGAA VGGLGLVIGP VSGGSMNPAR TLGPAIVLGR
      241 YDGVWIYVVA PVAGMLVGAL CNRAVRLSHR IVAFLCGTSV GIAGSP
//
LOCUS       OsPIP1-1       289 aa
DEFINITION  OsPIP1-1     289 aa
TITLE       OsPIP1-1
ORIGIN

        1 MEGKEEDVRL GANRYSERQP IGTAAQGAGD DKDYKEPPPA PLFEPGELKS WSFYRAGIAE
       61 FVATFLFLYI TILTVMGVSK SSSKCATVGI QGIAWSFGGM IFALVYCTAG ISGGHINPAV
      121 TFGLFLARKL SLTRAIFYIV MQCLGAICGA GVVKGFQQGL YMGNGGGANV VASGYTKGDG
```

```
      181 LGAEIVGTFI LVYTVFSATD AKRNARDSHV PILAPLPIGF AVFLVHLATI PITGTGINPA
      241 RSLGAAIIYN KDHAWNDHWI FWVGPFVGAA LAAIYHQVII RAIPFKSRS
//
LOCUS       OsPIP1-2       288 aa
DEFINITION  OsPIP1-2     288 aa
TITLE       OsPIP1-2
ORIGIN

        1 MEGKEEDVRL GANKFSERQP IGTAAQGSDD KDYKEPPPAP LFEPGELKSW SFYRAGIAEF
       61 MATFLFLYIT VLTVMGVNNS TSKCATVGIQ GIAWSFGGMI FALVYCTAGI SGGHINPAVT
      121 FGLFLARKLS LTRALFYMVM QCLGAICGAG VVKGFQKGLY ETTGGGANVV APGYTKGDGL
      181 GAEIVGTFIL VYTVFSATDA KRNARDSHVP ILAPLPIGFA VFLVHLATIP ITGTGINPAR
      241 SLGAAIIYNR GHAWDDHWIF WVGPFIGAAL AAIYHQVVIR AIPFKSRS
//
LOCUS       OsPIP1-3       288 aa
DEFINITION  OsPIP1-3     288 aa
TITLE       OsPIP1-3
ORIGIN

        1 MEGKEEDVRL GANRYTERQP IGTAAQGAEE KDYREPPAAP VFEVEELTSW SFYRAGIAEF
       61 VATFLFLYIS ILTVMGVNKS ASKCATVGIQ GIAWSFGGMI FALVYCTAGI SGGHINPAVT
      121 FGLFLARKLS LTRAVFYMAM QCLGAICGAG VVKGFQRGLY MGSGGGANAV NPGYTKGDGL
      181 GAEIVGTFVL VYTVFSATDA KRNARDSHVP ILAPLPIGFA VFLVHLATIP ITGTGINPAR
      241 SLGAAIVYNR AHAWHDHWIF WVGPFIGAAL AAIYHVVVIR AIPFKSRD
//
LOCUS       OsPIP2-1       290 aa
DEFINITION  OsPIP2-1     290 aa
TITLE       OsPIP2-1
ORIGIN

        1 MGKDEVMESG GAAGEFAAKD YTDPPPAPLI DAAELGSWSL YRAVIAEFIA TLLFLYITVA
       61 TVIGYKHQTD ASASGADAAC GGVGVLGIAW AFGGMIFILV YCTAGISGGH INPAVTFGLF
      121 LARKVSLVRA ILYIVAQCLG AICGVGLVKA FQSAYFNRYG GGANTLAAGY SKGTGLAAEI
      181 IGTFVLVYTV FSATDPKRNA RDSHVPVLAP LPIGFAVFMV HLATIPITGT GINPARSIGA
      241 AVIFNNEKAW HNHWIFWVGP FVGAAIAAFY HQYILRAGAI KALGSFRSNA
//
LOCUS       OsPIP2-2       288 aa
DEFINITION  OsPIP2-2     288 aa
TITLE       OsPIP2-2
ORIGIN

        1 MAKDIEASAP EGGEFSAKDY TDPPPAPLID VEELTKWSLY RAVIAEFIAT LLFLYITVAT
       61 VIGYKHQSDA TVNTTDAACS GVGILGIAWA FGGMIFILVY CTAGISGGHI NPAVTFGLFL
      121 ARKVSLIRAV LYIIAQCLGA ICGVGLVKGF QSSYYARYGG GANELSDGYS KGTGLGAEII
      181 GTFVLVYTVF SATDPKRNAR DSHIPVLAPL PIGFAVFMVH LATIPITGTG INPARSLGTA
      241 VIYNKDKAWD DQWIFWVGPL IGAAIAAAYH QYVLRASAAK LGSYRSNA
//
LOCUS       OsPIP2-3       290 aa
DEFINITION  OsPIP2-3     290 aa
TITLE       OsPIP2-3
ORIGIN

        1 MAKDIEAAAA AEGGEYMAKD YSDPPPAPLI DAEELTKWSL YRAVIAEFVA TLLFLYITVA
       61 TVIGYKHQSD PGANAADAAC SGVGILGIAW AFGGMIFILV YCTAGVSGGH INPAVTFGLF
```

```
      121 LARKVSLVRA VLYIVAQSLG AICGVGLVKG FQSAFYVRYG GGANELSDGY SKGTGLAAEI
      181 IGTFVLVYTV FSATDPKRNA RDSHVPVLAP LPIGFAVFMV HLATIPITGT GINPARSLGA
      241 AVIYNQHKAW HDHWIFWVGP LIGAAIAAAY HQYVLRASAA KLGSSSSFRG
//
LOCUS       OsPIP2-4       286 aa
DEFINITION  OsPIP2-4     286 aa
TITLE       OsPIP2-4
ORIGIN

        1 MGKEVDVSTL EAGGARDYID PPPAPLVDVD ELGKWSLYRA LIAEFVATLL FLYVTVATVI
       61 GYKHQTDAAV NGADAACGGV GVLGIAWAFG GMIFILVYCT AGVSGGHINP AVTLGLFLAR
      121 KVSLVRALLY MAAQCLGAIC GVALVKGFQS SLYDRYGGGA NELAAGYSTG TGLAAEIIGT
      181 FVLVYTVFSA TDPKRNARDS HVPVLAPLPI GFAVFMVHLA TIPITGTGIN PARSLGVAVV
      241 YNNNKAWSDQ WIFWVGPFIG AAIAALYHQV ILRASARGYG SFRSNA
//
LOCUS       OsPIP2-5       283 aa
DEFINITION  OsPIP2-5     283 aa
TITLE       OsPIP2-5
ORIGIN

        1 MGKEADVEAG GVRDYEDPPP APLVDIDELG RWSLYRAVIA EFVATLLFLY VTVATVIGYK
       61 HQTDASASGD DAACGGVGVL GIAWAFGGMI FILVYCTAGI SGGHINPAVT FGLFLARKVS
      121 LVRAILYIVA QCLGAVCGVA LVKGFQSSFY DRYGGGANEL AAGYSKGTGL AAEIIGTFVL
      181 VYTVFSATDP KRNARDSHVP VLAPLPIGFA VFMVHLATIP VTGTGINPAR SLGAAVVYNN
      241 SKAWSDQWIF WVGPFIGAAI AALYHQIVLR ASARGYGSFR SNA
//
LOCUS       OsPIP2-6       337 aa
DEFINITION  OsPIP2-6     337 aa
TITLE       OsPIP2-6
ORIGIN

        1 MSKEVSEEPE HVRPKDYTDP PPAPLFDVGE LRLWSFYRAL IAEFIATLLF LYITVATVIG
       61 YKVQSSADQC GGVGTLGIAW AFGGMIFILV YCTAGISGGH INPAVTFGLL LARKVSVIRA
      121 VMYIVAQCLG GIVGVGIVKG IMKHQYNANG GGANMVASGY STGTALGAEI IGTFVLVYTV
      181 FSATDPKRNA RDSHVPVLAP LPIGFAVFMV HLATIPITGT GINPARSIGA AVIYNQKKAW
      241 DDHVSCSPLT ATLCSCVHFT KCLCLHMLNC SGSSGRGRSS ERWRRRRITS TSSGRQPSRR
      301 WAPSGATPAT ELRWPWLLCC CWPGWTWSYR LHVRECV
//
LOCUS       OsPIP2-7       290 aa
DEFINITION  OsPIP2-7     290 aa
TITLE       OsPIP2-7
ORIGIN

        1 MASKEEVAVE TVEGGAAAAK APYWDPPPAP LLDTSELGKW SLYRALIAEF MATLIFLYVS
       61 IATVIGYKNQ RATVDACTGV GYLGVAWSFG ATIFVLVYCT GGVSGGHINP AVTLGLFFGR
      121 KLSLVRTVLY VVAQCLGAIA GAGIVKGIMK RPYDALGGGA NTVSDGYSAA GALGAEIVGT
      181 FILVYTVFSA TDPKRTARDS FIPVLVPLPI GFAVFVVHLA TIPITGTGIN PARSLGAAVL
      241 YNQHAAWKDH WIFWVGPVIG AFLAAAYHKL VLRGEAAKAL SSFRSTSVTA
//
LOCUS       OsPIP2-8       280 aa
DEFINITION  OsPIP2-8     280 aa
TITLE       OsPIP2-8
ORIGIN
```

```
        1 MAAGSGSGSN PKDYQDPPPA PLVDTGELGK WSLYRAAIAE FTATLLLVCI SVSTVIGEKR
       61 QSGEGGAGVL GIAWAFGGLI FVLVYCTAGI SGGHMNPAVT FAMVLARRVS LPRAALYTMA
      121 QCVGAVCGAG LARAMHGGGQ YARHGGGANE LAAGYSAGAG VVAEMVGTFV LVYTVFSATD
      181 PKRKARDSHV PVLAPLPIGL AVLVVHLATI PITGTGINPA RSLGPALVLG LGTTKAWSHL
      241 WIFWVGPFAG AAAAMIYHHY ILRGAAAKAF ASSSYRSPHF
//
LOCUS       OsSIP1-1       246 aa
DEFINITION  OsSIP1-1     246 aa
TITLE       OsSIP1-1
ORIGIN

        1 MAVAAVRAAA ADAAVTFLWV LCVSTLGAST AAVTSYLRIH EGIHYALLVT VSLLSVLLFA
       61 FNLLCDALGG ASFNPTALAA FHAAGLSSPR HSSLFPLALR FPAQAAGAVG GAMAISELMP
      121 EQYKHMLGGP SLKVDLHTGA AAELVLTFVI TLAVLWIIVK GPRNPIVKTW MLSISTVCLV
      181 LTGAAYTGPS MNPANAFGWA YVNNRHNTWE QFYVYWICPF VGAVLAAWVF RAVFPPPAPK
      241 PKAKKA
//
LOCUS       OsSIP2-1       250 aa
DEFINITION  OsSIP2-1     250 aa
TITLE       OsSIP2-1
ORIGIN

        1 MSPAPPPSRG RIRPWLVVGD LVVAAMWVCA GALVKLAVYG VLGLGGRPEA DAVKVALSLV
       61 YMFFFAWLEG FTGGASYNPL TVLAGALASR AGPSLYLFAA FVRMPAQVFG SILGVKLIRA
      121 ALPKVGKGAP LSVGVHHGAL AEGLATFMVV IVSVTLKKKE MKGFFMKTWI SSIWKMTFHL
      181 LSSDITGGVM NPASAFAWAY ARGDHTTFDH LLVYWLAPLQ ATLLGVWVVT LLTKPKKIEE
      241 EADESKTKKE
//
LOCUS       OsTIP1-1       250 aa
DEFINITION  OsTIP1-1     250 aa
TITLE       OsTIP1-1
ORIGIN

        1 MPIRNIAVGS HQEVYHPGAL KAALAEFIST LIFVFAGQGS GMAFSKLTGG GATTPAGLIA
       61 AAVAHAFALF VAVSVGANIS GGHVNPAVTF GAFVGGNITL FRGLLYWIAQ LLGSTVACFL
      121 LRFSTGGLAT GTFGLTGVSV WEALVLEIVM TFGLVYTVYA TAVDPKKGSL GTIAPIAIGF
      181 IVGANILVGG AFDGASMNPA VSFGPALVSW SWESQWVYWV GPLIGGGLAG VIYEVLFISH
      241 THEQLPTTDY
//
LOCUS       OsTIP1-2       252 aa
DEFINITION  OsTIP1-2     252 aa
TITLE       OsTIP1-2
ORIGIN

        1 MPVSRIAVGA PGELSHPDTA KAAVAEFISM LIFVFAGSGS GMAFSKLTDG GGTTPSGLIA
       61 ASLAHALALF VAVAVGANIS GGHVNPAVTF GAFVGGNISL VKAVVYWVAQ LLGSVVACLL
      121 LKIATGGAAV GAFSLSAGVG AWNAVVFEIV MTFGLVYTVY ATAVDPKKGD LGVIAPIAIG
      181 FIVGANILAG GAFDGASMNP AVSFGPAVVT GVWDNHWVYW LGPFVGAAIA ALIYDIIFIG
      241 QRPHDQLPTA DY
//
LOCUS       OsTIP2-1       248 aa
DEFINITION  OsTIP2-1     248 aa
TITLE       OsTIP2-1
ORIGIN
```

```
        1 MVKLAFGSLG DSFSATSVKA YVAEFIATLL FVFAGVGSAI AYGQLTNGGA LDPAGLVAIA
       61 IAHALALFVG VSVAANISGG HLNPAVTFGL AVGGHITILT GLFYWIAQLL GASIACLLLK
      121 FVTHGKAIPT HGVAGISELE GVVMEIVITF ALVYTVYATA ADPKKGSLGT IAPIAIGFIV
      181 GANILAAGPF SGGSMNPARS FGPAVAAGNF AGNWVYWVGP LIGGGLAGLV YGDVFIGSYQ
      241 PVADQDYA
//
LOCUS       OsTIP2-2       248 aa
DEFINITION  OsTIP2-2     248 aa
TITLE       OsTIP2-2
ORIGIN

        1 MSGNIAFGRF DDSFSAASLK AYVAEFISTL VFVFAGVGSA IAYTKLTGGA PLDPAGLVAV
       61 AVCHGFGLFV AVAIGANISG GHVNPAVTFG LALGGQITIL TGVFYWIAQL LGAIVGAVLV
      121 QFCTGVATPT HGLSGVGAFE GVVMEIIVTF GLVYTVYATA ADPKKGSLGT IAPIAIGFIV
      181 GANILVAGPF SGGSMNPARS FGPAVASGDY TNIWIYWVGP LVGGGLAGLV YRYVYMCGDH
      241 APVASSEF
//
LOCUS       OsTIP3-1       264 aa
DEFINITION  OsTIP3-1     264 aa
TITLE       OsTIP3-1
ORIGIN

        1 MSTAAARPGR RFTVGRSEDA THPDTIRAAI SEFLATAIFV FAAEGSILSL GKLYQDMSTP
       61 GGLVAVSLAH ALALAVAVAV AVNISGGHVN PAITFGALLG GRLSLIRALF YWLAQLLGAV
      121 VATLLLRLTT GGMRPPGFAL ASGVGDWHAV LLEATMTFGL MYAYYATVID PKRGHVGTIA
      181 PLAVGFLLGA NMLAGGPFDG AGMNPARVFG PALVGWRWRH HWVYWLGPFV GAGLAGLLYE
      241 YLVIPSADAA PHGGAHQPLA PEDY
//
LOCUS       OsTIP3-2       265 aa
DEFINITION  OsTIP3-2     265 aa
TITLE       OsTIP3-2
ORIGIN

        1 MLPGRHTPRR ADAAAAAAAM EPLVPGATRA ALSEFVATAV FVFAAEGSVY GLWKMYRDTG
       61 TLGGLLVVAV AHALALAAAV AVSRNASGGH VNPAVTFGVL VGRRISFARA ALYWAAQLLG
      121 AVLAVLLLRL ASGGMRPMGF TLGHRIHERH ALLLEVVMTF GLVYTVYATA VDRRSGGGDI
      181 APLAIGLVAG ANILAGGPFD GAAMNPARAF GPALVGWNWR HHWVYWLGPL IGAGMAGALY
      241 EFVMAEQPEP PAAADTRLPV AAEDY
//
LOCUS       OsTIP4-1       251 aa
DEFINITION  OsTIP4-1     251 aa
TITLE       OsTIP4-1
ORIGIN

        1 MAKEVDPCDH GEVVDAGCVR AVLAELVLTF VFVFTGVAAT MAAGVPEVAG AAMPMAALAG
       61 VAIATALAAG VLVTAGFHVS GGHLNPAVTV ALLARGHITA FRSALYVAAQ LLASSLACIL
      121 LRYLTGGMAT PVHTLGSGIG PMQGLVMEII LTFSLLFVVY ATILDPRSSV PGFGPLLTGL
      181 IVGANTIAGG NFSGASMNPA RSFGPALATG VWTHHWIYWL GPLIGGPLAG LVYESLFLVK
      241 RTHEPLLDNS F
//
LOCUS       OsTIP4-2       256 aa
DEFINITION  OsTIP4-2     256 aa
TITLE       OsTIP4-2
```

```
ORIGIN

        1 MPLLPMTKLE LGHRGEAWEP GCLRAVAGEL LFTFLFVFIG VASTITAGKA AGGAGEAAAV
       61 TAAAMAQALV VAVLATAGFH VSGGHLNPAV TLSLAVGGHI TLFRSALYVA AQLAGSSLAC
      121 LLLRCLTGGA ATPVHALADG VGPVQGVAAE AVFTFTLLLV ICATILDPRR AAPPGTGPLL
      181 TGLLVGANTV AGGALTGASM NPARSFGPAL ATGEWAHHWV YWVGPLAGGP LAVVAYELLF
      241 MDVEDAGGAH QPLPQE
//
LOCUS       OsTIP4-3       251 aa
DEFINITION  OsTIP4-3     251 aa
TITLE       OsTIP4-3
ORIGIN

        1 MAKLALGHHR EATDPGCLRA VVAELLLTFL FVFSGVGSAM AAAKLGGGGD TIMGLTAVAA
       61 AHALVVAVMV SAGLHVSGGH INPAVTLGLA AGGHITLFRS ALYAAAQLLG SSLACLLLAA
      121 LTGGEEAVPV HAPAPGVGAA RAVAMEAVLT FSLLFAVYAT VVDRRRAVGA LGPLLVGLVV
      181 GANILAGGPY SGASMNPARS FGPALAAGEW ADHWIYWVGP LIGGPLAGLV YEGLFMGPPG
      241 HEPLPRNDGD F
//
LOCUS       OsTIP5-1       269 aa
DEFINITION  OsTIP5-1     269 aa
TITLE       OsTIP5-1
ORIGIN

        1 MANICANMKR CFSPPALRAY FAEFFSTFLF VFIAVGSTIS ARMLTPDETS DASSLMATAV
       61 AQAFGLFAAV FIAADVSGGH VNPAVTFAYA IGGHITVPSA IFYWASQMLG STFACLVLHY
      121 ISAGQAVPTT RIAVEMTGFG AGILEGVLTF MVVYTVHVAG DPRGGGFGGR KGPAATALGA
      181 LVVGAVTGAC VLAAGSLTGA SMNPARSFGP AVVSGHYSNQ AVYWAGPMVG AAVAALVHQA
      241 LVFPTVPEPA PAPATNESAR HGSVQTVVV
//
LOCUS       PaNIP1-1       279 aa
DEFINITION  PaNIP1-1     279 aa
TITLE       PaNIP1-1
ORIGIN

        1 MALDNMPEEN FNSVRNIEEG RTESYVYTER TCGSLLPSVK FAQKVAAEII GTFFLIFIGC
       61 GSIIIDKKTN GSITHLGVSI VWGLAVMIII YAIGHISGAH LNPAVTLAFA AVRRFPCTEV
      121 PAYIGAQVFA AISAGFVLRL MFGDVANIGA TVPSGSDIQS FFLEIFVTFL LMFVISAVAT
      181 DTRAIGELAG MAVAGTVGMN VAMSGPISGA SMNPARTIGP ALAGNKYTSI WIYMVAPVVG
      241 AILGAVSYNM IRLTDKPVRE LTRTGSFLKS QRASRNGSR
//
LOCUS       PaNIP1-2       373 aa
DEFINITION  PaNIP1-2     373 aa
TITLE       PaNIP1-2
ORIGIN

        1 MQRQIAREIL SRVQRKGGER QSEEQSRGRS SSTLSRLIRK HITIHQSHLS SLILFHSECA
       61 GGVNGRAREE SLHRMASGMA ILVSGSALVQ GLIMALDNMP DQENVNAVRN VEEGRIESHV
      121 YTMRTCGSFL PSVTFAQKVV AEIIGTFFLI FIGCGSIVID KKTNGSITHL GVAIVWGLAA
      181 MIIIYSIGHI SGAHLNPAVT LAFAVVRRFP CTHVPAYIGA QVFAAISAGF VLRLMFGDVA
      241 YIGATVPSGS DMQSFFLEIF VTFLLMFVIS AVATDTRAIG ELAGMAIGAT IGMNVAISGP
      301 ISGASMNPAR TIGSAVAGDK YTSIWIYMVA PVLGAIIGAV SYNMIRLTDK PVREITKSGS
      361 FLKSQRSSRS GSI
//
```

```
LOCUS       PaNIP1-3       269 aa
DEFINITION  PaNIP1-3     269 aa
TITLE       PaNIP1-3
ORIGIN

        1 MALDNMPEQE NVNGVRNIEE GLIESHVYTE RTCRSFLPSV TFVQKVVAEI IGTFFLIFIG
       61 CGSVVIDKKT NGSITHLGVS IVWGLAVMII IYSIGHISGA HLNPAVTLAF AAVRRFPWTQ
      121 VPAYIGAQVF AAICAGFVLR LMFGDVAYIA ATVPSGSDMQ SFVLEIFVTF LLMFIGELAG
      181 MAVGATITMN VAISGPISGA SMNPARTIGS AVAGNKYTSI WIYMVAPVLG AIIGAMSYNM
      241 IRLTDKPVRE LTKSGSFLKS QRSSRSGSI
//
LOCUS       PaNIP1-4       293 aa
DEFINITION  PaNIP1-4     293 aa
TITLE       PaNIP1-4
ORIGIN

        1 MDTGLTSSQI STTPTSLPGL KEGFQDIDPD RLREIEEGRI ERHVAGQIVS GSCMPSLTFV
       61 RKVVAEIIGT FFLIFSGCGA VVIDQKSNSS ITHLGVSLVW GMAVMIIIYA IGHISGAHLN
      121 PSVTLASALV RRFPWAQVPA YIGAQVFASI SAGFVLRLMF GEVAHMAATI PTGSDMQSFI
      181 LEILITFFLM FVVSAVSTDT RAIGELAGLA VGTTVAMNVI IAGPISGASM NPARTIGSAV
      241 AGNKYTGIWI YMIAPVLGAV MGAWSYNMIR LTDKPVREIT KSGSFLSSLR SRR
//
LOCUS       PaNIP1-5       293 aa
DEFINITION  PaNIP1-5     293 aa
TITLE       PaNIP1-5
ORIGIN

        1 MRTTSPSCPS PSLPCLNAGS RDIIPEQENM CCRDVEENRM ESHDPGRIIC WSWMPSLTFA
       61 RKVVAEIIGT FFMIFAGCGS VIIDKKTDGS ITHLGVSLVW GMVVMILIYT IGHISGAHLN
      121 PSVTLAFAVV RRFPWTQVPA YIGAQVFASI SAGFVLRLMF GEVAHMAATV PAGSTMQSFV
      181 LEIIVTFFLM FVVSAVATDT RAIGELAGLA VGTTVAMNVI VAGPISGSSM NPARTIGSAV
      241 SGNKYTSIWI YMVAPVVGAI MGAMSYNMIR LTDKPVREIS KSGSFLKSQH SSR
//
LOCUS       PaNIP1-6       316 aa
DEFINITION  PaNIP1-6     316 aa
TITLE       PaNIP1-6
ORIGIN

        1 RNADRVVALL GRGRIDGPAE ERHAMNSDGE ERCTRGGVST IKTAPLIRRG DRGERARTVN
       61 GTLNGVMASH FHSVGGGEVH APIAWLVLRL EHNWKLGSTG MATENSPDMQ TVAFRNIVTQ
      121 NCSPDPQAVT FRNIEDGQTE GRVAGRTSGS FIPSVTFLKK VAAELIGTFF LIFAGCGSVV
      181 VDKKTHGSIT HLGVSIVWGM TVMILVYATG HISGAHFNPA VTLAFATVRR FPCIHVPAYM
      241 AAQIFASISA AFVLRLMFGE VAHMAATVPS GSDMQSFAFE IFTTFLLMFV ISAVATDTRA
      301 VDLYGIVPLI LVISDS
//
LOCUS       PaNIP2-1       212 aa
DEFINITION  PaNIP2-1     212 aa
TITLE       PaNIP2-1
ORIGIN

        1 EMDAENGSSQ LMTISSQEIE NQEARNVKEG SQFYRKDKHC PNRCMDFVLP NLLQNITTET
       61 ISTFILVFVT CGCSILDHDS RSLVSELEAS MASGFIVTVM IYLVGHISGA HMNPAVTIAF
      121 ATVRHFPWKQ VLAYITTQLG GSIEACFALR VMLKPVSNMG ITIPFGKVLQ ALAMEFMVSF
      181 VLMFVTSAVA TDTWYPSSSC SLPQQSQPIT VQ
```

```
//
LOCUS       PaNIP2-2       221 aa
DEFINITION  PaNIP2-2     221 aa
TITLE       PaNIP2-2
ORIGIN

        1 MDAENGSSQL VNISGQEVQD QEAGNVGSLF YKKDKQCPDG CMDFVPPTLL QKITAETIST
       61 FILVFVTCGS SILDHASPPL VSKLGGSVAS GLIVTVMIYS VGHISGAHMN PAVTIAFATV
      121 RHFPWKQVPA YITAQLGGSL AACFALRVML KAVSNTGITI PSGTVLQALA MEVVVSFVLM
      181 FVTSAVATDS SASRIKSLRS LKDKVFTLAT HFEAVLGVLG L
//
LOCUS       PaNIP2-3       294 aa
DEFINITION  PaNIP2-3     294 aa
TITLE       PaNIP2-3
ORIGIN

        1 MDAENSSRQL VNISGEEIEQ QEAGNVKERS LFYKKQCPNG CMDFVPPTLL QKITAETIST
       61 FILVFVTCGS SILDHRSPPL VSELGGSVAS GLIVMVMIYS VGHISGAHMN PAVTIAFATV
      121 RHFPWKQVPA YITAQLGGSL AACFALRVLL KAVSNTGITI PSGTVLQALV MEVVVSFVLM
      181 FVTSAVATDS SAIGELAGIA VGSMVMISSI FAGPISGGSM NPARSLGPAI VSNNYKAIWV
      241 YLVGPIAGTV MGACSYSIIR LTEKPLQTIS LRRASSFSSS KDGDSKSTVP NGLI
//
LOCUS       PaNIP3-1       230 aa
DEFINITION  PaNIP3-1     230 aa
TITLE       PaNIP3-1
ORIGIN

        1 MRQNTIEAAR AEIVVIEEAA KVLETLVLPC IGSFTEHLIL LGDHLQLRPS IAEYECTASC
       61 FEICPPCKNP SQVIAKFIGT FILIFVGTTT AIVNQKTDGS ISLLGMAASG GLAIMIIILS
      121 TGHISVAHVN PSLTFAFAAL RQFPWIQVPA YMGAQVLGSI CASFTLKLIF HPFLSGGVTI
      181 PLGSYAQAFA LEFIITFNLM FMVTAVATDT RAVGELAGIV VGAMAMTTAQ
//
LOCUS       PaNIP3-2       275 aa
DEFINITION  PaNIP3-2     275 aa
TITLE       PaNIP3-2
ORIGIN

        1 MTDCEDIPSA PQTPGTPGAP LFGVRVDKGS SGKRTLLQGC NSCLSMEAWA EERMLSDLPA
       61 ALPSASLAKK VIAEFIGTFI LIFAGTATAI VNQKTDGSVS LLGLAASGGL AIMIVILSTG
      121 HISGAHVPAY MGAQVLGSIC ASFTLKLIFH PFMSGGVTIP SGSYGQAFAL EFIITFNLMF
      181 VVTAVATDTR AVGELAGIAV GATVMLNILI AGSNSGASMN PVRTLGPAIA AGNYKGIWIY
      241 LLAPVVGALC GAAGYTVVRL KGEDNQGRPT RSFRR
//
LOCUS       PaNIP3-3       305 aa
DEFINITION  PaNIP3-3     305 aa
TITLE       PaNIP3-3
ORIGIN

        1 MESENSTPIQ SAPATPGTPG APLFRFRYKS SVPNENQSKQ HRCNCIPLKL NRCECLPMVA
       61 WGKDEPFFQR ASVILLSRKV VAEFLGTFIL IFAGTSTAIV DEKTGGKVSL IGKAASSGLG
      121 IMIVILSTGH ISGAHANPSL TIAFAAFRHF PWAQVPFYLA AQVLGSISAA FALKGIFNPF
      181 MGGGVTVPSG SDGQAFSLEF IITFNLMFVV TAVATDTRAV GELAGIAVGA TVMLNILIAG
      241 SNSGGSMNPV RTLGPAIAAG NYEAIWVYIV APITGALLGA GAYTLIRLKP DPDDESKRSV
      301 RSFRR
```

```
//
LOCUS       PaNIP3-4       290 aa
DEFINITION  PaNIP3-4     290 aa
TITLE       PaNIP3-4
ORIGIN

        1 MKVRPEWEAA MINQNSVEED RMNGGAHNLQ ASSAMVPAIT EKRYLRLLTL VCAEMVGTFI
       61 VVFSICAVVA VGEESQGRIG LMEYAATGGF AIMIVVFSIG HISGAHVNPA VTIAFAAAQQ
      121 FPWAQVPLYV SAQLTSSILA TFIAKKVYGV KGELAMTKPL SDSAKAFWVE LIATFFIMFL
      181 ASSLSMDARA IGQLSGIAVG ASIAMGVVIT ASVSGASMNP ARSFGPAVVA NNYDDIWIYL
      241 LGPTIGAVGG SLLYRLLRLQ QQLTPASSLP PPPPDEREST NSTFTSETRA
//
LOCUS       PaPIP1-1       285 aa
DEFINITION  PaPIP1-1     285 aa
TITLE       PaPIP1-1
ORIGIN

        1 MEDVSVGASK YSERQSLGIS AQTQRESKDY NEPGPAPLFE PEELRSWSFW RAGIAEFMAT
       61 FLFLYVTILT VMGVKRSPSM CQSVGIQGIA WSFGGMIFAL VYCTAGISGG HINPAVTFGL
      121 FLARKVSLPR TVFYMICQCL GAMCGAGVVK GMQKGMYEVE GGGANFVAHG YSKGDGLGAE
      181 IVGTFVLVYT VFSATDAKRS ARDSHVPVLA PLPIGFAVFL VHLATIPITG TGINPARSLG
      241 VAIIYDRSHA WDDQWIFWVG PLVGAALAAI YHQLIIRAIP FKSRS
//
LOCUS       PaPIP1-2       324 aa
DEFINITION  PaPIP1-2     324 aa
TITLE       PaPIP1-2
ORIGIN

        1 MSRTLGLSGR IQRDVGKGLS RKVERVTVKV AEMEGKEEDV KLGANKYSER QPLGTAAQTM
       61 EKDYTEPGPA PLFEPGEFRS WSFWRAGIAE FMATFLFLYI TILTVMGVKR SDDGSNGVCT
      121 GSVGIQGIAW AFGGMIFCLV YCTAGISGGH INPAVTFGLF LARKLSLPRA VFYMVCQCLG
      181 AICGAGVVKG FMESEYQMDG GGANVVAPGY TKGDGLGAEI VGTFVLVYTV FSATDAKRSA
      241 RDSHVPLLAP LPIGFAVFLV HLATIPITGT GINPARSLGA AIIYNKDHAW DDMWIFWVGP
      301 FIGAALAAFY HVIVIRAIPF KTRS
//
LOCUS       PaPIP2-1       284 aa
DEFINITION  PaPIP2-1     284 aa
TITLE       PaPIP2-1
ORIGIN

        1 MAKEGGKEMQ EQQQGFVAKD YKDPPPAPLV DINEFKLWSF YRALIAEFIA TLLFLYITIA
       61 TVIGHSRNKA DCGSVGVLGI AWSFGGMIFV LVYCTAGISG GHINPAVTFG LFLARKVSLP
      121 RAVMYMIAQC LGAICGAGLV KAFQKPYYDR YGGGANVVAH GYTKGVGLAA EIIGTFVLVY
      181 TVFSATDPKR SARDSHVPVL APLPIGFAVF MVHLATIPIT GTGINPARSF GAAVIYGHKH
      241 SWDDQWIFWV GPMVGAAAAA AYHQHILRAT AIKALGSFRS NPQV
//
LOCUS       PaPIP2-2       384 aa
DEFINITION  PaPIP2-2     384 aa
TITLE       PaPIP2-2
ORIGIN

        1 MQNPLHKSGE NVHKRLDGPY LKEAVLGGRT KLLSSSMKLN FAPPHKYNSK DHQTPSSSRP
       61 FIASATSGLC VSALPYRSEL LFYKPRRSFE FISSSRSSVQ AVMTKEEGKE LEQQGFAPKD
      121 YTDPPPAALI DANEFKLWSL YRALIAEFIA TLLFLYITIA TVIGHSRTSA DCGSVGVLGI
```

```
      181 AWSFGGMIFV LVYCTAGISG GHINPAVTFG LFLARKVSLP RAILYMIAQC LGAICGAGLV
      241 KAFQKSFYDR YGGGANFVHP GYTKGVGLAA EIIGTFVLVY TVFSATDPKR SARDSHVPVL
      301 APLPIGFAVF MVHLATIPIT GTGINPARSF GAAVIYGHKQ SWDDHWIFWV GPFVGAALAA
      361 AYHQYILRAA AVKALGSYRS NVDV
//
LOCUS       PaPIP2-3       201 aa
DEFINITION  PaPIP2-3     201 aa
TITLE       PaPIP2-3
ORIGIN

        1 MEWGGLFPPM GRGCLSCNFI ETVIIVGRIV TSVFVRCNSL WSFYRALIAE FIATLLFLYI
       61 TVATVIGHKR TQANCGSVGV LGIAWAFGGM IFVLVYCTAG ISGGHINPAV TFGLFLARKV
      121 SLPRAVLYMI AQCLGAICGV GLVKAFQKSY YDKYGGGANV VAYGYTKGVG LAAEIIGTFI
      181 LVYTVFSATD PKRSARDSHV P
//
LOCUS       PaPIP2-4       282 aa
DEFINITION  PaPIP2-4     282 aa
TITLE       PaPIP2-4
ORIGIN

        1 MRKEEGRDSE RQGFVAKDYT DPPPAALIDT HEFKLWSFYR ALIAEFTATL LFLYITIATV
       61 IGHSRTLADC GSVGVQGIAW AFGGMIFVLV YCTAGVSGGH INPAVTFGLF LARKVSLPRA
      121 ILYMIAQCLG AICGTRLVKA LQNSSYDKYG GGANYVHQGY TKGAGLGAEI IGTFVLVYTV
      181 FSATDPKRSA RDSHVPVLAP LPIGFAVFMV HLATIPITGT GINPARSFGT AVISGQSWND
      241 QWIFWIGPFV GAALAATYHR YILRASAIKA LALGSFRSNS NV
//
LOCUS       PaPIP2-5       327 aa
DEFINITION  PaPIP2-5     327 aa
TITLE       PaPIP2-5
ORIGIN

        1 MTKEEGKEME QHGFAPKDYT DPPPASLIDA GEFRLWSFYR ALIAEFIATL LFLYITIATV
       61 IGHSRTSTNC GSVGVLGIAW SFGGMIFVLV YCTAGISGGH INPAVTFGLF LARKVSLPRA
      121 ILYMIAQCLG AICGTGLVKA FQKSFYDKYG GGANYVHHGY TKGVGLAAEI IEGVGLAAEI
      181 IGTFVLVYVV FSATDPKRSA RDSHVPVLAP LPIGFAVFMV HLATIPITGT GINPARSFGA
      241 AVIYGHKQSW DDHWIFWVGP FIGAALAAAY HQYILRAAAI KALGSFRTSL RTLIGRSIAD
      301 YSAAERAMDV CSFGLNPVLL QKQSECL
//
LOCUS       PaPIP2-6       247 aa
DEFINITION  PaPIP2-6     247 aa
TITLE       PaPIP2-6
ORIGIN

        1 MGLLPCDHSR VHCHAAVSVH NMATVIGHKR NQAACGSVGL LGIAWAFGGM IFVLVYCTAG
       61 ISGGHINPAV TFGLFLARKV SLPRAVLYMV AQCLGAICGC GLVKAFQKSY YDQYGGGANS
      121 VAHGYTKGVG LSAEIIGTFV LVYTVFSATD PKRNARDSHV PVLAPLPIGF AVFMVHLATI
      181 PITGTGINPA RSFGAAVIYG HQKIWDEHWI FWVGPFLGAA GAAAYHQYIL RAGAIKALGS
      241 FRSNPHV
//
LOCUS       PaPIP2-7       257 aa
DEFINITION  PaPIP2-7     257 aa
TITLE       PaPIP2-7
ORIGIN
```

```
        1 MINEEGNELE KRGTVAKDYT DPPPAALIDI NEFKLWSFYR ALIAEFIATL LFLYITIATV
       61 IGHSRTLTKC GSVGVLGIAW SFGGMIFVLV YCTAGISGGH INPAVTFGLL LAKKVTLPRA
      121 ILYMVAQCLG AICGTGLGVG LAAEIIGTFV LVYTVFSATD PKRSARDSHV PVLAPLPIGF
      181 AVFMVHLATI PITGTGINPA RSFGPAVIYG HKQSWDDHWI FWVGPFAGAA LAAAYHQYIL
      241 RAAAIKALGS FRSNANV
//
LOCUS       PaPIP2-8       259 aa
DEFINITION  PaPIP2-8     259 aa
TITLE       PaPIP2-8
ORIGIN

        1 MVNEGRSQKA QYEEPPPAPF LDRNEFYLWS FYRAIIAEFV ATLLFLYVTI ATVIGNANQK
       61 KPCGGVGTLG IAWSFGGMIF VLVYCTAGVS GGHINPAVTF GLFIARKCLG AVCGAGMVKA
      121 LQKNYYSAGG GGANTVKEGY ASETALAAEI AGTFVLVYTV FCATDPKSNA RDSHVPALAP
      181 LSVGFAVFMV HLATIPITGT GINPARSFGA AVIYGHKKSW NDHWIFWVGP LIGATIAAAF
      241 HKYVIRATLK PHRGEHNHV
//
LOCUS       PaPIP2-9       292 aa
DEFINITION  PaPIP2-9     292 aa
TITLE       PaPIP2-9
ORIGIN

        1 MARTHQGGSG KAEYEDPSPG PVLDVNEFRL WSFYRAIVAE FVATFLFVYI TVSTIIGKAN
       61 QKNPCAGVGT LGISWCVGGM IFILVYCTAG ISGGHINPAV TFGMLVARKV SLNRAVLYIV
      121 AQCLGAICGA GMVKALQKTY YSTGGGGVNA VSEGYTNETA LAAEIIGTFV LVYTVFSATD
      181 PKRNARDSHV LAPLPIGFAV IVVHFATIHI TGTGINPARS FGPAVVYGHQ KSWDDHWIFW
      241 VGPLIGAALA AAYHQYVLRA TGFKAWAPSV DNSLRPPVPA KGEQFKGLGI LP
//
LOCUS       PaPIP2-10       277 aa
DEFINITION  PaPIP2-10     277 aa
TITLE       PaPIP2-10
ORIGIN

        1 MGQGGRSSKV EYRDPLPAPF LDVKEFCLWS FYRAIIAEFV ATFLFVYVIV STIIGQANEK
       61 NPCGGVGILG IAWCVGGMIF VLVYCTAGIS GGHINPAVTF GLLVARKVTL NRAVLYMVAQ
      121 CAGAVCGAGM VRELQKTYYY RGGGGVNSVS EGYTKEKALA AEIIGTFALV YTVFSATDPK
      181 RNARDSHVLA PLPIGFTVFV VNLATIPITG TGINPARSFG PAVVYGHQKS WDEHWIFWAG
      241 PLIGAAAAAA YHQCVLRVGG LKALGSSRSF SITSSTV
//
LOCUS       PaPIP2-11       283 aa
DEFINITION  PaPIP2-11     283 aa
TITLE       PaPIP2-11
ORIGIN

        1 MAAMEVDGGE QQTRDYEEHP PAPLLDSLEL KLWSFYRAVI AEFVATLLFL YITMTTVVEN
       61 KQSKGTCGGV GLLGEAWASG GMIFVLVYCI SGISGGHVNP AVTFALFLAR KVSLPRAVLY
      121 IVAQCLGALC GTALVKGIQG SFYASNGGGS NSVSPGYSKG SALLAEIIGT FVLVYTVFSA
      181 TDPKRKARDS HVPVLAPLPI GFAVFLVHLA TIPITGTGIN PARSFGPAVI YGHKKSWDDL
      241 WIFWVGPLIG AAVAAAYHQY VLKASGFGLK NLGSLRSHPA SAT
//
LOCUS       PaPIP2-12       193 aa
DEFINITION  PaPIP2-12     193 aa
TITLE       PaPIP2-12
ORIGIN
```

```
        1 MCVGGHVNPA VTFALFLARK VSLPRAVLYI VAQCLGALCG TALVRGIQGS FYASTGGGSN
       61 SVSAGYSKGS ALLAEIIGTF VLVYTVFSAT DPKRNARDSH IPVLAPLPIG FAVFLVHLAT
      121 IPITGTSINP ARSFGPAVIY GHKKSWDDLW IFWVGPLVGA AIAAAYHQYV LRAGGIGLKS
      181 LRSFRSQPTS LRA
//
LOCUS       PaPIP2-13       275 aa
DEFINITION  PaPIP2-13     275 aa
TITLE       PaPIP2-13
ORIGIN

        1 MEMEGEDYEE HPPAPLLDSL ELKLWSFYRA VIAEFVATLL FLYITMTTVV ENKQSKGTCG
       61 GVGLLGEAWA FGGMIFVLVY CISGISGGHV NPAVTFALFL ARKVSLPRAV LYVVAQCLGA
      121 VCGTALVKGI QGSFYASNGG GSNSVSPGYS KGSALLAEII GTFVLVYTVF SATDPKRKAR
      181 DSHVPVLAPL PIGFAVFLVY LATNSSTGTG INPARSFGPA VIYGHKKSRD DLWIFWIGPL
      241 IGAAVATAYH RYLLRAGAFG SKNLGSLRSH PASAI
//
LOCUS       PaPIP2-14       228 aa
DEFINITION  PaPIP2-14     228 aa
TITLE       PaPIP2-14
ORIGIN

        1 DYEEHPPAPL LDSLELKLWS FYRAVIAEFV ATLLFLYITM TTVVENKQSK GTCGGVGLLG
       61 EAWAFGGMIF VLVYCISGIS GGHVNPAVTF ALFLARKVSL PRAVLYVVAQ CLGAVCGTAL
      121 VKGIQGSFYA SNGGGSNSVL APLPIGFAVF LVYLATNSIT GTGINPARSF GPAVIYGHKK
      181 SRDDLWIFWI GPLIGAAVAT AYHRYLLRAG AFGSKNLGSL RSHPASAI
//
LOCUS       PaPIP2-15       326 aa
DEFINITION  PaPIP2-15     326 aa
TITLE       PaPIP2-15
ORIGIN

        1 MLATNRLTKL LKSNEIGSAV QIIFATSRLC NLARSTIQIS SSTNGPDRLS PFATSRLGLT
       61 VSSKLRNLNW TFEGTEAPVP DGAYDVVFNS FGSSNELSGL VPDVLTKCYI FLKVMEFLLK
      121 SVSLLESEAS MRCVSPYETI SVCDFMDSPS GSSLFPVLQV TFHFHLEFLM SRFPASPRRD
      181 AGGGHISPSV TFGLFVALKS TGGGGANSVS PGYTKETALS AEIIGTFILV YTVFCATYPK
      241 SIARDRHAPV LAPLAVGFAV FLLHLATIPI TGTGINPARS FGAAVIYGHK KSWNDHVLDL
      301 VYSWYIWPPF PSLGRASILP EALEQL
//
LOCUS       PaPIP2-16       233 aa
DEFINITION  PaPIP2-16     233 aa
TITLE       PaPIP2-16
ORIGIN

        1 MEMEGEDYEE HPPAPLLDSL ELKLWSFYRA VIAEFVATLL FLYITMTTVV ENKQSKGTCG
       61 GVGLLGEAWA FGGMIFVLVY CISGISGGHV NPAVTFALFL ARKVSLPRAG SALLAEIIGT
      121 FVLVYTVFSA TDPKRKARDS HVPVLAPLPI GFAVFLVYLA TNSSTGTGIN PARSFGPAVI
      181 YGHKKSRDDL WIFWIGPLIG AAVATAYHRY LLRAGAFGSK NLGSLRSHPA SAI
//
LOCUS       PaSIP1-1       238 aa
DEFINITION  PaSIP1-1     238 aa
TITLE       PaSIP1-1
ORIGIN
```

```
        1 MGIVKLAIGD AAITFLWVFG ASCLGAGTSI IASNLGVQGP MTLLITTSLL FLLVFLFSFL
       61 GQVMGGATWN PTASAAAFAL GVGNDNLISM SIRFPAQAAG AVGGALAIME LMPASYKHML
      121 GGPSLKVDLH RGAIAEGVLT FLISFMVLLI IMKGPKSSFW KSWMISLVTI ILVLAGSGYT
      181 GPSMNPANAF GWAYVNNRHN TWEQLYVYWI TPFIGSILAA WILRLISPPG SSKKEKKA
//
LOCUS       PaSIP2-1       241 aa
DEFINITION  PaSIP2-1     241 aa
TITLE       PaSIP2-1
ORIGIN

        1 MGGLKLVVAD MVVSFLWVLS GALVRVLIAV VASHFLQLKG NEELLKWALT VAVMFTFSWL
       61 GKLTNGASYN PVTVLSNAFA YSRAESLFTL AARIPAQAFG SLLGVKCVNR MIPHGGQGPL
      121 LKVGLMSGMA VEGILTFTIV MVALIASARG PTSFFLRTWI VSITKLSLSI VGANFTGPSM
      181 NPATAFGWAY SRGQHRTKEH VYVYWLAPLE ACLLAVWTFG SLVVPSQKKQ TRQKLKTLKE
      241 N
//
LOCUS       PaTIP1-1       257 aa
DEFINITION  PaTIP1-1     257 aa
TITLE       PaTIP1-1
ORIGIN

        1 MPFRGLAVGR PEEVIHPDAL KAALAEGIST LIFVFAGEGS GIAFAKLTSN ASTTPAGLVA
       61 VALAHGLGLF VAVAVSANIS GGHVNPAVTF GALVGGHITL LRGIVYWLAQ LIGATVACLL
      121 LKFTTSGLST SAFSLYSGVG VGNALVFEIV MTFGLVYTVY ATAIDPKKGS LGTIAPICIG
      181 FIVGANILAG GAFDGASMNP ARAFGPALVS WSWENHWIYW VGPLLGGALA GVVYELFMIA
      241 PEPTHKPTHE PLPAHDY
//
LOCUS       PaTIP1-2       253 aa
DEFINITION  PaTIP1-2     253 aa
TITLE       PaTIP1-2
ORIGIN

        1 MPFRGVAIGR PEEVTHPTAL KAALAELIST LIFVFAGEGS GMAFAKLTSD ASTTPAGLVA
       61 VALAHGLGLF VAVAVGANIS GGHVNPAVTF GALVGGHITL LRGILYWIAQ LIGATVACLL
      121 LKYTTGGLST SAFSLSSGVG VGNALVFEIV MTFGLVYTVY ATAIDPKKGT LGTIAPICIG
      181 FIVGANILAG GAFDGASMNP ARAFGPALVS WTWENHWIYW VGPLLGGGLA GVIYELFMIS
      241 NEPTHERLSS EDY
//
LOCUS       PaTIP1-3       253 aa
DEFINITION  PaTIP1-3     253 aa
TITLE       PaTIP1-3
ORIGIN

        1 MPVRRIALGR AEEATHPDSI RAALAEFFST LIFVFAGEGS VMSSSPRSSS SSLAKDPSWL
       61 TLTGGDSTTP SGLVAVALAH ALGLFVAVAV AINISGGHVN PAVTFGALMG GHISILRGIL
      121 YWIAQLLGAV VASLLLKFTT NGRSTSPFAV SSGVGSWNAV VLEIVMTFGL VYTVYATAID
      181 AKRGSLGTIA PLAIGFIVGA NILAGGAFDG ASMNPARAFG PALVSGKWRY HWIYWVGPLI
      241 GGGFAGLLYG SVP
//
LOCUS       PaTIP2-1       152 aa
DEFINITION  PaTIP2-1     152 aa
TITLE       PaTIP2-1
ORIGIN
```

```
        1 MREPYQKSQA FERQAIVACL LLKFVTGGLT TPTHNVAAGM STIEGVVMEI VITFALVYTV
       61 YATAADPKKG SLGTIAPIAI GFIVGANILA AGPFSGGSMN PARSFGPAVV SGDFTNNWVY
      121 WVGPLVGGGL AGAVYGGVFI GSHSHAPLSQ DY
//
LOCUS       PaTIP2-2       298 aa
DEFINITION  PaTIP2-2     298 aa
TITLE       PaTIP2-2
ORIGIN

        1 MDGASCHCSM TARCGGSKLP LGLARWDGLV YKRKNCLLDL LAGMDWMGRV EIAFGSFDDS
       61 FKLEAIRTYV AEFISTLLFV FAGVGSVMAY DKLTSDASLS PAGLVGVGLA HGLALFVTVS
      121 IAANISGGHV NPAVTFGLAL GGHITLLRGV FYWIAQLLGA IVACLLLKFT TGGLTTPIHS
      181 VASGMSTGEG VVMEIIITFA LVYTVYATAA DPKKGDLGTI APIAIGFIVG ANILAAGPFS
      241 GGSMNPARSF GPAVVSGDFT DNWVYWVGPL IGGGLAGIVY GGIFIGDDSH VPLPVSDF
//
LOCUS       PaTIP4-1       252 aa
DEFINITION  PaTIP4-1     252 aa
TITLE       PaTIP4-1
ORIGIN

        1 CPGGSRRGRP SDRDEAARPD CVRAVFAELI CTFLFVFAGV GSAMAMEQMS VPAKSPAGLT
       61 VVALAHAFVV FAMISAGFNI SGGHLNPAVT LGLAVGRHIT LIRSLLYWIA QLLASVLACF
      121 LLNFLTGGLA TPVHTLSSGM TYFQGVIMEI VLTFSLLFTV YATAVDPKKG SVGITAPLCV
      181 GLVVGANILA GGPFSGASMN PARSFGPALV TGIWKDHWVY WVGPLVGGGL AGFLYENIFI
      241 YETHTPLPDV EF
//
LOCUS       PpeNIP1-4       195 aa
DEFINITION  PpeNIP1-4     195 aa
TITLE       PpeNIP1-4
ORIGIN

        1 SNSFSISSPK LVLSTGHTAV QEAGATGTQK KRTVKAEIST SIFQRMMAEL VGTYILIFSG
       61 CGAALVNNIQ SLTIVGIALV WGLAFAATAY AVGHVSGAHF NPAVTIALAA GRRFPCKHAY
      121 HWSFYEPCQS FGPATVTGIY KNIWVYTAGP ILGAIAATMV YSVLRVPTPV GSDDSVLRAP
      181 TSISKFPNLQ QSVDT
//
LOCUS       PpeNIP3-3       298 aa
DEFINITION  PpeNIP3-3     298 aa
TITLE       PpeNIP3-3
ORIGIN

        1 MKAFSKDEIS VPSPQTSNCT STSDHSNKDD QEMGSNAMSD EGEVSNNSAF FCFPFHMDLN
       61 LVRAVFAEMV GTFMLMFCVC GIIASTQLMR GEVGLMEYAA TAGLTVVVVI FSIGSISGAH
      121 VNPAVTVAFA TLGHFPWCRV PVYILAQTMG SVLATYIGRL VYGIKPDLMT TRPLQSPAAA
      181 FCVELIATFM IMFLAASLTH QAHAVGYLSG FVVGIAIGLS VLITGPVSGG SMNPARSLGP
      241 AIVAWNFQDL WIYICGPTIG AVAGARLFQI LRLQPCSPSN TSSSNIRLLG QPLPYEAT
//
LOCUS       PpeNIP1-3       263 aa
DEFINITION  PpeNIP1-3     263 aa
TITLE       PpeNIP1-3
ORIGIN

        1 MALETQESDG IEEEISKIEK GLVSTSHNPG TNTSDDFRSS AAPPSLPQKL IAELIGTYFV
       61 IFAGCGSVAV NKIYGNVTFP GVCVTWGLIV MAMIYSVGHI SGAHFNPAVT ITFGVLRRLS
```

```
      121 FREVPSYIVA QLLGSTMASG TIAVLLDITP KAYFGTVPVG SNGQSLVFEI IISFLLMFVI
      181 SGASTDSRAI GDLAGVAVGM TILLNVFIAG PVSGASMNPA RSLGPAIVKH VYKGLWVYIV
      241 GPITGCLAGG IAYNFIRSKP LAS
//
LOCUS       PpeNIP3-2       267 aa
DEFINITION  PpeNIP3-2     267 aa
TITLE       PpeNIP3-2
ORIGIN

        1 MSYDRKSMPR CKCLPVNAPT WGQSHTCFTD FPTPTVSLTR KLGAEFVGTF ILMFAASAGP
       61 IVNQKYNGAE TLIGNAACAG LGVMIVILST GHISGAHLNP SLTIAFAALR HFPWVQVPAY
      121 IAAQVSGSIC ASFALKGAFH PYMSGGTTVP TVSTGQAFAL EFIITFNLLF VVTAVATDTR
      181 ALGELAGLAV GATVMLNILI AGPSSGGSMN PVRTLGPAVA AGNYTKLWVY LVAPTLGALA
      241 GAGTYTAVKL REDEVDEPVR EARSFRR
//
LOCUS       PpeNIP1-2       259 aa
DEFINITION  PpeNIP1-2     259 aa
TITLE       PpeNIP1-2
ORIGIN

        1 MEEGGSSGTD STNSGICSSS EIVQLTQKVI AEVIGTYFVV FIGCGSVAVN KIYGSVTFPG
       61 IAITWGLIVM VMVYSVGHIS GAHFNPAVTV TFAIFRHFPV KEVPLYVVAQ VLGSILASGT
      121 LCLLLDVPQT AYFGTLPVGS DIRSLIIEII ASFLLMFVIS GVATDNRAIG ELAGIAVGMT
      181 IVIDVFVAGP VSGASMNPAR SLGPAIVMHT YKGLWIYFAG PIVGTVLGGF AYNLIRFTDK
      241 PLRELTKSAS FIKSLSTKP
//
LOCUS       PpeNIP3-1       307 aa
DEFINITION  PpeNIP3-1     307 aa
TITLE       PpeNIP3-1
ORIGIN

        1 MDNNNEEVPS APSTPVTPGT PGAPLFGGFK ADHRSSGIGR KSLLKSCSRC FTVEDWALEE
       61 GTLPKVSCAL PHPPVSLARK VGAEFLGTFI LIFAGTATAI VNQKTQGTET LIGLAASTGL
      121 AVMIVILSTG HISGAHLNPS VTIAFAALKH FSWKHVPVYV GAQVLASICA AFALKVIFHP
      181 IMGGGVTVPS GSYGQAFALE FIISFNLMFV VTAVATDTRA VGELAGIAVG ATVMLNILIA
      241 GETTGASMNP VRTLGPAIAA NNYKAIWVYL TAPFLGALFG AGTYTAVKLP EEDGDHIEKP
      301 STRSFRR
//
LOCUS       PpeNIP1-1       281 aa
DEFINITION  PpeNIP1-1     281 aa
TITLE       PpeNIP1-1
ORIGIN

        1 MAETSGANGN GTHVFNVEDG DGNSHRLSQS STKTNQESSS FGLSVPFMQK VIAEVLGTYF
       61 VVFAGCGAVV VNLSTDKTVS FPGISIVWGL VVMVMVYSVG HISGAHFNPA VTIAFAITKR
      121 FPWKQVPAYI AAQVLGSTLA SGTLRLIFQG HQNHFAGTIP TGNPWQSFAI EFVITFYLMF
      181 VISGVATDNR AIGELAGLAV GATVLLNVMF AGPISGASMN PARSVGPAIV STQYKHLWVY
      241 IFAPTLGAVC GALVYNVIRF SDKPLREITK SSSFLKGVGS K
//
LOCUS       PpeNIP2-1       292 aa
DEFINITION  PpeNIP2-1     292 aa
TITLE       PpeNIP2-1
ORIGIN
```

```
        1 MATKHPEPGN HNTNELVSVQ ENHPISQNPT FEQQYYPPGF FRKVVAEMIA TYMLVFVTCG
       61 SAALAASDEH KVSRLGASIA GGLVVTVMIY AVGHISGAHM NPAVTLAFAA VRHFPWKQVP
      121 IYWVAQLAGS ISASFTLSVL LHPIKHVGTT SPSGSDFQAL IAEIVMTFSM MFIASAVATD
      181 TKAIGELAGV AVGSAVCITS IFAGPISGGS MNPARTIGPA IASTYYKGIW VYVVGPVIGT
      241 LLGSWSYNFI RVSDNPTQPI SPPPPPRSFA FKLRRMKSDN GQVPCKDPLD SA
//
LOCUS       PpeNIP5-1       222 aa
DEFINITION  PpeNIP5-1     222 aa
TITLE       PpeNIP5-1
ORIGIN

        1 LGAEFVGTFI LVFSAAAGPI VNEKYHGVET LLGNAACSGL AVMVVILSTG HISGAHLNPS
       61 VTLAFATFRH FPWSQVPLYI IVQVSASISA SFALKGVFHP FMSGGVTVPS VTHGQAFSLE
      121 FIVTFILMFT ITAVATDTRA VGEMAGVAIG ATVLLDILVA GPTTGGSMNP IRTLGPAIAA
      181 GNYKGLWIYL VAPTFGALAG AAAYTTVKLP DNKPPNQDFF IR
//
LOCUS       PpePIP2-4       281 aa
DEFINITION  PpePIP2-4     281 aa
TITLE       PpePIP2-4
ORIGIN

        1 MSKEVSEEGQ AHHHGKDYVD PPPAPLIDSD ELKRWSFYRA LIAEFIATLL FLYITVSTVI
       61 GNKVQSGPCD GVGLLGIAWA FGGMIFVLVY CTAGISGGHI NPAVTFGLFL ARKVSLIRAV
      121 AYIVAQSLGA IVGVGLVKAF QKHNYNSQGG GANTVAPGYS KGTALGAEII GTFVLVYTVF
      181 SATDPKRSAR DSHVPVLAPL PIGFAVFIVH LATIPITGTG INPARSFGPA VIFNNEKAWD
      241 DQWIFWVGPF VGALAAAAYH QYILRAAAIK ALGSFRSNPT N
//
LOCUS       PpePIP1-3       290 aa
DEFINITION  PpePIP1-3     290 aa
TITLE       PpePIP1-3
ORIGIN

        1 MEGKEEDVKL GANKFSERQP IGTSAQTQDE GKDYKEPPPA PLFEPGELTS WSFYRAGIAE
       61 FIATFLFLYI TILTVMGVVK SPSKCSTVGI QGIAWAFGGT IFALVYSTAG ISGGHINPAV
      121 TFGLFLARKL SLTRAVFYIV MQTLGAIAGA AVVKGFEKSR NFELLGGGAN SVAHGYTKGS
      181 GLGAEIVGTF VLVYTVFSAT DAKRSARDSH VPILAPLPIG FAVFLVHLAT IPITGTGINP
      241 ARSLGAAIIY NKKHAWDDHW IFWVGPFIGA ALAALYHVVV IRAIPFKSKS
//
LOCUS       PpePIP2-3       284 aa
DEFINITION  PpePIP2-3     284 aa
TITLE       PpePIP2-3
ORIGIN

        1 MAKDIEVGGG QHRFSGKDYQ DPPPAPLIDA EELGQWSFYR AIIAEFIATL LFLYITVLTV
       61 IGYKSQSEGD QCGGVGILGI AWAFGGMIFV LVYCTAGISG GHINPAVTFG LFLARKVSLV
      121 RAILYMVAQS LGAICGVGLV KAFQNAYFTK YGGGANGLAD GYSQGTGLAA EIIGTFVLVY
      181 TVFSATDPKR NARDSHVPVL APLPIGFAVF IVHLATIPIT GTGINPARSL GAAVIYNNDK
      241 AWDDQWIFWV GPFIGAAIAA FYHQYILRAG AVKALGSFRS SSNI
//
LOCUS       PpePIP1-2       286 aa
DEFINITION  PpePIP1-2     286 aa
TITLE       PpePIP1-2
ORIGIN
```

```
        1 MEGKEEDVKL GANKFPERQP IGTSAQTDKD YKEPPPAPLF EPGELTSWSF YRAGIAEFIA
       61 TFLFLYITIL TVMGVNRAPS KCASVGIQGI AWAFGGTIFA LVYSTAGISG GHINPAVTFG
      121 LFLARKLSLT RAVFYIVMQT LGAIAGAGVV KGFEKKQYEL LGGGANVVNH GYTKGGGLGA
      181 EIVGTFVLVY TVFSATDAKR NARDSHVPIL APLPIGFAVF LVHLATIPIT GTGINPARSL
      241 GAAIIFNKDR AWDDHWIFWV GPFIGAALAA IYHQIVIRAI PFKSRG
//
LOCUS       PpePIP2-2       281 aa
DEFINITION  PpePIP2-2     281 aa
TITLE       PpePIP2-2
ORIGIN

        1 MAKDVEGAEH GEFAAKDYHD PPPTPFFDPE ELTKWSFYRA LIAEFVATLL FLYITVLTVI
       61 GYKSQSSGDQ CGGVGILGIA WAFGGMIFVL VYCTAGISGG HINPAVTFGL FLARKVSLPR
      121 AVLYIVAQSL GAICGVGLVK AFQKTYYEEY GGGANELAAG YNKGTGLGAE IIGTFVLVYT
      181 VFSATDPKRN ARDSHVPVLA PLPIGFAVFI VHLATIPITG TGINPARSFG AAVIYNKDKA
      241 WDDQWIFWLG PFIGAAIAAL YHQYILRAGA IKALGSFRSN A
//
LOCUS       PpePIP1-1       329 aa
DEFINITION  PpePIP1-1     329 aa
TITLE       PpePIP1-1
ORIGIN

        1 MLFIISLTYF NSSHSSHSKN QSFQLCREGR PKPEFLRERE NREMEGKEED VRLGANKFSE
       61 RQPIGTSAQT DKDYKEPPPA PLFEPGELKS WSFWRAGIAE FIATFLFLYI TVLTVMGVSR
      121 SRSKCSTVGI QGIAWAFGGS IFALVYSTAG ISGGHINPAV TFGLFLARKL SLTRAVFYIV
      181 MQTLGAIAGA GVVKGFQKNQ YELLGGGANV VNHGYTKGDG LGAEIVGTFV LVYTVFSATD
      241 AKRNARDSHV PILAPLPIGF AVFLVHLATI PITGTGINPA RSLGAAIIYN KDRAWDDHWI
      301 FWVGPFIGAA LAALYHQIII RAIPFKTRD
//
LOCUS       PpePIP2-1       287 aa
DEFINITION  PpePIP2-1     287 aa
TITLE       PpePIP2-1
ORIGIN

        1 MAKDVEVAER GSFSAKDYHD PPPAPLFDAV ELTKWSFYRA LIAEFIATLL FLYVTVLTVI
       61 GYKSQTDTTV NADACGGVGI LGIAWAFGGM IFVLVYCTAG ISGGHINPAV TFGLFLARKV
      121 SLVRAVLYMV AQSLGAIAGV ALVKAFQESY YTKYGGGANE LSQGYSKGVG LGAEIIGTFV
      181 LVYTVFSATD PKRSARDSHV PVLAPLPIGF AVFIVHLATI PITGTGINPA RSLGAAVIYN
      241 KEKAWDDQWI FWVGPFIGAA IAAFYHQFIL RAGAVKALGS FRSNPHV
//
LOCUS       PpeSIP1-2       240 aa
DEFINITION  PpeSIP1-2     240 aa
TITLE       PpeSIP1-2
ORIGIN

        1 MGVIKAALGD AVLTSLWVFS APSMGVFTVI IASFLGIQAS SLLGLFITTI VATILVLAFS
       61 LIGKVLGDAS FNPSTTASFY AAGLTPGTSL FSMAVRFPAQ AAGGVGGAKA ILQVMPKQYK
      121 HMLKGPFLKV DLYTGVIAEG LLTFVLCFSL LVIILRGPRN PILQIWLLSI ATVGLVVAGG
      181 GYTGPSMNPA NAFAWAYVNN WHNTWELFLV YWIGPFIGAT LAASVFKTLL PPPIAKEKKA
//
LOCUS       PpeSIP1-1       244 aa
DEFINITION  PpeSIP1-1     244 aa
TITLE       PpeSIP1-1
ORIGIN
```

```
        1 MGAIKAAVGD TMLTFLWISC ASMLGLATSF IANAVGVQDL PWPPLFITTA LVFVLVFVFT
       61 FLGDALGGAS FNPTGTASFY AAGLGADTLL SMALRFPAQA FGAVGGVLAI NEVMPHQYKH
      121 MLGGPSLKVD VHTGAIVEGV LTFVISFLVL IIILKGPRSP VLKMLLLSVV TVTLVVSGSV
      181 YTGPSMNPAN AFGWAYLNNW HNTWEQFYVY WICPFIGAIL AGWIFRAVFP PLLAKGKKKK
      241 AKKA
//
LOCUS       PpeSIP2-1       236 aa
DEFINITION  PpeSIP2-1     236 aa
TITLE       PpeSIP2-1
ORIGIN

        1 MGRIGLLVSD FTMSFMWVWS GVLIKMYVYN NLGFGHEPTG EIIKSTLSIV NMFFFALLGK
       61 ITNGGTYNPL TVFAAAITGD FTRFLFTVAA RIPAQVIGSI AGVKLIIQLF PEVGLGPRLN
      121 VDIHRGALTE GLLTFAIVTI SLGLARNIPG SFFMKTWISS VSKLTLHILG SDLTGGCMNP
      181 ASVMGWAYAR GTHVTKEHIL VYWLAPIEAT LLAIWTFRVL VPQLKEEKVD KKAKSE
//
LOCUS       PpeTIP1-3       252 aa
DEFINITION  PpeTIP1-3     252 aa
TITLE       PpeTIP1-3
ORIGIN

        1 MPIRNIAVGR PEEAYHPDAL RAGLAEFIST LIFVFAGEGS GMAFAKLTHG AATTPAGLVA
       61 AALAHGFGLF VAVSVSANIS GGHVNPAVTF GAFVGGNISL LRGILYWIAQ LLGSAVACGL
      121 LRFATNYQTT SAFALSEGVG VWNAFVFEIV MTFGLVYTVY ATAIDPKRGS VGTIAPIAIG
      181 FVVGANILAG GAFDGASMNP AVSFGPALVS WNWHNHWIYW AGPLIGGGLA GLIYEFVFIG
      241 NSSHEPLPTA DY
//
LOCUS       PpeTIP1-2       252 aa
DEFINITION  PpeTIP1-2     252 aa
TITLE       PpeTIP1-2
ORIGIN

        1 MPINKIAVGT PGEASHPDAI RAAFAEFFST LIFVFAGEGS GMAFNKLTNN GSTTPSGLVA
       61 ASLAHAFALF VAVSVGANIS GGHVNPAVTF GAFIGGNITL LRGILYWIAQ LLGSVVACLL
      121 LKFATGGWET AAFSLSSGVS VWNALVFEIV MTFGLVYTVY ATAVDPKKGN VGIVAPIAIG
      181 LIVGANILAG GAFDGASMNP AVSFGPAVVS WSWTHHWVYW AGPLIGAAVA ALVYDNIFIG
      241 DGAHEPLPNN DF
//
LOCUS       PpeTIP2-2       248 aa
DEFINITION  PpeTIP2-2     248 aa
TITLE       PpeTIP2-2
ORIGIN

        1 MVKLAFGSFG DSFSVGSLKS YLAEFIATLL FVFAGVGSAI AYGKLTSAAL DPAGLVAVAI
       61 AHAFALFVGV SIAANISGGH LNPAVTLGLA IGGNITILTG IFYWIAQLLG SIVASFLLKF
      121 VTQLDVPTHT LASGVGAIEG VVFEIIITFA LVYTVYATAA DPKKGSLGII APIAIGFIVG
      181 ANILAAGPFS GGSMNPARSF GPAVASGDFT DNWIYWVGPL IGGGLAGLVY GDIFIGSYSP
      241 VPAAEDYA
//
LOCUS       PpeTIP3-1       256 aa
DEFINITION  PpeTIP3-1     256 aa
TITLE       PpeTIP3-1
ORIGIN
```

```
        1 MPPRRYAFGR ADEATHPDSM RATLAEFVAT FIFVFAGEGS VLALGKIYKD SGTSAAELIA
       61 IALAHAFSLF SAVSTSINVS GGHVNPAVTF GALIGGRLSV VRALYYWVAQ LLGAIVASLL
      121 LRLVTNGMRT VAFSMASGVG EWHGLILEIV MTFGLVYTVY ATAIDPKRGS LGTMAPLAIG
      181 LIVGANILVG GPFDGASMNP ARAFGPALVG WRWRNHWIYW VGPFIGGGLA ALIYEYMVIP
      241 TETPHHTHQP LAPEDY
//
LOCUS       PpeTIP1-1       252 aa
DEFINITION  PpeTIP1-1     252 aa
TITLE       PpeTIP1-1
ORIGIN

        1 MPVSRIAIGN PAEFGQPDAL KAALAEFISV LIFVFAGEGS GMAFSKLTDG ASTTPAGLIA
       61 AALAHAFALF VAVSIAANIS GGHVNPAVTF GAFIGGNISL VRSVLYWIAQ LLGAVIACLL
      121 LRFATGGLET SAFSLSSGVS VWNAVVLEIV ITFGLVYTVY ATALDPKKGN IGIIAPIAIG
      181 FIVGANILVA GAFDGASMNP AVSFGPAVVS WTWDNHWVYW LGPFIGAAIA ALVYDLIYIT
      241 PDTHEQLPTT DY
//
LOCUS       PpeTIP4-1       249 aa
DEFINITION  PpeTIP4-1     249 aa
TITLE       PpeTIP4-1
ORIGIN

        1 MAKIALGTTS EAALPELWRA LVVEFITTFL FVFAGVGSAM ATDKLEADAL VALFATAVTH
       61 ALVVAVMISA GHISGGHLNP AVTLGLLVGG HITVFRSLLY WIDQLLAAAA ACYLLEYLTG
      121 GLTTPIHSLA SGVGYLQGVI WEIILTFSLL FTVYATIVDP KKGALNGLGP TLTGFVVGAN
      181 ILAGGAFSGA SMNPARSFGP ALVSWNWTDH WVYWVGPLIG GGLAGCIYEN FFIFRPTTHL
      241 PIPTEEEAF
//
LOCUS       PpeTIP2-1       248 aa
DEFINITION  PpeTIP2-1     248 aa
TITLE       PpeTIP2-1
ORIGIN

        1 MAKIAFGRFD DSFSLGSLKA YLAEFISTLL FVFAGVGSAI AYNKLTSNAA LDPAGLVAIA
       61 IAHGFALFVA VAIGANISGG HVNPAVTFGL ALGGQITVLT GIFYWIAQLL GAIVAAFILK
      121 FVTGGLTIPI HSLAAGVGAI QGVIFEIIIT FALVYTVYAT AADPKKGALG TIAPMAIGFI
      181 VGANILAAGP FSGGSMNPAR SFGPAVASGD FHDNWIYWVG PLIGGGLAGL IYGNVFFHTE
      241 HAPLVNEY
//
LOCUS       PpeTIP5-1       255 aa
DEFINITION  PpeTIP5-1     255 aa
TITLE       PpeTIP5-1
ORIGIN

        1 MARIALTTRF RQAITPNALR SYLAEFISTF FFVFAVVGSM MSSRKLMPDA ASDPASLVVV
       61 AIANAFALAS AVYIAANASG GHVNPAVTFG MAVGGHISVP NAICYWISQM VASVMACLLL
      121 KVTVVGQHVP AYAITEEITG FGASVLEGVL TFGLVYTVYA AGDPRNGAVG GIGPLAIGLM
      181 AGANVLATGP FSGGSMNPAC AFGSAVVAGS FKNQAVYWVG PLIGAAVAGL LYDNVVFPTQ
      241 VPDSLTGVTE GVGVF
//
LOCUS       PpeXIP1-2       314 aa
DEFINITION  PpeXIP1-2     314 aa
TITLE       PpeXIP1-2
```

```
ORIGIN

        1 MAENRPVVED DRVHPFSSTP MSEHWNVEEV KKSSPSTLSK KLGLHELFSL KVWRASLAEL
       61 VGSAVLVFAI DTIVISSYET RTTTPNLVMA ILISITVAIL LLATNPISGG HINPVVTLSA
      121 VFVGLISLSR AAVYILSQCA GAVLGALALK AVVNSSIEET FSLGGCTLTV IAPGPHGPII
      181 IGIETTQALW LEIICTFVFL FASIWMAFDH RQAHAVGRVV VFSIVGTVVG LLVFISTTVT
      241 AVKGYAGVGM NPARCLGPAL IRGGHLWNGH WVFWVGPIIA CVTFYLYIKI IPRQHFHMDG
      301 YTQDTVNIVK TLPQ
//
LOCUS       PpeXIP1-1       304 aa
DEFINITION  PpeXIP1-1     304 aa
TITLE       PpeXIP1-1
ORIGIN

        1 MEGLVMTPNE ADEKLPKSAG RSCEAVGVEN SLRLKFLATI GAHEYFSPEM WRAAFTELVA
       61 TASLLFTLTS SIISCLESGE VDPKLLVPIA VFIIAFLFLL VTVPLSGGHM SPVFTFIAAL
      121 KGVITFARAS IYILAQCIGS ILGFLIIKTV MDQNAAQKYS LGGCTIKGSG STSGVGTQTA
      181 LMVEFACTFV VLFVGVTVAF DKRRCKELGL AMVCAVVAGA MALAVFVSIT VTGRVGYAGV
      241 GLSPARCLGP ALLQGGRLWD GHWVFWVGPI LACSVYYCVS LNLPNEGLKS VEEECDILKG
      301 EGNV
//
LOCUS       PpGIP1-1       370 aa
DEFINITION  PpGIP1-1     370 aa
TITLE       PpGIP1-1
ORIGIN

        1 MGYNEGQVFA AELLGTTTLC VVGLGGIANA VLPGTKGHGI GFLGIAFCFG LGVYLPLQFV
       61 GHISGFFNPA VALAAAVVDD ITWKRFVLCL CAEMLGGFLG GFLVWLTYIP HFQPLEFLES
      121 TEESICDCCD LEANGVAIIK QELSEPAHRY ASAGTRDNVT TGRVQSEDYE HPMTSFWHEL
      181 LRKIMGCGSH SSELKKESDK GSMDRLHQAR AKSMKSACGP SVKQRIAEDQ EVKLIVFCTR
      241 PSIAKRFWFH CVWAEGSLTF LLTYGAFAIV NRGKLMLNEY AAEKELYKSG IEPALIGLLV
      301 FALVLCGGGM TGPALNPARD LGPRLVHWLV PIPGKGRSEW GYSWVPVVAP CLGAIVGALF
      361 ANAMRDIRPS
//
LOCUS       PpNIP3-1       301 aa
DEFINITION  PpNIP3-1     301 aa
TITLE       PpNIP3-1
ORIGIN

        1 MVKVETAAAA QAPLTTVPTL FALPPERQLS IQSAGMERQL STTSATSAKC FPVECMIESS
       61 YFTTTPVVSPP NISLTRKFAT ELIGTFVLIF AATATPIVNE KTKGSVTLLG NAATAGLAIM
      121 IVIFATGHIS GAHVNPAITI AFASLRHFPW VQVPFYIAAQ VLGSIAASFT LKGIFHPYMH
      181 GGVTLPQGAY WPSFLLEFII SFNLMFVITA VATDTRAVGE LAGIAVGACV MMNIMIAGST
      241 SGASMNPVRT LGPAIAVNNY KGIWLYMLGP VLGMLAGATA YTAVRLKEED PPRLPVRVFH
      301 R
//
LOCUS       PpNIP5-3       290 aa
DEFINITION  PpNIP5-3     290 aa
TITLE       PpNIP5-3
ORIGIN

        1 MAEIKLRMPA SLSAEEYPTV TLGNKVTQDP SRNGDEPLVV VQGTTPASPY NVAVSLHWPS
       61 VTLQKKLVAE VISTFILVFT GCGAVMVNAI SNGKVTPVGI SLVFGLVITI MIYAVGHISG
      121 AHMNPAVTLA FAIAKHFPWT QVPMYIVAQC GGSVFASFLL RWILHPAASE GATIPAGSDI
```

```
      181 QSFLLEIVIT FILMFVVAAV ATDTRARGEL AGIAVGSCVA LNALMAGPIS GASMNPARSL
      241 GPAVASGNYR SIWVYIAGPI IGALVGILAY NCIRLPDTEA QCDKPAKNSI
//
LOCUS       PpNIP5-1a       295 aa
DEFINITION  PpNIP5-1a     295 aa
TITLE       PpNIP5-1a
ORIGIN

        1 MAEIKIRMPP AAGPLSAEDI LTTEAGTKGS QDLKQTGQLP VVITQEVERG RYSTFVTSLR
       61 PRTSLIQKLV AEIISTFILV FTGCGAVMVN EISNGKVTSV GVSLAFGLVV TIMIYAVGHI
      121 SGAHMNPAVT LAFAVARHFP WTQVPLYAAA QCIGSITASF MLRWILHPAA YEGATLPTGS
      181 DVQSFLLEIV ITFILMFVIA AVSTDTRACG ELAGIAVGSA VALNALMAGS ISGASMNPAR
      241 SLGPATASGN YHSLWVYMAG PTIGALMGML TYNCIRLPNQ AMQCACNKPA KSFRR
//
LOCUS       PpNIP5-1b       295 aa
DEFINITION  PpNIP5-1b     295 aa
TITLE       PpNIP5-1b
ORIGIN

        1 MEEINIRMPP APAPLFSEDR TSTAACSKRS LDMEHNGQAP IVHAQDVNPS RFSTFTSSLR
       61 PRTSLVRKLI AEVISTFILV FMGCGAAMVN VISNGKVTPV GISLSFGLVV TIMIYAVGHV
      121 SGAHMNPAVT LAFAVAKHFS WSQVPLYIAA QCSGAFTASF LLRWILHPAA SEGATLPAGS
      181 DFQSFLIEIV ITFILMFVIA AVATDTRACG ELAGIAIGSA VALNALMAGP ISGASMNPAR
      241 SLGPAIASGN YSSIWVYLVG PIIGSVMGML AYNCIRLPDK QMQCTCDKAP KSFHR
//
LOCUS       PpNIP6-1       393 aa
DEFINITION  PpNIP6-1     393 aa
TITLE       PpNIP6-1
ORIGIN

        1 MSLRFRGYGG PLCSNGLGFI SVVHATGGNV GAGKNFELPV VEVSTILIEG FTMLAGKVNL
       61 QPHKVYSLLE VGVLTQSLSQ PVTCGGFFNM EQVVPPLMNS KPCNKCRINA ELHESSDQCV
      121 LQVPLLLEGH LELHDEWQAQ KLSTMGESYR IGGASYDRVL RMAGAELIAV FLVMFSSCGT
      181 AIANKKANGN LNLLGFATAG GLSVMMMVFA VGNISGAHLN PAVTLAFASK KMFPLQLVPI
      241 YLIAQFLGAL LAAGILQAVT GDTEVALTVP FASYAQAFVV ELILGFNLLF VATAVSTGSS
      301 NNGELSGIAI GATIIILNVLL AGPVSGASMN PMRSLGPAIV ANKYDAIWIY IIAPPVGALA
      361 GTWTHTMLQI QSSQSSHPPT QESKAHQCSS TST
//
LOCUS       PpPIP1-1       289 aa
DEFINITION  PpPIP1-1     289 aa
TITLE       PpPIP1-1
ORIGIN

        1 MNQDKDDDIA LGTNKYGDRS ALGTHAPVPE KDYTEPSVTP FFDGSEFRRW SFWRAGIAEF
       61 IATLLFLYIT IQTVMGHKRS ADPCLGVGIQ GIAWAFGGMI FALVYCTAGI SGGHINPAVT
      121 FGLFLARKVS LNRALFYMIM QCLGAMCGAE IVKGFQPNFY QEQGGGSNSV AHGYTKGDGL
      181 GAEIVGTFVL VYTVFSATDA KRNARDSHVP VLAPLPIGFA VFLVHLATIP ITGTGINPAR
      241 SLGAAVVFNK QNNAWADHWI FWIGPMLGAA LAAAYHTLVI RALPFRKRV
//
LOCUS       PpPIP1-2       289 aa
DEFINITION  PpPIP1-2     289 aa
TITLE       PpPIP1-2
ORIGIN
```

```
        1 MQQDKDDDVA LGANKYGTRS ALGTHAPVPE KDYREPSVTP FFDGGELRLW SFWRAGIAEF
       61 FATLLFLYIT IQTVMGHVRN TDPCLGVGIQ GIAWAFGGMI FALVYCTAGI SGGHINPAVT
      121 FGLFLARKVS LNRALYYMIM QCLGAMAGAG IVKGFQPDFY QAQGGGANAV NHGYTKGDGL
      181 GAEIVGTFVL VYTVFSATDA KRSARDSHVP VLAPLPIGFA VFLVHLATIP ITGTGINPAR
      241 SLGAATIYNT QHNAWADHWI FWVGPFIGAA LAAAYHTLVI RALPFRKRV
//
LOCUS       PpPIP1-3       286 aa
DEFINITION  PpPIP1-3     286 aa
TITLE       PpPIP1-3
ORIGIN

        1 MADRGDDVAV GASRHERNPL GTSAQTREKD YIEPASSPFI DPVELGRWSF WRAGIAEFFA
       61 SFLFLYITVQ TVMGHNRGDA CAGVGIQGIA WAFGGMIFTL VYCTAGISGG HINPAVTFGL
      121 FLARKVTFPR TVLYIVCQCL GAICGAGAVK GFQPDFYQSV GGGANTVAHG YTKGDGLGAE
      181 IVGTFVLVYT VFSATDAKRN ARDSHVPLLA PLPIGFAVFL VHLATIPITG TSINPARSLG
      241 AAVIWNRDQA WNDHWIFWVG PILGATLAAM YHTLVIRAIP FSANRA
//
LOCUS       PpPIP2-1       279 aa
DEFINITION  PpPIP2-1     279 aa
TITLE       PpPIP2-1
ORIGIN

        1 MAKDAGTESG VPSKDYSDPP PAPLIDAAEF GRWSFYRAII AEFVATLLFL YITISTVIGA
       61 SRNAGCAGVG LLGIAWAFGG MIFVLVYCTA GVSGGHINPA VTFGLLMARK ISLPRALTYM
      121 IAQCLGAICG AGLAKGFQTA FYMRYGGGAN SVALGYSTGT GLAAEIIGTF VLVYTVFSAT
      181 DPKRNARDSH VPVLAPLPIG FAVFMVHLAT IPITGTGINP ARSFGAAVIY NRSKPWDDHW
      241 IFWVGPFLGA ALAASYHQYI LRAAPFKSLG SFRSAPSHV
//
LOCUS       PpPIP2-2       279 aa
DEFINITION  PpPIP2-2     279 aa
TITLE       PpPIP2-2
ORIGIN

        1 MAKDVGVEPG FPSKDYTDPP PAPLIDASEF GQWSFYRAVI AEFVATLLFL YITIATVIGA
       61 VRNAGCDGVG LLGIAWAFGG MIFVLVYCTA GISGGHINPA VTFGLLLARK ISLPRALAYM
      121 IAQCLGAICG AGLVKGFQTA FYMRYGGGAN SVAAGYSIGT GLAAEIIGTF VLVYTVFSAT
      181 DPKRNARDSH VPVLAPLPIG FAVFMVHLAT IPITGTGINP ARSFGAAVIY NRSKPWNDHW
      241 IYWVGPFLGA ALAAAYHQYV LRAGPFKSLG SFRSAPSHI
//
LOCUS       PpPIP2-3       280 aa
DEFINITION  PpPIP2-3     280 aa
TITLE       PpPIP2-3
ORIGIN

        1 MSKVPVGVEP GFPGKDYADP PAAPLIDASE FGQWSFYRAI IAEFVATLLF LYITIATVIG
       61 ASRNAGCAGV GTLGIAWAFG GMIFVLVYCT AGISGGHINP AVTFGLLLAR KISLPRALAY
      121 MIAQCLGAIC GAGLVKGFQQ SFYMTYGGGA NAVNAGYGIG TGLAAEIIGT FVLVYTVFSA
      181 TDPKRNARDS HVPVLAPLPI GFAVFMVHLA TIPITGTGIN PARSFGAAVI YNRSKPWDDH
      241 WIFWVGPFVG AALAAAYHQY VLRAGPFKQL GSFRSAPSRV
//
LOCUS       PpPIP2-4       280 aa
DEFINITION  PpPIP2-4     280 aa
TITLE       PpPIP2-4
ORIGIN
```

```
        1 MEKIGICEEP KFRSKDYIDP PAVPFVDASE LRKWSFYRAI ITEFISTLLF LYIAIGTVVG
       61 ASRNADCAGV GILGIAWAFG GMIFVLVYCT AGISGGHINP AVTFGLLLAR KISLTRSLAY
      121 MVAQCLGAIC GAGLVKEFQH SFYMDHGGGA NAVAPGYSTG TGLAAEIIGT FVLMFTVFSA
      181 TDPKRKARDS HVPVLAPLPI GFAVFVVHCA TIPITGTGIN PARSFGAAVI FNRSKSWDDH
      241 WIFWVGPFLG AALAAAYHQY ILRANPIKSM RSFGNGSNHT
//
LOCUS       PpPIP2-4       280 aa
DEFINITION  PpPIP2-4     280 aa
TITLE       PpPIP2-4
ORIGIN

        1 MEKIGICEEP KFRSKDYIDP PAVPFVDASE LRKWSFYRAI ITEFISTLLF LYIAIGTVVG
       61 ASRNADCAGV GILGIAWAFG GMIFVLVYCT AGISGGHINP AVTFGLLLAR KISLTRSLAY
      121 MVAQCLGAIC GAGLVKEFQH SFYMDHGGGA NAVAPGYSTG TGLAAEIIGT FVLMFTVFSA
      181 TDPKRKARDS HVPVLAPLPI GFAVFVVHCA TIPITGTGIN PARSFGAAVI FNRSKSWDDH
      241 WIFWVGPFLG AALAAAYHQY ILRANPIKSM RSFGNGSNHT
//
LOCUS       PpPIP3-1       257 aa
DEFINITION  PpPIP3-1     257 aa
TITLE       PpPIP3-1
ORIGIN

        1 MPTDNEFRDT HEPPPAPILA RDEFNEWSFY RAIIAEFIAT LLFLYVSLTT LMGTTRIFGG
       61 SVGLIETAWA FGGMIFILVY CTAGISGGHI NPAVTFGLFL AQQVTLPRAS AYIVAQCLGA
      121 IVGAAIARGV QEGGEYRSFA SNAVNGVQPG YNIGQALAAE IMGTFVLLYT VLSATDPTRK
      181 ARDSHVPVLA PLPIGFAIFV VHLATIPITG TGINPARSLG AAVWIFWVGP IVGSTCAAIY
      241 YTYVLKAASL RFRSLYE
//
LOCUS       PpSIP1-1       277 aa
DEFINITION  PpSIP1-1     277 aa
TITLE       PpSIP1-1
ORIGIN

        1 MGLARKAVAD ASITFLWVFA MASLGAVSTS IAPSLGLDGP GKGKMYIVFS LVSFLIFFFS
       61 FLGQALGGAS WNPTTIVAFS FAGVSNDDLF TLGVRLPAQM VGAVGGALTI WEVMPKKYKH
      121 TLGGPKLKVP LETGVAAETI LTFTITLIVM WAILRGPRNK ISRTFIIIGA TIALVIAGGA
      181 YTGPAMNPAN AFGWAFVSNK HTSWEHFAVY WAGPMIGTIC AVLTFNLIFG RHQVKGQATK
      241 KSKAKKTKKP GSEGQAAKSK GLKKESTGNA GDKMKAS
//
LOCUS       PpSIP1-2       279 aa
DEFINITION  PpSIP1-2     279 aa
TITLE       PpSIP1-2
ORIGIN

        1 MGLARKAVAD ASITFLWVFA MASLGAASTA IASSLGLDGP GKTKMYIVFA LVSFLVFFFS
       61 FLGHALGGAS WNPTAIVAFS YAGVSNDDLF TLGVRLPAQM VGAVGGALAI LEVMPKKYKH
      121 MLGGPKLKVP LQTGVIAEAI LTFTITLIVM WAMLRGPRNK MAKTFIIIGA TIALVTAGGA
      181 YTGPAMNPAN AFGWAFVSNQ HTSWDHFAVY WAGPMIGTIF AVWAFNLLFG PHSQATQASD
      241 SKKLKANKAK KSGSEGESAK DKKRGEGLSE NAAGKVKAS
//
LOCUS       PpTIP6-1       250 aa
DEFINITION  PpTIP6-1     250 aa
TITLE       PpTIP6-1
```

```
ORIGIN

        1 MKIAFGEADE ASSPDAIKGA VAEFISLFLF VFIGVGSVMA YEKIHVGDLD AAGLLMIAIA
       61 HGLAIAVLVA ATANISGGHV NPAVSLGLAL AGKITIIRLV LYWVAQLLGA VAGAWVLKAV
      121 TTGEDVARHA IGANMTGFSA MLMEIVLTFT LMFVVFATAV DPNKGTVGVI APLAIGFTVL
      181 AQIFVGAPFS GASMNPGRSF GPAVVAWDFK NHWVYWVGPL VGAALAALIY DGVFISPAPP
      241 AGHQPVPTEF
//
LOCUS       PpTIP6-2       250 aa
DEFINITION  PpTIP6-2     250 aa
TITLE       PpTIP6-2
ORIGIN

        1 MKVAFGEADE VSSPDALKGA LAEFISLFLF VFIGVGSVMS YEKIHVGDLE AGGLLIIAIA
       61 HGLAIAILVA ATANISGGHV NPAVSLGLAL AGKITIIRLV LYWIAQLLGA VAGAWVLKIV
      121 TTGEDLARHA IGAGMTTWSA TLMEIVLTFT LVFVVFATAV DPKKGTVGVI APLAIGFTVL
      181 AQIFVGAPFS GASMNPGRSF GPAVVAWDFT NHWVYWVGPF IGAALAALIY DGVFMSPAAP
      241 EGHQPVPTEF
//
LOCUS       PpTIP6-3       251 aa
DEFINITION  PpTIP6-3     251 aa
TITLE       PpTIP6-3
ORIGIN

        1 MVKLAFGESD EASSPDALKG ALAEFISLFL FVFIGVGSVM SYEKIHVGDL EAGGLLMIAI
       61 AHGLAIAILV AATANISGGH VNPAVSLGLA LAGKITVIRL VLYWVAQLLG AVAGAWVLKM
      121 VTTGEDVARH AIGVGMSPMS AVLMEIVLTF TLVFVVFATA VDPKKGTVGV IAPLAIGFTV
      181 LAQIFVGAPF SGASMNPGRS FGPAVIAMDF TNHWVYWVGP FIGAALAAVI YDGVFISPSP
      241 PAGHQAIPSD F
//
LOCUS       PpTIP6-4       251 aa
DEFINITION  PpTIP6-4     251 aa
TITLE       PpTIP6-4
ORIGIN

        1 MVKVAFGEAN EASSADALKG AFAEFLALFL FVFIGVGSVM SYEKIHAGDM DAAGLLVIAI
       61 AHGLAIAVLV SATANISGGH INPAVSLGLA LAGKITVIRL VLYWIAQLLG AAAGAWVLKI
      121 VTTGEDVARH AIGVGMTPWS AVLMEAVLTF TLVFVVFATA VDPKKGTVGV IAPLAIGFTV
      181 LAQIFVGAPF SGASMNPGRS FGPALVAMDF TNHWVYWVGP FIGAALAALI YDGVFISPSP
      241 PPGHHAIPSD F
//
LOCUS       PpXIP1-1       371 aa
DEFINITION  PpXIP1-1     371 aa
TITLE       PpXIP1-1
ORIGIN

        1 MGHSQAADVV VYQHTASAPG TPTLDEDRGT CKIPEPISAI SASKFSSEVL HRAVLRDLQN
       61 PEVWRAGVFE CVASFAATFV GILCTISTLE AEFSHPVAVI ACLQGLVLSL CIFAAAPATG
      121 GHVNPCITWT EMLTGHISPV RGVLYIIGQI LGSIVGSFMA KIVVGNALAT QYNLGGCYLQ
      181 SRVSATSGMM GLGTGRALVL EIVLAFFVLF ISYSVALDPP RLPRTGYTLA PFMIGGIVGL
      241 CIFAGAGLFS GYGGAGINPG RCIGPAVVLG GSMWTGHWVF WVGPGLSGAL MAALYRNIPP
      301 THIQVYKLRK EARKGLVGGR KNKPFFAKVN NLRLACGNEK DGERIRSDSS EDQSSSHHRG
      361 KPLTYGRDHA V
//
```

```
LOCUS       PpXIP1-2       466 aa
DEFINITION  PpXIP1-2     466 aa
TITLE       PpXIP1-2
ORIGIN

        1 MASSLGGVSA DACSGRVSYH RTGSSTRSTA VPVEHLRTIE SGIQSDWENF PQIQPSRVVR
       61 NDSVKGHYDG SVVNVPLTRK VKVWIGLHDS RKADVWRAAA VEFVATAGLT FLSIGAYQQG
      121 KSISVAAHVF IQALIYSLVI LAATPISGAH LNPSITFTTF LTGQATLVRT ILYVVAQLLG
      181 GILGALGMWA LTTHEMRREY SLGGCLLQKL PVEGTDLGLS TLSNKQGLVA ETVFTIIMLF
      241 VVYGIGFDSR NVVVTFLISS PFIIGGIFGI LIFISQGVGY TTAMNPARCF GPAILHHNKL
      301 WGPLYIFIFG PLIAAGIVAI FQHIMHQKHA AEVEPVLPLN FFHIVTPDHQ RPGFGPRCPT
      361 MFYPSIDQEV DHSQQLVSGN IPTMMSSELL QQPSNQLNSA ANKINLVLQV KAFMQPRTGE
      421 TRQASKTNIM DGARIDQNLL LQQYSNSLGS SDGIAIHHSG DFEKDR
//
LOCUS       PtNIP1-1       276 aa
DEFINITION  PtNIP1-1     276 aa
TITLE       PtNIP1-1
ORIGIN

        1 MAEIDGTNGN GNHGGVVLDI KDNYPSSSSI KEVSVLNFYV PFMQKLVAEI AGTYFLIFAG
       61 CSSVAVNLNF DKVVTLPGIS ITWGLAVMVL VYSVGHISGA HFNPAVTLAF ATCKRFPWKQ
      121 VPAYVACQVI GATLAAGTIR LLFQGDQDHF TGTMPAGSNL QSFVVEFIIT FYLMFIISGV
      181 ATDNRAIGEL AGLAVGSTVL LNVMFAGPIS GASMNPARSL GPAIVSHQYK GLWIYIVSPI
      241 LGAQAGAWVY NLIRYTDKPL REITKSASFL NGKESS
//
LOCUS       PtNIP1-2       278 aa
DEFINITION  PtNIP1-2     278 aa
TITLE       PtNIP1-2
ORIGIN

        1 MADIDGTGSN GNHGGVVLDI KDDHPPTSSN LTKEDSDLYF SVPFMQKLVA EIVGTYFLIF
       61 AGCSSVAVNL NFEKVVTLPG ISIVWGLAVM VLVYSLGHIS GAHFNPAVTL AFATCKRFPW
      121 KQVPAYISCQ VIGSTLAAGT IRLIFQGKQD HFTGTMPAGS DLQSFVVEFI ITFYLMFIIS
      181 GVATDNRAIG ELAGLAVGST VLLNVMFAGP ISGASMNPAR SLGPAMVSHE YRGIWIYVVS
      241 PILGAQAGAW VYNLIRYTDK PLREITKSAS FLQSKGRF
//
LOCUS       PtNIP1-3       263 aa
DEFINITION  PtNIP1-3     263 aa
TITLE       PtNIP1-3
ORIGIN

        1 MPWNNEFGDD TEGGKKTESS DEDSPPETTV QIIQKIIAEM IGTFFLIFMG CGSVVVNQMY
       61 GSVTFPGVCV VWGLIVMVMV YSVGHISGAH FNPAVTVTFA IFRHFPYKQV PLYIAAQLLG
      121 SLLASGTLSL LFSVTDEAYF GTIPVGPDIR SFVTEIIISF LLMFVISGVA TDNRAIGELA
      181 GIAVGMTIML NVFVAGPVSG ASMNPARSLG PAIVMRQFKG IWVYIVGPPI GTILGALCYN
      241 IIRFTDKPLR EITKTASFLK SKN
//
LOCUS       PtNIP1-4       279 aa
DEFINITION  PtNIP1-4     279 aa
TITLE       PtNIP1-4
ORIGIN

        1 MARKSDGIES QEITSMEEGL ATPTDPKENG KFDCCTSPAA VTITQKLIAE VIGTYFVIFA
       61 GCGSVAVNNI YGSVTFPGVC VTWGLIVMVM IYSLGHISGA HFNPAVTIAF AIFRRFPSWQ
```

```
      121 VPLYIIAQLM GSILASGTLA LALDVTPEAF FGTVPVGSDG QSLVLEIIIS FLLMFVISGV
      181 STDDRAVGDL AGIAVGMTIL LNVFVAGPVS GASMNPARSI GPAVVKHQFK GLWVYIVGPI
      241 IGAIAGAFAC NLIRWTDKPL GELTKVGSFI KSGSKNYAS
//
LOCUS       PtNIP1-5       282 aa
DEFINITION  PtNIP1-5     282 aa
TITLE       PtNIP1-5
ORIGIN

        1 MSSSNSITEP SPKFQLPTRR SIMAEAKAAS PAPEWLSTRN AALSNFQKIV AELMGTYILV
       61 FVGCGAALTD KVQRLNMLGI AIVWGAVLMA AIYALGHVSG AHFNPAVSIA LAVVRKFSWK
      121 EVPMYILAQV LGSTLASLTL RMLFHEQGNI QPIVNQYSDP TSDLEAIVWE FIITFILMFT
      181 ICGVATDPRA SKDLSGVAIG GAVMFNAMIA GPITGASMNP ARSLGPALVS GVYKNLWVYI
      241 VSPILGAMAA AAVYSVLRVP EPAKPEDTNK STYNNLNLHA DP
//
LOCUS       PtNIP2-1       278 aa
DEFINITION  PtNIP2-1     278 aa
TITLE       PtNIP2-1
ORIGIN

        1 MATVDQEMNI SVESSRFHFV KLFREHYPSG FLRKVVAEVI ATYLLVFVTC GAAAISASDE
       61 HKVSKLGASV AGGLIVTVMI YAVGHISGAH MNPAVTTAFA AVLNFPWKQV PFYAAAQLTG
      121 AISASFTLKV LLHPIRNVGT TSPSGTAVQA LIMEIVVTFS MMFITSAVAT DTKAVGELAG
      181 IAVGSAVCIT SILAGPVSGG SMNPARTLGP AIASRYFKGV WVYLLGPVTG TLLGAWSYNL
      241 IRVTDKPVQA IPRRFSFGSR RTRAIDEQSP SMGPLDAF
//
LOCUS       PtNIP3-1       303 aa
DEFINITION  PtNIP3-1     303 aa
TITLE       PtNIP3-1
ORIGIN

        1 MDNAEVPSVP STPATPGTPG APLFGGFKGE RGVHGRKSLL RSCKCFSVEE WAMEEGRLPP
       61 VSCSLPPPPV SLARKVGAEF IGTLILIFAG TATAIVNQKT QGSETLVGLA ASSGLAVMIV
      121 ILATGHISGA HLNPSITIAF AALKHFPWKH VPVYIGAQVL ASLCAAFALK GIFHPVMGGG
      181 VTVPSGGYGQ AFALEFITSF ILMFVVTAVA TDTRAVGELA GIAVGATVML NIFIAGETTG
      241 ASMNPVRTLG PAIAVNNYKA IWIYLTAPIL GALCGAGTYS AVKLPEEDGD SNEKTSAARS
      301 FRR
//
LOCUS       PtNIP3-2       303 aa
DEFINITION  PtNIP3-2     303 aa
TITLE       PtNIP3-2
ORIGIN

        1 MDTEEVPSAP STPATPGTPG APLFGGFKGE RGVHGRKSLL RSCKCFGVEE WAMEEGRLPP
       61 VSCSLPPPPV SLARKLGAEF MGTLILIFAG TATAIVNQKT QGSEALIGLA ASTGLAAMIV
      121 ILSTGHISGA HLNPSITIAF AALKHFPWKH VPVYIGAQVL ASLCAAFALK VIFHPMMGGG
      181 VTVPSGGHGQ AFALEFIISF ILMFVVTAVA TDTRAVGELA GIAVGATVML NILIAGETTG
      241 ASMNPVRTLG PAIAANNYKA IWVYLTAPIL GALCGAGTYS AVKLPEEDGD TNEKTSATRS
      301 FRR
//
LOCUS       PtNIP3-3       299 aa
DEFINITION  PtNIP3-3     299 aa
TITLE       PtNIP3-3
ORIGIN
```

```
        1 MPESEAGTPA VSAPNTPGTP GGPLFTGLRV DSLSYSDRKI MPKCKCLPVT APTWGQPHTC
       61 FLDFPAPDVS LTRKLGAEFV GTFILIFAAT AGPIVNQKYN NAETLIGNAA CAGLAVMIII
      121 LSTGHISGAH LNPSLTIAFA ALRHFPWVQV PAYIAAQVSA SICASFALKG VFHPFMSGGV
      181 TVPSVSTGQA FALEFLITFN LLFVVTAVAT DTRAVGELAG IAVGATVMLN ILVAGPSSGG
      241 SMNPVRSLGP AVAAGTYKDI WIYLVAPTLG ALVGAATYTA VKLREEEADP PRQVRSFRR
//
LOCUS       PtNIP3-4       300 aa
DEFINITION  PtNIP3-4     300 aa
TITLE       PtNIP3-4
ORIGIN

        1 MPGPEEAGTP TVTAPNTPGT PGGPLFTGLR VDSLSYSDRK IMPKCKCLPV TAPNWGQPHT
       61 CFLDIPSPDV SLTRKLGAEF VGTFILIFMA TAGPIVNQKY DHAETLIGNA ACAGLAVMII
      121 ILSTGHISGA HLNPSLTIAF AALRHFPWVQ VPAYIAAQVS ASICASFALK GVFHPFMSGG
      181 VTVPSVSTGQ AFALEFFITF NLLFVVTAVA TDTRAVGELA GIAVGATVML NILVAGPSTG
      241 GSMNPVRTLG PAIAAGNYKK IWIYLVAPTL GAVVGAGAYT LVKLRDDETD PPRPVRSFRR
//
LOCUS       PtNIP3-5       300 aa
DEFINITION  PtNIP3-5     300 aa
TITLE       PtNIP3-5
ORIGIN

        1 MKHLLEEITS AHVPKTAVLP PASSSSSSTD DQEMDSNSMP MKRHIFIKKS SFCSFLHGMD
       61 LNPARMVLAE MVGTFLLLFC VCGIVACTQI LRGEVGLMEY ASVAGLTIIV VIFSIGSISG
      121 AHVNPAVTIA FATFGHFPWS KVPLYILAQT VGSVSATYVG SSVYGVKTEL MTTRPAIGCS
      181 SAFWVEFMAT FMLMFLAASL TSQSRSIGPL SGFLYGIAIG LAVLITGPVS GGSLNPARSL
      241 GPAIVSWDFK DIWVYITAPT IGAVAGALMF HLLRIRPQAC SANSSPDDDL LVHSIAFTES
//
LOCUS       PtPIP1-1       287 aa
DEFINITION  PtPIP1-1     287 aa
TITLE       PtPIP1-1
ORIGIN

        1 MEGKEEDVRL GANKFNERQP LGTAAQSQDD KDYKEPPPAP LFEPSELTSW SFYRAGIAEF
       61 MATFLFLYIT VLTVMGVFKD TTKCTTVGIQ GIAWAFGGMI FALVYCTAGI SGGHINPAVT
      121 FGLFLARKLS LTRAVFYMLM QCLGAICGAG VVKGFYGKKN YELLNGGANM VSPGYTKGDG
      181 LGAEIVGTFV LVYTVFSATD AKRSARDSHV PILAPLPIGF AVFLVHLATI PITGTGINPA
      241 RSLGAAIIFN KDKAWDDHWI FWVGPFIGAA LAALYHQVVI RAIPFKK
//
LOCUS       PtPIP1-2       287 aa
DEFINITION  PtPIP1-2     287 aa
TITLE       PtPIP1-2
ORIGIN

        1 MEGKEEDVRL GANRFNERQP IGTAAQSLDD KDYKEPPPAP LFEPGELTSW SFYRAGIAEF
       61 MATFLFLYIT VLTVMGVVKD QTKCTTVGIQ GIAWAFGGMI FALVYCTAGI SGGHINPAVT
      121 FGLFLARKLS LTRAVFYMVM QCLGAICGAG VVKGFYGKTN YELHNGGANM VAHGYTKGDG
      181 LGAEIVGTFI LVYTVFSATD AKRSARDSHV PILAPLPIGF AVFLVHLATI PITGTGINPA
      241 RSLGAAIIFN KDSAWDDHWI FWVGPFIGAA LAALYHQVVI RAIPFKK
//
LOCUS       PtPIP1-3       356 aa
DEFINITION  PtPIP1-3     356 aa
TITLE       PtPIP1-3
```

```
ORIGIN

        1 MIPPPPPPDDN TFFLSGQIIE KLLSSIYKTT LSLFLSLNFC FVCLSVLSYF QREHREKNKP
       61 TTHLTRKKME GKEEDVKLGA NKFSERQPIG TSAQTDKDYK EAPPAPLFEP GELKSWSFYR
      121 AGIAEFIATF LFLYITVLTV MGVTKPGTSK CSTVGIQGIA WAFGGMIFAL VYCTAGISGG
      181 HINPAVTFGL FLARKLSLTR AVFYIIMQCL GAICGAGVVK GLQGSHNYEL QGGGANVVNH
      241 GYTKGDGLGA EIVGTFVLVY TVFSATDAKR NARDSHVPIL APLPIGFAVF LVHLATIPIT
      301 GTGINPARSL GAAIIFNKDH AWDDHWIFWV GPFIGAALAA VYHQIVIRAI PFKSRA
//
LOCUS       PtPIP1-4       287 aa
DEFINITION  PtPIP1-4     287 aa
TITLE       PtPIP1-4
ORIGIN

        1 MEEGEEDVKV GANRYGEGQP IGTAAQTQHG KDYTEPPPAP LYQPGEWLSW SFYRAGIAEF
       61 VATFLFLYIT VLTVMGVARS STKCSTVGIQ GIAWAFGGMI FVLVYCTAGI SGGHINPAVT
      121 FGLLLARKLT LTRAVFYMIM QCLGAICGAG VVKGFQKSPY EILGGGANTV STGYSKGSGL
      181 GVEILGTFVL VYTVFSATDA KRSARDSHVP VLAPLPIGFA VFLVHLATIP ITGTGINPAR
      241 SLGAALIYNK DKAWDDHWIF WVGPFIGAAL ASLYHQIVIR AIPFKSK
//
LOCUS       PtPIP1-5       287 aa
DEFINITION  PtPIP1-5     287 aa
TITLE       PtPIP1-5
ORIGIN

        1 MEGREEDVRV GANKYGERQP IGTAAQAQDV KDYTDPPPAP LFEPGELSSW SFYRAGIAEF
       61 VATFLFLYIT VLTVMGVAKS PTKCSTVGIQ GIAWAFGGMI FALVYCTAGI SGGHINPAVT
      121 FGLLLARKLS LTRAVFYMLM QCLGAICGAA VVKAFQKSQY EMLGGGANTV STGYAKGSGL
      181 GAEIVGTFVL VYTVFSATDA KRNARDSHVP ILAPLPIGFA VFLVHLATIP ITGTGINPAR
      241 SLGAALIYNK DQAWDDHWIF WVGPFIGAAL ASLYHQIVIR AIPFKSK
//
LOCUS       PtPIP2-1       279 aa
DEFINITION  PtPIP2-1     279 aa
TITLE       PtPIP2-1
ORIGIN

        1 MSKDVIEEGQ THTKDYVDPP PAPLFDVGEL KLWSFFRALI AEFIATLLFL YVTVATVIGH
       61 KKNQDACGGV GLLGIAWAFG GMIFILVYCT AGISGGHINP AVTFGLLLAR KVSLIRAVGY
      121 MVAQCLGAVC GVGLVKAFMK PYYNSLGGGA NMVAPGYSTG TAVGAEIIGT FVLVYTVFSA
      181 TDPKRSARDS HIPVLAPLPI GFAVFMVHLA TIPITGTGIN PARSFGAAVI INDKKAWDDH
      241 WIFWVGPFVG ALAAAAYHQY ILRAGAIKAL GSFRSHPTN
//
LOCUS       PtPIP2-2       279 aa
DEFINITION  PtPIP2-2     279 aa
TITLE       PtPIP2-2
ORIGIN

        1 MSKEVSEVGQ THGKDYVDPP PAPLLDLGEL KLWSFYRALI AEFIATLLFL YVTVATVIGH
       61 KSNKDPCDGV GLLGIAWAFG GMIFILVYCT AGISGGHINP AVTFGLFLAR KVSLIRAVAY
      121 MVAQCLGAIC GVGLVKAFMK KNYNSLGGGA NTVAMGYNTG TALGAEIIGT FVLVYTVFSA
      181 TDPKRSARDS HVPVLAPLPI GFAVFMVHLA TIPITGTGIN PARSFGAAVI FNNEKAWDDH
      241 WIFWVGPFVG ALAAAAYHQY ILRAAAIKAL GSFRSNPAN
//
LOCUS       PtPIP2-3       285 aa
```

```
DEFINITION  PtPIP2-3     285 aa
TITLE       PtPIP2-3
ORIGIN

        1 MAKDMEVAEA GSFSAKDYHD PPPAPLFDAK ELTKWSFYRA LIAEFIATLL FLYITVLTVI
       61 GYKSQIDGSA DSCGGVGILG IAWAFGGMIF VLVYCTAGIS GGHINPAVTF GLFLARKVSL
      121 IRAVMYMVAQ CLGAICGVGL VKAFQKSYYK KYGGGANTLA DGFSTGTGLG AEIIGTFVLV
      181 YTVFSATDPK RSARDSHVPV LAPLPIGFAV FMVHLATIPI TGTGINPARS LGAAVIYNQD
      241 KAWDGHWIFW VGPFAGAAIA AFYHQFILRA GAVKALGSFR SAQRF
//
LOCUS       PtPIP2-4       285 aa
DEFINITION  PtPIP2-4     285 aa
TITLE       PtPIP2-4
ORIGIN

        1 MAKDTEVAEA GSFSAKDYQD PPPAPLIDAE ELTKWSFYRA LIAEFIATML FLYITVLTVI
       61 GYKSQIDGNA DPCGGVGILG IAWAFGGMIF VLVYCTAGIS GGHINPAVTF GLFLARKVSL
      121 IRAVMYMVAQ CAGAICGVGL VKAFQKSYYT KYNGGANVLA DGYSTGTGLG AEIIGTFVLV
      181 YTVFSATDPK RSARDSHVPV LAPLPIGFAV FMVHLATIPI TGTGINPARS FGAAVIYNNK
      241 KAWHDQWIFW AGPFIGAAIA AFYHQFILRA GAIKALGSFR SNPNV
//
LOCUS       PtPIP2-5       285 aa
DEFINITION  PtPIP2-5     285 aa
TITLE       PtPIP2-5
ORIGIN

        1 MGKDIEVGGE FSAKDYHDPP PAPLIDAEEL TQWSLYRAII AEFIATLLFL YITVLTVIGY
       61 KSQTDTTKNS DACGGVGILG IAWAFGGMIF VLVYCTAGIS GGHINPAVTF GLFLARKVSL
      121 VRAVLYMVAQ CLGAICGCGL VKAFQKSYYT KYGGGVNELA TGFSKGTGLG AEIIGTFVLV
      181 YTVFSATDPK RNARDSHVPV LAPLPIGFAV FMVHLATIPI TGTGINPARS FGAAVIYNED
      241 KAWDDHWIFW VGPFIGAAIA ALYHQYVLRA AAVKALGSFR SSSNI
//
LOCUS       PtPIP2-6       285 aa
DEFINITION  PtPIP2-6     285 aa
TITLE       PtPIP2-6
ORIGIN

        1 MGKDVEVRGE FIAKDYHDPP PAPLIDAEEL TQWSLYRAII AEFIATLLFL YITVLTVIGY
       61 KSQTDTTKNS DACGGVGILG IAWAFGGMIF VLVYCTAGIS GGHINPAVTF GLFLARKVSL
      121 VRAVLYMVAQ CLGAICGCGL VKAFQKSYYT KYGGGANELA TGFSKGTGLG AEIIGTFVLV
      181 YTVFSATDPK RNARDSHVPV LAPLPIGFAV FMVHLATIPI TGTGINPARS FGAAVIYNKD
      241 KAWDDHWIFW VGPFIGAAIA ALYHQYVLRA AAVKALGSFR SSSNI
//
LOCUS       PtPIP2-7       285 aa
DEFINITION  PtPIP2-7     285 aa
TITLE       PtPIP2-7
ORIGIN

        1 MGKDIEVGGE FSAKDYHDPP PAPLIDAEEI TQWSFYRAII AEFVATLLFL YITVLTVIGY
       61 KSQTDVNKNG DECGGVGILG IAWAFGGMIF ILVYCTAGIS GGHINPAVTF GLFLARKVSL
      121 VRAILYMVAQ CLGAICGCGL VKAFQKSYYT NYGGGANGLA NGYSKGTGLG AEIIGTFVLV
      181 YTVFSATDPK RNARDSHVPV LAPLPIGFAV FMVHLATIPI TGTGINPARS FGAAVIFNKE
      241 KAWDDHWIFW VGPFIGAAIA ALYHQFILRA AAVKSLGSFR SSPNI
//
```

```
LOCUS       PtPIP2-8       284 aa
DEFINITION  PtPIP2-8     284 aa
TITLE       PtPIP2-8
ORIGIN

        1 MAKDIEVAEH GETVKDYQDP PPAPLIDAEE LGQWSFYRAL IAEFIATLLF LYVTVLTVIG
       61 YKSQTDPDKG LDACGGVGIL GIAWAFGGMI FVLVYCTAGI SGGHINPAVT FGLFLARKVS
      121 LIRAVLYMVA QCLGAICGCG LVKAFQKSYY NHYGGGANEL QEGYNKGTGL GAEIIGTFVL
      181 VYTVFSATDP KRNARDSHVP VLAPLPIGFA VFMVHLATIP ITGTGINPAR SFGAAVIFNQ
      241 SKAWDDHWLF WVGPFIGAAI AAFYHQFILR AAAIKALGSF RSNA
//
LOCUS       PtPIP2-9       254 aa
DEFINITION  PtPIP2-9     254 aa
TITLE       PtPIP2-9
ORIGIN

        1 MEELKQWSFY RALIAEFVAT FLFLYIGVGT VVGYKGVHNN LCDGAGYLGV AWAFGGMIFV
       61 LVYCTAGISG GHINPAVTFG LFVARKVSLI RAVAYMMAQC LGAMLGVWMV MILTGIHYDQ
      121 AGGAVNVVAP GYSKGTALGA EIIGTFVLVY TVLAATDPKR MARDSHVPVL APLPIGFAVF
      181 VVHLALIPIT GTGINPARSL GAAVVKNAKE IWDDHWIFWV GPFVGALAAA VYHQYILGSG
      241 AAKALASFRS NPTS
//
LOCUS       PtPIP2-10       289 aa
DEFINITION  PtPIP2-10     289 aa
TITLE       PtPIP2-10
ORIGIN

        1 MSSEERNIER QHGRDYHDPP PAPLLDMGEL KQWSFYRAAI AEFIATFLFL FFSVSTVVNY
       61 KEPNYTDQCS RVGHLGIAWA NGGMIFVLVY CTSGISGGHL NPAVTFGMLV ARKMSLIRAA
      121 AYMLAQCLGA ILGHLFVFLF MYADEQQSSV GVVNVVSRNY SKGAGLGAEF IGTFVLVYTV
      181 FSATDPKRNA RDSHVPVLAP LPIGFAVFVV HLATIPITGT GINPARSLAT NLLHRSTAEA
      241 MDDLWIFWVG PFLGALAAAV YHKYVLRAGA VKTLKSFRAL GSFGSQPPV
//
LOCUS       PtSIP1-1       239 aa
DEFINITION  PtSIP1-1     239 aa
TITLE       PtSIP1-1
ORIGIN

        1 MGAVKAAIGD AVFTFMWVFV SSMFGLFTNV IVTALGLQTL VWAPVLANAS LIFAFVFLFN
       61 FLGEFLGGAT FNPTGTASFY AAGVGGDSLF SMALRFPAQA AGSVGGSLAI LEVMPLQYKH
      121 MLGGPTLQVD LQTGGLAEGV LTFLMTFAVL VIILKGPRSS LVQAWFLATV TVTLVSAGST
      181 YTGPSMNPAF AFGWAYVNKW HNTWEQLYVY WICPFIGAIL AAWVFRVVFP PPAPKQKKT
//
LOCUS       PtSIP1-2       239 aa
DEFINITION  PtSIP1-2     239 aa
TITLE       PtSIP1-2
ORIGIN

        1 MGAIKAASGD AVLTFMWVFV SSMFGLFTNL IVTALGLQTL VWAPLVITTF IVFTFVFLFN
       61 LIGEALGGAS FNPTGTASFY AAGVGGDTLF SMALRFPAQA AGAVGGALAI MEVMPVQYKH
      121 MLGGPTLQVD LHTGGLAEGV LTFLMSFAVL VIILKGPRNP LVQTLFLAIA TITLVVAGST
      181 YTGPSMNPAN AFGWAYVRKW HNTWEQLYVY WICPFIGAIL ASWVFRAVFP PPAPKQKKA
//
LOCUS       PtSIP1-4       241 aa
```

```
DEFINITION  PtSIP1-4     241 aa
TITLE       PtSIP1-4
ORIGIN

        1 MGAIKGAIVD GILTCMWVFS VPLLGVFSSI IATYVGVEAM SIAGLFITIN VAALFMLTFS
       61 LIGAACGGAS FNPATTITLY TAGLKPDASL MSMALRFPVQ AAGGVAGAMA ITEVMPKQYR
      121 YVLRGGPSLK VDLHTGAIAE GVLTFLICLA LHFVLLKGPK NFVLKVWLLA VATVGLVMAG
      181 GKYTGPSMNP ANAYGWAYLS NRHTTWDFFY VYWICPFIGA TLAALISKFL FKAPPIKDKK
      241 A
//
LOCUS       PtSIP1-3a       241 aa
DEFINITION  PtSIP1-3a     241 aa
TITLE       PtSIP1-3a
ORIGIN

        1 MGAIKGAIVD GILTAMWVFS VPLLGVFSSI IATYVGVEAM SIAGLFISIN VAALFMLTFS
       61 LIGAAFGGAS FNPATTITLY IAGLKPDASL LSMALRFPVQ AAGGVGGAMA IRGVMPKHYR
      121 HVLKGGPSLR VDLHTGAIAE GVLTFLICLT LHFLLLKGPK NVVLKVWFLA VATVGLVMAG
      181 GKYTGPSMNP ANAYGWAYLG NRHTTWDFFY VYWICPFIGA ILAAFVSKFL FKAAPIKEKK
      241 A
//
LOCUS       PtSIP1-3b       230 aa
DEFINITION  PtSIP1-3b     230 aa
TITLE       PtSIP1-3b
ORIGIN

        1 MGAIKGAIVD GILTAMWVFS VPLLGVFSSI IATYVGVEAM SIAGLFISIN VAALFMLTFS
       61 LIGAAFGGAS FNPATTITLY IAGLKPDASL LSMALRFPVQ AAGGVGGAMA IRGVMPKHYR
      121 HVLKGGPSLR VDLHTGAIAE GVLTFLICLT LHFLLLKGPK NVVLKVWLLA VATVGLVMAG
      181 GKYTGPSMNP ANAYGWAYLG NRHTTWDFFY VYWICPFIGA ILAAFVSKFL
//
LOCUS       PtSIP2-1       238 aa
DEFINITION  PtSIP2-1     238 aa
TITLE       PtSIP2-1
ORIGIN

        1 MVSKTRLILS DFVVSLMWVW SGSLIKIFVF KVLGMGHDSR GEFLKNSLSI MNMFLFAFLG
       61 KFTKGGAYNP LTILSSAISG DFSQFLFTIG ARIPAQVIGS ITGVRLFIDT FPEIGLGPRL
      121 TVDIHKGALT EGLLTFAIVT ISLGLARKIP GSFFMKTWIS SVSKLSLHIL GSDLTGGCMN
      181 PASVMGWAYA RGDHITKEHI LVYWLAPIEG TLLAVWTFKL LFRPQKQDEK EKLKGKTE
//
LOCUS       PtSIP2-2       238 aa
DEFINITION  PtSIP2-2     238 aa
TITLE       PtSIP2-2
ORIGIN

        1 MVSKTRLIVS DFIVSIIWVW NGALIKMFVF KVLQMGHDSR GEFMRQSLTV VSLFFFAFLA
       61 KVTKGASFNP LAVLSSAISG DFSHFLFTIG TRIPAQVIGS ITAVRLLIDT FPEIGRGPRL
      121 NVDIHKGALT EGLLAFGVVT ISLGLARKIP GSFFMKTWIS SISKLSLHIL GSDLTGGCMN
      181 PASVMGWAYA RGDHITKEHI LVYWLAPIQG ALLAAYTFKL LFRPQKQDEK EKLKGKTD
//
LOCUS       PtTIP1-1       252 aa
DEFINITION  PtTIP1-1     252 aa
TITLE       PtTIP1-1
```

```
ORIGIN

        1 MPITSIAFGS PAEAGQPDAL RAALAEFISM LIFVFAGEGS GMAFNKLTDN GSSTPAGLVA
       61 ASLAHAFALF VAVSVGANIS GGHVNPAVTF GAFIGGHITF IRSLLYWVAQ CLGSVVACLL
      121 LKLATGGQET SAFALSSGVG AWNAVVFEIV MTFGLVYTVY ATAVDPKKGD IGIIAPIAIG
      181 FIVGANILAG GAFDGASMNP AVSFGPAVVS WTWDSHWVYW LGPFVGSAIA AIVYEVIFIN
      241 PSTHEQLPST DF
//
LOCUS       PtTIP1-2       252 aa
DEFINITION  PtTIP1-2     252 aa
TITLE       PtTIP1-2
ORIGIN

        1 MAITSIAFGS PAEVGQSDAL KAALAEFISM LIFVFAGEGS GMAFNKLTDD GSSTPAGLVA
       61 ASLAHAFALF VAVSVGANIS GGHVNPAVTF GAFLGGHITF IRSILYWVAQ CLGSVVACLL
      121 LKLATGGLET SAFSLSSGVG VWNAVVFEIV MTFGLVYTVY ATAVDPKRGD IGIIAPIAIG
      181 FIVGANILAG GAFDGASMNP AVSFGPAVVS WTWDNHWVYW LGPFVGSAIA AIVYEVCFIS
      241 PTTHEQLTSS DF
//
LOCUS       PtTIP1-3       252 aa
DEFINITION  PtTIP1-3     252 aa
TITLE       PtTIP1-3
ORIGIN

        1 MPINRIAFGT PREASHPDAL RAALAEFISM LIFVFAGSGS GMAFNKLTDN ASTTPSGLVA
       61 AALAHAFALF VAVSVGANIS GGHVNPAVTF GALIGGNITL LRSILYWIAQ LLGSVVACLL
      121 LKFATGGLET PAFGLSSGVG AWNALVFEIV MTFGLVYTVY ATAVDPKKGN LGIIAPIAIG
      181 FIVGANILAG GAFDGASMNP AVSFGPAVVS WTWTNHWVYW LGPFIGAAIA ALVYDNIFIG
      241 SGGHEPLPTN DF
//
LOCUS       PtTIP1-4       252 aa
DEFINITION  PtTIP1-4     252 aa
TITLE       PtTIP1-4
ORIGIN

        1 MPINRIAVGT PGEASHPDSL RAALAEFIST LIFVFAGSGS GMAFNKLTDS ASTTPAGLVA
       61 AALAHAFALF VAVSVGANIS GGHVNPAVTF GALIGGNITL LRSILYWIAQ LLGSVVACLL
      121 LKFSTGGLET PAFGLSSGVG AWNAVVFEIV MTFGLVYTVY ATAVDPKKGN LGIIAPIAIG
      181 FIVGANILAG GAFDGASMNP AVSFGPAVVS WTWTNHWVYW LGPFIGAGIA ALVYDNIFIG
      241 SGGHEPLPTN DF
//
LOCUS       PtTIP1-5       252 aa
DEFINITION  PtTIP1-5     252 aa
TITLE       PtTIP1-5
ORIGIN

        1 MPIRNIAVGH YRETTQPDAL KAALAEFIST LIFVFAGEGS GMAFSKLTDG ASNTPAGLIA
       61 AAIAHAFALF VAVSVGANIS GGHVNPAVTF GAFIGGNITL FRGILYWIAQ LLGSTVACLL
      121 LKFVTGGLET SAFALSTGVG VWNAFVLEIV MTFGLVYTVY ATAIDPKKGN LGIIAPIAIG
      181 FIVGANILVG GAFDGASMNP AVSFGPALVS WSWTNHWVYW AGPLVGGGLA GLIYELFFIG
      241 FGTHEQLPTT DY
//
LOCUS       PtTIP1-6       252 aa
DEFINITION  PtTIP1-6     252 aa
```

```
TITLE       PtTIP1-6
ORIGIN

        1 MPIRNIAVGH YHEATQPDAL RAALAEFIST LIFVFAGEGS GMAFAKLTDG AANTPAGLIA
       61 AAIAHAFALF VAVSVGANIS GGHVNPAVTF GAFIGGNITL LRGILYWIAQ LLGSTVACLL
      121 LKFTTGGLET SAFALSSGVG VWNAFVLEIV MTFGLVYTVY ATAVDPKKGN LGIIAPIAIG
      181 FIVGANILAG GAFDGASMNP AVSFGPALVS WTWTNHWVYW AGPLIGGGLA GLIYEFFFIG
      241 FGNHEQLPTA DY
//
LOCUS       PtTIP1-8       256 aa
DEFINITION  PtTIP1-8     256 aa
TITLE       PtTIP1-8
ORIGIN

        1 MRNFIIIERI TIGRVEDDFH SNAFKAALAE FISTLIFVFA GQGSTMAYNK LTSNAPTSPA
       61 GLIAVALAHA FGLFVGVAVS ANISGGHVNP AVTFGAFIGG NISLLRGILY WIAQLLGSTV
      121 ACLLLKYTTH HMTVSVFTLS PGVTVWNAFV FEIVMTFALV YTVYATAIDP KKGDVGVIAP
      181 LAIGFVLGAN ILVGGAFEGA ALNPAVPFGP ALVSWNWYHH WVYWAGPLIG GGLAGIVYEL
      241 IFMSHSTHEP LPGGEF
//
LOCUS       PtTIP1-7a       255 aa
DEFINITION  PtTIP1-7a     255 aa
TITLE       PtTIP1-7a
ORIGIN

        1 MPNLIVIDRI AIGTVAADFH PNAFKAALAE FISTLIFVFA GQGSTMAYNK LTSNAPTSPA
       61 GLIAVALAHA FGLFVAVATS ANISGGHCNP AVTFGAFLGG NITLLRGILY WIAQLLGSTV
      121 ACLLLKFTTH YMTVSVFTLS PGVSVWNAFV FEIVMTFALV YTVYATAIDA KKGDVGVIAP
      181 LAIGFVLGAN ILAGGAFEGA ALNPAVPFGP ALVSWNWHHH WVYWAGPLIG GGLAGVVYEL
      241 IFISHTHEPL PVVEY
//
LOCUS       PtTIP1-7b       255 aa
DEFINITION  PtTIP1-7b     255 aa
TITLE       PtTIP1-7b
ORIGIN

        1 MPNLIVIDRI AIGTVAADFH PNAFKAALAE FISTLIFVFA GQGSTMAYNK LTSNAPTSPA
       61 GLIAVALAHA FGLFVAVATS ANISGGHCNP AVTFGAFLGG NITLLRGILY WIAQLLGSTV
      121 ACLLLKFATH YMTVSVFTLS SGVSVWNAFV FEIVMTFALV YTVYATAIDA KKGDVGVIAP
      181 LAIGFVLGAN ILAGGAFEGA ALNPAVPFGP ALVSWNWHHH WVYWAGPLIG GGLAGVVYEL
      241 IFISHTHEPL AVVEY
//
LOCUS       PtTIP2-1       247 aa
DEFINITION  PtTIP2-1     247 aa
TITLE       PtTIP2-1
ORIGIN

        1 MAGIAFGRFD DSFSLGSFKA YLAEFISTLL FVFAGVGSAM AYNKLTGDAA LDPAGLVAIA
       61 VCHGFALFVA VSVGANISGG HVNPAVTFGL ALGGQITILT GIFYWIAQLL GSIVACYLLK
      121 VATGGLAVPI HSVAAGVGAI EGVVMEIIIT FALVYTVYAT AADPKKGSLG TIAPIAIGFI
      181 VGANILAAGP FSGGSMNPAR SFGPAVASGD FHDNWIYWAG PLVGGGIAGL IYGNVFITDH
      241 TPLSGDF
//
LOCUS       PtTIP2-2       247 aa
```

```
DEFINITION  PtTIP2-2     247 aa
TITLE       PtTIP2-2
ORIGIN

        1 MARIAFGRFN DSFSLGSLKA YLAEFISTLL FVFAGVGSAM AYNKLTGDAA LDPAGLVAIA
       61 VCHGFALFVA VAVGANISGG HVNPAVTLGL ALGGQMTILT GIFYWIAQLL GSIVACYLLK
      121 VVTGGLAVPI HSVAAGVGAI EGVVMEIIIT FALVYTVYAT AADPKKGSLG TIAPIAIGFI
      181 VGANILAAGP FSGGSMNPAR SFGPAVASGD FHDNWIYWVG PLIGGGLAGL IYGNLYITDH
      241 SPSSYEF
//
LOCUS       PtTIP2-3       250 aa
DEFINITION  PtTIP2-3     250 aa
TITLE       PtTIP2-3
ORIGIN

        1 MAKIAFGSLG DSFSLASIKA YLSEFIATLL FVFAGVGSAI AYSKLTTDAA LDPPGLVAVA
       61 VAHAFALFVG VSIAANISGG HLNPAVTFGL AIGGNITFLT GLLYWIAQCL GSIVACLLLK
      121 VVTSAEGIPT HGVASGMSAI EGVVMEIVIT FALVYTVYAT AADPKKGSLG IIAPIAIGFI
      181 VGANILAAGP FSGGSMNPAR SFGPAVVSGD FSQNWIYWLG PLVGGGLAGL VYGGIFIGSY
      241 APAPVSEDYA
//
LOCUS       PtTIP2-4       250 aa
DEFINITION  PtTIP2-4     250 aa
TITLE       PtTIP2-4
ORIGIN

        1 MVKIAFGSLG DSFSVGSLKA YLSEFIATLL FVFAGVGSAI AYSKLTTDAA LDPPGLVAVA
       61 VAHAFALFVG VSIAANISGG HLNPAVTFGL AIGGNITILT GLLYWIAQCL GSIAACLLLK
      121 FATSAESIPT HGVASGMSAV EGVVMEIVIT FALVYTVYAT AADPKKGSIG IIAPIAIGFI
      181 VGANILAAGP FSGGSMNPAR SFGPAVVSGD FSQNWIYWLG PLIGGGLAGL VYGDIFIGSY
      241 TAAPVSEDYA
//
LOCUS       PtTIP3-1       258 aa
DEFINITION  PtTIP3-1     258 aa
TITLE       PtTIP3-1
ORIGIN

        1 MPRRYAFGKA DEATRPDAMR AALAELVSTF IFVFAGEGSI LALDKLYKGT GPPASGLLVV
       61 ALAHALALFS AVASSINISG GHVNPAVTFG SLVGGRISVI RAVSYWVAQL LGSIFAALLL
      121 RLVTNGMIPA GFHVQSEVGE VHGLLLEMAL TFGLVYTVYA TAIDPKRGSL GIIAPLAIGF
      181 VVGANILVGG PFDGASMNPA RAFGPALVGW RWRNHWIYWV GPFLGGGLAA LIYEYIVISA
      241 EPVAHHTHQH QPLAPEDY
//
LOCUS       PtTIP3-2       258 aa
DEFINITION  PtTIP3-2     258 aa
TITLE       PtTIP3-2
ORIGIN

        1 MPRRHAFGRA DEATHPDSMR AALAEFVSTF VFVFAGEGSV LALDKLYKET GPLASGLVVV
       61 ALAHALALFS AVASSINISG GHVNPAVTFG SLVGGRISVI RAVYYWVAQL LGSIVAALLL
      121 RLVTNGMRPV GFHVQSGVGE VHGLLMEMAL TFGVVYTVYA TALDPKRGSL GIIAPLAIGF
      181 IVGANILVGG PFDGASMNPA RAFGPALIGW RWRNHWIYWV GPFLGGGLAA LIYEYIVIPT
      241 EPVPRHAHQH QPLAPEDY
//
```

```
LOCUS       PtTIP4-1       247 aa
DEFINITION  PtTIP4-1     247 aa
TITLE       PtTIP4-1
ORIGIN

        1 MTKIALGSRH EAAQPDCLKA LVVEFVTTFL FVFAGVGSAM AADKLTGDAL LGLFVVAVAH
       61 AFVVAVMISA GHISGGHLNP AVTIGLLFGG HITVVRSILY WIDQLLASTA ACFLLKYLTG
      121 GLATPVHTLA SGMDYLQGVV WEIVLTFSLL FTVYATIVDP KKGSIDGLGP MLTGFVVGAN
      181 ILAGGAFSGA SMNPARSFGP ALVSWDWTDH WVYWVGPLIG GGLAGFIYEN FFITRSHRPL
      241 PSEEEPF
//
LOCUS       PtTIP5-1       254 aa
DEFINITION  PtTIP5-1     254 aa
TITLE       PtTIP5-1
ORIGIN

        1 MASTSLTARF KQSVTPASLR AYLAEFISTF FYVFAVVGSA MASRKLLPDA AAVPSSLVIV
       61 AIANAFALSS AVYIAANASG GHVNPAVTFG MAVGGRINVP TALFYWISQM LASVMACIFL
      121 KVATVGQHVP TNTIAEEMTG FGASLLEGVM AFGLVYTVYA AGDPRRGSLG AIGPLAVGLT
      181 AGANVLAAGP FSGGSMNPAC AFGSAVIAGR LKNQAVYWVG PLIGAAVAGL LYDNVVFPTE
      241 APDSLRGVSD DVGV
//
LOCUS       PtTIP5-2       255 aa
DEFINITION  PtTIP5-2     255 aa
TITLE       PtTIP5-2
ORIGIN

        1 MAPTSLTARF QQSVTPASLR AYLAEFISTF FYVFAVVGSA MASRKLLPDA AADPSSLVIV
       61 AIANAFALSS AVYIAANASG GHVNPAVTFG MAVGGHINVP TALFYWISQL LASVMASIFL
      121 KVTTVGQHVP TYTIAEEMTG FGASLLEGVM TFGLVYTVYA AGDPRRSSLG AIGPLAVGLM
      181 AGANVLAAGP FSGGSMNPAC AFGSAVIAGK FKNQAVYWVG PLIGASVAGL LYDNVVFPTQ
      241 APDSGRRGVS EGVGV
//
LOCUS       PtXIP1-1       279 aa
DEFINITION  PtXIP1-1     279 aa
TITLE       PtXIP1-1
ORIGIN

        1 MAEALKNEGG KTKQITWREI LGLEDLLSLT VWRASVAELL GTAVLVFALD TIVISTIQTG
       61 TNMPNLILST LVAIIITILL LATFPISGGH INPIITFAAF LTGLISLSKT FIYILAQCVG
      121 AIFGALALKA VVNSEIEKTY SLGGCTLTIV APGPHGPTVI GLETNQALWL EIICGFVFLF
      181 ASVWMAFDHR QAQGIGRVGV FIIGGIVLGL LVFVSTTVTT TKGYAGAGLN PARCLGPAIV
      241 RGGHLWNGHW VFWVGPAVAC VAFAVYTKII PRQLAHTIE
//
LOCUS       PtXIP1-2       279 aa
DEFINITION  PtXIP1-2     279 aa
TITLE       PtXIP1-2
ORIGIN

        1 MAEALKNEGG KTKQITLREI LGLEDLLSLT VWRASVAELL GTAVLVFALD TIVISTIQTG
       61 TNMPNLILST LVAIIITILL LATFPISGGH INPIITFAAF LTGLISLSKT FIYILAQCVG
      121 AIFGALALKA VVNSEIEKTY SLGGCTLTIV APGPHGPTVI GLETNQALWL EIICGFVFLF
      181 ASVWMAFDHR QAQGIGRVRV LIIVGIVLGL LVFVSTTVTA TKGYAGAGLN PARCLGPAIV
      241 RGGHLWNGHW VFWVGPAIAC VAFAVYTKII PRQLAHTIE
```

```
//
LOCUS       PtXIP1-3       309 aa
DEFINITION  PtXIP1-3     309 aa
TITLE       PtXIP1-3
ORIGIN

        1 MAGYPGSTVE DEESLYSGKK PQPSATTPMA KVVQNEGGIQ KKKSPTLREI LGLEDLFSLT
       61 TWRASVAELL GTAVLVFALD TIVISTIQTQ TKTPNLILST LVAIIVTILL LATYPISGGH
      121 INPIVTFAAL LTGLISISKA FIYILAQCVG GIVGALALKA VVNSEIERTF SLGGCTLTVV
      181 APGPEGPTVV GLETGQALWL EIICGFVFLF ASVWMAFDHR QAKGLGRVNV LIIVGIVLGL
      241 LVYVSTTVTA TKGYAGAGLN PARCLGPAIV RGGHLWNGHW VFWVGPAIAC VAFAIYTKVI
      301 PSQLSHTIE
//
LOCUS       PtXIP1-4       271 aa
DEFINITION  PtXIP1-4     271 aa
TITLE       PtXIP1-4
ORIGIN

        1 MKKQKSTKLS EILGLEDLVS LTVWRASVAE LIGTAVLVFT LDTIVISTIR IETKIPNLIL
       61 SILAAIIITI LILATFPISG GHINPLVTFA ALLTGLVSLS KAIIYILAQC VGGIFGALAL
      121 KAVVNREIQQ TFSLGGCTLT VVAPGPDGQT VIGLETSQAL WLEIICGFVF LFASVWMAFD
      181 QRQAKALGRV NVFIIIGIVV GLLVYISTTV TATKGYAGAG LNPARCLGPA IVRGGHLWDG
      241 HWVFWVGPGI ACVLFALYTK LIPPQLSHTI E
//
LOCUS       PtXIP1-5       331 aa
DEFINITION  PtXIP1-5     331 aa
TITLE       PtXIP1-5
ORIGIN

        1 MAGNAGVVQD EEIGYGGNKV QPFASTPRPS KTERGKRDSS ALSRILGLDE LVSLNVWRAS
       61 LAEVFGTAVL VFAMDTIVIS SYETQTKTPN LVMATLIAIT IAILLLATFP ISGGHINPAI
      121 TLSAMFTGLI TVSRAAIYIL AQCIGAILGA LALKAVVNST IEQTFSLGGC TLEIVAPGPS
      181 GPVAIGLETG QALWLEIICT FVFLFSSIYI AFDRRQAIAL GRVVFCSIIG LVVGLLVFIS
      241 TTVTATKGYA GVGMNPARCL GPALVRGGHL WKGHWVFWVG PVVASVAFSL YTKMIPREHL
      301 LEQNQNREAF HVVVSVNVII HLLPFHGHNV V
//
LOCUS       PtXIP2-1a       240 aa
DEFINITION  PtXIP2-1a     240 aa
TITLE       PtXIP2-1a
ORIGIN

        1 MWRATLTELV ATTLTTCLLF TLTTSIISCL ESTTVEPKFL IPFAIIVIAF FFLLTTVPLS
       61 GGHMSPVFTF IAALEGVITP VRALFYMSAQ CVGSIVAYLV IKSVMDKNAE EKYSLGGCMI
      121 DGNGEGISPT NAFILEFSCT FIVLFVGVTV AFDKRRCKEL GLQMVCGILA GAMTLAFFVS
      181 ISVTGRAGYA GAGLNPARCL GPSLLKGGRL WYGHWVFWVG PFVACIVYYG FTLTLPTGTS
//
LOCUS       PtXIP2-1b       233 aa
DEFINITION  PtXIP2-1b     233 aa
TITLE       PtXIP2-1b
ORIGIN

        1 IWRATPTELV ATTLTTCLLF TLTTSIISCL ESTTVEPKFL IPFAIIVIAF FFLLTTVPLS
       61 GGHMSPVFTF IDALEGVITP VRALFSVSAQ CIGSIVAYLV IKKEKYSLDG CMIDGNGEGI
      121 SPTNAFILEF SCTFIVLFVG VTVAFDKRRC KELGLQMVCG ILAEAMTLAF FVSISVTGKA
```

```
      181 GYAGVGLNPA RCLGPSLLKG GRLWYGHWVF WVGPFVACIV YYRFTLTLPT EMS
//
LOCUS       RcNIP1-1       282 aa
DEFINITION  RcNIP1-1     282 aa
TITLE       RcNIP1-1
ORIGIN

        1 MASPNSITSE VSSKIQLPIK HSIPTEAKAS RSREWFVTDD ASPSVFQKIV AELVGTYILI
       61 FVGCGVALTD EVQRLTMVGI AIAWGVVLMA LIYAVGHVSG AHFNPAVSIA FAAGRKFPWK
      121 HVPMYILAQV LGSTLASLTL RVLFNDLDDI EVTVTQYKDS TSDLEAIIWE FIITFILMFN
      181 ILAVATDYRA VKYLSGVAIG GTLLFNALLA GPITGASMNP ARSLGPAIVS GVYKNLWVFI
      241 VSPIFGALAA TYVYNMLRVP EPEKSEEKTK NIFNHLYTAT DP
//
LOCUS       RcNIP1-2       271 aa
DEFINITION  RcNIP1-2     271 aa
TITLE       RcNIP1-2
ORIGIN

        1 MAEISGNGNH GVVLDIKDVN PPPSASKDSV LSFSVPFMQK LIAEMVGTYF LIFAGCTSVA
       61 VNLNFDKVVT LPGISIVWGL AVMVLVYSVG HISGAHFNPA VTLAFATCKR FPWKQVPAYI
      121 ACQVIGSTLA AGTIRLIFTG KQDHFTGTMP AGSDMQSFVV EFIITFYLMF IISGVATDNR
      181 AIGELAGLAV GATVLLNVMF AGPISGASMN PARSLGPAIV SHKYKGLWIY IVSPTLGAQA
      241 GAWVYNMIRY TDKPLREITK SASFLKSTGR A
//
LOCUS       RcNIP1-3       367 aa
DEFINITION  RcNIP1-3     367 aa
TITLE       RcNIP1-3
ORIGIN

        1 MSGENHVRSL EEGQCSDFVP PNNSKSDFCS SNATLIAETI GTYLVIFCGC GSVAVNKIYG
       61 SVTFPGICVV WGLIVMVMVY SVGHISGAHF NPAVTITFAI FRQFPYKQVP IYIVAQVVGS
      121 LLASGTLYYI FSVTDEAFFG TVPVGPPMRS FVLEIIISFL LMFVISGVAT DNRAIGELAG
      181 IAVGMTIMLN VFIAGPVSGA SMNPARTLGP AIVMRTYKGI WVYMAGPVIG AILGGFAYNL
      241 ISNFSHAPRL CCPGILVLFT IININPDAKK LESGLYYGSI IDMRSLYTKY EVQQEEWGLA
      301 ISLSNLLLML NVLLQIAMLT LTILVPLFVG PALLTFPLHS SKLFSQQNSA SSSSSHGTFM
      361 STKELRG
//
LOCUS       RcNIP1-4       252 aa
DEFINITION  RcNIP1-4     252 aa
TITLE       RcNIP1-4
ORIGIN

        1 MTNIEEGLVV TSTKSNPNPT SFGTCLSPKS VNIAQKVVAE LIGTYFVIFA GCGSVAVNNI
       61 YGSVTFPGVC VTWGLIVAVM IYSVGHISGA HFNPAVTITS AIFHRFPMHE VPLYIVAQVM
      121 GSILASGTLA LVVDVNPKAY FGTVPVGSNW QSLIMEIIIT FLLMFVISGV TTDDRTTAGP
      181 LGGIGVGMTI LLNVFVAGPV SGASMNPARS IGPAIVKHVY KGLWVYIVGP IVGAILGASA
      241 YNLLRSPYNQ TP
//
LOCUS       RcNIP2-1       297 aa
DEFINITION  RcNIP2-1     297 aa
TITLE       RcNIP2-1
ORIGIN

        1 METIIDPNLN NSSSSPASPE HLVSVENPKS EKSFLCLVQS FQNQYPPRFP RKVVAEVIAT
```

```
       61 YLLVFVTCGA AAISSADDKR ISKLGASLAG GLIVTVMIYA VGHVSGAHMN PAVTTAFAAV
      121 RHFPWKEVPY YAAAQLTGAI SASFTLKVLL HPVKHIGTTS PSGSDFQALV MEIVVTFCMM
      181 FVTSAVATDT KAIGELAGIA VGSAVCITSI LAGPISGGSM NPARTLGPAI ASAYYKGIWV
      241 YIVGPVVGTL LGSWSYNFIR VTDQPLQAIS PRSFSAKLRR IRSTNEQPTN KDPFDAL
//
LOCUS       RcNIP3-1       298 aa
DEFINITION  RcNIP3-1     298 aa
TITLE       RcNIP3-1
ORIGIN

        1 MPESEAGTPT VSAPNTPGTP GGPLFSALRI DSLSYDRKSM PRCKCFPVNA PTFGPPHTCF
       61 TDFPAPDISL TRKLGAEFVG TFILIFAATA GPIVNQKYNG VETLIGNAAC AGLAVMIIIL
      121 STGHISGAHL NPSLTIAFAA LRHFPWVQVP AYIAAQVSAS ICASFALKGV FHPFMSGGVT
      181 VPSVSTGQAF ALEFLITFNL LFVVTAVATD TRAVGELAGI AVGATVMLNI LVAGPSSGGS
      241 MNPVRTLGPA VAAGNYRALW IYLVAPTLGA IAGAGTYSAV KLREEEVDPP RPVRSFRR
//
LOCUS       RcNIP3-2       308 aa
DEFINITION  RcNIP3-2     308 aa
TITLE       RcNIP3-2
ORIGIN

        1 MDNEEVPSAP STPATPGTPG APLFGGFKGD RGNGNGVGFG RKSLLKSCKC FSVEEWSLEE
       61 GRLPPVSCSL LPPPVSLARK VGAEFIGTLI LMFAGTATAI VNQKTQGTET LIGLAVSTGL
      121 AVMIVILSTG HISGAHLNPS ITIAFAALRH FPWKHVPVYI GAQVSASVCA AFALKVIFHP
      181 FMSGGVTVPS GGYGQAFALE FIISFNLMFV VTAVATDTRA VGELAGIAVG ATVTLNILIA
      241 GETTGASMNP VRTLGPAIAA NNYKAIWVYL TAPILGALCG AGIYSAVKLP EEDGDAREKP
      301 STARSFRR
//
LOCUS       RcNIP3-3       298 aa
DEFINITION  RcNIP3-3     298 aa
TITLE       RcNIP3-3
ORIGIN

        1 MKMKHLLEEQ PSPDTFMNAS SSDASRDCSQ DTGSNALSTN GDIFAKYSNF GCFPKELDLN
       61 PARMVLAEFM GTFILMFCVC GIMASTQLTG GQVGLLEYAA TAGLTVIVLV FAIGPISGAH
      121 VNPAVTIAFA TFGHFPWSKV PFYVVAQTVG SVLATYAAKL VYGIKADLMV TRPVQGCNSA
      181 FSVEFITTFL MMFLAASLAY QAATRHLSGF VIGLSIGLAV LISGPVSGGS LNPARSLGPA
      241 IVSWNFKDIW VYIIAPTTGA VAGALMFHVL RIQRPPCSPT TPSPNTGLLG HSINFARR
//
LOCUS       RcPIP1-1       287 aa
DEFINITION  RcPIP1-1     287 aa
TITLE       RcPIP1-1
ORIGIN

        1 MEGKEEDVRL GANKYRETQP IGTAAQSQDD KDYTEPPPAP LFEPGELTSW SFYRAGIAEF
       61 IATFLFLYIS VLTVMGVVKA PTKCSTVGIQ GIAWAFGGMI FALVYCTAGI SGGHINPAVT
      121 FGLFLARKLS LTRALFYMVM QCLGAICGAG VVKGFEGSHD YTRLGGGANS VNPGYTKGDG
      181 LGAEIVGTFV LVYTVFSATD AKRSARDSHV PILAPLPIGF AVFLVHLATI PITGTGINPA
      241 RSLGAAIIFN KDQGWDEHWI FWVGPFIGAA LAALYHQVVI RAIPFKK
//
LOCUS       RcPIP1-2       288 aa
DEFINITION  RcPIP1-2     288 aa
TITLE       RcPIP1-2
ORIGIN
```

```
        1 MEGKEEDVRL GANKYRETQP IGTAAQSQDD KDYTEPPPAP LFEPGELTSW SFYRAGIAEF
       61 IATFLFLYIS VLTVMGVVKA PTKCSTVGIQ GIAWAFGGMI FALVYCTAGI SGGHINPAVT
      121 FGLFLARKLS LTRALFYMVM QCLGAICGAG VVKGFEGSHD YTRLGGGANS VNPGYTKGDG
      181 LGAEIVGTFV LVYTVFSATD AKRSARDSHV PILAPLPIGF AVFLVHLATI PITGTGINPA
      241 RSLGAAIIFN KDQGWDDHWI FWVGPFIGAA LAALYHQVVI RAIPFKKC
//
LOCUS       RcPIP1-3       286 aa
DEFINITION  RcPIP1-3     286 aa
TITLE       RcPIP1-3
ORIGIN

        1 MEGKEEDVTL GANRFPERQP IGTAAQTEKD YNEPPSAPLF LPGELKSWSY YRAGIAEFMA
       61 TFLFLYITVL TVMGYNRSPN KCASVGVQGI AWAFGGMIFA LVYCTAGISG GHINPAVTFG
      121 LLLARKLSLN RAIFYMVMQC LGAICGAGVV KGFQPTPYER VGGGANMVNP GYSKGDGLGA
      181 EIVGTFVLVY TVFSATDAKR SARDSHVPVL APLPIGFAVF LVHLATIPIT GTGINPARSL
      241 GAAIIYNKDH AWDDHWIFWV GPFIGAALAA LYHQIIIRAI PFKARA
//
LOCUS       RcPIP1-4       287 aa
DEFINITION  RcPIP1-4     287 aa
TITLE       RcPIP1-4
ORIGIN

        1 MEGKEEDVRL GANKFTERQP IGTSAQTDKD YKEPPPAPLF EPGELSSWSF YRAGIAEFMA
       61 TFLFLYITVL TVMGVSKSGN KCATVGTQGI AWAFGGMIFA LVYCTAGISG GHINPAVTFG
      121 LFLARKLSLT RALFYMVMQC LGAICGAGVV KGFEGDRTYE TLGGGANVVN AGYTKGDGLG
      181 AEIVGTFVLV YTVFSATDAK RNARDSHVPI LAPLPIGFAV FLVHLATIPI TGTGINPARS
      241 LGAAIIFNKD HAWDDHWIFW VGPFIGAALA AVYHQIVIRA IPFKSRA
//
LOCUS       RcPIP1-5       288 aa
DEFINITION  RcPIP1-5     288 aa
TITLE       RcPIP1-5
ORIGIN

        1 MEGREEDVSV GANRYGERQP IGTAAQTQDV KDYSEPPPAP LFEPGELSSW SFYRAGIAEF
       61 VATFLFLYIT VLTVMGVVKS PSKCSTVGIQ GIAWAFGGMI FALVYCTAGI SGGHINPAVT
      121 FGLFLARKLS LTRAVFYMIM QCLGAICGAG VVKALEKGHE YERLGGGANT VSSGYSKGDG
      181 LGAEIVGTFI LVYTVFSATD AKRNARDSHV PILAPLPIGF AVFLVHLATI PITGTGINPA
      241 RSLGAALIYN KDQAWDDHWI FWVGPFIGAA LAALYHQIVI RAIPFQSK
//
LOCUS       RcPIP2-1       287 aa
DEFINITION  RcPIP2-1     287 aa
TITLE       RcPIP2-1
ORIGIN

        1 MAKDVEVAEA TGGEFSAKDY HDPPPAPLID VDELGKWSFY RALIAEFIAT LLFLYITVLT
       61 VIGYKSQTDP DKNADACGGV GILGIAWAFG GMIFILVYCT AGISGGHINP AVTFGLFLGR
      121 KVSLIRALGY MVAQCLGAIC GCGLVKAFQK AYYNRYGGGA NELADGYNKG TGLGAEIIGT
      181 FVLVYTVFSA TDPKRSARDS HVPVLAPLPI GFAVFMVHLA TIPITGTGIN PARSFGAAVI
      241 YNKEKAWDDQ WIFWVGPFIG AAIAAFYHQY ILRAAAIKAL GSFRSNA
//
LOCUS       RcPIP2-2       280 aa
DEFINITION  RcPIP2-2     280 aa
TITLE       RcPIP2-2
```

```
ORIGIN

        1 MAKDVGEETQ TSHGKDYVDP PPAPLVDMAE LKLWSFYRAL IAEFIATLLF LYITVATVIG
       61 YKKQTDPCGG VGILGIAWAF GGMIFILVYC TAGISGGHIN PAVTFGLFLA RKVSLIRAVA
      121 YMVAQCLGAI CGVGLVKAFM KNPYNRLGGG ANSVASGYSN GTALGAEIIG TFVLVYTVFS
      181 ATDPKRSARD SHVPVLAPLP IGFAVFMVHL ATIPITGTGI NPARSFGAAV IYNNDKVWDD
      241 HWIFWVGPFV GALAAAAYHQ YVLRAAGIKA LGSFRSNPTN
//
LOCUS       RcPIP2-3       283 aa
DEFINITION  RcPIP2-3     283 aa
TITLE       RcPIP2-3
ORIGIN

        1 MVKDMEVGER GPFSAKDYHD PPPAPLIDAV ELTKWSFYRA LIAEFIATLL FLYITVLTVI
       61 GYKSQSESDS CGGVGILGIA WAFGGMIFIL VYCTAGISGG HINPAVTFGL FLARKVSLVR
      121 AIMYMVAQCL GAICGVGLVK AFQSSHYKRY GGGANTLDDN YSTGVGLGAE IIGTFVLVYT
      181 VFSATDPKRS ARDSHVPVLA PLPIGFAVFM VHLATIPITG TGINPARSLG AAVIYNQDKA
      241 WDDQWIFWVG PFIGAAIAAF YHQFILRAGA VKALGSFRSN PTV
//
LOCUS       RcPIP2-4       198 aa
DEFINITION  RcPIP2-4     198 aa
TITLE       RcPIP2-4
ORIGIN

        1 MIFVLVYCTA GISGGHINPA VTFGLLVARK VSLDRAVSYM ISQCLGAICG VGLVKAFMEH
       61 DYINITLGGG ANSVATGYSK GAALGAEIVG TFVLVYTVFS ATDPKRKARD SHVPILAPLP
      121 IGFAVFAVHL ATIPITGTGI NPARSLAAAI IYNTRKVWEE HVCPFSSFIY YTNICQRQRT
      181 INELCCKSCI QLSVSHMK
//
LOCUS       RcPIP2-5       288 aa
DEFINITION  RcPIP2-5     288 aa
TITLE       RcPIP2-5
ORIGIN

        1 MGKDVEVGGH GGEFHAKDYH DPPPAPLIDA EELTKWSFYR AIIAEFIATL LFLYITVLTV
       61 IGYKSQTDPA KNADACGGVG ILGIAWAFGG MIFILVYCTA GISGGHINPA VTFGLFLARK
      121 VSLVRAIMYM AAQCLGAICG CGLVKAFQRA YYNRYGGGAN ELADGYSTGT GLGAEIIGTF
      181 VLVYTVFAAT DPKRNARDSH VPVLAPLPIG FAVFMVHLAT IPVTGTGINP ARSFGAAVIY
      241 NKEKAWDDQW IFWVGPFIGA AIAAIYHQYV LRASAAKALG SFRSSSNI
//
LOCUS       RcSIP1-1       239 aa
DEFINITION  RcSIP1-1     239 aa
TITLE       RcSIP1-1
ORIGIN

        1 MRAIKAAAGD AVLTFMWVFC SSLFGFFTTL IATALGVQHH VWATLFITTV LVFVFVFLFG
       61 LIAEFLGGAS FNPTGTASFY AAGATADNLF SMALRFPAQA AGAVGGALAI LEVMPLQYKH
      121 MLGGPALKVD LHTGAIAEGV LTFFISFAVL VIILRGPRNL FVQNWLIAVV TVALVVTGSK
      181 YTGPSMNPAN AFGWAYINKW HNTWEQFYVY WISPFIGATL AAWIFRLIFP PPAPKQKKA
//
LOCUS       RcSIP1-2       234 aa
DEFINITION  RcSIP1-2     234 aa
TITLE       RcSIP1-2
ORIGIN
```

```
        1 MGVIKSAVGD AVLTSMWVFI APFLGVLTSI IASYVGIEPR SVPALFITIN LATLRLLTFS
       61 FIGALLGGAS FNPNTTVSLY AAGLKSDMSL VSMAVRFPAQ AAGGAGGATA MKILQALPRK
      121 YHHMLKGPTL NVDLHTGAVV GFLYCLALLL VMTKGPKNFL LKIWLLAWAR AVVGGEKSGT
      181 LMNPANAYGW AYVNNLHNTW EFFHVYWFCP FIGATLAAWN FRLLFKASAK HKQA
//
LOCUS       RcSIP1-3       239 aa
DEFINITION  RcSIP1-3     239 aa
TITLE       RcSIP1-3
ORIGIN

        1 MGVIKSAVGD AVLTSIWVFT LPFLGVLTSI VSTYVGVEPR SIPGLFITIN LATLLYLMFS
       61 FLGAALGGAS FNPATTVTLY ASGLRPDASL MSMAVRFPAQ AAGGVSGAMA ILQAMPRKYK
      121 HLLKGPSLKV DLHTGAIAEG VLSFMLCLAF LSLMSKGPKN SMLKLWLLAC VITGLAACGA
      181 KYTGPSLNPA NVYGWAYVHN WHNSWELFYV YWIGPLVGAT LSAWVFRFLF KPSSKQKQA
//
LOCUS       RcSIP2-1       240 aa
DEFINITION  RcSIP2-1     240 aa
TITLE       RcSIP2-1
ORIGIN

        1 MGGGVTGRLI ISDFVIAFMW VWSGALIKMF VNGVLRMGHE PSGEVLKSTL SIINMFFFAF
       61 LGKISKGAAY NPLTIFSSAI SGDFSQFLLT VGARIPAQVI GSITGVTLVI QTFPEIGFGP
      121 RLNVDIHRGA LTEGLLTFAI VTISLGLARK IPGSFFMKTW ISSVSKLTLH ILGSDLTGGV
      181 MNPASVMGWA YARGDHITKE HILVYWLAPV EATLLAVWTF KLLVRPTTQE RKENLKGKSD
//
LOCUS       RcTIP1-1       252 aa
DEFINITION  RcTIP1-1     252 aa
TITLE       RcTIP1-1
ORIGIN

        1 MAITSIAFGS PAEAGQPDAL KAALAEFISM LIFVFAGEGS GMAFNKLTSD GATTPAGLVA
       61 ASLAHGFALF VAVSVGANIS GGHVNPAVTF GAFVGGHITF IRSILYWIAQ LLGSVVACLL
      121 LKFSTGGLET SAFALSSGVG AWNAVVFEIV MTFGLVYTVY ATAVDPKKGN IGIIAPLAIG
      181 FIVGANILAG GAFDGASMNP AVSFGPAVVS WTWDNHWVYW LGPFVGAGIA AIVYETFFIS
      241 PSTHEQLPSA DF
//
LOCUS       RcTIP1-2       252 aa
DEFINITION  RcTIP1-2     252 aa
TITLE       RcTIP1-2
ORIGIN

        1 MPISRIAVGN PGEASQPDAL RAALAEFFSM IIFVFAGEGS GMAFNKLTDG GSTTPSGLVA
       61 ASLSHAFALF VAVSVGANIS GGHVNPAVTF GAFIGGHITL LRGILYWIAQ LLGSVVACLL
      121 LKFSTGGLET SAFALSSGVS SWNAVVFEIV MTFGLVYTVY ATAVDPKKGN IGIIAPIAIG
      181 FIVGANILAG GAFDGASMNP AVSFGPAVVS WTWTNHWVYW VGPLIGSAIA AIVYDNIFIG
      241 YGAHEPLPVN DF
//
LOCUS       RcTIP1-3       252 aa
DEFINITION  RcTIP1-3     252 aa
TITLE       RcTIP1-3
ORIGIN

        1 MPITRIAIGL PQHDILQSDA LKAALAEFFS TAIFVFAGEG SSMAFSKLTD DGSSTPAAII
```

```
      61 MASLAHAFGL FVGVSTAANI SGGHCNPAVT FGAFLGGNIS LLRGILYWIA QLLGSTVACL
     121 LLKFSTHGMT TSAFALSSGV NVWNAFVFEI VMTFGLVYTV YATAIDPKKS EVGIIAPLAI
     181 GFVVGANILA GGAFEGASMN PAVSFGPALV SWDWTNHWVY WAGPLIGGGL AGIVYDLFFI
     241 TYSHEPVPSS EL
//
LOCUS       RcTIP1-4       251 aa
DEFINITION  RcTIP1-4     251 aa
TITLE       RcTIP1-4
ORIGIN

       1 MPIRNIAVGH PHEATQPDAL KAALAEFIST LIFVFAGEGS GMAFNKLTNN GAATPSGLVA
      61 AAIAHAFALF VAVSVGANIS GGHVNPAVTF GAFVGGNITL LRGILYWIAQ LLGSTVACLL
     121 LKFSTGGLTT SAFALSSGVG VWNAFVFEIV MTFGLVYTVY ATAVDPKKGS LGTIAPIAIG
     181 FIVGANILAG GAFDGASMNP AVSFGPALVS WSWENHWVYW AGPLVGGGLA GLVYEFFFIH
     241 STHEQLPTTD Y
//
LOCUS       RcTIP2-1       248 aa
DEFINITION  RcTIP2-1     248 aa
TITLE       RcTIP2-1
ORIGIN

       1 MAGIAFGRFD DSFSLGSFKA YLAEFISTLL FVFAGVGSAI AYGKLTSDAA LDPAGLVAIA
      61 ICHGFALFVA VAVGANISGG HVNPAVTFGL ALGGQITILT GIFYWIAQLL GSIVACFLLK
     121 VVTGGLATPI HSVAAGVGAI EGVVMEIVIT FALVYTVYAT AADPKKGSLG TIAPIAIGFI
     181 VGANILAAGP FSGGSMNPAR SFGPAVVSGD FTDNWIYWVG PLVGGGLAGL IYGNLYMPGD
     241 HAPLSSDF
//
LOCUS       RcTIP2-2       203 aa
DEFINITION  RcTIP2-2     203 aa
TITLE       RcTIP2-2
ORIGIN

       1 MVKIAFGSLG DSFSAGSLKA YLSEFIATLL FVFAGVGSAI AYSKLTTDAA LDPPGLVAVA
      61 VAHAFGLFVG VAIAANISVS IPTHGVASGM SAIEGVVMEI VITFALVYTV YATAADPKKG
     121 DLGIIAPIAI GFIVGANILA AGPFSGGSMN PARSFGPAVV SGDFSENWIY WVGPLIGGGL
     181 AGLVYSCSFI GSYSAAPSSE EYA
//
LOCUS       RcTIP3-1       255 aa
DEFINITION  RcTIP3-1     255 aa
TITLE       RcTIP3-1
ORIGIN

       1 MPRRYAFGRA EEATHPDSIR ATLAEFVSTL IFVFAGEGSV LALDKLYRET GPPASGLVMV
      61 ALAHGLALFS AVSASINISG GHLNPAVTFG ALVGGRISVV LAFYYWIAQL LGAIVASLLL
     121 RLVTNGMRPV GFHVTSGVGE VHGLIMEMVM TFGLVYTVYA TAIDPKRGSL GIIAPLAIGF
     181 IVGANILVGG PFDGASMNPA RAFGPALVGW RWNNHWIYWL GPFVGGGLAA LIYEYMVIPT
     241 EPPHHTHQPL APEDY
//
LOCUS       RcTIP4-1       247 aa
DEFINITION  RcTIP4-1     247 aa
TITLE       RcTIP4-1
ORIGIN

       1 MAKIALGTRR EATQSDCIKA LIVEFITTFL FVFAGVGSAM AANKLLGDSL VGLFFVAMAH
```

```
      61 TLVVAVMISA GHISGGHLNP AVTLGLLAGG HITVVRSILY WIDQLLASSA ACFLLNYLTG
     121 GMATPVHTLA SGVGYVQGIV WEIVLTFSLL FTVYATIVDP KKGSIDGLGP TLTGFVVGAN
     181 ILAGGPFSGA SMNPARSFGP ALVSWDWTDH WVYWVGPLIG GGLAGFIYEN FFIIRSHRPL
     241 PNDEENY
//
LOCUS       RcTIP5-1       252 aa
DEFINITION  RcTIP5-1     252 aa
TITLE       RcTIP5-1
ORIGIN

       1 MAPTSLLARF EQSVTRDALR SYLAEFISTF FFVFAVVGSS MASRKLMPAA DPSNLVIVAL
      61 ANAFALSSAV YIAANISGGH VNPAVTFSMA IGGHISVPTA LFYWVSQLVA SVMACLLLRV
     121 AAVGQNLPTY IIAEEMTGFG ASIVEGVLTF GLVYTVYAAG DPRRSLLGAT GPLVIGLMAG
     181 ANVLAAGPFS GGSMNPACAF GSAVVAGRFK NQAVYWVGPL LGATVAGLLY DNVVFPNQVP
     241 DSIRGISDGV GA
//
LOCUS       RcXIP1-1       303 aa
DEFINITION  RcXIP1-1     303 aa
TITLE       RcXIP1-1
ORIGIN

       1 MALGAENQAC SNLKGRQSPN SSFCTRIGVH ELYSLEMWRA AVTELVASAT FLFTLSTTII
      61 ACLESHETSP KLVIPVVVFL IAFFWLLTTV PLSGGFFSPT FAFMAALSGV ISFVRALFYC
     121 LGQCFGAIIG YMILKSVMDP TIAHKYALGG CMVNGGGEGV SAGTALMVEF SCTFLVLYVA
     181 MTIILDKKKC QELGLTMVCV IISGAYAVSV FVSTTVTGRI GYGGVGLNPA RCLAPALLLG
     241 GSLWDGHWVF WVGPICSCSV YYVFSLLLPK QGFVRADEQQ HLIQLVRSSC LGSESPDYFE
     301 GKV
//
LOCUS       RcXIP1-2       309 aa
DEFINITION  RcXIP1-2     309 aa
TITLE       RcXIP1-2
ORIGIN

       1 MALGAENRAF SDSGQALNVM GRQFPNSSFL TRLGVHELYS PEMWRAAFTE LVASATFLFT
      61 LSTTIIACLE SHETAPKLVI PVAVFFIALF WLLPTVPLSG GFFSPTFTFM AALRGVISFT
     121 RALFYCLGQC LGAIIGYIIL KSVMDPTIAH KYALGGCMVN GNGEGVSAGT ALMIEFSCTF
     181 LVLYTAMTIV LDKKKCQDLG LTMVCIIISG AYAVSVFVST TVTGRVGYGG VGLNPARCLA
     241 PALLLGGALW DGHWVFWVGP VCACSVYYVC SLMLPKQGFV RADEEQHVLQ LVRSSCLGSE
     301 SANYFEGKV
//
LOCUS       RcXIP1-3       243 aa
DEFINITION  RcXIP1-3     243 aa
TITLE       RcXIP1-3
ORIGIN

       1 MADNLRVIAD EENGYGGRRV QPFASTPLGA ALDNTNGGKK QNPTTFSRVL GLEELSSLNV
      61 WRASVAEVLG TAALVFATDT IVISTYETET KTPNLIMAAL IAMTVTILLT ATFPISGGHI
     121 NPVITISAAF TGLVSPVRAA VYILAQCLGA TLGALLVATS CLLFTLTISI ISSLESHESE
     181 PKILVPLAVF IIAFFFLLTT VPLSGAGINP ARCFGPAIVR GDHLWNGHWV FWVGPFIACV
     241 AFT
//
LOCUS       RcXIP2-1       285 aa
DEFINITION  RcXIP2-1     285 aa
TITLE       RcXIP2-1
```

```
ORIGIN

        1 MWRAAVTELV ATGTFLFTLS TTIIACLESH ESDPKLLIPI AVFFIAFLWL MVTVPLSGGL
       61 FSPAFSFIAA LRGVITFVRA LFYSLGQLLG ALIAYLILKG VMDPNMAHKY ALAGCMVNGN
      121 GAGVSVGTAL IIEILCTFMV LYVAMTIILD KQKCMDLGLT TVCVIISGIY AASVFVSITV
      181 TGRPGYGGVG LNPARCLGPV LLMGGALWEG HWVFWVGPFC ACMIYYAYSL MLPKRGFVRA
      241 DIEEDIIKLV RASCSGSDCP SCVEKKVTSL YEKILYKLSF GFICD
//
LOCUS       RcXIP2-2       208 aa
DEFINITION  RcXIP2-2     208 aa
TITLE       RcXIP2-2
ORIGIN

        1 MSSQQAANVK DSSKYAMTTF LARIGAHEFF SPEMWRAVAT ELVATACLLF MLTTTIIACL
       61 ESKETEPKLL IPIVVFVIVF LVLVVTVPLS GGHMSPVFTF IAALRGLISL VRALFYVLAQ
      121 CVGSIMAYLV IKSVMDETVA DKYALGGCMV NGNGAGVSTG TALVIEFACT FLVLYVAITV
      181 AFDKKMCQEL GLAILIRQFG LKATIYYL
//
LOCUS       SbNIP1-1       287 aa
DEFINITION  SbNIP1-1     287 aa
TITLE       SbNIP1-1
ORIGIN

        1 MAGGGDHNSA QTNGGGHDQR AMEEGRKEAE YADHQGCAAM VVSVPFIQKI IAEIFGTYFL
       61 MFAGCGAVTI NASKNGQITF PGVAIVWGLA VMVMVYAVGH ISGAHFNPAV TFAFATSGRF
      121 PWRQLPAYVL AQMLGAVLAS GTLRLMFGGR HEHFPGTLPT GSDVQSLVIE IITTFYLMFV
      181 ISGVATDNRA IGELAGLAVG ATILLNVLIA GPVSGASMNP ARSVGPALVS GEYRSIWVYV
      241 VGPLVGAVAG AWAYNLIRFT NKPLREITKS TSFLKSMSTN RMNSAAA
//
LOCUS       SbNIP1-2       271 aa
DEFINITION  SbNIP1-2     271 aa
TITLE       SbNIP1-2
ORIGIN

        1 MEEGQAGYQS SEDGSHGSGS ASNRCNDDMI SVQFMQKIIA EVLGTYFMIF AGCGSVVVNL
       61 STNGTVTFPG ICAVWGLVVM VLVYSVGHIS GAHFNPAVTV AFATCGRFPW KQVPSYAVAQ
      121 VLGSTLASLT LRVVFGGATA HEHFFGTAPS GTVAQAVVLE FVISFYLMFV VSGVATDNRA
      181 IGELAGLAVG ATVLLNVLVA GPITGASMNP ARTLGPAIVA GRYRSIWVYM VGPVCGTVTG
      241 AWAYNLVRFT DKPLREITKS GSFLRATGRT S
//
LOCUS       SbNIP1-3       283 aa
DEFINITION  SbNIP1-3     283 aa
TITLE       SbNIP1-3
ORIGIN

        1 MAGAEVANGA VHEGGALALE EGRGGDEARC ESSEQDGAGR SRPMFSVPFV QKIVAEVLGT
       61 YFLIFAGCAA VAVNLRTGGT VTFPGICIVW GLAVMVMVYS VGHISGAHLN PAVSVAFATC
      121 GRFPWKQVPA YAAAQVMGAT AASLTLRLLF GNAREHFFGT VPAGSDVQSL VIEFIISFNL
      181 MFVVSGVATD NRAIGELAGL AVGATVLLNV LFAGPVSGAS MNPARTLGPA IVVGRYAGIW
      241 VYFAGPICGT VAGAWAYNLI RFTDKPLREI TQTSSFLRSV RRT
//
LOCUS       SbNIP2-1       297 aa
DEFINITION  SbNIP2-1     297 aa
TITLE       SbNIP2-1
```

```
ORIGIN

        1 MSTNSRSNSR ANFNNEIHDI GTVQNSTMMP PTYYDRSLAD IFPPHLLKKV VSEVVSTFLL
       61 VFVTCGAAGI YGSDKDRISQ LGQSVAGGLI VTVMIYAVGH ISGAHMNPAV TLAFAVFRHF
      121 PWIQVPFYWA AQFTGAICAS FVLKAVLHPI SVLGCTTPTG PHWHSLIIEI IVTFNMMFVT
      181 LAVATDTRAV GELAGLAVGS AVCITSIFAG AVSGGSMNPA RTLGPALASN LYTGLWIYFL
      241 GPVLGTLSGA WTYTYIRFEE APSTHKDMSQ KLSSFKLRRL QSQSVAAEDD ELDHIQV
//
LOCUS       SbNIP2-2       295 aa
DEFINITION  SbNIP2-2     295 aa
TITLE       SbNIP2-2
ORIGIN

        1 MAASTASRTN SRVNYSNEIH DLSTVQSGSA VPTLFYPDKS IADIFPPHLG KKVISEVVAT
       61 FLLVFVTCGA ASIYGEDNKR ISQLGQSVAG GLIVTVMIYA TGHISGAHMN PAVTLSFACF
      121 RHFPWIQVPF YWAAQFTGAM CAAFVLKAVL HPIAVIGTTT PSGPHWHALV IEIVVTFNMM
      181 FVTCAVATDS RAVGELAGLA VGSAVCITSI FAGPVSGGSM NPARTLAPAV ASNVFTGLWI
      241 YFLGPVIGTL SGAWVYTYIR FEEAPAAKDT QRLSSFKLRR LQSQSALAAD EFDTV
//
LOCUS       SbNIP3-1       289 aa
DEFINITION  SbNIP3-1     289 aa
TITLE       SbNIP3-1
ORIGIN

        1 MDGRRRSVSS VDVSLSIPSA PAASMLVDKD NMSDDKISIF IPQRSPSNKI RPLGFQQNEA
       61 SNDPPPLSAK SMTLALIKKV VAEFLGTFLL IFTVVSALIM NETHNGALGL LGVAATAGMA
      121 VVVIVSSIFH VSGGQLNPAV SVTMVVFGHL PPAHLVPYIV AQLLGSTAAS FVAKALYDPV
      181 NLGAIVATVP RIGAFEAFWV EFITTFILLF VITALATDTR AVKELVAVGA GAAVMMSALI
      241 SGESTGASMN PARTLGTAIA TGIYTKIWIY VVAPPLGAIA GCGAYHALK
//
LOCUS       SbNIP3-2       297 aa
DEFINITION  SbNIP3-2     297 aa
TITLE       SbNIP3-2
ORIGIN

        1 MADQTTVSVE EQRQVAISVL CSTPSERSLA VAVAAGGSAS TPRSNTSKQL VPLDSLQKLM
       61 LKSPPQADVV EDDEQTQLVP LAKKVAAEFI GTFILMFAVV STIVADAQHG GGAEGLVGVA
      121 ASAGLAVVAV VLAVVHVSGS HLNPAVSLAM AVFGHLPRAH VLPYAAAQTM GSLAATFLAK
      181 AMYRPADPAV MATVPRAGVG AAQAFFLELV LTFVLVFVIT AVATDPTSSK ELVAIAIAAA
      241 IMMNALIGGP STGPSMNPAR TIAAALATGK YKDIWVYLLA PPLGAIAGAA TYTLIKP
//
LOCUS       SbNIP3-3       297 aa
DEFINITION  SbNIP3-3     297 aa
TITLE       SbNIP3-3
ORIGIN

        1 MEEQKRSMGD VTAVAVPPMQ TSESNKISII ISPRAATSKI MPFELIHAGS VSSRPHADVA
       61 ESSGAHATHH HRWNQGLPKI NAVPLIKKVG AEFLGTFILI FTVSSTIIMN EQHDGVESLL
      121 GIATSAGLAV TVLVLSLIHI SGCHLNPAVS IAMAVFGHLP LAHILPYVAA QILGSIAASF
      181 TVKGIYHPVN PGIATIPKVG TTEAFFLEFI TTFVLLFIIT ALATDPHAVK ELIAVAVGAT
      241 IMMNALVAGP STEASMNPAR TLGPAIATCR YTQIWIYMVA TPLGAIAGTG AYVAIKL
//
LOCUS       SbNIP3-4       301 aa
DEFINITION  SbNIP3-4     301 aa
```

```
TITLE       SbNIP3-4
ORIGIN

        1 MAEPGSTTPP NGSAPATPGT PAPLFSGGPR VDSLSYERKS MPRCKCLPAV EGWGIATHTC
       61 VVEIPAPDVS LTRKLGAEFV GTFILIFFAT AAPIVNQKYG GAISPFGNAA CAGLAVTIII
      121 LSTGHISGAH LNPSLTIAFA ALRHFPWLQV PAYVSVQVLG SICASFALKG VFHPFLSGGV
      181 TVPDVTISTA QAFFTEFIIS FNLLFVVTAV ATDTRAVGEL AGIAVGAAVT LNILVAGPTT
      241 GGSMNPVRTL GPAVAAGNYR QLWIYLLAPT LGALAGAGVY TAVKLRDENG ETPRTQRSFR
      301 R
//
LOCUS       SbNIP4-1       289 aa
DEFINITION  SbNIP4-1     289 aa
TITLE       SbNIP4-1
ORIGIN

        1 MAEKDFADGV GAVDEQDLER SCRDQDDPAA AADGVSRGLA IGHFVRELMV ECVASFLLVF
       61 WSAVAALMQE MHGTLTFPMV CLVVALTVGF VLCWLGPAHF NPAVTLTFTV FGYLPWPKLP
      121 LYVVAQLAGS LLACVAANGV MKPREEHFYG TAPMMAGGHT RLPFLLELVA SAVLMIVIAI
      181 AARGSNQTAG GLAIGAAVGT LGLVIGPVSG GSMNPIRTLG PAIVLGRYTS VWIYLVAPVA
      241 GMLIGALCNR VVRGSDAILA FLCGTKPTRA VAPRATPRAV GSLASSPHY
//
LOCUS       SbPIP1-1       289 aa
DEFINITION  SbPIP1-1     289 aa
TITLE       SbPIP1-1
ORIGIN

        1 MEGKEEDVRL GANKFSERQP IGTAAQGAGD DKDYKEPPPA PLFEPGELKS WSFYRAGIAE
       61 FVATFLFLYI TILTVMGVSK SNSKCATVGI QGIAWSFGGM IFALVYCTAG ISGGHINPAV
      121 TFGLFLARKL SLTRAIFYII MQCLGAICGA GVVKGFQQGL YMGNGGGANV VAPGYTKGDG
      181 LGAEIVGTFI LVYTVFSATD AKRNARDSHV PILAPLPIGF AVFLVHLATI PITGTGINPA
      241 RSLGAAIIYN RDHAWSDHWI FWVGPFIGAA LAAIYHQVII RAIPFKSRS
//
LOCUS       SbPIP1-2       288 aa
DEFINITION  SbPIP1-2     288 aa
TITLE       SbPIP1-2
ORIGIN

        1 MEGKEEDVRL GANKYSERQP IGTAAQGTDD KDYKEPPPAP LFEPGELKSW SFYRAGIAEF
       61 VATFLFLYIS ILTVMGVSKS TSKCATVGIQ GIAWSFGGMI FALVYCTAGI SGGHINPAVT
      121 FGLFLARKLS LTRAVFYIIM QCLGAICGAG VVKGFQQGLY MGNGGGANVV APGYTKGDGL
      181 GAEIVGTFVL VYTVFSATDA KRNARDSHVP ILAPLPIGFA VFLVHLATIP ITGTGINPAR
      241 SLGAAVVYNQ NHAWSDHWIF WVGPFIGAAL AAIYHQVIIR AIPFKSRS
//
LOCUS       SbPIP1-3       290 aa
DEFINITION  SbPIP1-3     290 aa
TITLE       SbPIP1-3
ORIGIN

        1 MEGKEEDVRL GANRYSERQP IGTAAQGTEE NSKDYKEPPA APLFEAEELT SWSLYRAGIA
       61 EFVATFLFLY ISILTVMGVS KSGSKCGTVG IQGIAWSFGG MIFALVYCTA GISGGHINPA
      121 VTFGLLLARK LSLTRALLYM VMQCLGAICG AGVVKGFQQT LYMGAGGGAN SVNPGYTKGD
      181 GLGAEIVGTF VLVYTVFSAT DAKRSARDSH VPILAPLPIG FAVFLVHLAT IPITGTGINP
      241 ARSLGAAIVY NRSNAWNDHW IFWVGPFIGA ALAAIYHVVI IRAIPFKSRD
//
```

```
LOCUS       SbPIP1-4       296 aa
DEFINITION  SbPIP1-4     296 aa
TITLE       SbPIP1-4
ORIGIN

        1 MAGGNRSQDE DVRVGVDRFP ERQPIGTTAA DDLGGRDYSE PPAAPLFESS ELSSWSFYRA
       61 GIAEFVATFL FLYVTVLTVM GVSKSPSKCG TVGIQGIAWA FGGMIFALVY CTAGVSGGHI
      121 NPAVTFGLLL ARKLSLARAV YYVVMQCLGA VCGAGVVKAL VGSALYQSAG GGANAVAPGY
      181 TKGDGLGAEI VGTFVLVYTV FSATDAKRSA RDSHVPVLAP LPIGFAVFLV HLATIPITGT
      241 GINPARSLGA AIIYDSPHGW HGHWIFWVGP FAGAALAAVY HQVVIRAIPF KSSAHY
//
LOCUS       SbPIP2-1       286 aa
DEFINITION  SbPIP2-1     286 aa
TITLE       SbPIP2-1
ORIGIN

        1 MAKEVDVSAL EAGGARDYID PPPAPLVDIH ELGKWSLYRA VIAEFVATLL FLYITVATVI
       61 GYKHQTDATA SGADAACSGV GILGIAWAFG GMIFILVYCT AGISGGHINP AVTFGLFLAR
      121 KVSLVRALLY MAAQSLGAIC GVALVKGFQS GFYARYGGGA NEVSPGYSTG TGLAAEIIGT
      181 FVLVYTVFSA TDPKRNARDS HVPVLAPLPI GFAVFMVHLA TIPITGTGIN PARSLGAAVV
      241 YNNSKAWSDQ WIFWVGPFIG AAIAALYHQI VLRASARGYG SFRSNA
//
LOCUS       SbPIP2-2       286 aa
DEFINITION  SbPIP2-2     286 aa
TITLE       SbPIP2-2
ORIGIN

        1 MAKEVDVSTL EAGGARDYID PPPAPLVDID ELGKWSLYRA VIAEFVATLL FLYITVATVI
       61 GYKHQTDATA SGADAACGGV GILGIAWAFG GMIFILVYCT AGISGGHINP AVTFGLFLAR
      121 KVSLVRALLY MAAQSLGAIC GVALVKGFQS GFYTRYGGGA NEVSPGYSTG TGLAAEIIGT
      181 FVLVYTVFSA TDPKRNARDS HVPVLAPLPI GFAVFMVHLA TIPITGTGIN PARSLGAAVV
      241 YNNSKAWSDQ WIFWVGPFIG AAIAALYHQI VLRASARGHG SFRSNA
//
LOCUS       SbPIP2-3       286 aa
DEFINITION  SbPIP2-3     286 aa
TITLE       SbPIP2-3
ORIGIN

        1 MGKEVDVSAL EAGGVRDYAD PPPVPLIDID ELGKWSLYRA VIAEFVATLL FLYITVATVI
       61 GYKHQTDATA SGADAACGGV GILGIAWAFG GMIFILVYCT AGISGGHINP AVTFGLFLAR
      121 KVSLVRALLY MAAQSLGAIC GVALVKGFQS GFYARYGGGA NEVSPGYSTG TGLAAEIIGT
      181 FVLVYTVFSA TDPKRNARDS HVPVLAPLPI GFAVFMVHLA TIPITGTGIN PARSLGAAVV
      241 YNNSKAWSDQ WIFWVGPFIG AAIAALYHQI VLRASARGHG SFRSNA
//
LOCUS       SbPIP2-4       286 aa
DEFINITION  SbPIP2-4     286 aa
TITLE       SbPIP2-4
ORIGIN

        1 MGKEVDVSTL EAGGVRDYAD PPPAPLIDID ELGKWSLYRA VIAEFVATLL FLYITVATVI
       61 GYKHQTDATA SGADAACGGV GILGIAWAFG GMIFILVYCT AGISGGHINP AVTFGLFLAR
      121 KVSLVRAILY MAAQSLGAIC GVALVKGFQS GFYARYGGGA NEVSPGYSTG TGLAAEIIGT
      181 FVLVYTVFSA TDPKRNARDS HVPVLAPLPI GFAVFMVHLA TIPITGTGIN PARSLGAAVV
      241 YNNSKAWSDQ WIFWVGPFIG AAIAALYHQI VLRASARGYG SFRSNA
```

```
//
LOCUS       SbPIP2-5       197 aa
DEFINITION  SbPIP2-5     197 aa
TITLE       SbPIP2-5
ORIGIN

        1 MIFILVYCTA GISGGHINPA VTFGLFLARK VSLVRAILYM AAQSLGAICG VALVKGFQSG
       61 FYARYGGGAN GVSAEYSTGT GLAAEIIGTF VLVYTVFSAT DPKRNARDSH VPVLAPLPIG
      121 FAVFVVHLAT IPITGTGINP ARSLGAAVVY NNSKAWSDQW IFWVGPFIGA AIAALYHQIV
      181 LRASGRGYGS RSFRSYA
//
LOCUS       SbPIP2-6       290 aa
DEFINITION  SbPIP2-6     290 aa
TITLE       SbPIP2-6
ORIGIN

        1 MGKDDVIESG AGGGEFAAKD YTDPPPAPLI DAAELGSWSL YRAVIAEFIA TLLFLYITVA
       61 TVIGYKHQTD ATASGADAAC GGVGVLGIAW AFGGMIFVLV YCTAGISGGH INPAVTFGLF
      121 LARKVSLVRA LLYIVAQCLG AICGVGLVKA FQSAYFDRYG GGANSLASGY SRGTGLGAEI
      181 IGTFVLVYTV FSATDPKRNA RDSHVPVLAP LPIGFAVFMV HLATIPVTGT GINPARSLGA
      241 AVIYNKDKPW DDHWIFWVGP FVGAAIAAFY HQYILRAGAI KALGSFRSNA
//
LOCUS       SbPIP2-7       292 aa
DEFINITION  SbPIP2-7     292 aa
TITLE       SbPIP2-7
ORIGIN

        1 MAKDIEAAAA AHGGGGEYTA KDYSDPPPAP LVDAEELTKW SLYRAVIAEF VATLLFLYIT
       61 VATVIGYKHQ TDASSSGPDA ACGGVGILGI AWAFGGMIFI LVYCTAGVSG GHINPAVTFG
      121 LFLARKVSLV RALLYIVAQC LGAICGVGLV KGFQSAFYVR YGGGANGLSD GYSKGTGLAA
      181 EIIGTFVLVY TVFSATDPKR NARDSHVPVL APLPIGFAVF MVHLATIPIT GTGINPARSL
      241 GAAVIYNNDK TWDDHWIFWV GPFIGAAIAA AYHQYVLRAS AAKLGSSASF SR
//
LOCUS       SbPIP2-8       289 aa
DEFINITION  SbPIP2-8     289 aa
TITLE       SbPIP2-8
ORIGIN

        1 MAKDIEASGP EAGEFSAKDY TDPPPAPLID AAELTQWSLY RAVIAEFIAT LLFLYITVAT
       61 VIGYKHQTDA TASGPDAACG GVGILGIAWS FGGMIFILVY CTAGISGGHI NPAVTFGLFL
      121 ARKVSLVRAL LYIIAQCLGA ICGVGLVKGF QSAYYVRYGG GANELSDGYS KGTGLAAEII
      181 GTFVLVYTVF SATDPKRNAR DSHVPVLAPL PIGFAVFMVH LATIPITGTG INPARSLGAA
      241 VIYNNDKAWD DQWIFWVGPL IGAAIAAAYH QYVLRASASK LGSSYRSNA
//
LOCUS       SbPIP2-9       282 aa
DEFINITION  SbPIP2-9     282 aa
TITLE       SbPIP2-9
ORIGIN

        1 MDPCHQSIET AGAKDYSDPP PAPLVNAGEL GKWSLYRAAI AEFVATLLFV YVTLATVIGH
       61 KRQAESQPCG SVGVLGIAWS FGGMIFVLVY CTAGISGGHI NPAVTFGLLL ARKVSLVRAA
      121 LYVVAQCLGA MCGAGLVKAF HGAHWYLHYG GGANELSAGY SKGAGLAAEI VGTFVLVYTV
      181 FSATDPKRKV RDSHVPVLAP LPIGFAVFMV HLATIPVTGT GINPARSLGP AVVYNQRKAW
      241 EDQWIFWVGP LIGAAAAMIY HQLVLRAGAA KALASLRNNY HI
```

```
//
LOCUS       SbPIP2-10       289 aa
DEFINITION  SbPIP2-10     289 aa
TITLE       SbPIP2-10
ORIGIN

        1 MSKDDVTAEA EAAAKAPYWD PPPAPLLDTS ELKKWSLYRA LIAEFMATLI FLYVSIATVI
       61 GYKNQSKAES CTGVGALGIA WSFGATIFIL VYCTGGISGG HINPAVTFGL FVGRKLSLVR
      121 TLLYIVAQCL GAICGVGIVK GIMKVPYNSL GGGANAVATG PSDGVLTGYS VGSALAAEIV
      181 GTFILVYTVF SATDPKRTAR DSFIPVLVPL PIGFAVFVVH LATIPITGTG INPARSLGAA
      241 VVHAAWKDHW IFWVGPLIGA TVAALYHKLV LRGEAVKALG SFRSTSATV
//
LOCUS       SbSIP1-1       246 aa
DEFINITION  SbSIP1-1     246 aa
TITLE       SbSIP1-1
ORIGIN

        1 MAMGAAVRAA AADAVVTFLW VLCASALGAS TAAVTSCLGV QEGAGGHYAL LVTASLLAVL
       61 LFAFDRLCGA LGGASFNPTD FAASYAAGLD SPSLFSVALR FPAQAAGAVG GALAISELMP
      121 AQYKHTLAGP SLKVDPHTGA LAEGVLTFVV TLAVLCVIVK GPRNAILRIL LISVSIVSLI
      181 VAGAEYTGPS MNPANAFGWA YVNNWHNTWE QLYVYWICPF IGALLAGWIF RAVFLPPAPK
      241 PKTKKA
//
LOCUS       SbSIP1-2       243 aa
DEFINITION  SbSIP1-2     243 aa
TITLE       SbSIP1-2
ORIGIN

        1 MAMGPALRAA AADAVVTFLW VLCVSTLGAS TAAVTSYLSL QGVHYALLVT VSLLSVLLFT
       61 FNILCDALGG ASFNPTGIAA FYAAGVTSPS LFSVALRLPA QAAGAVGGAL AISELMPAQY
      121 KHMLGGPSLK VDPHTGAIAE LVLTFVITLA VLLIIVKGPR NPIIKTWMIS ICTLCLVLSG
      181 AAYTGPSMNP ANAFGWAYVN NRHNTWEQFY VYWICPFIGA ILAAWIFRAL FLAPPPKPKA
      241 KKA
//
LOCUS       SbSIP2-1       249 aa
DEFINITION  SbSIP2-1     249 aa
TITLE       SbSIP2-1
ORIGIN

        1 MSPAPSRPRI RPWLVVGDLA LAAAWVCAGA LVKLLVYGGL GLAGRPEAEA VKVSLSIVYM
       61 FIFAWLEVAL GGASYNPLTV LAAALASHGG PAVYVFTAFA RIPAQVIGAV LGVNLIQLTF
      121 PNVGKGARLS VGAHHGALAE GLATFMVVMV SVTLKKKEMK SFFMKTWITS MWKNTIHILS
      181 SDITGGIMNP ASAFAWAYAR GDHTTFDHLL VYWLAPLQAT LLGVWAVTYL TKSKKIKEQE
      241 ADENKTKKE
//
LOCUS       SbTIP1-1       250 aa
DEFINITION  SbTIP1-1     250 aa
TITLE       SbTIP1-1
ORIGIN

        1 MPISRIAVGT HHEVYHPGAL KAALAEFIST LIFVFAGQGS GMAFSKLTSG GATTPSGLIA
       61 AAVAHAFALF VAVSVGANIS GGHVNPAVTF GAFVGGNITL FRGLLYWVAQ LLGSTVACFL
      121 LRFSTGGLAT GTFGLTGVSV WEAVVMEIVM TFGLVYTVYA TAVDPKKGSL GTIAPIAIGF
      181 IVGANILVGG AFTGASMNPA VSFGPALVSW EWGYQWVYWV GPLIGGGLAG VIYELLFISQ
```

```
      241 THEQLPTTDY
//
LOCUS       SbTIP1-2       258 aa
DEFINITION  SbTIP1-2     258 aa
TITLE       SbTIP1-2
ORIGIN

        1 MPVSRIAVGA PGELSHPDTA KAAVAEFISM LIFVFAGSGS GMAFSKLTTD GGASGTPCGL
       61 IAASLAHALA LFVAVSVGAN ISGGHVNPAV TFGAFVGGNM SLVKAVVYWV AQLLGSVVAC
      121 ILLKMATGGA AVGGFSLSAG VGAWNAVVLE MVMTFGLVYT VYATAVDPNK GDLGVIAPIA
      181 IGFIVGANIL AGGAFDGASM NPAVSFGPAV VSGVWENHWV YWLGPFAGAA IAALVYDIIF
      241 IGQQQRPTHH HHLPTTDY
//
LOCUS       SbTIP2-1       249 aa
DEFINITION  SbTIP2-1     249 aa
TITLE       SbTIP2-1
ORIGIN

        1 MVKLAFGSVG DSFSATSIKA YVSEFIATLL FVFAGVGSAI AFGKQINDRA LDPAGLVAIA
       61 IARALALFVC VSISLYISGG HLNPAVTFGL AVGGHITILT GIFYWVAQLL GASVACLLLK
      121 FVTHGKAIPT HGVSGISELE GVVFEIIITF ALVYTVYATA ADPKKGSLGT IAPIAIGFIV
      181 GANILAAGPF SGGSMNPARS FGPAVAAGNF AGNWVYWVGP LIGGGLAGLI YGDVFIGGNY
      241 QQVADQDYA
//
LOCUS       SbTIP2-2       249 aa
DEFINITION  SbTIP2-2     249 aa
TITLE       SbTIP2-2
ORIGIN

        1 MVKLAFGSLG DSFSAASLKS YVAEFIATLL FVFAGVGSAI AYSQLTKGGA LDPAGLVAIA
       61 IAHAFALFVG VSMAANVSGG HLNPAVTFGL AVGGHITILT GIFYWVAQVL GASVACLLLK
      121 YVTHGQAIPT HGVSGISEIE GVVMEIVITF ALVYTVYATA ADPKKGSLGT IAPIAIGFIV
      181 GANILAAGPF SGGSMNPARS FGPAVAAGNF AGNWVYWVGP LIGGGLAGLV YGDVFIASYQ
      241 PVGQQDQYP
//
LOCUS       SbTIP2-3       248 aa
DEFINITION  SbTIP2-3     248 aa
TITLE       SbTIP2-3
ORIGIN

        1 MSGNIAFGRF DDSFSAASLK AYVAEFISTL VFVFAGVGSA IAYTKLTGGA PLDPAGLIAV
       61 AVCHGFGLFV AVAIGANISG GHVNPAVTFG LALGGQITIL TGIFYWIAQL LGAIVGAVLV
      121 QYSTGVATPT HGLSGIGAFE GVVMEIIVTF GLVYTVYATA ADPKKGSLGT IAPIAIGFIV
      181 GANILVAGPF SGGSMNPARS FGPAVASGDF TNIWIYWVGP LVGGGLAGIV YRYIYMCGDH
      241 APVASSGF
//
LOCUS       SbTIP3-1       271 aa
DEFINITION  SbTIP3-1     271 aa
TITLE       SbTIP3-1
ORIGIN

        1 MNMIRSVRRR FTVGHMATAT DPATLRRAAA ELLATAIFVF AAEGATLSLG RMHRHDKGGG
       61 VVGGLVVVAL AHALALAAAV ACAANTSGGH VNPAVTFGAL LAGRICLVRS LVYWAAQLLG
      121 AVAAALVLRL ATGGMHLPEY ALAGCVSGWQ AAVLEAAMAF GLMHAYFVTV MDHHTRRVRA
```

```
      181 GAGAGAVAAP LAVGLLAGAN VLACGALEGA VMNPARAFGP AVVGSRRWGN HWVYWVGPMV
      241 GAGLSGVLYE HLVAGGEEAE PAPSCGGRRR E
//
LOCUS       SbTIP3-2       266 aa
DEFINITION  SbTIP3-2     266 aa
TITLE       SbTIP3-2
ORIGIN

        1 MSTGARPGRR FTVGRSEDAT HPDTIRAAIS EFIATAIFVF AAEGSVLSLG KMYYHDTAST
       61 ASGLVTVALA HALALAVAVA VAVNVSGGHV NPAVTFGALV GGRISLVRAV FYWVAQLLGA
      121 VAATLLLRLA TGGARPPGFA LASGVGDWHA VLLEAAMTFG LMYAYYATVV DPKRGHVGTI
      181 APLAVGFMLG ANVLAGGPFD GAGMNPARVF GPALVGWRWR HHWVYWLGPF LGAGLAGLVY
      241 EYLVIPSADA APPHSTHHQP LAPEDY
//
LOCUS       SbTIP3-3       268 aa
DEFINITION  SbTIP3-3     268 aa
TITLE       SbTIP3-3
ORIGIN

        1 MLPGRHPPRR SDTAGTRPLL PDATRAAVAE FVATAMFVFA AEGSVYGLWK LYKDTATPGG
       61 LLAVAIAHAL ALVAAVAVAS NASGGHVNPA VTFGLLVGRR ISFGRAAVYW LAQLLGAVVA
      121 SLLLTLVSGG TRPVGVGLVR GIHERHALLL EAVMTFGLMY AVYATAVDHR GRSGATTTIA
      181 IAPLAIGFVL GANILAGGPF DGAAMNPARA FGPALVGWSW RHHWVYWVGP LIGAGLAGAL
      241 YESVMVEQEP EAAPAAAPPR MPLASEDY
//
LOCUS       SbTIP4-1       314 aa
DEFINITION  SbTIP4-1     314 aa
TITLE       SbTIP4-1
ORIGIN

        1 MSHPPPPPVD QNISLRFCES FSLQGTETPG AFTPPAPAFP SRPRTGATTR LLLETFHSSM
       61 AKLVNKLVDS FEEQDTPDVG CVRAVLAELV LTFLFVFTGV SAAMAAGSGV KPGEAMPMAT
      121 LAAVAIAHAL AAGVLVTAGF HVSGGHLNPA VTVGLMVRGH ITKLRAVLYV AAQVLASSLA
      181 CILLRYLSGG MVTPVHALGA GISPMQGLVM EVILTFSLLF VTYAMILDPR SQVRTIGPLL
      241 TGLIVGANSL AGGNFSGASM NPARSFGPAL ASGVWTNHWI YWIGPLLGGP LAGFIYESLF
      301 IVNKTHEPLL NGDI
//
LOCUS       SbTIP4-2       318 aa
DEFINITION  SbTIP4-2     318 aa
TITLE       SbTIP4-2
ORIGIN

        1 MGKLTLGHRG EASEPDFFRG VLGELVLTFL FVFIGVGAAM TADGGTTSGS NAGGDLTAVA
       61 LGQALVVSVI ATAGFHISGG HVNPAVTLSL AVGGHITLFR SSLYIVAQMV GSSAACFLLR
      121 WLTGGLATPV HALAAGVGAV QGVVAETVFT FSLLLVIYAT ILDPRKLLPG AGPLLTGLLV
      181 GANSIAGAVL SGASMNPARS FGPAVATGVW THHWVYWVGP LAGGPLAVLV FEKASSAVVA
      241 VRRLHAPQDA SGHRRALHVV VRRAVRENLL EARAEGVVVA AGGPSLLTVA DAASAAVILF
      301 FPALLGCAAA KVEGVVDA
//
LOCUS       SbTIP4-3       252 aa
DEFINITION  SbTIP4-3     252 aa
TITLE       SbTIP4-3
ORIGIN
```

```
        1 MAKFALGHHR EVSDAGCVRA VLAELILTFL FVFAGVGSAM ATGKLAGGGG DTVVGLTAVA
       61 LAHTLVVAVM VSAGLHVSGG HINPAVTLGL AATGRITLFR SALYVAAQLL GSALACLLLA
      121 FLTAGGGGGV PVHALGDGVG ALRGVLMEVV LTFSLLFAVY ATVVDPRRAV GSMGPLLVGL
      181 VVGANVLAGG PFSGASMNPA RSFGPALVAG VWADHWVYWV GPLIGGLLAG LVYDGLFMAQ
      241 AGHEPLPRDD HF
//
LOCUS       SbTIP5-1       222 aa
DEFINITION  SbTIP5-1     222 aa
TITLE       SbTIP5-1
ORIGIN

        1 MLTTPDVTNS ASPLVATAVA QAFGLFAAVL IAADVSGGHV NPAVTFAYAI GGHIGVPSAI
       61 FYWASQLLGA TMACLSLNLF SAGEEVPTTR IAVAMTGFGG AVMEGVLTFL LVYTVHVVSE
      121 PRLSGGGGCG KRGFAATALG ALAVGLTEGA CVLAAGSLTG ASMNPARSFG AAVVSGRFKN
      181 QAVYWAGPMI GAAVAALVYQ IMACPSVAAA ESRHGNVEAV VV
//
LOCUS       SbTIP5-2       303 aa
DEFINITION  SbTIP5-2     303 aa
TITLE       SbTIP5-2
ORIGIN

        1 MASSTSNNLL VHLKHCFSAP SLRSYLAEFI STFLFVFTAA GSAISARTSL RFHFHAHQLM
       61 QVFYMDTVFQ ISCVATIIAR DADDAGAGPA APRPWWPRPS PRRSGSSPRY SSPPTSPAAM
      121 STPAVTVGFA IGGHIGVPSA IFYWASQLLG ATLACFSLNL FSAGEACRRN RSRRCRVPTT
      181 TIAVAMTGFG GAVLEGVLTF LLVYTVHVVG VSNGGKRGFA ATALGALAVG LTYGGRVRAR
      241 RRRADGRVHE PGALLRGHFK NQAVYWAGPM VGAAVAALVY QILACPDVAE SSSRHGNVEA
      301 VVV
//
LOCUS       SiNIP1-1       278 aa
DEFINITION  SiNIP1-1     278 aa
TITLE       SiNIP1-1
ORIGIN

        1 MAGGGDNSTN GARDQRAMEE GRKEEFATDQ GCAALSVPFI QKIIAEIFGT YFLIFAGCGA
       61 VTINASRNGQ ITFPGVAIVW GLAVMVMVYA VGHISGAHFN PAVTFAFATC GRFPWRQLPA
      121 YVLAQMLGAT LASGTLRLMF GGRHEHFPGT LPTGSDVQSL VLEIITTFYL MFVISGVATD
      181 NRAIGELAGL AVGATILLNV LIAGPVSGAS MNPARSVGPA LVSGQYRSIW VYVVGPVVGA
      241 VAGAWAYNLI RFTNKPLREI TKSTSFLKSM NRMNSASS
//
LOCUS       SiNIP1-2       281 aa
DEFINITION  SiNIP1-2     281 aa
TITLE       SiNIP1-2
ORIGIN

        1 MAGAELANGL HESTVAMEEG RGGGDEACRE SSEQDGAGSR PMFSVPFVQK IIAEIFGTYF
       61 LIFAGCAAVA VNLRTGGTVT FPGICIVWGL AVMVMVYSVG HISGAHLNPA VSVAFATCGR
      121 FPWRQVPAYA AAQVMGSTAA SLTLRLLFGN AREHFFGTVP AGSDVQSLVI ELIISFNLMF
      181 VVSGVATDNR AIGELAGLAI GATVLLNVLF AGPISGASMN PARTLGPAIV AGRYAGIWVY
      241 FAGPILGTVA GAWAYNLIRF TDKPLREITQ TSSFLRSARR N
//
LOCUS       SiNIP1-3       286 aa
DEFINITION  SiNIP1-3     286 aa
TITLE       SiNIP1-3
ORIGIN
```

```
        1 MARREDDSYT NGSVFEVSVE EGRKDKSEAY ADASKQPEEA NDGIDDAVCG MPASISYIQQ
       61 LIAEFLATFF LIFAGCGVIT VNDKNGMATF PGIAVVWGMT VMAMVYAVGH VSGAHINPAV
      121 TVGFAVSGRF PWRKVPAYMV VQTVAATFAS LLLRQMFGRR HLVASVTVPS GISSQSLVLE
      181 FIITFYLMFV IMAVATDDRA VGQMAGLAVG GTIMLNALFA GPVSGASMNP VRSIGPALVG
      241 GKYTGLWVYI FGPFAGAAAG AWAYNLIRHT DKTLAEITKS ISRTNN
//
LOCUS       SiNIP2-1       341 aa
DEFINITION  SiNIP2-1     341 aa
TITLE       SiNIP2-1
ORIGIN

        1 MTATMPLDQC KQSKRAVSFF FLLLLLLFPV GTHVCSRTHV ARSPEMSTNS RSNSRANFNN
       61 EIHDISTVQN STMPPMYYSD RSLADFFPPH LLKKVVSEVV STFLLVFVTC GAAAISASDL
      121 NRISQLGQSV AGGLIVTVMI YAVGHISGAH MNPAVTLAFA VFRHFPWIQV PFYWAAQFTG
      181 AICASFVLKA VLHPITVIGT TTPTGPHWHA LVIEVIVTFN MMFVTLAVAT DTRAVGELAG
      241 LAVGSAVCIT SIFAGAVSGG SMNPARTLGP ALASNLYTGL WIYFLGPVLG TLSGAWTYTY
      301 IRFEEAPSNK DAPQKLSSFK LRRLQSQSVA ADDDELDHIQ V
//
LOCUS       SiNIP2-2       297 aa
DEFINITION  SiNIP2-2     297 aa
TITLE       SiNIP2-2
ORIGIN

        1 MAASTAPSRT NSRVNYSNEI HDLSTVQSGG SAVPTMYYPE KSLADIFPPH LGKKVISEVV
       61 ATFLLVFVTC GAASIYGEDL KRISQLGQSV AGGLIVTVMI YATGHISGAH MNPAVTLSFA
      121 CFRHFPWIQV PFYWAAQFTG AMCAAFVLKA VLHPIEVIGT TTPTGPHWHA LVIEIVVTFN
      181 MMFVTCAVAT DSRAVGELAG LAVGSAVCIT SIFAGPVSGG SMNPARTLAP AVASNVYTGL
      241 WIYFLGPVIG TLSGAWVYTY IRFEEAPAKD APQRLSSFKL RRMQSQSALA ADEFDTV
//
LOCUS       SiNIP2-3       282 aa
DEFINITION  SiNIP2-3     282 aa
TITLE       SiNIP2-3
ORIGIN

        1 MAASTAPSRT NSRVNYSNEI HDLSTVQSGG SAVPTMYYPE KSLADIFPPH LGKKVISEVV
       61 ATFLLVFVTC GAASIYGEDL KRISQLGQSV AGGLIVTVMI YATGHISGAH MNPAVTLSFA
      121 CFRHFPWIQV PFYWAAQFTG AMCAAFVLKA VLHPIEVIGT TTPTGPHWHA LVIEIVVTFN
      181 MMFVTCAVAT DSRAVGELAG PVSGGSMNPA RTLAPAVASN VYTGLWIYFL GPVIGTLSGA
      241 WVYTYIRFEE APAKDAPQRL SSFKLRRMQS QSALAADEFD TV
//
LOCUS       SiNIP2-4       215 aa
DEFINITION  SiNIP2-4     215 aa
TITLE       SiNIP2-4
ORIGIN

        1 MAASTAPSRT NSRVNYSNEI HDLSTVQSGG SAVPTMYYPE KSLADIFPPH LGKKVISEVV
       61 ATFLLVFVTC GAASIYGEDL KRISQLGQSV AGGLIVTVMI YATGHISGAH MNPAVTLSFA
      121 CFRHFPWIQV PFYWAAQFTG AMCAAFVLKA VLHPIEVIGT TTPTGPHWHA LVIEIVVTFN
      181 MMFVTCAVAT DSRAVGELAG LAVGSAVCIT SIFAG
//
LOCUS       SiNIP3-1       299 aa
DEFINITION  SiNIP3-1     299 aa
TITLE       SiNIP3-1
```

```
ORIGIN

        1 MEPGSTPPNG SAPATPGTPA PLFSGGPRVD SLSYERKSMP RCRCLPAVEG WGLATHTCVV
       61 EIPAPDVSLT RKLGAEFMGT FILIFFATAA PIVNQKYGGV ISPFGNAACA GLAVTIIILS
      121 TGHISGAHLN PSLTIAFAAL RHFPWLQVPA YVSVQVLGSI CASFALKGVF HPFLSGGVTV
      181 PDVTISTAQA FFTEFIITFN LLFVVTAVAT DTRAVGELAG IAVGAAVTLN ILVAGPTTGG
      241 SMNPVRTLGP AVAAGNYRQL WIYLLAPTLG AVAGAGVYTA VKLRDENGET PRTQRSFRR
//
LOCUS       SiNIP3-2       222 aa
DEFINITION  SiNIP3-2     222 aa
TITLE       SiNIP3-2
ORIGIN

        1 MGTFILIFFA TAAPIVNQKY GGVISPFGNA ACAGLAVTII ILSTGHISGA HLNPSLTIAF
       61 AALRHFPWLQ VPAYVSVQVL GSICASFALK GVFHPFLSGG VTVPDVTIST AQAFFTEFII
      121 TFNLLFVVTA VATDTRAVGE LAGIAVGAAV TLNILVAGPT TGGSMNPVRT LGPAVAAGNY
      181 RQLWIYLLAP TLGAVAGAGV YTAVKLRDEN GETPRTQRSF RR
//
LOCUS       SiNIP3-3       296 aa
DEFINITION  SiNIP3-3     296 aa
TITLE       SiNIP3-3
ORIGIN

        1 MEEQKRGMGV AAVTVPPMLA SESNKISIII SPRAASSKVM PFELLNTGSV SSHPHADPAE
       61 SSDAHAAHYH RWNKGLPKIK AVPLIKKVVA EFLGTFILIF TVLSTIIMNE QHDGVESLLG
      121 IATSAGLAVT VLVLSLIHIS GCHLNPAVSI AMTVFGHLPL AHLLPYVTAQ ILGSIAASFS
      181 VKGIYHPVNP GIATIPKLGT TEAFFLEFIT TFVLLFIITA LATDPNAVKE LIAVAVGATI
      241 MMNALVAGPS TGASMNPART LGPAIATGRY TQIWIYMVAT PLGAVAGTGA YVAIKL
//
LOCUS       SiNIP3-4       220 aa
DEFINITION  SiNIP3-4     220 aa
TITLE       SiNIP3-4
ORIGIN

        1 MDPAAVTVPP VQSSESNKIS IIISPRAASS KVIAEFLGTF ILNITVLSTI IMNEQHDGVE
       61 SLLGIATSAG LAVTILVLSL IHISGCHLKP AVSIAMSAFG HLPLAHLLPY MTAQILGSIT
      121 ASFTVKGIYH PVNPGIATVP KFGTVKELIA VAVGAEVMMN ALVAGMPSTG ASMMNPARSI
      181 GPAITTGRYT QIWMYMVATP LGAIAGTGAC VAIKLYFLHS
//
LOCUS       SiNIP3-5       277 aa
DEFINITION  SiNIP3-5     277 aa
TITLE       SiNIP3-5
ORIGIN

        1 MDVSVSIPAA ASPMENMSDD KIAIVIPHRS PSNKILPLGF QLQQHEPSPH PPPTGFAERV
       61 ALPLIKKVAA ELLGTFLLVF TVLSALITDE AHGGALGLPG VAAAAGMAIV VLVSSLAHVS
      121 GGHMNPAVSA AMAAFGHLPR PHLAPYVAAQ LLGSTAASFA AKALYDPLNM GATVATVPTI
      181 GGAEAFAVEF ATTFVFLFVV TALATDPKAV KEMVAVGAGA AVVMSALVSG KWTGASMNPA
      241 RTLGPAIATG TYAKIWVYMV APPLGAIAGS GAYHALK
//
LOCUS       SiNIP3-6       286 aa
DEFINITION  SiNIP3-6     286 aa
TITLE       SiNIP3-6
ORIGIN
```

```
        1 MEHMRSISMD LTLSIPGAAS MENMSDDKIA IIVPQTSPIF SKSPSNKTLP QWVQNEASHP
       61 APISEKRAAL ALVKKVVAEF LGTFLLIFIL LSALIMNEEH GGALGLLGVA AVAGSAVLVI
      121 VASLVHVSGA HLNPAVSVAM AAFGYLPRAH LLPYVAAQLL GSTTASFAAK AVYNNPANLG
      181 ATVATVPAVG AGEALVVEFF TTFVLLFVIT ALSTDPKAVK ELIAVGAGAA VMMSALISGE
      241 STGASMNPAR TLGPAIAAGT YTKIWVYMVA PPLGAIAGTG AYIALE
//
LOCUS       SiNIP3-7       291 aa
DEFINITION  SiNIP3-7     291 aa
TITLE       SiNIP3-7
ORIGIN

        1 MADHTTMSSE DQRPVAISVC STPSDDRSLA GAGAGAVAST PRSSKLVPLD SLQKLMLKSP
       61 LAPVQDEPAP VPLVKKVAAE FIGTFILMFT VVSAIVADAH QHGGGGGAGG VLGIAAAAGL
      121 AVVAVVLAVV DVSGSHLNPA VSIAMAAFGH LPRAHVAPYA AAQTLGSAAA TFLAKGMYRP
      181 ADPGVMATVP RGGAVAEAFF VELALTFVLV FVIAAVATDP ASSKEAVAVS IAAAITMNAL
      241 VGASWTGPSM NPARTIGAAV ATGKYKDIWV YLVAPPLGAI AGAGTYTLIK P
//
LOCUS       SiNIP4-1       210 aa
DEFINITION  SiNIP4-1     210 aa
TITLE       SiNIP4-1
ORIGIN

        1 MAADHVGENV TGSDGDQRSK VNGQDLEQHP RGDQEPAADH VSRGLAVGHF IRELMVEGMA
       61 SFLLVFWSGV AALMQEMHGT LSFPMVCLVV ALTVGFVLCW LGPAHFNPAV TATFAAFGYL
      121 SWAKLPFYVM VQLAGSVLAC LSVNGVMRPR EEHFYGTAPM PGHTRLPFLL ELLASAVLMI
      181 VIATAARGSN PTAGGLAIGA AVGTLGLIIG
//
LOCUS       SiPIP1-1       269 aa
DEFINITION  SiPIP1-1     269 aa
TITLE       SiPIP1-1
ORIGIN

        1 MEGKEEDVRL GANKYSERQP IGTAAQGSDD KDYKEPPPAP LFEPGELKSW SFYRAGIAEF
       61 VATFLFLYIS ILTVMGVSKS QSKCATVGIQ GIAWSFGGMI FALVYCTAGI SGGHINPAVT
      121 FGLFLARKLS LTRAVFYMIM QCLGAICGAG VVKGFQQGLY MGNGGGANMV AAGYTKGDGL
      181 GAEIVGTFIL VYTVFSATDA KRNARDSHVP ILAPLPIGFA VFLVHLATIP ITGTGINPAR
      241 SLGAAVIYNR RQAWDDHVSS LTENWCNLV
//
LOCUS       SiPIP1-2       215 aa
DEFINITION  SiPIP1-2     215 aa
TITLE       SiPIP1-2
ORIGIN

        1 MEGKEEDVRL GANKYSERQP IGTAAQGSDD KDYKEPPPAP LFEPGELKSW SFYRAGIAEF
       61 VATFLFLYIS ILTVMGVSKS QSKCATVGIQ GIAWSFGGMI FALVYCTAGI SGGHINPAVT
      121 FGLFLARKLS LTRAVFYMIM QCLGAICGAG VVKGFQQGLY MGNGGGANMV AAGYTKGDGL
      181 GAEIVGTFIL VYTVFSATDA KRNARDSHVP VSVHI
//
LOCUS       SiPIP1-3       288 aa
DEFINITION  SiPIP1-3     288 aa
TITLE       SiPIP1-3
ORIGIN
```

```
        1 MEGKEEDVRL GANKYSERQP IGTAAQGSDD KDYKEPPPAP LFEPGELKSW SFYRAGIAEF
       61 VATFLFLYIS ILTVMGVSKS QSKCATVGIQ GIAWSFGGMI FALVYCTAGI SGGHINPAVT
      121 FGLFLARKLS LTRAVFYMIM QCLGAICGAG VVKGFQQGLY MGNGGGANMV AAGYTKGDGL
      181 GAEIVGTFIL VYTVFSATDA KRNARDSHVP ILAPLPIGFA VFLVHLATIP ITGTGINPAR
      241 SLGAAVIYNR RQAWDDHWIF WVGPFIGAAL AAIYHQVVIR AIPFKSRS
//
LOCUS       SiPIP1-4       288 aa
DEFINITION  SiPIP1-4     288 aa
TITLE       SiPIP1-4
ORIGIN

        1 MEGKEEDVRL GANRYSERQP IGTAAQGSDD KDYKEPPPAP LFEAEELTSW SFYRAGIAEF
       61 VATFLFLYIS ILTVMGVSKS PSKCATVGIQ GIAWSFGGMI FALVYCTAGI SGGHINPAVT
      121 FGLFLARKLS LTRALFYMVM QCLGAICGAG VVKGFQQSLY MGNGGGANAV NPGYTKGDGL
      181 GAEIVGTFVL VYTVFSATDA KRSARDSHVP ILAPLPIGFA VFLVHLATIP ITGTGINPAR
      241 SLGAAIVYNR SQAWNDHWIF WVGPFIGAAL AAIYHVVIIR AIPFKSRD
//
LOCUS       SiPIP1-5       337 aa
DEFINITION  SiPIP1-5     337 aa
TITLE       SiPIP1-5
ORIGIN

        1 MEGKEEDVRL GANKFSERQP IGTAAQGAGD DKDYKEPPPA PLFEPGELKS WSFYRAGIAE
       61 FVATFLFLYI TILTVMGVSK SNSKCATVGI QGIAWSFGGM IFALVYCTAG ISGGHINPAV
      121 TFGLFLARKL SLTRAIFYII MQCLGAICGA GVVKGFQQGL YMGNGGGANV VAPGYTKGSG
      181 LGAEIVGTFV LVYTVFSATD AKRNARDSHV PILAPLPIGF AVFLVHLATI PITGTGINPA
      241 RSLGAAIIYN REHAWSHHVM DLLGRPLHRR CPGCYLPPGD HQGHPVQEQV LSCDGPARRD
      301 MPARRKERAS SSLMSCVPTP SISVDSSPLF TTAALFC
//
LOCUS       SiPIP1-6       289 aa
DEFINITION  SiPIP1-6     289 aa
TITLE       SiPIP1-6
ORIGIN

        1 MEGKEEDVRL GANKFSERQP IGTAAQGAGD DKDYKEPPPA PLFEPGELKS WSFYRAGIAE
       61 FVATFLFLYI TILTVMGVSK SNSKCATVGI QGIAWSFGGM IFALVYCTAG ISGGHINPAV
      121 TFGLFLARKL SLTRAIFYII MQCLGAICGA GVVKGFQQGL YMGNGGGANV VAPGYTKGSG
      181 LGAEIVGTFV LVYTVFSATD AKRNARDSHV PILAPLPIGF AVFLVHLATI PITGTGINPA
      241 RSLGAAIIYN REHAWSHHWI FWVGPFIGAA LAAIYHQVII RAIPFKSRS
//
LOCUS       SiPIP1-7       299 aa
DEFINITION  SiPIP1-7     299 aa
TITLE       SiPIP1-7
ORIGIN

        1 MAGGKLQDRF QDDEDVRVGV DRFPERHPIG ATAADDLGRD YTEPPPAPLF DAAELSSWSF
       61 YRAGIAEFVA TFLFLYVTVL AVMGVSNSPS KCGTVGVQGI AWAFGGMIFA LVYCTAGVSG
      121 GHINPAVTFG LLLARKLSLP RAGYYAVMQC LGAACGAGVV KALVGGALYE AAGGGANAVN
      181 PGYTKGDGLG AEIVGTFVLV YTVFSATDAK RSARDSHVPV LAPLPIGLAV FLVHLATIPI
      241 TGTGINPARS LGAAIIYDRP HGWHGHWIFW VGPFTGAALA AVYHQVVIRA IPFKSSAHY
//
LOCUS       SiPIP2-1       289 aa
DEFINITION  SiPIP2-1     289 aa
TITLE       SiPIP2-1
```

```
ORIGIN

        1 MGKDDVIESG AGGGEFAAKD YTDPPPAPLI DAAELGSWSL YRAVIAEFIA TLLFLYITVL
       61 TVIGYKHQTD PNVAGTDACG GVGILGIAWA FGGMIFVLVY CTAGISGGHI NPAVTFGLFL
      121 ARKVSLVRAL LYIVAQCLGA ICGVGLVKAF QSAYFDRYGG GANSLASGYS RGTGLGAEII
      181 GTFVLVYTVF SATDPKRNAR DSHVPVLAPL PIGFAVFMVH LATIPVTGTG INPARSLGAA
      241 VIYNKDKPWD DHWIFWVGPF AGAAIAAFYH QYILRAGAIK ALGSFRSNA
//
LOCUS       SiPIP2-2       266 aa
DEFINITION  SiPIP2-2     266 aa
TITLE       SiPIP2-2
ORIGIN

        1 MGKDDVIESG AGGGEFAAKD YTDPPPAPLI DAAELGSWSL YRAVIAEFIA TLLFLYITVL
       61 TVIGYKHQTD PNVAGTDACG GVGILGIAWA FGGMIFVLVY CTAGISGGHI NPAVTFGLFL
      121 ARKVSLVRAL LYIVAQCLGA ICGVGLVKAF QSAYFDRYGG GANSLASGYS RGTGLGAEII
      181 GTFVLVYTVF SATDPKRNAR DSHVPVLAPL PIGFAVFMVH LATIPVTGTG INPARSLGAA
      241 VIYNKDKPWD DHVRYVSTHP VPSPFT
//
LOCUS       SiPIP2-3       290 aa
DEFINITION  SiPIP2-3     290 aa
TITLE       SiPIP2-3
ORIGIN

        1 MAKDIEAAAA PEGGEYTAKD YSDPPPAPLI DAEELTKWSL YRAVIAEFVA TLLFLYITVA
       61 TVIGYKHQTD AAASGPDAAC GGVGILGIAW AFGGMIFILV YCTAGVSGGH INPAVTLGLF
      121 LARKVSLVRA LLYIVAQCLG AICGVGLVKG FQSAYYVRYG GGANELSDGY SKGTGLAAEI
      181 IGTFVLVYTV FSATDPKRNA RDSHVPVLAP LPIGFAVFMV HLATIPITGT GINPARSLGA
      241 AVIYNNDKAW DDHWIFWVGP FIGAAIAAAY HQYVLRASAS KLGSSASFSR
//
LOCUS       SiPIP2-4       288 aa
DEFINITION  SiPIP2-4     288 aa
TITLE       SiPIP2-4
ORIGIN

        1 MAKDIEASGP EAGEFSAKDY SDPPPAPLID AEELTKWSLY RAAIAEFVAT LLFLYITVAT
       61 VIGYKHQTDA AASGPDAACG GVGILGIAWA FGGMIFILVY CTAGISGGHI NPAVTFGLFL
      121 ARKVSLVRAV LYIIAQCLGA ICGVGLVKGF QSAYFVRYGG GANELSAGYS KGTGLAAEII
      181 GTFVLVYTVF SATDPKRSAR DSHVPVLAPL PIGFAVFMVH LATIPITGTG INPARSLGAA
      241 VIYNNDKAWD DQWIFWVGPL IGAAIAAAYH QYVLRASAAK LGSFRSNA
//
LOCUS       SiPIP2-5       286 aa
DEFINITION  SiPIP2-5     286 aa
TITLE       SiPIP2-5
ORIGIN

        1 MGKEVDVSTL EAGGARDYAD PPPAPLVDID ELGKWSLYRA VIAEFVATLL FLYITVATVI
       61 GYKHQTDASA SGPDAACGGV GILGIAWAFG GMIFILVYCT AGISGGHINP AVTFGLFLAR
      121 KVSLVRAILY MAAQCLGAIC GVALVKGFQS GFYVRYGGGA NEVSTGYSTG TGLAAEIVGT
      181 FVLVYTVFSA TDPKRNARDS HIPVLAPLPI GFAVFMVHLA TIPITGTGIN PARSLGAAVV
      241 YNNNKAWSDQ WIFWVGPFIG AAIAALYHQI VLRASARGYG SFRSNA
//
LOCUS       SiPIP2-6       286 aa
DEFINITION  SiPIP2-6     286 aa
```

```
TITLE       SiPIP2-6
ORIGIN

        1 MGKEVDVSAL EAGGARDYVD PPPAPLVDID ELGKWSLYRA VIAEFVATLL FLYITVATVI
       61 GYKHQTDASA SGPDAACGGV GILGIAWAFG GMIFILVYCT AGISGGHINP AVTFGLFLAR
      121 KVSLVRAVLY MAAQCLGAIC GVALVKGFQS GFYARYGGGA NEVSAGYSTG TGLAAEIIGT
      181 FVLVYTVFSA TDPKRNARDS HVPVLAPLPI GFAVFMVHLA TIPITGTGIN PARSLGAAVV
      241 YNNNKAWSDQ WIFWVGPFIG AAIAALYHQI VLRASARGYG SFRSNS
//
LOCUS       SiPIP2-7       284 aa
DEFINITION  SiPIP2-7     284 aa
TITLE       SiPIP2-7
ORIGIN

        1 MNPSESMEAA GGKKDYKDPA PAPLVNAGEL GKWSLYRAVI TEFVATLLFV YVTLATVIGH
       61 KRQSESQPCG GAGVLGIAWS FGGMIFVLVY CTAGVSGGHV NPAVTFGLLL ARKVSLVRAA
      121 LYIVAQCLGA ICGAGLVRAF HGTSSYLRHG GGANELAAGY SKGAGLAAEI VGTFVLVYTV
      181 FSATDPKRKV RDTHVPVLAP LPIGFAVFMV HLATIPITGT GINPARSLGP AVVYNQRKAW
      241 EDHWMFWVGP LIGSAAAMVY HQLVLRAGAA KAFASWRNNN HTGI
//
LOCUS       SiPIP2-8       294 aa
DEFINITION  SiPIP2-8     294 aa
TITLE       SiPIP2-8
ORIGIN

        1 MAVGHEIVQQ QRQRDPEHGG GGESSGKDYT DPPPQPVLTA SELRRWSLYR AAIAEFVATL
       61 LFLYLTVATV IGYKRQAESD ASGCGGVGVL GIAWAFGGMI FLLVYCAAGI SGGHINPAVT
      121 LALLQARKVS VPRAALYVAA QCLGAVCGAG LVRAIHSPDA FVRLGGGANA VGDGYGRGTG
      181 LAAEVVGTFV LVYTVFSATD AKRNARDSHI PVLAPLPIGF AVFVVHLATI PITGTGINPA
      241 RSFGAAVVYN QARAWQDQWI FWVGPLTGAA MATLYHEHVL RASAIKALGS FKAG
//
LOCUS       SiPIP2-9       285 aa
DEFINITION  SiPIP2-9     285 aa
TITLE       SiPIP2-9
ORIGIN

        1 MPIEDVSIET TEAAGPQKVP YWDPPPAPLL ETSELMKWSL YRALIAEFVA TLIFLYVSIA
       61 TVIGYKDQSK ALACNGVGFL GVAWSFGATI FILVYCIGGI SGGHINPAVT FGLFVGRKLS
      121 LLRTVLYIVA QCLGAICGVA IVKGITGDQY SLLGGGANSV ADGFSVVAGL GAEIMGTFVL
      181 VYTVFSATDP KRTARDSFIP VLVPLPIGFA VFVVHLATIP ITGTGINPAR SLGAAVIFGE
      241 AWKNHWIFWV GPLIGATAAA LYHKLVLRGE AAKALGSFRS TSATV
//
LOCUS       SiSIP1-1       200 aa
DEFINITION  SiSIP1-1     200 aa
TITLE       SiSIP1-1
ORIGIN

        1 MAMGAAAARG AAADAVVTFL WVLCASALGA TTAAVTSLLG VAQEEGGGGH YALLVTASLL
       61 AALLFAFDLL CGALGGASFN PTDFAASYAA GLDSPSLFSV ALRFPAQAAG AVGGALAISE
      121 LMPEQYKHTL AAAGPALKVD PHTGAVAEGV LTFVITLAVL WIIVKGPRNP VLKTMLLSVS
      181 IVSLILAGAE YTGPSMNPAN
//
LOCUS       SiSIP1-2       243 aa
DEFINITION  SiSIP1-2     243 aa
```

```
TITLE       SiSIP1-2
ORIGIN

        1 MAIGAALRAA AADAVVTFLW VLCVSTLGAS TAAVTSYLKL QGVQYALLIT VSLVSVLLFV
       61 FNILCDALGG ASFNPTGIAA FYAAGVTSPS LFAVALRFPA QAAGAVCGAL AISELMPAQY
      121 KHMLGGPSLK VDPHTGAVAE LVLTFVITMA VLWIIVKGPR NPIVKTLMIS ISTVCLVLSG
      181 AAYTGPSMNP ANAFGWAYVN NRHNTWEQFY VYWISPFIGA VLAAWIFKAL FLAPPPKPKA
      241 KKA
//
LOCUS       SiSIP2-1       252 aa
DEFINITION  SiSIP2-1     252 aa
TITLE       SiSIP2-1
ORIGIN

        1 MSPAPPPPSR ARIRPWLVAG DLALAAAWVC AGALVKLLVY GPLGFGGRPE AEAVKVSLSL
       61 VYMFIFAWLE AATGGASYNP LTVLAAAVAS HGGPAVYLFT AFVRIPAQVV GAVLGVKLIQ
      121 FTFPNVGKGA RLSVGAHHGA LAEGLATFMV VMVSVTLKKK KMKSFFMKTW ITSIWKNTIH
      181 ILSSDITGGI MNPASAFAWA YARGDHTTFD HLLVYWLAPL QATLLGVWVV TFLTKPKKIK
      241 EQEADENKTK KE
//
LOCUS       SiTIP1-1       249 aa
DEFINITION  SiTIP1-1     249 aa
TITLE       SiTIP1-1
ORIGIN

        1 MPINRIAVGS HEEVYHPGAL KAAFAEFIST LIFVFAGQGS GMAFSKLSPG GSTPTGLIAA
       61 AIAHAFALFV AVSVGANISG GHVNPAVTFG AFVGGNITLF RGILYWIAQL LGSTVACFLL
      121 RFSTGGLPTG TFGLTGISVW EALVLEIVMT FGLVYTVYAT AVDPKKGSLG TIAPIAIGFI
      181 VGANILVGGA FDGASMNPAV SFGPALVSWS WGYQWVYWVG PLIGGGLAGV IYEVLFISHT
      241 HEQLPTTDY
//
LOCUS       SiTIP1-2       268 aa
DEFINITION  SiTIP1-2     268 aa
TITLE       SiTIP1-2
ORIGIN

        1 MPVSRISVGA PGELSHPDTA KAAVAEFISM LIFVFAGSGS GMAFSKQSSF SSSSSSSWCA
       61 GKLTAGGATT PSGLIAAALA HALALFVAVA VGANISGGHV NPAVTFGAFV GGNITLLKAV
      121 VYWVAQLLGS VVACLLLKIA TGGEAVGAFS LSAGVGAWNA VVFEIVMTFG LVYTVYATAV
      181 DPKKGDLGVI APIAIGFIVG ANILAGGAFD GASMNPAVSF GPAVVSGVWE NHWVYWLGPF
      241 VGAAIAALVY DIIFIGQRPH DHLPTTDY
//
LOCUS       SiTIP2-1       243 aa
DEFINITION  SiTIP2-1     243 aa
TITLE       SiTIP2-1
ORIGIN

        1 MAYVAEFIAT LLFVFAGVGS AITYGQLSHG GALDASGLVA IAFTHALALF VGVSVAANIS
       61 VGHLNPAVTF GLAVGGHITI LTGLFYWVAQ LLGASVACLL LKFVTHGKAI PTHGVAGISE
      121 LEGVVFEIII TFALLYTVYA TAADPKKGSL GTIAPISIGF IVGANILAAG PFSGGSVNPA
      181 RSFDPAVAAG NFAGNWVYWV GPLIGGGLAG LIYGDVFIGG NYQQVADHRL AHPRRRIWEE
      241 RNG
//
LOCUS       SiTIP2-2       249 aa
```

```
DEFINITION  SiTIP2-2     249 aa
TITLE       SiTIP2-2
ORIGIN

        1 MVKLAFGSVG DSFSTTSIKA YVAEFIATLL FVFAGVGSAI AYGQLSHGGA LDASGLVAIA
       61 IAHALALFVG VSVAANISGG HLNPAVTFGL AVGGHITILT GLFYWVAQLL GASVACLLLK
      121 FVTHGKAIPT HGVAGISELE GVVFEIIITF ALVYTVYATA ADPKKGSLGT IAPIAIGFIV
      181 GANILAAGPF SGGSMNPARS FGPAVAAGNF AGNWVYWVGP LIGGGLAGLI YGDVFIGGNY
      241 QQVADQDYA
//
LOCUS       SiTIP2-3       248 aa
DEFINITION  SiTIP2-3     248 aa
TITLE       SiTIP2-3
ORIGIN

        1 MVKLAFGSFG DSFSAASLKA YVAEFIATLL FVFAGVGSAI AYSQLTKGGA LDPAGLVAIA
       61 IAHAFALFVG VSMAANISGG HLNPAVTFGL AVGGHITILT GIFYWVAQLL GASVACLLLK
      121 FVTHGQAIPT HGVSGISEVE GVVMEIVITF ALVYTVYATA ADPKKGSLGT IAPMAIGFIV
      181 GANILAAGPF SGGSMNPARS FGPAVAAGNF AGNWVYWVGP LIGGGLAGLV YGDVFIASYQ
      241 PVGQQEYP
//
LOCUS       SiTIP2-4       252 aa
DEFINITION  SiTIP2-4     252 aa
TITLE       SiTIP2-4
ORIGIN

        1 MSGNIAFGRF DDSFSAASLK AYVAEFISTL VFVFAGVGSA IAYTKLSGGA PLDAAGLVAV
       61 AVCHGFGLFV AVAIGANISG GHVNPAVTFG LALGGQITIL TGLFYWIAQL LGAIVGAVLV
      121 QYSTGVVRQQ ATPTHGLSGI GALEGVVMEI IVTFGLVYTV YATAADPKKG SLGTIAPIAI
      181 GFIVGANILV AGPFSGGSMN PARSFGPAVA SGDFTNIWIY WVGPLVGGGL AGIVYRYIYM
      241 CGDHAPVASS DF
//
LOCUS       SiTIP2-5       248 aa
DEFINITION  SiTIP2-5     248 aa
TITLE       SiTIP2-5
ORIGIN

        1 MSGNIAFGRF DDSFSAASLK AYVAEFISTL VFVFAGVGSA IAYTKLSGGA PLDAAGLVAV
       61 AVCHGFGLFV AVAIGANISG GHVNPAVTFG LALGGQITIL TGLFYWIAQL LGAIVGAVLV
      121 QYSTGVATPT HGLSGIGALE GVVMEIIVTF GLVYTVYATA ADPKKGSLGT IAPIAIGFIV
      181 GANILVAGPF SGGSMNPARS FGPAVASGDF TNIWIYWVGP LVGGGLAGIV YRYIYMCGDH
      241 APVASSDF
//
LOCUS       SiTIP3-1       262 aa
DEFINITION  SiTIP3-1     262 aa
TITLE       SiTIP3-1
ORIGIN

        1 MSTGARPGRR FTVGRSEDAT HPDTIRAAIS EFLATAIFVF AAEGSVLSLG KMYHDMSTAG
       61 GLVAVALAHA LALSVAVAVA VNISGGHVNP AITFGALIGG RISLVRAVFY WVAQLLGAIA
      121 ASLLLRLATG GMRPPGFALA SGVGDWHAVL LEAAMTFGLM YAYYATVIDP KRGSVGTIGP
      181 LAVGFLLGAN VLAGGPFDGA GMNPARVFGP ALVGWRWRHH WVYWLGPFLG AGVAGLVYEY
      241 LVIPSADAAA PHAHQPLAPE DY
//
```

```
LOCUS       SiTIP3-2       257 aa
DEFINITION  SiTIP3-2     257 aa
TITLE       SiTIP3-2
ORIGIN

        1 MRAGRRRFNV GRLATATDPG TLRDAAAELL ATAVFVFAAE GATLSFGRDK SGGLVAVALA
       61 HALALAAAVA CTLNISGGHV NPAITFGAFL GGRICLVRSL VYWAAQLIGA VTAALLLRLA
      121 TGGVRLPEYA LAGGVSGWHA VVLEAAMAFG LMYAYCATAM EPRRGRAAGA VAPLAVGLLA
      181 GANVLACGAL DGAVMNPARA FGPAIVGSRR WSNHWVYWAG PMVGAGLSGF FYEHLVVTPA
      241 DEEEPAAAPS RGSSRRA
//
LOCUS       SiTIP3-3       242 aa
DEFINITION  SiTIP3-3     242 aa
TITLE       SiTIP3-3
ORIGIN

        1 YRADAAGTGP LLPAATRAAV AEFVATGIFV FAAEGSVYGL SVQGHGDAGR PLVAVAIAHA
       61 LALAAAVAVA SNASDGHVNP AVTFGVLVGR RISFGRAVVY YWAAKLLGAV AAAFLLMLVS
      121 GGTRPMGFGL GQGVHERHAL LLEAVMTFGL VYAVYATAVD HRSRVGFVLG ANILAGGPFD
      181 GAAMNPALVG WSWRHHWVYW VGPLIGAGLA GALYEFVMVE QPPSEAPAAA AGPRMLVAAA
      241 ED
//
LOCUS       SiTIP4-1       377 aa
DEFINITION  SiTIP4-1     377 aa
TITLE       SiTIP4-1
ORIGIN

        1 MPTWTNGRWT GRTSGMMCSR EKRMLGAKHK GVAGKQRRAF TELGRQVDKP ELQSPRRACP
       61 TALEIEISPC TFPCAFPSGQ QENLSLLPPP LSPPPRPSSP PCQEQSEEAP RNPSVRPAMA
      121 TKLMQKFVDS CDDGGDAGQQ DAGCVRAVLA ELVLTFLFVF TVVSAAMAAG SGVKAGEAMP
      181 MAALAAVAIT NALAAGVLVT AGFHVSGGHL NPAVTVAMMV RGHLSKLRTV LYVAAQLLAS
      241 SLACILLRYL TGGMVTPVHA LGAGIRPMQG LVMEVILTFS LLFVTYAMIL DPRSQVRTIG
      301 PLLTGLIVGA NSLAGGNFTG ASMNPARSFG PALATGDWTH HWVYWLGPLL GGSLAAVVYE
      361 SLFVVNKTHE PLLNGDC
//
LOCUS       SiTIP4-2       246 aa
DEFINITION  SiTIP4-2     246 aa
TITLE       SiTIP4-2
ORIGIN

        1 MGKLALGRRG EASEPDFFRG VLGELVLTFL FVFIQVGAAM TIDGKTNAGG NLTAVALGQA
       61 LAVCVIATAG FHISGGHVNP AVTLSMAVGG QITLFRSALY IVAQMLGSST ACVLLRWLTG
      121 GLVATPVHAL AAGVGPIQGV VAEVVFTFSL LFVIYATILD PRKILPGVGP LLTGLLVGAN
      181 SIAGAVLSGA SMNPARSFGP ALATGVWTHH WVYWVGPLAG GPLAVVVYEC LFMAPAGAHQ
      241 LLPEEA
//
LOCUS       SiTIP4-3       246 aa
DEFINITION  SiTIP4-3     246 aa
TITLE       SiTIP4-3
ORIGIN

        1 MGKVALGHRG EASEPDFFRG VLGELVLTFL FVFIQVGAAM TAGAKTDAGG DLTAVALGQA
       61 LVVCVIATAG FHISGGHVNP AVTLSLAVGG QITLFRSALY IVAQMLGSST ACILLRWLTG
      121 GLATPVHALA AGVGPIQGVV AEAVFTFSLL FVIYATILDP RKLLPGAGPL LTGLLVGANS
```

```
      181 IAGAVLSGAS MNPARSFGPA VATGVWTHHW VYWVGPLAGG PLAVVVYECF FMAPARTHQL
      241 LPQEAP
//
LOCUS       SiTIP4-4       250 aa
DEFINITION  SiTIP4-4     250 aa
TITLE       SiTIP4-4
ORIGIN

        1 MAKFALGHHR EAADAGCVRA VLAELILTFL FVFAGVGSAM ATGKLGGGAD SVAGLTAVAL
       61 AHTLVVAVMI SAGLHVSGGH INPAVTLGLA VTGRITLFRS ALYVVAQLLG SVAACLLLAF
      121 LAGGAATPVH ALAAGVGTLQ GVLMEVVLTF SLLFAVYATV VDPRRTVGGM GPLLVGLVVG
      181 ANVLAGGPFS GASMNPARSF GPALAAGVWA DHWVYWVGPL IGGPLAGLVY DGLFMAQGGH
      241 EPLPRDENDF
//
LOCUS       SiTIP5-1       259 aa
DEFINITION  SiTIP5-1     259 aa
TITLE       SiTIP5-1
ORIGIN

        1 MASSLLAKLK RCVSPPSLRS YFAEFISTFL FVFAAVGSAI SARMVTALDG GTASDAASLV
       61 ATAVAQAFGL FAAVLIAADV SGGHANPAVT FAFAIGGHIG VPSAIFYWAS QMLGSTFACL
      121 AVPTTRIAVA MTGFGAAIIE GVLTFMLVYT VHVAGDLRAA AGGKRGFADT ALGALAVGLV
      181 AGALVLSAGP LTGASMNPAR SFGPAVVSGN YKNQAVYWAG PMIGAAVAAL AHQILAGAPD
      241 AAAAGSSSSC HGNVETVVV
//
LOCUS       SiTIP5-2       259 aa
DEFINITION  SiTIP5-2     259 aa
TITLE       SiTIP5-2
ORIGIN

        1 MASCVVGCLQ QCFSPSALRS YLAEFISTFL FVFATAGSAI SARMLTPDST SSDASSLVAT
       61 AVAQAFALFV AVFIAADASG GHANPAVTFA FAICGHIAVL PAVLYCAAQL LGATFACLVV
      121 HILSAGQAVP TTRIAVDMTG FGASVLEAAA TFMVVYTVHA ACDPRRVRAG GGRSAAETAT
      181 GSLAIGLVTG ACALATGSLT GASMNPARSF GPAVVSGDFR NQAVYWAGPM VGAALAAIVH
      241 QHVMYPASLR PGSVETVVV
//
LOCUS       SlNIP1-1       290 aa
DEFINITION  SlNIP1-1     290 aa
TITLE       SlNIP1-1
ORIGIN

        1 MGDQQIGGGA NGSISLNIRD ADDNLNNKNC ANSVSHQDSS SNSTCSFVTV PFIQKIIAET
       61 LGTYFLIFAG CGSVAVNADK GMVTFPGISI VWGLVVMVMV YSVGHISGAH FNPAVTIAFA
      121 SNKRFPWKQV PAYVAAQVIG STLASGTLRL IFNGKHDHFV GTSPTGSDVQ SLVLEFIITF
      181 YLMFVISGVA TDNRAIGELA GLAVGATVLL NVMFAGPISG ASMNPARSLG PAIVSSHYKG
      241 LWVYMLGPIG GAIAGAWVYN IIRFTDKPLR EITKSGSFLK SKTLLRNPSH
//
LOCUS       SlNIP1-2       277 aa
DEFINITION  SlNIP1-2     277 aa
TITLE       SlNIP1-2
ORIGIN

        1 MADHQINVNG NINHGVSLNI KEDHDLNNHK ESSSTSSFLT VPFIQKVIAE MIGTYFLIFA
       61 GCGSVVVNAD KGMITFPGVA ITWGLVVMVM VYSVGHISGA HFNPSVTIAF ASVKRFPWKQ
```

```
      121 VPAYVAAQVL GATLASGTLR LIFNGKHDHF AGTLPSGTDF QSFVIEFIIT FYLMFVISGV
      181 ATDNRAIGEL AGLAVGATIL LNVMFTGPIS GASMNPARSL GPAIVSSHYK GLWIYLVSPT
      241 LGAIAGAWVY NIIRFTDKPL REITKSGSFL KSKNSST
//
LOCUS       SlNIP1-3       267 aa
DEFINITION  SlNIP1-3     267 aa
TITLE       SlNIP1-3
ORIGIN

        1 MVSNKEDQIT QNMEEGNVQS ASNNKVGFCS SPAVVVLGQK LIAEVIGTYF VIFAGCGSVV
       61 VNKLYGGTIT FPGISVTWGL IVMVMVYTVG HISGAHFNPA VTITFSVFGR FPWKEVPFYI
      121 VAQLMGSILA SGTLSLMFDV TPEAYFGTVP VGSNVQSLAA EIVISFLLMF VISGVGTDER
      181 AIGHIAGIAV GMTITLNVFV VGPISGASMN PARSIGPAIV RHTYKGLWVY IVGPIVGTLA
      241 GAFMYNLIRA TDKPLNELTK SVSSLRS
//
LOCUS       SlNIP1-4       272 aa
DEFINITION  SlNIP1-4     272 aa
TITLE       SlNIP1-4
ORIGIN

        1 MSTKDIREIE EGNCSNYTNN VSGDDSSLCT SPEVVIIIQK VIAEAIGTYF LIFVGCGAVA
       61 VDKTYGSVTF PGICVAWGLI VMVMVYSVGH ISGAHFNPAV TIAFALFRHF PVKQVPLYIM
      121 AQMVGAILGS GTLYLLLDLK TQAFFGTTPV GTNLQSLILE FIISYLLMFV ISGVATDNRS
      181 IGELAGIAIG MTILLNVLIA GPVSGASMNP ARSIGPAIVM HHYKGLWVYI IGPILGTICG
      241 AFTYNLIRFT EKPLRELTLT KTSTFLKSMS RK
//
LOCUS       SlNIP1-5       231 aa
DEFINITION  SlNIP1-5     231 aa
TITLE       SlNIP1-5
ORIGIN

        1 MSNLVFSMQL VAELLGTYLS MFAGFAAMVI NKKIMMVMIY TVGPVFGAHF NPAVTVAFAS
       61 CKRVAWRNVP AYMLAQVVGA TLATVTVRLM FKEEQLQFLR TILAGTAMDK QAVSDHCLFR
      121 CKDPRTEFDL FYPSLCLLMQ VGELNGHVIG AVITINSILA GPISGGSINP TRSLGPAILS
      181 NCYKKQWIYI LGPTAGATTG IWFYNAMKSV KSYNEVTKFL PFLRRLAQNK V
//
LOCUS       SlNIP1-6       345 aa
DEFINITION  SlNIP1-6     345 aa
TITLE       SlNIP1-6
ORIGIN

        1 MEEISEGIRA TSLRINDCPS PLPSAIASSA TPQKHLKCFI SVHFVQKLIA EFVGTYMLIF
       61 AGCAAIVLNI NKNNVVTLPG IASVWGLVVM VLIYSVGHVS GAHFNPAVTI AFATSKMFPW
      121 IQVPAYILVQ VVGSTLASGS LRLIFNGKED QFVGTVPAGT DLQALILEFI ATFYLMFVIA
      181 GVATDDRAMK HLSGVAIGAT VSLDILFSGP LTGASMNPAR SLGPAIVTGH YKGLWIYIIG
      241 PTLGAIFGAW TYNLMRLTNK SWGEAAKEIS HSQTAIEVSS KDKVICNCGE GWSCVVSKTE
      301 AAEVGNIFFE CAEGCICIVD ETSTLKKHVY VYEKTKRRKS YKMYI
//
LOCUS       SlNIP1-7       260 aa
DEFINITION  SlNIP1-7     260 aa
TITLE       SlNIP1-7
ORIGIN

        1 MASITSIIST NSSKNGIFAD FSSIEEGKHG TIQSPFLSAF QKIIAELVGT YIFIFVGCGS
```

```
       61 ALVDRERTLT IVGIALAWGL SLMALIYTLS HVSGAHFNPA VTIAFAAARK LPLMQVPMYV
      121 LPQFLGSTLA SLTLRVLFNH QGDILPMLTQ YKSPVTDFEA IFWEFLMTLI LMFVICGAAT
      181 DDRATKGVAG VAIGVTLVFE VLIAGPITGA SMNPARSLGP AIVSGVYKNQ WVFVIAPILG
      241 AMTATGIYGL LRQPKQNTKI
//
LOCUS       SlNIP2-1       283 aa
DEFINITION  SlNIP2-1     283 aa
TITLE       SlNIP2-1
ORIGIN

        1 MESEGGNCSK SINQNELVLK EDPKSNFFQK YYRSGIIKKV IAEIIATYLL VFVTCGAASL
       61 SWSDEHKVSK LGASVAGGLI VTVMIYAVGH ISGAHMNPAV TFAFAAVRHF PWTQVPVYAA
      121 AQVTGAISAA FTLRVLLHPV TKNVGTTTPS GSDIQALIME IVVTFSMMFI TSAVATDTKA
      181 IGELAGIAVG SAVCITSILA GPVSGGSMNP ARSIGPAMAS NDYRAIWVYI IGPVCGTLLG
      241 AWSYNFIKVN DKPVQAIVPG QSFSFKLRRM KSNNHDEEQC VTL
//
LOCUS       SlNIP3-1       306 aa
DEFINITION  SlNIP3-1     306 aa
TITLE       SlNIP3-1
ORIGIN

        1 MDPEEGVSAP STPATPGTPG APLFGGLIKH ERRNGGNGKK SLLKSCKCFG VEPWASEEGT
       61 LPAVTCMLPP PPISLARKVG AEFIGTLILI FAGTATAIVN QKTQGSETLI GLAASTGLAV
      121 MIVILSTGHI SGAHLNPAVT IGFAALNHFP WKHVPVYIGA QIIASFCAAF TLKVVLHPIM
      181 GGGVTVPSGS YVQAFALEFI ISFNLMFVVT AVATDTRAVG ELAGIAVGAT VMLNILIAGE
      241 TTGASMNPVR TLGPAVAVGN YKAIWIYLTA PILGALIGAG VYSAVKLPDE DRDNHPKPSL
      301 EHSFRR
//
LOCUS       SlNIP3-2       295 aa
DEFINITION  SlNIP3-2     295 aa
TITLE       SlNIP3-2
ORIGIN

        1 MAELENGISA PATPGTPTPL FPSLRVDSMG SYDRKSMPRC KCLPLDAPTW GAPHTCLADF
       61 PAPDVSLTRK LGAEFVGTFI LIFAATAGPI VNQKYNGAES LIGNAACSGL AVMIVILSTG
      121 HISGAHLNPS LTIAFAALRH FPWVQVPAYV AAQVSASICA SFALKGVFHP FMSGGVTVPS
      181 VNTGQAFALE FLITFNLLFV VTAVATDTRA VGELAGIAVG ATVMLNILVA GPSSGASMNP
      241 VRTLGPAVAA GNYKSLWIYL VAPTLGALAG AAVYTLVKLR GDTTETPRQV RSFRR
//
LOCUS       SlNIP3-3       284 aa
DEFINITION  SlNIP3-3     284 aa
TITLE       SlNIP3-3
ORIGIN

        1 MKLPSYENGL SVEFQVDASA SEQSTYDQET TSSNVEMLER RNVCNSILGI DPIFLRMVLA
       61 EALGTFLLMF CICGMMASME IMGVQVGLME YATTAALTVV VVVFSIGPIS GAHINPAVTL
      121 AFAAVGHFPW SKVPLYVVAQ VGGSILATYT GKLVYGLKAE FVTTKPLHSC TSAFFVELLA
      181 TFIVLFLSAS LTNYDPQSTG PLSGFLVGVA IGLAVLISGP VSGGSMNPAR SLGPAIVAWK
      241 FNNLWIYVIA PIIGAVAGVV FYRFLRLQGW SCKPNSTPTT HQHI
//
LOCUS       SlPIP1-1       286 aa
DEFINITION  SlPIP1-1     286 aa
TITLE       SlPIP1-1
ORIGIN
```

```
        1 MEHREEDVRL GANKYSERQA IGIAAQSEDK DYKEPPPAPL FEPGELMSWS FYRAGIAEFV
       61 ATFLFLYITV LTVMGVSKSD SKCSTVGIQG IAWAFGGMIF ALVYCTAGIS GGHINPAVTF
      121 GLFLARKLSL TRAVFYMVMQ CLGAICGAAL VKAFGKTLYQ TKGGGANVVN VGYTKGDGLG
      181 AEIIGTFVLV YTVFSATDAK RSARDSHVPI LAPLPIGFAV FLVHLATIPV TGTGINPARS
      241 LGAAVIYNNE QAWKDHWIFW VGPFIGAALA ALYHQVVIRA IPFKSK
//
LOCUS       SlPIP1-2       285 aa
DEFINITION  SlPIP1-2     285 aa
TITLE       SlPIP1-2
ORIGIN

        1 MEGKEEDVKV GANKYSERQP LGTSAQSKDY KEAPPAPLFE AGELHSWSFW RAGIAEFMAT
       61 FLFLYITVLT VMGYSRANSK CSTVGVQGIA WAFGGMIFAL VYCTAGISGG HINPAVTFGL
      121 FLARKLSLTR AVFYIVMQCL GAICGAGVVK GFQPSLFETK GGGANVVAHG YTKGDGLGAE
      181 IIGTFVLVYT VFSATDAKRN ARDSHVPILA PLPIGFAVFL VHLATIPITG TGINPARSLG
      241 AAIVYNKEHA WDDHWIFWVG PFIGAALAAL YHQVIIRAIP FKSGN
//
LOCUS       SlPIP1-3       287 aa
DEFINITION  SlPIP1-3     287 aa
TITLE       SlPIP1-3
ORIGIN

        1 MAENKEEDVN LGANKFREPQ PLGTSAQTDK DYKEPPPAPL YEPGELSSWS FYRAGIAEFM
       61 ATFLFLYITI LTVMGLKRSD SLCSSVGIQG VAWAFGGMIF ALVYCTAGIS GGHINPAVTF
      121 GLFLARKLSL TRAVFYMVMQ CLGAICGAGV VKGFMQGPYQ RLGGGANVVQ PGYTKGDGLG
      181 AEIIGTFVLV YTVFSATDAK RNARDSHVPI LAPLPIGFAV FLVHLATIPI TGTGINPARS
      241 LGAAIIFNQD QAWDDHWIFW FGPFIGAALA AIYHQIIIRA IPFKSRA
//
LOCUS       SlPIP1-4       286 aa
DEFINITION  SlPIP1-4     286 aa
TITLE       SlPIP1-4
ORIGIN

        1 MAENKEEDVK LGANKFRETQ PLGTAAQTDK DYKEPPPAPL FEPGELSSWS FYRAGIAEFM
       61 ATFLFLYITI LTVMGLKRSD SLCSSVGIQG VAWAFGGMIF ALVYCTAGIS GGHINPAVTF
      121 GLFLARKLSL TRAVFYMVMQ CLGAICGAGV VKGFMVGPYQ RLGGGANVVN PGYTKGDGLG
      181 AEIIGTFVLV YTVFSATDAK RNARDSHVPI LAPLPIGFAV FLVHLATIPI TGTGINPARS
      241 LGAAIIYNDE HAWNDHWIFW VGPMIGAALA AIYHQIIIRA MPFHRS
//
LOCUS       SlPIP1-5       288 aa
DEFINITION  SlPIP1-5     288 aa
TITLE       SlPIP1-5
ORIGIN

        1 MAENKEEDVN LGANKYRETQ PLGTAAQTDK DYKEPPPAPL FEPGELSSWS FYRAGIAEFM
       61 ATFLFLYITI LTVMGLKRSD SLCSSVGVQG VAWAFGGMIF ALVYCTAGIS GGHINPAVTF
      121 GLFLARKLSL TRAVFYIVMQ CLGAICGAGV VKGFMVGPYE RLGGGANVVN PGYTKGDGLG
      181 AEIIGTFVLV YTVFSATDAK RSARDSHVPI LAPLPIGFAV FLVHLATIPI TGTGINPARS
      241 LGAAIIFNKD EAWDDHWIFW VGPFIGAALA AVYHQIIIRA IPFKSSRS
//
LOCUS       SlPIP2-1       306 aa
DEFINITION  SlPIP2-1     306 aa
TITLE       SlPIP2-1
```

```
ORIGIN

        1 MVRHEWLKES TRAKKQRQPK KKTETQKWAK IWRLVLNMHQ KTYQDPPPAP LIDPEELGKW
       61 SFYRAIIAEF IATLLFLYIT VLTVIGYKSQ SSTDQCGGVG ILGIAWAFGG MIFVLVYCTA
      121 GISGGHINPA VTFGLFLARK VSLVRAIMYI VAQCLGAICG CGLVKAFQKA YYVKYGGGAN
      181 TLNDGYSTGT GLGAEIIGTF VLVYTVFAAT DPKRNARDSH VPVLAPLPIG FAVFMVHLAT
      241 IPVTGTGINP ARSFGAAVVY GHNKAWDDQW IFWVGPFIGA AIAAFYHQFI LRAGAVKALG
      301 SFRSNA
//
LOCUS       SlPIP2-2       259 aa
DEFINITION  SlPIP2-2     259 aa
TITLE       SlPIP2-2
ORIGIN

        1 MHQKIYQDPP PAPLIDPEEL GKWSFYRAII AEFIATLLFL YITVLTVIGY KSQSSTDQCG
       61 GVGILGIAWA FGGMIFVLVY CTAGISGGHI NPAVTFGLFL ARKVSLVRAI MYIVAQCLGA
      121 ICGCGLVKAF QKAYYVKYGG GANTLNDGYS TGTGLGAEII GTFVLVYTVF AATDPKRNAR
      181 DSHVPVLAPL PIGFAVFMVH LATIPVTGTG INPARSFGAA VVYGHNKAWD DQWIFWVGPF
      241 IGAAIAAFYH QFILRGWSC
//
LOCUS       SlPIP2-3       281 aa
DEFINITION  SlPIP2-3     281 aa
TITLE       SlPIP2-3
ORIGIN

        1 MAKDMEYGND QYAPSKDYQD PPPAPLIDPE ELGKWSFYRA IIAEFIATLL FLYITVLTVI
       61 GYKSQSDGDQ CGGVGILGIA WAFGGMIFVL VYCTAGISGG HINPAVTFGL LLARKVSLVR
      121 AIFYIVAQCL GAICGCGLVK LFQKAYYVKY GGGANELAVG YNIATGLGAE IIGTFVLVYT
      181 VFSATDPKRN ARDSHVPVLA PLPIGFAVFM VHLATIPITG TGINPARSFG AAVIYGKNKS
      241 WDDQWIFWVG PFIGAAIAAI YHQYILRAGA SKSINSFRSN A
//
LOCUS       SlPIP2-4       280 aa
DEFINITION  SlPIP2-4     280 aa
TITLE       SlPIP2-4
ORIGIN

        1 MTKEVTDFSA KDYTDPPPAP LVDFEELRQW SFYRAIIAEF IATLLFLYVT ILTVIGYKHQ
       61 ADVDAGGDVC GGVGILGIAW AFGGMIFILV YCTAGISGGH INPAVTFGLF LARKVSLIRA
      121 VLYMVAQCLG AICGVGFVKA FQSAYYNRYG GGVNVMAGGH SKGVGLGAEI IGTFVLVYTV
      181 FSATDPKRNA RDSHVPVLAP LPIGFAVFMV HLATIPVTGT GINPARSFGA AVIFNGDKAW
      241 DEHWIFWVGP FIGAFIAAFY HQFVLRAGAI KALGSFRSTA
//
LOCUS       SlPIP2-5       287 aa
DEFINITION  SlPIP2-5     287 aa
TITLE       SlPIP2-5
ORIGIN

        1 MTKEVEAVHE QAVEYSAKDY TDPPPAPLID FEELTKWSLY RAAIAEFIAT LLFLYITILT
       61 VIGYKHQADV KAGGDICGGV GLLGIAWAFG GMIFVLVYCT AGISGGHINP AVTFGLFLAR
      121 KVSLIRAILY MVAQCLGAIC GVGFVKAFQS AYYNRYGGGV NVMAGGHTKG VGLAAEIIGT
      181 FVLVYVVFSA TDPKRSARDS HVPVLAPLPI GFAVFMVHLA TIPITGTGIN PARSFGAAVI
      241 FNGDKAWDEH WIFWVGPFIG AFIAAVYHQY ILRAGAIKAL GSFRSNA
//
LOCUS       SlPIP2-6       283 aa
```

```
DEFINITION  SlPIP2-6     283 aa
TITLE       SlPIP2-6
ORIGIN

        1 MSKEVIEEGQ VQQHGKDYVD PPPAPLLDFA ELKLWSFYRA LIAEFIATLL FLYVTVATVI
       61 GHKKLNGADK CDGVGILGIA WAFGGMIFVL VYCTAGISGG HINPAVTFGL FLARKVSLVR
      121 AVGYIIAQCL GAICGVGFVK AFMTHPYNAL GGGANFVQSG YNNGTALGAE IIGTFVLVYT
      181 VFSATDPKRS ARDSHIPVLA PLPIGFAVFM VHLATIPITG TGINPARSFG AAVIADNKNV
      241 WDDQWIFWVG PFVGALLAAA YHQYILRAAA IKALGSFRSN ATN
//
LOCUS       SlPIP2-7       283 aa
DEFINITION  SlPIP2-7     283 aa
TITLE       SlPIP2-7
ORIGIN

        1 MSKDVIEEGQ AHHHGKDYVD PPPAPLLDMA ELTKWSFYRA LIAEFIATLL FLYVTVATVI
       61 GHKKLNALDQ CDGVGILGIA WAFGGMIFVL VYCTAGISGG HINPAVTFGL FLARKVSLIR
      121 AVAYIIAQSL GAICGVGFVK AFMKHYYNTE GGGANFVQPG YNNGTALGAE IIGTFVLVYT
      181 VFSATDPKRS ARDSHVPVLA PLPIGFAVFM VHLATIPITG TGINPARSFG AAVIYGNEKI
      241 WDDQWIFWVG PMVGAMAAAI YHQFILRAGA VKALGSFRSN QTN
//
LOCUS       SlPIP2-8       259 aa
DEFINITION  SlPIP2-8     259 aa
TITLE       SlPIP2-8
ORIGIN

        1 MVKDYVDPPS APLFQTAELY NWSFYRALIA EFVATLLFLY VSVATVIGHK KQLGPCDGVG
       61 LVGIAWAFGG MIFVLVYSTA GISGGHINPA VTFGLLLARK VSLLRAVAYM VAQCLGAICG
      121 VGLVKGVMKD DYTKHGGGAN TVAVGYSTGA ALGAEIIATF LLMYTVFSAT DAKRKARDSH
      181 VPVLAPLPIG FSVFMVHLAT IPITGTGINP ARSFGAAVIY NDTTAWNDHW IFWVGPFLGA
      241 LAAVIYHQQI LRGHAAKAF
//
LOCUS       SlPIP2-9       289 aa
DEFINITION  SlPIP2-9     289 aa
TITLE       SlPIP2-9
ORIGIN

        1 MSNEVSSALP ERSSSPAKDY HEPPPAPFIG AAELKKWALY RALIAEFVAT LLLLYIGQLT
       61 IMGYKSESDH DPCGSVGLLG VAWVFGGMVF ILVYCTAGIS GGHINPAVTF GLFLSRKISL
      121 IRGLLYIVVQ YLGAICGTAL VKAIYKSKFE LYGGGVNSVS PGYTRGVAWS AEMIGTFVLV
      181 YTVLSATDSK RNARDSHVPV LAPLPIGFAV FLVHLATIPI TGTGINPARS LGAAVIYNQQ
      241 IAWEDPWDLF WRTFHRSTYC SNLPTNTEGM QMEISDATYI CSRICVCDL
//
LOCUS       SlSIP1-1       242 aa
DEFINITION  SlSIP1-1     242 aa
TITLE       SlSIP1-1
ORIGIN

        1 MGVIKAAIAD GLLTFLWVFC SSNIGVSTYF IASYFGIVNE IPSLFITTLI VFVIFLMFDF
       61 LGDVLGGAGF NPTGNAAFYA AGLGDDSLVS AAVRCPAQVA GAVAGSLALV ELIPKHYHHM
      121 LDGPALKVDV QTGAIAEGVL TFVITFMIFV IVLRGPESVL LKNWLLTMVT LPLVLAGSNF
      181 TGPSMNPANA FGWAYLSNTH KTLEHFYVYW ISPFIGAILA AWIFRVLFPP PVEQKPQKQK
      241 RN
//
```

```
LOCUS       SlSIP1-2       243 aa
DEFINITION  SlSIP1-2     243 aa
TITLE       SlSIP1-2
ORIGIN

        1 MGAVKAAVGD FVLTLMWVFC SSTLGIFTYL IATAFGIAQG MASLFITTVL LFMLFFVFGI
       61 IGDALGGAAF NPAGTAAFYA AGVGKDSLFT VATRFPAQAA GAVAGAVAIL EVIPTQYKHM
      121 LGGPSLKVDL HNGAIAEGIL TFVMTFLVFI IVLKGPKSAL LKNWLLAMST VTMVVAGSKY
      181 TGPSMNPANA FGWAYINNMH NTWEQFYVYW ICPFVGAIMA AWTFRAVFPA PAKKKKPQKK
      241 KRN
//
LOCUS       SlSIP2-1       216 aa
DEFINITION  SlSIP2-1     216 aa
TITLE       SlSIP2-1
ORIGIN

        1 MGVSRRSLVI SDFIMSFMWV WSSVLIKMFV HKILGYGAHD LKGGAYNPLT ILSGAISGDL
       61 TNFIFTVAAR IPSQVFGSIT GVRFIIAAFP NIGRGPVLSI DIHRGALTEG ILTFAIVSIS
      121 LGLSRRSRAS TFMKTWISSL SKLTLHILGS DLTGGCMNPA SVMGWAYARG DHITKEHIHV
      181 YWLAPIQATL LAVWTFNLLV SPSKDKEAKK TEKKSE
//
LOCUS       SlTIP1-1       250 aa
DEFINITION  SlTIP1-1     250 aa
TITLE       SlTIP1-1
ORIGIN

        1 MPINQITIGS HEELRHPGAL KAALAEFIST LIFVFAGQGS GMAFNKLTDG VATPAGLISA
       61 SIAHAFGLFV AVSVGANISG GHVNPAVTFG AFVGGNITLF RGILYIIAQL LGSTAACALL
      121 EFATGGMSTG SFALSAGVSV WNAFVFEIVM TFGLVYTVYA TAVDPKKGDL GVIAPIAIGF
      181 IVGANILAGG AFTGASMNPA VSFGPSLVSW TWTHQWVYWA GPLIGGGLAG FIYEFIFISH
      241 THEQIPSGDF
//
LOCUS       SlTIP1-2       248 aa
DEFINITION  SlTIP1-2     248 aa
TITLE       SlTIP1-2
ORIGIN

        1 MPISRIAIGR REEATHPDAL KAALAEFIST LIFVFAGSGS GVAFSKLTGG GANTPTGLIA
       61 AAIAHAFGLF VAVSVGANIS GGHVNPAVTF GAFVGGNITL LRGILYWIAQ LLGSVVACLL
      121 LKFTTGGMEI GAFSLSNGVG VGNALVLEIV MTFGLVYTVY ATAVDPKKGS LGTIAPIAIG
      181 FIVGANILVG GAFDGASMNP AVSFGPALVS WAWSNHWVYW VGPLIGGGLA GLIYEFFFIN
      241 QTHEPLPQ
//
LOCUS       SlTIP1-3       253 aa
DEFINITION  SlTIP1-3     253 aa
TITLE       SlTIP1-3
ORIGIN

        1 MPISRIAIGN LAEATKPDAL KAATAEFFSM LIFVFAGSGS GMAFGKLTNG GAATPAGLIS
       61 ASIAHAFALF VAVSVGANIS GGHVNPAVTF GAFVGGHITL FRSVLYWIAQ LLGSVVACVL
      121 LKFSTGGLET SAFALSSGVT PWNAVVFEIV MTFGLVYTVY ATAVDPKKGD LGIIAPIAIG
      181 FIVGANILAG GAFDGASMNP AVSFGPAVVS WTWDNHWVYW LGPFGGAAIA ALVYEIIFIG
      241 QNTHEQLPTT DDY
//
```

```
LOCUS       SlTIP2-1       273 aa
DEFINITION  SlTIP2-1     273 aa
TITLE       SlTIP2-1
ORIGIN

        1 MGSSPFKQLT QPRNKHIANY SFSQNALYSL GRFDDSLNCG SIKAYLAEFI STLLFVFAGV
       61 GSAIAYNKLT ADAALDPAGL VAVAVCHGFA LFVAVSIGAN ISGGHVNPAV TFGLALGGQI
      121 TLLTGLFYWV AQLLGAIVAC CLLKFVTGGL TVPIHGVAAG VGATEGVVME IIITFALVYT
      181 VYATAADPKK GSLGTIAPIA IGFIVGANIL AAGPFSGGSM NPARSFGPAV VSGNFAGNWI
      241 YWVGPLIGGG LAGLIYSNVF MNYGDHVPLS SDF
//
LOCUS       SlTIP2-2       248 aa
DEFINITION  SlTIP2-2     248 aa
TITLE       SlTIP2-2
ORIGIN

        1 MPCIAFGRFD DSFSLGSIKA YIAEFISTLL FVFAGVGSAI AYNKVTADAA LDPSGLVAVA
       61 VCHGFALFVA VAIAANISGG HVNPAVTFGL ALGGQITLLT GLFYWIAQLL GAIVGCYLLK
      121 VVTGGMAVPI HGVAAGVGAA EGVVMEIIIT FALVYTVYAT AADPKKGSLG TIAPIAIGFI
      181 VGANILAAGP FSGGSMNPAR SFGPAVVSGN FAGIWIYWVG PLVGGGLAGL IYSNVFMNHE
      241 HAPLSSDF
//
LOCUS       SlTIP2-3       250 aa
DEFINITION  SlTIP2-3     250 aa
TITLE       SlTIP2-3
ORIGIN

        1 MVKIAFGSIG DSLSVGSLKA YLAEFIATLL FVFAGVGSAI AFNKLTSGAA LDPAGLVAIA
       61 VAHAFALFVG VSMAANISGG HLNPAVTLGL AVGGNITILT GLFYWVAQLL GSTVACLLLK
      121 YVTNGLAVPT HGVAAGMSGA EGVVMEIVIT FALVYTVYAT AADPKKGSLG TIAPIAIGFI
      181 VGANILAAGP FSGGSMNPAR SFGPAVVAGD FSQNWIYWVG PLIGGGLAGF IYGDVFIGCH
      241 TPLPTSEDYA
//
LOCUS       SlTIP3-1       260 aa
DEFINITION  SlTIP3-1     260 aa
TITLE       SlTIP3-1
ORIGIN

        1 MAMPARRYAF GRADEATHPD SMRATLSELL STFIFVFAGE GSVLAIDKLY PDTGLGSSRL
       61 IVIALAHAFS FFAAVASSLN VSGGHINPAV TFGSLVGGRI SVVRAIYYWV AQLFGSVLAS
      121 LLLRLATDGL RPRGFSVAAG VGNLNALVME IVMTFGLMYT VYATAVDPRR GSLSTIAPLA
      181 IAFILGANTL VGGPFEGASM NPARAFGPAL VGWRWRNHWI YWLGPFIGAA LAGLIYEYGI
      241 IQHETVPRPT THQPLAPEDY
//
LOCUS       SlTIP3-2       259 aa
DEFINITION  SlTIP3-2     259 aa
TITLE       SlTIP3-2
ORIGIN

        1 MQPRRYEFGR ADEATHPDSV RATLSEFLST FIFVFAGEGS VLALDKLYPD RALGASRLTA
       61 IALAHAFSLF AAVASSMNVS GGHINPAVTF GALVGGRVSV LRAMYYWIGQ LLGAVVASAL
      121 LRLATDGLRP VGFAVASGVG NGNALVMEIV MTFGLVYTVY ATAIDPKRGS LGIIAPLAIA
      181 FIVGANVLVG GPFEGASMNP ARAFGPALVG WRWRNHWIYW VGPFIGAAIA GIIYEFGLIQ
      241 AHDEAPVHTH HQPLAPEDY
```

```
//
LOCUS       SlTIP4-1       247 aa
DEFINITION  SlTIP4-1     247 aa
TITLE       SlTIP4-1
ORIGIN

        1 MAKIAVGSSR EAIQPDCIQA LIVEFICTFL FVFAGVGSAM AANKLNGDPL VSLFFVAMAH
       61 ALVVAVTISA GFRISGGHLN PAVTLGLCMG GHITVFRSIL YWIDQLLASV AACALLNYLT
      121 DGMITPVHTL ANGMSYGQGL IMEVILTFSL LFTVYTTLVD PKKGILEGMG PLLTGLVVGA
      181 NIMAGGPFSG ASMNPARSFG PAFVSGIWTD HWVYWIGPFI GGGLAGFICE NFFIVRSHVP
      241 LPNEETF
//
LOCUS       SlTIP5-1       251 aa
DEFINITION  SlTIP5-1     251 aa
TITLE       SlTIP5-1
ORIGIN

        1 MASLASRLQH SVTPNALRSY LAEFLSTFFF VFAAAGASMS TRKMVPDATS DPSSLVAIAV
       61 ANAFALSVAV YISANISGGH VNPAVTFGMA VGGHISIPMS IFYWISQMIG SVTACLLLKF
      121 TNQQVPTHGI PQEMTGFGAA VLEGVMTFGL VYTVYAAADP RRCVHAAIGP LAIGLMLGAN
      181 VMASGPFTGG SMNPAYSFGS AVVKGSFGNQ AVYWIGPFIG AAIAGLVYDN VVFPLQVTES
      241 LRGIGGGIVA V
//
LOCUS       SlXIP1-1       325 aa
DEFINITION  SlXIP1-1     325 aa
TITLE       SlXIP1-1
ORIGIN

        1 MASKNNVVFG DEENQLPGGT NKVQPCSSTS RKNTIDDEGK KPIFLSFSER LGVPDFFCLD
       61 VWRASMGELL GSAVLVFMLD TIVISTLESD TKMPNLIMSI LIAIVITILL LAVVPVSGGH
      121 INPIISFSAA LVGIISMSRA IIYIMAQCVG AILGALALRA VVSSSIEDTF SLGGCTITII
      181 APGPNGPVIV GLEMAQALWL EIFCTFVFLF ASIWMAYDHR QAKSLGHVTV LSIVGVVLGL
      241 LVFISTTVTA RKGYGGAGMN PARCLGPAII RGGHLWDGHW IFWVGPTIGC VAFYVYTKII
      301 PPKHFLGEYG FKHDFVGVVK ALSNV
//
LOCUS       SlXIP1-2       327 aa
DEFINITION  SlXIP1-2     327 aa
TITLE       SlXIP1-2
ORIGIN

        1 MASNSNVVFG DEESQISGGT NRVQPCSSTP RKINTIDDDE GKKHNFLSLS QRLGVADFFC
       61 LDVWRASMGE LLGSAVLVFM LDTIVISTFE SETKMPNLIM SILIAVVITI LLLAVVPVSG
      121 GHINPVISFS AALVGIISMS RAIIYIVAQC VGAILGALAL RAVVSSSIED TFSLGGCTVT
      181 IIAPGPNGPV IVGLETAQAL WLEIFCTFVF LFASIWMAYD HRQAKALGHV TVLSIVGLVL
      241 GLLVFISTTV TAKKGYGGAG INPARCLGPA IIRGGHLWDG HWIFWVGPTI GCVAFYVYTK
      301 IIPTKHFLAE YGFKHDFVGV VKALSNV
//
LOCUS       SlXIP1-3       302 aa
DEFINITION  SlXIP1-3     302 aa
TITLE       SlXIP1-3
ORIGIN

        1 MFSNTQRFKL NNIDDDGKKP NFLSILGVPG FFCLDVWRAS MGELIGSAVL VFMLDTIVIS
       61 TLESDTKMPN LIMSILIAIV ITILLLAVVP VSGGHINPII SFSAALVGII SMSRAIIYIM
```

```
      121 AQCVGAILGA LALRAVVSSS IEDTFSLGGC TITIIASGPN GPVIVGLEMA QALWLEIFCT
      181 FVFLFASIWM AYDHRQAKAL GLVTVLSIVG LVLGLLVFIS TTITAKRGYG GAGMNPARCL
      241 GPALVRGGHL WDGHWIFWVG PTIGCVAFYV YTKIIPAEHF NAEYGYKHDF VGVVKALFGS
      301 NV
//
LOCUS       SlXIP1-4       327 aa
DEFINITION  SlXIP1-4     327 aa
TITLE       SlXIP1-4
ORIGIN

        1 MGSNNNAVFG DEENQFSCGT NKVQPCSSTP KRSTIDDEGK KLNFLSFSER LGVSDFFSLD
       61 VWRASIGELL GTAVLVFMLD TIVISTLESD IKMPNLILSI LAAVIITILL LAVVPVSGGH
      121 INPVISFSAA LVGIISMSRA IIYIMAQCLG AILGALALKA VVSSSIEDTF SLGGCTITII
      181 APGPNGPISV GLETAQALWL EIFCTFVFLF ASIWMAYDHR QAKALGHVTV LSIVGVVLGL
      241 LVFISTTVTA KKGYAGAGMN PARCFGPAIV RGGHLWDGHW IFWVGPIIGC VAFYVYTKII
      301 PTKHFNAEYG YKHDFVGVVK ALVGSNV
//
LOCUS       SlXIP1-5       328 aa
DEFINITION  SlXIP1-5     328 aa
TITLE       SlXIP1-5
ORIGIN

        1 MGSNNNIFLG DEESQLSAGN TNRVQPYSST PKKNTIDDEG KKQNFLSLSQ RLGVPDFFSL
       61 DVWRASMGEL LGSAVLVFMV DTIVISTSES DAEMPNLIMS ILLAIVLIVL LLAVVPVSGG
      121 HLNPVISFSA ALVGIISMSR AIIYIMAQCL GAVLGALALK AVVSSTIEDT FSLGGCTITI
      181 IAPGPNGPVT LGLETAQALW LEIFCTFVLL FASIWMAYDH RQAKALGLVT VLTIVGLVLG
      241 LLVFISTTVT TKRGYAGVGM NPARCLGPAI VRGGHLWDGH WIFWIGPTIS CLAFYVYTKI
      301 IPPKHFHAEY GYKHDFVGVV KALFESNV
//
LOCUS       SlXIP1-6       520 aa
DEFINITION  SlXIP1-6     520 aa
TITLE       SlXIP1-6
ORIGIN

        1 MAIRMPRIIK KSSDIPKGHF VVYVGEKQKK RFVIPISFLS EPLFQDLLNQ AEEEFGFDHP
       61 MGGVTIPCTE DLFVNLTSRL RNLHLGYVPV LVDSVADTNR HKAHMVLMMR GPLFQMAIRV
      121 PRIIKKSSTS LQVPKGHFAV YVGEKQKKRF VIPISYLSQS SFQDLLSQAE EEFGFDHPMG
      181 GVTIPCPEDI FIMDSTNGNV LGDEESQLSF GSSNKIQPTI SITQKQVPSS DDEKKKYTCL
      241 TMAERLGLPD FFSLDVWRAS VGELLGSAVL VFMLDTVVIS TLESDVKMPN LIMSILIAIT
      301 LTILILAVFP VSGGHISPVI TFSSALVGLI SMSRAIIYIV AQCVGAILGA LALKAVLSST
      361 IEQRFSLGGC TVTVVTPGLN GPEIVGLEIA QAFWLEFMCT FALLFGSLWM AYDHRQSKAL
      421 GLITVMSIVG LLAGILVFIS TSVTAKKGYA GAGMNPARCF GAAFVRGGHL WNGHWIFWVG
      481 PGLACFAFYF YTKIIPSDHF QTDGYKHDFL AIIETLFNQR
//
LOCUS       SmHIP1-1       215 aa
DEFINITION  SmHIP1-1     215 aa
TITLE       SmHIP1-1
ORIGIN

        1 RAAGAEFLGT LLFVYLGCGS VIASGMLGPG MTAARLVAIA LGHGLAIAFL AGATGAISGG
       61 HLNPAVTLAF VVAGKETLLR AGLYVGAQLF GAIMGAAILR WSTPGPWVGA LGAHDLSNGV
      121 YPGQGFIMEF MLTFVLVFVI FGVAVDRRGP GVIAPLPIGF AVLVDHLVGV PFTGASMNPA
      181 RSFGPAVVSG AWSASFWIYW FGPCFGAAAA SALYK
//
```

```
LOCUS       SmNIP3-1       221 aa
DEFINITION  SmNIP3-1     221 aa
TITLE       SmNIP3-1
ORIGIN

        1 QLLSEFLGTF ILIFAAAGTA IMNEKSHGAL GVHGLAGGAG ITVMVVIFAT GHISGAHINP
       61 AVTVAFATYR HFPWFQVPLY IAAQVTASIS ASFLLKGIYH PDLAGGVTVP AGTHWQSFLF
      121 EIILTAIMMF VVTSVATDTR AVGELAGIAV GATVFLNNLI AGLISGASMN PCRSLGPAIA
      181 ANNFKGFWIY VIGPLIGTQL GAAAYTAIRF KELERPKSFR R
//
LOCUS       SmNIP3-2       259 aa
DEFINITION  SmNIP3-2     259 aa
TITLE       SmNIP3-2
ORIGIN

        1 MGSGSTDYAL IQNDEEAGTS LEPVSGYSPS FTISKKLLCE FLGSVVLLLG GAGSAIINTQ
       61 TNGALGIHGL AGGSAIAVAI VIMSTGHISG AHINPAVTLA FATFRHFSWI QVPLYIVAQL
      121 AGSLACAFLL KGMYNPDHLA TGVTVPAGST LQSLLFEIVL TAVLMFVITS VATDTRAVGE
      181 LAGIAVGLAV YLDILLGGYV SGASMNPVRT LGPAVAARDF RALWIYFVGP VVGAQIGGGL
      241 YTLIRFKDHT ERRKVFRKE
//
LOCUS       SmNIP3-3       221 aa
DEFINITION  SmNIP3-3     221 aa
TITLE       SmNIP3-3
ORIGIN

        1 MCSLDSFVKV LAEFLGSLVL LLGCAGTAIL ASKPSNGFGI HGISAGAALT VMIVIFSTGH
       61 ISGAHVNPAV SLAFASLGRF PWIQVPLYSG AQFLGSVCAS FLLNALFQGD PNIHAGVTVP
      121 SNTEWQSFAV ELVISAILMF VVTAVATDPH AIGDSAAVAV AATVYLNNLL ASAISGASMN
      181 PIRTFGPALA AGEYRGLWVY FFGPILGTQL GAGFYTLIRS S
//
LOCUS       SmNIP3-4       276 aa
DEFINITION  SmNIP3-4     276 aa
TITLE       SmNIP3-4
ORIGIN

        1 MAPDRISNVS TSFGRSGRIN KCFPVDSCVM EDGLFSEFQA AIAPSSISRR KKIGAEFIGT
       61 FMLIFAGTAT AIVNEKTSGS ITTVGLAATS GLAVMIVILA TGHISGAHLN PSLTLSFAAL
      121 RHFPWVEVPL YIGAQVAASI CAAFALKGIF NPFMHGGVTI PSGSYWESFV LEFIISFNLM
      181 FVVTAVATDS RAVGELAGIA VGATVMLNIL IAGSTSGASM NPVRTLGPAI AANNYKGIWL
      241 YIVGPIFGAL AGAAAYTLVR LKGEPEPGRP RFSMRR
//
LOCUS       SmNIP3-5       226 aa
DEFINITION  SmNIP3-5     226 aa
TITLE       SmNIP3-5
ORIGIN

        1 LIFLIQALAG EALGTFLLVF TIAALTAVNE GTPGGIGLLS FAMAASFCVT VIILTIGHTS
       61 GAHINPSITV GFAAAGRFPW SQVPFYMVSQ ITGSVLAILA AKWVYSFPES DFAVTQPRSG
      121 PWQSLVLETA MSFVVMFLAC ILSNNTSQSG NAAAMAVPAA IGLSVMVAGP ISGGSLNPAR
      181 SLGPAIVSRN FKAIWIYIAG PFLGCVTAGI AHSPVLEGKE DSQNSM
//
LOCUS       SmNIP5-1       284 aa
DEFINITION  SmNIP5-1     284 aa
```

```
TITLE       SmNIP5-1
ORIGIN

        1 MAAAAAGMND FQLSEIRVTP APSIALPGNG HQIHHQASPP AASRGCFPVA IVPKSTLFQK
       61 IGAEVISTFI LVFAGCGAAM VDAKYKDSIT HLGVSAAFGL VVMIMVYAVG HISGAHMNPA
      121 VTLAFATVRH FPWQQVPAYI GAQITAAITA AFALRLIISP VANIGATIPA GSDLQSFYLE
      181 AIITYILMFV VSAVATDARA IGELAGLAIG ATVGLNAIFA GPISGASMNP ARSLGPAIAA
      241 NNYSGLWVYI VGPTVGALAG ACSYNMIRLP VKPDELPRAA SFKR
//
LOCUS       SmNIP5-2       210 aa
DEFINITION  SmNIP5-2     210 aa
TITLE       SmNIP5-2
ORIGIN

        1 QLGAEVIATF ILVFAGAGAG MVNELTNGSL TFFGVAAANG LVVMMMIHAT GHISGAHMNP
       61 AVTVAFATVR HFPWAQVPLY IGSQIAASVS ACFVLRQLLT EVNKIGATVP AAGNVVQALV
      121 LEIIVSYILM FVVAAVSTDT RAVGELAGLA VGATVALNNL IAGPLSGASM NPARSIGPAV
      181 ARNNYSDVWI YIVGPVLGTL GGAWSYNLIR
//
LOCUS       SmNIP5-3       260 aa
DEFINITION  SmNIP5-3     260 aa
TITLE       SmNIP5-3
ORIGIN

        1 MRSSYSRLLL LLLERSTDPI HLFDHANFFG LVLILKVGAE FIGTFILIFT AAATPIVNKQ
       61 LGGLSVFALS ATPALAVTTI IFSTGHICGA HLNPSVTISF AALGQFPWIQ VPVYIVAQLL
      121 ASVLASFILK GVYYPDIAAG VTVPIGSDLQ AFVLELVISF ILMFVNTALA TDRSAVGDMA
      181 AVAVGATVFV NNLAASQATG ASMNPARTIG PAIAANCYKS LWVYIVAPTL GCLLGAAGYT
      241 IVRTTGASKP KKWGRNELLQ
//
LOCUS       SmPIP1-1       281 aa
DEFINITION  SmPIP1-1     281 aa
TITLE       SmPIP1-1
ORIGIN

        1 MEGNREDVHV GVAKYPEREL GTSAQAEKDY VEPPPTRLIE PSEFSSWSFW RAGIAEFFAT
       61 FLFLYITILT VVGNVDRTSC LGVGIQGIAW AFGGMIFALV YCTAGISGGH INPAVTFGLF
      121 LARKVSLPRT LLYMVAQCLG AICGAGVVKG FQKAKFNAAG GGANYVHHGY TIGDGLGAEI
      181 VGTFVLVYTV FSATDAKRSA RDSHVPLLAP LPIGFAVFLV HLATIPITGT GINPARSLGS
      241 AVIYNGDQAW DDHWIFWVGP LIGAALAAFY HQFVIRAIPF H
//
LOCUS       SmPIP2-1       290 aa
DEFINITION  SmPIP2-1     290 aa
TITLE       SmPIP2-1
ORIGIN

        1 MAKDASKESE AFVTAKDYEE PPPARLVDPK EFGSWSFYRA GIAEFVATLL FLYITVQTVI
       61 GHSRNAANCG GVGLLGIAWA FGGMIFVLVY CTAGISGGHI NPAVTFGLFV ARKVSLPRAI
      121 FYMIMQCLGA IVGCGLAKGF QKSFYVQQGG GANSVARARG YSTGTGLGAE IIGTFVLVYT
      181 VFSATDPKRV ARDSHVPVLA PLPIGFAVFM VHLATIPITG TGINPARSFG AAVIFNKSVS
      241 WDDQWIFWVG PFIGAAAAAI YHQYVLRAGS ALKALGSFRS NPPHQHHGHP
//
LOCUS       SmPIP2-2       266 aa
DEFINITION  SmPIP2-2     266 aa
```

```
TITLE       SmPIP2-2
ORIGIN

        1 MSKDLENGNA AKDFSEPPPT PLIDLAELKS WSLYRACIVE LVATLLFLYI GVTALIGHAR
       61 AQAAAGGDSC GGIGLLGVAW VFGGMIFVLV YCTAGVSGGH LNPAVTFGMF LARKVSIPRA
      121 LLYVASQVAG AIFGAGLAKG FQASFYNGNM GGATFIQNGY TKAEGLGAEI IGTFVLAYTV
      181 FSATDPKRVA RDSHVPVLAP LPIGFAVFMV HLALLPVTGS SVNPARSFAT AVIYNNSRVW
      241 NDQWVYWVGP LLGAALAAMY HQYILR
//
LOCUS       SmSIP1-1       236 aa
DEFINITION  SmSIP1-1     236 aa
TITLE       SmSIP1-1
ORIGIN

        1 MGMLKLILAD AAISFLWVFC TSCIGAATEI IASCAGVEGD RKFYIVFGLI ALLIVVFSAL
       61 AQAIGGASWN PTAVLAFSGV GVGDSIFNLA LRIPAQAVGS AAGALAIFEL MPSSFKRTLG
      121 GPSLKVDLRT GAIAEGLLSF IMSFVVLWAV LRGPRNAALK SGIIISTTIA LIVLGSGYTG
      181 PAMNPANAFA WAYVNKSHNT NEHLYVVYWLT PLLGSLAASM VFKRMFALGQ NKEKTA
//
LOCUS       SmTIP2-1       244 aa
DEFINITION  SmTIP2-1     244 aa
TITLE       SmTIP2-1
ORIGIN

        1 MARLAVGGAD EYRQPDAMKG ALAEFIGTFL FVFAGVGSAM AFAKLEGPIL TPAGLVQIAL
       61 AHGIALFVVI AATANISGGH INPAVTFGLA VGGHITIARG VLYWIAQLLG SVLAALVLKL
      121 TFLHESVPIH AVGAHESLIS ALVIEIVTTF ALIFTVYGTA VDHKRGVVGT IAPIAIGFIV
      181 LANILAAGPF SGGSMNPARS FGPALITFNW TNHWIYWVGP LIGGGLAGLV YNEILITPPP
      241 PEEY
//
LOCUS       SmTIP2-2       244 aa
DEFINITION  SmTIP2-2     244 aa
TITLE       SmTIP2-2
ORIGIN

        1 MARLTVGGAD EYRQPDAMKA ALAEFIGMFL FVFAGVGSAM AFAKLGGPIL TPAGLVQIAL
       61 AHGIALFVVI AATANISGGH INPAVTFGLA VGGHITIARG VLYWIAQLLG SVLAALVLKF
      121 TFLHEAVPIH AVGAHESVIS ALVIEIVTTF ALIFTVYGTA VDHKRGVVGT IAPIAIGFIV
      181 LANILAAGPF SGGSMNPARS FGPALVTFDW TNHWIYWVGP LIGGGLAGLV YNEILITPPP
      241 PEEY
//
LOCUS       SmTIP6-1       263 aa
DEFINITION  SmTIP6-1     263 aa
TITLE       SmTIP6-1
ORIGIN

        1 MLRSWSLGVR DPFSPTTLLG CAAEFIGTFL FVFLGCGSVV SSGIVDDQLN SARLLVIAIA
       61 HGFAIAILVA ATAGVSGGHL NPAVSFGFMM SGNMSIIKGL MYWISQLAGA VLGAGFYREF
      121 PSAIAGHFGA HAVNSKFTVI EAFGLEALLT FVLVYVIFGT AVDKKGPSTI APLTIGMAVL
      181 VDHLVGVPVT GASMNPARSL GAALWSGQWK NHWIYWAAPL LGATAAALIY TALFLPTVSS
      241 TQKQSTNDLI KNIKNEKHTE AVV
//
LOCUS       SmXIP1-1       297 aa
DEFINITION  SmXIP1-1     297 aa
```

```
TITLE       SmXIP1-1
ORIGIN

        1 METLEHQNFH HHHKQASSLQ ILKFLQLQDF ITLEAWRSCL VECLATIAFV FSSILIIVSS
       61 SQQSSSGSPM ASIAILHSIL VFIIILSVAP SSGGHFNPCI TFLAMVLGYV SPSKLVMYTG
      121 AQCVGSIFAS LCAKLVLPDD LALKFALGGC VMQNSAGSGL SIGSAFVAES MFTFVLMFVV
      181 ANLALDPFQG RAFGAILPPS FIGAAVGLLI FASGGLVVGY SGAGMNPARC LGPAVVMGSS
      241 STRDGHWVWW IAPYSATVMV SVLYCLVPPH HALYRGREDL DLVSSVKEAF HPPIVVS
//
LOCUS       SmXIP1-2       277 aa
DEFINITION  SmXIP1-2     277 aa
TITLE       SmXIP1-2
ORIGIN

        1 LQVWRSCLVE CLATGAFVFA SILIIVSCSQ QSASPMTAIS VLHFLVISFL ILSVAPSSGG
       61 HLNPCITFMA MVLGYVSPSR VVMYTGAQCI GSIFGSLCMK LVLPHEVAHK FALGGCTIRN
      121 SAGAGFPVGS AFVAETFFTF FLMFIVATLA LDPFRAPVFG AILAPTFIAA AVGVLVFVSG
      181 GLALGYSGAG MNPARCIGPA VVMGSHNEEL WRGHWLWWVA PYFATVLVAV VYCLVPPHHV
      241 DIYRGRKDFI SSLKKAFGSH HPQVPSETRI SSENLTP
//
LOCUS       SmXIP1-3       182 aa
DEFINITION  SmXIP1-3     182 aa
TITLE       SmXIP1-3
ORIGIN

        1 VGAGHLNPIF TYTTMLTGHT TAVRAMLYMA AQSLGALIGT LAAKQVVARD FVEKNSLAGC
       61 LLGEVVRTSR GLEWPEGLTP RAGLISEIFF TFLLIFVAYS TLLEPGNFRQ TGPILAALSI
      121 GGAAGLFTFI SSQLKSPYYS GAGMNPARCL GPAIVQGSTL WDCHWVFWAG PFIAGTIFGV
      181 VF
//
LOCUS       StNIP1-1       181 aa
DEFINITION  StNIP1-1     181 aa
TITLE       StNIP1-1
ORIGIN

        1 MAEISDISHA TALDIKDDNV HHILSCPCLA SKKLGDETIS FTQKLVAELL GTYLSMFAGF
       61 AAMVVNKNIG LLGIAVLWGL DMMVMIYTVG PVSGAHFNPA VTVVVASCKR IAWRHEEQLQ
      121 FLRTIPAGSD LQSLGLEFLI TFYLMFAVAG TTMDNRAVGE LTGRVIGAVI TINSILAGYA
      181 K
//
LOCUS       StNIP1-2       290 aa
DEFINITION  StNIP1-2     290 aa
TITLE       StNIP1-2
ORIGIN

        1 MGDQQIGGGA NGSVSLNIRD ADDDLNNKNC ANSASHQDSS STSTCSFVTI PFIQKIIAET
       61 LGTYFLIFAG CGSVAVNADK GMVTFPGISI VWGLVVMVMV YSVGHISGAH FNPAVTIAFA
      121 SNKRFPWKQV PAYVAAQVIG STLASGTLRL IFNGKHDHFV GTSPTGSDIQ SLVLEFIITF
      181 YLMFVISGVA TDNRAIGELA GLAVGATVLL NVMFAGPISG ASMNPARSLG PAIVSSHYKG
      241 LWVYMLGPIA GAIAGAWVYN IIRFTDKPLR EITKSGSFLK SKSLLRNPSN
//
LOCUS       StNIP1-3       277 aa
DEFINITION  StNIP1-3     277 aa
TITLE       StNIP1-3
```

```
ORIGIN

        1 MADHQINVNG NINHGVSLNI KEDHDLNNHK ESSSTSSFLT VPFIQKIIAE MIGTYFLIFA
       61 GCGSVVVNAD KGMITFPGIA ITWGLVVMVM VYSVGHISGA HFNPAVTIAF ASVKRFPWKQ
      121 VPAYVAAQVL GATLASGTLR LIFNGKHDHF AGTLPSGTDF QSFVVEFIIT FYLMFVISGV
      181 ATDNRAIGEL AGLAVGATIL LNVMFTGPIS GASMNPARSL GPAIVSSHYK GLWIYLVSPT
      241 LGAIAGAWVY NIIRFTDKPL REITKSGSFL KSKNSST
//
LOCUS       StNIP1-4       272 aa
DEFINITION  StNIP1-4     272 aa
TITLE       StNIP1-4
ORIGIN

        1 MSTKDIREIE EGNCSNYTNN ASGEDSSLCT SPEVVIVIQK VIAEVIGTYF LIFVGCGAVA
       61 VDKTYGSVTF PGICVAWGLI VMVMVYSVGH ISGAHFNPAV TIAFAIFRHF PFKQVPLYIM
      121 AQLVGAILGS GTLYLLLDLK TQAFFGTTPV GSNVQSLILE FIISYLLMFV ISGVATDNRS
      181 IGELAGIAIG MTILLNVLIA GPVSGASMNP ARSIGPAIVM HHYKGLWVYI IGPILGTICG
      241 AFTYNLIRFT EKPLKELTLT KTTTFLKSMS RK
//
LOCUS       StNIP1-5       347 aa
DEFINITION  StNIP1-5     347 aa
TITLE       StNIP1-5
ORIGIN

        1 MEEIPEIEGI RSTSLRINDC PSPLPSAITT SATPQKHLKC FMSVHFVQKL IAEFVGTYML
       61 IFAGCAAIIL NINKNNVVTL PGIASVWGLV VMVLIYSVGH VSGAHFNPAV TIAFATSKMF
      121 PWIQVPAYIL VQVVGSILAS GSLRLIFNGK EDQFVGTVPA GTNLQALILE FIATFYLMFV
      181 IAGVATDDRA MKHLSGVAIG ATVSLDILFS GPLTGASMNP ARSLGPAIVT GHYKGLWIYI
      241 IGPTLGAIFG AWTYNLMRLK NKSWGEAVKE LSHSQKAIEV SSKDKVICNC GEGWSCVVSK
      301 TEEAEVGNIF FECAEGCLCI VDETNTLKKH VYVYEKTKRR KSYKMHI
//
LOCUS       StNIP1-6       260 aa
DEFINITION  StNIP1-6     260 aa
TITLE       StNIP1-6
ORIGIN

        1 MASITSIIST TSSKNGIVAD FTSMEEGKHG TVQSPFLSAF QKIIAELVGT YIFIFVGCGS
       61 ALVDRERTLT IVGIALAWGL SLMALIYTLG HVSGAHFNPA VTIAFAAARK LPLMQVPIYV
      121 LPQFLGSTLA SLTLRVLFNH QGDILPMLTQ YKSPVTDFEA IFWEFLMTLI LMFVICGAAT
      181 DDRATKEIAG VAIGVTLVFE VLIAGPITGA SMNPARSLGP AIVSGVYKNQ WVFVIAPILG
      241 AMTATGIYSL LRQPKQNTKI
//
LOCUS       StNIP2-1       178 aa
DEFINITION  StNIP2-1     178 aa
TITLE       StNIP2-1
ORIGIN

        1 MLFVKVPVYA AAQITGAISA AFTLRVLLHP VIKHVGTTTP SGSDIQALIM EIVVTFSMMF
       61 ITSAVATDTK AIGELAGIAV GSAVCITSIL AGPVSGGSMN PARTIGPAMA SNDYRAIWVY
      121 IIGPVCGTLL GAWSYNFIKV NDKPVQAIAP GHSFSFKLRR MKSNHDEEQC VNKDPLND
//
LOCUS       StNIP3-1       306 aa
DEFINITION  StNIP3-1     306 aa
TITLE       StNIP3-1
```

```
ORIGIN

        1 MDPEEGVSAP STPATPGTPG VPLFGGLIKH ERRNGGNGKK SFLKSCKCFS VEPWASEEGT
       61 LPAVSCMLPP PPVSLARKVG AEFIGTLILI FAGTATAIVN QKTQGSETLF GLAASTGLAV
      121 MIVILSTGHI SGAHLNPAVT IGFAALKHFP WKHVPVYIGA QIIASFCAAF TLKVVLHPIM
      181 GGGVTIPSGS YVQAFALEFI ISFNLMFVVT AVATDTRAVG ELAGIAVGAT VMLNILIAGE
      241 TTGASMNPVR TLGPAVAVGN YKAIWIYLTA PILGALIGAG VYSAVKLPDE DGDNHPKPSL
      301 EHSFRR
//
LOCUS       StNIP3-2       296 aa
DEFINITION  StNIP3-2     296 aa
TITLE       StNIP3-2
ORIGIN

        1 MAELENGISA PATPGTPTPL FPSLRVDSMG SYDRKSMPRC KCLPLDAATW GAPHTCLADF
       61 PAPDVSLTRK LGAEFVGTFI LIFAATAGPI VNQKYNGAES LIGNAACSGL AVMIVILSTG
      121 HISGAHLNPS LTIAFAALRH FPWVQVPAYV AAQVSASICA SFALKGVFHP FMSGGVTVPS
      181 VNTGQAFALE FLITFNLLFV VTAVATDTRA VGELAGIAVG ATVMLNILVA GPSSGASMNP
      241 VRTLGPAVAA GNYKSLWIYL VAPTLGAIAG AAVYTLVKLR GDDTTETPSQ VRSFRR
//
LOCUS       StNIP3-3       287 aa
DEFINITION  StNIP3-3     287 aa
TITLE       StNIP3-3
ORIGIN

        1 MIMKLPSYEN GLSVEFQVDA STSEQSTYDQ ETTSSNVEML ERRNVCNSIL GIDPIFLRMV
       61 LAEALGTFLL MFCICGMMAS MEIMGVQVGL MEYATTAALT VVVVVFSIGP ISGAHINPAV
      121 TLAFAAVGHF PWSKVPLYVV AQVGGSIVAT YTGKMVYGLK AEFVTTKPLH SCTSAFFVEL
      181 LATFIVLFLS ASLTNYDPQS TGPLSGFLVG VAIGLAVLIS GPVSGGSMNP ARSLGPAIVA
      241 WKFNNLWIYV IAPIIGAVAG VVFYRFLRIQ GWSCKPNSTP TTTHQQI
//
LOCUS       StNIP4-1       267 aa
DEFINITION  StNIP4-1     267 aa
TITLE       StNIP4-1
ORIGIN

        1 MVSNKEDQIT QNMEEGNVQV ASNNKVGFCS SPAVVVLGQK LIAEVIGTYF VIFAGCGSVV
       61 VNKLYGGTVT FPGICVTWGL IVMVMAYTVG HISGAHFNPA VTITFSVFGR FPWKEVPLYI
      121 IAQLMGSTLA SGTLSLMFDV TPQAYFGTVP TGSNVQSLAA EIIISFLLMF VISGVGTDER
      181 AIGHVSGIAV GMTITLNVFV AGPISGASMN PARSIGPALV RHVYKGLWVY IVGPIVGTLA
      241 GAFVYNLIRS TDKPLNELTK SVSSLRS
//
LOCUS       StPIP1-1       286 aa
DEFINITION  StPIP1-1     286 aa
TITLE       StPIP1-1
ORIGIN

        1 MAENKEEDVK LGANKFRETQ PLGTSAQTDK DYKEPPPAPL FEPGELSSWS FYRAGIAEFM
       61 ATFLFLYITI LTVMGLKRSD SLCSSVGIQG IAWAFGGMIF ALVYCTAGIS GGHINPAVTL
      121 GLFLARKLSL TRSVFYIVMQ CLGAICGAGV VKGFMVGPYQ RLGGGANVVA PGYTIGSGLG
      181 AEIIGTFVLV YTVFSATDAK RNARDSHVPI LAPLPIGFAV FLVHLATIPI TGTGINPARS
      241 LGAAIIFNDK HAWRDHWIFW VGPIIGAILA AAYHQIIIRA MPFRRS
//
LOCUS       StPIP1-2       287 aa
```

```
DEFINITION  StPIP1-2     287 aa
TITLE       StPIP1-2
ORIGIN

        1 MAENKEEDVN LGANKFREPQ PLGTSAQTDK DYNEPPAAPL FEPGELSSWS FYRAGIAEFM
       61 ATFLFLYITI LTVMGLKRSD SLCSSVGIQG VAWAFGGMIF ALVYCTAGIS GGHINPAVTF
      121 GLFLARKLSL TRAVFYIVMQ CLGAICGAGV VKGFMQGPYQ RFGGGANVVQ PGYTKGDGLG
      181 AEIIGTFVLV YTVFSATDAK RNARDSHVPI LAPLPIGFAV FLVHLATIPI TGTGINPARS
      241 LGAAIIFNQD QAWDDHWIFW VGPFIGAALA AVYHQIIIRA IPFKSRA
//
LOCUS       StPIP1-3       288 aa
DEFINITION  StPIP1-3     288 aa
TITLE       StPIP1-3
ORIGIN

        1 MAENKEEDVN LGANKYRETQ PLGTAAQTDK DYKEPPPAPL FEPGELSSWS FYRAGIAEFM
       61 ATFLFLYITI LTVMGLKRSD SLCSSVGIQG VAWAFGGMIF ALVYCTAGIS GGHINPAVTF
      121 GLFLARKLSL TRAVFYIVMQ CLGAICGAGV VKGFMVGPYE RLQGGANFVS PGYTKGDGLG
      181 AEIIGTFVLV YTVFSATDAK RSARDSHVPI LAPLPIGFAV FLVHLATIPI TGTGINPARS
      241 LGAAIIYNKD QAWDDHWIFW VGPFIGAALA AVYHQIIIRA IPFKSSRS
//
LOCUS       StPIP1-4       285 aa
DEFINITION  StPIP1-4     285 aa
TITLE       StPIP1-4
ORIGIN

        1 MEGKEEDVKV GANKYSERQP LGTSAQSKDY KETPPAPFFE AGELHSWSFW RAGIAEFMAT
       61 FLFLYITVLT VMGYSRANNK CSTVGVQGIA WAFGGMIFAL VYCTAGISGG HINPAVTFGL
      121 FLARKLSLTR AVFYIVMQCL GAICGAGVVK GFQPSLFETK GGGANVVAHG YTKGDGLGAE
      181 IIGTFVLVYT VFSATDAKRN ARDSHVPILA PLPIGFAVFL VHLATIPITG TGINPARSLG
      241 AAIVYNKEHA WDDHWIFWVG PFIGAALAAL YHQVIIRAIP FKSGN
//
LOCUS       StPIP1-5       286 aa
DEFINITION  StPIP1-5     286 aa
TITLE       StPIP1-5
ORIGIN

        1 MEHREEDVRL GANKYSERQA IGIAAQSQDK DYKEPPPAPL FEPGELMSWS FYRAGIAEFV
       61 ATFLFLYITV LTVMGVSKSD SKCSTVGIQG IAWAFGGMIF ALVYCTAGIS GGHINPAVTF
      121 GLFLARKLSL TRAVFYMVMQ CLGAICGAGV VKAFGKTLYQ TKGGGANVVN VGYTNGDGLG
      181 AEIIGTFVLV YTVFSATDAK RSARDSHVPI LAPLPIGFAV FLVHLATIPI TGTGINPARS
      241 LGAAVIYNNE HAWNDHWIFW VGPFIGAALA ALYHQVVIRA IPFKSK
//
LOCUS       StPIP2-1       307 aa
DEFINITION  StPIP2-1     307 aa
TITLE       StPIP2-1
ORIGIN

        1 MGKDIEVGTE YAPKDYQDPP PAPLIDPEEL GKWSFYRAII AEFIATLLFL YITVLTVIGY
       61 NSQNSTDQCG GVATLLFLYI TVLTVIGYNS QNSTDQCGGV GILGIAWAFG GMIFVLVYCT
      121 AGISGGHINP AVTFGLFLAR KVSLVRAIMY IVAQCLGAIC GCGLVKSFQK AYYVQYGGGA
      181 NKLNDGYSTG TGLGAEIIGT FILVYTVFAA TDPKRNARDS HVPVLAPLPI GFAVFMVHLA
      241 TIPVTGTGIN PARSFGAAVI YGHHKAWNDQ WIFWVGPFIG AAIAAFYHQF ILRAGAVKAL
      301 GSFRSNA
```

```
//
LOCUS       StPIP2-2       196 aa
DEFINITION  StPIP2-2     196 aa
TITLE       StPIP2-2
ORIGIN

        1 MIFVLVYCTA GISGGHINPA VTFGLFLARK VSLVRAIMYI VAQCLGAICG CGLVKSFQKA
       61 YYVQYGGGAN KLNDGYSTGT GLGAEIIGTF ILVYTVFAAT DPKRNARDSH VPILAPLPIG
      121 FAVFMVHLAT IPVTGTGINP ARSFGAAVIY GDHKAWNDQW IFWVGPFIGA AIAAFYHQFI
      181 LRAGAVKALG SFRSNA
//
LOCUS       StPIP2-3       279 aa
DEFINITION  StPIP2-3     279 aa
TITLE       StPIP2-3
ORIGIN

        1 MGKDIEVGTE YAPKDYQDPP PAPLIDPEEL GKWSFYRAII AEFIATLLFL YITVLTVIGY
       61 KSQNSTDQCG GVGILGIAWA FGGMIFVLVY CTAGISGGHI NPAVTFGLFL ARKVSLVRAI
      121 MYIVAQCLGA ICGCGLVKCF QKAYYVKYGG GANMLNDGYS TGTGLGAEII GTFVLVYTVF
      181 AATDPKRNAR DSHVPVLAPL PIGFAVFMVH LATIPVTGTG INPARSFGAA VIYGHHKAWN
      241 DQWIFWVGPF IGAAIAAFYH QFILRAGAVK ALGSFRSNA
//
LOCUS       StPIP2-4       281 aa
DEFINITION  StPIP2-4     281 aa
TITLE       StPIP2-4
ORIGIN

        1 MAKDMEYGND QYAPSKDYQD PPPAPLIDPE ELGKWSFYRA IIAEFIATLL FLYITVLTVI
       61 GYKSQSDGDQ CGGVGILGIA WAFGGMIFVL VYCTAGISGG HINPAVTFGL FLARKVSLVR
      121 AIFYIVAQCL GAICGCGLVK LFQKAYYVKY GGGANELAVG YNTATGLGAE IIGTFVLVYT
      181 VFSATDPKRN ARDSHVPVLA PLPIGFAVFM VHLATIPITG TGINPARSFG AAVIYGKNKS
      241 WDDQWIFWVG PFIGAAIAAL YHQYILRAGA SKSINSFRSN A
//
LOCUS       StPIP2-5       287 aa
DEFINITION  StPIP2-5     287 aa
TITLE       StPIP2-5
ORIGIN

        1 MTKEVEAVHE QAAEYSAKDY TDPPPAPLID FEELTKWSLY RAAIAEFIAT LLFLYITVLT
       61 VIGYKHQADV NAGGDVCGGV GLLGIAWAFG GMIFVLVYCT AGISGGHINP AVTFGLFLAR
      121 KVSLIRAVLY MVAQCLGAIC GVGFVKAFQS AYYNRYGGGV NVMAPGHTKG VGLAAEIIGT
      181 FVLVYVVFSA TDPKRSARDS HVPVLAPLPI GFAVFMVHLA TIPITGTGIN PARSFGAAVI
      241 FNGDKAWDEH WIFWVGPFIG AFIAAVYHQY ILRAGAIKAL GSFRSNA
//
LOCUS       StPIP2-6       286 aa
DEFINITION  StPIP2-6     286 aa
TITLE       StPIP2-6
ORIGIN

        1 MTKEVESVTE RGEFSAKDYT DPPPAPLVDF EELTQWSFYR AIIAEFIATL LFLYITILTV
       61 IGYQHQADVD AGGDVCGGVG ILGIAWAFGG MIFILVYCTA GISGGHINPA VTFGLFLARK
      121 VSLIRAVLYM VAQCLGAICG VGFVKAFQSA YYNRYGGGVN VMAAGHNKGV GLGAEIIGTF
      181 VLVYTVFSAT DPKRNARDSH VPVLAPLPIG FAVFMVHLAT IPITGTGINP ARSFGAAVIY
      241 NGDKAWDEHW IFWVGPFIGA FIAAFYHQFV LRAGAIKALG SFRSNA
```

```
//
LOCUS       StPIP2-7       251 aa
DEFINITION  StPIP2-7     251 aa
TITLE       StPIP2-7
ORIGIN

        1 MVKDYVDPPS ALLFDTAELN NWSFYRALIA EFVATLLFLY VSVATVIGHK KQVGPCDGVG
       61 LVGIAWAFGG MIFVLVYSTA GISGGHINPA VTFGLLLARK VSLLRAVAYM VAQCLGAICG
      121 VGLVKGVMKD DYNTHGGGAN TVAVGYSTGA ALGAEVIGTF LLMYTVFSAT DAKRKARDSH
      181 VPVLAPLPIG FSVFMVHLAT IPITGTGINP ARSFGAAVIY NDTTAWNDHW IFWVGPFLGA
      241 LAAVIYHQQI L
//
LOCUS       StPIP2-8       283 aa
DEFINITION  StPIP2-8     283 aa
TITLE       StPIP2-8
ORIGIN

        1 MSKEVIEEGQ VQQHGKDYVD PPPAPLLDFA ELKLWSFHRA LIAEFIATLL FLYVTVATVI
       61 GHNKLNGADK CDGVGILGIA WAFGGMIFVL VYCTAGISGG HINPAVTFGL FLARKVSLVR
      121 AVGYIIAQCL GAICGVGFVK AFMKHPYNAF GGGANFVQSG YSNGTALGAE IIGTFVLVYT
      181 VFSATDPKRS ARDSHIPVLA PLPIGFAVFM VHLATIPITG TGINPARSFG AAVIADNKNV
      241 WDDQWIFWVG PFVGALLAAA YHQYILRAAA IKALGSFRSN ATN
//
LOCUS       StPIP2-9       283 aa
DEFINITION  StPIP2-9     283 aa
TITLE       StPIP2-9
ORIGIN

        1 MSKDVIEEGQ AHHHGKDYVD PPPAPLLDMA ELTKWSFYRA VIAEFIATLL FLYVTVATVI
       61 GHKKLNKLNQ CDGVGILGIA WAFGGMIFVL VYCTAGISGG HINPAVTFGL FLARKVSLIR
      121 AVAYIIAQSL GAICGVGFVK LFMKHYYNAE GGGANFVQPG YNKGTALGAE IIGTFVLVYT
      181 VFSATDPKRS ARDSHVPVLA PLPIGFAVFM VHLATIPITG TGINPARSFG AAVIYGNEKI
      241 WDDQWIFWVG PMVGAMAAAI YHQFVLRAGA VKALGSFRSN QTN
//
LOCUS       StPIP2-10       275 aa
DEFINITION  StPIP2-10     275 aa
TITLE       StPIP2-10
ORIGIN

        1 MRNEVSSALP EKSSSPAKDY HEPPPAPFIG AAELKKWALY RALIAEFVAT LLLLYIGMLT
       61 IMGNVSERAK NDDPCSGVGL LGIAWALGGM VFILVYCTAG ISGGHINPAV TFGLFLSRKI
      121 SLIRGLLYII VQYLGAICGA ALVKAIYKSK YEHYGGGVNL IAPGYTKGVA WSAEMIGTFV
      181 LVYTVLSATD SKRNARDSHV PVLAPLPIGF AVFLVHLATI PITGTGINPA RSLGAAVIYN
      241 KEIAWKDLGI FSGGPFTGAI IAAIYQLILR GCKWR
//
LOCUS       StSIP1-1       243 aa
DEFINITION  StSIP1-1     243 aa
TITLE       StSIP1-1
ORIGIN

        1 MGVVKAAVAD FVMTFIAIFC VSTIGVLTYI IGSAFGIAPG LASLSITIVI VFLLFLMLSV
       61 IAEALGGAAF NPAGTAAFYA AGVGNDSLFS VAARFPAQAA GAVAGAVAIL EVIPTQYKHM
      121 LGGPSLKVDL HNGAIAEGIL TFVMTFLVFI IVLKGPKSAL LKNWLLAMST VTMVVAGSKY
      181 TGPSMNPANA FGWAYINNMH NTWEQFYVYW ICPFVGAILA AWTFRAVFPA PAKKKKPQKK
```

```
      241 KRN
//
LOCUS       StSIP1-2       239 aa
DEFINITION  StSIP1-2     239 aa
TITLE       StSIP1-2
ORIGIN

        1 MGVIKSAIAD GLLTFLWVFC SSNIGVSTYF IASYFGVANE IASLFITTLI VFLIFLVFGF
       61 LGDVLGGAGF NPTGNAAFYA AGLGDDSLVS AAVRCPAQVV GAVAGSLALM ELMPKHYHHM
      121 LDGPALKVDV QTGAIAEGVL TFVITFMIFV IVLRGPESTL LKNLLLTMVT LPLVLAGSNY
      181 TGPSMNPANA FGWAYLSNTH NTWEHFYVYW ISPFIGAILA AWIFRALFPP PVEQKQKRN
//
LOCUS       StTIP1-1       253 aa
DEFINITION  StTIP1-1     253 aa
TITLE       StTIP1-1
ORIGIN

        1 MPISRIAIGN LAEATKPDAL KAATAEFFSM LIFVFAGSGS GMAFGKLTNG GAATPAGLIS
       61 ASIAHAFALF VAVSVGANIS GGHVNPAVTF GAFVGGHITL FRSVLYWIAQ LLGSVVACVL
      121 LKFSTGGLET SAFALSSGVT PWNAVVFEIV MTFGLVYTVY ATAVDPKKGD LGIIAPIAIG
      181 FIVGANILAG GAFDGASMNP AVSFGPAVVS WTWDNHWVYW LGPFGGAAIA ALLYEIIFIG
      241 QNTHEQLPCT DDY
//
LOCUS       StTIP1-2       250 aa
DEFINITION  StTIP1-2     250 aa
TITLE       StTIP1-2
ORIGIN

        1 MPINQITIGS HEELRHPGAL KAALAEFIST LIFVFAGQGS GMAFNKLTDG VATPAGLISA
       61 SIAHAFGLFV AVSVGANISG GHVNPAVTFG AFVGGNITLF RGILYIIAQL LGSTAACALL
      121 EFATGGMSTG SFALSAGVSV WNAFVFEIVM TFGLVYTVYA TAVDPKKGDL GVIAPIAIGF
      181 IVGANILAGG AFTGASMNPA VSFGPSLISW TWTHQWVYWA GPLIGGGLAG FIYEFIFISH
      241 THEQIPSGDF
//
LOCUS       StTIP1-3       248 aa
DEFINITION  StTIP1-3     248 aa
TITLE       StTIP1-3
ORIGIN

        1 MPISRIAIGR TEEATHPDAL KAALAEFIST LIFVFAGSGS GVAFSKLTGG GANTPTGLIA
       61 AAIAHAFGLF VAVSVGANIS GGHVNPAVTF GAFVGGNITL LRGILYWIAQ LLGSVVACLL
      121 LKFTTGGMEI GAFSLSDGVG VGNALVLEIV MTFGLVYTVY ATAVDPKKGS LGTIAPIAIG
      181 FIVGANILVG GAFDGASMNP AVSFGPAVVS WTWNNHWVYW VGPLIGGGLA GLIYEFFFIN
      241 QTHEPLPQ
//
LOCUS       StTIP2-1       249 aa
DEFINITION  StTIP2-1     249 aa
TITLE       StTIP2-1
ORIGIN

        1 MPCIAFGRFD DSFSGGSIKA YVAEFISTLL FVFAGVGSAI AYNKLTADAA LDPAGLVAVA
       61 VCHGFALFVA VSIGANISGG HVNPAVTFGL ALGGQITLLT GLFYWVAQLL GAIVACCLLK
      121 FVTGGLAVPI HGVAAGVGAT EGVVMEIIIT FALVYTVYAT AADPKKGSLG TIAPIAIGFI
      181 VGANILAAGP FSGGSMNPAR SFGPAVASGN FAGNWIYWVG PLIGGGLAGL IYSNVFMNYG
```

```
      241 DHVPLSSDF
//
LOCUS       StTIP2-2       248 aa
DEFINITION  StTIP2-2     248 aa
TITLE       StTIP2-2
ORIGIN

        1 MPCIAFGRFD DSFSLGSIKA YIAEFISTLL FVFAGVGSAI AYNKVTADAA LDPSGLVAVA
       61 VCHGFALFVA VAIAANISGG HVNPAVTFGL ALGGQITVLT GLFYWIAQLL GAIVASYLLK
      121 VVTGGMAVPI HGVAAGVGAA EGVVMEIIIT FALVYTVYAT AADPKNGALG TIAPIAIGFI
      181 VGANILAAGP FSGGSMNPAR SFGPAVVSGN FAGIWIYWVG PLVGGGLAGL IYSNVFMNHE
      241 HAPLSGDF
//
LOCUS       StTIP2-3       249 aa
DEFINITION  StTIP2-3     249 aa
TITLE       StTIP2-3
ORIGIN

        1 MAGGVAIGSF SDSFSVVSLK AYLAEFISTL IFVFAGVGSA IAYSKLTANA ALDPAGLVAI
       61 AVCHGFALFV AVSVSANISG GHVNPAVTCG LTFGGHITFI TGSFYMLAQL TGAAVACFLL
      121 KFVTGGCAIP THGVGAGVGI IEGLVMEIII TFGLVYTVFA TAADPKKGSL GTIAPIAIGF
      181 IVGANILAAG PFSGGSMNPA RSFGPAMATG NFEGFWIYWI GPLVGGSLAG LIYTNVFMQQ
      241 EHAPLSNEF
//
LOCUS       StTIP2-4       250 aa
DEFINITION  StTIP2-4     250 aa
TITLE       StTIP2-4
ORIGIN

        1 MVKIAFGSIG DSLSVGSLKA YLAEFIATLL FVFAGVGSAI AYNKLTSDAA LDPAGLVAIA
       61 VAHAFALFVG VSMAANISGG HLNPAVTLGL AVGGNITILT GLFYWVAQLL GSTVACLLLK
      121 YVTNGLAVPT HGVAAGMSGA EGVVMEIVIT FALVYTVYAT AADPKKGSLG TIAPMAIGFI
      181 VGANILAAGP FSGGSMNPAR SFGPAVVAGD FSQNWIYWVG PLIGGGLAGF IYGDVFIGSH
      241 TPLPTSEDYA
//
LOCUS       StTIP3-1       260 aa
DEFINITION  StTIP3-1     260 aa
TITLE       StTIP3-1
ORIGIN

        1 MAMPARRYAF GRADEATHPD SMRATLSELL STFIFVFAGE GSVLAIDKLY PDTGLGSSRL
       61 IVIALAHAFS FFAAVASSLN VSGGHINPAV TFGSLVGGRI SVVRAIYYWV AQLFGSVLAS
      121 LLLRLATDGL RPRGFSVAAG VGNLNALVME IVMTFGLMYT VYATAVDPRR GSLSTIAPLA
      181 IAFILGANTL VGGPFEGASM NPARAFGPAL VGWRWRNHWI YWLGPFVGAA LAGLIYEYGI
      241 IQHETVPRPT THQPLAPEDY
//
LOCUS       StTIP3-2       259 aa
DEFINITION  StTIP3-2     259 aa
TITLE       StTIP3-2
ORIGIN

        1 MQPRRYEFGR ADEATHPDSM RATLSEFLST FIFVFAGEGS VLALDKLYPD RGLGASRLTA
       61 IALAHAFSLF AAVASSMNVS GGHINPAVTF GALVGGRVSV LRAIYYWIGQ LLGAVVASAL
      121 LRLATDGLRP VGFAVAPGVG NGNALVMEIV MTFGLVYTVY ATAIDPKRGS LGIIAPLAIA
```

```
      181 FIVGANVLVG GPFEGASMNP ARAFGPALVG WRWRNHWIYW LGPFIGAAIA GIIYEFGLIQ
      241 AHDEAPVHTH HQPLAPEDY
//
LOCUS       StTIP4-1       247 aa
DEFINITION  StTIP4-1     247 aa
TITLE       StTIP4-1
ORIGIN

        1 MAKIAIGSSR EAIQPDCIQA LIVEFICTFL FVFAGVGSAM AANKLNGDPL VSLFFVAMAH
       61 ALVVAVTISA GFRISGGHLN PAVTLGLCMG GHITVFRSIL YWIDQLLASV AACALLNYLT
      121 DGMITPVHTL ANGMSYGQGL IMEVILTFSL LFTVYTTLVD PKKGILEGMG PLLTGLVVGA
      181 NIMAGGPFSG ASMNPARSFG PALVSGIWTD HWVYWIGPLI GGGLAGFICE NFFIVRSHVP
      241 LPSEETF
//
LOCUS       StTIP5-1       251 aa
DEFINITION  StTIP5-1     251 aa
TITLE       StTIP5-1
ORIGIN

        1 MASLASRLQH SVTPNALRSY LAEFLSTFFF VFAAAGAAMS TRKMVPDATS DPSSLVAIAV
       61 ANAFALSVAV YISANISGGH VNPAVTFGMA VGGHISIPMS IFYWISQMIG SVTACLLLKF
      121 TNQQVPTHGI PQEMTGFGAA VLEGVMTFGL VYTVYAAADP RRCVHAAIGP LAIGLMLGAN
      181 VMASGPFTGG SMNPAYSFGS AVVKGSFRNQ AVYWIGPFIG AAIAGLVYDN VVFPLQVTES
      241 LRGIGGGIVA V
//
LOCUS       StXIP1-1       326 aa
DEFINITION  StXIP1-1     326 aa
TITLE       StXIP1-1
ORIGIN

        1 MASNSNVVFG DEESQLSGGT NRVQPCSSTP RNRNTIDDDG KKPNFLSLSQ RLGIQDFFCL
       61 DVWRASMGEL LGSAVLVFML DTIVISTFES ETKMPNLIMS ILIAVVITIL LLAVVPVSGG
      121 HINPVISFSA ALVGIISMSR AIIYIVAQCL GAILGALALR AVVSSSIEDT FSLGGCTVTI
      181 IAPGPNGPVT KGLETAQALW LEIFCTFVFL FASIWMAYDH RQAKALGHVT VLSIVGVVLG
      241 LLVFISTTVT AKKGYGGAGI NPARCLGPAI IRGGHLWDGH WIFWVGPTIG CVAFYVYTKI
      301 IPSKHFLAEY GFKHDFVGVV KALSNV
//
LOCUS       StXIP1-2       328 aa
DEFINITION  StXIP1-2     328 aa
TITLE       StXIP1-2
ORIGIN

        1 MASKNNVVFG DEENQLSGGT NRVQTCSSTP RNRNTIDDEG KKPNFLSFSE RLGVPDFFCL
       61 DVWRASMGEL LGTAVLVFML DTIVISTFES DTKMPNLIMS ILIAVVITIL LLAVVPVSGG
      121 HINPVISFSA ALVGIISMSR AIIYIMAQCI GAILGALALR AVVSSSIEDT FSLGGCTVTI
      181 IAPGPNGPIT LGLETAQALW LEIFCTFVFL FASIWMAYDH RQAKALGHVT VLSIVGVVLG
      241 LLVFISTTVT GKKGYGGAGI NPARCLGPAI IRGGHLWDGH WIFWVGPTIG CVAFYVYTKI
      301 IPAKHFNAEY GYKHDFVGVV KALFGSNV
//
LOCUS       StXIP1-3       262 aa
DEFINITION  StXIP1-3     262 aa
TITLE       StXIP1-3
ORIGIN
```

```
        1 MGELLGTAVL VFMLDTIVIS TLESDTKMPN LILSILAAVI ITILLLAVVP VSGGHINPVI
       61 SFSAALVGII SMSRAIIYIM AQCVGAILGA LALKAVVSST IEGTFSLGGC TVTVIAPGPN
      121 GPVTVGLETA QALWLEIFCT FVFLFASIWM AYDHRQAKAL GHVTVLSIVG VVLGLLVFIS
      181 TTVTAKKGYA GAGINPARCF GPAIVRGGHL WDGHWIFWVG PTIGCVAFYV YTKIIPTKHF
      241 NAEYEYKHDF VGVVKALFGS NV
//
LOCUS       StXIP1-4       183 aa
DEFINITION  StXIP1-4     183 aa
TITLE       StXIP1-4
ORIGIN

        1 MAQCLGAVLG ALALKAVVNS TIEDNFSLGG CTITVIAPGP NGPVTLALET AQALWLKIFC
       61 TFVLFFASIW MAYDHRQAKA LGLVTALSIV GLVLGLLVFI STTVTSKRGY AGVGMNPARC
      121 LGPAIVRGGH LWDGHWIFWI GPTISGVAFY VYTEIIPPKH FQAKYGYKHD FVGVVKDLFG
      181 SNV
//
LOCUS       StXIP1-5       248 aa
DEFINITION  StXIP1-5     248 aa
TITLE       StXIP1-5
ORIGIN

        1 MLDTIVISTL ESDVKMPNLI MSILIAITLT ILILAVFPVS GGHISPVITF SSALVGLISM
       61 SRAIIYIVAQ CIGAILGALA LKAVLSSTIE QRFSLGGCTV TVVTPGLNGP EIVGLEIAQA
      121 FWLEFMCTFA LLFGSLWMAY DHRQSKALGL ITVMSIVGLL AGILVFISTS VTAKKGYAGA
      181 GMNPARCFGA AFVRGGHLWN GHWIFWVGPG LACFAFYFYI KIIPSNHFQT DGYKYDFLAI
      241 IEALFNQR
//
LOCUS       VvNIP1-1       181 aa
DEFINITION  VvNIP1-1     181 aa
TITLE       VvNIP1-1
ORIGIN

        1 MIVMVMIYTL GHVSGGHFNP AVTIAFAASR NCYLKIVPPY VLSQVAGSSL AILALFVMLN
       61 TSIPICATVT QFSSPTTIPE AFTWEFIISF ILMLAICGVA TDSRAINELS GVTVGATVLV
      121 NVLLAGPITG ASMNPARSIG PALVSMEFDC LWIYIVAPIL GTTTATVIYS FVRLPLPEKR
      181 L
//
LOCUS       VvNIP1-2       181 aa
DEFINITION  VvNIP1-2     181 aa
TITLE       VvNIP1-2
ORIGIN

        1 MIVMVMIYTL GHVSGGHFNP AVTIAFAASR NCYLKIVPPY VLSQVAGSSL AILTLFVMLN
       61 TSIPICATVT QFSSPTTIPE AFTWEFIISF ILMLAICGVA TDSRAINELS GVTVGATVLV
      121 NVLLAGPITG ASMNPARSIG PALVSMEFDC LWIYIVAPIL GTTTATVIYS FVRLPLPEKR
      181 L
//
LOCUS       VvNIP1-3       187 aa
DEFINITION  VvNIP1-3     187 aa
TITLE       VvNIP1-3
ORIGIN

        1 MGIAVGWGMI VMVMIYTLGH VSGGHFNPAV TIAFAASRKF PWRQASILSS FFNCCEHRIV
       61 ASCSVSICCT RKHWPPDFFQ STTIPEAFTW EFIISFILML AIYGVATDSR AINELSGVTV
```

```
      121 GATVLVNVLL AGPITGASMN PARSIGPALV SMEFDCLWIY IVAPILGTTT ATVIYSLVRL
      181 PLPENRL
//
LOCUS       VvNIP1-4       466 aa
DEFINITION  VvNIP1-4     466 aa
TITLE       VvNIP1-4
ORIGIN

        1 MSRKPDEMTE EISKVEEGLG NAYKPKAIDE VYLCTSPAVV TITQKVIAEV IGTYFVVFAG
       61 CGSVAVNGIY GSVTFPGVAA TWGLIVLVMI YALGHISGAH FNPAVTITFA ILRRFPYWQV
      121 PLYIIGQLMG SILASGTLSF MFNIDREAYF GTVPAGSHGQ SLVLEIIITF LLMFVISGVA
      181 TDSRATGELA GIAVGMTIML NVFVAGPVSG ASMNPARSIG PALVKHVYKG LWVYVIGPII
      241 GAIAGGLTYN LIRFTEKPLS ELTKTSSLLR TISKSVPRKQ LLVEMIGTYI LIFMGCGSMV
      301 VNKIYGQVTL LGIAMTWGLT IMVIVYSIGH VSGAHFNPSI TIAFFMVGHL PYPQVPLYIT
      361 AQLIGSLLAI CAVATYSRAS GGFAGLAIGM TILLVSGASL NPARSIGPAM VKHIYTQDFG
      421 SIYLAQLLEP LQESIYRTQE EMHELLLSFA EARATVVRLK GEDRAL
//
LOCUS       VvNIP1-5       282 aa
DEFINITION  VvNIP1-5     282 aa
TITLE       VvNIP1-5
ORIGIN

        1 MGEISGANGN HEVSLNIKDS DANHNPPPPS TATKQGSTSS FSFPFVQKLI AEVLGTYFLI
       61 FAGCAAVVVN SDKDSVVTLP GISIVWGLVV MVMVYSVGHI SGAHFNPAVT IAFATCKRFP
      121 WKQVPAYVVA QVIGSTLASG TLRLIFNGKQ DHFPGTLPAG SDLQSFVIEF IITFYLMFVI
      181 SGVATDNRAI GELAGLAVGA TVLLNVMFAG PISGASMNPA RSLGPAIVSN TYRGIWIYLL
      241 APTCGAISGA WVYNIIRFTD KPLREITKSG SFLKSKSSRN GS
//
LOCUS       VvNIP2-1       294 aa
DEFINITION  VvNIP2-1     294 aa
TITLE       VvNIP2-1
ORIGIN

        1 MATTDPNLSN STSINELTTA LHHPDSQNSN PCFLWRLFLE HYPPGFLRKV VAEVIATYLL
       61 VFVTCGSAAL SASDEQRVSK LGASVAGGLI VTAMIYAVGH ISGAHMNPAV TLAFAAVRHF
      121 PWKQVPLYAA AQLTGAIGAA FTLRELLYPI KHLGTTTPSG TEIQALVMEI VVTFSMMFIT
      181 SAVATDTKAI GELAGIAVGS AVCITSILAG PVSGGSMNPA RTLGPAIASA DYKGIWVYAV
      241 GPVSGTLLGT WSYNFIRVTE KPVQAISPHS FSLKLCRMRS NAGEISSKDP LNHV
//
LOCUS       VvNIP3-1       354 aa
DEFINITION  VvNIP3-1     354 aa
TITLE       VvNIP3-1
ORIGIN

        1 MDTDHDVPSA PSTPVTPSTP GAPLFHGFKA HGTSSGNGRR SFLRSCKCFS VEQWAMEEGS
       61 LPTLSCSWPT PPLPVSLARK MGAEFIGTFM LIFGGAATGI VNQKTQGSET LLGLAASTGL
      121 AVMVIILSTG HISGAHLNPA VTIAFAALRH FPWKHVPVYI GSQLMGSLCA AFALKGIFNP
      181 VMDGGVTVPS HSGAYGQAFA LEFIISFFLM FVVTAVATDT RAVGSLAGIA VGGTVMLNIL
      241 IAGETTGASM NPVRTLGPAI AVNNFKAIWV YLTAPILGAL CGAGVYTAVK LPEEDGNTHS
      301 LNKSMTVQSE GDQSMSSVGL QHKFRNFTRK VRQFFEEIRN NSPMDRDPAS TYNT
//
LOCUS       VvNIP3-2       298 aa
DEFINITION  VvNIP3-2     298 aa
TITLE       VvNIP3-2
```

```
ORIGIN

        1 MAEAETGTPT ASAPATPGTP GGPLFSSLRV DSLSYDRKSM PRCKCLPVGA ASWAPSPTCF
       61 TDFPAPDVSL TRKLGAEFVG TFILIFAATA GPIVNQKYSG VETLIGNAAC AGLAVMIVIL
      121 STGHISGAHL NPSLTIAFAA LRHFPWVQVP AYIAAQVSAS ICASFALKAV FHPFMSGGVT
      181 VPSVSIGQAF ALEFLITFNL LFVVTAVATD TRAVGELAGI AVGATVMLNI LVAGPSSGGS
      241 MNPVRTLGPA VAAGNYRAIW IYLVAPTLGA VAGAAIYTAV KLRADEGEQP RQVRSFRR
//
LOCUS       VvNIP3-3       293 aa
DEFINITION  VvNIP3-3     293 aa
TITLE       VvNIP3-3
ORIGIN

        1 MKSLFEKQLS PGTSNNSSSS GQSRDDQELG SHAVPKNGDH VRKKNSWFCC SPPDHMDLNP
       61 ARMILAEMVG TFILVFCVYG IEAVTQLMKG EVGLLEYAVT GGLTVVVLVF SIGSISGAHV
      121 NPSVTITFAT LCQFPWSKVP YYISAQIVGS VLATYVGRSI YGIKPELITT KPLQGCSSAF
      181 WVEFIATFII MFLAVSLTSQ PQSVSHLSGF VVGIAIGLAV LITGPVSGGS MNPARSLGPA
      241 IVSWKFDDIW IYTIAPTLGA VAGGHLFHLL RLRHQPCTPN SSPNTILLSN AFQ
//
LOCUS       VvPIP1-1       190 aa
DEFINITION  VvPIP1-1     190 aa
TITLE       VvPIP1-1
ORIGIN

        1 MIFALVYCTA GISGGHINPA VTFGLLLARK LSLTRAVFYM IMQCLGAICG AGVVKGFQGH
       61 QYEVLGGGAN VVAAGYSKGD GLGAEIVGTF VLVYTVFSAT DAKRNARDSH VPILAPLPIG
      121 FAVFLVHLAT IPITGTGINP ARSLGAAIIY NREHAWDDMW IFWVGPFIGA ALAALYHQIV
      181 IRAIPFKTRA
//
LOCUS       VvPIP1-2       215 aa
DEFINITION  VvPIP1-2     215 aa
TITLE       VvPIP1-2
ORIGIN

        1 MGVKKSPTMC ASVGIQGIAW AFGGMIFALV YCTAGISGGH INPAVTFGLL LARKLSLTRA
       61 IFYIIMQCLG AICGAGVVKG FEGSQSYEVL GGGANVVNSG YTKGDGLGAE IVGTFVLVYT
      121 VFSATDAKRN ARDSHVPILA PLPIGFAVFL VHLATIPITG TGINPARSLG AAIIFNREHA
      181 WDDMWIFWVG PFIGAALAAM YQQIVIRAIP FKSRA
//
LOCUS       VvPIP1-3       213 aa
DEFINITION  VvPIP1-3     213 aa
TITLE       VvPIP1-3
ORIGIN

        1 MGVVRADSKC STVGIQGIAW AFGGMIFALV YCTAGISGGH INPAVTFGLF LARKLSLTRA
       61 VYYMVMQCLG AICGAGVVKG FEKGRYNSLG GGANVVNAGY TKGDGLGAEI VGTFVLVYTV
      121 FSATDAKRNA RDSHVPILAP LPIGFAVFLV HLATIPITGT GINPARSLGA AIIYNKDHAW
      181 DDHWIFWVGP FIGAALAALY HQVVIRAIPF KSK
//
LOCUS       VvPIP1-4       190 aa
DEFINITION  VvPIP1-4     190 aa
TITLE       VvPIP1-4
ORIGIN
```

```
        1 MIFVLVYCTA GISGGHINPA VTFGLLLARK LSLTRAVFYM IMQCLGAICG AGMVKWFQRH
       61 DFETLGGGIN AVASGYSKLA GLGAEIVGTF VLVYTVLSAT DAKRNARDSH VPILAPLPIG
      121 FAVFLVHLAT IPITGTGINP ARSLGAAIIY NRGNAWDDMW IFWVGPFIGA TLATLYHQIV
      181 IRAIPFKTRA
//
LOCUS       VvPIP1-5       429 aa
DEFINITION  VvPIP1-5     429 aa
TITLE       VvPIP1-5
ORIGIN

        1 MHGHTRRFHQ ATCSSKLSKV QALEAVDYGD ADPGGTIALY GRWRMEPLCL PCAVNGIGSC
       61 QRMSRVKWMY GRKSHWVSGR KGYFRRQHIK GTCFIHSLET NFFSKTLVWA LIISHFCHNW
      121 GIFILLTWMR YMRSLKPRSV QACWILLYPT GKRSSLCSFP LPPNGIDPKL GSVIFYLEDM
      181 EEATNQTLEA LEALVSKIFM NISSLKSAYI QLQVAHTPYE PDKIQAADSF HICRCNHHLP
      241 PHPRLPSMPA FQHQHSSQMG STGDHEIHVV EADAKDYREP PPALLFEPGE LHSRPFWRVG
      301 IAECMATFLF LYVIISAIIG VMLGGGINAV ASGYPKLAGL GAEIVGTVIL VYPVLSATDA
      361 KSNVRDSHVS NLAPLSIEFA VLLVHPATIP ITGTGINPAR SLGPPSSTTR EMPGMICGFP
      421 WLDPSLELL
//
LOCUS       VvPIP2-1       198 aa
DEFINITION  VvPIP2-1     198 aa
TITLE       VvPIP2-1
ORIGIN

        1 MIFILVYCTA GISGGHINPA VTFGLFLARK VSLIRALAYM VAQCLGAICG VGLVKAFMKS
       61 FYNSLGGGAN SVAAGYNKGT ALGAEIIGTF VLVYTVFSAT DPKRSARDSH VPVLAPLPIG
      121 FAVFMVHLAT IPITGTGINP ARSFGAAVIY NNEKVWDDQW IFWVGPFVGA LAAAAYHQYI
      181 LRAAAIKALG SFRSNPTN
//
LOCUS       VvPIP2-2       196 aa
DEFINITION  VvPIP2-2     196 aa
TITLE       VvPIP2-2
ORIGIN

        1 MIFILVYCTA GISGGHINPA VTFGLFLARK VSLVRALMYM IAQCLGAVCG VGLVKAFQSS
       61 LYNRYGGGAN ELAGGYNKGV GLGAEIIGTF VLVYTVFSAT DPKRNARDSH VPVLAPLPIG
      121 FAVFMVHLAT IPITGTGINP ARSFGAAVIY NNDKAWDDHW MFWVGPFIGA AIAAFYHQYI
      181 LRAAAIKALG SFRSNA
//
LOCUS       VvPIP2-3       198 aa
DEFINITION  VvPIP2-3     198 aa
TITLE       VvPIP2-3
ORIGIN

        1 MIFILVYCTA GISGGHINPA VTFGLFLARK VSLIRAILYM VAQCLGAICG VGLVKAFQSA
       61 YYDRYGGGAN ELSTGYSKGT GLGAEIIGTF VLVYTVFSAT DPKRSARDSH VPVLAPLPIG
      121 FAVFMVHLAT IPITGTGINP ARSLGAAVIY NNEKAWDDQW IFWVGPFIGA AIAAFYHQFI
      181 LRAGAVKALG SFRSTTHV
//
LOCUS       VvPIP2-4       163 aa
DEFINITION  VvPIP2-4     163 aa
TITLE       VvPIP2-4
ORIGIN
```

```
        1 MIFILVYCTA GISGGHINPA VTFGLFLARK VSLVRALASG YSIGTGLAAE IIGTFVLVYT
       61 VFSATDPKRN ARDSHVPVLA PLPIGFAVFM VHLATIPITG TGINPARSLG AAVIYNQPKA
      121 WSDHWVFWAG PFIGAAIAAF YHQFILRAGA VKALGSFKSS SHM
//
LOCUS       VvSIP2-1       236 aa
DEFINITION  VvSIP2-1     236 aa
TITLE       VvSIP2-1
ORIGIN

        1 MAKIRLIVSD FLLAFMWVSS GALNKLFVNR VLGWGHEPRG EIMKATLSII NMFFFAFLGK
       61 ISKGGAYNPL TVLAGAISGD FSRFLFTVGA RIPAQVIGSI TGVRLLINTF PEVGFGPRLT
      121 VDIHHGALTE GFLTFAIVMI SLGLSRNIPG SFFMKTWISS VSKLALHILG SDLTGGCMNP
      181 AAVMGWAYAR GDHITKEHIL VYWLAPMEAT LLAVWTFRLL VQPHKEEKEK MKAKSE
//
LOCUS       VvTIP1-1       213 aa
DEFINITION  VvTIP1-1     213 aa
TITLE       VvTIP1-1
ORIGIN

        1 MPIRNIAFGR PEEATRPDAL KAAFAEFICT LIFVFAGEGS GMAFNKLTDN GSTTPAGLVA
       61 ASLAHAFALF VALLGSTVAC LLLKFSTNGM TTSAFSLSSG VTVWNAFVFE IVMTFGLVYT
      121 VYATAIDPKK GNLGIIAPIA IGFIVGANIL AGGAFDGASM NPAVSFGPAL VSWTWTNHWV
      181 YWAGPLIGGG LAGLVYEVFF ISHTHEQLPS TDY
//
LOCUS       VvTIP1-2       251 aa
DEFINITION  VvTIP1-2     251 aa
TITLE       VvTIP1-2
ORIGIN

        1 MPINRIAIGT PGEASHPDAL KAALAEFFSM LIFVFAGEGS GMAFNKLTDS GSSTPAGLVA
       61 AALAHGFALF VAVSVGANIS GGHVNPAVTF GAFIGGHITL LRGILYWIAQ LLGSVVACLL
      121 LKFSTGGLET SAFSLSSGVS VWNALVFEIV MTFGLVYTVY ATAVDPKKGN LGIIAPIAIG
      181 FIVGANILAG GAFDGASMNP AVSFGPAVVS WSWANHWVYW AGPLIGAAIA AIIYDLIFID
      241 STHEQLPTTD Y
//
LOCUS       VvTIP1-3       252 aa
DEFINITION  VvTIP1-3     252 aa
TITLE       VvTIP1-3
ORIGIN

        1 MPIPRIALGS PAEAGQADAL KAALAEFISV LIFVFAGEGS GMAFNKLTDD GSSTPAGLVA
       61 AAVAHAFALF VAVSIAANIS GGHVNPAVTF GALVGGHITL FRSLLYWIAQ LLGSVVACLL
      121 LKFATGGLTT SAFSLSSGVS AWNALVFEIV MTFGLVYTVY ATAIDPKKGN IGIIAPIAIG
      181 FIVGANILAG GAFDGASMNP AVSFGPAVVS WTWSNHWVYW LGPFIGAAIA AFVYSIFYLS
      241 PTTHEQLPTT DY
//
LOCUS       VvTIP2-1       174 aa
DEFINITION  VvTIP2-1     174 aa
TITLE       VvTIP2-1
ORIGIN

        1 MAYNKLTSDA ALDPAGLVAV AVAHGFALFV AVAISANISG GHVNPAVTFG LVVGGQITIL
       61 TGILYWIAQL VGSILACFLL KLVTGGLTTP VHSLGAGVGV IDAIVFEIVI TFALVYTVYA
      121 TAVDPKKGSL GIIAPIAIGL VVGANILAAG PFSGSVYAIM YMGSDHQPLA SSEF
```

```
//
LOCUS       VvTIP2-2       219 aa
DEFINITION  VvTIP2-2     219 aa
TITLE       VvTIP2-2
ORIGIN

        1 MVKLAFGSFG DSFSVGSLKA YLSEFNATLL FVFAGVGSAI AYGKLTSDAA LDPPGLVAVA
       61 IAHAFALFVG VSIAANISGG HLNPAVTFGL AIGGHITILT GIFYVIAQCL GSIVACLLLK
      121 FATNGESIPT HGVAAGMNAI EGVVMEIVIT FALVYTVYAT AVDPKKGSLG IIAPIAIGFI
      181 VENWIYWVGP LVGGGLAGLV YGNIFIESYA SVPISDEYA
//
LOCUS       VvTIP2-3       251 aa
DEFINITION  VvTIP2-3     251 aa
TITLE       VvTIP2-3
ORIGIN

        1 MHCIKVSSLL RLGSELLNLT EELFSPTSLR SYFAEFISTF IFVFLGVGSA MSADATSAET
       61 GVLAVAVAHA FALVVAMYLA GDISDGHVNP AVTYGLVVGG HVSGLTGICY CMAQLSGSVT
      121 ACVALILAIP TTRPDPKISG LADVAIEALA TFAIVYAVYV ARDLRNGSRG IMGPIAVGFI
      181 YGANILVTAP LTGGSMNPAR SFGPAFVTGD MKKQWVYWVG PLVGGGIAGL VYESLMTTSN
      241 GQPPSSISSV G
//
LOCUS       VvTIP3-1       259 aa
DEFINITION  VvTIP3-1     259 aa
TITLE       VvTIP3-1
ORIGIN

        1 MPPRRYAFGR AEEATHPDSM RATLSELVAT FIFVFAGEGS VLALGKLYAG ATALTVPALV
       61 MVALAHGLAL FAAVSASINV SGGHVNPAVT FGALVGGRIS LLRALYYWVA QLLGAVLAAL
      121 LLRFATGGLR PVGFTVASGY DEWHAMLLEI AMTFGLVYTV YATAIDPKRG SLGVIAPLAI
      181 GFIVGANILV GGAFDGASMN PARAFGPALV GWRWRYQWVY WVGPLVGGAL AALIYEFLVI
      241 PTEPQHIHTH HQPLAPEDY
//
LOCUS       VvTIP4-1       253 aa
DEFINITION  VvTIP4-1     253 aa
TITLE       VvTIP4-1
ORIGIN

        1 MAKMALGSGR EFAQPDCIRA LVMEFIVTFL FVFAGVGSAM ATEKLKGDSL DSLFFVAMAH
       61 ALVVAVMVSA ALQISGGHLN PAVTLGLCVG GHITVVRSVL YFIDQCLAST VACILLKFLT
      121 GGRATPVHTL ASGVGCLQGV MLEFILTFSL LFTVYANIVS AQKSAHIDGL GPMITGLVVG
      181 ANVMAGGAFS GASMNPARSF GPALVSWDWT NHWVYWVGPL VGGAVAGFVY ENFFINRPHL
      241 RLPTRDEEEE EGF
//
LOCUS       VvTIP5-1       281 aa
DEFINITION  VvTIP5-1     281 aa
TITLE       VvTIP5-1
ORIGIN

        1 MAPNSLSVRL EESFTRNALR SYLAEFISTF IFVFAAVGSA MSSRKLMPDA TSDPSSLLVV
       61 ALANAFALSS AVYIAAGISG GHVNPAVTFG LAVGGHVSIP TAMLYWISQM LGATMACLFL
      121 RVTTVGQHVP THAIAEEMTG FGASVLEGVL TFALVYTVYA AGDPRRGPLG SIGPLAIGLI
      181 EGANVLAAGP FTGGSMNPAS SFGSALVGGT FKNQAVYWVG PLIGGALAGL LYENVVFPPQ
      241 DTAISQDTGI TIVKLFWLFK FLKFCQIKAK STRSSHCVHE R
```

```
//
LOCUS       VvXIP1-1       154 aa
DEFINITION  VvXIP1-1     154 aa
TITLE       VvXIP1-1
ORIGIN

        1 MGSHNGVVGD EENPHNGIRI QPVLSTPMAE QWKSEEGKKM NTTMGERLGL NELFSPQVWR
       61 ASLAELLGTA LLVFLLDTIV ISSIQTQTKT PNLIMSVVVA ITIAILLLAT IPVSGGHINP
      121 VITFSAALLG LISFSRAAVY FLAQLLLSQA QKGP
//
LOCUS       VvXIP1-2       304 aa
DEFINITION  VvXIP1-2     304 aa
TITLE       VvXIP1-2
ORIGIN

        1 MDPVVMNIVG QDSPRKVEES KATSLEGKDS SRTAFLPCII SHEFFSPEVW RASVAELLGT
       61 AVLVFVLDTI VISSYQTKTE TPNLVISFFI FPTLTILLLA TFPISGGHIN PIITFSAALV
      121 GVISHSRACV YVLAQCAGAV LGALALKAVV NSNIEETFSL GGCTLSVIVP GPDGAITVGI
      181 NTGQALWLEI ICTFVLLFAS VWIGFDDRQA KALGLVLVCS IIGAVAGVLV FVSTTVTATK
      241 GYAGAGMNPA RCLGAALVRG GQLWNGLWVF WVGPAIACLL FHVYVKTIRD LPPWTEFLSK
      301 NELP
//
LOCUS       ZmNIP1-1       284 aa
DEFINITION  ZmNIP1-1     284 aa
TITLE       ZmNIP1-1
ORIGIN

        1 MAGAEVANGV HDGALDLEEG RGGGVDGAGC ESSEQDGAGR RPMFSVPFVQ KILAEALGTY
       61 FLIFAGCAAV AVNLRTGGTV TFPGICAVWG LAVMVMVYSV GHISGAHLNP AVSLAFATCG
      121 RFPWRQVPAY AAAQVTGATA ASLTLRLLFG SAREHFFGTV PAGSDAQSLV VEFIISFNLM
      181 FVVSGVATDN RAIGELAGLA VGATVLLNVL FAGPISGASM NPARTLGPAL VVGRYAGIWV
      241 YFAGPICGTV AGAWAYNLIR FTDKPLREIT QTSSFLRSVR RSSS
//
LOCUS       ZmNIP1-2       317 aa
DEFINITION  ZmNIP1-2     317 aa
TITLE       ZmNIP1-2
ORIGIN

        1 MARREDDSYT NASVFETSVE DGRKDKSESY AVDEPPQPVD DALCGMSTSV SFIQQLIAEF
       61 LATFFLIFAG CGVIAVNDKN GMATFPGIAV VWGMVVMAMI YAVGHVSGAH INPAVPAYML
      121 VQTVAATMAS LVLRLMFGRQ HELASVTVPA PGGSIFQSLV LEFIITFYLM FVVMAVATDD
      181 RAVGQMAGLA VGGTIMLNAL FAGSEVSRFF RSIAMGRPVS GASMNPARSI GPALVSNKFR
      241 ALWVYIFGPF AGAAAGAWAY NLIRHTDKTL AEEHEPNVWE NLKETNVAKM LDVYGIIFLE
      301 HEPNVWKKLN ETDVINP
//
LOCUS       ZmNIP2-1       303 aa
DEFINITION  ZmNIP2-1     303 aa
TITLE       ZmNIP2-1
ORIGIN

        1 MSTNSRANSR ANFNNEIHDI GTAVHNSSSL PPAYYDRSLA DMFPPHLLKK VVSEVVSTFL
       61 LVFVTCGAAG IYGSDKDRIS QLGQSVAGGL IVTVMIYAVG HISGAHMNPA VTLAFAVFRH
      121 FPWIQVPFYW AAQFTGAICA SFVLKAVLHP IAVLGTTTPA GPHWHSLIIE VIVTFNMMFV
      181 TLAVATDTRA VGELAGLAVG SAVCITSIFA GAVSGGSMNP ARTLGPALAS NLYTGLWIYF
```

```
      241 LGPVLGTLSG AWTYTFIRFE EAPSKDASSS HSQKLSSFKL RRLQSQSVAA DADDDEELDH
      301 IQV
//
LOCUS       ZmNIP2-2       295 aa
DEFINITION  ZmNIP2-2     295 aa
TITLE       ZmNIP2-2
ORIGIN

        1 MSTNSRSNSR ANFNNEIHDI GTAQNSSMPP TYYDRSLADI FPPHLLKKVV SEVVSTFLLV
       61 FVTCGAAGIY GSDKDRISQL GQSVAGGLIV TVMIYAVGHI SGAHMNPAVT LAFAVFRHFP
      121 WIQVPFYWAA QFTGSICASF VLKAVLHPIA VLGTTTPTGP HWHSLVIEII VTFNMMFVTL
      181 AVATDTRAVG ELAGLAVGSA VCITSIFAGA VSGGSMNPAR TLGPALASNL YTGLWIYFLG
      241 PVLGTLSGAW TYTYIRFEEA PSHKDMSQKL SSFKLRRLQS QSVAVDDDEL DHIQV
//
LOCUS       ZmNIP2-3       294 aa
DEFINITION  ZmNIP2-3     294 aa
TITLE       ZmNIP2-3
ORIGIN

        1 MAAASTTSRT NSRVNYSNEI HDLSTVQSGS VVPTLFYPDK SIADIFPPHL GKKVISEVVA
       61 TFLLVFVTCG AASIYGEDNR RISQLGQSVA GGLIVTVMIY ATGHISGAHM NPAVTLSFAC
      121 FRHFPWIQVP FYWAAQFTGA MCAAFVLKAV LHPIAVIGTT TPSGPHWHAL LIEIVVTFNM
      181 MFVTCAVATD SRAVGELAGL AVGSAVCITS IFAGPVSGGS MNPARTLAPA VASNVFTGLW
      241 IYFLGPVIGT LSGAWVYTYI RFEEAPAAKD TQRLSSFKLR RMQSQLAADE FDTV
//
LOCUS       ZmNIP2-4       301 aa
DEFINITION  ZmNIP2-4     301 aa
TITLE       ZmNIP2-4
ORIGIN

        1 MAASTTSRTN SRVNYSNEIH DLSTVQGGSA AAAAAALFYP DSKSIADIFP PHLGKKVISE
       61 VVATFLLVFV TCGAASIYGE DNARISQLGQ SVAGGLIVTV MIYATGHISG AHMNPAVTLS
      121 FACFRHFPWI QVPFYWAAQF TGAMCAAFVL KAVLQPIAVI GTTTPSGPHW HALAIEIVVT
      181 FNMMFVTCAV ATDSRAVGEL AGLAVGSAVC ITSIFAGPVS GGSMNPARTL APAVASNVFT
      241 GLWIYFLGPV VGTLSGAWVY TYIRFEEAPA AAKPDTQRLS SFKLRRMQSQ SALAADEFDT
      301 V
//
LOCUS       ZmNIP3-1       297 aa
DEFINITION  ZmNIP3-1     297 aa
TITLE       ZmNIP3-1
ORIGIN

        1 MAEHATTGVE EQRQVAISMC SAPTPSKLVA VESSSLQKLM LKSPPQADAH GDEQQGREVP
       61 LAKKVAAEFV GTFILMFAVV STVVADAQHG GAEGLVGVAA AAGLAVVAVV LAVVSVSGSH
      121 LNPAVSLAMG VFGYLPRAHV LPYAAAQTAG SAAAAFLAKA MYRPADPAVM ATVPRVGAAQ
      181 AFFLELVLTF VLMFVIAAVA TDPTSAKQRA GGHRDRGGDN DERSHRRKSI RMNWGSDPTD
      241 ERCVCMRCRP STGPSMNPAR TIGAALATGK YKDIWVYLLA PPLGAIAGAA TYTLIKP
//
LOCUS       ZmNIP3-2       312 aa
DEFINITION  ZmNIP3-2     312 aa
TITLE       ZmNIP3-2
ORIGIN

        1 MADDGRRRNV SMDFSVSIPS AAAASMLVDK ENTSDDRISI IIPHSRSPSS KILPLGFQRN
```

```
       61 EAPSDHSPAR PVSAKRVALA LTKKVAAELL GTFLLVFTVL SALITNEAHG GALGVLGVAV
      121 AGGTAVVVVV SSIFHVSGGH VNPAVSVAMA VFGHLPPAHL ALYAAAQLLG SVAASFVAKA
      181 LYAGPANLLG PTVATVPSVG ASQAFWVEFI TTFVVLFVVT ALATDPKAVK EMVAVGAGAA
      241 VMMSALISGY MDRVTDKVST QNGGRESTGA SMNPARTLGT AIATGTYTKI WVYMVAPPLG
      301 AIAGCGAYHA LK
//
LOCUS       ZmNIP3-3       302 aa
DEFINITION  ZmNIP3-3     302 aa
TITLE       ZmNIP3-3
ORIGIN

        1 MEPGSTPPNG SAPATPGTPA PLFSSGGPRV DSLSYERKSM PRCKCLPLPA VEGWGVATHT
       61 CVVEIPAPDV SLTRKLGAEF VGTFILIFFA TAAPIVNQKY GGAISPFGNA ACAGLAVATV
      121 ILSTGHISGA HLNPSLTIAF AALRHFPWLQ VPAYVAVQAL ASVCAAFALK GVFHPFLSGG
      181 VTVPDATVST AQAFFTEFII SFNLLFVVTA VATDTRAVGE LAGIAVGAAV TLNILVAGPT
      241 TGGSMNPVRT LGPAVAAGNY RQLWIYLLAP TLGALAGASV YKAVKLRDEN GETPRTQRSF
      301 RR
//
LOCUS       ZmNIP4-1       299 aa
DEFINITION  ZmNIP4-1     299 aa
TITLE       ZmNIP4-1
ORIGIN

        1 MAAMMMDSTA EKDLAGDGAA VSGHGQDLER SCHDQEPAAA DGASSRGLAI GRFVRELMVE
       61 GVASFLLVFW SAVAALMQEM HGTLTFPMVC LVVALTVVFV LCWLGPAHFN PAVTVTFTVF
      121 GYLSWTKLPF YVAAQLAGSL LACLSANGVM EPRAEHFYGT VPMAGGDTRL PFLLELVASA
      181 LLMVVIATAA RGSNQTAGGL AIGAAVGALG LVIGPVSGGS MNPIRTLGPA IVLGRYTSVW
      241 IYLVAPVAGM LIGALCNRLV RRSDAIIAFL CGAKPRVVAP GQNRAARRWS TCVSALLAG
//
LOCUS       ZmPIP1-1       289 aa
DEFINITION  ZmPIP1-1     289 aa
TITLE       ZmPIP1-1
ORIGIN

        1 MEGKEEDVRL GANKFSERQP IGTAAQGAAD DKDYKEPPPA PLFEPGELKS WSFYRAGIAE
       61 FVATFLFLYI TILTVMGVSK STSKCATVGI QGIAWSFGGM IFALVYCTAG ISGGHINPAV
      121 TFGLFLARKL SLTRALFYII MQCLGAICGA GVVKGFQQGL YMGNGGGANV VAPGYTKGDG
      181 LGAEIVGTFI LVYTVFSATD AKRNARDSHV PILAPLPIGF AVFLVHLATI PITGTGINPA
      241 RSLGAAIIYN RDHAWNDHWI FWVGPFIGAA LAAIYHQVII RAIPFKSRS
//
LOCUS       ZmPIP1-2       292 aa
DEFINITION  ZmPIP1-2     292 aa
TITLE       ZmPIP1-2
ORIGIN

        1 MEGKEEDVRL GANKFSERQP IGTAAQGAGA GDDDKDYKEP PPAPLFEPGE LKSWSFYRAG
       61 IAEFVATFLF LYITVLTVMG VSKSTSKCAT VGIQGIAWSF GGMIFALVYC TAGISGGHIN
      121 PAVTFGLFLA RKLSLTRAIF YIIMQCLGAI CGAGVVKGFQ QGLYMGNGGG ANVVAPGYTK
      181 GDGLGAEIVG TFILVYTVFS ATDAKRNARD SHVPILAPLP IGFAVFLVHL ATIPITGTGI
      241 NPARSLGAAI IYNRDHAWSD HWIFWVGPFI GAALAAIYHQ VIIRAIPFKS RS
//
LOCUS       ZmPIP1-3       288 aa
DEFINITION  ZmPIP1-3     288 aa
TITLE       ZmPIP1-3
```

ORIGIN

```
        1 MEGKEEDVRL GANKFSERQP IGTAAQGTDD KDYKEPPPAP LFEPGELKSW SFYRAGIAEF
       61 VATFLFLYIS ILTVMGVSKS TSKCATVGIQ GIAWSFGGMI FALVYCTAGI SGGHINPAVT
      121 FGLFLARKLS LTRAVFYIIM QCLGAICGAG VVKGFQQGLY MGNGGGANVV APGYTKGDGL
      181 GAEIVGTFIL VYTVFSATDA KRNARDSHVP ILAPLPIGFA VFLVHLATIP ITGTGINPAR
      241 SLGAAVIYNQ HHAWADHWIF WVGPFIGAAL AAIYHQVIIR AIPFKSRS
```

//
LOCUS   ZmPIP1-4   288 aa
DEFINITION ZmPIP1-4   288 aa
TITLE   ZmPIP1-4
ORIGIN

```
        1 MEGKEEDVRL GANRYSERQP IGTAAQGTEE KDYKEPPPAP LFEAEELTSW SFYRAGIAEF
       61 VATFLFLYIS ILTVMGVSKS SSKCATVGIQ GIAWSFGGMI FALVYCTAGI SGGHINPAVT
      121 FGLFLARKLS LTRALFYMVM QCLGAICGAG VVKGFQEGLY MGAGGGANAV NPGYTKGDGL
      181 GAEIVGTFVL VYTVFSATDA KRSARDSHVP ILAPLPIGFA VFLVHLATIP ITGTGINPAR
      241 SLGAAIVYNR SHAWNDHWIF WVGPFIGAAL AAIYHVVIIR ALPFKSRD
```

//
LOCUS   ZmPIP1-5   296 aa
DEFINITION ZmPIP1-5   296 aa
TITLE   ZmPIP1-5
ORIGIN

```
        1 MAGGTLQDRS EAEDVRVGVD RFPERQPIGT AADDLGRDYS EPPAAPLFEA SELSSWSFYR
       61 AGIAEFVATF LFLYVTVLTV MGVSKSPSKC GTVGIQGIAW AFGGMIFALV YCTAGVSGGH
      121 INPAVTFGLL LARKLSLTRA VYYVVMQCLG AVCGAGVVKA FGSALYESAG GGANAVSPGY
      181 TKGDGLGAEV VGTFVLVYTV FSATDAKRTA RDSHVPALAP LPIGFAVFLV HLATIPITGT
      241 GINPARSLGA AIIYDNPHGW HGHWIFWVGP FAGAALAAVY HQVVLRAIPF KSSAHY
```

//
LOCUS   ZmPIP2-1   290 aa
DEFINITION ZmPIP2-1   290 aa
TITLE   ZmPIP2-1
ORIGIN

```
        1 MGKDDVIESG AGGGEFAAKD YTDPPPAPLI DAAELGSWSL YRAVIAEFIA TLLFLYITVA
       61 TVIGYKHQTD ASASGADAAC GGVGVLGIAW AFGGMIFVLV YCTAGISGGH INPAVTFGLF
      121 LARKVSLVRA LLYIVAQCLG AICGVGLVKA FQSAYFDRYG GGANSLASGY SRGTGLGAEI
      181 IGTFVLVYTV FSATDPKRNA RDSHVPVLAP LPIGFAVFMV HLATIPVTGT GINPARSLGA
      241 AVIYNKDKPW DDHWIFWVGP LVGAAIAAFY HQYILRAGAI KALGSFRSNA
```

//
LOCUS   ZmPIP2-2   292 aa
DEFINITION ZmPIP2-2   292 aa
TITLE   ZmPIP2-2
ORIGIN

```
        1 MGKDDVVQSG AGGGEFAAKD YTDPPPAPLV DAAELGSWSL YRAVIAEFIA TLLFLYVTVA
       61 TVIGYKHQTD ASASGAGADA ACGGVGVLGI AWAFGGMIFV LVYCTAGISG GHINPAVTFG
      121 LFLARKVSLV RALLYMVAQC LGAVCGVGLV KAFQSAYFDR YGGGANSLAS GYSRGAGLGA
      181 EIVGTFVLVY TVFSATDPKR NARDSHVPVL APLPIGFAVF MVHLATIPVT GTGINPARSL
      241 GAAVVYNKDK PWDDHWIFWV GPLLGAAIAA FYHQYILRAG AIKALGSFRS NA
```

//
LOCUS   ZmPIP2-3   283 aa
DEFINITION ZmPIP2-3   283 aa

```
TITLE       ZmPIP2-3
ORIGIN

        1 MDTCHQSIET ADGKKDYSDP VPAPFVNAGE LGKWSLYRAV IAEFVATLLF VYVTLATVIG
       61 HKREAESQPC GSVGVLGIAW SFGGMIFVLV YCIAGISGGH INPAVTFGLL LARKLSLVRA
      121 ALYVVAQCLG AMCGAGLVKA FHGAHWYLRY GGGANELAAG YSKGAGLGAE IVGTFVLVYT
      181 VFSATDPKRK VRDSHVPVLA PLPIGFAVFM VHLATIPVTG TGINPARSLG PAVVYNQRKA
      241 WEDHWIFWVG PLIGAAAAML YHQLVLRAGA AKAFASFRNN QHF
//
LOCUS       ZmPIP2-4       289 aa
DEFINITION  ZmPIP2-4     289 aa
TITLE       ZmPIP2-4
ORIGIN

        1 MAKQDIEASG PEAGEFSAKD YTDPPPAPLI DADELTKWSL YRAVIAEFIA TLLFLYITVA
       61 TVIGYKHQTD AAASGPDAAC GGVGILGIAW AFGGMIFILV YCTAGISGGH INPAVTFGLF
      121 LARKVSLVRA LLYIIAQCLG AICGVGLVKG FQSAYYVRYG GGANELSDGY SKGTGLAAEI
      181 IGTFVLVYTV FSATDPKRSA RDSHVPVLAP LPIGFAVFMV HLATIPITGT GINPARSLGA
      241 AVIYNKDKAW DDQWIFWVGP LIGAAIAAAY HQYVLRASAT KLGSYRSNA
//
LOCUS       ZmPIP2-5       288 aa
DEFINITION  ZmPIP2-5     288 aa
TITLE       ZmPIP2-5
ORIGIN

        1 MAKDIEASGP EAGEFSAKDY TDPPPAPLID AEELTQWSLY RAVIAEFIAT LLFLYITVAT
       61 VIGYKHQTDA SASGPDAACG GVGILGIAWA FGGMIFILVY CTAGISGGHI NPAVTFGLFL
      121 ARKVSLVRAL LYIIAQCLGA ICGVGLVKGF QSAYYVRYGG GANELSDGYS KGTGLAAEII
      181 GTFVLVYTVF SATDPKRSAR DSHVPVLAPL PIGFAVFMVH LATIPITGTG INPARSLGAA
      241 VIYNKDKAWD DQWIFWVGPL IGAAIAAAYH QYVLRASATK LGSYRSNA
//
LOCUS       ZmPIP2-6       285 aa
DEFINITION  ZmPIP2-6     285 aa
TITLE       ZmPIP2-6
ORIGIN

        1 MAKDIEAAAA HEGKDYSDPP PAPLVDAEEL TKWSLYRAVI AEFVATLLFL YITVATVIGY
       61 KHQTDAAASG PDAACGGVGV LGIAWAFGGM IFILVYCTAG VSGGHINPAV TFGLFLARKV
      121 SLVRALLYIV AQCLGAICGV GLVKGFQSAF YVRYGGGANE LSAGYSKGTG LAAEIIGTFV
      181 LVYTVFSATD PKRNARDSHV PVLAPLPIGF AVFMVHLATI PITGTGINPA RSLGAAVIYN
      241 NDKAWDDHWI FWVGPFIGAA IAAAYHQYVL RASAAKLGSS ASFSR
//
LOCUS       ZmPIP2-7       286 aa
DEFINITION  ZmPIP2-7     286 aa
TITLE       ZmPIP2-7
ORIGIN

        1 MGKEVDVSTL EAGGVRDYAD PPPAPLIDVD ELGKWSLYRA VIAEFVATLL FLYITVATVI
       61 GYKHQTDASA SGPGAACGGV GVLGIAWAFG GMIFILVYCT AGISGGHINP AVTFGLFLAR
      121 KVSLVRALLY MAAQSLGAIC GVALVKGFQS GLYARYGGGA NEVSAGYSTG TELAAEIVGT
      181 FVLVYTVFSA TDPKRNARDS HVPVLAPLPI GFAVFMVHLA TIPITGTGIN PARSLGAAVV
      241 YNNSKAWSDQ WIFWVGPFIG AAIAALYHQI VLRASARGYG SFRSNA
//
LOCUS       ZmPIP2-8       195 aa
```

```
DEFINITION  ZmPIP2-8     195 aa
TITLE       ZmPIP2-8
ORIGIN

        1 MIFILVYCTA GISGGHINPA VTFGLFLARK VSLVRALLYM AAQSLGAICG VALVKGFQSG
       61 FYARYGGGAN EVSAGYSTGT GLAAEIIGTF VLVYTVFSAT DPKRNARDSH VPVLAPLPIG
      121 FAVFMVHLAT IPITGTGINP ARSLGAAVVY NNSKAWSDQW IFWVGPFIGA AIAALYHQIV
      181 LRASARGYGS FRSNA
//
LOCUS       ZmSIP1-1       262 aa
DEFINITION  ZmSIP1-1     262 aa
TITLE       ZmSIP1-1
ORIGIN

        1 MPIPARPPPL RNPSVESPPA RLLCACPANS PRKPRERRPY LVSGCRDLTD AERWRSEILR
       61 EIGAKVDEIQ NEGLGEHRLR DLNDEINKLL PAVTSYQGAQ EGASHYALLV TTSLLSVLLF
      121 TFDLLCGVLG GASFNPTDFA VSYAAGLDSP SLFSVALRFL VQVVGVVGGA LAISELMQAQ
      181 YKHTLAGPSL KVDPHTGALA EGMLTFIITL TVLWVIIKGP SNVILKALLL STSIVSVILP
      241 GAEYKGPSMN PANVSILPFL CF
//
LOCUS       ZmSIP1-2       487 aa
DEFINITION  ZmSIP1-2     487 aa
TITLE       ZmSIP1-2
ORIGIN

        1 MHRARPANPP RKPRERRPYL VSECRDLTDA ARWRSEILRE ISAKVDEIQN EGLGEHRLRD
       61 LNDEINKLLP AVTSYQGAQE GASHYALLVT TSLLSVLLFT FDLLCGVLGG ASFNPTDFAV
      121 SYAAGLDSPS LFSVALRFSV QAVGVVGGAL AISELMQAQY KHTLAGPSLK VDPHTGALAE
      181 GMLTFVITLT VLWVIIKGPS NVILKALLLS TSIVSVILPG AEYKGPSMNP TNVSFARIPF
      241 SNSYIICSCF FIDIDIGGFT SYYPKFIKSK EYIPSISKYA QRFHGVPAEE RLHWRSFLVK
      301 LGSENLKGSK NEELHVASHK SYKNGFVWHD LSEDDLVLPA TDDEYVLKGS ELVDQSPSVH
      361 GIRQRLEKAN LVDDVVDFHR VTANQAERYL TSYQRSAQMV LFIPCQVTPH LVMTLRSEDQ
      421 ALAIYENHPM LAKEYERVRA GKPPFMLDMY RYGLEPPPMN KRNDVGAWRQ ALRNAQSQLQ
      481 HQIISVM
//
LOCUS       ZmSIP1-3       507 aa
DEFINITION  ZmSIP1-3     507 aa
TITLE       ZmSIP1-3
ORIGIN

        1 MPIPACPPPP RNLSVDCPPP TSSAHAPRPP RLLSTNAPRP PRKPPRKPRE RQPYLVSEFR
       61 DLVDAERWRS EIFREIGAKV AEIQNEGLAA ITSYQGRRRA LATMRFSSPR PSSRCSSSPS
      121 TSFSECRDLT DAERWRSEIL REIGAKVAEI QNEGLDEHRL RDLNDEINKL LHERGHWERR
      181 IVELELLLSK PQSSLLFPIE PQTRQEAQEG ASHYALLVTT SLLSVLLFTF DLLCGVLGGA
      241 SFNPTDFVVS YAAGLDSPSL FSIALRFSAQ AVGVVGDALA ISELMQAQYK HTLAGPSLKV
      301 DPHTGALAEG MLTFVITLTM LWVIIKGPSN IILKALLLST SIVSVILAGA EYKGPSMNPA
      361 NVGFTSYYPK FTGGFTSYYP KFRKSKEYIP SISKYAQRLS PYILACKLSN QIGLGDISRN
      421 LKYECTLLSD QPLQITNQEA IQAVACEGNK YLLGAELDNT VGIVGALELA ETWSHLQVWT
      481 SYSISSRRLI IVGNSNNTGK QKYVSYS
//
LOCUS       ZmSIP1-4       245 aa
DEFINITION  ZmSIP1-4     245 aa
TITLE       ZmSIP1-4
ORIGIN
```

```
        1 MAMGATVRAA AADAVVTFLW VLCASALGAS TAAVTSYLGV QEGAGHYALL VTTSLLSVLL
       61 FTFDLLCGAL GGASFNPTDF AASYAAGLDS PSLFSVALRF PAQAAGAVGG ALAISELMPA
      121 QYKHTLAGPS LKVDPHTGAL AEGVLTFVIT LTVLWVIVKG PRNVILKTLL LSTSIVSVIL
      181 AGAEYTGPSM NPANAFGWAY VNNWHNTWEQ LYVYWICPFI GAMLAGWIFR VVFLPPAPKP
      241 KTKKA
//
LOCUS       ZmSIP1-5       243 aa
DEFINITION  ZmSIP1-5     243 aa
TITLE       ZmSIP1-5
ORIGIN

        1 MAMGEALRAA AADAVVTFLW VLCVSTLGAS TTAVTSYLRL QGVHFALLVT VSLLSVLLFV
       61 FNILCDALGG ASFNPTGVAA FYAAGVTSPS LFSIALRLPA QAAGAVGGAL AISELMPAQY
      121 RHMLGGPSLK VDPHTGAGAE LVLTFVITLA VLLIIVKGPR NPIIKTWMIS ICTLCLVLSG
      181 AAYTGPSMNP ANAFGWAYVN NRHNTWEQFY VYWICPFIGA ILAAWIFRAM FLTPPPKPKA
      241 KKA
//
LOCUS       ZmSIP1-6       857 aa
DEFINITION  ZmSIP1-6     857 aa
TITLE       ZmSIP1-6
ORIGIN

        1 MAMGATVRAA AADAMVTFLW VLCASALDVS IAAVTSYQGL QEGTGHYALL VTTSLLSVLL
       61 FTFDLLYGVL GGASFNFTDF ATSYSAGLDS PSLFSVALHF LARTVVVVKR TTLRVARKDA
      121 YMAELDLFAK GLHERLVPFL GHCLDKEEEK VHVYRFVQNG DLSSALHKKS REEDEGLQSL
      181 DWIKRLKIAT GVAEALCYLH HECSPPMVHR DVQASSILLD DKFDVRLGSL SELPAIESVL
      241 NAMKSNTNQR VSPIFALVLC PTRELAIQLT AEANVLLKYH EGIGVQSLIG GTRFKLDQRR
      301 LESDPCQPDH LLDETNRAGS KIRVERAERS SYTFQSQLDG GAQFCVSQEA SRLTSLFLVF
      361 MPTAQDNTTT PMELEDFIRL DSIWMRWTLL AEDRTPKPGS QPRKPGDRRR QVENTLGGRS
      421 QAASESRGGV AVLAHGGGHS RRRSGMATPT PMPGSEGTLV AVMPRSPSPT PAEAGTSATE
      481 TPVLIFLYFH KAIRAELEAL HGAAVLLATE RTGDVAALAE RCRFFFSIYK HHCDAEDAVI
      541 FPALDIRVKN VAGTYSLEHK GESDLFSQLF DLLELDIQND DALRRELASC TGFSFLPLPA
      601 SVLLFIRLVF PLLTKKFSCE EQADLVWQFL CNIPVNMVAE FLPWLSTSVT SDEHQDIRDC
      661 LCKVVPDEKL LQQVVFTWME GKAAREVAES FATGNLVRNH SAEDVSDHGE IYVCSQQESK
      721 LGSKNCAESN GSQADRHPID DILYWHNAIR MELHDIKKET RRVQQSGNFS DISAFNERLQ
      781 FIADVCIYHS IAEDQVVFPA VDSELSFVQE HAEEEHRFNN FRCLIQQFQI AGAKSTALDF
      841 YNTRVIKTDT ADCSTPN
//
LOCUS       ZmSIP2-1       249 aa
DEFINITION  ZmSIP2-1     249 aa
TITLE       ZmSIP2-1
ORIGIN

        1 MSPAPSRPRI RPWLVVGDLA LAAAWVCAGA LVKLLVYGGL GLGGRPEAEA VKVSLSLVYM
       61 FLFAWLEAAS GGASYNPLTV LAAALASHGG PAVYLFTAFA RIPAQVIGAV LGVKLIQVTF
      121 PNVGKGARLS VGAHHGALAE GLATFMVVMV SVTLKKKEMK SFFMKTWITS IWKNTIHLLS
      181 SDITGGIMNP ASAFAWAYAR GDHTTFDHLL VYWLAPLQAT LLGVWAVTFF TKPKKIKEQK
      241 VDENKIKKE
//
LOCUS       ZmTIP1-1       254 aa
DEFINITION  ZmTIP1-1     254 aa
TITLE       ZmTIP1-1
ORIGIN
```

```
        1 MPVSRIAVGA PGELSHPDTA KAAVAEFISM LIFVFAGSGS GMAFSKLTDG GATTPAGLIA
       61 ASLAHALALF VAVSVGANIS GGHVNPAVTF GAFVGGNISL LKALVYWVAQ LLGSVVACLL
      121 LKIATGGAAI GAFSLSAGVG AMNAVVLEMV MTFGLVYTVY ATAVDPKKGD LGVIAPIAIG
      181 FIVGANILAG GAFDGASMNP AVSFGPAVVT GVWENHWVYW VGPLAGAAIA ALVYDIIFIG
      241 QRPHQQLPTT AADY
//
LOCUS       ZmTIP2-1       249 aa
DEFINITION  ZmTIP2-1     249 aa
TITLE       ZmTIP2-1
ORIGIN

        1 MVKLAFGSVG DSFSATSIKA YVAEFIATLL FVFAGVGSAI AYGQLTNGGA LDPAGLVAIA
       61 IAHALALFVG VSVAANISGG HLNPAVTFGL AVGGHITILT GVFYWVAQLL GATVACLLLG
      121 FVTHGKAIPT HAVAGISELE GVVFEVVITF ALVYTVYATA ADPKKGSLGT IAPIAIGFIV
      181 GANILAAGPF SGGSMNPARS FGPAVAAGDF AGNWVYWVGP LVGGGLAGLV YGDVFIGGSY
      241 QQVADQDYA
//
LOCUS       ZmTIP2-2       250 aa
DEFINITION  ZmTIP2-2     250 aa
TITLE       ZmTIP2-2
ORIGIN

        1 MVKLAFGSVG DSFSVTSIKA YVAEFIATLL FVFAGVGSAI AFGQLTNGGA LDPAGLVAIA
       61 VAHALALFVG VSVAANTSGG HLNPAVTFGL AVGGHITVLT GLFYWVAQLL GASVACLLLR
      121 FVTHGKAIPT HGVSGGTTEL EGVVFEIVIT FALVYTVYAT AADPKKGSLG TIAPIAIGFI
      181 VGANILAAGP FSGGSMNPAR SFGPAVAAAD FAGNWVYWVG PLIGGGLAGL VYGDVFIGGS
      241 YQQVADQDYA
//
LOCUS       ZmTIP2-3       248 aa
DEFINITION  ZmTIP2-3     248 aa
TITLE       ZmTIP2-3
ORIGIN

        1 MVKPAFGSFG DSFSAASLKA YAAEFIATLL FVFAGVGSAI AYSQLTKGGA LDPAGLVAIA
       61 IAHAFALFVG VSMAANISGG HLNPAVTFGL AVGGHITILT GVFYWVAQLL GASVACLLLQ
      121 FVTHGQAIPT HGVSGISEIE GVVMEIVITF ALVYTVYATA ADPKKGSLGT IAPIAIGFIV
      181 GANILAAGPF SGGSMNPARS FGPAVAAGNF AGNWVYWVGP LIGGGLAGLV YGDVFIASYQ
      241 PVGQQEYP
//
LOCUS       ZmTIP2-4       248 aa
DEFINITION  ZmTIP2-4     248 aa
TITLE       ZmTIP2-4
ORIGIN

        1 MVKLAFGSFR DSLSAASLKA YVAEFIATLL FVFAGVGSAI AYSQLTKGGA LDPAGLVAIA
       61 IAHAFALFVG VSMAANISGG HLNPAVTFGL AVGGHITILT GILYWVAQLL GASVACFLLQ
      121 YVTHGQAIPT HGVSGISEIE GVVMEIVITF ALVYTVYATA ADPKKGSLGT IAPMAIGFIV
      181 GANILAAGPF SGGSMNPARS FGPAVAAGNF AGNWVYWVGP LVGGGLAGLV YGDVFIASYQ
      241 PVGQQEYP
//
LOCUS       ZmTIP3-1       266 aa
DEFINITION  ZmTIP3-1     266 aa
TITLE       ZmTIP3-1
```

```
ORIGIN

        1 MSTATGVRAG RRFTVGRSED ATHPDTIRAA ISEFIATAIF VFAAEGSVLS LGKMYHDHST
       61 ISTAGGLVAV ALAHALGLAV AVAVAVNVSG GHVNPAVTFG ALVGGRVSLV RAVLYWAAQL
      121 LGAVAATLLL RLATGGARPP GFALASGVGD GHAVLLEAVM TFGLVYAYYA TVVDPKRGHL
      181 GTIAPLAVGF LLGANVLAGG PFDGAGMNPA RVFGPALVGW RWRHHWVYWL GPFLGAGLAG
      241 LVYEYLLIPP ADAVPHTHQP LAPEDY
//
LOCUS       ZmTIP3-2       262 aa
DEFINITION  ZmTIP3-2     262 aa
TITLE       ZmTIP3-2
ORIGIN

        1 MSTGVRPGRR FTVGRSEDAT HPDTIRAAIS EFIATAIFVF AAEGSVLSLG KMYHDMSTAG
       61 GLVAVALAHA LALAVAVAVA VNISGGHVNP AVTFGALVGG RVSLVRAVLY WVAQLLGAVA
      121 ATLLLRLATG GMRPPGFALA SGVGDWHAVL LEAVMTFGLM YAYYATVIDP KRGHVGTIAP
      181 LAVGFLLGAN VLAGGPFDGA GMNPARVFGP ALVGWRWRHH WVYWLGPFLG AGLAGLVYEY
      241 LVIPSADAAV PHAHQPLAPE DY
//
LOCUS       ZmTIP3-3       267 aa
DEFINITION  ZmTIP3-3     267 aa
TITLE       ZmTIP3-3
ORIGIN

        1 MNMIRAVRRR FTVGHLATAK DPATLRHAAA ELLATAIFVF AAEGATLSLG RMHHHDKGGG
       61 GLVAVALAHA LALAAAVGCA ANISGGHVNP AVTFGALLAG RICLVRSLVY WAAQLLGAVA
      121 AALVLRLATG GMHLPEYALA GGVSGWNAAV LEAAMAFGLM YAYFATVMDK ARRVRAGAGA
      181 LAAPLAVGLL AGANVLACGA LEGAVMNPAR AFGPAVVGSR RWRHQWVYWV GPMVGAGLSG
      241 VVYEHLVAGP AAEEEEPAPS CGDRRRA
//
LOCUS       ZmTIP3-4       265 aa
DEFINITION  ZmTIP3-4     265 aa
TITLE       ZmTIP3-4
ORIGIN

        1 MLPGRHPARR ADTTGTGPLL PDATRAVVSE FVATAMFVFA AEGSVYGLWK LYKDTATPGG
       61 LLAVAIAHTL ALVAAVAVAS NASGGHVNPA VTFGLLVGRR ISFGRAAVYW LAQMLGAVVA
      121 SLLLTLVSGG TRPVGFGLVR GVHERHALLL EAVMTFGLMY AVYATAVDHR SRGGAVAIAP
      181 LAIGFVLGAN ILAGGPFDGA AMNPARAFGP ALVGWSWRHH WVYWVGPLIG AGLAGGLYEF
      241 VMVEQEPEAP APAAVPRMPV ASEDY
//
LOCUS       ZmTIP4-1       311 aa
DEFINITION  ZmTIP4-1     311 aa
TITLE       ZmTIP4-1
ORIGIN

        1 MSHSPLPPPV FQNISLRFSE SFSLQGTETT GAFTPPAFPS PPGTTGLLAI IRPSMAKLVN
       61 KLVDSFDHHE APAPDVGCVR AVLAELVLTF LFVFTGVSAS MAAGAGGKPG EAMPMATLAA
      121 VAIAHALAAG VLVTAGFHVS GGHLNPAVTV GILVRGHITK LRALLYVAAQ LLASSLACIL
      181 LRYLSGGMVT PVHALGAGIS PMQGLVMEVI LTFSLLFVTY AMILDPRSQV RTIGPLLTGL
      241 IVGANSLAGG NFTGASMNPA RSFGPAMATG VWTNHWVYWI GPLLGGSLAG FVYESLFMVY
      301 KTHEPLLNGD I
//
LOCUS       ZmTIP4-2       255 aa
```

```
DEFINITION  ZmTIP4-2     255 aa
TITLE       ZmTIP4-2
ORIGIN

        1 MAKLMNKLVD SFEHDEILDV GCVRAVLAEL VLTFLFVFTG VSAAMAAGSD GKPGDAMPMA
       61 TLAAVAIAHA LAAGVLVTAG FHVSGGHLNP AVTVGLMVRG HITKLRAVLY VAAQLLASSA
      121 ACVLLRFLSG GMVTPVHALG RGISPMQGLV MEVILTFSLL FVTYAMILDP RSQVRAIGPL
      181 LTGLIVGANS LAGGNFTGAS MNPARSFGPA LATGDWTNHW VYWIGPLLGG PLAGFVYESL
      241 FLVQKMHEPL LNGEV
//
LOCUS       ZmTIP4-3       249 aa
DEFINITION  ZmTIP4-3     249 aa
TITLE       ZmTIP4-3
ORIGIN

        1 MGKLTLGHRG EASEPDFFRG VLGELVLTFL FVFIGVGAAM TDGATTKGST AGGDLTAVAL
       61 GQALVVAVIA TAGFHISGGH VNPAVTLSLA VGGHVTLFRS SLYIAAQMLG SSAACFLLRW
      121 LTGGLATPVH ALAEGVGALQ GVVAEAVFTF SLLFVIYATI LDPRKLLPGA GPLLTGLLVG
      181 ANSVAGAALS GASMNPARSF GPAVASGIWT HHWVYWVGPL AGGPLAVLVY ECCFIAAAPT
      241 HALLPQQDP
//
LOCUS       ZmTIP4-4       287 aa
DEFINITION  ZmTIP4-4     287 aa
TITLE       ZmTIP4-4
ORIGIN

        1 MYPSPPPHLF YGVSVSRQHS PRSNRHFFNP LAKRAMAKFA LGHHREASDA GCVRAVLAEL
       61 ILTFLFVFAG VGSAMATGKL AGGGGDTVVG LTAVALAHTL VVAVMVSAGL HVSGGHINPA
      121 VTLGLAATGR ITLFRSALYV AAQLLGSTLA CLLLAFLAVA DSGVPVHALG AGVGALRGVL
      181 MEAVLTFSLL FAVYATVVDP RRAVGGMGPL LVGLVVGANV LAGGPFSGAS MNPARSFGPA
      241 LVAGVWADHW VYWVGPLIGG PLAGLVYDGL FMAQGGHEPL PRDDTDF
//
LOCUS       JcPIP1-1[Jatropha curcas]       287 aa
DEFINITION  JcPIP1-1[Jatropha curcas]     287 aa
TITLE       JcPIP1-1[Jatropha curcas]
ORIGIN

        1 MEGKEEDVRL GANKFRETQP IGTAAQSQDD KDYTEPPPAP LFEPTELTSW SFYRAGIAEF
       61 IATFLFLYIS VLTVMGVVKA PTKCSTVGIQ GIAWAFGGMI FALVYCTAGI SGGHINPAVT
      121 FGLFLARKLS LTRAIYYMVM QCLGAICGAG VVKGFEGRKQ YTLLGGGANS VAPGYTKGDG
      181 LGAEIVGTFV LVYTVFSATD AKRNARDSHV PILAPLPIGF AVFLVHLATI PITGTGINPA
      241 RSLGAAIIFN KDQGWDDHWI FWVGPFIGAA LAALYHQVVI RAIPFKK
//
LOCUS       JcPIP1-2[Jatropha curcas]       287 aa
DEFINITION  JcPIP1-2[Jatropha curcas]     287 aa
TITLE       JcPIP1-2[Jatropha curcas]
ORIGIN

        1 MEGKEEDVRL GANKFTERQP IGTAAQTDKD YKEPPPAPLF EPGELSSWSF YRAGIAEFVA
       61 TFLFLYITVL TVMGVSKSGN KCATVGTQGI AWAFGGMIFA LVYCTAGISG GHINPAVTFG
      121 LFLARKLSLT RALFYIIMQC LGAICGAGVV KGFEGNRVYE SLGGGANVVA SGYTKGDGLG
      181 AEIVGTFVLV YTVFSATDAK RSARDSHVPI LAPLPIGFAV FLVHLATIPI TGTGINPARS
      241 LGAAIIFNKD HAWDDHWVFW VGPFIGAALA ALYHQIVIRA IPFKSRA
//
```

```
LOCUS       JcPIP1-3 [Jatropha curcas]       212 aa
DEFINITION  JcPIP1-3 [Jatropha curcas]     212 aa
TITLE       JcPIP1-3 [Jatropha curcas]
ORIGIN

        1 MEGREEDVKM GANRAGIAEF VATFLFLYIT FLTVIGVAKS PTKCSTVGIQ GIAWAFGGMI
       61 FALVYCTAGI SGGHINPAGF EKNQYERLGG GANTISAGYS KGDGLGAEIV GTFLLVYTVF
      121 SATDAKRNAR DSHVPILAPL PIGFAVFLVH LATIPITGTG INPARSLGAA LIYNKEQAWD
      181 DHWIFWIGPF IGAALAALYH QIVIRAIPFK SK
//
LOCUS       JcPIP1-4 [Jatropha curcas]       286 aa
DEFINITION  JcPIP1-4 [Jatropha curcas]     286 aa
TITLE       JcPIP1-4 [Jatropha curcas]
ORIGIN

        1 MEGREEDVRV GATKFPEKQA IGTSAQTDKD YKEPPSTPLF EPGELQSWSF WRAGIAEFMA
       61 SFLFLYVAIS TVMGYSRSSN KCATVGVQGI AWAFGGMIFA LVYSTAGISG GHINPAVTFG
      121 LLLARKVSLT RAIFYMVMQC LGAICGAGVV KGFQPTPYQM GGGGANMVQP GYSKGDGLGA
      181 EIVGTFVLVY TVFSATDAKR SARDSHVPIL APLPIGFAVF VVNMATIPIT GAGINPARSL
      241 GAAVIYNNDN GWDDHWIFWV GPFIGAALAA LYHQIVIRAI PFKSRA
//
LOCUS       JcPIP2-1 JCGZ_16499 [Jatropha curcas]       284 aa
DEFINITION  JcPIP2-1 JCGZ_16499 [Jatropha curcas]     284 aa
TITLE       JcPIP2-1 JCGZ_16499 [Jatropha curcas]
ORIGIN

        1 MGKDVEGGDF QAKDYHDPPP APLIDAEEFT QWSFYRAIIA EFIATLLFLY ITVLTVIGYK
       61 SQTDPSKNSD ACGGVGILGI AWAFGGMIFI LVYCTAGISG GHINPAVTFG LFLARKVSLV
      121 RAILYMAAQC LGAICGCGLV KAFQKAYYTR YGGGANELAD GYSKGTGLGA EIIGTFVLVY
      181 TVFSATDPKR NARDSHVPVL APLPIGFAVF MVHLATIPIT GTGINPARSF GAAVIYNKDK
      241 AWDDQWIFWV GPFIGAAIAA IYHQYILRAG AVKALGSFRS TSNV
//
LOCUS       JcPIP2-2 JCGZ_05520 [Jatropha curcas]       286 aa
DEFINITION  JcPIP2-2 JCGZ_05520 [Jatropha curcas]     286 aa
TITLE       JcPIP2-2 JCGZ_05520 [Jatropha curcas]
ORIGIN

        1 MAKDVEVAEP TGEFSGKDYH DPPPAPLIDM DELGQWSFYR ALIAEFIATL LFLYITVLTV
       61 IGYKSQTDPN KNADACGGVG ILGIAWAFGG MIFILVYCTA GISGGHINPA VTFGLFLGRK
      121 VSLIRALGYM IAQCLGAICG CGLVKAFQKA YYNRYGGGAN ELADGYNKGT GLGAEIIGTF
      181 VLVYTVFSAT DPKRNARDSH VPVLAPLPIG FAVFMVHLAT IPITGTGINP ARSFGAAVIY
      241 NKDKAWDDQW IFWVGPFVGA AIAAFYHQYI LRAAAIKALG SFRSNA
//
LOCUS       JcPIP2-3 JCGZ_20043 [Jatropha curcas]       285 aa
DEFINITION  JcPIP2-3 JCGZ_20043 [Jatropha curcas]     285 aa
TITLE       JcPIP2-3 JCGZ_20043 [Jatropha curcas]
ORIGIN

        1 MVKDVAEQGS FSAKDYHDPP PAPLIDAEEL TKWSFYRALI AEFIATLLFL YITVLTVIGY
       61 KSQTDPAKNA DSCGGVGILG IAWAFGGMIF ILVYCTAGIS GGHINPAVTF GLFLARKVSL
      121 VRAVMYMVAQ SLGAICGVGL VKAFQSAYYK RYGGGANTLA DGYSTGVGLG AEIIGTFVLV
      181 YTVFSATDPK RSARDSHVPV LAPLPIGFAV FMVHLATIPI TGTGINPARS LGAAVIYNHD
      241 KAWDDQWIFW VGPFLGAAIA AFYHQFILRA GAVKALGSFR SNPTV
//
```

```
LOCUS       JcPIP2-4 JCGZ_18836 [Jatropha curcas]       280 aa
DEFINITION  JcPIP2-4 JCGZ_18836 [Jatropha curcas]     280 aa
TITLE       JcPIP2-4 JCGZ_18836 [Jatropha curcas]
ORIGIN

        1 MAKEVSEETQ TTHAKDYVDP PPAPLIDMAE IKLWSFYRAL IAEFIATLLF LYITVATVIG
       61 YKKQTDPCGG VGLLGIAWAF GGMIFILVYC TAGISGGHIN PAVTFGLFLA RKVSLIRALA
      121 YMVAQCLGAI CGVGLVKAFM KNPYNHLGGG ANSVNTGYSK GTALGAEIIG TFVLVYTVFS
      181 ATDPKRSARD SHVPILAPLP IGFAVFMVHL ATIPITGTGI NPARSFGAAV IYNNDKVWDD
      241 HWIFWVGPFV GAIAAAAYHQ YILRAAAIKA LGSFRSNPTN
//
LOCUS       JcPIP2-5 JCGZ_25357 [Jatropha curcas]       283 aa
DEFINITION  JcPIP2-5 JCGZ_25357 [Jatropha curcas]     283 aa
TITLE       JcPIP2-5 JCGZ_25357 [Jatropha curcas]
ORIGIN

        1 MANKELGEEV AHQHGKDYVD PPPAPLLDIE ELTKWSFYRA VIAELVATLI FLFVTVATVI
       61 ANKASTMSSG CGGVGLLGVA WSFGSTIFIL VYCTAGISGG HINPAVTFGL LLARKVSLNR
      121 AVAYIVAQCV GAIIGVALVK GLVKDLYKSL GGGANSVTAG FSIGTGLGVE ILGTFFLEYT
      181 VLSATDPKRK ARDSHVPVLA PLPIGFTVFV VHMATLPITG TGINPARSFG AAVIYNKKMV
      241 WDDHWIFWVG PLVGAAAAAA YHQIVLRAGA VKALGSFRGN PVM
//
LOCUS       JcTIP1-1 JCGZ_16577 [Jatropha curcas]       252 aa
DEFINITION  JcTIP1-1 JCGZ_16577 [Jatropha curcas]     252 aa
TITLE       JcTIP1-1 JCGZ_16577 [Jatropha curcas]
ORIGIN

        1 MPIRNIAIGH PQEATHPDAL KAALAEFIST LIFVFAGEGS GMAFNKLTNN GATTPAGLVA
       61 ASLAHGFGLF VAVSVGANIS GGHVNPAVTF GAFVGGNITL LRGILYWIAQ LLGSTVACLL
      121 LKFSTGGLTT SAFALSSGVG VWNAFVFEIV MTFGLVYTVY ATAIDPKKGS LGTIAPIAIG
      181 FIVGANILAG GAFDGASMNP AVSFGPALVS WSWDNHWVYW AGPLVGGGLA GLVYEFFFIG
      241 HNTHEQLPTA DY
//
LOCUS       JcTIP1-2 JCGZ_18448 [Jatropha curcas]       252 aa
DEFINITION  JcTIP1-2 JCGZ_18448 [Jatropha curcas]     252 aa
TITLE       JcTIP1-2 JCGZ_18448 [Jatropha curcas]
ORIGIN

        1 MPITRIAIGN PGEASQPDAL RAALAEFFSM VIFVFAGEGS GMAFNKLTSD GSSTPAGLVA
       61 ASLAHAFALF VAVSVGANIS GGHVNPAVTF GAFIGGNITL LRGILYWIAQ LLGSVVACLL
      121 LKYATGGLET SAFALSSGVS AWNAVVFEIV MTFGLVYTVY ATAVDPKKGN LGIIAPIAIG
      181 FIVGANILAG GAFDGASMNP AVSFGPAVVS WTWTNHWVYW VGPLIGAAIA ALVYDNIFIG
      241 EGAHEPLSTS DF
//
LOCUS       JcTIP1-3 JCGZ_05655 [Jatropha curcas]       252 aa
DEFINITION  JcTIP1-3 JCGZ_05655 [Jatropha curcas]     252 aa
TITLE       JcTIP1-3 JCGZ_05655 [Jatropha curcas]
ORIGIN

        1 MPITSIAIGS PAEVGQADAL KAALAEFIST LIFVFAGEGS GMAFNKLTSD GSTTPAGLVA
       61 ASLAHGFALF VAVSVGANIS GGHVNPAVTF GAFVGGHITL MRSILYWVGQ LLGSVVACLL
      121 LKFATGGLET SAFALSSGVS SWNALVFEIV MTFGLVYTVY ATAVDPRKGN LGTIAPIAIG
      181 FIVGANILAG GAFDGASMNP AVSFGPAVVS WTWSNHWVYW LGPFIGGGIA AVVYETFFIS
      241 PNTHEQLPSA DF
```

```
//
LOCUS       JcTIP1-4 JCGZ_05430 [Jatropha curcas]       254 aa
DEFINITION  JcTIP1-4 JCGZ_05430 [Jatropha curcas]     254 aa
TITLE       JcTIP1-4 JCGZ_05430 [Jatropha curcas]
ORIGIN

        1 MPPVNFRIAV GLPHEDITHP GAIKAALAEF ISTAIFVFAG EGSGMAFNKL TDDGSSTPAG
       61 IIMASLAHAF GLFIGVATAA NISGGHVNPA VTFGAFVGGN ITLLRGILYW IAQLLGSTVA
      121 CLLLKFSTHG MTTSAFALSS GVNVWNALVF EIVMTFGLVY TVYATAIDPR KGQLGTIAPI
      181 AIGFIVGANV LAGGAFEGAS MNPAVSFGPA LVSWDWTHHW VYWAGPLIGG GLAGLIYETI
      241 FISRTHEPVP SPEF
//
LOCUS       JcTIP2-1 JCGZ_06324 [Jatropha curcas]       248 aa
DEFINITION  JcTIP2-1 JCGZ_06324 [Jatropha curcas]     248 aa
TITLE       JcTIP2-1 JCGZ_06324 [Jatropha curcas]
ORIGIN

        1 MAKIAFGRFD DSFSLGSFKA YLAEFISTLL FVFAGVGSAI AYNKLTGNAA LDPAGLVAIA
       61 ICHGFALFVA VAVGANISGG HVNPAVTFGL ALGGQITILT GIFYWIAQLL GSIVACLLLK
      121 VVTGGLETPT HSLAAGVGAI EGVVMEIIVT FALVYTVYAT AADPKKGSLG TIAPIAIGFI
      181 VGANILAAGP FSGGSMNPAR SFGPAVASGD FHDNWIYWVG PLIGGGLAGL IYGNLYINND
      241 HAPLSNDF
//
LOCUS       JcTIP2-2 JCGZ_03415 [Jatropha curcas]       250 aa
DEFINITION  JcTIP2-2 JCGZ_03415 [Jatropha curcas]     250 aa
TITLE       JcTIP2-2 JCGZ_03415 [Jatropha curcas]
ORIGIN

        1 MVKIAFGNFG DSFSVGSLKA YLSEFIATLL FVFAGVGSAI AYSKLTADAA LDPPGLVAVA
       61 VAHAFALFVG VSIAANISGG HLNPAVTFGL AIGGHITLLT GLFYWIAQSL GSIVACLLLK
      121 FVTNGKSIPT HGVASGVNAF EGVVFEIVIT FGLVYTVYAT AADPKKGSLG IIAPIAIGFI
      181 VGANILAAGP FSGGSMNPAR SLGPAVVSGD FSQIWIYWLG PLIGGGLAGL VYGDIFIGSY
      241 SPAPSSEEYA
//
LOCUS       JcTIP3-1 JCGZ_08448 [Jatropha curcas]       257 aa
DEFINITION  JcTIP3-1 JCGZ_08448 [Jatropha curcas]     257 aa
TITLE       JcTIP3-1 JCGZ_08448 [Jatropha curcas]
ORIGIN

        1 MPPRRYIFGR TEEATHPDSI RATLAEFVST LIFVFAGEGS VLALEKLYRE TGPPASGLVM
       61 IALAHALALF AAVAASINIS GGHVNPAVTL GALVGGRISV VRAFYYWIAQ LLGSIVASLL
      121 LRLVTNGMRP EGFHVTAGVG EVHGLIMEIV MTFGLVYTVY ATAIDPKRGS LGIIAPLAIG
      181 LIVGANILVG GPFDGAAMNP ARAFGPALVG WRWSNHWIYW VGPFIGGALA GLIYEYMVIP
      241 TEPPLHHTHQ PLAPEDY
//
LOCUS       JcTIP4-1 JCGZ_07757 [Jatropha curcas]       247 aa
DEFINITION  JcTIP4-1 JCGZ_07757 [Jatropha curcas]     247 aa
TITLE       JcTIP4-1 JCGZ_07757 [Jatropha curcas]
ORIGIN

        1 MAKIALGSRR EATQPDCIKA LIVEFITTFL FVFAGVGSAM AADKLVGGPL VGLFFVAVAH
       61 ALVVAVMISA GHISGGHLNP AVTLGLLFGG HITLVRSILY WIDQLLASSA ACLLLNYLTG
      121 GLATPVHTLA SGVGYLQGVV WEIILTFSLL FTVYATIVDP KKGAIDGLGP TLTGFVVGAN
      181 ILAGGAFSGA SMNPARSFGP ALVSWDWTDH WVYWVGPLIG GGLAGFIYEN FFIIRSHIPI
```

```
      241 PIVEENY
//
LOCUS       JcTIP5-1 JCGZ_26261 [Jatropha curcas]       252 aa
DEFINITION  JcTIP5-1 JCGZ_26261 [Jatropha curcas]     252 aa
TITLE       JcTIP5-1 JCGZ_26261 [Jatropha curcas]
ORIGIN

        1 MAPTSLIVRF QQSVSPDALR SYLAEFISTF FYVFAVVGSS MAARKLMPAA DPSSLIIVAT
       61 ANSFALSSAI YIAANISGGH VNPAVTFSMA VGGHISVPTA LFYWISQMLA SVMACVFLKV
      121 AIVGQNLPTY TIAEEMTGFG ASILEGVLTF GLVYTIYAAG DPRRSLPGAI GPLAIGLVAG
      181 ANVLAAGPFS GGSMNPASAF GSAVVAGRFK NQAVYWVGPL IGATVAGLLY DNVVFPNQVP
      241 DSIRGISDGV RV
//
LOCUS       JcNIP1-1 JCGZ_21622 [Jatropha curcas]       275 aa
DEFINITION  JcNIP1-1 JCGZ_21622 [Jatropha curcas]     275 aa
TITLE       JcNIP1-1 JCGZ_21622 [Jatropha curcas]
ORIGIN

        1 MGEIAGSNGK QVVLDVKDDN NPPPCPSKHK EDSALSISVP FMQKMIAEVA GTYFLIFAGC
       61 TAVAVNLNFD KVVTHPGISI VWGLAVMVLV YSVGHISGAH FNPAVTLAFA TCKRFPWKQV
      121 PAYIVCQVIG STLAAGTIRL IFTGKQDHFV GTMPAGSNMQ SFVVEFIITF YLMFVISGVA
      181 TDNRAIGELA GLAVGATVLL NVIFAGPISG ASMNPARSLG PAIVSCKFKG LWIYLVSPTL
      241 GAQAGAWVYN MIRYTDKPLR EITKSASFIK STGRA
//
LOCUS       JcNIP2-1 JCGZ_19849 [Jatropha curcas]       291 aa
DEFINITION  JcNIP2-1 JCGZ_19849 [Jatropha curcas]     291 aa
TITLE       JcNIP2-1 JCGZ_19849 [Jatropha curcas]
ORIGIN

        1 MATTSASDHT ASPSTEYLVS VENPKSENPF LIPLLSFQNH YPPRFFRKVV AEMIATFLLV
       61 FVTCGTAAIS RSDEKRVSEL GASVAGGLIV TVMIYAVGHV SGAHMNPAVT TAFAAVREFP
      121 WKQVPFYAVA QMTGAIGASF TLKVLLHPIK QLGTTSPSGS DFQALVMEIV VTFSMMFVTS
      181 AVATDTKAIG ELAGIAVGSA VCITSILAGP ISGGSMNPAR TIGPAIASAY YKGIWVYIVG
      241 PVAGTLLGAW SYNLIRVTDK PVQAISPSFS FRIRRTSIDE QTNNKDPLSA V
//
LOCUS       JcNIP3-1 JCGZ_15791 [Jatropha curcas]       277 aa
DEFINITION  JcNIP3-1 JCGZ_15791 [Jatropha curcas]     277 aa
TITLE       JcNIP3-1 JCGZ_15791 [Jatropha curcas]
ORIGIN

        1 MASHSITSTD ISPKLQLPVK RPVLQVPEDN PNSTPSSFLL FQKILAEFLG TYVLIFIGCG
       61 SALTNDIQKL TILGIAIVWG VVLMALIYAV GHISGAHFNP AVSIALAAVR KFSWKHVPVF
      121 VLAQVLGSTL AILTLKVLFH DQDDIQATMT QYKNSTSHLE AIIWEFIITF ILMFNICAVA
      181 TDHRASKDFS GVAIGGTLLV NVMVAGPITG ASMNPARSLG PAIVSGVYKN LWVFLLSPVL
      241 GALAATMVYN MLRVPKPDNP EEKTKNIFNH LYTHADP
//
LOCUS       JcNIP3-2 JCGZ_24027 [Jatropha curcas]       280 aa
DEFINITION  JcNIP3-2 JCGZ_24027 [Jatropha curcas]     280 aa
TITLE       JcNIP3-2 JCGZ_24027 [Jatropha curcas]
ORIGIN

        1 MAAVSPSPLD EFSPKQPRVV ITTPDLITIE EGKPTPSQAS TTDIPVVSPN NAHKIIAEMV
       61 GTYVIVLIGC GSLIVDRING PLSVVGIAVA WGLVVMVMIY TFGHISGGHF NPAITIAFAI
      121 SCKYPWRQVP GYVASQLAGS TLAILTLNVM FHREKIDIKI TTTQYEGRAT DLESFIWEFI
```

```
      181 TSFILMLTIC GVAIDTKAIN ELSGVAVGSA MLFDMLIAGN ITGASMNPAR SIGPALVSKD
      241 FCGLWVYIFA PILGMIAAST MYTFLWPPTQ NVDKDNSKSV
//
LOCUS       JcNIP4-1 JCGZ_02488 [Jatropha curcas]       263 aa
DEFINITION  JcNIP4-1 JCGZ_02488 [Jatropha curcas]     263 aa
TITLE       JcNIP4-1 JCGZ_02488 [Jatropha curcas]
ORIGIN

        1 MPDIIEEETQ ISNTEKGLVV KSSKPNNPND NTASRSSTCF VTIIQKLVAE VIGTYFVIFA
       61 GCGAVAVNKI YGSVTFPGIC VSWGLIVMVM IYTVGHVSGA HFNPAVTIAS AIFRRFPFRE
      121 VPLYIVAQVI GSILASGTLA LLFDITPMAY FGTLPVGSNV QSLVIEIIIT FLLMFVVSGV
      181 NTDDRAVGDL GGIAVGMTIL LNVFVAGPVS GASMNPARSL GPAIVKHTYK GLWVYIAGPI
      241 VGAIAGAFAY NLLRSTDKPL DDE
//
LOCUS       JcNIP5-1 JCGZ_20348 [Jatropha curcas]       298 aa
DEFINITION  JcNIP5-1 JCGZ_20348 [Jatropha curcas]     298 aa
TITLE       JcNIP5-1 JCGZ_20348 [Jatropha curcas]
ORIGIN

        1 MPESEAGTPT VSAPATPGTP GGPLFSSLRV DSLSYDRKSM PRCKCLPVNA PSWGQTHTCF
       61 TDFPAPDVSL TRKLGAEFVG TFILIFAATA GPIVNQKYSG VETLIGNAAC SGLAVMIVIL
      121 STGHISGAHL NPALTIAFAA LRHFPWVQVP AYIAAQVSAS ICASFALKGV FHPFMSGGVT
      181 IPSVSTGQAF ALEFLITFNL LFVVTAVATD TRAVGELAGI AVGATVALNI LVAGPSSGAS
      241 MNPVRTLGPA VAAGNYKAIW IYLVAPILGG LAGGTTYTAV KLREDEADPP RQVRSFRR
//
LOCUS       JcNIP6-1 JCGZ_27003 [Jatropha curcas]       307 aa
DEFINITION  JcNIP6-1 JCGZ_27003 [Jatropha curcas]     307 aa
TITLE       JcNIP6-1 JCGZ_27003 [Jatropha curcas]
ORIGIN

        1 MDNNEEIPSA PSTPATPGTP GAPLFGGFKG TERAGGLTRK SILNGCRCFS IEEWALEEGR
       61 LPPVSCSIPP PPVSLARKVG AEFIGTLILI FAGTATAIVN QKTQGAETLV GLAASSGLAV
      121 MIVILSTGHI SGAHLNPSVT IAFAALNHFP WKHVPVYIGA QVLASLGAAF ALKGILHPIM
      181 GGGVTVPSGG YGQAFALEFI ISFNLMFVVT AVATDTRAVG ELAGIAVGAT VMLNILIAGP
      241 ATGASMNPVR TLGPAIAANN YKAIWIYLTA PILGALCGAG TYSAVKLPEE DGNARQKPLA
      301 ATASFRR
//
LOCUS       JcNIP7-1 JCGZ_02114 [Jatropha curcas]       618 aa
DEFINITION  JcNIP7-1 JCGZ_02114 [Jatropha curcas]     618 aa
TITLE       JcNIP7-1 JCGZ_02114 [Jatropha curcas]
ORIGIN

        1 MDCDDGSYLS STLKHSFCGS APCSVAKAQC FGQCQPGRRR PGCDKEHCYI LADNTIPALG
       61 SGFDIGLISL DKIALQSTDG SKFGPTVTVS DFIFGCLGAR ERLSNLAKGA DGMIGLGRQP
      121 ITLPTQLSSG GSFRKKFAIC LPSTPKLNGV MFFGDSPYAF YPSYNTSKTI DVSTRFHYTK
      181 LYVRTEFSGS SIVTRGPPSP EYFVNVTSIL VNRKPIFINP TFLEFHRNGK GGAKIATVEP
      241 YTKLETTIYK SLVKAFDKEI AVLSGSKVSP VAPFTDCYKI DHIGMTPLGI GVPDLAFEFE
      301 NNKNEQWEMY GFNSMVEVSR DVACLAFLDA GDDPIVTTPI VIGAHQLQDN LLQFDLASNR
      361 LAFTRTLLLA AAECSNFKNA QVIAEMMGTF VLMICVCGII GTTQLTRDQL GLLEYATTAG
      421 LTVIVLVFSI GPISGAHVNP AITIAFATFG HFPWSRVPFY VSAQIVGSAL ASYAGGSIYG
      481 IKPDLMTTRP FHGCSSAFWV EFIATFIIMF VAASLAYQTS VRQLSGFVLG VAIALAVLIT
      541 GPLSGGSLNP ARSLGPAIVS RNFKDIWVYI TAPILGSITG ALMFHVLRIQ RRPCSSTSSP
      601 DTGLLAHSMD FGGTVDSS
//
```

```
LOCUS       JcXIP1-1 JCGZ_19604 [Jatropha curcas]       271 aa
DEFINITION  JcXIP1-1 JCGZ_19604 [Jatropha curcas]     271 aa
TITLE       JcXIP1-1 JCGZ_19604 [Jatropha curcas]
ORIGIN

        1 MNSFKETIRT TKTGFLFRIG AHELFSQEMW RAALTELVAT ACLLFTLTIS IISCLDSHEA
       61 DPKLLIPFTI FIIAFFLLLT TIPLSGGHMS PIFTFIAALK GLTTLVRALF YILAQCIGSV
      121 MAYMLIKSVM DHRIAEKYYL GGCIIDGNGK GIAQTTALVL EFCCTFVVLF VGVTVGFDTR
      181 RFKELGLVMV CVILAASMGL AVFVSISVTG RAGYGGVGLN PARCLGPALL HGGSLWDGHW
      241 VFWVGPFLAC IFYHAFSFTL PRQEMESVDE N
//
LOCUS       JcXIP2-1 JCGZ_19603 [Jatropha curcas]       303 aa
DEFINITION  JcXIP2-1 JCGZ_19603 [Jatropha curcas]     303 aa
TITLE       JcXIP2-1 JCGZ_19603 [Jatropha curcas]
ORIGIN

        1 MAENLRFVED EENEHGGTKI QPLASTPMPN LDKTEGGKKQ SPTNMNKVLG LEELSSSNVW
       61 RASLAELLGT AALVFAMDTI VISTYETETK TPNLIMSALI AITVTILLIA TSPISHGHIN
      121 PVITFAALFT GRVSLSRAAV YILAQCLGAI LGALALKAVL NSTVEETFSL GGCTLSIVAP
      181 GPHGPILIGL ETDRALWLEI ICTFFFLFSS IWLAFDKRQS TPLGRVIVCC IIGLVVGLLV
      241 FISTTVTAQK GYAGVGMNPA RCLGPALIRG GHLWSGHWVF WVGPIISCIA FGVYVKIIPV
      301 ANA
//
LOCUS       JcSIP1-2 JCGZ_01828 [Jatropha curcas]       235 aa
DEFINITION  JcSIP1-2 JCGZ_01828 [Jatropha curcas]     235 aa
TITLE       JcSIP1-2 JCGZ_01828 [Jatropha curcas]
ORIGIN

        1 MADSILTTMW VFSLPFLGIF TSIIASNIGV EPKSIPALFI AINIATPFVL IFSLIGAALG
       61 GASFNPTTTV SLYAAGLKPD VSLISMAIRF PAQAAGGVFG AKAILQFMPI KYKNFLKGPS
      121 LKVDLHTGAT AEGVLSFVFC LFLLIVLVKG PKNFLVKVWL LAVATVGLVV TGGKYTGPSL
      181 NPANAYGWAY MNNWHNSWEL FYVYWICPLI GATLAAWVFR LLFSFSPPVV KPKQA
//
LOCUS       JcSIP1-3 JCGZ_01827 [Jatropha curcas]       243 aa
DEFINITION  JcSIP1-3 JCGZ_01827 [Jatropha curcas]     243 aa
TITLE       JcSIP1-3 JCGZ_01827 [Jatropha curcas]
ORIGIN

        1 MGLIRVAMAD AILTSLWVFS VPILRVLTTI IASTVGVEPK SLSGLFISIN LSTSFMLIFI
       61 LVGAALGGAS YNPTTTVSLY AAGLKPSGSL SLKTMAVRFP AQAAGGVVGV KAILQAMPRT
      121 YRNLLKGPSL KVDLHTGGLA EGILSFGLCF SLLLVMVRGP KNLWVKIWLV KAATAGLVVI
      181 GGKYTGPCMN PANAYGWAYA NNWHNSWDLF YVYWICPLIG ATSAAWVFRF LFNPTINPKP
      241 KQA
//
LOCUS       JcSIP2-1 JCGZ_14885 [Jatropha curcas]       241 aa
DEFINITION  JcSIP2-1 JCGZ_14885 [Jatropha curcas]     241 aa
TITLE       JcSIP2-1 JCGZ_14885 [Jatropha curcas]
ORIGIN

        1 MGDGSVTSRL IISDFVISFM WVWSGALIKL FVNHVLGLAH QPSGEAINYA LSVIVMFFFA
       61 FLGKITKGGG YNPLTIFSSA ISGDFSQFLL TVGARIPAQV IGSIYGVRLI IETFPEVGRG
      121 PRLNVDIHRG ALTEGVLTFI IVIISLGLAR KIPGSFFRKT WISSISKLAL HILGSDLTGG
      181 CMNPASVMGW AYARGDHITK EHILVYWLAP IEATLLAVWT FKLLVRPKKE EKKEKSEGKS
      241 D
```

//
