# Supplementary material for: Plant Aquaporins: Genome-Wide Identification, Transcriptomics, Proteomics, and Advanced Analytical Tools
Source: Front Plant Sci. 2016 Dec 20;7:1896. doi: 10.3389/fpls.2016.01896 (PMC5167727; doi:10.3389/fpls.2016.01896)
Supplement: Supplementary file 7 [file DataSheet2.DOC]

SourceInteraction Interaction TargetInteraction MutualRank WeightedPCC

Os09g0541000

Os01g0975900 xx Os06g0255000 2.449 0.651119

Os01g0975900 xx Os06g0229000 3.464 0.650254

Os06g0255000 xx Os03g0265800 3 0.622684

Os06g0255000 xx Os05g0394900 3.464 0.621536

Os03g0265800 xx Os01g0343300 2.828 0.670118

Os03g0265800 xx Os07g0447800 4 0.622618

Os05g0394900 xx Os09g0286400 2 0.668516

Os06g0229000 xx Os01g0695200 2 0.680368

Os06g0229000 xx Os03g0729000 3.464 0.633421

Os01g0695200 xx Os01g0966100 2.828 0.657055

Os04g0521100

Os10g0492600 xx Os03g0663800 3.464 0.916723

Os10g0492600 xx Os08g0320900 4.243 0.910300

Os03g0663800 xx Os11g0582400 2 0.944992

Os03g0663800 xx Os03g0842900 2.449 0.935377

Os03g0663800 xx Os08g0320900 2.828 0.924891

Os03g0663800 xx Os05g0140800 4 0.915773

Os03g0663800 xx Os03g0793700 4.472 0.916864

Os11g0582400 xx Os03g0793700 2.828 0.927267

Os11g0582400 xx Os03g0842900 3 0.934225

Os11g0582400 xx Os09g0324000 3.873 0.916362

Os11g0582400 xx Os05g0140800 4.583 0.910953

Os03g0842900 xx Os03g0793700 5 0.916143

Os05g0140800 xx Os09g0370300 2.828 0.906634

Os05g0140800 xx Os05g0569500 4.243 0.895986

Os03g0793700 xx Os10g0542100 4.583 0.908233

Os08g0320900 xx Os03g0793700 3.464 0.917154

Os08g0320900 xx Os03g0842900 3.464 0.924862

Os02g0658100

Os02g0666200 xx Os07g0448800 2.449 0.705684

Os02g0666200 xx Os04g0559700 3.464 0.666678

Os06g0228200 xx Os09g0458900 4.899 0.752378

Os09g0458900 xx Os02g0120600 2 0.834815

Os09g0458900 xx Os06g0691600 3.464 0.808070

Os09g0458900 xx Os08g0499000 3.873 0.778411

Os09g0458900 xx Os02g0128000 4.243 0.778245

Os09g0458900 xx Os07g0605800 4.69 0.753869

Os02g0120600 xx Os06g0125500 2.828 0.809503

Os02g0120600 xx Os05g0488600 3.162 0.797493

Os02g0120600 xx Os01g0910500 3.873 0.811550

Os02g0120600 xx Os06g0321700 4.899 0.787779

Os01g0910500 xx Os06g0321700 2 0.829121

Os06g0691600 xx Os02g0128000 2 0.861905

Os06g0691600 xx Os09g0344900 2.449 0.827755

Os06g0691600 xx Os03g0325500 3.162 0.772798

Os08g0499000 xx Os10g0552400 3.464 0.791918

Os08g0499000 xx Os12g0105800 4 0.757334

Os02g0128000 xx Os02g0490500 4.899 0.701174

Os02g0128000 xx Os09g0344900 4.899 0.760020

Os03g0146100 xx Os02g0760000 2.449 0.740557

Os03g0146100 xx Os05g0574400 3 0.727796

Os03g0146100 xx Os09g0558900 5 0.684807

Os02g0760000 xx Os09g0558900 2 0.761596

Os02g0760000 xx Os05g0574400 3.162 0.733956

Os05g0574400 xx Os07g0571600 3.873 0.704430

Os05g0574400 xx Os02g0664000 4 0.692059

Os05g0574400 xx Os09g0532700 4.472 0.719120

Os09g0532700 xx Os04g0403500 3.742 0.760956

Os09g0532700 xx Os04g0640500 4.243 0.726152

Os09g0532700 xx Os02g0805800 4.583 0.708985

Os09g0532700 xx Os03g0231800 4.69 0.683348

Os09g0558900 xx Os02g0620200 2.828 0.708295

Os09g0558900 xx Os01g0846800 4.899 0.761387

Os02g0620200 xx Os01g0703400 3.162 0.648931

Os02g0620200 xx Os03g0825600 3.464 0.654210

Os02g0620200 xx Os11g0707600 4.899 0.628227

Os01g0846800 xx Os01g0281100 2 0.895476

Os01g0846800 xx Os08g0178800 3.162 0.820287

Os01g0846800 xx Os09g0527900 3.873 0.861757

Os01g0846800 xx Os06g0728700 4.472 0.749077

Os01g0846800 xx Os03g0387900 4.472 0.835169

Os01g0846800 xx Os09g0375900 4.899 0.731229

Os01g0281100 xx Os08g0178800 3.873 0.755241

Os01g0281100 xx Os09g0527900 4.243 0.851349

Os01g0281100 xx Os03g0387900 4.899 0.830381

Os09g0527900 xx Os03g0387900 2.449 0.909608

Os08g0560000 xx Os08g0113000 4.899 0.644643

Os08g0113000 xx Os10g0109600 2.828 0.732163

Os08g0113000 xx Os03g0235000 4.583 0.679197

Os10g0109600 xx Os03g0235000 2 0.764844

Os10g0109600 xx Os01g0185900 3.873 0.738429

Os10g0109600 xx Os07g0677600 3.873 0.704501

Os03g0235000 xx Os07g0542400 3.873 0.657678

Os03g0235000 xx Os03g0225900 4.899 0.636146

Os07g0448800 xx Os01g0658400 2.828 0.698635

Os07g0448800 xx Os05g0582000 2.828 0.713170

Os07g0448800 xx Os04g0559700 4 0.672565

Os07g0448800 xx Os12g0189300 4.899 0.691259

Os01g0658400 xx Os09g0297100 3.464 0.674579

Os09g0297100 xx Os03g0800700 2 0.701960

Os09g0297100 xx Os10g0580200 3.742 0.658545

Os05g0582000 xx Os06g0216700 2 0.724267

Os05g0582000 xx Os02g0594700 4.243 0.653919

Os06g0216700 xx Os02g0594700 4.243 0.651354

Os06g0216700 xx Os05g0410200 4.243 0.685109

Os02g0594700 xx Os02g0594600 2.828 0.638669

Os12g0189300 xx Os01g0700100 4.472 0.747049

Os01g0700100 xx Os05g0220500 2 0.812309

Os01g0700100 xx Os06g0712300 2.449 0.797146

Os05g0231700 xx Os05g0142400 3.873 0.649693

Os05g0231700 xx Os04g0691400 4.899 0.649046

Os05g0142400 xx Os01g0971000 3 0.733380

Os05g0142400 xx Os09g0344900 4 0.733464

Os05g0142400 xx Os06g0500300 4.472 0.731999

Os01g0971000 xx Os01g0810300 2.449 0.751351

Os01g0971000 xx Os10g0148400 3.464 0.703277

Os01g0810300 xx Os10g0330000 2.828 0.752387

Os09g0344900 xx Os06g0500300 3 0.788580

Os09g0344900 xx Os05g0541800 4 0.788396

Os09g0344900 xx Os01g0635200 4.472 0.760987

Os01g0635200 xx Os06g0321700 4 0.774709

Os06g0500300 xx Os05g0541800 2 0.835393

Os06g0500300 xx Os10g0330000 4.899 0.737833

Os10g0330000 xx Os06g0125500 3.464 0.768492

Os04g0691400 xx Os04g0649900 3.162 0.671717

Os04g0649900 xx Os02g0180000 2 0.690443

Os04g0649900 xx Os02g0661900 2.449 0.683041

Os01g0202800

Os07g0448100 xx Os07g0448200 2 0.921959

Os07g0448200 xx Os07g0448400 3 0.597418

Os06g0336200 xx Os04g0671300 2.828 0.623582

Os06g0336200 xx Os12g0113500 3.742 0.662639

Os12g0113500 xx Os11g0113700 2 0.887920

Os12g0113500 xx Os03g0850400 2.449 0.756572

Os12g0113500 xx Os04g0640900 3.464 0.754012

Os12g0113500 xx Os03g0823400 4.472 0.687363

Os12g0113500 xx Os07g0246600 4.69 0.646603

Os11g0113700 xx Os04g0640900 2.449 0.765530

Os11g0113700 xx Os03g0850400 3.464 0.742929

Os11g0113700 xx Os03g0823400 3.464 0.704384

Os11g0113700 xx Os02g0136000 3.873 0.720644

Os03g0850400 xx Os09g0294000 3.162 0.721188

Os03g0850400 xx Os04g0640900 4 0.733309

Os03g0850400 xx Os10g0483500 4 0.635646

Os03g0850400 xx Os02g0749300 4.899 0.679900

Os04g0640900 xx Os02g0749300 3.162 0.702290

Os03g0823400 xx Os02g0749300 3 0.691028

Os03g0823400 xx Os07g0187700 4.243 0.641355

Os01g0182200 xx Os02g0317200 2.449 0.602845

Os01g0182200 xx Os10g0487400 3.464 0.585086

Os02g0317200 xx Os02g0318200 2.828 0.582459

Os02g0318200 xx Os06g0224100 4.472 0.535906

Os10g0487400 xx Os09g0559800 4.69 0.592194

Os09g0559800 xx Os05g0350900 2.449 0.657587

Os09g0559800 xx Os05g0356700 3.464 0.683212

Os09g0559800 xx Os01g0801500 4.899 0.650251

Os01g0866000 xx Os06g0665800 3.742 0.653167

Os06g0665800 xx Os01g0290600 2 0.703349

Os06g0665800 xx Os05g0168500 4.243 0.702203

Os01g0290600 xx Os01g0856500 3.464 0.644564

Os05g0168500 xx Os03g0413400 2 0.758321

Os05g0168500 xx Os09g0557800 2.449 0.735404

Os05g0168500 xx Os03g0237000 2.828 0.734995

Os05g0168500 xx Os06g0132100 3.873 0.734186

Os05g0168500 xx Os01g0631100 4.583 0.690272

Os05g0168500 xx Os01g0856500 4.899 0.656628

Os03g0413400 xx Os06g0132100 4.472 0.686819

Os09g0557800 xx Os06g0132100 3.464 0.694648

Os03g0861300

Os01g0112400 xx Os06g0237400 3.464 0.779234

Os01g0112400 xx Os07g0556800 3.464 0.790963

Os01g0112400 xx Os12g0242700 4.243 0.812443

Os01g0112400 xx Os04g0267000 4.583 0.761759

Os06g0237400 xx Os03g0212000 4.472 0.773059

Os03g0212000 xx Os08g0163800 2.828 0.775636

Os07g0556800 xx Os07g0144900 4.243 0.802277

Os07g0556800 xx Os01g0641200 4.583 0.771707

Os07g0556800 xx Os12g0242700 4.69 0.804749

Os07g0144900 xx Os07g0499900 2.828 0.844553

Os07g0144900 xx Os01g0641200 4.899 0.781516

Os12g0242700 xx Os10g0484800 2 0.873451

Os12g0242700 xx Os05g0428600 3.464 0.830960

Os12g0242700 xx Os12g0427000 4.899 0.871976

Os10g0484800 xx Os04g0573100 3 0.832143

Os10g0484800 xx Os01g0127500 3.873 0.784387

Os10g0484800 xx Os06g0611400 4.899 0.758417

Os05g0428600 xx Os12g0427000 4.899 0.835021

Os12g0427000 xx Os08g0413000 2.449 0.909153

Os12g0427000 xx Os11g0593500 2.828 0.886038

Os12g0427000 xx Os05g0577900 3.464 0.898362

Os12g0427000 xx Os12g0589400 3.873 0.881595

Os12g0427000 xx Os11g0594400 4.243 0.864003

Os11g0593500 xx Os05g0577900 4.899 0.861401

Os05g0577900 xx Os12g0589400 3.464 0.890319

Os04g0267000 xx Os03g0212000 3.464 0.766261

Os04g0233400 xx Os06g0179000 4.899 0.709898

Os06g0179000 xx Os01g0758700 3 0.729730

Os01g0232100 xx Os06g0317200 2 0.644126

Os03g0320000 xx Os11g0575900 2 0.548045

Os11g0575900 xx Os12g0603700 5 0.508034

Os12g0603700 xx Os06g0607000 3.464 0.559781

Os04g0550800

Os02g0629200 xx Os11g0167800 2.449 0.836870

Os02g0629200 xx Os06g0143100 2.449 0.840881

Os02g0629200 xx Os07g0634400 3.742 0.782784

Os02g0629200 xx Os02g0722400 4.472 0.787459

Os11g0167800 xx Os04g0678200 2.449 0.830221

Os11g0167800 xx Os01g0166100 2.828 0.821948

Os11g0167800 xx Os02g0131100 3.162 0.808471

Os04g0678200 xx Os07g0142000 3 0.824626

Os04g0678200 xx Os02g0131100 4 0.793252

Os07g0142000 xx Os03g0165300 2.828 0.832274

Os01g0166100 xx Os01g0935400 3.464 0.780957

Os01g0166100 xx Os01g0948600 3.742 0.769856

Os01g0166100 xx Os11g0158600 3.873 0.814306

Os01g0166100 xx Os02g0131100 3.873 0.797870

Os01g0166100 xx Os03g0124500 4.472 0.761270

Os11g0158600 xx Os03g0165300 3.162 0.828958

Os02g0131100 xx Os07g0211900 3.162 0.786091

Os06g0143100 xx Os04g0660100 2 0.843388

Os06g0143100 xx Os05g0126100 2.828 0.837046

Os06g0143100 xx Os02g0711400 3.464 0.801023

Os06g0143100 xx Os01g0166100 4.472 0.813628

Os04g0660100 xx Os04g0533500 2.828 0.811065

Os04g0660100 xx Os05g0126100 3.873 0.806428

Os04g0660100 xx Os03g0165300 4.243 0.812268

Os04g0660100 xx Os02g0740700 4.472 0.790339

Os04g0660100 xx Os03g0843100 4.583 0.796651

Os04g0660100 xx Os10g0442800 4.899 0.795102

Os05g0126100 xx Os02g0734300 3.873 0.782080

Os05g0126100 xx Os07g0211900 4.243 0.774867

Os07g0634400 xx Os06g0239500 2.449 0.773779

Os07g0634400 xx Os09g0494600 2.828 0.765099

Os07g0634400 xx Os02g0191800 3.464 0.755361

Os07g0634400 xx Os02g0174800 4.243 0.737938

Os07g0634400 xx Os05g0344400 4.472 0.737937

Os07g0634400 xx Os06g0214800 4.899 0.727835

Os06g0239500 xx Os02g0174800 3.464 0.729517

Os06g0239500 xx Os07g0471900 4 0.716407

Os09g0494600 xx Os06g0210000 2.828 0.727358

Os09g0494600 xx Os02g0191800 3.464 0.728481

Os09g0494600 xx Os03g0858800 4.243 0.696366

Os09g0494600 xx Os02g0809800 4.899 0.688060

Os03g0858800 xx Os02g0809800 4.899 0.657307

Os02g0191800 xx Os05g0550300 3.464 0.738217

Os02g0191800 xx Os03g0858800 3.742 0.709696

Os02g0174800 xx Os07g0471900 2.449 0.730529

Os05g0344400 xx Os12g0119800 3.162 0.684160

Os02g0722400 xx Os11g0275200 2.828 0.827556

Os02g0722400 xx Os03g0856000 4.899 0.798734

Os11g0275200 xx Os02g0590400 2 0.857731

Os11g0275200 xx Os05g0178300 2.449 0.840073

Os11g0275200 xx Os01g0168800 3.162 0.822570

Os11g0275200 xx Os08g0238600 4.243 0.809874

Os11g0275200 xx Os03g0184300 4.583 0.801707

Os11g0275200 xx Os08g0239000 4.69 0.763478

Os02g0590400 xx Os05g0178300 3 0.812895

Os03g0856000 xx Os03g0305800 2 0.856408

Os03g0856000 xx Os11g0121300 3 0.812649

Os03g0856000 xx Os03g0393700 3.464 0.807184

Os03g0856000 xx Os04g0605300 3.873 0.803146

Os03g0856000 xx Os09g0455900 4.69 0.781858

Os03g0305800 xx Os04g0605300 2.449 0.823174

Os01g0202900

Os02g0823100 xx Os01g0826000 2.449 0.727570

Os02g0823100 xx Os08g0101500 2.828 0.684374

Os01g0826000 xx Os01g0935400 2.449 0.777404

Os08g0101500 xx Os05g0168200 3.162 0.635361

Os02g0745100

Os04g0527900

Os02g0232900

Os01g0232000

Os10g0513200 xx Os07g0617700 4.472 0.676674

Os07g0617700 xx Os06g0680500 2 0.772515

Os07g0617700 xx Os02g0688800 2.828 0.715236

Os07g0617700 xx Os12g0577600 3.873 0.741740

Os07g0617700 xx Os04g0442000 5 0.703702

Os06g0680500 xx Os12g0577600 2.449 0.770329

Os06g0680500 xx Os05g0332300 4.243 0.723515

Os06g0680500 xx Os07g0562800 4.472 0.742992

Os06g0680500 xx Os02g0649400 4.472 0.704126

Os07g0562800 xx Os12g0152800 4.472 0.742348

Os02g0688800 xx Os05g0354400 3.464 0.641284

Os12g0577600 xx Os12g0152800 3.464 0.748038

Os12g0577600 xx Os12g0569900 3.464 0.734010

Os12g0569900 xx Os11g0158500 4.899 0.712109

Os04g0442000 xx Os11g0158500 2.449 0.747506

Os08g0559000 xx Os03g0841700 4.899 0.572383

Os03g0841700 xx Os11g0216000 2.828 0.699040

Os11g0216000 xx Os02g0580500 2 0.715680

Os11g0216000 xx Os05g0331200 2.449 0.708305

Os11g0216000 xx Os01g0276700 4.583 0.613159

Os11g0216000 xx Os07g0490800 5 0.652863

Os12g0204100

Os01g0219000

Os05g0205000 xx Os03g0663400 3 0.683828

Os03g0663400 xx Os11g0592000 2.828 0.687528

Os03g0663400 xx Os01g0925400 3.742 0.653821

Os11g0592000 xx Os11g0592100 2 0.774821

Os11g0592000 xx Os10g0491000 3.464 0.724572

Os11g0592000 xx Os12g0630500 4 0.673304

Os11g0592000 xx Os11g0592200 4.583 0.674269

Os01g0925400 xx Os08g0157000 4.472 0.631691
